# Stereoselective Synthesis and Antiproliferative Activity of Steviol-Based Diterpene 1,3-Aminoalcohol Regioisomers

**DOI:** 10.3390/molecules28247962

**Published:** 2023-12-05

**Authors:** Dorottya Bai, Zsuzsanna Schelz, Mária Fanni Boncz, István Zupkó, Zsolt Szakonyi

**Affiliations:** 1 Interdisciplinary Excellence Center, Institute of Pharmaceutical Chemistry, University of Szeged, Eötvös utca 6, H-6720 Szeged, Hungary; baidorottya@gmail.com (D.B.); bonczmaria@gmail.com (M.F.B.); 2 Institute of Pharmacodynamics and Biopharmacy, University of Szeged, Eötvös utca 6, H-6720 Szeged, Hungary; schelz.zsuzsanna@szte.hu (Z.S.); zupko.istvan@szte.hu (I.Z.); 3 Interdisciplinary Centre of Natural Products, University of Szeged, Eötvös utca 6, H-6720 Szeged, Hungary

**Keywords:** diterpene, steviol, aminoalcohol, stereoselective, regioisomer, antiproliferative activity

## Abstract

A series of novel diterpene-type 1,3-aminoalcohols and their regioisomers have been synthesised from natural stevioside in a stereoselective manner. The key intermediate β-keto alcohol was prepared using Wagner–Meerwein rearrangement of the epoxide derived from steviol methyl ester. The primary aminoalcohol was formed via Raney-nickel-catalysed hydrogenation of an oxime, and a versatile library of aminoalcohols was synthesised using a Schiff base with the primary amines. The aminoalcohol regioisomers were prepared from the mesylate of the β-keto alcohols. The corresponding primary aminoalcohol was formed via the palladium-catalysed hydrogenation of hydroxyl-azide, and click reactions of the latter were also carried out. The new compounds were characterised using 1D- and 2D-NMR techniques and HRMS measurements. The in vitro investigations showed high inhibition of cell growth in human cancer cell lines (HeLa, SiHa, A2780, MCF-7 and MDA-MB-231) in the case of naphthalic *N*-substituted derivatives. The antiproliferative effects were assayed using the MTT method.

## 1. Introduction

In recent decades, the development of bioactive terpene-based aminoalcohols has earned major scientific attention [1,2,3,4]. Terpenoids are a large and structurally multifaceted class of natural products consisting of isoprene units, present in most plants. Several monoterpenes, such as camphor, (+)-pulegone [2], as well as α- and β-pinene [5,6,7], have proved to be excellent sources for the preparation of various alicyclic 1,3-aminoalcohols, which may serve as catalysts and chiral auxiliaries in enantioselective reactions, as well as building blocks in the asymmetric syntheses of potential pharmaceutical agents, e.g., esomeprasol [8,9,10,11]. Numerous studies have confirmed the wide array of pharmacological properties of these compounds. Furthermore, their heterocyclic derivatives display, among other things, antifungal, antimicrobial, BACE1-inhibiting [12] and antiproliferative action on a panel of human cancer cell lines [8,9,13]. Terpenes play an important role in depolarising the membrane of cancer cells and, in particular, the membrane of mitochondria, activating apoptosis via caspases or inactivating the PI3K/Akt/NF-κB pathway, along with the inhibition of angiogenesis [14].

Small-molecule drugs with an 1,3-aminoalcohol moiety, such as clofedanol, phenoperidine and precursors of fluoxetine and atomoxetine, are well known in applied medicine [15,16,17], while only limited knowledge is available on diterpenoids with functional groups. Among a large number of diterpenes, stevioside (**I**), with a complex *ent*-kaurane skeleton and three glucose moieties, is regularly exerted as the starting material of biologically active derivatives. The glycoside can be isolated at an industrial scale from the perennial herbal shrub *Stevia rebaudiana* [18] and it can be transformed into its aglycons, steviol and isosteviol, which exhibit a series of pharmacological effects themselves, e.g., antihypertensive, antihyperglycemic, antibacterial, anti-inflammatory and antitumor activities [19]. Combining the benefits of diterpenes and aminoalcohols could unlock the path to new cytostatic compounds.

**Figure 1:**
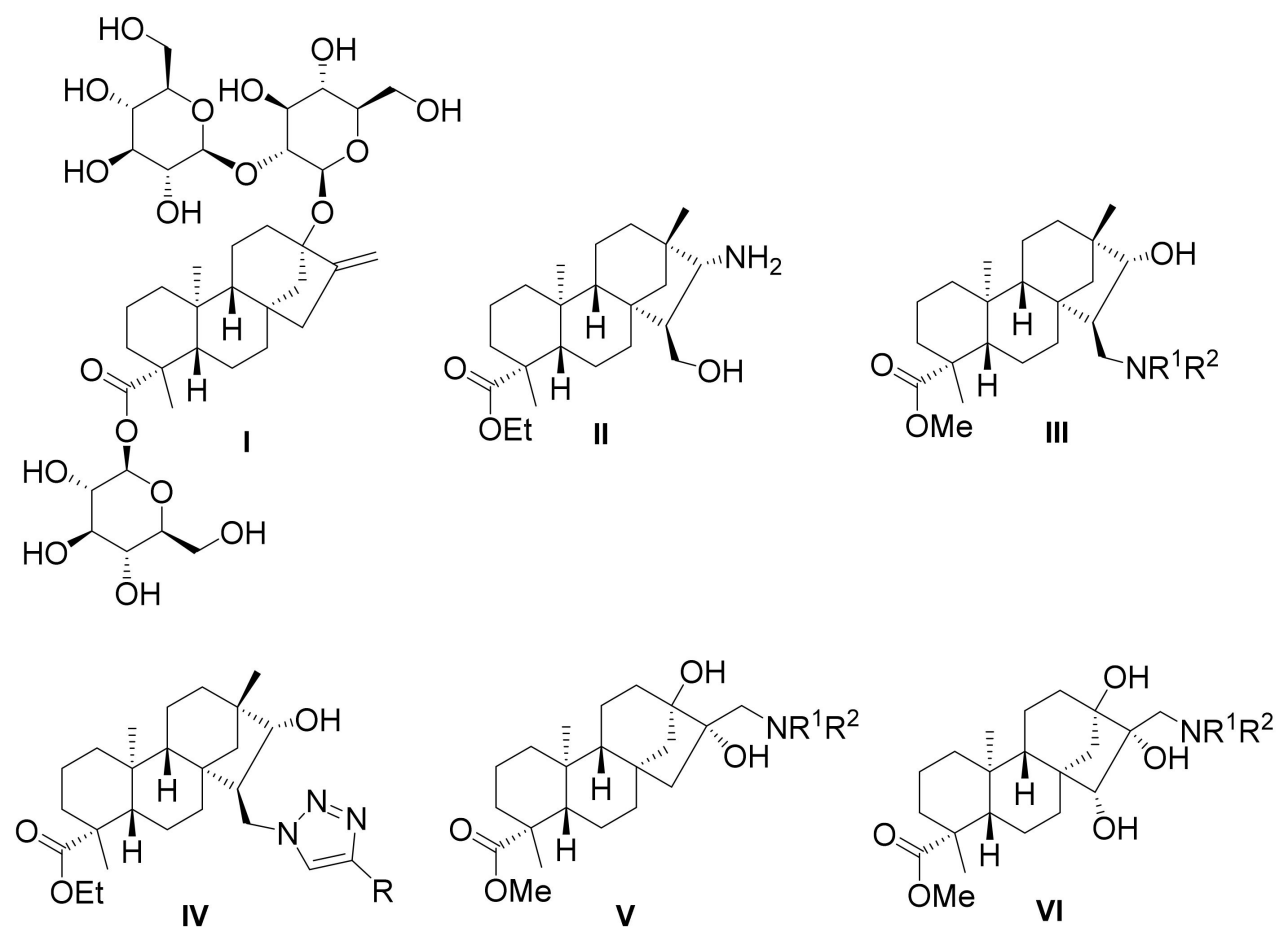
*Ent*-kaurane and *ent*-beyerane type compounds with biological activity derived from stevioside.

Aminoalcohols (**II**, **III**) and heteroaromatic derivatives (**IV**), as well as aminodiols (**V**) and, in our forthcoming work, aminotriols (**VI**), with antiproliferative activity derived from both steviol and isosteviol via stereoselective transformations have been studied previously (Figure 1) [19,20,21,22,23,24]. The aromatic *N*-substituted derivatives exhibited remarkable pharmacological effects in all cases. Therefore, to expand our knowledge on this family of diterpenes, our aim was to extend this class of compounds via the synthesis of novel 1,3-aminoalcohols from stevioside (**I**), to study the effect of the regioisomeric position of the aminoalcohol moiety on the antiproliferative activity and compare it to that of the former analogues.

Herein, we report the stereoselective synthesis of new chiral bifunctional compounds, such as 1,3-aminoalcohol and heterocyclic derivatives with 1,2,3-triazole moiety, starting from steviol. Furthermore, we also disclose our findings with respect to their antiproliferative activity on human cancer cell lines and the effect of various *N*-substituted amines on their bioactivity to decode the structure-activity relationships.

## 2. Results and Discussion

### 2.1. Synthesis of Key Intermediate β-Keto Alcohol ***4***

**Scheme 1:**
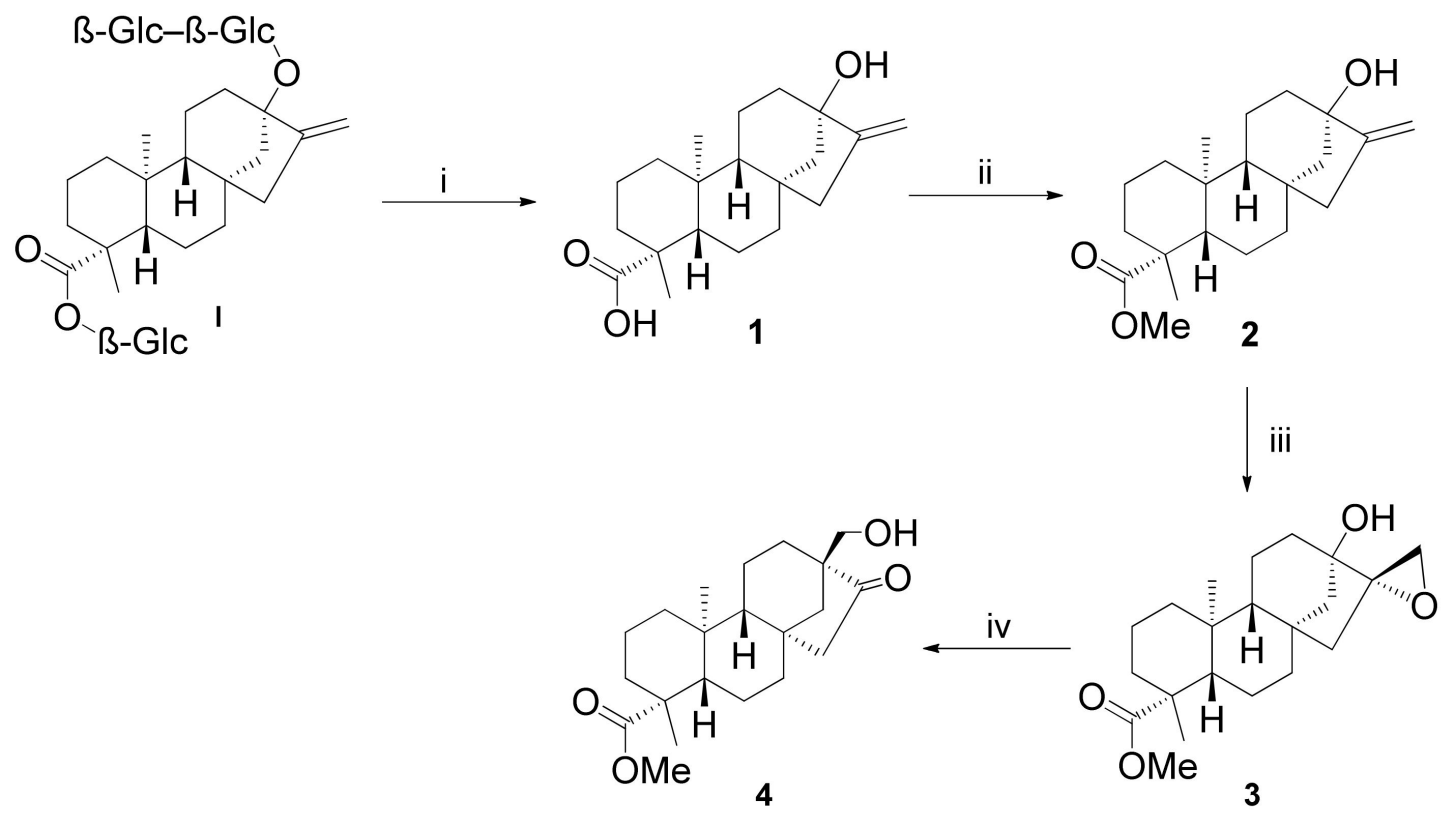
Stereoselective synthesis of key intermediate β-keto alcohol **4** from stevioside. (i) (1) NaIO_4_, H_2_O, 16 h, 25 °C; (2) KOH, H_2_O, 1 h, 100 °C, 57%. (ii) CH_2_N_2_, Et_2_O, 2 min, 25 °C, 86%. (iii) *t*-BuOOH, VO(acac)_2_, anhydrous toluene, 30 min, 0 °C, 1 h, 25 °C, 72%. (iv) BF_3_·Et_2_O, anhydrous toluene, 1 h, 25 °C, 88%.

Steviol (**1**) was synthesised from the commercially available natural glycoside stevioside (**I**) in a two-step reaction as described in the literature [25,26]. For esterification of the steviol, diazomethane was used, resulting in methyl ester **3** in mere minutes without observation of cyclopropanation as a side reaction (Scheme 1) [25]. The epoxidation of methyl ester **3** was carried out with *t*-BuOOH as an oxidising agent and vanadyl acetylacetonate (VO(acac)_2_) as the catalyst, a method applied in our previous studies [23,24]. According to Van Speybroeck et al., the concerted Sharpless mechanism is preferred for alkylperoxo species from which V^+IV^O(L)(OO*t*Bu) and V^+V^O(L_1_)(L_2_)(OO*t*Bu) are the most abundant. Throughout the process, the oxidation state of vanadium changes periodically in a catalytic cycle between the +IV and +V oxidation levels, which causes the mixture to change its colour from deep red to amber, aiding us in tracking the progress and the completion of the reaction [27].

**Scheme 2:**
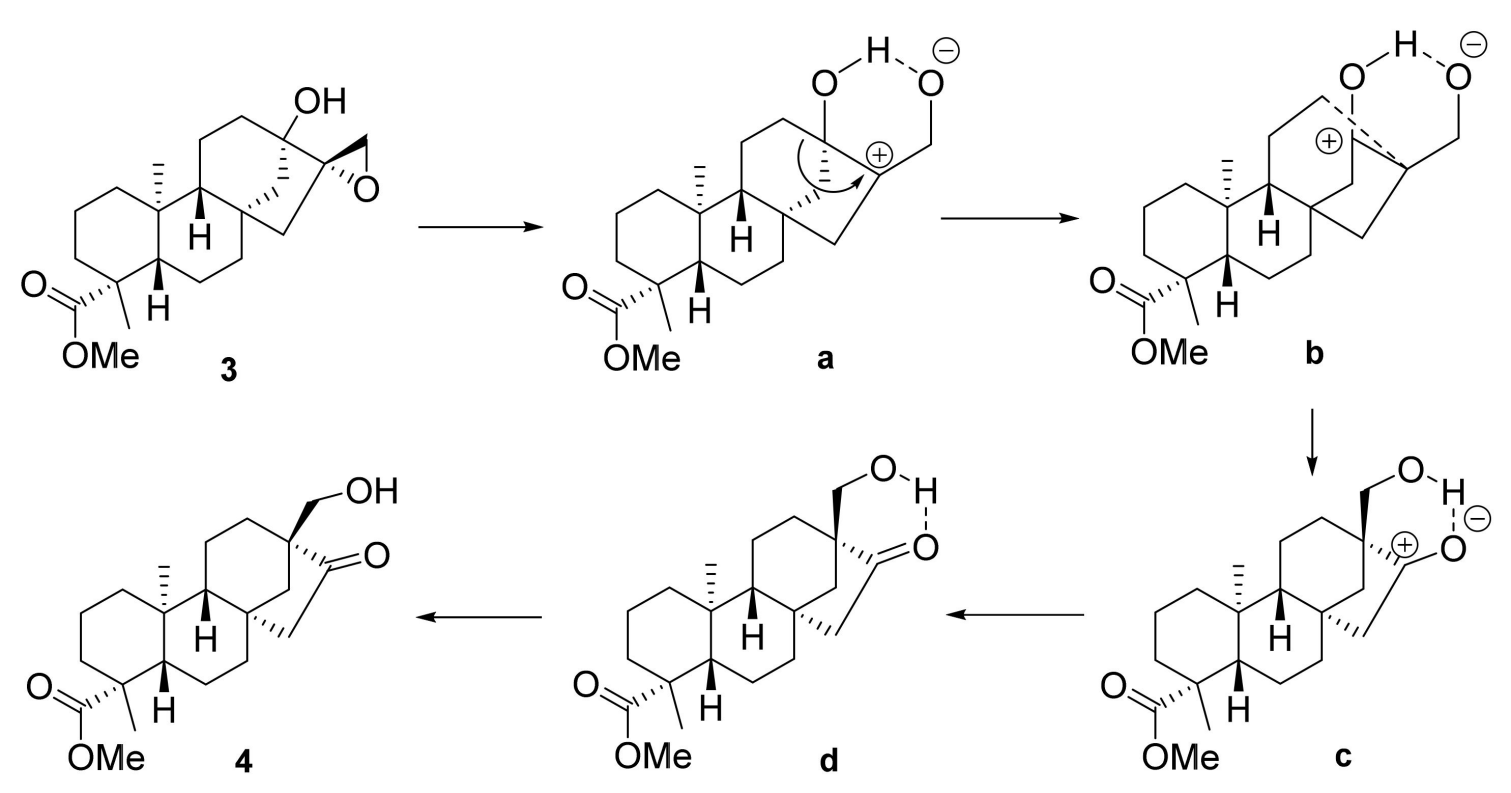
Proposed mechanism through intermediates (**a**–**d**) for Wagner–Meerwein rearrangement of compound **3**.

For the transformation of epoxide **3**, it was treated with BF_3_·Et_2_O in anhydrous toluene, and the rearrangement was completed in an hour at room temperature [28]. Consequently, 2D-NMR spectroscopy confirmed the change in stereochemistry in the structure, where the skeleton was converted from *ent*-kaurane into isosteviol-type *ent*-beyerane [29]. The mechanism of the reaction is similar to that described by Schreiber et al. for 8*S*,15-epoxy-gibberellic acid. On the basis of their findings, we propose the following process (Scheme 2). Via coordination of the Lewis acid to the oxygen of the epoxide, the oxirane ring opens up, and through interaction with the neighbouring hydroxy group, a six-membered ring is built (**a**). The carbonium ion is stabilised via Wagner–Meerwein rearrangement, and the bond between C-12 and C-13 breaks, while a new bond is created between C-12 and C-16 (**b**). The semipolar bond of the carbonyl function (**d**) is created due to the displacement of the negative charge over the hydrogen bridge (**c**) [30].

### 2.2. Synthesis of 1,3-Aminoalcohol Derivatives ***6***–***17***

**Scheme 3:**
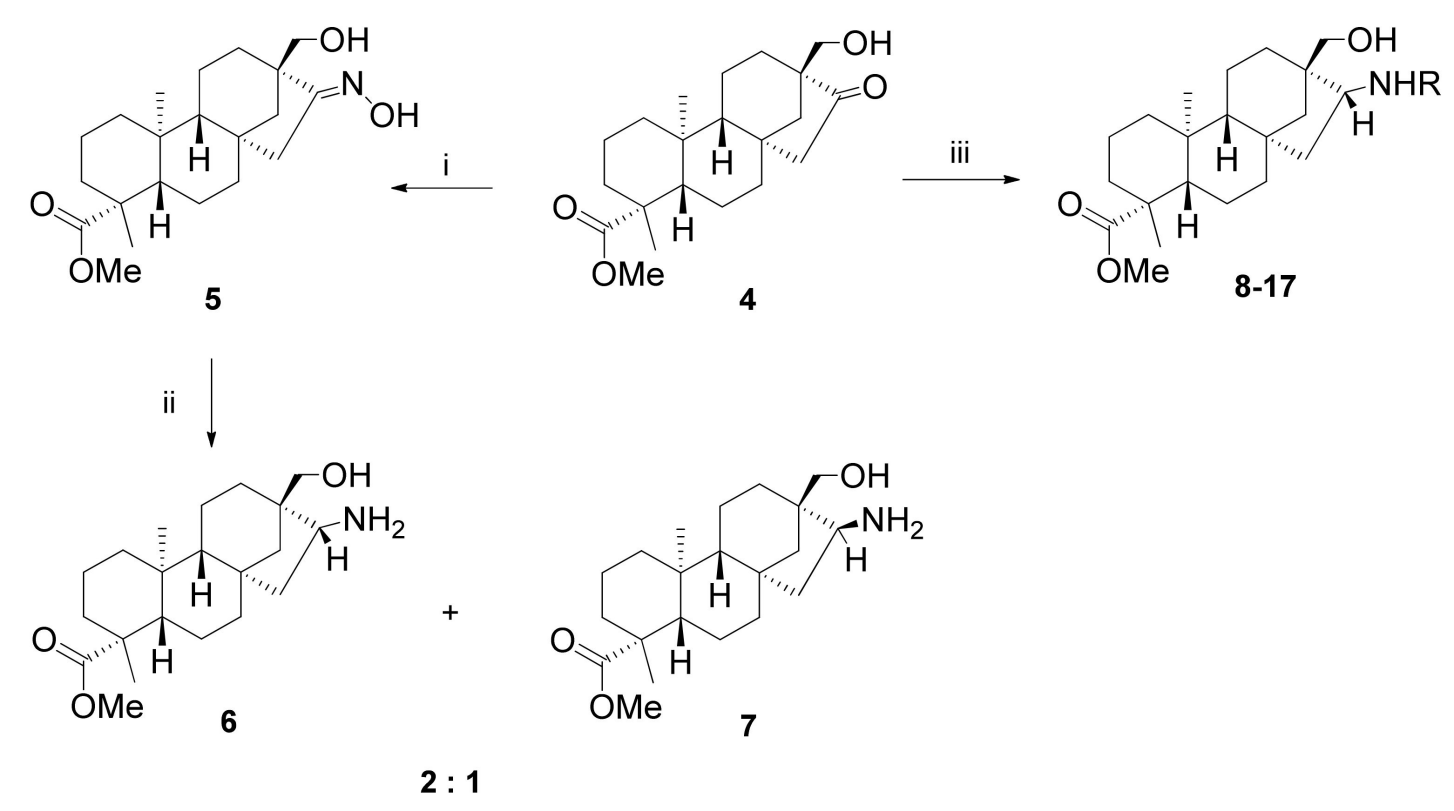
Stereoselective synthesis of aminoalcohols **6**–**17**. (i) NH_2_OH·HCl, NaHCO_3_·EtOH, 4 h, 60 °C, 72%; (ii) Raney Ni, H_2_ (10 atm), anhydrous THF, 1 day, 25 °C, 51% overall yield; (iii) (1) NH_2_R, BF_3_·Et_2_O, anhydrous toluene, 24 h, 130 °C, (2) NaBH_4_, anhydrous MeOH, 1 h, 0 °C, 41–78%.

Compound **4** was first submitted to oximation with hydroxylamine hydrochloride in the presence of NaHCO_3_ in ethanol (**5**) [31], and then the product was converted into primary aminoalcohols **6** and **7** using hydrogenation catalysed by Raney Ni in THF (Scheme 3) [21]. Diastereomers **6** and **7** were obtained in a 2:1 ratio and they could be successfully separated using preparative column chromatography. The nucleophilic addition of amines to carbonyl compounds followed by dehydration is a convenient way to prepare enamines [21,32]. The reaction was accomplished in the presence of a molecular sieve to remove water from the system. The addition of a Lewis acid catalyst such as BF_3_·Et_2_O induced the interaction with the nucleophilic amines via the adducts formed with oxygen, and improved the reaction time. Reduction of the resulting Schiff bases with NaBH_4_ provided the corresponding novel 1,3-aminoalcohols **8**–**17** in moderate to good yields (Table 1).

### 2.3. Synthesis of 1,3-Aminoalcohol Regioisomers ***22***–***30*** and Preparation of 1,2,3-Triazole Derivatives via a Click Reaction

**Scheme 4:**
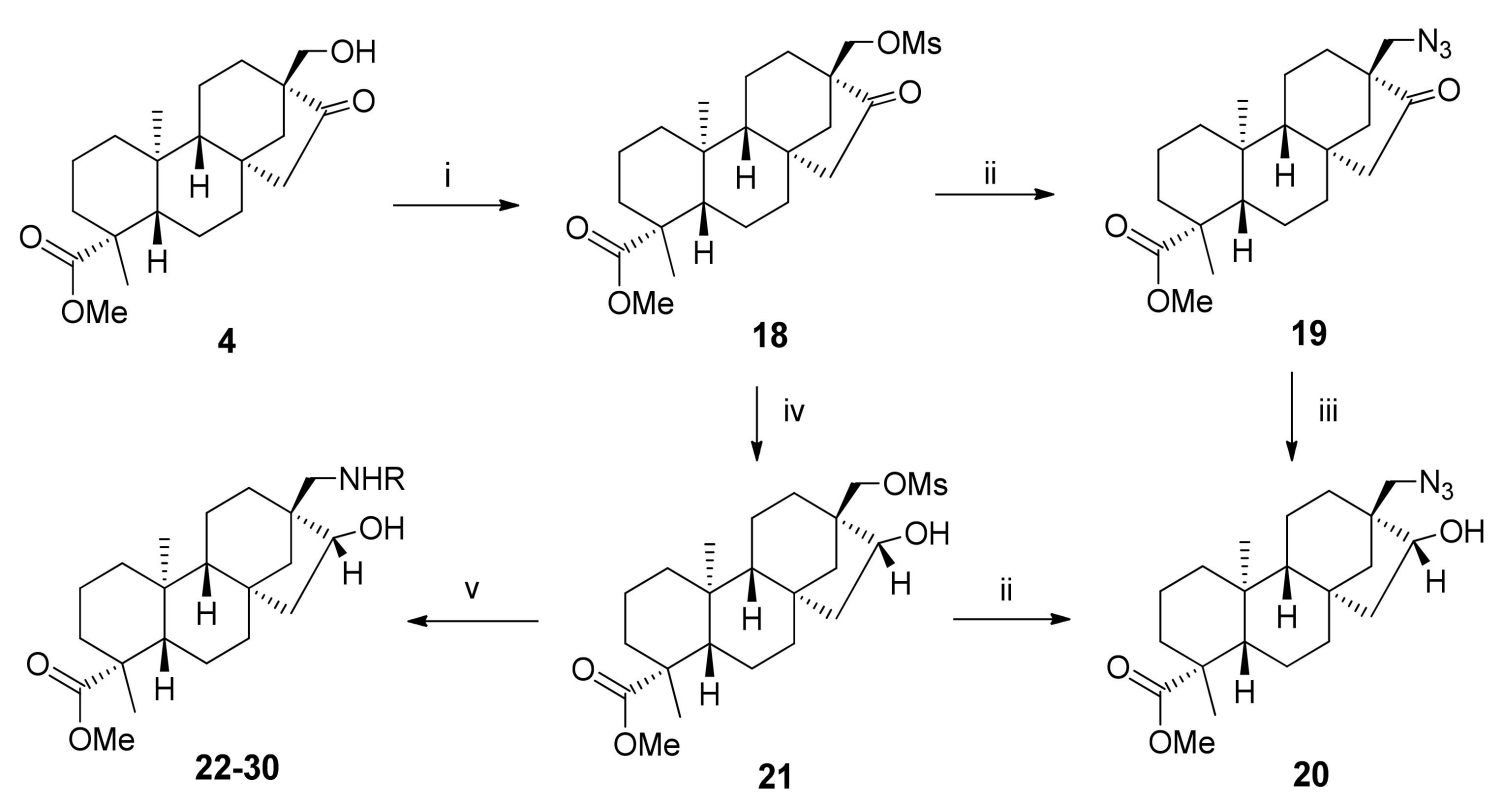
Azide synthesis and preparation of aminoalcohol regioisomers **22**–**30**. (i) MsCl, anhydrous pyridine, 24 h, 25 °C, 68%; (ii) NaN_3_, anhydrous DMF, 24 h, 80 °C, 65% (**19**), 80% (**20**); (iii) NaBH_4_, anhydrous MeOH, 1 h, 0 °C, 86%; (iv) NaBH_4_, MeOH/CH_2_Cl_2_ = 1:1, 1 h, 0 °C, 88%; (v) NH_2_R, TEA/MeCN = 1:1, 4 days, 80 °C, 15–44%.

The versatile β-keto alcohol was further modified with the intention of synthesising the regioisomeric analogue of the prepared aminoalcohols. First, the hydroxy group was changed into mesylate, a better leaving group, using methanesulfonyl chloride in dry pyridine (Scheme 4) [33]. In the following steps, the O-mesyl function was converted into azide (**19**) with sodium azide in anhydrous DMF, and the ketone was transformed into alcohol **20** via reduction with NaBH_4_ [34]. Azide **20** was also prepared in an alternative pathway starting from **18**, to optimise the overall yield (Scheme 4). To decrease the reactivity of the mesylate in the presence of hydride ions, a 1:1 ratio of MeOH and dichloromethane was used instead of DMF. With the steps switched, the process proved to be slightly more effective, and hydroxy-mesylate derivative **21** served as the starting material for the preparation of aminoalcohols **22**–**30** (Table 2). For comparison, nucleophilic substitution was accomplished with the same selection of *N*-substituted primary amines as before, in acetonitrile and triethylamine in a 1:1 ratio. This was determined experimentally to maximise the yield and minimise the development of side products [35].

**Scheme 5:**
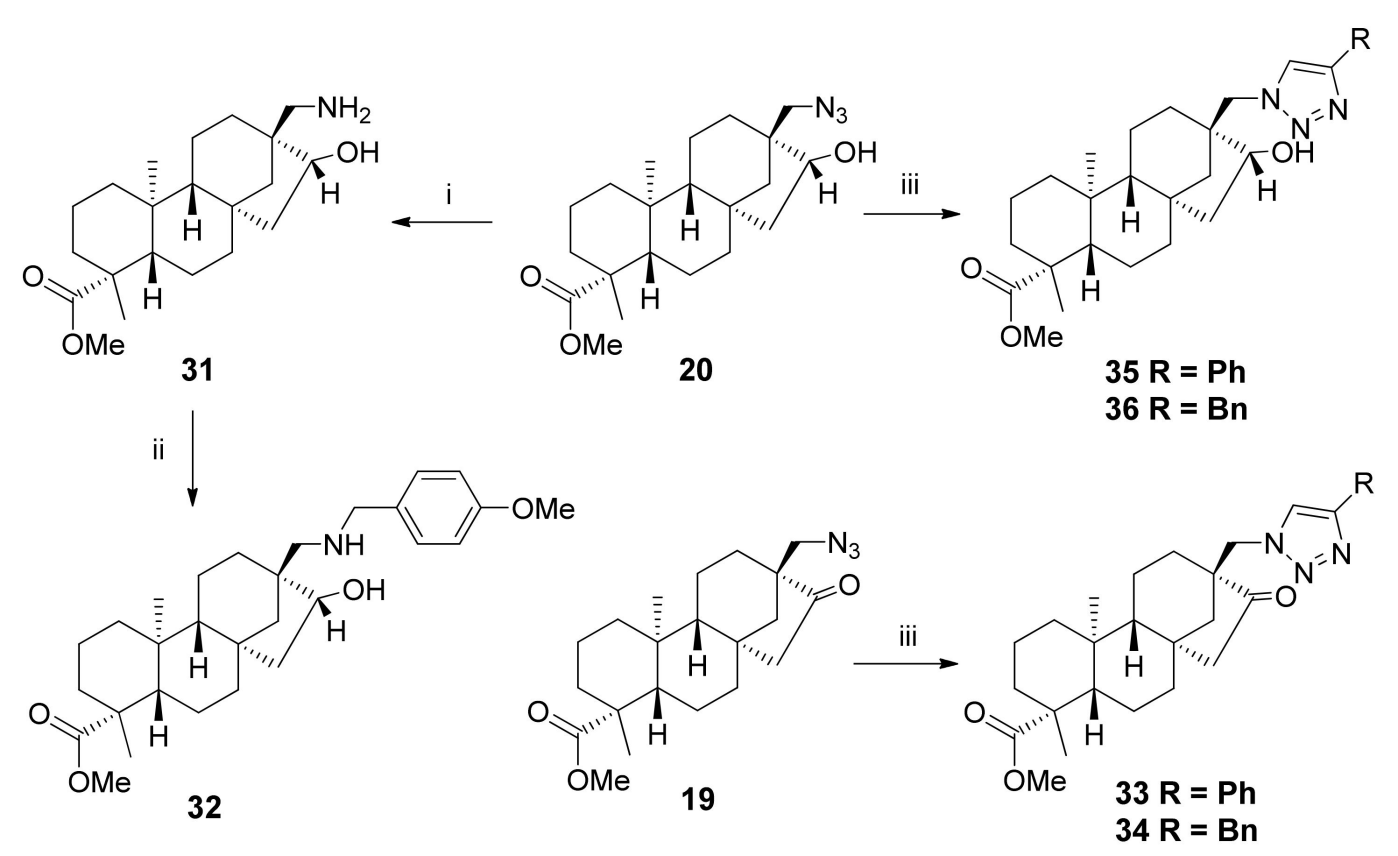
Synthesis of 4-methoxybenzyl-substituted derivative **34** using primary aminoalcohol and a click reaction. (i) Pd/C, H_2_ (1 atm), MeOH, 1 day, 25 °C, 66%; (ii) (1) 4-methoxybenzaldehyde, anhydrous MeOH, 5 h, 25 °C, (2) NaBH_4_, anhydrous MeOH, 1 h, 0 °C 15%; (iii) Alkyne, CuSO_4_.5H_2_O, sodium ascorbate, *t*-BuOH/H_2_O = 2:1, 2 days, 25 °C, 42–67%.

In the case of 4-methoxybenzylamine, despite testing different conditions, no product could be observed using TLC. Thus, we synthesised the desired derivative using primary aminoalcohol **31**, which was prepared via palladium-catalysed hydrogenation of hydroxyl-azide **20** in methanol (Scheme 5). Compound **31** was then treated with 4-methoxybenzaldehyde to form the Schiff base, followed by reduction without isolation using NaBH_4,_ resulting in aminoalcohol **32** [21].

We were also interested in the synthesis of heteroaromatic derivatives due to their widely acclaimed biological activity. Moreover, the heterocyclic unit is beneficial for decreasing the overall lipophilicity of the diterpene, thereby improving the ADME parameters [23,36]. Starting with keto-azide **19**, it was coupled with aromatic alkynes in *t*-BuOH/H_2_O = 2:1 medium for better solubility of the catalysts CuSO_4_·5H_2_O and sodium ascorbate, generated in situ from ascorbic acid and NaOH in methanol. The reaction was repeated using hydroxyl-azide **20** as the starting material, forming compounds **35** and **36** in good yields (Scheme 5) [37,38].

### 2.4. In Vitro Antiproliferative Studies of Steviol-Based Aminoalcohols and Structure–Activity Relationship

**Figure 2:**
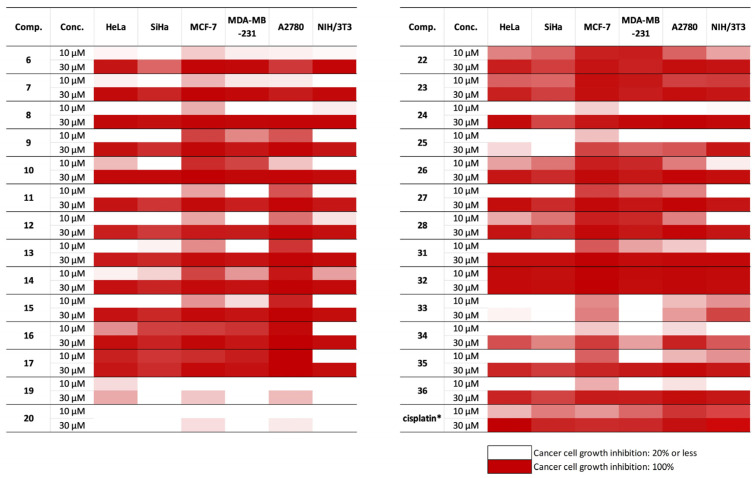
Antiproliferative properties of the prepared steviol analogues against cancer cells and NIH/3T3 fibroblasts. The numeric values are given in Table S1 in the Supplementary Materials. *: data from reference [24].

The antiproliferative properties of the prepared steviol-based 1,3-aminoalcohol analogue derivatives were determined against human cancer cell lines of gynaecological origin. Generally, the MCF-7 breast cancer and A2780 ovarian cancer cell lines seemed more sensitive than the cell lines from cervical (HeLa and SiHa) and triple-negative breast cancer MBA-MD-231 (Figure 2 and Table S1 in the Supplementary Materials). Most molecules exerted relevant action against the non-malignant fibroblasts (NIH/3T3), indicating the limited selectivity of the tested substances. Compounds with an azido function at the diterpene skeleton (**19**, **20**) elicited no substantial action even at higher concentration (30 μM). The aromatic substituent on the diterpene core increased the biological effect, since only compounds with a primary amino function (**6**, **7**) caused pronounced cell growth inhibition at 30 μM. However, the regioisomeric analogue **31** was more active, especially against the MCF-7 cells. Incorporating the originally basic nitrogen into a triazole ring (**33**–**36**) resulted in analogues with poor or modest activities without considerable cancer selectivity. Concerning the analogues with benzyl or naphthyl moieties, the connection between these aromatic rings and the diterpene skeleton proved relevant. Significant differences in antiproliferative activity between regioisomers was observed. Molecules in which the aromatic building block was connected to the core via a methylene linker tended to elicit higher activities than analogues connected directly (e.g., **8**, **13**, **14**, **15**, and **22**, **27**, **28**, **26**, respectively). The stereochemistry of the nitrogen had no substantial impact on the antiproliferative effects (**13** and **14**, **27** and **28**, or **16** and **17**). Some of the presented compounds exhibited more pronounced growth-inhibitory actions than the reference agent cisplatin [39]. In the case of the most active agents, the assays were repeated by applying a range of concentrations (0.1–30 μM). Molecules **23** and **32** exerted outstanding activities against both the cancer cells and fibroblasts (IC_50_ values for **23**: 1.59–5.15 µM, for **32**: 1.04–5.77 µM). Compound **16**, on the other hand, had similar effects on the malignant cells (IC_50_ values: 3.44–6.33 µM) with limited action on the fibroblasts (IC_50_ value: 17.44 µM), indicating considerable cancer selectivity. Since this latter agent seems to be superior to the clinically utilised cisplatin, it could be regarded as a potential hit compound and may be subjected to further investigation.

## 3. Materials and Methods

### 3.1. General Methods

The commercially available reagents were used as obtained from the suppliers (Novochem Co., Ltd., 1089 Budapest, Hungary, Orczy út 6.; Merck Ltd., Budapest, Hungary and VWR International Ltd., Debrecen, Hungary), while the solvents were dried according to the standard procedures. The ^1^H-, ^13^C *J*-MOD and ^19^F-NMR spectra were recorded using a Bruker Avance DRX-500 spectrometer (Bruker Biospin, Karlsruhe, Baden-Württemberg, Germany) [500 MHz (^1^H), 125 MHz (^13^C *J*-MOD) and 470 MHz (^19^F) δ = 0 (TMS)]. Chemical shifts are expressed in ppm (δ) relative to TMS as an internal reference. *J* values are given in Hz. All the ^1^H-, ^13^C *J*-MOD-, ^19^F-NMR, COSY, NOESY, 2D-HMBC and 2D-HMQC spectra are available in the Supplementary Materials. Chromatographic separations and monitoring of reactions were accomplished on Merck Kieselgel 60 (Merck Ltd., Budapest, Hungary). Optical rotations were measured in MeOH at 20 °C using a PerkinElmer 341 polarimeter (PerkinElmer Inc., Shelton, CT, USA). HRMS flow injection analysis was performed using a Thermo Scientific Q Exactive Plus Hybrid Quadrupole-Orbitrap (Thermo Fisher Scientific, Waltham, MA, USA) mass spectrometer coupled to a Waters ACQUITY I-Class UPLC™ (Waters, Manchester, UK). The melting points were determined using a Kofler apparatus (Nagema, Dresden, Germany) [24].

### 3.2. Starting Materials

The starting material stevioside **I** was obtained from Molar Chemicals Ltd., Halász-telek, Hungary. Preparation of the key intermediate steviol **1** and steviol methyl ester **2** was carried out according to a literature method from **I**, and its spectroscopic data were the same as those reported therein [40,41]. The ^1^H, ^13^C *J*-MOD, ^19^F, COSY, NOESY, HSQC and HMBC NMR spectra of the new compounds are available in the Supplementary Materials.

#### 3.2.1. (2’*S*,4*R*,4a*S*,6a*S*,11a*R*,11b*S*)-Methyl 9-hydroxy-4,11b-dimethyldodecahydro-1*H*-spiro [6a,9-methanocyclohepta[a]naphthalene-8,2’-oxirane]-4-carboxylate (**3**)

An emerald green mixture of **2** (5.00 g, 15.04 mmol) and VO(acac)_2_ (50 mg) in dry toluene (150 mL) was stirred at 0 °C for 30 min. A solution of *t*-BuOOH (70% in H_2_O, 10 mL) in anhydrous toluene (100 mL) was dried on Na_2_SO_4_ and filtered before addition dropwise to the mixture. The colour of the solution changed to maroon during the addition, and after stirring for 1 h at 25 °C, it faded to orange. Saturated NaHCO_3_ solution (30 mL) was added to the mixture, followed by extraction with toluene (3 × 30 mL), and the organic layer was washed with brine before it was dried (Na_2_SO_4_), filtered and concentrated. The crude product was purified using column chromatography on silica gel with *n*-hexane/EtOAc 2:1. Yield: 3.78 g (72%); white crystals; m.p.: 124–126 °C; [*α*]_D_^20^ = –116 (*c* 0.093 MeOH); ^1^H-NMR (500 MHz, CDCl_3_) δ (ppm): 0.81–0.84 (m, 1H), 0.85 (s, 3H), 0.96 (d, 1H, *J* = 8.2 Hz), 0.93–1.03 (m, 1H), 1.03–1.07 (m, 1H), 1.17 (s, 3H), 1.36–1.39 (m, 1H), 1.43–1.50 (m, 3H), 1.58–1.63 (m, 1H), 1.67–1.75 (m, 3H), 1.76–1.78 (m, 1H), 1.79–1.84 (m, 2H), 1.85–1.90 (m, 3H), 2.17–2.22 (m, 2H), 2.32 (s, 1H), 2.78 (d, 1H, *J* = 3.8 Hz), 2.93 (d, 1H, *J* = 3.8 Hz), 3.64 (s, 3H); ^13^C-NMR (125 MHz, CDCl_3_) δ (ppm): 15.5 (CH_3_), 19.1 (CH_2_), 19.6 (CH_2_), 21.8 (CH_2_), 28.7 (CH_3_), 34.8 (CH_2_), 38.0 (CH_2_), 39.3 (C_q_), 40.7 (CH_2_), 41.3 (CH_2_), 41.6 (C_q_), 43.8 (C_q_), 45.8 (CH_2_), 46.5 (CH_2_), 48.7 (C_q_), 51.2 (CH_3_), 53.8 (CH), 56.8 (CH), 65.3 (C_q_), 74.7 (C_q_), 177.9 (C=O). HRMS (ESI+): *m*/*z* calcd. for C_21_H_33_O_4_^+^ [M + H]^+^ 349.2373; found 349.2372 [23].

#### 3.2.2. (4*R*,4a*S*,6a*R*,9*S*,11a*R*,11b*S*)-Methyl 9-(hydroxymethyl)-4,11b-dimethyl-8-oxotetradecahydro-6a,9-methanocyclohepta[a]naphthalene-4-carboxylate (**4**)

Of **3**, 3.00 g (8.61 mmol) was dissolved in anhydrous toluene (200 mL) and 1.1 mL (8.91 mmol) BF_3_·Et_2_O was added dropwise at 25 °C. The mixture was stirred for 1 h and then it was washed with water (3 × 20 mL). The organic phases were dried (Na_2_SO_4_) and evaporated to dryness. The resulting product was purified using column chromatography on silica gel with *n*-hexane/EtOAc 1:1. Yield: 2.63 g (88%); white crystals; m.p.: 168–172 °C; [*α*]_D_^20^ = –91 (*c* 0.097 MeOH); ^1^H-NMR (500 MHz, CDCl_3_) δ (ppm): 0.69 (s, 3H), 0.88–0.97 (m, 1H), 0.99–1.07 (m, 1H), 1.11–1.17 (m, 1H), 1.19 (s, 3H), 1.22–1.28 (m, 2H), 1.28–1.33 (m, 1H), 1.33–1.39 (m, 1H), 1.41–1.47 (m, 1H), 1.49–1.57 (m, 1H), 1.67–1.75 (m, 3H), 1.77–1.87 (m, 5H), 1.88–1.94 (m, 1H), 2.19 (d, 1H, *J* = 13.7 Hz), 2.22 (s, 1H), 2.66 (q, 1H, *J* = 4.2 Hz, 19.2 Hz), 3.52 (d, 1H, *J* = 11.6 Hz), 3.62 (d, 1H, *J* = 4.2 Hz), 3.64 (s, 3H); ^13^C-NMR (125 MHz, CDCl_3_) δ (ppm): 13.2 (CH_3_), 18.9 (CH_2_), 19.8 (CH_2_), 21.7 (CH_2_), 28.8 (CH_3_), 32.1 (CH_2_), 37.9 (CH_2_), 38.1 (C_q_), 39.7 (C_q_), 39.8 (CH_2_), 41.4 (CH_2_), 43.8 (C_q_), 48.9 (CH_2_), 49.0 (CH_2_), 51.3 (CH_3_), 54.1 (C_q_), 55.4 (CH), 57.0 (CH), 65.2 (CH_2_), 177.8 (C=O), 223.1 (C=O). HRMS (ESI+): *m*/*z* calcd. for C_21_H_33_O_4_^+^ [M + H]^+^ 349.2373; found 349.2372 [29].

#### 3.2.3. (4*R*,4a*S*,6a*R*,9*R*,11a*R*,11b*S*,E)-Methyl 8-(hydroxyimino)-9-(hydroxymethyl)-4,11b-dimethyltetradecahydro-6a,9-methanocyclohepta[a]naphthalene-4-carboxylate (**5**)

To a solution of 1.00 g (2.88 mmol) **4** in EtOH (50 mL), 0.67 g (9.68 mmol) hydroxylamine hydrochloride was added, and the mixture was stirred in the presence of NaHCO_3_ (2.74 mmol, 0.23 g) at 60 °C for 4 h. The white crystals were filtered from the reaction mixture, and the solution was concentrated under vacuum and extracted using 50 mL CH_2_Cl_2_ and 50 mL water. The water phase was extracted further with CH_2_Cl_2_ (3 × 50 mL) and the combined organic phases were washed with brine, dried on Na_2_SO_4_ and evaporated to dryness. The product obtained was purified using column chromatography on silica gel with *n*-hexane/EtOAc = 1:2. Yield: 0.75 g (72%); white crystals; m.p.: 198–204 °C; [*α*]_D_^20^ = –32 (*c* 0.077 MeOH); ^1^H-NMR (500 MHz, CDCl_3_) δ (ppm): 0.75 (s, 3H), 0.86–0.93 (m, 1H), 0.99–1.04 (m, 1H), 1.09–1.16 (m, 3H), 1.18 (s, 3H), 1.25–1.29 (m, 1H), 1.34–1.39 (m, 1H), 1.40–1.44 (m, 1H), 1.45–1.50 (m, 1H), 1.55–1.59 (m, 1H), 1.61–1.64 (m, 1H), 1.65–1.69 (m, 1H), 1.70–1.73 (m, 2H), 1.80–1.84 (m, 1H), 1.85–1.88 (m, 1H), 1.92–1.97 (m, 1H), 2.00 (d, 1H, *J* = 19.2 Hz), 2.18 (d, 1H, *J* = 13.3 Hz), 2.98 (q, 1H, *J* = 3.2 Hz, 15.9 Hz), 3.04 (s, 1H), 3.59 (q, 2H, *J* = 10.6 Hz, 19.2 Hz), 3.64 (s, 3H), 7.67 (s, 1H); ^13^C-NMR (125 MHz, CDCl_3_) δ (ppm): 13.2 (CH_3_), 18.9 (CH_2_), 19.9 (CH_2_), 21.7 (CH_2_), 28.8 (CH_3_), 34.1 (CH_2_), 37.0 (CH_2_), 38.0 (CH_2_), 38.1 (C_q_), 39.9 (CH_2_), 40.8 (CH_2_), 40.9 (C_q_), 43.8 (C_q_), 49.4 (C_q_), 51.1 (CH_2_), 51.2 (CH_3_), 55.6 (CH), 57.1 (CH), 66.9 (CH_2_), 170.3 (C=N), 177.9 (C=O). HRMS (ESI+): *m*/*z* calcd. for C_21_H_34_O_4_^+^ [M + H]^+^ 364.2482; found 364.2478 [31].

### 3.3. Hydrogenation of Oxime ***5***

Raney nickel (0.20 g) was suspended in THF (20 mL) and, to this mixture, the anhydrous THF (30 mL) solution of oxime **5** (0.80 g, 2.21 mmol) was added. The mixture was stirred under a H_2_ atmosphere (10 atm) at 25 °C for 24 h; then, it was filtered and evaporated. The resulting diastereomers were separated using column chromatography on silica gel with CHCl_3_/MeOH = 1:1.

#### 3.3.1. (4*R*,4a*S*,6a*R*,8*R*,9*R*,11a*R*,11b*S*)-Methyl 8-amino-9-(hydroxymethyl)-4,11b-dimethyltetradecahydro-6a,9-methanocyclohepta[a]naphthalene-4-carboxylate (**6**)

Yield: 0.22 g (31%); white crystals; m.p.: 166–170 °C; [*α*]_D_^20^ = –45 (*c* 0.077 MeOH); ^1^H-NMR (500 MHz, CDCl_3_) δ (ppm): 0.71 (s, 3H), 0.85–0.91 (m, 1H), 0.92–0.95 (m, 1H), 0.95–1.03 (m, 1H), 1.04–1.07 (m, 2H), 1.16 (s, 3H), 1.22–1.26 (m, 1H), 1.28–1.38 (m, 3H), 1.39–1.44 (m, 1H), 1.53–1.59 (m, 3H), 1.70–1.82 (m, 5H), 2.11–2.21 (m, 5H), 3.23 (q, 1H, *J* = 5.3 Hz, 6.0 Hz), 3.47 (q, 2H, *J* = 5.3 Hz, 10.5 Hz), 3.63 (s, 3H); ^13^C-NMR (125 MHz, CDCl_3_) δ (ppm): 13.2 (CH_3_), 18.9 (CH_2_), 20.6 (CH_2_), 21.8 (CH_2_), 28.7 (CH_2_), 28.9 (CH_3_), 37.9 (CH_2_), 38.0 (C_q_), 39.9 (CH_2_), 41.6 (CH_2_), 42.9 (C_q_), 43.8 (C_q_), 44.5 (CH_2_), 45.3 (C_q_), 51.2 (CH_3_), 51.5 (CH_2_), 56.4 (CH), 57.1 (CH), 59.4 (CH), 72.3 (CH_2_), 178.1 (C=O). HRMS (ESI+): *m*/*z* calcd. for C_21_H_36_NO_3_^+^ [M + H]^+^ 350.2690; found 350.2676.

#### 3.3.2. (4*R*,4a*S*,6a*R*,8*S*,9*R*,11a*R*,11b*S*)-Methyl 8-amino-9-(hydroxymethyl)-4,11b-dimethyltetradecahydro-6a,9-methanocyclohepta[a]naphthalene-4-carboxylate (**7**)

Yield: 0.035 g (15%); white crystals; m.p.: 127–130 °C; [*α*]_D_^20^ = –14 (*c* 0.08 MeOH); ^1^H-NMR (500 MHz, CDCl_3_) δ (ppm): 0.72 (s, 3H), 0.77–0.93 (m, 3H), 0.93–1.15 (m, 5H), 1.17 (s, 3H), 1.24 (t, 1H, *J* = 6.9 Hz), 1.41 (d, 1H, *J* = 14.3 Hz), 1.48–1.65 (m, 5H), 1.69–1.76 (m, 2H), 1.77–1.83 (m, 2H), 2.03 (s, 3H), 2.16 (d, 1H, *J* = 13.2 Hz), 3.53 (q, 2H, *J* = 11.4 Hz, 11.4 Hz), 3.63 (s, 3H), 3.72 (q, 2H, *J* = 7.1 Hz, 7.1 Hz); ^13^C-NMR (125 MHz, CDCl_3_) δ (ppm): 13.3 (CH_3_), 18.9 (CH_2_), 19.7 (CH_2_), 21.8 (CH_2_), 28.7 (C_q_), 28.8 (CH_3_), 34.8 (CH_2_), 37.9 (CH_2_), 40.0 (CH_2_), 41.5 (CH_2_), 43.8 (C_q_), 46.1 (C_q_), 47.6 (C_q_), 48.7 (CH_2_), 51.1 (CH_3_), 51.5 (CH_2_), 55.6 (CH), 57.2 (CH), 57.9 (CH), 67.9 (CH_2_), 178.1 (C=O). HRMS (ESI+): *m*/*z* calcd. for C_21_H_36_NO_3_^+^ [M + H]^+^ 350.2690; found 350.2678.

### 3.4. General Procedure for the Preparation of Aminoalcohols Using Primary Amines from β-keto Alcohol ***4***

Step 1: 0.20 g (0.58 mmol) of **4** was dissolved in anhydrous toluene (30 mL) and then 8 µL (0.06 mmol) of BF_3_.Et_2_O and 3 equivalents of the corresponding amine were added. The mixture was stirred at 130 °C for 24 h in the presence of a molecular sieve (0.25 g). The reaction mixture was filtered and concentrated under vacuum.

Step 2: The crude product obtained in step 1 was dissolved in anhydrous MeOH (20 mL), 65 mg (1.73 mmol) NaBH_4_ was added and the solution was stirred at 0 °C for 1 h. After the completion of the reaction, the solvent was evaporated, and the product was dissolved in CH_2_Cl_2_ (50 mL) and washed with water (3 × 15 mL). The organic phases were washed with brine, dried (Na_2_SO_4_) and evaporated to dryness. The crude product was purified using column chromatography on silica gel with CHCl_3_/MeOH = 19:1.

#### 3.4.1. (4*R*,4a*S*,6a*R*,8*R*,9*R*,11a*R*,11b*S*)-Methyl 8-(benzylamino)-9-(hydroxymethyl)-4,11b-dimethyltetradecahydro-6a,9-methanocyclohepta[a]naphthalene-4-carboxylate (**8**)

The reaction was accomplished using benzylamine, as described in the general procedure. Yield: 109 mg (43%); yellow oil; [*α*]_D_^20^ = –79 (*c* 0.083 MeOH); ^1^H-NMR (500 MHz, CDCl_3_) δ (ppm): 0.71 (s, 3H), 0.85–0.94 (m, 3H), 0.96–1.02 (m, 1H), 1.03–1.07 (m, 2H), 1.17 (s, 3H), 1.19–1.22 (m, 1H), 1.29–1.33 (m, 2H), 1.34–1.37 (m, 1H), 1.39–1.43 (m, 1H), 1.54–1.62 (m, 2H), 1.69–1.73 (m, 3H), 1.78–1.83 (m, 2H), 2.17 (d, 1H, *J* = 13.8 Hz), 2.21–2.26 (m, 1H), 2.85 (s, 2H), 3.13 (q, 1H, *J* = 3.1 Hz, 6.1 Hz), 3.48 (q, 2H, *J* = 9.9 Hz, 12.3 Hz), 3.65 (s, 3H), 3.74 (d, 1H, *J* = 13.1 Hz), 3.89 (d, 1H, *J* = 13.1 Hz), 7.26–7.29 (m, 1H), 7.31–7.34 (m, 4H); ^13^C-NMR (125 MHz, CDCl_3_) δ (ppm): 13.2 (CH_3_), 18.9 (CH_2_), 20.6 (CH_2_), 21.7 (CH_2_), 28.9 (CH_3_), 29.2 (CH_2_), 37.9 (CH_2_), 38.1 (C_q_), 39.9 (CH_2_), 40.1 (CH_2_), 41.6 (CH_2_), 43.1 (C_q_), 43.8 (C_q_), 45.3 (C_q_), 51.2 (CH_3_), 51.3 (CH_2_), 52.5 (CH_2_), 56.4 (CH), 57.1 (CH), 65.5 (CH), 73.0 (CH_2_), 127.3 (CH), 128.3 (2×CH), 128.5 (2×CH), 139.2 (C_q_), 178.0 (C=O). HRMS (ESI+): *m*/*z* calcd. for C_28_H_42_NO_3_^+^ [M + H]^+^ 440.3159; found 440.3149.

#### 3.4.2. (4*R*,4a*S*,6a*R*,8*R*,9*R*,11a*R*,11b*S*)-Methyl 8-((4-fluorobenzyl)amino)-9-(hydroxymethyl)-4,11b-dimethyltetradecahydro-6a,9-methanocyclohepta[a]naphthalene-4-carboxylate (**9**)

The reaction was accomplished using 4-fluorobenzylamine, as described in the general procedure. Yield: 126 mg (48%); yellow oil; [*α*]_D_^20^ = –85 (*c* 0.115 MeOH); ^1^H-NMR (500 MHz, CDCl_3_) δ (ppm): 0.71 (s, 3H), 0.85–0.92 (m, 2H), 0.97–1.02 (m, 1H), 1.03–1.07 (m, 2H), 1.17 (s, 3H), 1.19–1.21 (m, 1H), 1.29–1.33 (m, 2H), 1.35–1.38 (m, 1H), 1.39–1.43 (m, 1H), 1.54–1.57 (m, 1H), 1.57–1.63 (m, 1H), 1.67–1.71 (m, 3H), 1.72–1.74 (m, 1H), 1.78–1.83 (m, 2H), 2.17 (d, 1H, *J* = 13.3 Hz), 2.22 (d, 1H, *J* = 8.9 Hz), 2.76 (s, 2H), 3.07 (t, 1H, *J* = 8.5 Hz), 3.47 (q, 2H, *J* = 8.1 Hz, 10.1 Hz), 3.64 (s, 3H), 3.69 (d, 1H, *J* = 12.9 Hz), 3.84 (d, 1H, *J* = 12.9 Hz), 7.01 (t, 2H, *J* = 7.9 Hz), 7.26–7.30 (m, 2H); ^13^C-NMR (125 MHz, CDCl_3_) δ (ppm): 13.3 (CH_3_), 18.9 (CH_2_), 20.7 (CH_2_), 21.7 (CH_2_), 28.9 (CH_3_), 29.2 (CH_2_), 37.9 (CH_2_), 38.1 (C_q_), 39.9 (CH_2_), 40.3 (CH_2_), 41.7 (CH_2_), 43.1 (C_q_), 43.8 (C_q_), 45.3 (C_q_), 51.2 (CH_3_), 51.3 (CH_2_), 51.8 (CH_2_), 56.4 (CH), 57.0 (CH), 65.3 (CH), 73.1 (CH_2_), 115.2 (CH), 115.4 (CH), 129.7 (CH), 129.8 (CH), 161.1 (C_q_), 163.1 (C_q_), 178.0 (C=O). ^19^F-NMR (470 MHz, CDCl_3_) δ (ppm): −115.5 (C_q-F_). HRMS (ESI+): *m*/*z* calcd. for C_28_H_41_FNO_3_^+^ [M + H]^+^ 458.3065; found 458.3058.

#### 3.4.3. (4*R*,4a*S*,6a*R*,8*R*,9*R*,11a*R*,11b*S*)-Methyl 9-(hydroxymethyl)-8-((4-methoxybenzyl)amino)-4,11b-dimethyltetradecahydro-6a,9-methanocyclohepta[a]naphthalene-4-carboxylate (**10**)

The reaction was accomplished with 4-methoxybenzylamine, as described in the general procedure. Yield: 189 mg (70%); yellow oil; [*α*]_D_^20^ = –80 (*c* 0.088 MeOH); ^1^H-NMR (500 MHz, CDCl_3_) δ (ppm): 0.72 (s, 3H), 0.83–0.89 (m, 1H), 0.91 (d, 1H, *J* = 11.9 Hz), 0.96–1.02 (m, 1H), 1.03–1.07 (m, 2H), 1.16 (s, 3H), 1.19–1.23 (m, 1H), 1.29–1.33 (m, 1H), 1.34–1.38 (m, 1H), 1.39–1.42 (m, 1H), 1.52–1.56 (m, 1H), 1.56–1.62 (m, 1H), 1.63–1.78 (m, 5H), 1.79–1.83 (m, 2H), 2.16 (d, 1H, *J* = 13.6 Hz), 2.23 (d, 1H, *J* = 12.7 Hz), 3.12 (q, 1H, *J* = 4.3 Hz, 6.1 Hz), 3.47 (q, 2H, *J* = 10.4 Hz, 34.9 Hz), 3.64 (s, 3H), 3.64 (s, 1H, overlapping with CH_3_), 3.71 (d, 1H, *J* = 12.9 Hz), 3.77 (s, 1H), 3.79 (s, 3H), 3.89 (d, 1H, *J* = 12.3 Hz), 6.87 (d, 2H, *J* = 8.6 Hz), 7.28 (d, 2H, *J* = 7.6 Hz, overlapping with CDCl_3_); ^13^C-NMR (125 MHz, CDCl_3_) δ (ppm): 13.2 (CH_3_), 18.9 (CH_2_), 20.5 (CH_2_), 21.7 (CH_2_), 28.9 (CH_3_), 29.2 (CH_2_), 37.9 (CH_2_), 38.1 (C_q_), 39.4 (CH_2_), 39.8 (CH_2_), 41.6 (CH_2_), 43.1 (C_q_), 43.8 (C_q_), 45.4 (C_q_), 51.2 (CH_3_), 51.3 (CH_2_), 51.7 (CH_2_), 55.3 (CH_3_), 56.4 (CH), 57.0 (CH), 64.8 (CH), 72.4 (CH_2_), 113.9 (2×CH), 129.8 (2×CH), 130.1 (C_q_), 159.1 (C_q_), 177.9 (C=O). HRMS (ESI+): *m*/*z* calcd. for C_29_H_44_NO_4_^+^ [M + H]^+^ 470.3265; found 470.3255.

#### 3.4.4. (4*R*,4a*S*,6a*R*,8*R*,9*R*,11a*R*,11b*S*)-Methyl 9-(hydroxymethyl)-4,11b-dimethyl-8-(((*S*)-1-phenylpropyl)amino)tetradecahydro-6a,9-methanocyclohepta[a]naphthalene-4-carboxylate (**11**)

The reaction was accomplished using *R*-(+)-α-ethylbenzylamine, as described in the general procedure. Yield: 175 mg (65%); white crystals; m.p.: 88–95 °C; [*α*]_D_^20^ = –26 (*c* 0.083 MeOH); ^1^H-NMR (500 MHz, CDCl_3_) δ (ppm): 0.48 (s, 3H), 0.76 (t, 3H, *J* = 6.9 Hz), 0.82–0.88 (m, 2H), 0.93–0.99 (m, 3H), 1.11 (s, 1H), 1.13 (s, 3H), 1.14–1.17 (m, 1H), 1.24–1.27 (m, 1H), 1.27–1.30 (m, 2H), 1.34–1.37 (m, 1H), 1.38–1.40 (m, 1H), 1.43 (s, 1H), 1.56–1.66 (m, 4H), 1.66–1.80 (m, 4H), 1.84–1.88 (m, 1H), 2.13 (d, 1H, *J* = 13.2 Hz), 2.25 (d, 1H, *J* = 13.2 Hz), 3.20 (q, 1H, *J* = 3.7 Hz, 6.3 Hz), 3.46–3.50 (m, 1H), 3.53 (q, 2H, *J* = 7.9 Hz, 9.5 Hz), 3.61 (s, 3H), 7.21–7.25 (m, 3H), 7.28–7.32 (m, 2H); ^13^C-NMR (125 MHz, CDCl_3_) δ (ppm): 10.7 (CH_3_), 12.9 (CH_3_), 18.8 (CH_2_), 20.7 (CH_2_), 21.6 (CH_2_), 28.8 (CH_3_), 29.2 (C_q_), 29.3 (CH_2_), 30.6 (CH_2_), 37.9 (CH_2_), 39.8 (CH_2_), 40.7 (CH_2_), 41.6 (CH_2_), 43.1 (C_q_), 43.7 (C_q_), 45.3 (C_q_), 51.0 (CH_3_), 51.1 (CH_2_), 56.4 (CH), 56.9 (CH), 64.2 (CH), 65.2 (CH), 73.6 (CH_2_), 127.1 (3×CH), 128.5 (2×CH), 145.1 (C_q_), 178.0 (C=O). HRMS (ESI+): *m*/*z* calcd. for C_30_H_46_NO_3_^+^ [M + H]^+^ 468.3472; found 468.3462.

#### 3.4.5. (4*R*,4a*S*,6a*R*,8*R*,9*R*,11a*R*,11b*S*)-Methyl 9-(hydroxymethyl)-4,11b-dimethyl-8-(((*R*)-1-phenylpropyl)amino)tetradecahydro-6a,9-methanocyclohepta[a]naphthalene-4-carboxylate (**12**)

The reaction was accomplished using *S*-(–)-α-ethylbenzylamine, as described in the general procedure. Yield: 161 mg (60%); white crystals; m.p.: 174–176 °C; [*α*]_D_^20^ = –169 (*c* 0.087 MeOH); ^1^H-NMR (500 MHz, CDCl_3_) δ (ppm): 0.76 (s, 3H), 0.82 (s, 1H), 0.84 (t, 3H, *J* = 7.3 Hz), 0.89–0.93 (m, 1H), 1.00–1.07 (m, 4H), 1.16 (s, 3H), 1.28–1.31 (m, 1H), 1.31–1.34 (m, 1H), 1.40–1.41 (m, 1H), 1.44–1.47 (m, 1H), 1.55–1.56 (m, 3H), 1.61 (s, 1H), 1.64 (t, 2H, *J* = 6.9 Hz), 1.70–1.74 (m, 2H), 1.75–1.85 (m, 4H), 2.17 (d, 1H, *J* = 13.8 Hz), 2.29 (d, 1H, *J* = 10.3 Hz), 2.78 (q, 1H, J = 4.9 Hz, 5.8 Hz), 3.27 (d, 1H, *J* = 9.9 Hz), 3.38 (d, 1H, *J* = 9.9 Hz), 3.51 (t, 1H, *J* = 6.6 Hz), 3.66 (s, 3H), 7.27–7.29 (m, 3H), 7.32–7.35 (m, 2H); ^13^C-NMR (125 MHz, CDCl_3_) δ (ppm): 10.9 (CH_3_), 13.3 (CH_3_), 18.9 (CH_2_), 20.8 (CH_2_), 21.7 (CH_2_), 28.9 (CH_3_), 29.2 (CH_2_), 31.8 (CH_2_), 37.9 (CH_2_), 38.1 (C_q_), 39.7 (CH_2_), 39.9 (CH_2_), 41.6 (CH_2_), 42.9 (C_q_), 43.8 (C_q_), 45.1 (C_q_), 51.2 (CH_3_), 51.3 (CH_2_), 56.4 (CH), 57.1 (CH), 62.6 (CH), 63.3 (CH), 73.0 (CH_2_), 127.2 (CH), 127.5 (CH), 128.5 (CH), 145.1 (C_q_), 178.2 (C=O). HRMS (ESI+): *m*/*z* calcd. for C_30_H_46_NO_3_^+^ [M + H]^+^ 468.3472; found 468.3464.

#### 3.4.6. (4*R*,4a*S*,6a*R*,8*R*,9*R*,11a*R*,11b*S*)-Methyl 9-(hydroxymethyl)-4,11b-dimethyl-8-(((*R*)-1-(naphthalen-1-yl)ethyl)amino)tetradecahydro-6a,9-methanocyclohepta[a]naphthalene-4-carboxylate (**13**)

The reaction was accomplished using *R*-(+)-1-(1-naphthyl)ethylamine, as described in the general procedure. Yield: 119 mg (41%); white crystals; m.p.: 180–185 °C; [*α*]_D_^20^ = –79 (*c* 0.08 MeOH); ^1^H-NMR (500 MHz, CDCl_3_) δ (ppm): 0.56 (s, 3H), 0.80–0.87 (m, 1H), 0.91 (d, 1H, *J* = 11.0 Hz), 0.93–0.98 (m, 1H), 0.99 (s, 1H), 1.02–1.04 (m, 1H), 1.15 (s, 3H), 1.16–1.20 (m, 1H), 1.21–1.24 (m, 1H), 1.25–1.29 (m, 1H), 1.30–1.35 (m, 1H), 1.35–1.39 (m, 1H), 1.47–1.54 (m, 2H), 1.55 (d, 3H, *J* = 6.3 Hz), 1.59–1.69 (m, 5H), 1.70–1.82 (m, 3H), 2.15 (d, 1H, *J* = 13.2 Hz), 2.23 (d, 1H, *J* = 13.2 Hz), 3.38 (q, 1H, *J* = 3.8 Hz, 6.3 Hz), 3.56 (q, 2H, *J* = 9.4 Hz, 20.1 Hz), 3.63 (s, 3H), 4.67 (q, 1H, *J* = 6.1 Hz, 6.7 Hz), 7.44–7.53 (m, 3H), 7.59 (d, 1H, *J* = 7.0 Hz), 7.76 (d, 1H, *J* = 8.2 Hz), 7.86–7.89 (m, 1H), 8.15 (d, 1H, *J* = 7.9 Hz); ^13^C-NMR (125 MHz, CDCl_3_) δ (ppm): 13.1 (CH_3_), 18.8 (CH_2_), 20.7 (CH_2_), 21.4 (CH_3_), 21.7 (CH_2_), 28.8 (CH_3_), 29.2 (CH_2_), 37.9 (CH_2_), 38.0 (C_q_), 39.8 (CH_2_), 39.9 (CH_2_), 41.7 (CH_2_), 43.2 (C_q_), 43.7 (C_q_), 45.3 (C_q_), 51.1 (CH_3_), 51.3 (CH_2_), 51.4 (CH), 56.5 (CH), 57.0 (CH), 64.1 (CH), 73.6 (CH_2_), 122.6 (CH), 123.3 (CH), 125.5 (CH), 125.8 (CH), 126.1 (CH), 127.6 (CH), 129.2 (CH), 130.7 (C_q_), 133.9 (2×C_q_), 178.0 (C=O). HRMS (ESI+): *m*/*z* calcd. for C_33_H_46_NO_3_^+^ [M + H]^+^ 504.3472; found 504.3464.

#### 3.4.7. (4*R*,4a*S*,6a*R*,8*R*,9*R*,11a*R*,11b*S*)-Methyl 9-(hydroxymethyl)-4,11b-dimethyl-8-(((*S*)-1-(naphthalen-1-yl)ethyl)amino)tetradecahydro-6a,9-methanocyclohepta[a]naphthalene-4-carboxylate (**14**)

The reaction was accomplished using *S*-(–)-1-(1-naphthyl)ethylamine, as described in the general procedure. Yield: 110 mg (38%); white crystals; m.p.: 151–157 °C; [*α*]_D_^20^ = –112 (*c* 0.087 MeOH); ^1^H-NMR (500 MHz, CDCl_3_) δ (ppm): 0.76 (s, 3H), 0.81 (d, 1H, *J* = 11.3 Hz), 0.99–1.06 (m, 4H), 1.15 (s, 3H), 1.24–1.27 (m, 1H), 1.31–1.36 (m, 1H), 1.37–1.39 (m, 1H), 1.40–1.45 (m, 2H), 1.50 (d, 3H, *J* = 6.4 Hz), 1.51–1.59 (m, 2H), 1.71–1.90 (m, 8H), 2.18 (d, 1H, *J* = 13.7 Hz), 2.29 (d, 1H, *J* = 13.7 Hz), 2.87 (q, 1H, *J* = 5.5 Hz, 5.5 Hz), 3.36 (q, 2H, *J* = 10.0 Hz, 37.4 Hz), 3.68 (s, 3H), 4.75 (q, 1H, *J* = 6.4 Hz, 6.4 Hz), 7.47–7.55 (m, 3H), 7.65 (d, 1H, *J* = 7.3 Hz), 7.76 (d, 1H, *J* = 7.3 Hz), 7.88 (d, 1H, *J* = 8.2 Hz), 8.20 (d, 1H, *J* = 9.1 Hz); ^13^C-NMR (125 MHz, CDCl_3_) δ (ppm): 13.4 (CH_3_), 18.9 (CH_2_), 20.8 (CH_2_), 21.7 (CH_2_), 24.9 (CH_3_), 28.9 (CH_3_), 29.1 (CH_2_), 37.9 (CH_2_), 38.1 (C_q_), 39.9 (CH_2_), 40.3 (CH_2_), 41.5 (CH_2_), 42.8 (C_q_), 43.8 (C_q_), 45.3 (C_q_), 51.2 (CH_3_), 51.4 (CH_2_), 51.6 (CH), 56.5 (CH), 57.1 (CH), 62.9 (CH), 72.6 (CH_2_), 122.6 (CH), 123.6 (CH), 125.4 (CH), 125.8 (CH), 125.9 (CH), 127.4 (CH), 129.1 (CH), 131.6 (C_q_), 133.9 (C_q_), 140.1 (C_q_), 178.1 (C=O). HRMS (ESI+): *m*/*z* calcd. for C_33_H_46_NO_3_^+^ [M + H]^+^ 504.3472; found 504.3466.

#### 3.4.8. (4*R*,4a*S*,6a*R*,8*R*,9*R*,11a*R*,11b*S*)-Methyl 9-(hydroxymethyl)-4,11b-dimethyl-8-((naphthalen-1-ylmethyl)amino)tetradecahydro-6a,9-methanocyclohepta[a]naphthalene-4-carboxylate (**15**)

The reaction was accomplished using 1-naphthylmethylamine, as described in the general procedure. Yield: 219 mg (78%); yellow oil; [*α*]_D_^20^ = –44 (*c* 0.07 MeOH); ^1^H-NMR (500 MHz, CDCl_3_) δ (ppm): 0.68 (s, 3H), 0.82–0.88 (m, 1H), 0.91 (d, 1H, *J* = 11.8 Hz), 0.96–1.02 (m, 1H), 1.04 (d, 2H, *J* = 11.8 Hz), 1.17 (s, 3H), 1.20–1.23 (m, 1H), 1.27–1.34 (m, 2H), 1.36–1.42 (m, 2H), 1.54–1.61 (m, 2H), 1.66–1.70 (m, 2H), 1.75–1.78 (m, 2H), 1.79–1.84 (m, 2H), 2.17 (d, 2H, *J* = 11.8 Hz), 2.67 (s, 2H), 3.21 (t, 1H, *J* = 8.1 Hz), 3.46 (s, 2H), 3.66 (s, 3H), 4.16 (d, 1H, *J* = 12.4 Hz), 4.31 (d, 1H, *J* = 13.0 Hz), 7.42–7.52 (m, 4H), 7.78 (d, 1H, *J* = 7.9 Hz), 7.85 (d, 1H, *J* = 7.9 Hz), 8.09 (d, 1H, *J* = 8.4 Hz); ^13^C-NMR (125 MHz, CDCl_3_) δ (ppm): 13.1 (CH_3_), 18.9 (CH_2_), 20.7 (CH_2_), 21.8 (CH_2_), 28.9 (CH_3_), 29.2 (CH_2_), 37.9 (CH_2_), 38.1 (C_q_), 39.9 (CH_2_), 40.5 (CH_2_), 41.7 (CH_2_), 43.2 (C_q_), 43.8 (C_q_), 45.4 (C_q_), 50.5 (CH_2_), 51.2 (CH_3_), 51.3 (CH_2_), 56.4 (CH), 57.1 (CH), 66.2 (CH), 73.1 (CH_2_), 123.5 (CH), 125.4 (CH), 125.8 (CH), 126.3 (CH), 126.5 (CH), 128.1 (CH), 128.8 (CH), 131.8 (C_q_), 133.9 (C_q_), 135.5 (C_q_), 178.1 (C=O). HRMS (ESI+): *m*/*z* calcd. for C_32_H_44_NO_3_^+^ [M + H]^+^ 490.3316; found 490.3306.

#### 3.4.9. (4*R*,4a*S*,6a*R*,8*R*,9*R*,11a*R*,11b*S*)-Methyl 9-(hydroxymethyl)-4,11b-dimethyl-8-(((*S*)-1-(naphthalen-2-yl)ethyl)amino)tetradecahydro-6a,9-methanocyclohepta[a]naphthalene-4-carboxylate (**16**)

The reaction was accomplished using (*R*)-1-(2-naphthyl)ethylamine, as described in the general procedure. Yield: 174 mg (60%); white crystals; m.p.: 105–108 °C; [*α*]_D_^20^ = –31 (*c* 0.093 MeOH); ^1^H-NMR (500 MHz, CDCl_3_) δ (ppm): 0.49 (s, 3H), 0.79–0.86 (m, 1H), 0.88 (d, 1H, *J* = 11.0 Hz), 0.92–0.96 (m, 1H), 0.96–1.01 (m, 2H), 1.11 (s, 3H), 1.16–1.22 (m, 2H), 1.26–1.31 (m, 2H), 1.34–1.39 (m, 2H), 1.43–1.47 (m, 6H), 1.63 (d, 1H, *J* = 13.6 Hz), 1.65–1.71 (m, 2H), 1.72–1.78 (m, 1H), 2.12 (d, 1H, *J* = 13.6 Hz), 2.25 (d, 1H, *J* = 12.9 Hz), 3.02 (s, 2H), 3.29 (t, 1H, *J* = 7.1 Hz), 3.52 (s, 3H), 3.56 (q, 2H, *J* = 9.7 Hz, 12.3 Hz), 3.96 (q, 1H, *J* = 6.5 Hz, 6.5 Hz), 7.41–7.48 (m, 3H), 7.71 (s, 1H), 7.80 (d, 3H, *J* = 8.4 Hz); ^13^C-NMR (125 MHz, CDCl_3_) δ (ppm): 13.0 (CH_3_), 18.8 (CH_2_), 20.7 (CH_2_), 21.6 (CH_2_), 22.7 (CH_3_), 28.7 (CH_3_), 29.2 (CH_2_), 37.9 (CH_2_), 38.0 (C_q_), 39.8 (CH_2_), 40.5 (CH_2_), 41.6 (CH_2_), 43.2 (C_q_), 43.7 (C_q_), 45.3 (C_q_), 51.0 (CH_3_), 51.2 (CH_2_), 56.4 (CH), 56.9 (CH), 57.3 (CH), 64.6 (CH), 73.6 (CH_2_), 124.8 (CH), 124.9 (CH), 125.7 (CH), 126.1 (CH), 127.6 (CH), 127.8 (CH), 128.4 (CH), 132.9 (C_q_), 133.5 (C_q_), 143.8 (C_q_), 178.0 (C=O). HRMS (ESI+): *m*/*z* calcd. for C_33_H_46_NO_3_^+^ [M + H]^+^ 504.3472; found 504.3462.

#### 3.4.10. (4*R*,4a*S*,6a*R*,8*R*,9*R*,11a*R*,11b*S*)-Methyl 9-(hydroxymethyl)-4,11b-dimethyl-8-(((*R*)-1-(naphthalen-2-yl)ethyl)amino)tetradecahydro-6a,9-methanocyclohepta[a]naphthalene-4-carboxylate (**17**)

The reaction was implemented using (*S*)-1-(2-naphthyl)ethylamine, according to the general procedure. Yield: 162 mg (56%); white crystals; m.p.: 150–153 °C; [*α*]_D_^20^ = –209 (*c* 0.08 MeOH); ^1^H-NMR (500 MHz, CDCl_3_) δ (ppm): 0.78 (s, 3H), 0.80 (d, 1H, *J* = 12.3 Hz), 0.86–0.93 (m, 1H), 0.97–1.06 (m, 4H), 1.16 (s, 3H), 1.24–1.28 (m, 1H), 1.28–1.33 (m, 1H), 1.34–1.39 (m, 1H), 1.40–1.46 (m, 5H), 1.54–1.58 (m, 1H), 1.59–1.64 (m, 1H), 1.71–1.76 (m, 2H), 1.77–1.86 (m, 3H), 2.18 (d, 1H, *J* = 13.3 Hz), 2.30 (d, 1H, *J* = 12.8 Hz), 2.68 (s, 2H, overlapping with quartet CH), 2.77 (q, 1H, *J* = 4.9 Hz, 5.7 Hz), 3.25 (d, 1H, *J* = 9.9 Hz), 3.38 (d, 1H, *J* = 10.5 Hz), 3.67 (s, 3H), 3.99 (q, 1H, *J* = 6.4 Hz, 6.4 Hz), 7.43–7.51 (m, 3H), 7.69 (s, 1H), 7.81–7.84 (m, 3H); ^13^C-NMR (125 MHz, CDCl_3_) δ (ppm): 13.4 (CH_3_), 18.9 (CH_2_), 20.8 (CH_2_), 21.7 (CH_2_), 25.3 (CH_3_), 28.9 (CH_3_), 29.1 (CH_2_), 37.9 (CH_2_), 38.1 (C_q_), 39.8 (CH_2_), 39.9 (CH_2_), 41.5 (CH_2_), 42.9 (C_q_), 43.8 (C_q_), 45.1 (C_q_), 51.1 (CH_2_), 51.2 (CH_3_), 56.4 (CH), 56.7 (CH), 57.0 (CH), 63.1 (CH), 72.9 (CH_2_), 124.5 (CH), 125.6 (CH), 125.9 (CH), 126.0 (CH), 127.7 (2×CH), 128.6 (CH), 133.0 (C_q_), 133.4 (C_q_), 141.7 (C_q_), 178.1 (C=O). HRMS (ESI+): *m*/*z* calcd. for C_33_H_46_NO_3_^+^ [M + H]^+^ 504.3472; found 504.3465.

### 3.5. (4R,4aS,6aR,9S,11aR,11bS)-Methyl 4,11b-dimethyl-9-(((methylsulfonyl)oxy)methyl)-8-oxotetradecahydro-6a,9-methanocyclohepta[a]naphthalene-4-carboxylate (***18***)

A mixture of 1.00 g (2.88 mmol) of **4**, 1.34 mL (17.31 mmol) of methanesulfonyl chloride and anhydrous pyridine (15 mL) was stirred at 25 °C for 24 h. The solution was diluted with CH_2_Cl_2_ (50 mL) and extracted with diluted HCl (10%, 3 × 20 mL), and the organic phase was washed with brine, dried (Na_2_SO_4_) and evaporated to dryness. The crude product was purified using column chromatography on silica gel with n-hexane/EtOAc = 2:1. Yield: 0.81 g (68%); white crystals; m.p.: 122–124 °C; [*α*]_D_^20^ = –51 (*c* 0.093 MeOH); ^1^H-NMR (500 MHz, CDCl_3_) δ (ppm): 0.69 (s, 3H), 0.88–0.95 (m, 1H), 0.99–1.07 (m, 1H), 1.12–1.16 (m, 1H), 1.19 (s, 3H), 1.24–1.28 (m, 2H), 1.41–1.54 (m, 4H), 1.56–1.59 (m, 1H), 1.68–1.74 (m, 3H), 1.76–1.84 (m, 2H), 1.86 (d, 1H, *J* = 18.9 Hz), 1.90–1.95 (m, 2H), 2.19 (d, 1H, *J* = 13.1 Hz), 2.68 (q, 1H, *J* = 3.6 Hz, 15.3 Hz), 2.99 (s, 3H), 3.64 (s, 3H), 4.05 (d, 1H, *J* = 10.2 Hz), 4.28 (d, 1H, *J* = 10.2 Hz); ^13^C-NMR (125 MHz, CDCl_3_) δ (ppm): 13.2 (CH_3_), 18.9 (CH_2_), 19.5 (CH_2_), 21.6 (CH_2_), 28.8 (CH_3_), 31.9 (CH_2_), 36.9 (CH_3_), 37.9 (CH_2_), 38.0 (C_q_), 39.4 (C_q_), 39.7 (CH_2_), 41.1 (CH_2_), 43.8 (C_q_), 48.1 (CH_2_), 48.4 (CH_2_), 51.3 (CH_3_), 52.4 (C_q_), 54.9 (CH), 56.9 (CH), 70.7 (CH_2_), 177.7 (C=O), 217.8 (C=O). HRMS (ESI+): *m*/*z* calcd. for C_22_H_35_O_6_S^+^ [M + H]^+^ 427.2149; found 427.2133.

### 3.6. (4R,4aS,6aR,8R,9S,11aR,11bS)-Methyl 8-hydroxy-4,11b-dimethyl-9-(((methylsulfonyl)oxy)methyl)tetradecahydro-6a,9-methanocyclohepta[a]naphthalene-4-carboxylate (***21***)

Of NaBH_4_, 147 mg (3.89 mmol) was added to a solution of 0.80 g (1.94 mmol) of **18** in 80 mL of a MeOH/CH_2_Cl_2_ mixture (1:1) and stirred at 0 °C for 1 h. The reaction mixture was concentrated under vacuum, extracted with CH_2_Cl_2_ (50 mL) and washed with water (3 × 15 mL) and brine, dried (Na_2_SO_4_) and evaporated to dryness. The crude product was purified using column chromatography on silica gel with *n*-hexane/EtOAc = 1:1. Yield: 0.71 g (88%); white crystals; m.p.: 129–132 °C; [*α*]_D_^20^ = –71 (*c* 0.087 MeOH);); ^1^H-NMR (500 MHz, CDCl_3_) δ (ppm): 0.74 (s, 3H), 0.85–0.91 (m, 1H), 0.97–1.07 (m, 4H), 1.17 (s, 3H), 1.25–1.30 (m, 1H), 1.32–1.37 (m, 1H), 1.38–1.44 (m, 2H), 1.52–1.56 (m, 1H), 1.58 (s, 2H), 1.66–1.71 (m, 2H), 1.71–1.75 (m, 1H), 1.77–1.82 (m, 3H), 1.84–1.89 (m, 1H), 1.89–1.94 (m, 1H), 2.17 (d, 1H, *J* = 13.6 Hz), 3.00 (s, 3H), 3.63 (s, 3H), 4.02 (q, 2H, *J* = 9.5 Hz, 12.7 Hz), 4.18 (d, 1H, *J* = 9.5 Hz); ^13^C-NMR (125 MHz, CDCl_3_) δ (ppm): 13.1 (CH_3_), 18.9 (CH_2_), 19.7 (CH_2_), 21.7 (CH_2_), 28.8 (CH_3_), 28.9 (CH_2_), 37.2 (CH_3_), 37.9 (CH_2_), 38.1 (C_q_), 39.9 (CH_2_), 41.4 (CH_2_), 42.1 (CH_2_), 42.3 (C_q_), 43.8 (C_q_), 45.7 (C_q_), 49.6 (CH_2_), 51.2 (CH_3_), 56.0 (CH), 57.1 (CH), 75.7 (CH_2_), 75.9 (CH), 177.9 (C=O). HRMS (ESI+): *m*/*z* calcd. for C_22_H_37_O_6_S^+^ [M + H]^+^ 429.2305; found 429.2288.

### 3.7. General Procedure for Azide Synthesis of ***18*** and ***21*** for Preparation of ***19*** and ***20***

Of NaN_3_, 4.36 mmol was added to a solution of 0.90 g (2.18 mmol) of **18** and **21**, respectively, in anhydrous DMF (50 mL). Stirring was continued at 80 °C for 24 h; then, the mixture was concentrated under vacuum, dissolved in CH_2_Cl_2_ (50 mL) and washed with water (3 × 20 mL). The organic phase was washed with brine, dried (Na_2_SO_4_) and evaporated to dryness. Compounds **19**–**20** were purified using column chromatography on a silica gel column with *n*-hexane/EtOAc = 2:1.

#### 3.7.1. (4*R*,4a*S*,6a*R*,9*S*,11a*R*,11b*S*)-Methyl 9-(azidomethyl)-4,11b-dimethyl-8-oxotetradecahydro-6a,9-methanocyclohepta[a]naphthalene-4-carboxylate (**19**)

The reaction was accomplished using compound **18**, as described in the general procedure. Yield: 0.53 g (65%); white crystals; m.p.: 97–99 °C; [*α*]_D_^20^ = –35 (*c* 0.083 MeOH); ^1^H-NMR (500 MHz, CDCl_3_) δ (ppm): 0.69 (s, 3H), 0.88–0.94 (m, 1H), 0.99–1.06 (m, 1H), 1.11–1.15 (m, 1H), 1.19 (s, 3H), 1.22–1.26 (m, 2H), 1.38–1.45 (m, 2H), 1.45–1.49 (m, 1H), 1.49–1.55 (m, 1H), 1.59 (s, 1H), 1.68–1.77 (m, 4H), 1.79–1.87 (m, 3H), 1.89–1.94 (m, 1H), 2.19 (d, 1H, *J* = 13.2 Hz), 2.66 (q, 1H, *J* = 3.3 Hz, 15.5 Hz), 3.19 (d, 1H, *J* = 12.1 Hz), 4.51 (d, 1H, *J* = 12.1 Hz), 3.63 (s, 3H); ^13^C-NMR (125 MHz, CDCl_3_) δ (ppm): 13.2 (CH_3_), 18.9 (CH_2_), 19.7 (CH_2_), 21.7 (CH_2_), 28.8 (CH_3_), 33.3 (CH_2_), 37.8 (CH_2_), 37.9 (C_q_), 39.5 (C_q_), 39.8 (CH_2_), 41.3 (CH_2_), 43.8 (C_q_), 48.6 (CH_2_), 49.2 (CH_2_), 51.3 (CH_3_), 53.3 (C_q_), 54.3 (CH_2_), 54.9 (CH), 56.9 (CH), 177.7 (C=O), 219.1 (C=O). HRMS (ESI+): *m*/*z* calcd. for C_21_H_31_N_3_O_3_Na^+^ [M + Na]^+^ 396.2258; found 396.2244.

#### 3.7.2. (4*R*,4a*S*,6a*R*,8*R*,9*S*,11a*R*,11b*S*)-Methyl 9-(azidomethyl)-8-hydroxy-4,11b-dimethyltetradecahydro-6a,9-methanocyclohepta[a]naphthalene-4-carboxylate (**20**)

The reaction was accomplished using compound **21**, as described in the general procedure. Yield: 0.65 g (80%); white crystals; m.p.: 72–74 °C; [*α*]_D_^20^ = –73 (*c* 0.073 MeOH); ^1^H-NMR (500 MHz, CDCl_3_) δ (ppm): 0.73 (s, 3H), 0.86–0.89 (m, 1H), 0.99–1.04 (m, 4H), 1.16 (s, 3H), 1.21–1.25 (m, 1H), 1.27–1.29 (m, 1H), 1.30–1.37 (m, 2H), 1.38–1.41 (m, 1H), 1.51–1.52 (m, 1H), 1.57–1.59 (m, 1H), 1.63–1.66 (m, 2H), 1.73–1.76 (m, 2H), 1.81–1.88 (m, 3H), 1.92–1.96 (m, 1H), 2.16 (d, 1H, *J* = 13.2 Hz), 3.23 (q, 1H, *J* = 11.8 Hz, 28.5 Hz), 3.62 (s, 3H), 4.05 (q, 1H, *J* = 4.4 Hz, 6.5 Hz); ^13^C-NMR (125 MHz, CDCl_3_) δ (ppm): 13.1 (CH_3_), 18.9 (CH_2_), 19.9 (CH_2_), 21.7 (CH_2_), 28.9 (CH_3_), 30.2 (CH_2_), 37.9 (CH_2_), 38.0 (C_q_), 39.9 (CH_2_), 41.5 (CH_2_), 42.2 (CH_2_), 42.3 (C_q_), 43.8 (C_q_), 46.1 (C_q_), 50.9 (CH_2_), 51.1 (CH_3_), 56.1 (CH), 57.1 (CH), 60.2 (CH_2_), 77.4 (CH), 178.0 (C=O). HRMS (ESI+): *m*/*z* calcd. for C_21_H_34_N_3_O_3_^+^ [M + H]^+^ 376.2595; found 376.2588.

### 3.8. General Procedure for Preparation of Aminoalcohol Regioisomers with Primary Amines from Hydroxy-Mesylate ***21***

To 0.20 g (0.48 mmol) of **21** in a 1:1 mixture of trimethylamine and MeCN (5 mL), four equivalents of the corresponding amine were added. The solution was stirred at 80 °C for 4 days and, upon completion, evaporated to dryness. Extraction followed using CH_2_Cl_2_ (40 mL), then washing with water (3 × 15 mL), washing with brine, drying (Na_2_SO_4_) and concentration under vacuum. The crude product was purified using column chromatography on silica gel with CHCl_3_/MeOH = 19:1.

#### 3.8.1. (4*R*,4a*S*,6a*R*,8*R*,9*S*,11a*R*,11b*S*)-Methyl 9-((benzylamino)methyl)-8-hydroxy-4,11b-dimethyltetradecahydro-6a,9-methanocyclohepta[a]naphthalene-4-carboxylate (**22**)

The reaction was accomplished using benzylamine, as described in the general procedure. Yield: 45 mg (22%); white crystals; m.p.: 98–104 °C; [*α*]_D_^20^ = –51 (*c* 0.07 MeOH); ^1^H-NMR (500 MHz, CDCl_3_) δ (ppm): 0.73 (s, 3H), 0.81–0.83 (m, 1H), 0.94–0.96 (m, 1H), 0.98–1.04 (m, 3H), 1.10–1.14 (m, 1H), 1.14–1.15 (m, 1H), 1.16 (s, 3H), 1.25–1.28 (m, 1H), 1.37–1.41 (m, 1H), 1.49–1.53 (m, 1H), 1.53–1.64 (m, 3H), 1.66–1.71 (m, 2H), 1.75–1.83 (m, 3H), 1.96–2.04 (m, 3H), 2.16 (d, 1H, *J* = 13.4 Hz), 2.29 (d, 1H, *J* = 11.6 Hz), 2.68 (d, 1H, *J* = 11.6 Hz), 3.62 (s, 3H), 3.76 (q, 2H, *J* = 13.0 Hz, 17.4 Hz), 4.03 (q, 1H, *J* = 5.1 Hz, 5.8 Hz), 7.26–7.28 (m, 1H), 7.29–7.34 (m, 4H); ^13^C-NMR (125 MHz, CDCl_3_) δ (ppm): 13.1 (CH_3_), 18.9 (CH_2_), 20.0 (CH_2_), 21.8 (CH_2_), 28.9 (CH_3_), 30.3 (CH_2_), 38.1 (C_q_), 39.9 (CH_2_), 40.9 (CH_2_), 41.67 (CH_2_), 41.68 (CH_2_), 42.5 (C_q_), 43.8 (C_q_), 45.5 (C_q_), 51.1 (CH_3_), 51.9 (CH_2_), 54.9 (CH_2_), 56.6 (CH), 57.2 (CH), 58.8 (CH_2_), 80.1 (CH), 127.1 (CH), 128.1 (2xCH), 128.5 (2xCH), 139.9 (C_q_), 178.1 (C=O). HRMS (ESI+): *m*/*z* calcd. for C_28_H_42_NO_3_^+^ [M + H]^+^ 440.3159; found 440.3153.

#### 3.8.2. (4*R*,4a*S*,6a*R*,8*R*,9*S*,11a*R*,11b*S*)-Methyl 9-(((4-fluorobenzyl)amino)methyl)-8-hydroxy-4,11b-dimethyltetradecahydro-6a,9-methanocyclohepta[a]naphthalene-4-carboxylate (**23**)

The reaction was accomplished using 4-fluorobenzylamine, as described in the general procedure. Yield: 88 mg (41%); yellow oil; [*α*]_D_^20^ = –36 (*c* 0.098 MeOH); ^1^H-NMR (500 MHz, CDCl_3_) δ (ppm): 0.72 (s, 3H), 0.82–0.85 (m, 2H), 0.97–1.04 (m, 4H), 1.12–1.14 (m, 1H), 1.15 (s, 3H), 1.17–1.19 (m, 1H), 1.28–1.30 (m, 1H), 1.36–1.39 (m, 1H), 1.49–1.53 (m, 1H), 1.58–1.62 (m, 2H), 1.68–1.71 (m, 2H), 1.78–1.82 (m, 2H), 1.83–1.86 (m, 1H), 1.99–2.03 (m, 1H), 2.16 (d, 1H, *J* = 13.2 Hz), 2.23 (s, 2H), 2.29 (d, 1H, *J* = 11.4 Hz), 2.66 (d, 1H, *J* = 11.4 Hz), 3.62 (s, 3H), 3.77 (q, 2H, *J* = 13.2 Hz, 23.9 Hz), 4.03 (q, 1H, *J* = 4.2 Hz, 6.6 Hz), 7.01 (t, 2H, *J* = 8.9 Hz), 7.26–7.30 (m, 2H); ^13^C-NMR (125 MHz, CDCl_3_) δ (ppm): 13.1 (CH_3_), 18.9 (CH_2_), 19.9 (CH_2_), 21.7 (CH_2_), 28.9 (CH_3_), 30.3 (CH_2_), 38.05 (CH_2_), 38.06 (C_q_), 39.9 (CH_2_), 41.2 (CH_2_), 41.6 (CH_2_), 42.4 (C_q_), 43.8 (C_q_), 45.3 (C_q_), 51.1 (CH_3_), 51.9 (CH_2_), 53.8 (CH_2_), 56.7 (CH), 57.1 (CH), 58.3 (CH_2_), 79.7 (CH), 115.3 (CH), 115.4 (CH), 129.90 (CH), 129.96 (CH), 161.2 (C_q_), 163.1 (C_q_), 178.0 (C=O). ^19^F-NMR (470 MHz, CDCl_3_) δ (ppm): −115.3 (C_q-F_). HRMS (ESI+): *m*/*z* calcd. for C_28_H_41_FNO_3_^+^ [M + H]^+^ 458.3065; found 458.3058.

#### 3.8.3. (4*R*,4a*S*,6a*R*,8*R*,9*S*,11a*R*,11b*S*)-Methyl 8-hydroxy-4,11b-dimethyl-9-((((*R*)-1-phenylpropyl)amino)methyl)tetradecahydro-6a,9-methanocyclohepta[a]naphthalene-4-carboxylate (**24**)

The reaction was accomplished using *R*-(+)-α-ethylbenzylamine, as described in the general procedure. Yield: 87 mg (40%); yellow oil; [*α*]_D_^20^ = –41 (*c* 0.077 MeOH); ^1^H-NMR (500 MHz, CDCl_3_) δ (ppm): 0.72 (s, 3H), 0.79 (t, 3H, *J* = 6.8 Hz), 0.85–0.89 (m, 2H), 0.97–1.02 (m, 4H), 1.14 (s, 3H), 1.24–1.25 (m, 1H), 1.39–1.42 (m, 1H), 1.43–1.47 (m, 1H), 1.55–1.58 (m, 1H), 1.60–1.65 (m, 3H), 1.78–1.83 (m, 4H), 2.05–2.07 (m, 1H), 2.08–2.18 (m, 4H), 2.53 (d, 1H, *J* = 11.6 Hz), 3.42 (s, 1H), 3.62 (s, 3H), 3.83 (d, 1H, *J* = 6.8 Hz), 4.03 (q, 1H, *J* = 9.7 Hz, 12.5 Hz), 7.27 (s, 2H), 7.29–7.35 (m, 3H); ^13^C-NMR (125 MHz, CDCl_3_) δ (ppm): 10.7 (CH_3_), 13.1 (CH_3_), 18.9 (CH_2_), 20.0 (CH_2_), 21.7 (CH_2_), 28.9 (CH_3_), 30.4 (CH_2_), 38.1 (CH_2_), 39.9 (CH_2_), 40.7 (CH_2_), 41.6 (CH_2_), 42.5 (C_q_), 43.8 (C_q_), 45.1 (C_q_), 49.6 (C_q_), 51.1 (CH_3_), 51.8 (CH_2_), 56.6 (CH), 56.9 (CH_2_), 57.1 (CH), 65.9 (CH), 75.7 (CH_2_), 79.8 (CH), 127.3 (CH), 127.5 (2×CH), 128.5 (2×CH), 148.3 (C_q_), 178.1 (C=O). HRMS (ESI+): *m*/*z* calcd. for C_30_H_46_NO_3_^+^ [M + H]^+^ 468.3472; found 468.3466.

#### 3.8.4. (4*R*,4a*S*,6a*R*,8*R*,9*S*,11a*R*,11b*S*)-Methyl 8-hydroxy-4,11b-dimethyl-9-((((*S*)-1-phenylpropyl)amino)methyl)tetradecahydro-6a,9-methanocyclohepta[a]naphthalene-4-carboxylate (**25**)

The reaction was accomplished using *S*-(–)-α-ethylbenzylamine, as described in the general procedure. Yield: 79 mg (36%); yellow oil; [*α*]_D_^20^ = –53 (*c* 0.073 MeOH); ^1^H-NMR (500 MHz, CDCl_3_) δ (ppm): 0.72 (s, 3H), 0.78 (t, 3H, *J* = 7.5 Hz), 0.81–0.83 (m, 1H), 0.86 (s, 1H), 0.90–0.92 (m, 1H), 0.92–0.96 (m, 2H), 1.00 (m, 1H), 1.12 (d, 1H, *J* = 2.5 Hz), 1.14 (s, 3H), 1.26 (s, 3H), 1.36–1.38 (m, 1H), 1.47–1.50 (m, 1H), 1.52–1.60 (m, 3H), 1.68–1.71 (m, 2H), 1.75 (s, 1H), 1.77–1.79 (m, 1H), 1.80–1.82 (m, 1H), 1.93–1.97 (m, 1H), 2.15 (d, 1H, *J* = 13.6 Hz), 2.35 (d, 1H, *J* = 12.0 Hz), 2.45 (d, 1H, *J* = 12.0 Hz), 2.94 (s, 2H), 3.01 (q, 2H, *J* = 7.5 Hz, 7.0 Hz), 3.46–3.52 (m, 1H), 3.62 (s, 3H), 4.14 (q, 1H, *J* = 4.9 Hz, 6.1 Hz), 7.23 (s, 1H), 7.28–7.30 (m, 1H), 7.35–7.37 (m, 1H), 7.46 (t, 1H, *J* = 7.3 Hz), 7.95–7.98 (m, 1H); ^13^C-NMR (125 MHz, CDCl_3_) δ (ppm): 10.8 (CH_3_), 13.1 (CH_3_), 18.9 (CH_2_), 19.9 (CH_2_), 21.7 (CH_2_), 28.8 (CH_3_), 29.7 (C_q_), 30.2 (CH_2_), 31.8 (CH_2_), 38.0 (CH_2_), 39.9 (CH_2_), 41.3 (CH_2_), 41.6 (CH_2_), 42.4 (C_q_), 43.8 (C_q_), 44.8 (C_q_), 51.1 (CH_3_), 51.9 (CH_2_), 56.5 (CH), 56.8 (CH_2_), 57.1 (CH), 65.9 (CH), 78.9 (CH), 127.2 (CH), 127.7 (CH), 127.9 (CH), 128.5 (CH), 128.7 (CH), 132.9 (C_q_), 178.0 (C=O). HRMS (ESI+): *m*/*z* calcd. for C_30_H_46_NO_3_^+^ [M + H]^+^ 468.3472; found 468.3463.

#### 3.8.5. (4*R*,4a*S*,6a*R*,8*R*,9*S*,11a*R*,11b*S*)-Methyl 8-hydroxy-4,11b-dimethyl-9-(((naphthalen-1-ylmethyl)amino)methyl)tetradecahydro-6a,9-methanocyclohepta[a]naphthalene-4-carboxylate (**26**)

The reaction was accomplished using 1-naphthylmethylamine, as described in the general procedure. Yield: 101 mg (44%); yellow oil; [*α*]_D_^20^ = –39 (*c* 0.05 MeOH); ^1^H-NMR (500 MHz, CDCl_3_) δ (ppm): 0.70 (s, 3H), 0.81–0.84 (m, 1H), 0.94–0.96 (m, 1H), 0.97–0.98 (m, 1H), 0.99–1.02 (m, 2H), 1.08–1.11 (m, 1H), 1.15 (s, 3H), 1.16 (s, 1H), 1.26 (s, 1H), 1.34–1.37 (m, 1H), 1.49 (s, 1H), 1.53–1.59 (m, 3H), 1.65 (s, 1H), 1.67–1.69 (m, 1H), 1.75–1.79 (m, 2H), 1.80 (s, 1H), 1.93 (s, 1H), 2.03 (s, 2H), 2.13 (s, 1H), 2.46 (d, 1H, *J* = 12.0 Hz), 2.79 (d, 1H, *J* = 11.6 Hz), 3.61 (s, 3H), 4.05 (q, 1H, *J* = 4.9 Hz, 5.8 Hz), 4.24 (q, 2H, *J* = 12.9 Hz, 15.1 Hz), 7.43 (s, 1H), 7.47–7.53 (m, 3H), 7.77–7.80 (m, 1H), 7.86 (d, 1H, *J* = 8.0 Hz), 8.08 (d, 1H, *J* = 8.5 Hz); ^13^C-NMR (125 MHz, CDCl_3_) δ (ppm): 13.0 (CH_3_), 18.9 (CH_2_), 19.9 (CH_2_), 21.7 (CH_2_), 28.9 (CH_3_), 30.2 (CH_2_), 38.0 (CH_2_), 38.1 (C_q_), 39.9 (CH_2_), 41.2 (CH_2_), 41.6 (CH_2_), 42.4 (C_q_), 43.8 (C_q_), 45.4 (C_q_), 51.1 (CH_3_), 51.9 (CH_2_), 52.4 (CH_2_), 56.5 (CH), 57.1 (CH), 59.2 (CH_2_), 79.8 (CH), 123.4 (CH), 125.3 (CH), 125.8 (CH), 126.4 (CH), 128.2 (CH), 128.7 (CH), 128.8 (CH), 133.9 (3×C_q_), 178.1 (C=O). HRMS (ESI+): *m*/*z* calcd. for C_32_H_44_NO_3_^+^ [M + H]^+^ 490.3316; found 490.3306.

#### 3.8.6. (4*R*,4a*S*,6a*R*,8*R*,9*S*,11a*R*,11b*S*)-Methyl 8-hydroxy-4,11b-dimethyl-9-((((*S*)-1-(naphthalen-1-yl)ethyl)amino)methyl)tetradecahydro-6a,9-methanocyclohepta[a]naphthalene-4-carboxylate (**27**)

The reaction was accomplished using *R*-(+)-1-(1-naphthyl)ethylamine, as described in the general procedure. Yield: 68 mg (29%); yellow oil; [*α*]_D_^20^ = –81 (*c* 0.03 MeOH); ^1^H-NMR (500 MHz, CDCl_3_) δ (ppm): 0.72 (s, 3H), 0.85 (s, 1H), 0.87 (s, 1H), 0.87–0.89 (m, 3H), 0.85–0.97 (m, 1H), 0.97 (s, 1H), 1.00 (s, 1H), 1.04 (s, 1H), 1.12 (s, 1H), 1.14 (s, 3H), 1.21 (s, 1H), 1.36–1.38 (m, 1H), 1.42–1.44 (m, 1H), 1.57–1.58 (m, 1H), 1.59–1.60 (m, 1H), 1.65–1.67 (m, 2H), 1.71 (s, 1H), 1.73 (s, 1H), 1.79–1.80 (m, 1H), 1.81–1.82 (m, 1H), 2.03–2.05 (m, 1H), 2.14 (s, 1H), 2.23 (s, 2H), 2.27 (d, 2H, *J* = 11.8 Hz), 2.63 (d, 1H, *J* = 11.6 Hz), 3.62 (s, 3H), 3.99–4.03 (m, 1H), 4.68 (d, 1H, *J* = 6.5 Hz), 7.48–7.53 (m, 3H), 7.66 (d, 1H, *J* = 7.4 Hz), 7.78 (d, 1H, *J* = 8.5 Hz), 7.88 (d, 1H, *J* = 7.9 Hz), 8.11 (d, 1H, *J* = 7.9 Hz); ^13^C-NMR (125 MHz, CDCl_3_) δ (ppm): 13.1 (CH_3_), 14.1 (CH_3_), 18.9 (CH_2_), 19.9 (CH_2_), 21.7 (CH_2_), 28.9 (CH_3_), 30.3 (CH_2_), 38.03 (CH_2_), 38.04 (C_q_), 39.9 (CH_2_), 41.1 (CH_2_), 41.5 (CH_2_), 42.4 (C_q_), 43.7 (C_q_), 44.9 (C_q_), 51.1 (CH_3_), 51.8 (CH_2_), 54.2 (CH), 56.5 (CH), 56.9 (CH_2_), 57.1 (CH), 79.4 (CH), 122.5 (CH), 122.9 (CH), 125.6 (CH), 125.7 (CH), 126.2 (CH), 127.8 (CH), 129.1 (CH), 131.3 (C_q_), 134.0 (2×C_q_), 178.1 (C=O). HRMS (ESI+): *m*/*z* calcd. for C_33_H_46_NO_3_^+^ [M + H]^+^ 504.3472; found 504.3464.

#### 3.8.7. (4*R*,4a*S*,6a*R*,8*R*,9*S*,11a*R*,11b*S*)-Methyl 8-hydroxy-4,11b-dimethyl-9-((((*R*)-1-(naphthalen-1-yl)ethyl)amino)methyl)tetradecahydro-6a,9-methanocyclohepta[a]naphthalene-4-carboxylate (**28**)

The reaction was accomplished using *S*-(–)-1-(1-naphthyl)ethylamine, as described in the general procedure. Yield: 50 mg (21%); yellow oil; [*α*]_D_^20^ = +12 (*c* 0.04 MeOH); ^1^H-NMR (500 MHz, CDCl_3_) δ (ppm): 0.70 (s, 3H), 0.84–0.86 (m, 2H), 0.88–0.89 (m, 3H), 0.95–0.97 (m, 2H), 0.99 (s, 1H), 1.11–1.12 (s, 1H), 1.14 (s, 3H), 1.24 (s, 1H), 1.33 (s, 2H), 1.39 (s, 1H), 1.46 (s, 1H), 1.59 (s, 1H), 1.60 (s, 1H), 1.67–1.69 (m, 3H), 1.76–1.79 (m, 2H), 1.83 (s, 1H), 2.15 (s, 1H), 2.55 (q, 2H, *J* = 5.7 Hz, 13.6 Hz), 2.67 (s, 2H), 3.61 (s, 3H), 4.13–4.19 (m, 1H), 4.76 (d, 1H, *J* = 5.9 Hz), 7.52–7.53 (m, 2H), 7.80 (d, 1H, *J* = 7.7 Hz), 7.87–7.90 (m, 2H), 7.94 (d, 1H, *J* = 7.7 Hz), 8.05 (d, 1H, *J* = 8.3 Hz); ^13^C-NMR (125 MHz, CDCl_3_) δ (ppm): 13.1 (CH_3_), 14.2 (CH_3_), 18.9 (CH_2_), 19.9 (CH_2_), 21.7 (CH_2_), 28.8 (CH_3_), 30.2 (CH_2_), 38.01 (CH_2_), 38.02 (C_q_), 39.9 (CH_2_), 41.5 (CH_2_), 41.6 (CH_2_), 42.4 (C_q_), 43.7 (C_q_), 44.8 (C_q_), 51.1 (CH_3_), 51.8 (CH_2_), 54.1 (CH), 56.4 (CH), 56.8 (CH_2_), 57.1 (CH), 78.7 (CH), 122.3 (CH), 126.0 (CH), 126.5 (CH), 128.1 (CH), 128.4 (CH), 128.6 (CH), 129.2 (CH), 131.0 (C_q_), 133.9 (2×C_q_), 178.0 (C=O). HRMS (ESI+): *m*/*z* calcd. for C_33_H_46_NO_3_^+^ [M + H]^+^ 504.3472; found 504.3462.

#### 3.8.8. (4*R*,4a*S*,6a*R*,8*R*,9*S*,11a*R*,11b*S*)-Methyl 8-hydroxy-4,11b-dimethyl-9-((((*R*)-1-(naphthalen-2-yl)ethyl)amino)methyl)tetradecahydro-6a,9-methanocyclohepta[a]naphthalene-4-carboxylate (**29**)

The reaction was accomplished using (*R*)-1-(2-naphthyl)ethylamine, as described in the general procedure. Yield: 46 mg (19%); yellow oil; [*α*]_D_^20^ = +2 (*c* 0.08 MeOH); ^1^H-NMR (500 MHz, CDCl_3_) δ (ppm): 0.71 (s, 3H), 0.79–0.85 (m, 2H), 0.96–0.99 (m, 3H), 1.06 (d, 1H, *J* = 10.7 Hz), 1.13 (s, 3H), 1.21–1.23 (m, 1H), 1.35–1.37 (m, 1H), 1.39–1.42 (m, 1H), 1.49–1.54 (m, 1H), 1.57–1.62 (m, 5H), 1.63–1.65 (m, 1H), 1.67–1.69 (m, 1H), 1.71–1.73 (m, 1H), 1.79–1.84 (m, 2H), 2.14 (d, 1H, *J* = 12.4 Hz), 2.25 (d, 1H, *J* = 12.4 Hz), 2.66 (d, 1H, *J* = 10.7 Hz), 3.44 (s, 2H), 3.60 (s, 3H), 3.96 (d, 1H, *J* = 7.4 Hz), 4.07 (s, 1H), 7.48–7.50 (m, 2H), 7.59 (d, 1H, *J* = 8.6 Hz), 7.78 (s, 1H), 7.83–7.86 (m, 2H), 7.86–7.88 (m, 1H); ^13^C-NMR (125 MHz, CDCl_3_) δ (ppm): 13.1 (CH_3_), 18.9 (CH_2_), 19.9 (CH_2_), 21.7 (CH_2_), 23.2 (CH_3_), 28.8 (CH_3_), 30.5 (CH_2_), 38.01 (CH_2_), 38.04 (C_q_), 39.9 (CH_2_), 41.2 (CH_2_), 41.5 (CH_2_), 42.3 (C_q_), 43.7 (C_q_), 44.8 (C_q_), 51.1 (CH_3_), 51.7 (CH_2_), 56.1 (CH_2_), 56.4 (CH), 57.1 (CH), 59.5 (CH), 78.7 (CH), 124.6 (CH), 126.1 (CH), 126.3 (CH), 126.5 (CH), 127.7 (CH), 127.9 (CH), 128.9 (CH), 133.1 (C_q_), 133.3 (C_q_), 140.5 (C_q_), 178.0 (C=O). HRMS (ESI+): *m*/*z* calcd. for C_33_H_46_NO_3_^+^ [M + H]^+^ 504.3472; found 504.3461.

#### 3.8.9. (4*R*,4a*S*,6a*R*,8*R*,9*S*,11a*R*,11b*S*)-Methyl 8-hydroxy-4,11b-dimethyl-9-((((*S*)-1-(naphthalen-2-yl)ethyl)amino)methyl)tetradecahydro-6a,9-methanocyclohepta[a]naphthalene-4-carboxylate (**30**)

The reaction was implemented using (*S*)-1-(2-naphthyl)ethylamine, according to the general procedure. Yield: 36 mg (15%); white crystals; m.p.: 102–106 °C; [*α*]_D_^20^ = –50 (*c* 0.067 MeOH); ^1^H-NMR (500 MHz, CDCl_3_) δ (ppm): 0.73 (s, 3H), 0.80–0.85 (m, 1H), 0.87–0.89 (m, 1H), 0.96–1.03 (m, 4H), 1.10–1.14 (m, 1H), 1.15 (s, 3H), 1.28–1.32 (m, 1H), 1.36–1.40 (m, 1H), 1.43 (m, 3H, *J* = 6.5 Hz), 1.49–1.53 (m, 1H), 1.58–1.61 (m, 2H), 1.63–1.66 (m, 1H), 1.68–1.74 (m, 3H), 1.76 (s, 1H), 1.79 (s, 1H), 1.82–1.86 (m, 2H), 1.96–1.99 (m, 1H), 2.15 (d, 1H, *J* = 13.1 Hz), 2.23 (d, 1H, *J* = 11.9 Hz), 2.49 (d, 1H, *J* = 11.9 Hz), 3.62 (s, 3H), 3.84 (q, 1H, *J* = 6.3 Hz, 6.8 Hz), 4.10 (q, 1H, *J* = 4.8 Hz, 6.1 Hz), 7.38 (d, 1H, *J* = 8.3 Hz), 7.44–7.48 (m, 2H), 7.64 (s, 1H), 7.80–7.83 (m, 3H); ^13^C-NMR (125 MHz, CDCl_3_) δ (ppm): 13.1 (CH_3_), 18.9 (CH_2_), 20.0 (CH_2_), 21.8 (CH_2_), 23.8 (CH_3_), 28.9 (CH_3_), 30.2 (CH_2_), 38.05 (CH_2_), 38.06 (C_q_), 39.9 (CH_2_), 40.9 (CH_2_), 41.7 (CH_2_), 42.5 (C_q_), 43.8 (C_q_), 45.4 (C_q_), 51.1 (CH_3_), 51.8 (CH_2_), 56.6 (CH), 57.1 (CH), 57.4 (CH_2_), 59.2 (CH), 80.2 (CH), 124.4 (CH), 125.1 (CH), 125.6 (CH), 126.1 (CH), 127.6 (CH), 127.7 (CH), 128.4 (CH), 132.8 (C_q_), 133.4 (C_q_), 142.8 (C_q_), 178.1 (C=O). HRMS (ESI+): *m*/*z* calcd. for C_33_H_46_NO_3_^+^ [M + H]^+^ 504.3472; found 504.3464.

### 3.9. (4. R,4aS,6aR,8R,9S,11aR,11bS)-Methyl 9-(aminomethyl)-8-hydroxy-4,11b-dimethyltetradecahydro-6a,9-methanocyclohepta[a]naphthalene-4-carboxylate (***31***)

To the solution of **21** (0.60 g, 1.59 mmol) in MeOH (70 mL), a suspension of palladium on carbon (Pd/C, 0.07 g) in MeOH (10 mL) was added. After stirring the mixture at 25 °C for 24 h under a H_2_ atmosphere (1 atm), it was filtered through a Celite pad, and the resulting solution was evaporated to dryness. The product was purified using column chromatography on silica gel with CHCl_3_/MeOH = 1:1, resulting in primary aminotriol **31**. Yield: 0.37 g (66%); white crystals; m.p.: 64–68 °C; [*α*]_D_^20^ = –76 (*c* 0.09 MeOH); ^1^H-NMR (500 MHz, CDCl_3_) δ (ppm): 0.74 (s, 3H), 0.85–0.90 (m, 1H), 0.93–0.96 (m, 1H), 0.98–1.05 (m, 3H), 1.08–1.15 (m, 1H), 1.16 (s, 3H), 1.17–1.19 (m, 1H), 1.29–1.35 (m, 1H), 1.38–1.42 (m, 1H), 1.50–1.55 (m, 1H), 1.57–1.62 (m, 1H), 1.63–1.69 (m, 2H), 1.71–1.76 (m, 2H), 1.77–1.79 (m, 1H), 1.80–1.86 (m, 2H), 1.97–2.00 (m, 1H), 2.05 (s, 3H), 2.16 (d, 1H, *J* = 12.9 Hz), 2.45 (d, 1H, *J* = 12.2 Hz), 2.73 (d, 1H, *J* = 12.2 Hz), 3.63 (s, 3H), 4.07 (q, 1H, *J* = 4.9 Hz, 6.3 Hz); ^13^C-NMR (125 MHz, CDCl_3_) δ (ppm): 13.2 (CH_3_), 18.9 (CH_2_), 20.1 (CH_2_), 21.8 (CH_2_), 28.9 (CH_3_), 29.7 (CH_2_), 38.1 (CH_2_), 39.97 (CH_2_), 39.98 (C_q_), 41.5 (CH_2_), 41.7 (CH_2_), 42.4 (C_q_), 43.8 (C_q_), 45.8 (C_q_), 50.9 (CH_2_), 51.1 (CH_3_), 51.5 (CH_2_), 56.6 (CH), 57.2 (CH), 79.5 (CH), 178.1 (C=O). HRMS (ESI+): *m*/*z* calcd. for C_21_H_36_NO_3_^+^ [M + H]^+^ 350.2690; found 350.2676.

### 3.10. (4R,4aS,6aR,8R,9S,11aR,11bS)-Methyl 8-hydroxy-9-(((4-methoxybenzyl)amino) methyl)-4,11b-dimethyltetradecahydro-6a,9-methanocyclohepta[a]naphthalene-4-carboxylate (***32***)

Step 1: To 0.20 g (0.57 mmol) of **31** in anhydrous MeOH (10 mL), 4-methoxybenzaldehyde (100 µL, 0.82 mmol) was added, and the mixture was stirred at 25 °C for 3 h, then evaporated. The resulted crude product was redissolved in MeOH (10 mL), and the solution was stirred for another 2 h. The reaction mixture was concentrated under vacuum.

Step 2: The crude product obtained in step 1 was dissolved in 10 mL of anhydrous MeOH, and NaBH_4_ (43 mg, 1.14 mmol) was added to the solution at 0 °C. The mixture was stirred at 0 °C for 1 h and evaporated, extracted with CH_2_Cl_2_ (30 mL), washed with water (3 × 15 mL) and with brine, dried (Na_2_SO_4_) and concentrated under vacuum. The crude product was purified using column chromatography on silica gel with EtOAc. Yield: 40 mg (15%); white crystals; m.p.: 80–83 °C; [*α*]_D_^20^ = –51 (*c* 0.09 MeOH); ^1^H-NMR (500 MHz, CDCl_3_) δ (ppm): 0.73 (s, 3H), 0.82–0.87 (m, 1H), 0.92–0.99 (m, 2H), 1.00–1.04 (m, 2H), 1.09–1.14 (m, 2H), 1.15 (s, 3H), 1.29–1.31 (m, 1H), 1.36–1.41 (m, 1H), 1.49–1.53 (m, 1H), 1.54–1.65 (m, 3H), 1.66–1.71 (m, 2H), 1.72–1.79 (m, 2H), 1.80–1.83 (m, 1H), 1.96–2.01 (m, 1H), 2.16 (d, 1H, *J* = 13.5 Hz), 2.26 (d, 1H, *J* = 11.4 Hz), 2.28 (s, 2H), 2.66 (d, 1H, *J* = 12.1 Hz), 3.62 (s, 3H), 3.69 (q, 2H, *J* = 12.8 Hz, 20.6 Hz), 3.79 (s, 3H), 4.01 (q, 1H, *J* = 4.9 Hz, 6.4 Hz), 6.85 (d, 2H, *J* = 8.5 Hz), 7.20 (d, 2H, *J* = 8.5 Hz); ^13^C-NMR (125 MHz, CDCl_3_) δ (ppm): 13.1 (CH_3_), 18.9 (CH_2_), 20.0 (CH_2_), 21.8 (CH_2_), 28.9 (CH_3_), 30.3 (CH_2_), 38.05 (CH_2_), 38.06 (C_q_), 39.9 (CH_2_), 40.9 (CH_2_), 41.7 (CH_2_), 42.5 (C_q_), 43.8 (C_q_), 45.4 (C_q_), 51.1 (CH_3_), 51.9 (CH_2_), 54.2 (CH_2_), 55.3 (CH_3_), 56.6 (CH), 57.2 (CH), 58.6 (CH_2_), 80.1 (CH), 113.8 (2×CH), 129.3 (2×CH), 132.0 (C_q_), 158.7 (C_q_), 178.1 (C=O). HRMS (ESI+): *m*/*z* calcd. for C_29_H_44_NO_4_^+^ [M + H]^+^ 470.3265; found 470.3244.

### 3.11. General Procedure for Click Reaction of ***19*** and ***20*** for the Preparation of ***33***–***36***

To a stirred solution of 0.15 g (0.39 mmol) of **19** or **20**, respectively, in a 2:1 ratio of *t*-BuOH/H_2_O (12 mL), CuSO_4_.5H_2_O (5 mol%; 5 mg) as a catalyst, 15 mol% sodium ascorbate (12 mg) and alkyne (0.78 mmol) were added at 25 °C. Stirring was continued for 2 days at 25 °C, then water (20 mL) was added and the mixture was extracted using CH_2_Cl_2_ (3 × 20 mL). The organic phase was dried (Na_2_SO_4_) and evaporated to dryness. Compounds **33**–**36** were purified using column chromatography on a silica gel column with *n*-hexane/EtOAc = 2:1.

#### 3.11.1. (4*R*,4a*S*,6a*R*,9*S*,11a*R*,11b*S*)-Methyl 4,11b-dimethyl-8-oxo-9-((4-phenyl-1*H*-1,2,3-triazol-1-yl)methyl)tetradecahydro-6a,9-methanocyclohepta[a]naphthalene-4-carboxylate (**33**)

The reaction was accomplished from compound **19** using phenylacetylene, as described in the general procedure. Yield: 97 mg (51%); white crystals; m.p.: 137–140 °C; [*α*]_D_^20^ = –61 (*c* 0.07 MeOH); ^1^H-NMR (500 MHz, CDCl_3_) δ (ppm): 0.68 (s, 3H), 0.88–0.93 (m, 1H), 0.98–1.05 (m, 1H), 1.08–1.13 (m, 1H), 1.17 (s, 3H), 1.23–1.26 (m, 2H), 1.41–1.52 (m, 5H), 1.55–1.59 (m, 1H), 1.61 (s, 1H), 1.67–1.73 (m, 3H), 1.76–1.83 (m, 2H), 1.84–1.89 (m, 1H), 2.18 (d, 1H, *J* = 12.9 Hz), 2.67 (q, 1H, *J* = 3.5 Hz, 15.3 Hz), 3.62 (s, 3H), 4.47 (q, 2H, *J* = 14.9 Hz, 49.7 Hz), 7.33 (t, 1H, *J* = 7.6 Hz), 7.42 (t, 2H, *J* = 7.6 Hz), 7.69 (s, 1H), 7.83 (d, 2H, *J* = 7.1 Hz); ^13^C-NMR (125 MHz, CDCl_3_) δ (ppm): 13.2 (CH_3_), 18.9 (CH_2_), 19.7 (CH_2_), 21.5 (CH_2_), 28.8 (CH_3_), 33.7 (CH_2_), 37.8 (CH_2_), 37.9 (C_q_), 39.3 (C_q_), 39.7 (CH_2_), 41.0 (CH_2_), 43.8 (C_q_), 48.4 (CH_2_), 48.8 (CH_2_), 51.3 (CH_3_), 52.4 (CH_2_), 53.9 (CH), 54.7 (C_q_), 56.8 (CH), 120.8 (CH), 125.7 (2×CH), 128.2 (CH), 128.8 (2xCH), 130.5 (C_q_), 144.8 (C_q_), 177.7 (C=O), 219.3 (C=O). HRMS (ESI+): *m*/*z* calcd. for C_29_H_38_N_3_O_3_^+^ [M + H]^+^ 476.2908; found 476.2899.

#### 3.11.2. (4*R*,4a*S*,6a*R*,9*S*,11a*R*,11b*S*)-Methyl 9-((4-benzyl-1*H*-1,2,3-triazol-1-yl)methyl)-4,11b-dimethyl-8-oxotetradecahydro-6a,9-methanocyclohepta[a]naphthalene-4-carboxylate (**34**)

The reaction was accomplished from compound **19** using 3-phenyl-1-propine, as described in the general procedure. Yield: 132 mg (67%); white crystals; m.p.: 172–176 °C; [*α*]_D_^20^ = –72 (*c* 0.097 MeOH); ^1^H-NMR (500 MHz, CDCl_3_) δ (ppm): 0.67 (s, 3H), 0.87–0.92 (m, 1H), 0.98–1.05 (m, 1H), 1.08–1.13 (m, 1H), 1.18 (s, 3H), 1.19–1.25 (m, 2H), 1.33–1.37 (m, 1H), 1.39–1.48 (m, 4H), 1.53–1.57 (m, 1H), 1.62–1.68 (m, 4H), 1.73–1.82 (m, 2H), 1.85–1.89 (m, 1H), 2.18 (d, 1H, *J* = 13.5 Hz), 2.64 (q, 1H, *J* = 3.6 Hz, 15.0 Hz), 3.63 (s, 3H), 4.05 (q, 2H, *J* = 5.7 Hz, 15.5 Hz), 4.36 (q, 2H, *J* = 13.9 Hz, 38.8 Hz), 7.12 (s, 1H), 7.19–7.22 (m, 2H), 7.23–7.32 (m, 3H); ^13^C-NMR (125 MHz, CDCl_3_) δ (ppm): 13.2 (CH_3_), 18.9 (CH_2_), 19.7 (CH_2_), 21.5 (CH_2_), 28.8 (CH_3_), 32.2 (CH_2_), 33.6 (CH_2_), 37.8 (CH_2_), 37.9 (C_q_), 39.3 (C_q_), 39.7 (CH_2_), 41.1 (CH_2_), 43.8 (C_q_), 48.4 (CH_2_), 48.9 (CH_2_), 51.3 (CH_3_), 52.3 (CH_2_), 53.9 (C_q_), 54.6 (CH), 56.8 (CH), 122.7 (CH), 126.5 (2×CH), 128.6 (3×CH), 139.1 (C_q_), 147.6 (C_q_), 177.7 (C=O), 219.2 (C=O). HRMS (ESI+): *m*/*z* calcd. for C_30_H_40_N_3_O_3_^+^ [M + H]^+^ 490.3064; found 490.3056.

#### 3.11.3. (4*R*,4a*S*,6a*R*,8*R*,9*S*,11a*R*,11b*S*)-Methyl 8-hydroxy-4,11b-dimethyl-9-((4-phenyl-1*H*-1,2,3-triazol-1-yl)methyl)tetradecahydro-6a,9-methanocyclohepta[a]naphthalene-4-carboxylate (**35**)

The reaction was accomplished from compound **20** using phenylacetylene, as described in the general procedure. Yield: 86 mg (45%); white crystals; m.p.: 154–156 °C; [*α*]_D_^20^ = –59 (*c* 0.07 MeOH); ^1^H-NMR (500 MHz, CDCl_3_) δ (ppm): 0.72 (s, 3H), 0.82–0.87 (m, 1H), 0.94–1.00 (m, 1H), 1.01–1.05 (m, 2H), 1.09–1.13 (m, 1H), 1.16 (s, 3H), 1.26 (s, 1H), 1.31–1.35 (m, 1H), 1.36–1.39 (m, 1H), 1.43–1.47 (m, 1H), 1.49–1.54 (m, 1H), 1.58–1.65 (m, 3H), 1.68–1.73 (m, 2H), 1.76–1.85 (m, 3H), 1.89–1.94 (m, 1H), 2.16 (d, 1H, *J* = 11.1 Hz), 2.55 (s, 1H), 3.62 (s, 3H), 4.24 (q, 2H, *J* = 14.0 Hz, 24.5 Hz), 4.28 (s, 1H, overlapping with quartet CH_2_), 7.34 (t, 1H, *J* = 7.5 Hz), 7.43 (t, 2H, *J* = 7.5 Hz), 7.69 (s, 1H), 7.79 (d, 2H, *J* = 7.1 Hz); ^13^C-NMR (125 MHz, CDCl_3_) δ (ppm): 13.1 (CH_3_), 18.9 (CH_2_), 19.7 (CH_2_), 21.7 (CH_2_), 28.9 (CH_3_), 29.8 (CH_2_), 37.9 (CH_2_), 38.1 (C_q_), 39.9 (CH_2_), 41.5 (CH_2_), 41.8 (C_q_), 42.3 (CH_2_), 43.8 (C_q_), 46.1 (C_q_), 51.2 (CH_3_), 51.7 (CH_2_), 55.9 (CH), 57.1 (CH), 58.5 (CH_2_), 77.3 (CH), 121.5 (CH), 125.7 (2xCH), 128.3 (CH), 128.9 (2×CH), 130.4 (C_q_), 147.4 (C_q_), 177.9 (C=O). HRMS (ESI+): *m*/*z* calcd. for C_29_H_40_N_3_O_3_^+^ [M + H]^+^ 478.3064; found 478.3057.

#### 3.11.4. (4*R*,4a*S*,6a*R*,8*R*,9*S*,11a*R*,11b*S*)-Methyl 9-((4-benzyl-1*H*-1,2,3-triazol-1-yl)methyl)-8-hydroxy-4,11b-dimethyltetradecahydro-6a,9-methanocyclohepta[a]naphthalene-4-carboxylate (**36**)

The reaction was accomplished from compound **20** using 3-phenyl-1-propine, as described in the general procedure. Yield: 82 mg (42%); white crystals; m.p.: 112–114 °C; [*α*]_D_^20^ = –61 (*c* 0.1 MeOH); ^1^H-NMR (500 MHz, CDCl_3_) δ (ppm): 0.72 (s, 3H), 0.81–0.88 (m, 1H), 0.98–1.10 (m, 5H), 1.16 (s, 3H), 1.29–1.35 (m, 1H), 1.36–1.42 (m, 2H), 1.50–1.54 (m, 1H), 1.55–1.60 (m, 2H), 1.61–1.66 (m, 2H), 1.70 (d, 1H, *J* = 12.6 Hz), 1.76–1.83 (m, 3H), 1.87–1.92 (m, 1H), 2.16 (d, 1H, *J* = 13.2 Hz), 2.75 (s, 1H), 3.62 (s, 3H), 4.09 (q, 2H, *J* = 13.7 Hz, 36.0 Hz), 4.08 (s, 2H, overlapping with quartet CH_2_), 4.25 (d, 1H, *J* = 10.3 Hz), 7.09 (s, 1H), 7.21–7.26 (m, 3H), 7.28–7.33 (m, 2H); ^13^C-NMR (125 MHz, CDCl_3_) δ (ppm): 13.1 (CH_3_), 18.9 (CH_2_), 19.7 (CH_2_), 21.7 (CH_2_), 28.9 (CH_3_), 29.7 (CH_2_), 32.2 (CH_2_), 38.00 (CH_2_), 38.04 (C_q_), 39.9 (CH_2_), 41.5 (CH_2_), 41.8 (C_q_), 42.2 (CH_2_), 43.8 (C_q_), 45.9 (C_q_), 51.2 (CH_3_), 51.8 (CH_2_), 56.0 (CH), 57.1 (CH), 58.5 (CH_2_), 77.5 (CH), 123.6 (CH), 126.5 (CH), 128.6 (2×CH), 128.7 (2×CH), 138.9 (C_q_), 147.2 (C_q_), 177.9 (C=O). HRMS (ESI+): *m*/*z* calcd. for C_30_H_42_N_3_O_3_^+^ [M + H]^+^ 492.3221; found 492.3210.

### 3.12. Determination of the Antiproliferative Activities

The in vitro anticancer effects of the presented compounds were assessed using the standard MTT [3-(4,5-dimethylthiazol2-yl)-2,5-diphenyltetrazolium bromide] assay on the following human cancer cell lines: cervical (HeLa and SiHa), breast (MCF-7 and MDA-MB-231) and ovarian cancer (A2780) [39]. The selectivity of the tested compounds was determined on murine embryonal fibroblast cells (NIH/3T3). The utilised cell lines were purchased from the European Collection of Cell Cultures (Salisbury, UK). The cells were cultivated in Eagle’s minimal essential medium supplemented with 10% fetal bovine serum, 1% non-essential amino acids and 1% antibiotic-antimycotic complex (penicillin, streptomycin, amphotericin B) at 37 °C in a humidified atmosphere containing 5% CO_2_. All media and supplements were purchased from Lonza Group Ltd. (Basel, Switzerland). The cancer cells were seeded into 96-well plates (5000 cells/well) and incubated overnight, and the test compounds were applied in two different concentrations (10 µM and 30 µM) and incubated for another 72 h under cell-culturing conditions. [24] IC_50_ determinations for the most active compounds required 6 gradually increasing concentrations (0.1 µM, 0.3 µM, 1.0 µM, 3.0 µM, 10,0 µM and 30.0 µM). After 72 h, 20 µL of the 5 mg/mL MTT solution was added to each well, and the plates were incubated for 4 h. The medium was removed, and the formazan crystals produced by the mitochondrial activity were dissolved in dimethyl sulfoxide (DMSO) via shaking at 37 °C for 30 min. The absorbances were measured at 545 nm using a microplate reader (SPECTROStar Nano, BMG Labtech, Offenburg, Germany). The reference agent was cisplatin, which is a widely used cytotoxic agent. The IC_50_ values were calculated using sigmoid curve fitting. Two independent experiments were performed with five parallel wells for each condition. Calculations were performed using the GraphPad Prism 9 software (GraphPad Software Inc., San Diego, CA, USA).

## 4. Conclusions

A series of novel diterpene-type chiral 1,3-aminoalcohols has been synthesised from natural stevioside in a stereoselective manner. The key intermediate β-keto alcohol was prepared via Wagner–Meerwein rearrangement of the epoxide derived from steviol methyl ester. The primary aminoalcohol was formed using Raney-nickel-catalysed hydrogenation of the oxime, and a versatile library of aminoalcohols was synthesised via the Schiff base with primary amines. The regioisomeric aminoalcohols were prepared from the mesylate of the β-keto alcohol via reduction of the keto function, followed by Ms → NHR exchange. Both the reduction of the *O*-mesyl ketone and Schiff bases during the formation of new chiral centres occurred stereoselectively, resulting in the formation of single diastereoisomers. The corresponding primary aminoalcohol was formed via the palladium-catalysed hydrogenation of hydroxyl-azide, from which click reactions were also carried out. The antiproliferative effects were assayed using the MTT method, whereas several *N*-substituted derivatives showed remarkable inhibition of cell growth on the human cancer cell lines (HeLa, SiHa, A2780, MCF-7 and MDA-MB-231). A significant difference was observed in the antiproliferative activity between the regioisomers. Compared with our former results with previously prepared *ent*-beyerane-based 1,3-aminoalcohols [21], similar antiproliferative activity but higher selectivity was observed. Compound **16** exerted outstanding activities against the malignant cells with limited action on the fibroblasts, indicating considerable cancer selectivity. This latter agent seems to be superior to the clinically utilised cisplatin. Consequently, it could be regarded as a potential hit compound and may be subjected to further investigation.

## Figures and Tables

**Table 1:** Stereoselective syntheses of steviol-derived *N*-substituted aminoalcohols.

Entry	Product	R	Yield [%]
1	**8**	benzyl	43
2	**9**	4-fluorobenzyl	48
3	**10**	4-methoxybenzyl	70
4	**11**	(*R*)-α-ethylbenzyl	65
5	**12**	(*S*)-α-ethylbenzyl	60
6	**13**	(*R*)-1-(1-naphthyl)ethyl	41
7	**14**	(*S*)-1-(1-naphthyl)ethyl	38
8	**15**	1-naphthylmethyl	78
9	**16**	(*R*)-1-(2-naphthyl)ethyl	60
10	**17**	(*S*)-1-(2-naphthyl)ethyl	56

**Table 2:** Stereoselective syntheses of *N*-substituted aminoalcohol regioisomers.

Entry	Product	R	Yield [%]
1	**22**	benzyl	22
2	**23**	4-fluorobenzyl	41
3	**24**	(*R*)-α-ethylbenzyl	40
4	**25**	(*S*)-α-ethylbenzyl	36
5	**26**	1-naphthylmethyl	44
6	**27**	(*R*)-1-(1-naphthyl)ethyl	29
7	**28**	(*S*)-1-(1-naphthyl)ethyl	21
8	**29**	(*R*)-1-(2-naphthyl)ethyl	19
9	**30**	(*S*)-1-(2-naphthyl)ethyl	15

## Data Availability

Data are contained within the article and Supplementary Materials.

## Supplementary Materials

The supporting information, Table S1, Figure S1 and 1H, 13C J-MOD, 19F, COSY, NOESY, HSQC and HMBC NMR spectra of the new compounds can be downloaded at https://www.mdpi.com/article/10.3390/molecules28247962/s1.
