# Supplementary material for: Stereoselective Synthesis and Antiproliferative Activity of Steviol-Based Diterpene 1,3-Aminoalcohol Regioisomers
Source: Molecules. 2023 Dec 5;28(24):7962. doi: 10.3390/molecules28247962 (PMC10745768; doi:10.3390/molecules28247962)

Supporting informations
for

Stereoselective Synthesis and Antiproliferative Activity of Steviol-Based Diterpene 1,3-Aminoalcohol Regioisomers

Dorottya Bai[1], Zsuzsanna Schelz[2], Mária Fanni Boncz[1], István Zupkó[2,3], and Zsolt Szakonyi[1,3,*]

[1] Interdisciplinary Excellence Center, Institute of Pharmaceutical Chemistry, University of Szeged, Eötvös utca 6, H-6720 Szeged, Hungary; baidorottya@gmail.com; bonczmaria@gmail.com

[2] Department of Pharmacodynamics and Biopharmacy, University of Szeged, Eötvös utca 6, H-6720 Szeged, Hungary; schelz.zsuzsanna@szte.hu; zupko.istvan@szte.hu

[3] Interdisciplinary Centre of Natural Products, University of Szeged, Eötvös utca 6, H-6720 Szeged, Hungary

* Correspondence: szakonyi.zsolt@szte.hu; Tel.: +36-62-546809

Contents

Table S1. Antiproliferative effects of the investigated compounds 3 – 4

Concentration–response curves of compound **16**, **17**, **23** and **32** 5

^{1}H, ^{13}C, ^{19}F NMR, COSY, NOESY, HSQC, HMBC spectra of compounds **3** - **36** 6 - 213

HRMS spectra of compounds **3** -**36** 214 - 247

Table S1. Antiproliferative effects of the investigated compounds

Comp.	Cc. (μM)	Growth inhibition (%) ± SEM and calculated IC_{50} values (μM)*					
		HeLa	SiHa	MCF-7	MDA-MB-231	A2780	NIH/3T3
**6**	10	23.23 ± 1.97	< 20	36.44 ± 3.48	25.84 ± 2.49	24.82 ± 5.19	22.06 ± 1.54
	30	93.94 ± 0.55	68.24 ± 0.61	98.58 ± 0.22	97.60 ± 0.67	82.50 ± 4.66	98.27 ± 0.37
**7**	10	24.87 ± 2.49	< 20	43.12 ± 3.79	28.03 ± 1.84	27.03 ± 4.31	< 20
	30	96.89 ± 0.54	88.56 ± 2.01	99.33 ± 0.24	97.67 ± 0.67	91.63 ± 1.97	97.76 ± 0.70
**8**	10	< 20	< 20	45.43 ± 1.94	< 20	20.30 ± 2.05	25.69 ± 0.97
	30	97.49 ± 0.52	95.34 ± 1.23	98.90 ± 0.18	97.89 ± 0.66	98.25 ± 0.40	98.27 ± 0.37
**9**	10	< 20	< 20	79.84 ± 1.59	57.99 ± 2.59	73.56 ± 2.61	< 20
	30	95.35 ± 0.58	85.99 ± 2.92	98.07 ± 0.35	93.42 ± 0.39	99.01 ± 0.11	93.88 ± 1.94
**10**	10	41,90 ± 4.29	< 20	86.52 ± 0.88	78.67 ± 2.63	38.96 ± 1.87	20.31 ± 2.93
	30	97.65 ± 0.64	97.61 ± 0.90	100.0 ± 0.20	98.09 ± 0.59	98.38 ± 0.51	96.59 ± 1.10
**11**	10	< 20	< 20	49.48 ± 3.06	< 20	74.25 ± 2.10	22.16 ± 2.22
	30	94.48 ± 0.84	83.71 ± 3.19	96.94 ± 0.57	90.78 ± 1.35	98.12 ± 0.30	93.61 ± 2.02
**12**	10	< 20	< 20	48.62 ± 0.50	< 20	64.73 ± 1.95	29.23 ± 1.33
	30	90.80 ± 3.71	82.43 ± 2.09	92.35 ± 2.89	91.59 ± 2.36	98.32 ± 0.23	95.53 ± 0.49
**13**	10	< 20	24.18 ± 3.46	56.73 ± 3.88	<20	83.65 ± 1.95	< 20
	30	92.34 ± 2.70	87.92 ± 1.62	96.84 ± 0.55	94.30 ± 1.83	99.02 ± 0.39	96.88 ± 0.74
**14**	10	24.62 ± 2.79	34.15 ± 2.53	78.87 ± 4.11	53.21 ± 2.44	93.33 ± 1.99	50.36 ± 2.91
	30	95.13 ± 1.60	88.88 ± 0.87	97.44 ± 0.53	94.95 ± 1.24	99.57 ± 0.35	97.15 ± 0.60
**15**	10	< 20	< 20	53.99 ± 3.39	31.33 ± 1.89	89.51 ± 1.76	< 20
	30	94.88 ± 2.04	87.89 ± 1.33	97.43 ± 0.59	94.23 ± 0.98	99.67 ± 0.49	96.50 ± 0.77
**16**	10	55.42 ± 2.81	80.05 ± 1.41	80.95 ± 2.03	84.43 ± 2.92	97.64 ± 2.81	< 20
	30	95.69 ± 1.12	89.39 ± 1.03	97.95 ± 0.56	93.61 ± 1.23	100.3 ± 0.57	96.83 ± 0.66
	IC_{50}	3.40	6.33	3.88	4.51	4.43	17.44
**17**	10	90.55 ± 1.34	83.33 ± 1.58	86.31 ± 2.86	91.46 ± 1.24	100.6 ± 0.76	20.50 ± 1.70
	30	93.53 ± 2.50	86.66 ± 1.74	97.42 ± 0.71	95.03 ± 0.91	100.0 ± 0.91	96.54 ± 0.62
	IC_{50}	3.51	5.37	2.47	5.62	4.06	8.70
**19**	10	31.57 ± 1.34	< 20	< 20	< 20	< 20	< 20
	30	46.86 ± 0.27	< 20	38.02 ± 1.36	< 20	41.72 ± 1.99	< 20
**20**	10	< 20	< 20	< 20	< 20	< 20	< 20
	30	< 20	< 20	30.71 ± 0.93	< 20	27.04 ± 2.25	< 20
**22**	10	59.22 ± 6.61	69.00 ± 2.02	90.56 ± 0.98	90.95 ± 0.40	69.51 ± 3.98	49.04 ± 1.37
	30	90.87 ± 0.35	80.80 ± 2.91	94.23 ± 0.82	89.66 ± 0.28	96.04 ± 0.45	94.25 ± 0.45
**23**	10	70.14 ± 1.97	68.12 ± 1.85	95.81 ± 0.84	92.99 ± 0.39	79.97 ± 1.60	81.84 ± 3.07
	30	90.41 ± 0.78	80.48 ± 3.16	95.33 ± 1.05	91.40 ± 0.39	95.71 ± 0.58	93.62 ± 0.77
	IC_{50}	5.07	4.41	1.59	3.28	4.39	5.15
**24**	10	< 20	< 20	34.80 ± 1.99	< 20	< 20	21.83 ± 2.15
	30	96.56 ± 0.88	78.10 ± 1.56	93.61 ± 0.94	97.59 ± 1.01	99.28 ± 0.52	97.69 ± 0.87
**25**	10	< 20	< 20	40.09 ± 1.44	< 20	< 20	< 20
	30	32.24 ± 2.88	< 20	78.81 ± 1.52	69.28 ± 1.96	73.83 ± 0.57	93.70 ± 1.11
**26**	10	49.50 ± 1.23	63.39 ± 0.91	90.70 ± 1.57	87.61 ± 0.59	61.39 ± 1.45	25.74 ± 1.93
	30	93.63 ± 1.56	86.89 ± 1.69	97.49 ± 0.53	93.09 ± 1.53	98.21 ± 0.43	95.62 ± 0.96
**27**	10	< 20	< 20	79.62 ± 2.89	67.25 ± 1.94	59.04 ± 1.68	< 20
	30	96.38 ± 0.83	86.89 ± 2.27	97.63 ± 0.31	93.69 ± 0.42	98.92 ± 0.22	93.97 ± 1.64
**28**	10	46.83 ± 1.44	63.43 ± 2.66	90.63 ± 0.54	88.00 ± 0.54	60.10 ± 2.24	< 20
	30	94.66 ± 0.63	86.13 ± 3.79	97.75 ± 0.39	92.92 ± 1.04	98.53 ± 0.42	93.97 ± 1.95
**29**	10	< 20	< 20	32.51 ± 0.92	< 20	21.89 ± 0.73	< 20
	30	92.17 ± 0.38	79.47 ± 2.33	93.08 ± 0.30	95.30 ± 0.97	74.95 ± 1.79	32.03 ± 1.30
**30**	10	40.79 ± 0.99	< 20	79.51 ± 1.01	50.73 ± 3.21	33.06 ± 1.23	21.47 ± 0.48
	30	94.51 ± 0.19	101.40 ± 0.6	95.63 ± 0.55	98.70 ± 0.49	99.71 ± 0.27	100.70 ± 0.2
**31**	10	< 20	< 20	72.69 ± 3.15	49.47 ± 3.47	37.30 ± 1.04	< 20
	30	96.02 ± 0.89	95.68 ± 0.81	99.42 ± 0.23	96.82 ± 0.65	97.52 ± 0.80	97.62 ± 0.71
**32**	10	97.09 ± 0.79	95.59 ± 2.12	99.59 ± 0.13	97.12 ± 0.60	97.94 ± 0.60	96.93 ± 0.92

	30	97.18 ± 0.76	96.46 ± 1.09	99.88 ± 0.23	96.32 ± 1.02	98.01 ± 0.60	96.61 ± 1.05
	IC_{50}	3.09	5.77	1.04	2.30	3.78	3.71
**33**	10	< 20	< 20	57.13 ± 2.13	< 20	41.14 ± 1.23	54.53 ± 1.93
	30	23.94 ± 5.01	< 20	60.27 ± 3.65	< 20	51.53 ± 1.41	78.89 ± 2.52
**34**	10	< 20	< 20	37.49 ± 2.10	< 20	31.77 ± 1.60	< 20
	30	75.20 ± 1.26	58.82 ± 2.24	81.29 ± 2.02	50.12 ± 2.41	89.59 ± 0.45	72.46 ± 0.96
**35**	10	< 20	< 20	69.95 ± 3.11	< 20	43.21 ± 1.63	54.79 ± 1.40
	30	88.11 ± 0.75	81.41 ± 2.84	88.79 ± 0.44	92.44 ± 0.59	97.37 ± 0.38	93.02 ± 1.01
**36**	10	< 20	< 20	45.89 ± 3.40	< 20	29.33 ± 1.68	< 20
	30	89.32 ± 0.63	79.36 ± 3.17	91.80 ± 0.74	91.38 ± 0.65	96.31 ± 0.53	92.91 ± 0.87
Cisplatin**	10	42.61 ± 2.33	60.98 ± 0.92	53.03 ± 2.29	67.51 ± 1.01	83.57 ± 1.21	73.88 ± 1.63
	30	99.93 ± 0.26	88.95 ± 0.53	86.90 ± 1.22	87.75 ± 1.10	95.02 ± 0.28	97.10 ± 0.15

*: for selected outstandingly active compounds; **: data from reference 10.3390/ijms24021121 (Bai D, Int J Mol Sci 2023, Lit. 24 in manuscript)

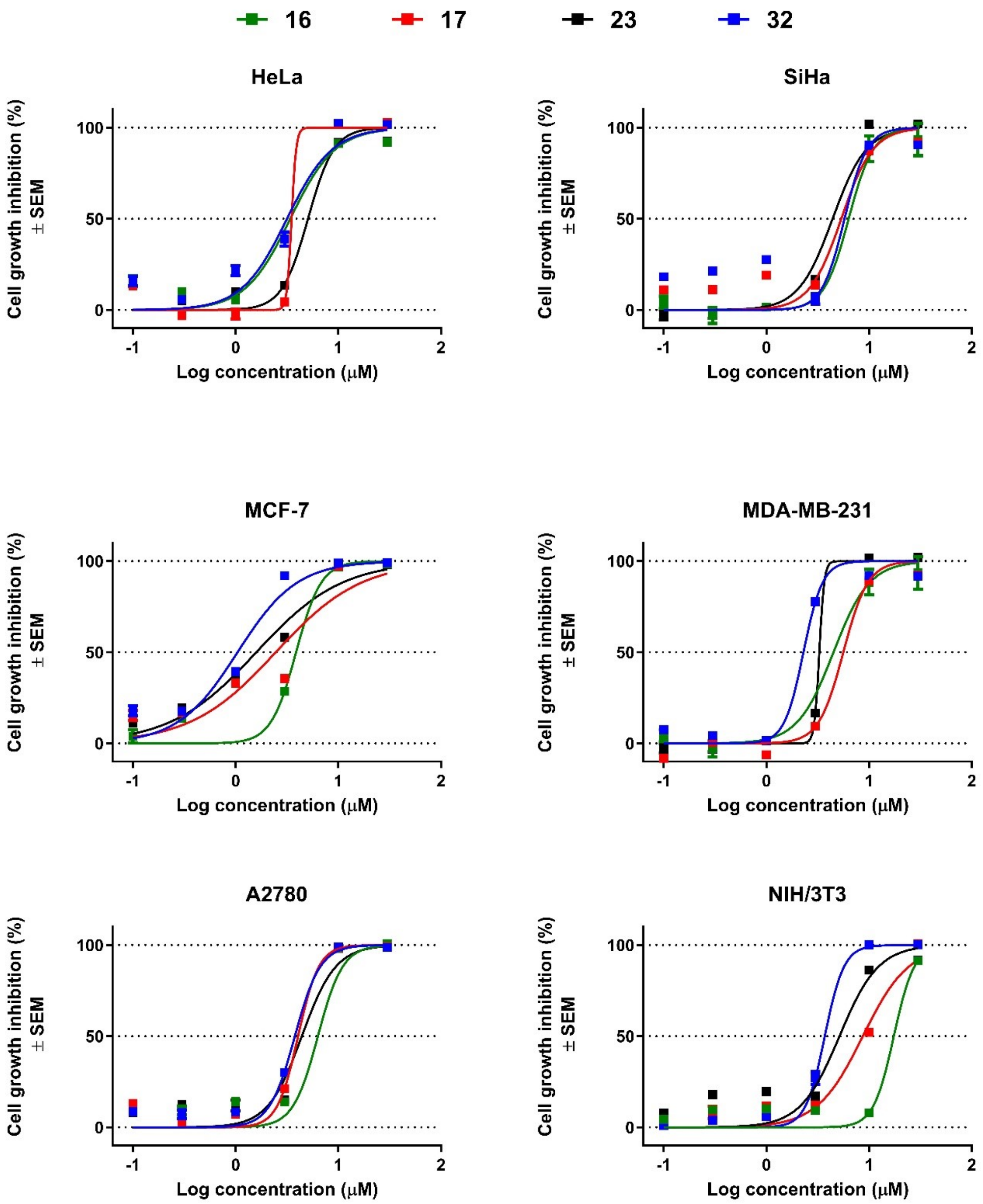

Figure S1 Concentration–response curves of *antiproliferative* activity for compound **16**, **17**, **23** and **32**

^{1}H-NMR of compound (2'*S*,4*R*,4a*S*,6a*S*,11a*R*,11b*S*)-methyl 9-hydroxy-4,11b-dimethyldodecahydro-1*H*-spiro[6a,9-methanocyclohepta[a]naphthalene-8,2'-oxirane]-4-carboxylate **(3)**

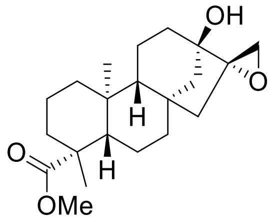

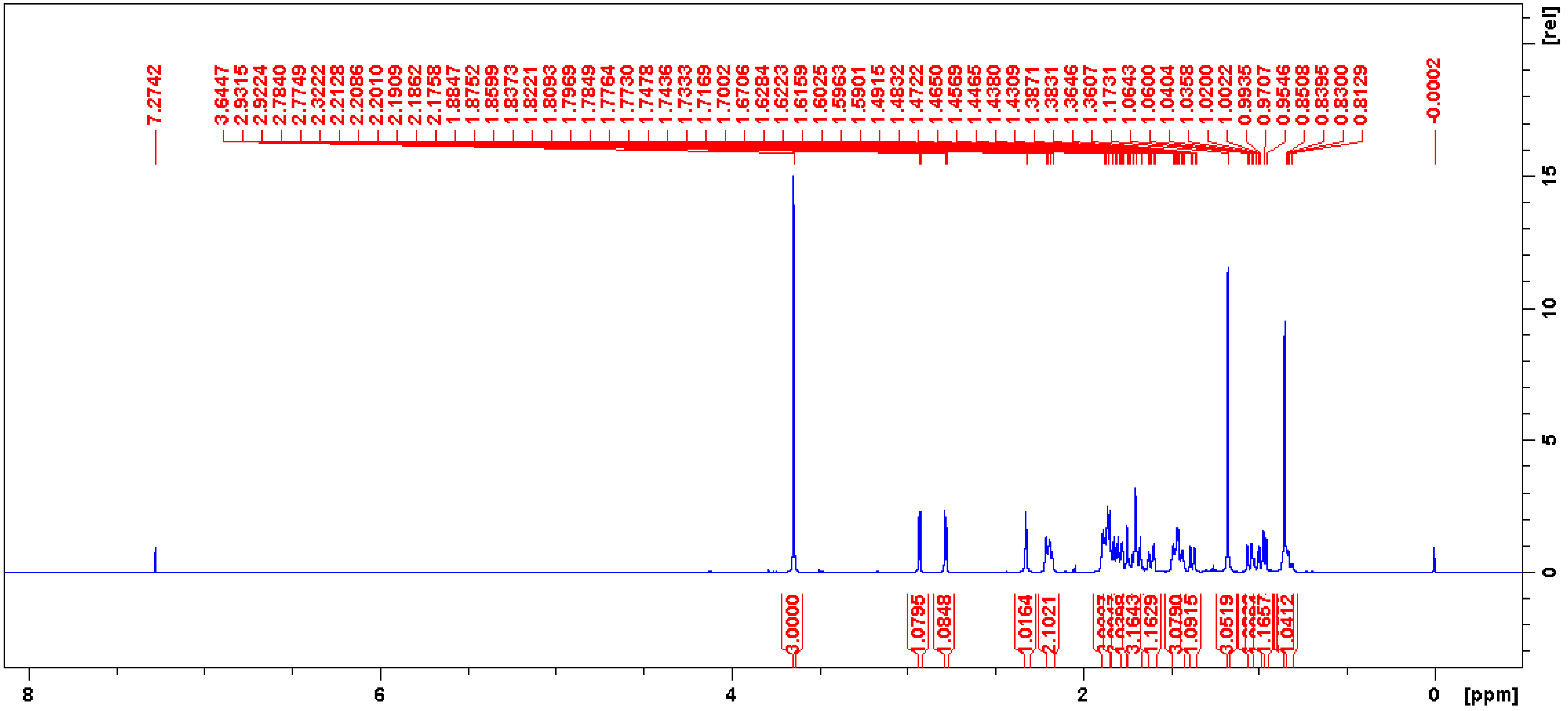

^{13}C-NMR of compound (2'*S*,4*R*,4a*S*,6a*S*,11a*R*,11b*S*)-methyl 9-hydroxy-4,11b-dimethyldodecahydro-1*H*-spiro[6a,9-methanocyclohepta[a]naphthalene-8,2'-oxirane]-4-carboxylate **(3)**

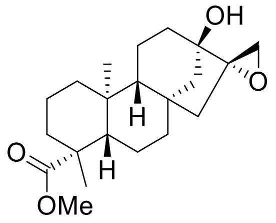

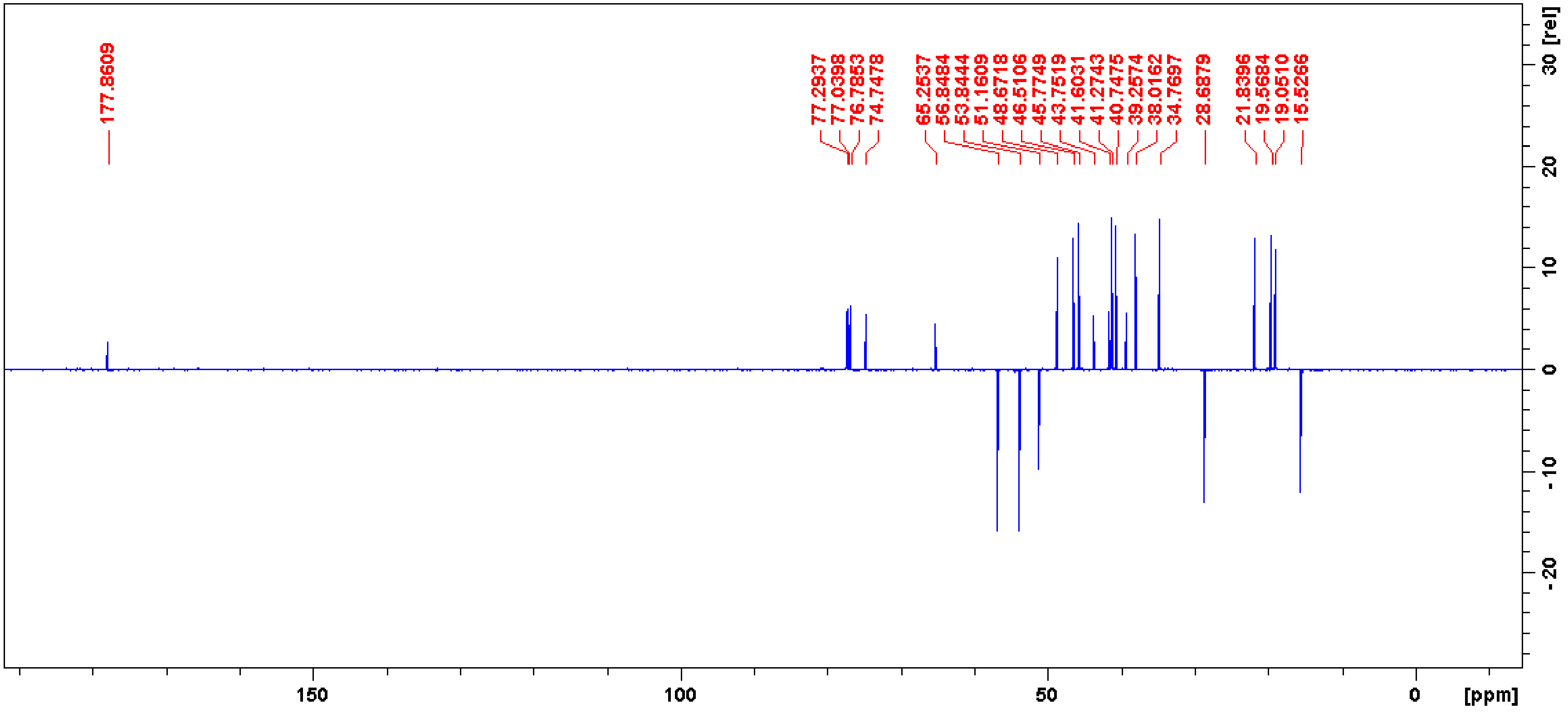

COSY of compound (2'*S*,4*R*,4a*S*,6a*S*,11a*R*,11b*S*)-methyl 9-hydroxy-4,11b-dimethyldodecahydro-1*H*-spiro[6a,9-methanocyclohepta[a]naphthalene-8,2'-oxirane]-4-carboxylate **(3)**

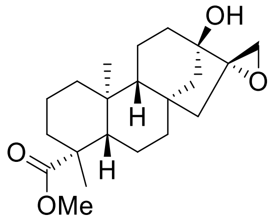

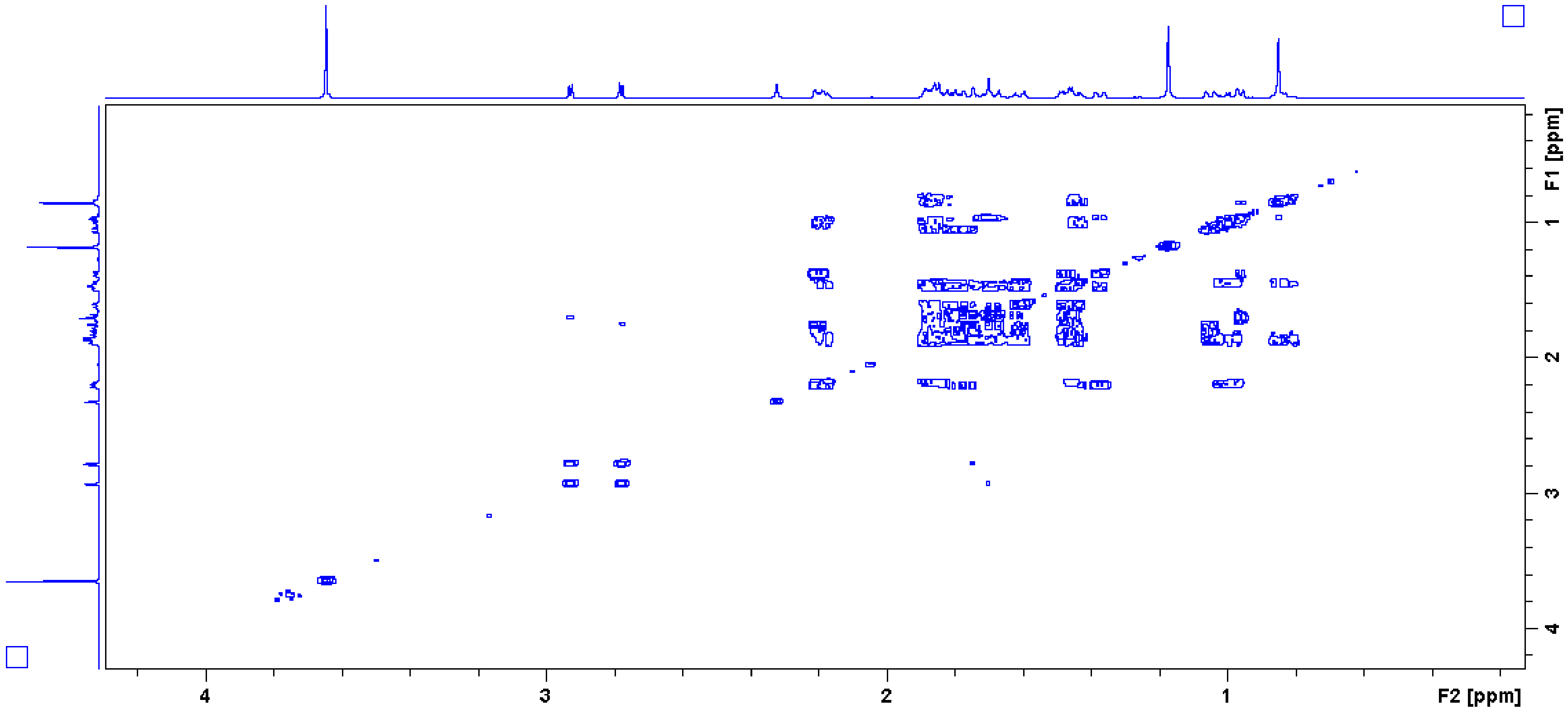

NOESY of compound (2'*S*,4*R*,4a*S*,6a*S*,11a*R*,11b*S*)-methyl 9-hydroxy-4,11b-dimethyldodecahydro-1*H*-spiro[6a,9-methanocyclohepta[a]naphthalene-8,2'-oxirane]-4-carboxylate **(3)**

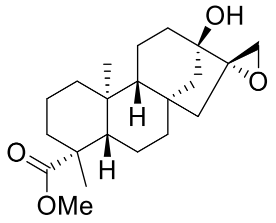

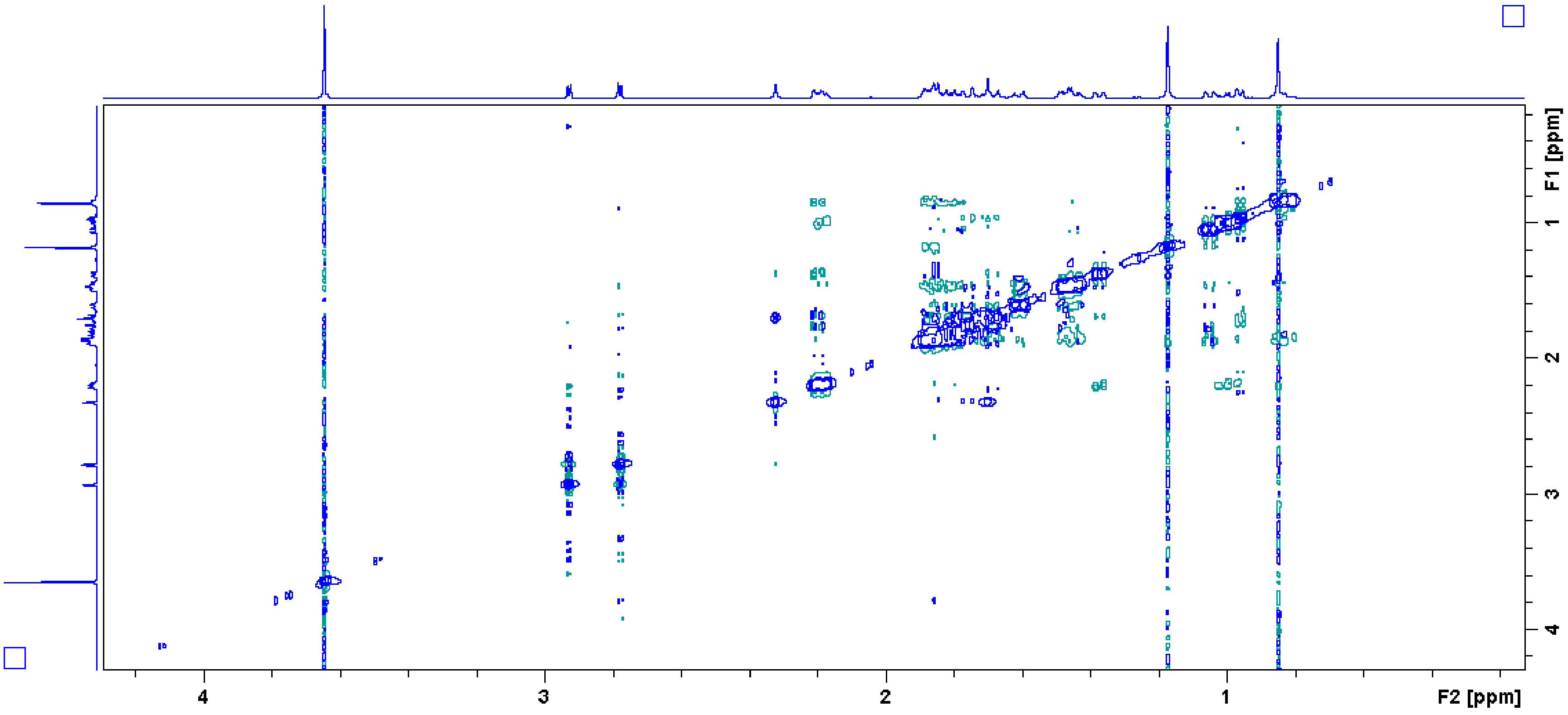

HSQC of compound (2'*S*,4*R*,4a*S*,6a*S*,11a*R*,11b*S*)-methyl 9-hydroxy-4,11b-dimethyldodecahydro-1*H*-spiro[6a,9-methanocyclohepta[a]naphthalene-8,2'-oxirane]-4-carboxylate **(3)**

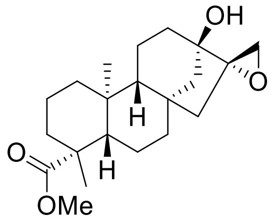

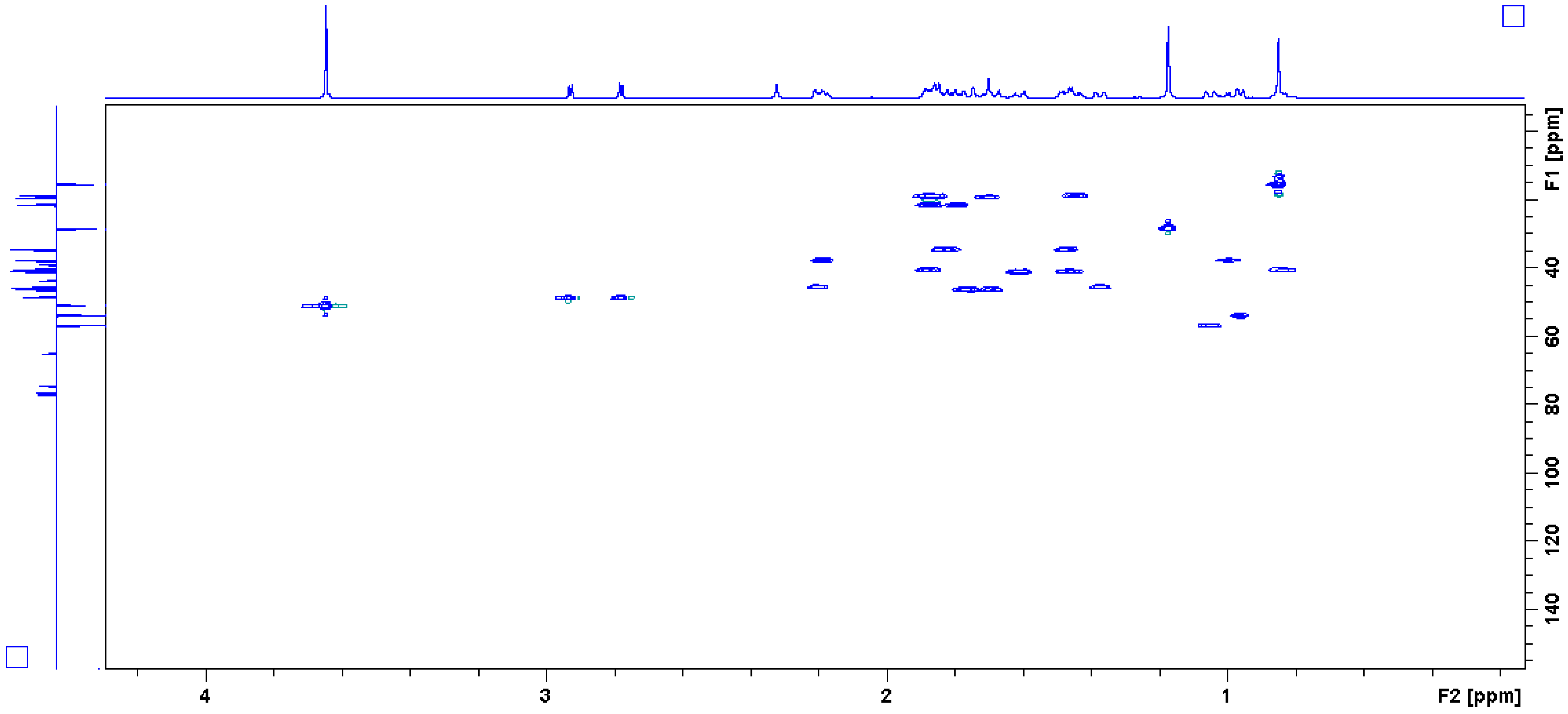

HMBC of compound (2'*S*,4*R*,4a*S*,6a*S*,11a*R*,11b*S*)-methyl 9-hydroxy-4,11b-dimethyldodecahydro-1*H*-spiro[6a,9-methanocyclohepta[a]naphthalene-8,2'-oxirane]-4-carboxylate **(3)**

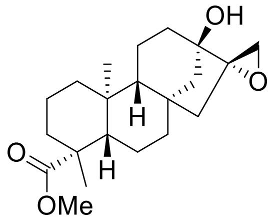

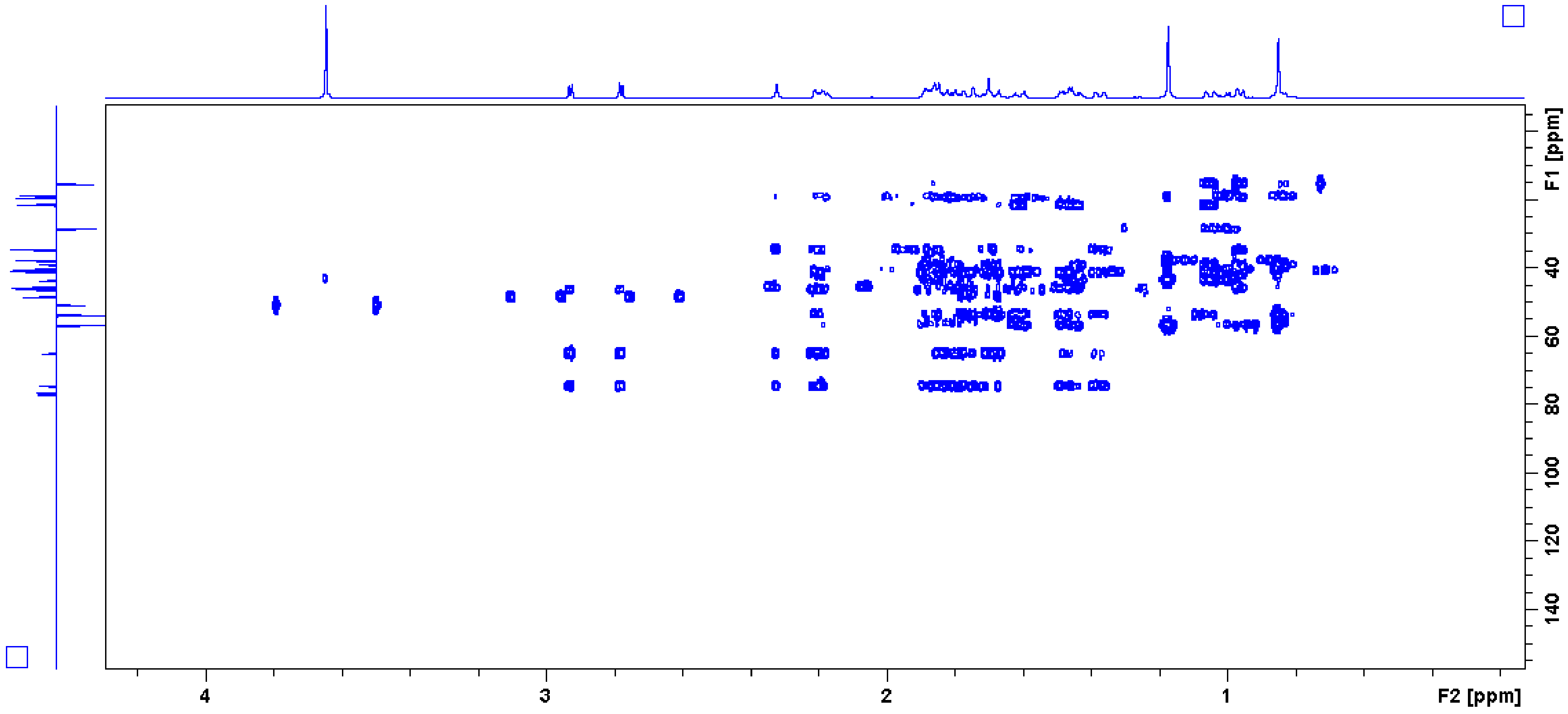

^{1}H-NMR of compound (4*R*,4a*S*,6a*R*,9*S*,11a*R*,11b*S*)-methyl 9-(hydroxymethyl)-4,11b-dimethyl-8-oxotetradecahydro-6a,9-methanocyclohepta[a]naphthalene-4-carboxylate **(4)**

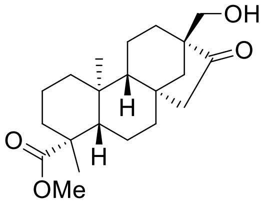

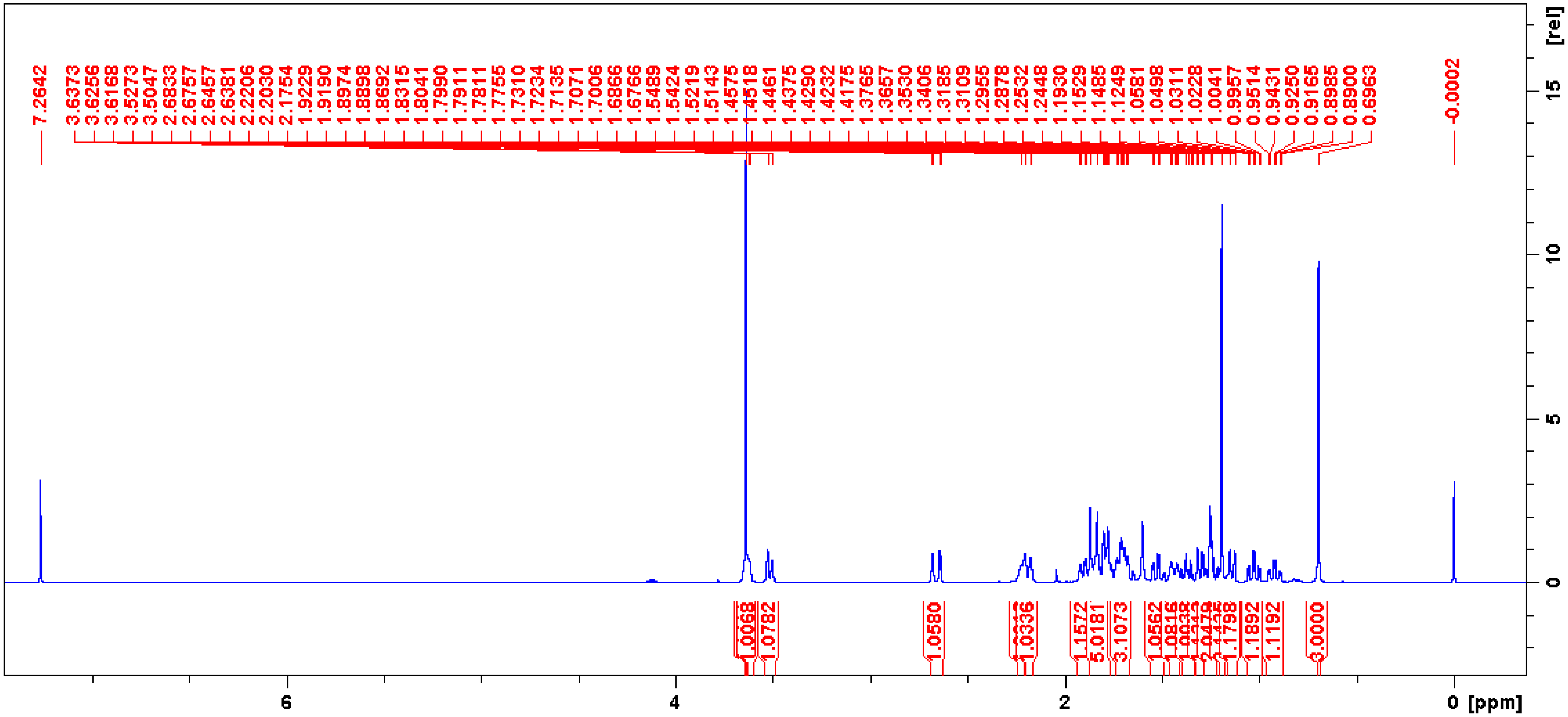

^{13}C-NMR of compound (4*R*,4a*S*,6a*R*,9*S*,11a*R*,11b*S*)-methyl 9-(hydroxymethyl)-4,11b-dimethyl-8-oxotetradecahydro-6a,9-methanocyclohepta[a]naphthalene-4-carboxylate **(4)**

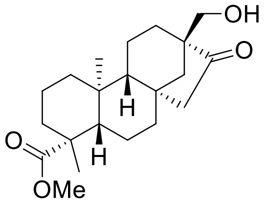

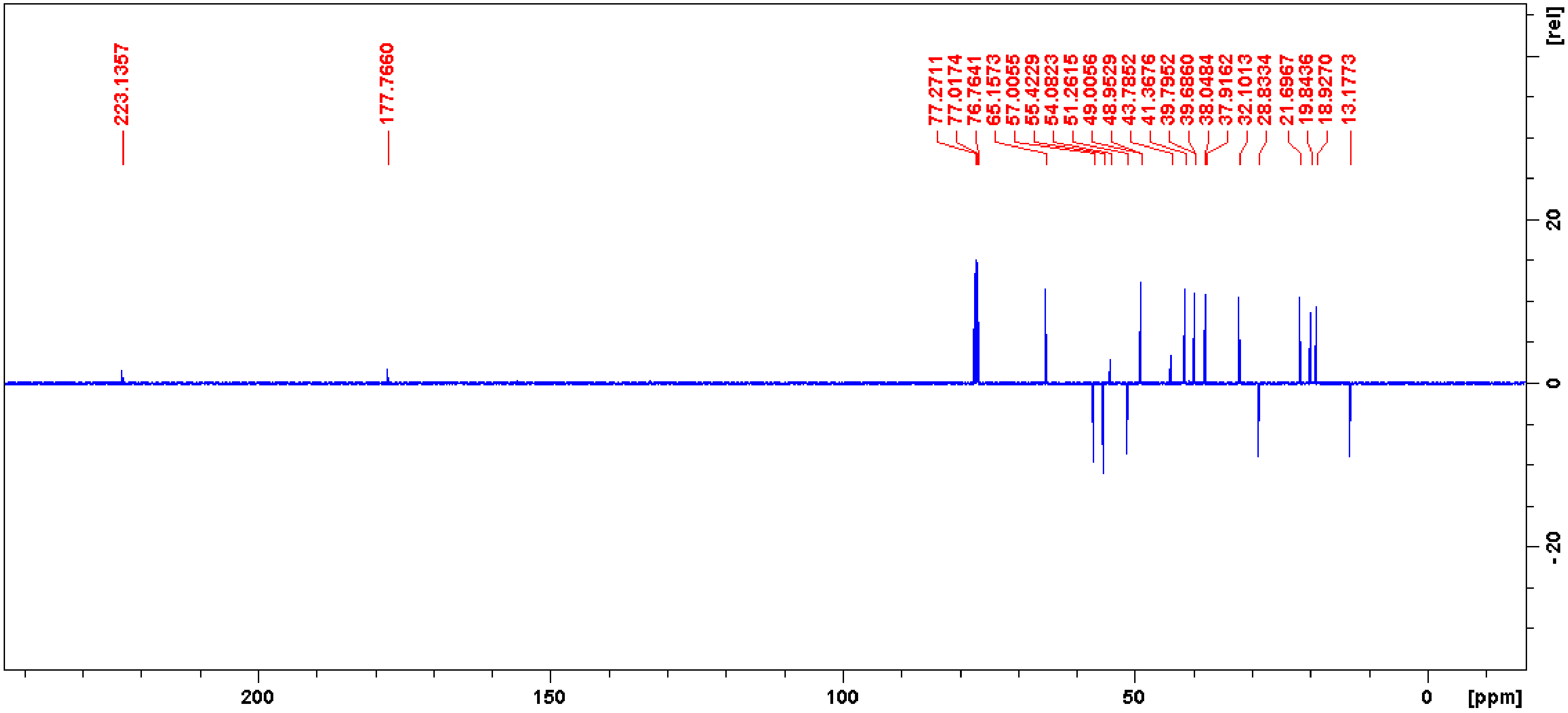

COSY of compound (4*R*,4a*S*,6a*R*,9*S*,11a*R*,11b*S*)-methyl 9-(hydroxymethyl)-4,11b-dimethyl-8-oxotetradecahydro-6a,9-methanocyclohepta[a]naphthalene-4-carboxylate **(4)**

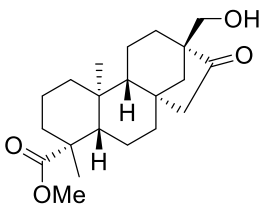

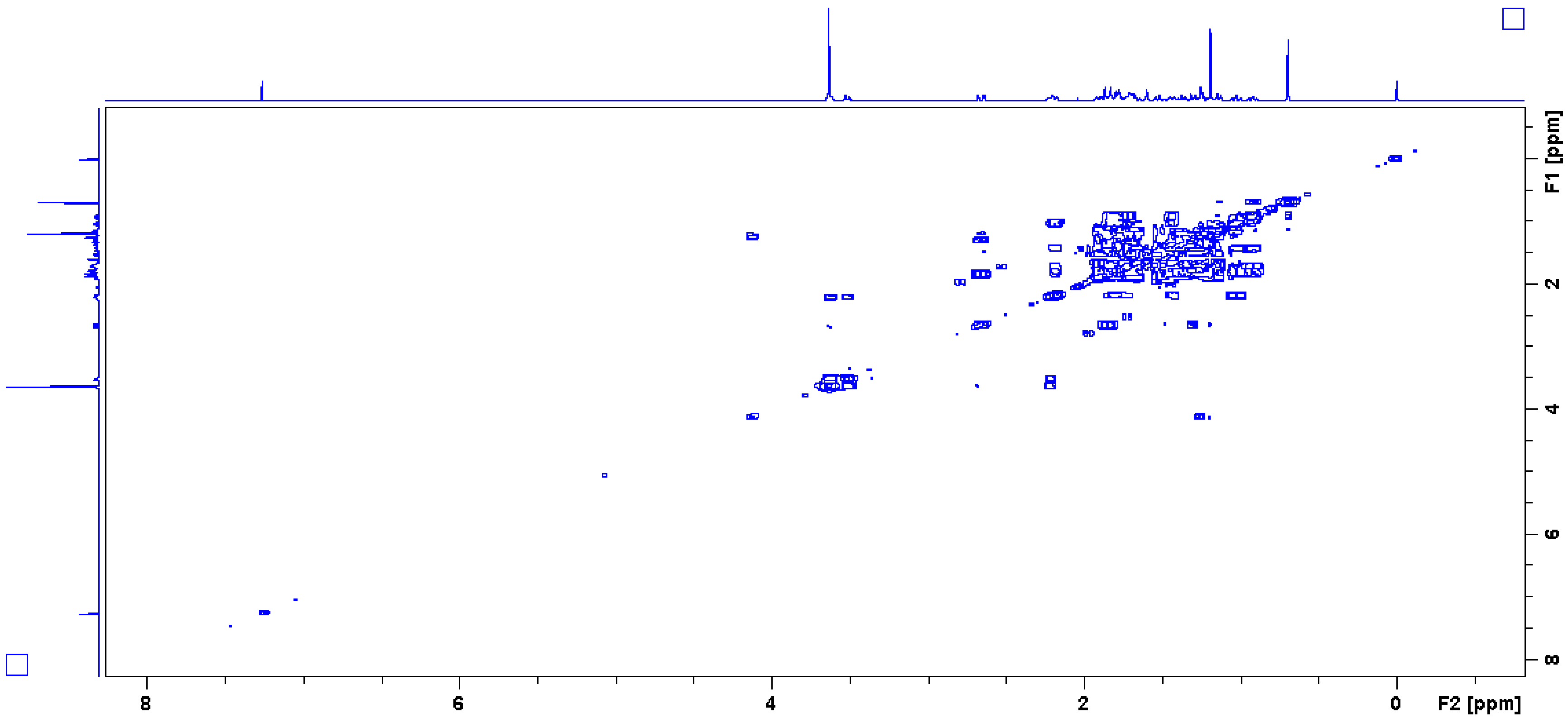

NOESY of compound (4*R*,4a*S*,6a*R*,9*S*,11a*R*,11b*S*)-methyl 9-(hydroxymethyl)-4,11b-dimethyl-8-oxotetradecahydro-6a,9-methanocyclohepta[a]naphthalene-4-carboxylate **(4)**

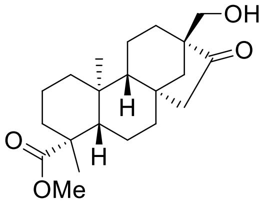

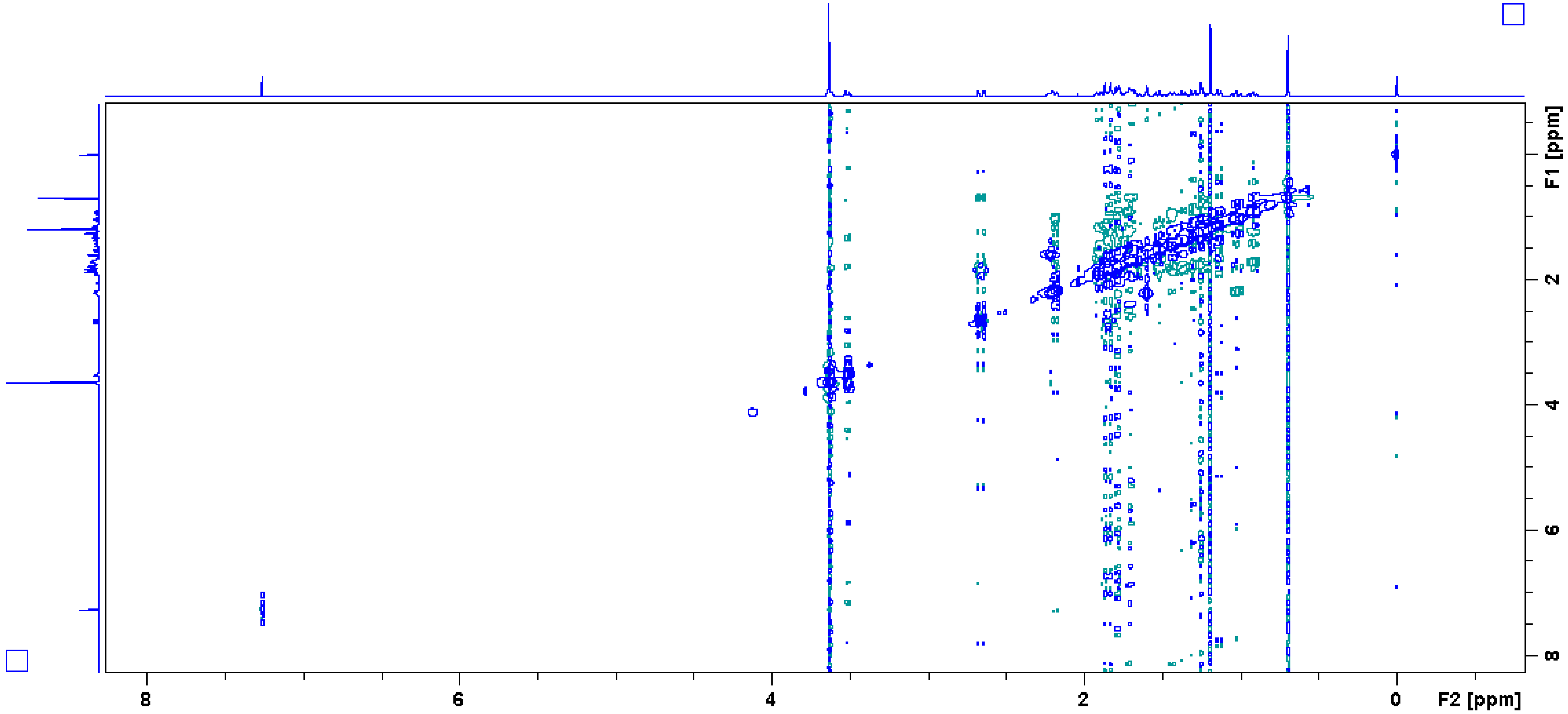

HSQC of compound (4*R*,4a*S*,6a*R*,9*S*,11a*R*,11b*S*)-methyl 9-(hydroxymethyl)-4,11b-dimethyl-8-oxotetradecahydro-6a,9-methanocyclohepta[a]naphthalene-4-carboxylate **(4)**

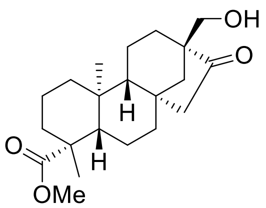

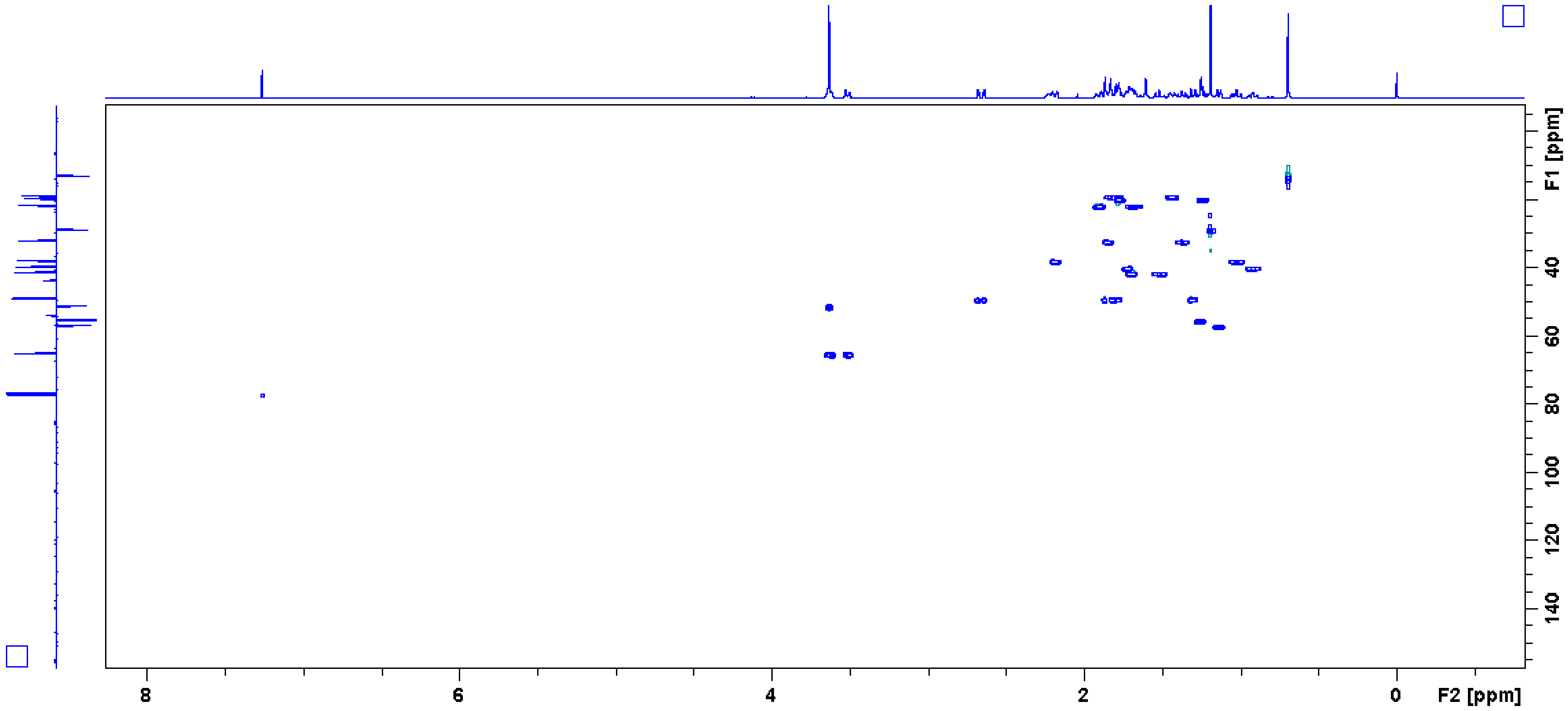

HMBC of compound (4*R*,4a*S*,6a*R*,9*S*,11a*R*,11b*S*)-methyl 9-(hydroxymethyl)-4,11b-dimethyl-8-oxotetradecahydro-6a,9-methanocyclohepta[a]naphthalene-4-carboxylate **(4)**

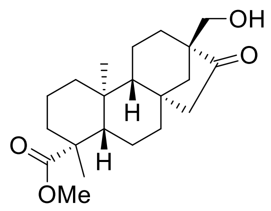

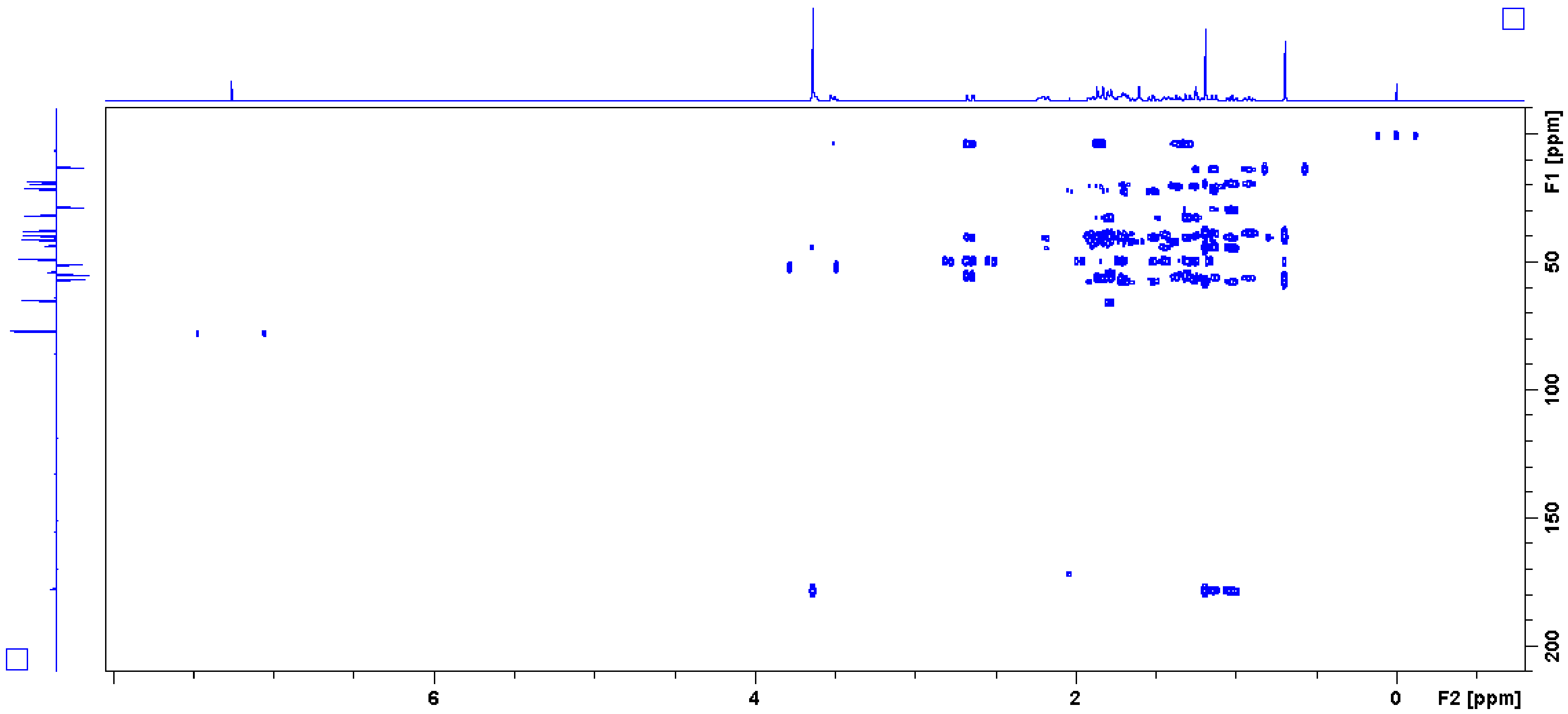

^{1}H-NMR of compound (4*R*,4a*S*,6a*R*,9*R*,11a*R*,11b*S*,E)-methyl 8-(hydroxyimino)-9-(hydroxymethyl)-4,11b-dimethyltetradecahydro-6a,9-methanocyclohepta[a]naphthalene-4-carboxylate **(5)**

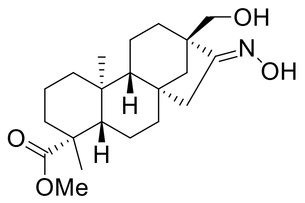

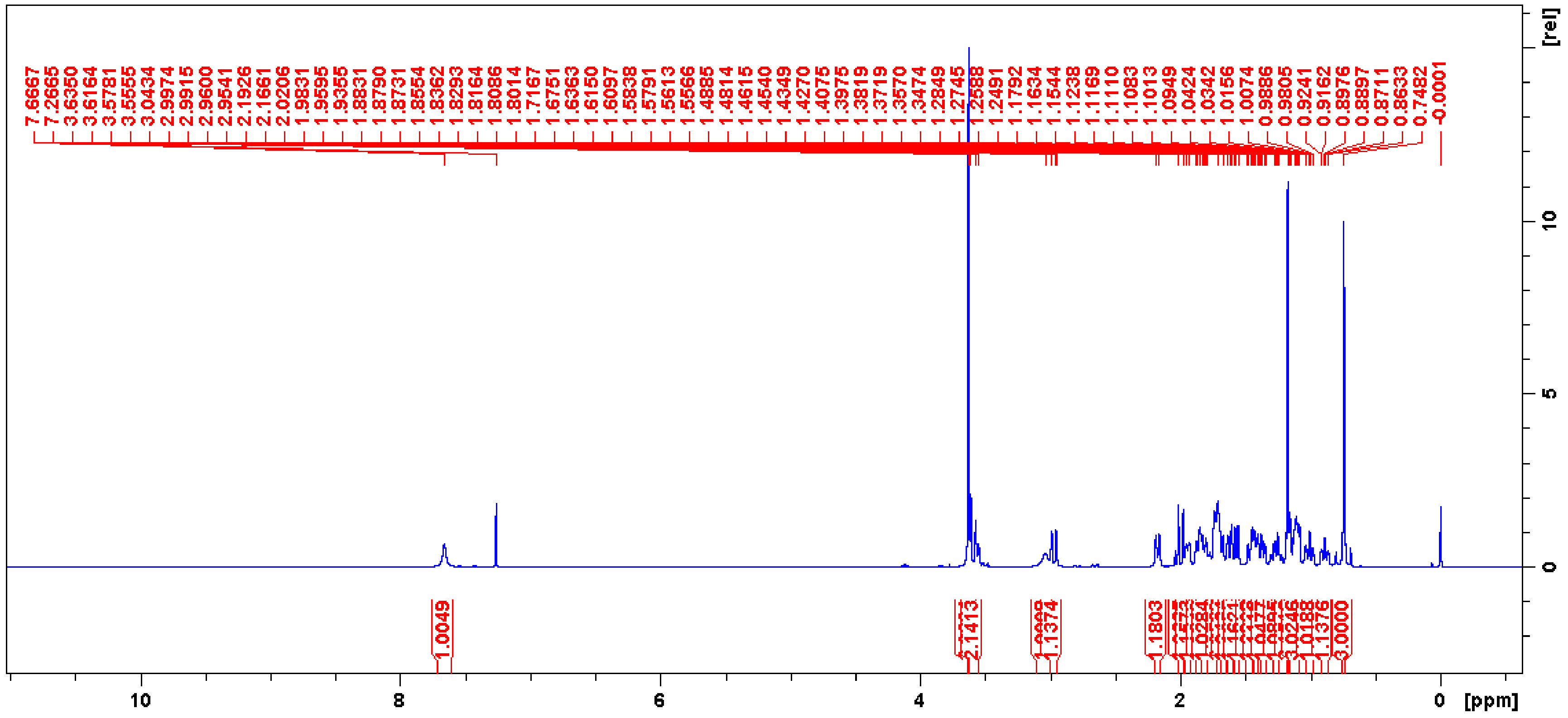

^{13}C-NMR of compound (4*R*,4a*S*,6a*R*,9*R*,11a*R*,11b*S*,E)-methyl 8-(hydroxyimino)-9-(hydroxymethyl)-4,11b-dimethyltetradecahydro-6a,9-methanocyclohepta[a]naphthalene-4-carboxylate **(5)**

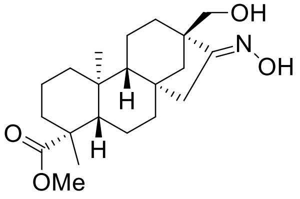

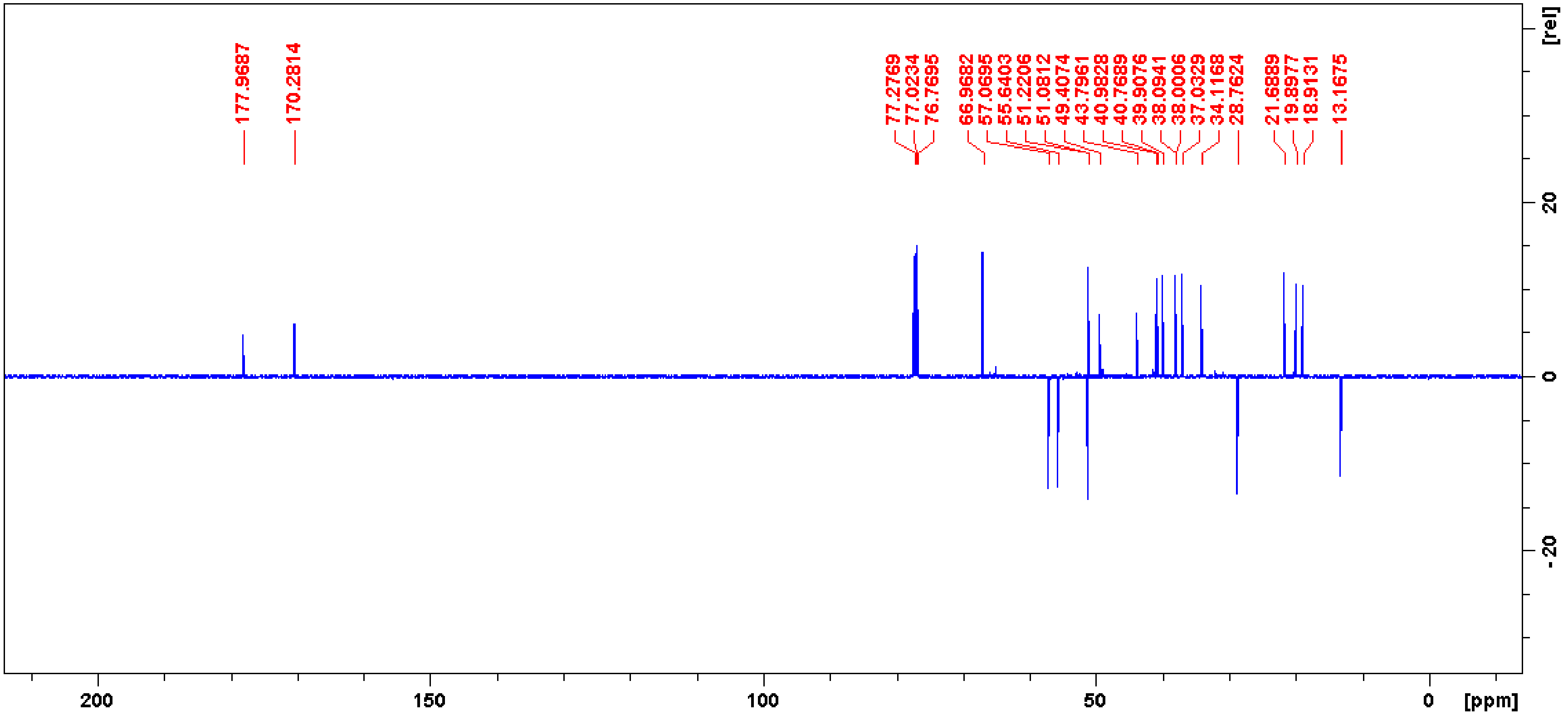

COSY of compound (4*R*,4a*S*,6a*R*,9*R*,11a*R*,11b*S*,E)-methyl 8-(hydroxyimino)-9-(hydroxymethyl)-4,11b-dimethyltetradecahydro-6a,9-methanocyclohepta[a]naphthalene-4-carboxylate **(5)**

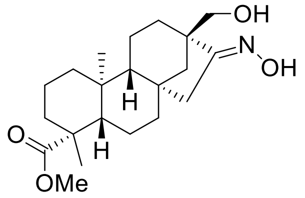

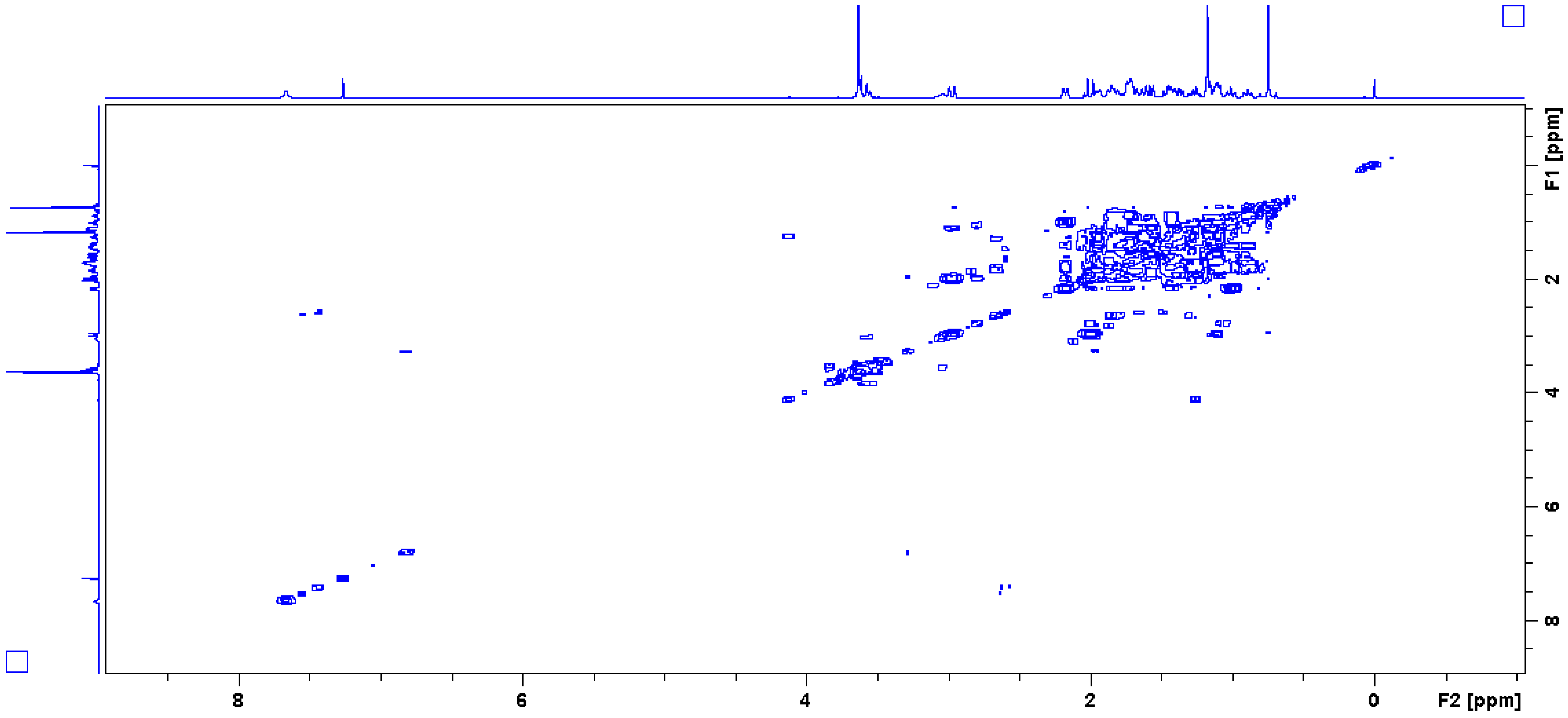

NOESY of compound (4*R*,4a*S*,6a*R*,9*R*,11a*R*,11b*S*,E)-methyl 8-(hydroxyimino)-9-(hydroxymethyl)-4,11b-dimethyltetradecahydro-6a,9-methanocyclohepta[a]naphthalene-4-carboxylate **(5)**

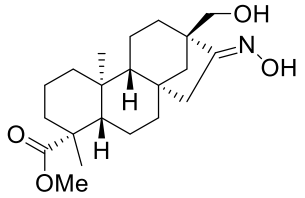

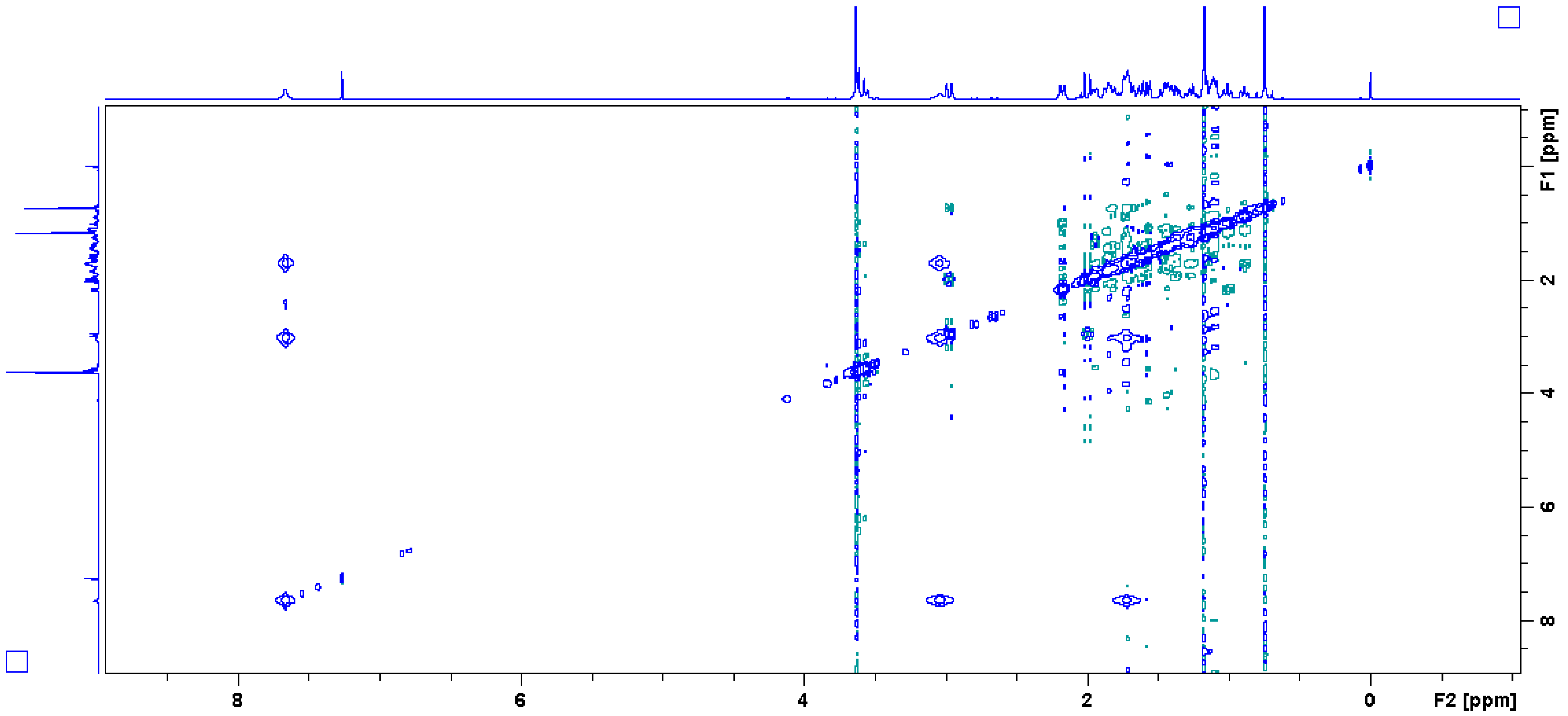

HSQC of compound (4*R*,4a*S*,6a*R*,9*R*,11a*R*,11b*S*,E)-methyl 8-(hydroxyimino)-9-(hydroxymethyl)-4,11b-dimethyltetradecahydro-6a,9-methanocyclohepta[a]naphthalene-4-carboxylate **(5)**

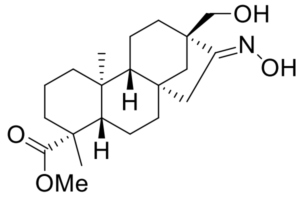

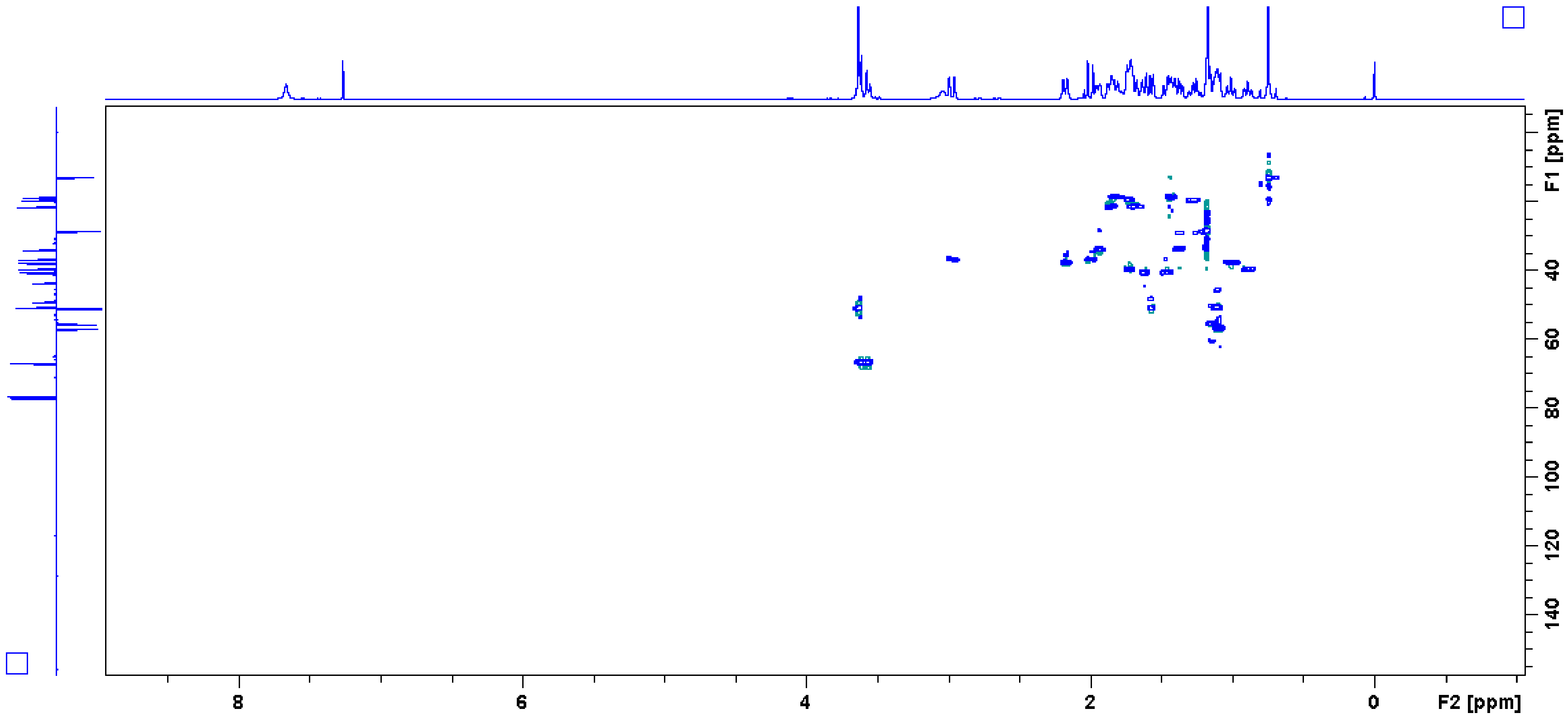

HMBC of compound (4*R*,4a*S*,6a*R*,9*R*,11a*R*,11b*S*,E)-methyl 8-(hydroxyimino)-9-(hydroxymethyl)-4,11b-dimethyltetradecahydro-6a,9-methanocyclohepta[a]naphthalene-4-carboxylate **(5)**

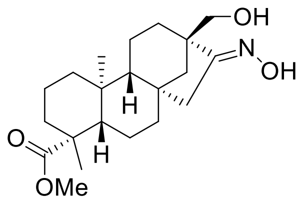

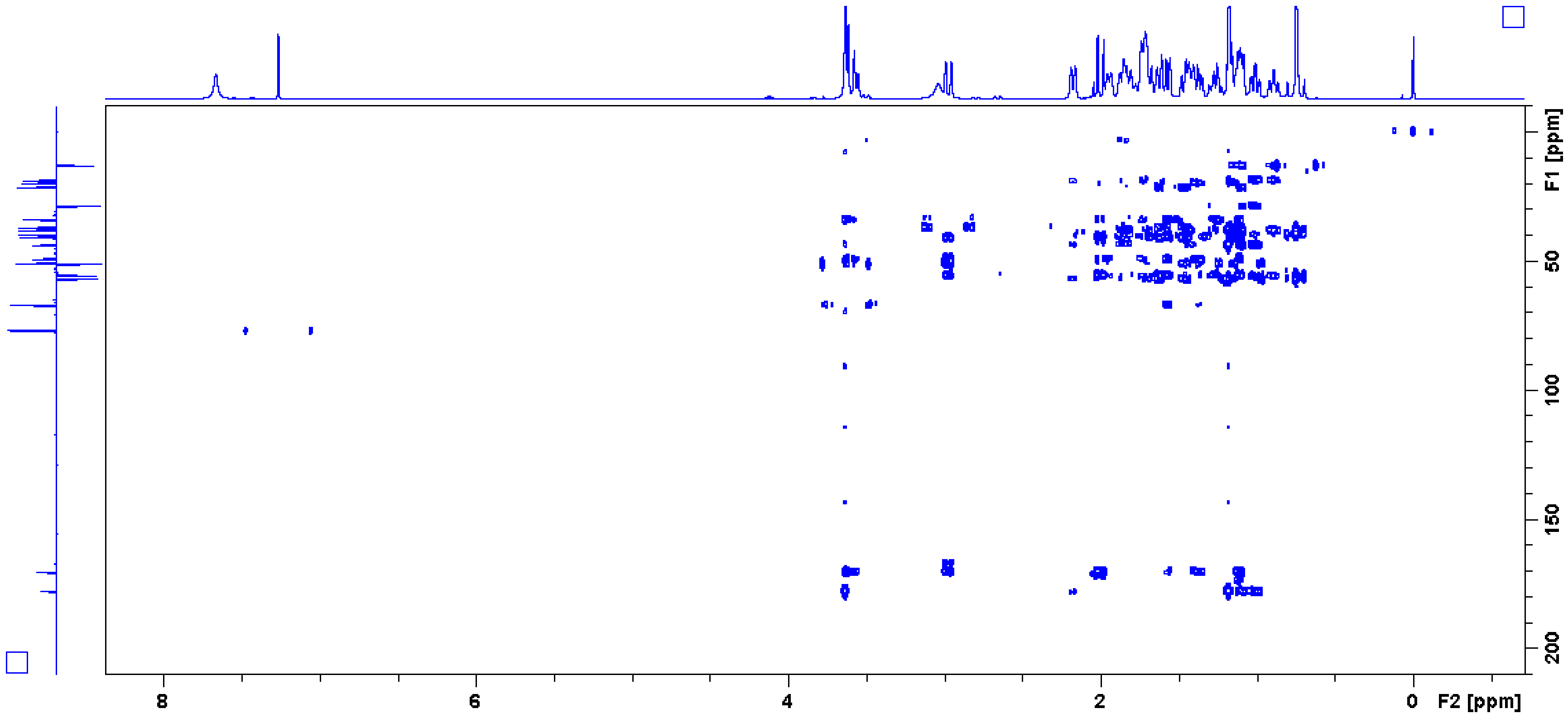

^{1}H-NMR of compound (4*R*,4a*S*,6a*R*,8*R*,9*R*,11a*R*,11b*S*)-methyl 8-amino-9-(hydroxymethyl)-4,11b-dimethyltetradecahydro-6a,9-methanocyclohepta[a]naphthalene-4-carboxylate **(6)**

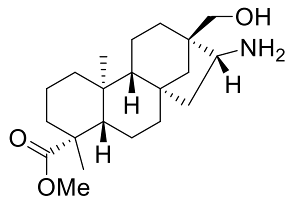

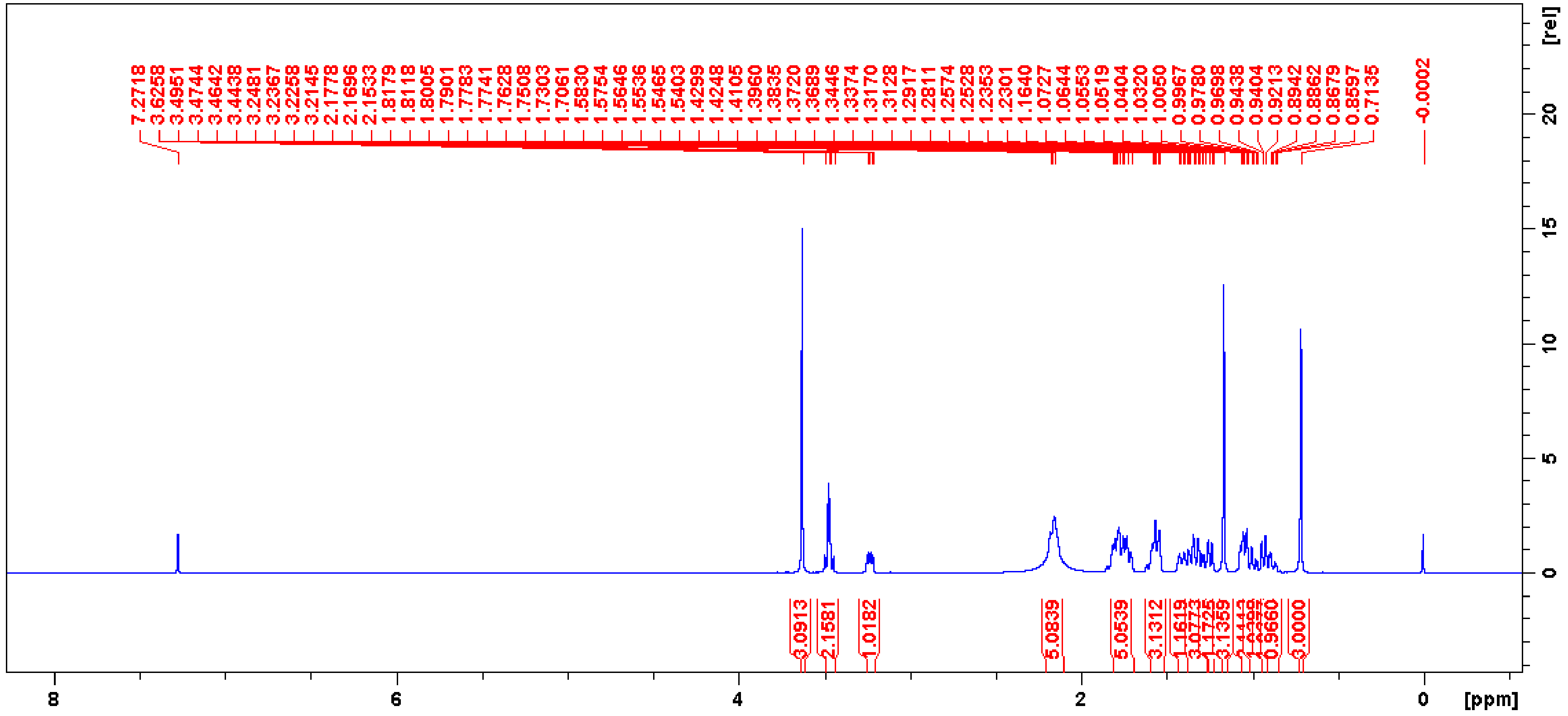

^{13}C-NMR of compound (4*R*,4a*S*,6a*R*,8*R*,9*R*,11a*R*,11b*S*)-methyl 8-amino-9-(hydroxymethyl)-4,11b-dimethyltetradecahydro-6a,9-methanocyclohepta[a]naphthalene-4-carboxylate **(6)**

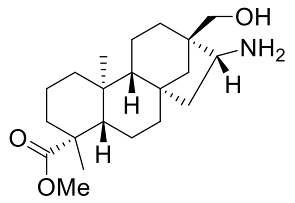

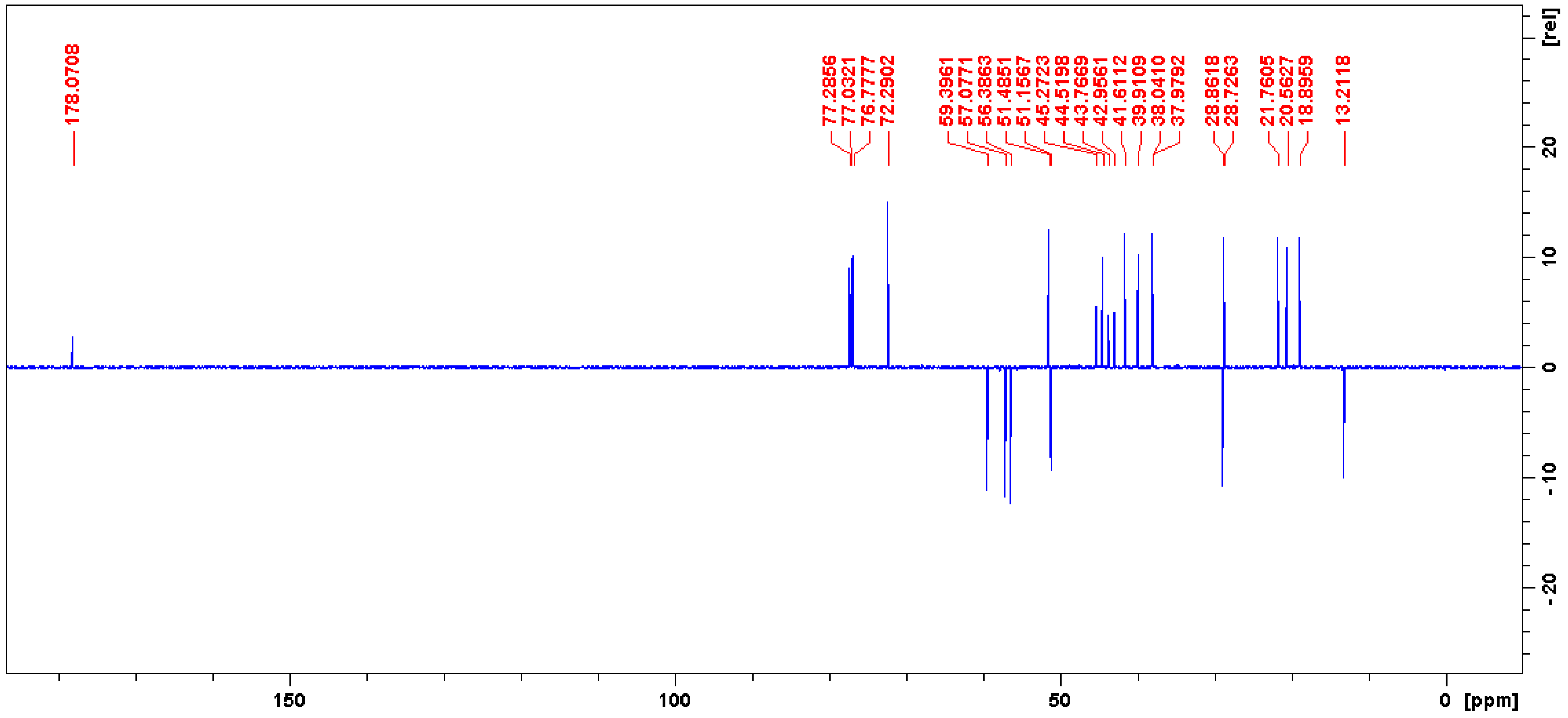

COSY of compound (4*R*,4a*S*,6a*R*,8*R*,9*R*,11a*R*,11b*S*)-methyl 8-amino-9-(hydroxymethyl)-4,11b-dimethyltetradecahydro-6a,9-methanocyclohepta[a]naphthalene-4-carboxylate **(6)**

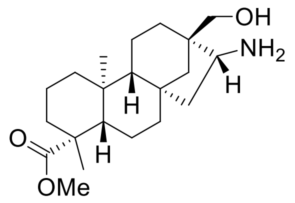

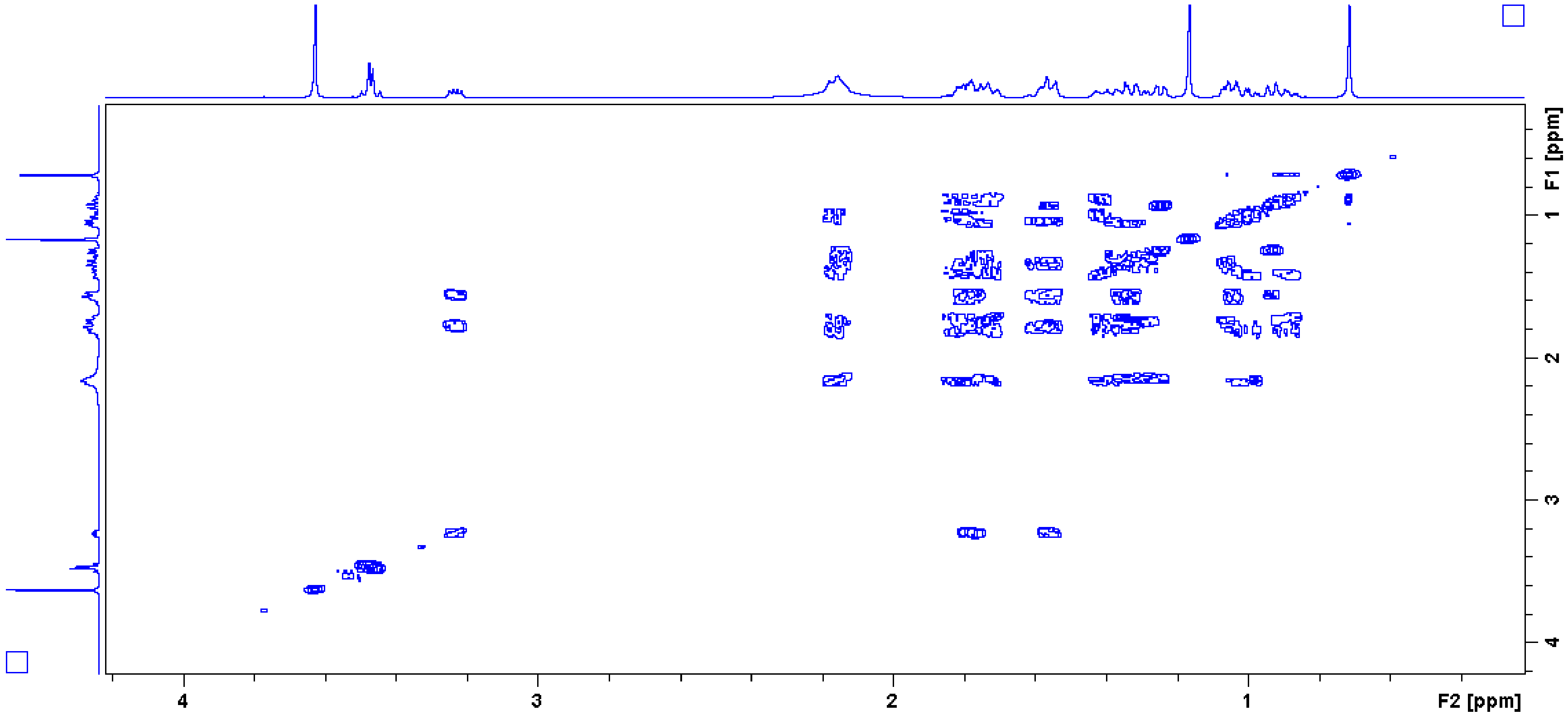

NOESY of compound (4*R*,4a*S*,6a*R*,8*R*,9*R*,11a*R*,11b*S*)-methyl 8-amino-9-(hydroxymethyl)-4,11b-dimethyltetradecahydro-6a,9-methanocyclohepta[a]naphthalene-4-carboxylate **(6)**

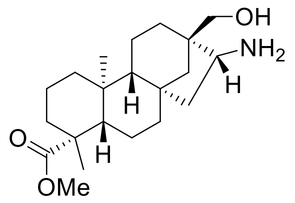

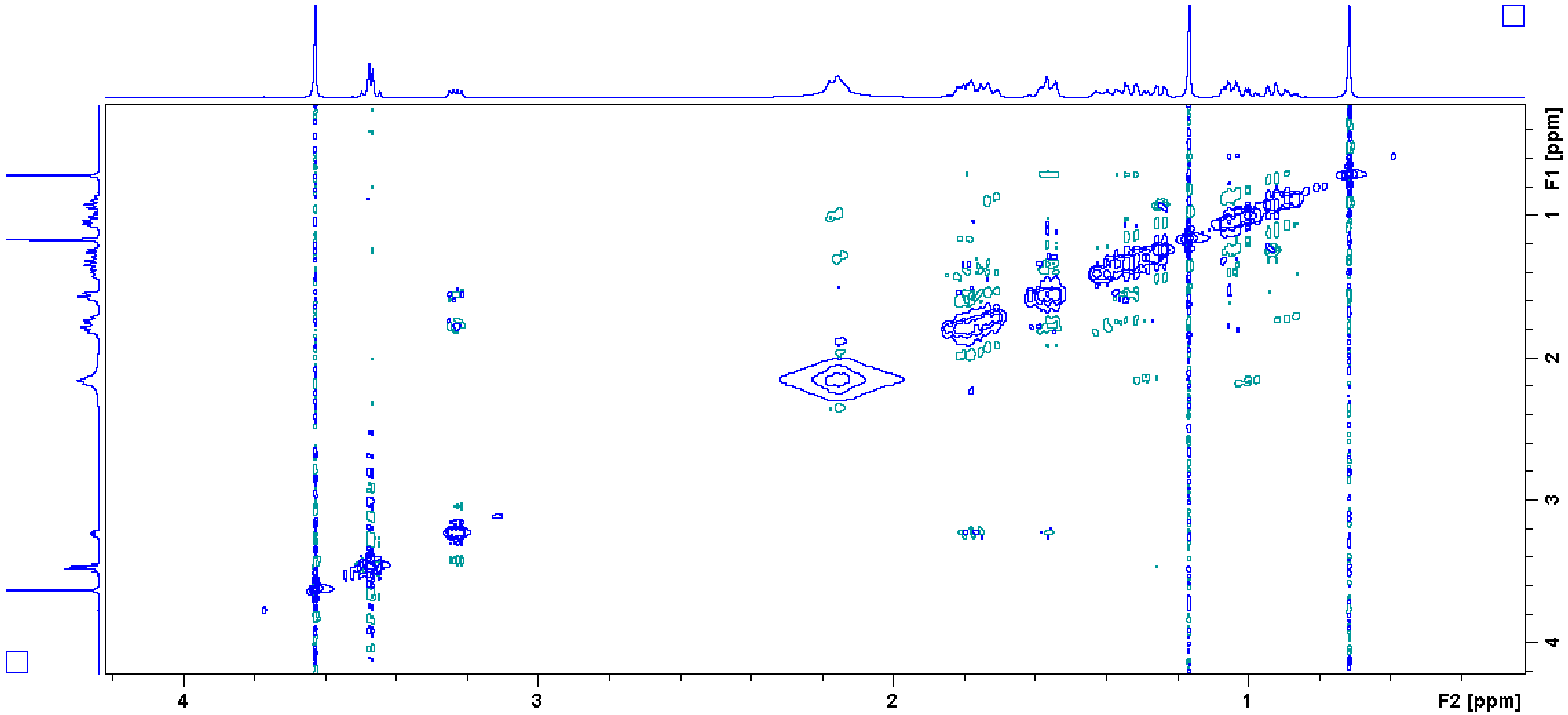

HSQC of compound (4*R*,4a*S*,6a*R*,8*R*,9*R*,11a*R*,11b*S*)-methyl 8-amino-9-(hydroxymethyl)-4,11b-dimethyltetradecahydro-6a,9-methanocyclohepta[a]naphthalene-4-carboxylate **(6)**

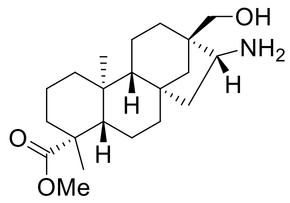

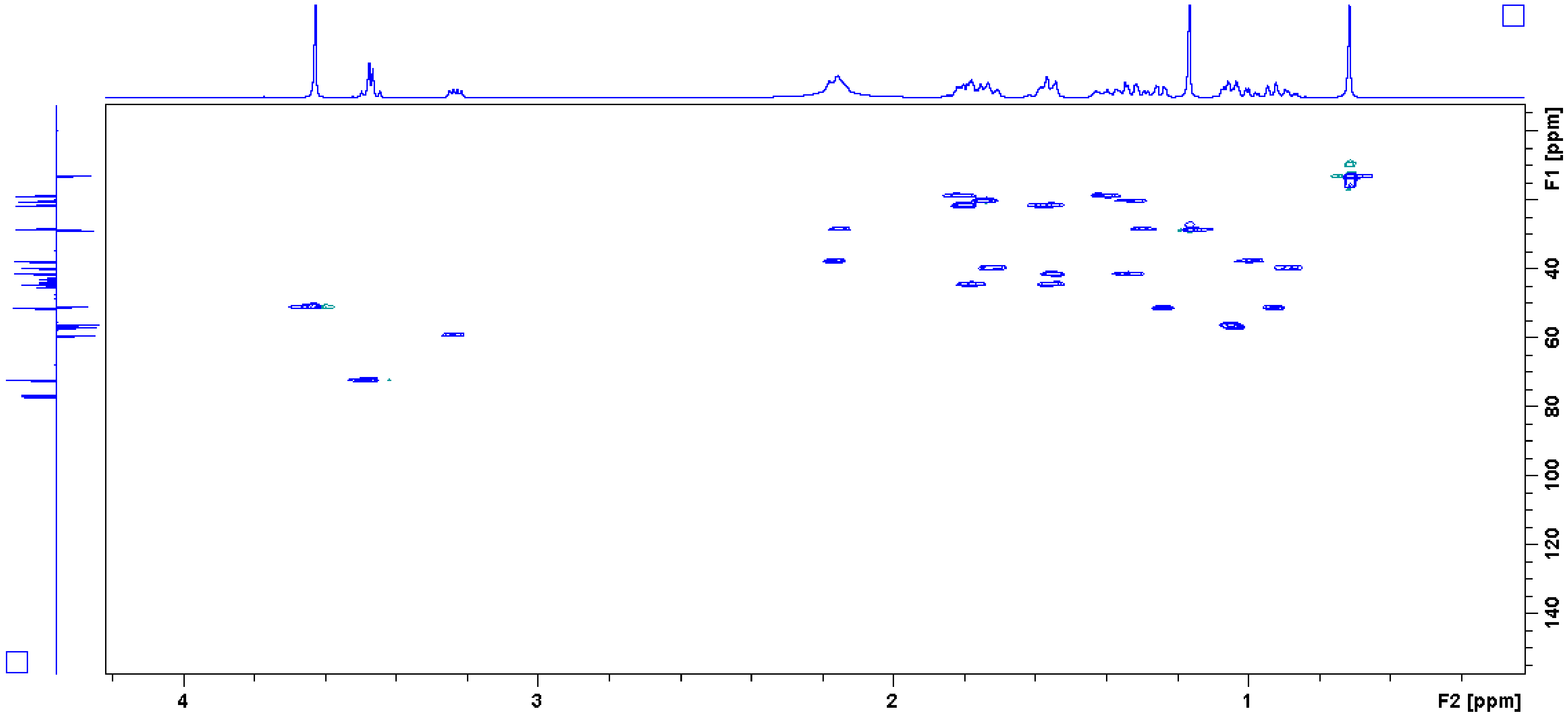

HMBC of compound (4*R*,4a*S*,6a*R*,8*R*,9*R*,11a*R*,11b*S*)-methyl 8-amino-9-(hydroxymethyl)-4,11b-dimethyltetradecahydro-6a,9-methanocyclohepta[a]naphthalene-4-carboxylate **(6)**

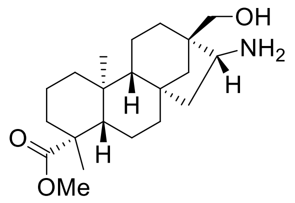

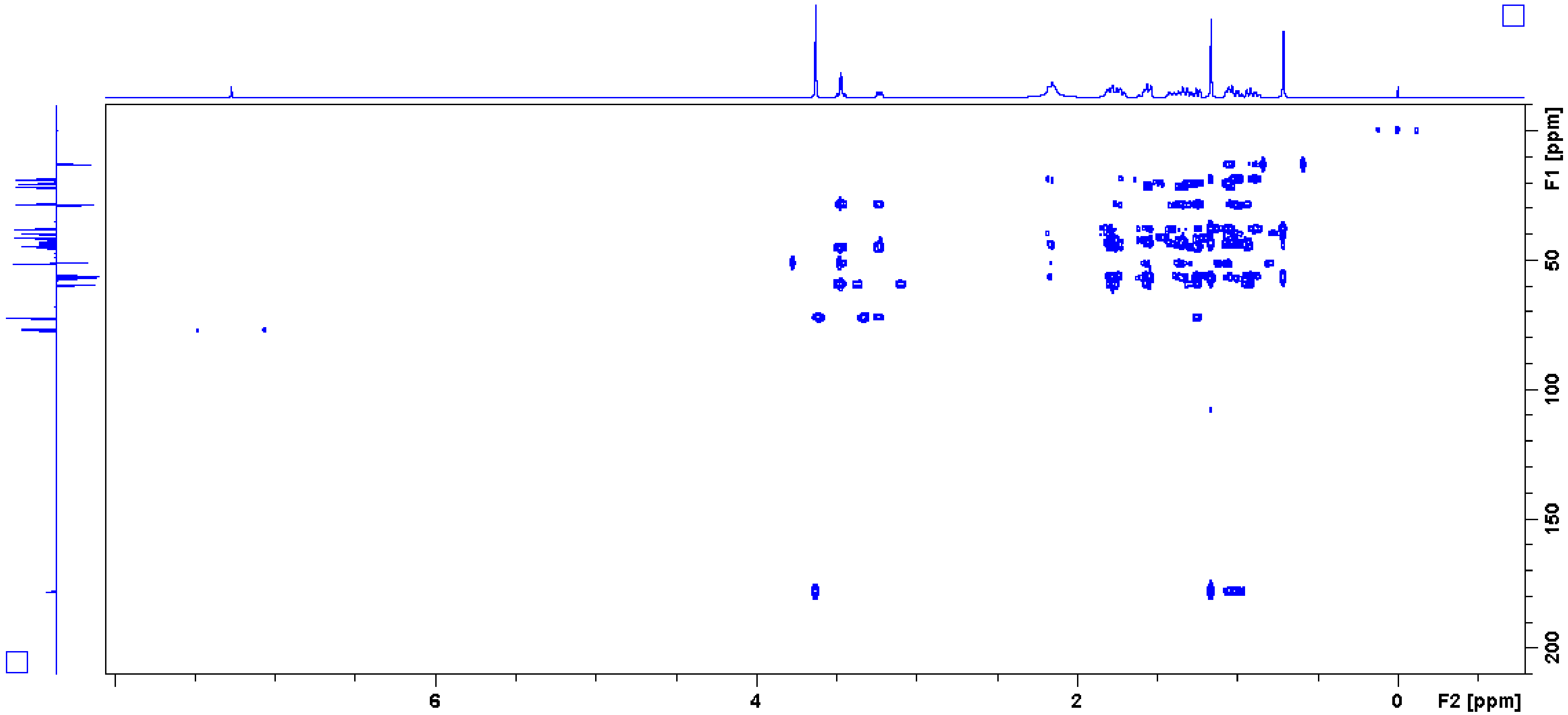

^{1}H-NMR of compound (4*R*,4a*S*,6a*R*,8*S*,9*R*,11a*R*,11b*S*)-methyl 8-amino-9-(hydroxymethyl)-4,11b-dimethyltetradecahydro-6a,9-methanocyclohepta[a]naphthalene-4-carboxylate **(7)**

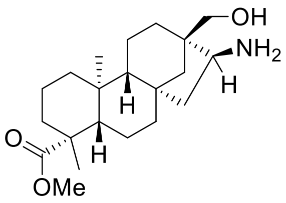

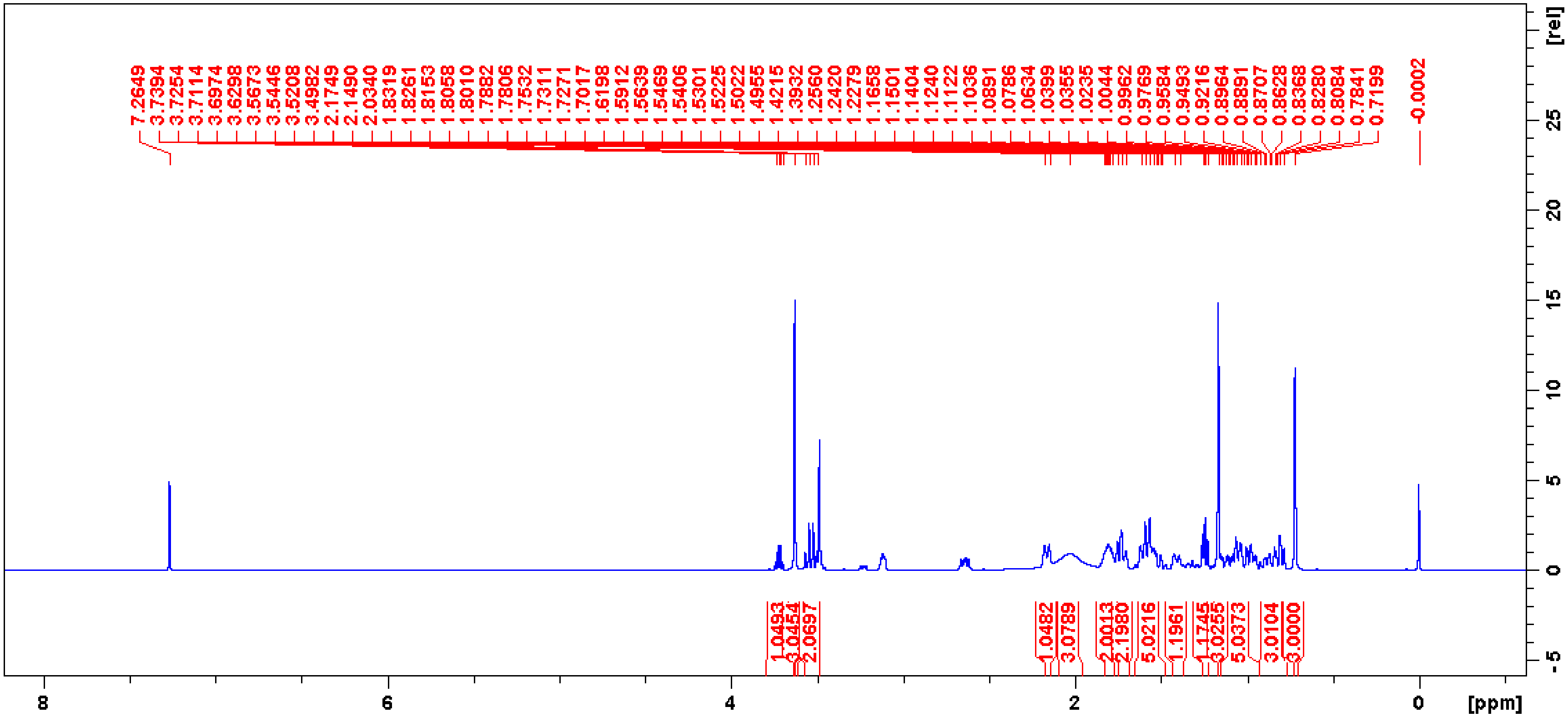

^{13}C-NMR of compound (4*R*,4a*S*,6a*R*,8*S*,9*R*,11a*R*,11b*S*)-methyl 8-amino-9-(hydroxymethyl)-4,11b-dimethyltetradecahydro-6a,9-methanocyclohepta[a]naphthalene-4-carboxylate **(7)**

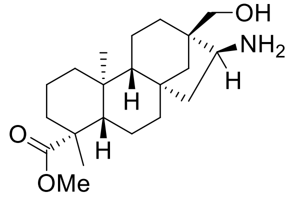

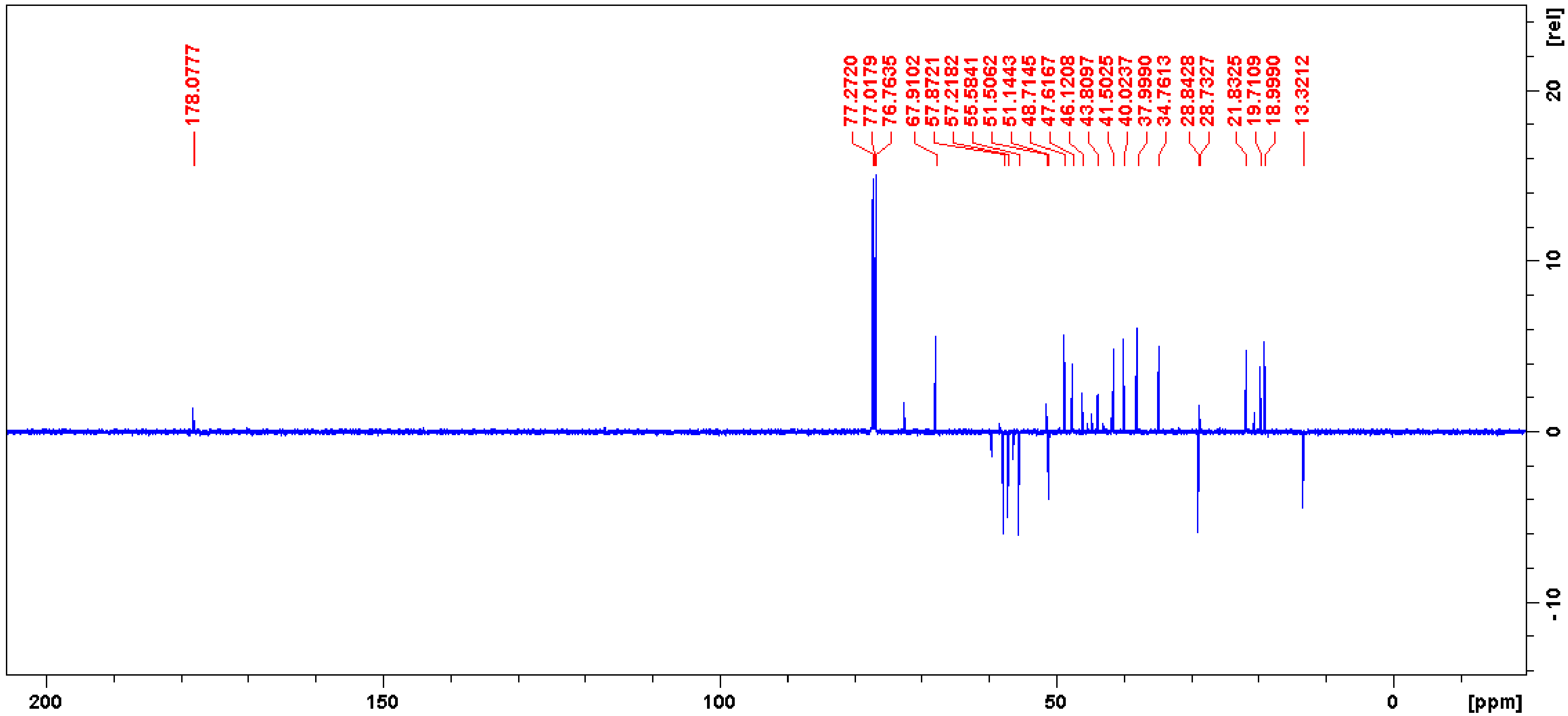

COSY of compound (4*R*,4a*S*,6a*R*,8*S*,9*R*,11a*R*,11b*S*)-methyl 8-amino-9-(hydroxymethyl)-4,11b-dimethyltetradecahydro-6a,9-methanocyclohepta[a]naphthalene-4-carboxylate **(7)**

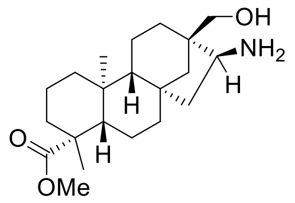

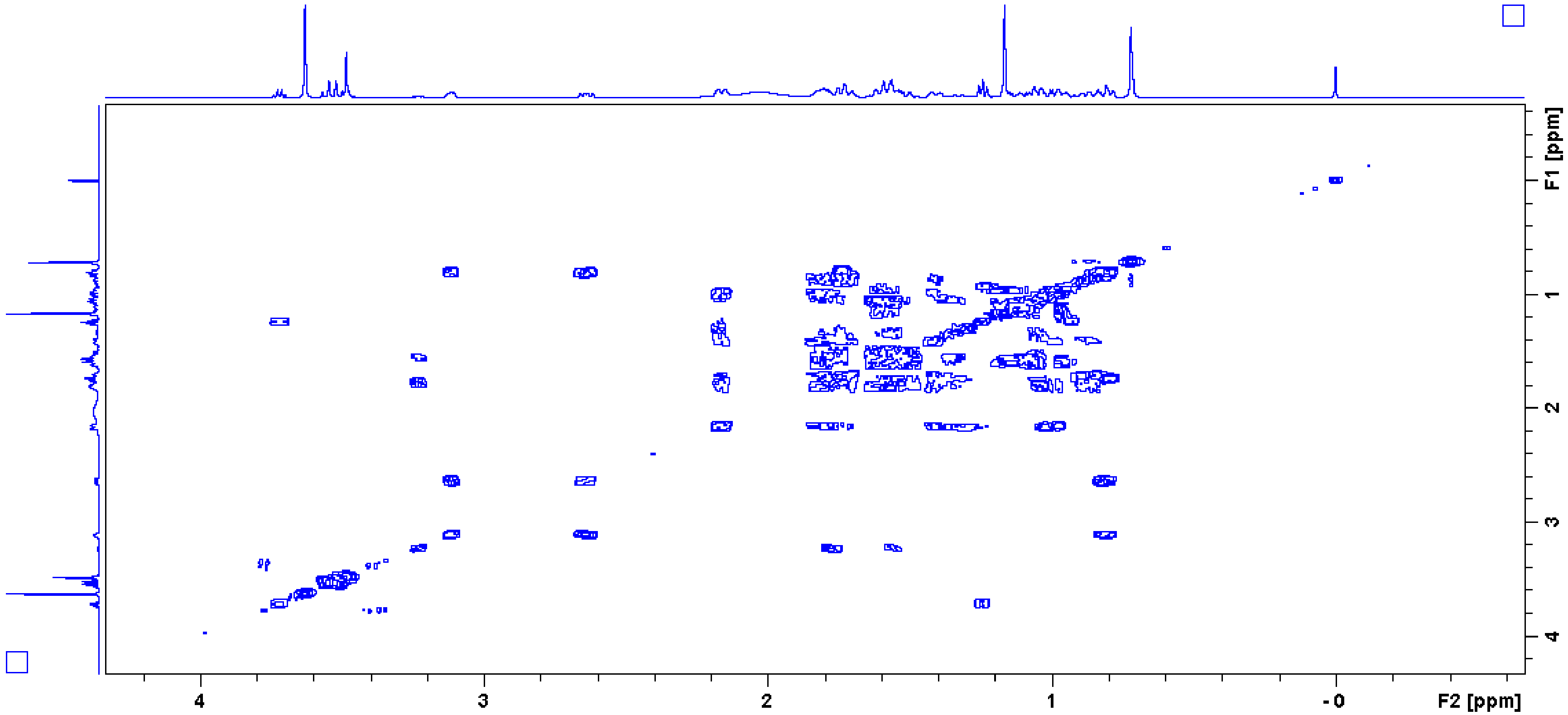

NOESY of compound (4*R*,4a*S*,6a*R*,8*S*,9*R*,11a*R*,11b*S*)-methyl 8-amino-9-(hydroxymethyl)-4,11b-dimethyltetradecahydro-6a,9-methanocyclohepta[a]naphthalene-4-carboxylate **(7)**

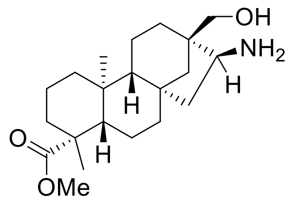

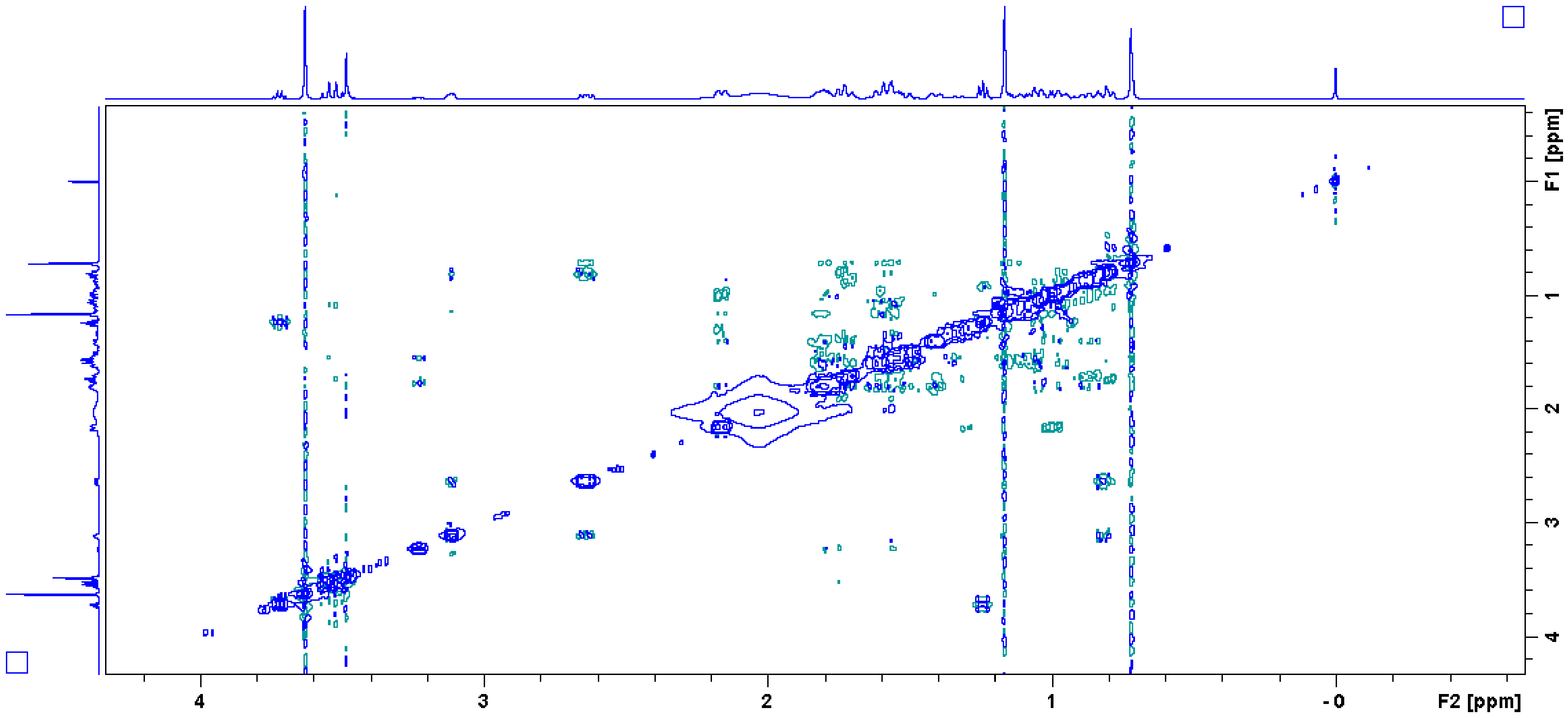

HSQC of compound (4*R*,4a*S*,6a*R*,8*S*,9*R*,11a*R*,11b*S*)-methyl 8-amino-9-(hydroxymethyl)-4,11b-dimethyltetradecahydro-6a,9-methanocyclohepta[a]naphthalene-4-carboxylate **(7)**

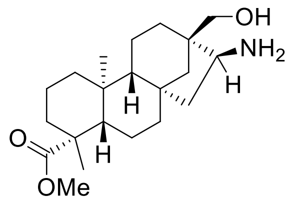

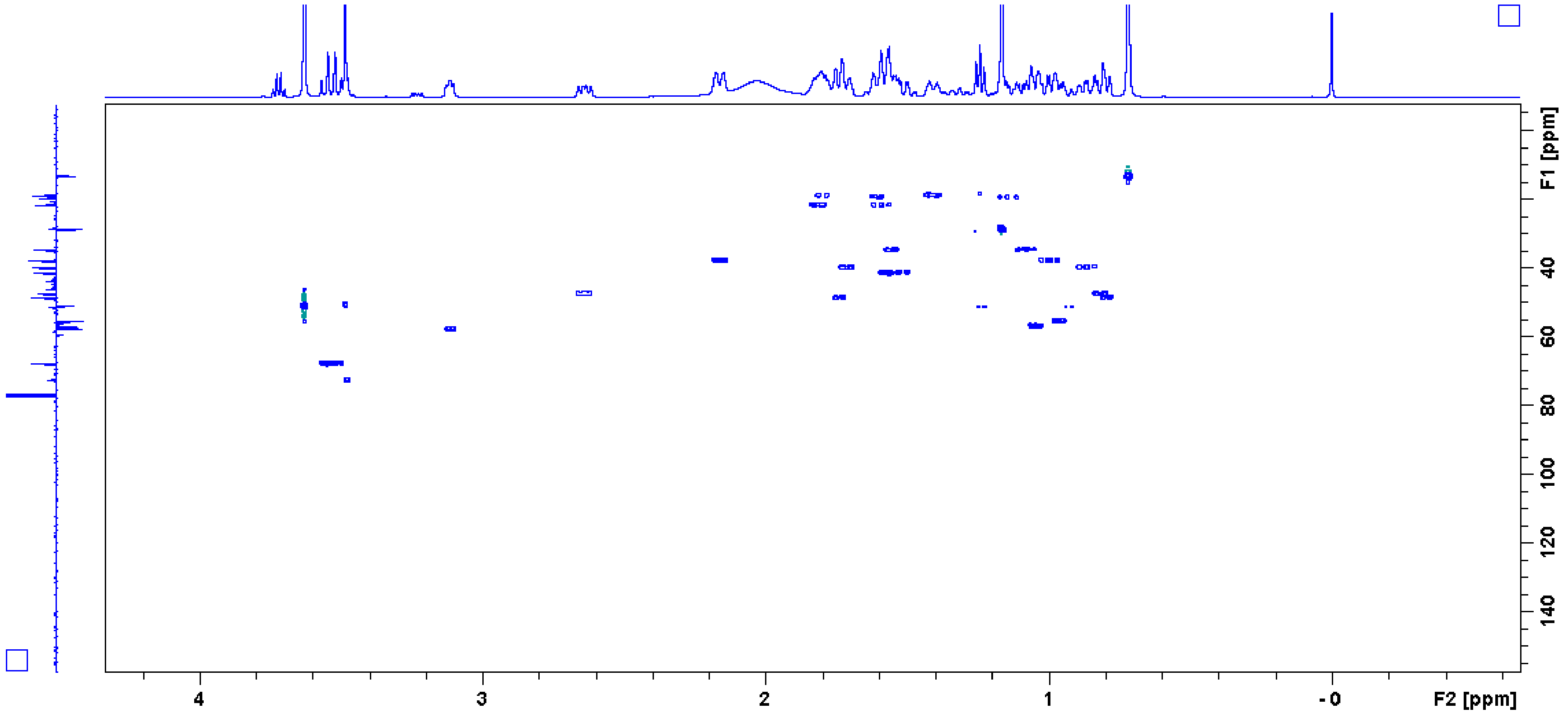

HMBC of compound (4*R*,4a*S*,6a*R*,8*S*,9*R*,11a*R*,11b*S*)-methyl 8-amino-9-(hydroxymethyl)-4,11b-dimethyltetradecahydro-6a,9-methanocyclohepta[a]naphthalene-4-carboxylate **(7)**

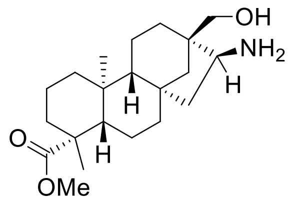

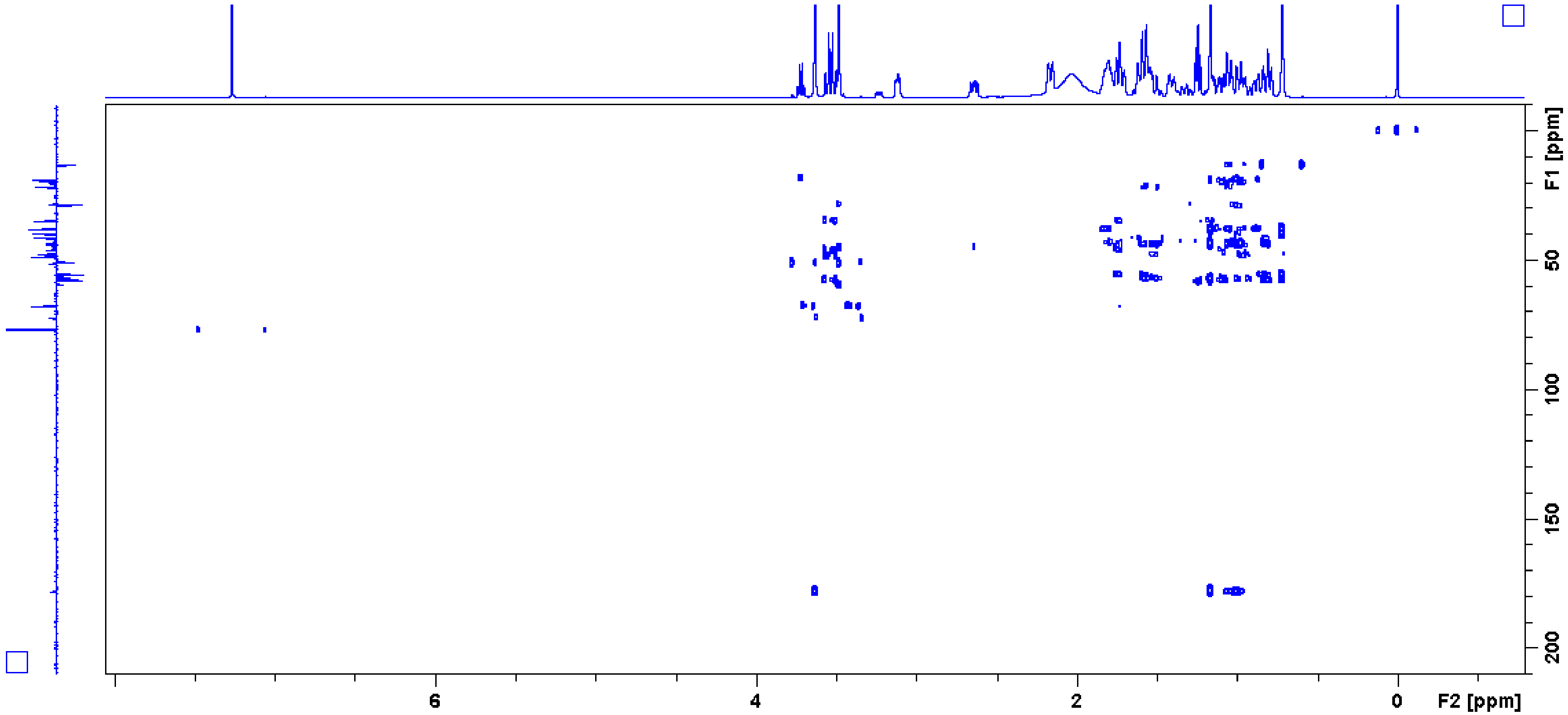

^{1}H-NMR of compound (4*R*,4a*S*,6a*R*,8*R*,9*R*,11a*R*,11b*S*)-methyl 8-(benzylamino)-9-(hydroxymethyl)-4,11b-dimethyltetradecahydro-6a,9-methanocyclohepta[a]naphthalene-4-carboxylate **(8)**

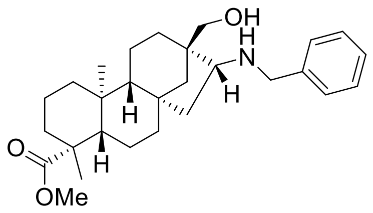

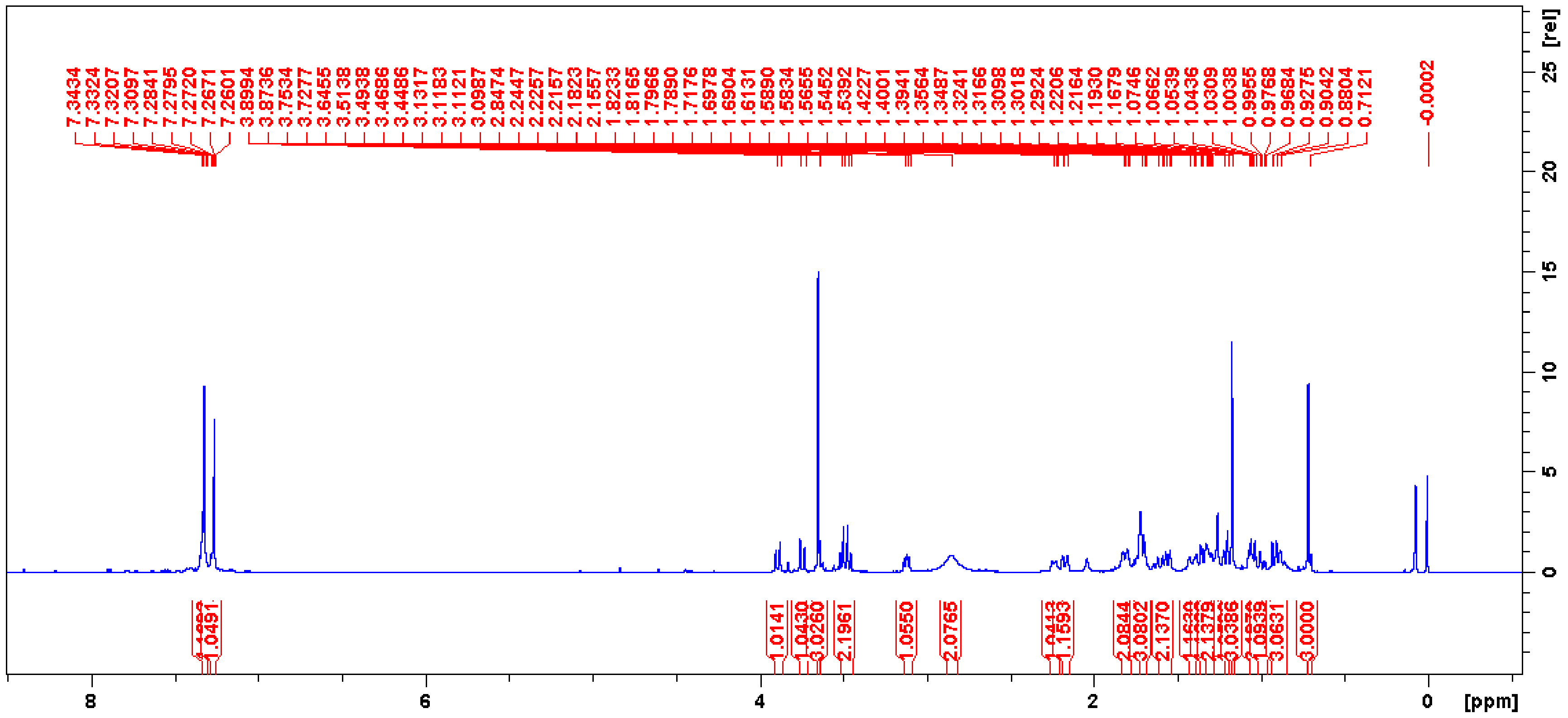

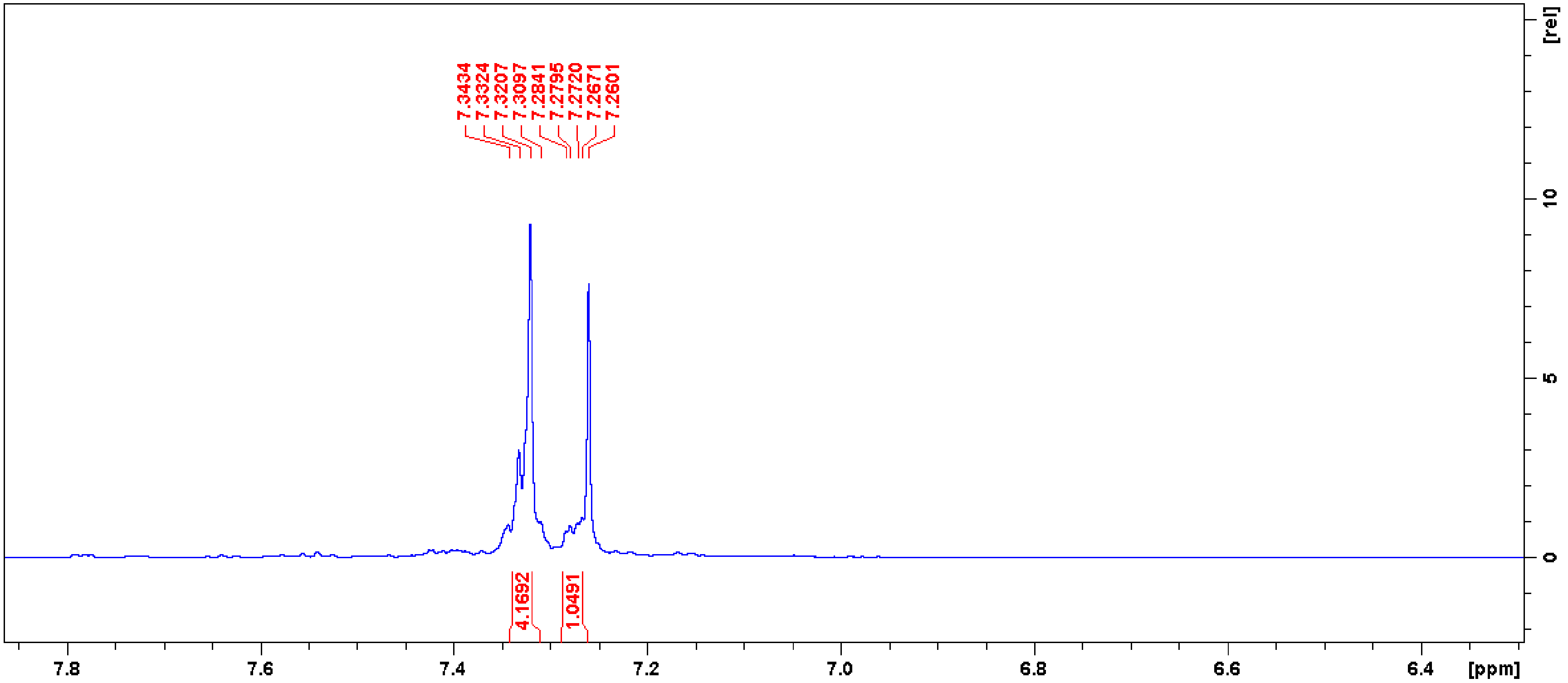
7.3434
7.3324
7.3207
7.3097
7.2841
7.2795
7.2720
7.2671
7.2601
4.1692
1.0491
7.8
7.6
7.4
7.2
7.0
6.8
6.6
6.4
[ppm]
[rel]
10
5
0

^{13}C-NMR of compound (4*R*,4a*S*,6a*R*,8*R*,9*R*,11a*R*,11b*S*)-methyl 8-(benzylamino)-9-(hydroxymethyl)-4,11b-dimethyltetradecahydro-6a,9-methanocyclohepta[a]naphthalene-4-carboxylate **(8)**

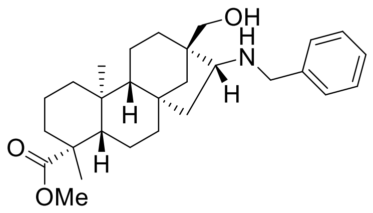

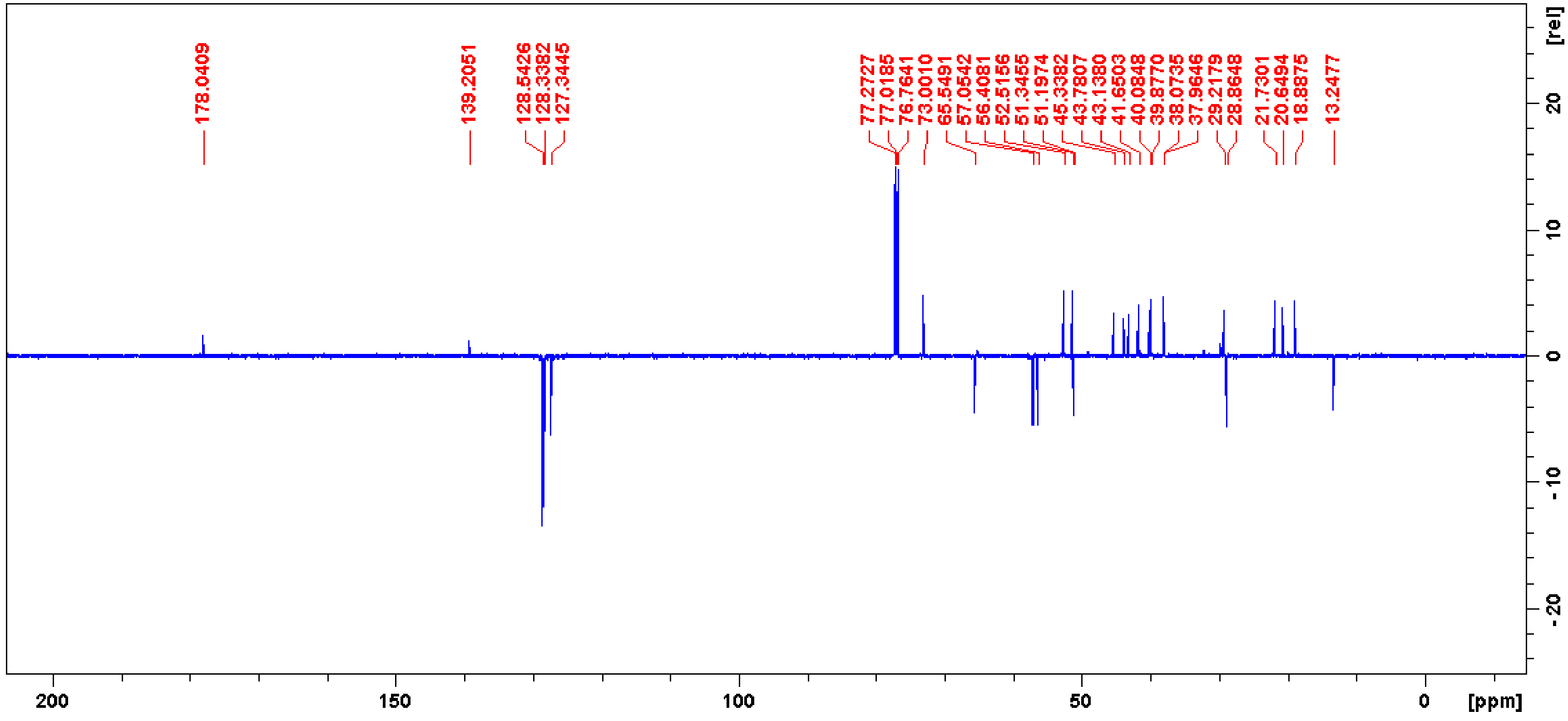

COSY of compound (4*R*,4a*S*,6a*R*,8*R*,9*R*,11a*R*,11b*S*)-methyl 8-(benzylamino)-9-(hydroxymethyl)-4,11b-dimethyltetradecahydro-6a,9-methanocyclohepta[a]naphthalene-4-carboxylate **(8)**

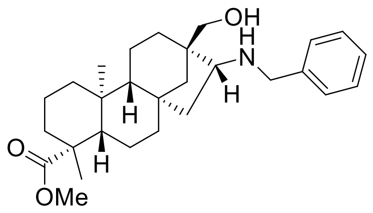

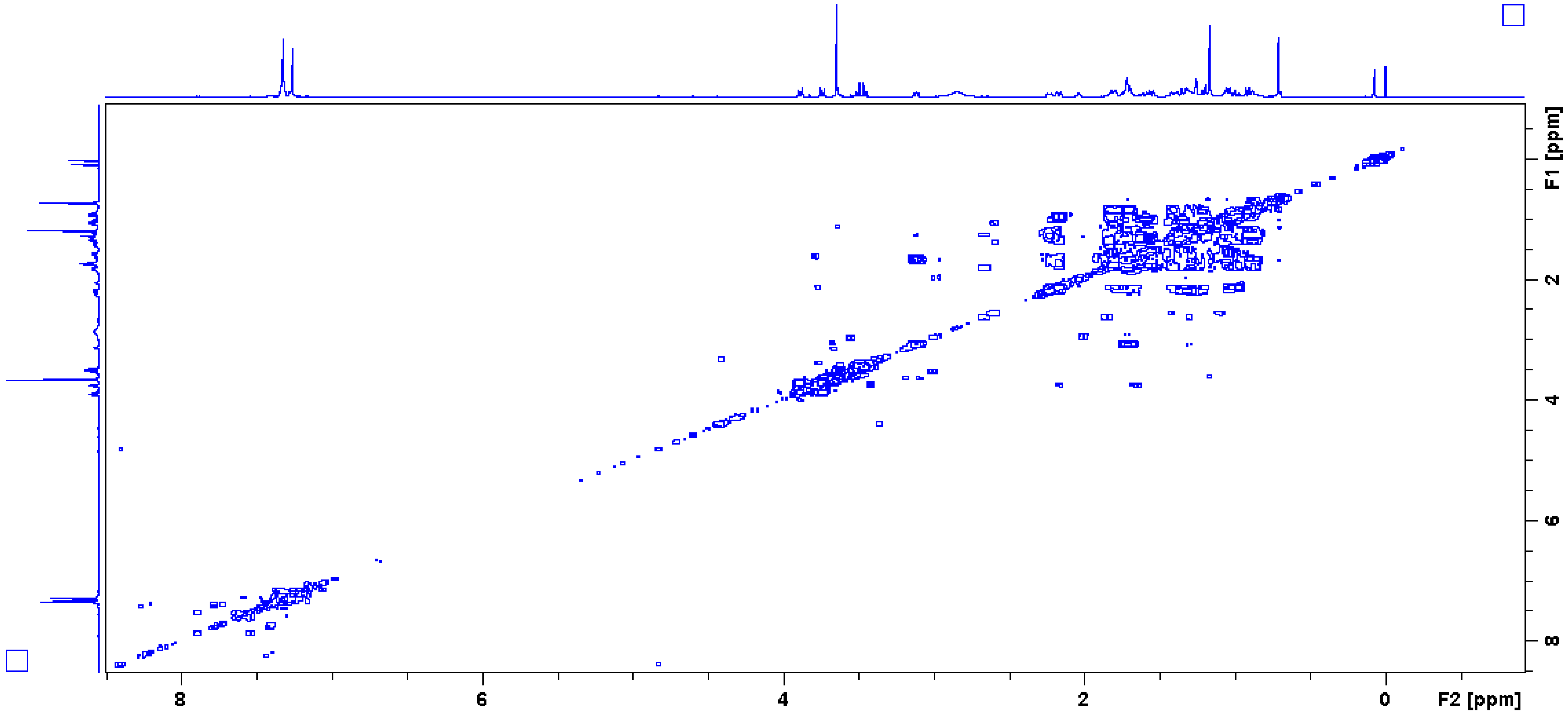

NOESY of compound (4*R*,4a*S*,6a*R*,8*R*,9*R*,11a*R*,11b*S*)-methyl 8-(benzylamino)-9-(hydroxymethyl)-4,11b-dimethyltetradecahydro-6a,9-methanocyclohepta[a]naphthalene-4-carboxylate **(8)**

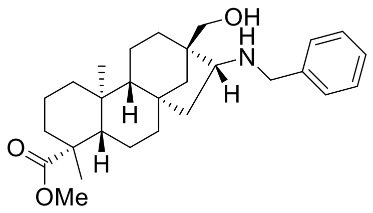

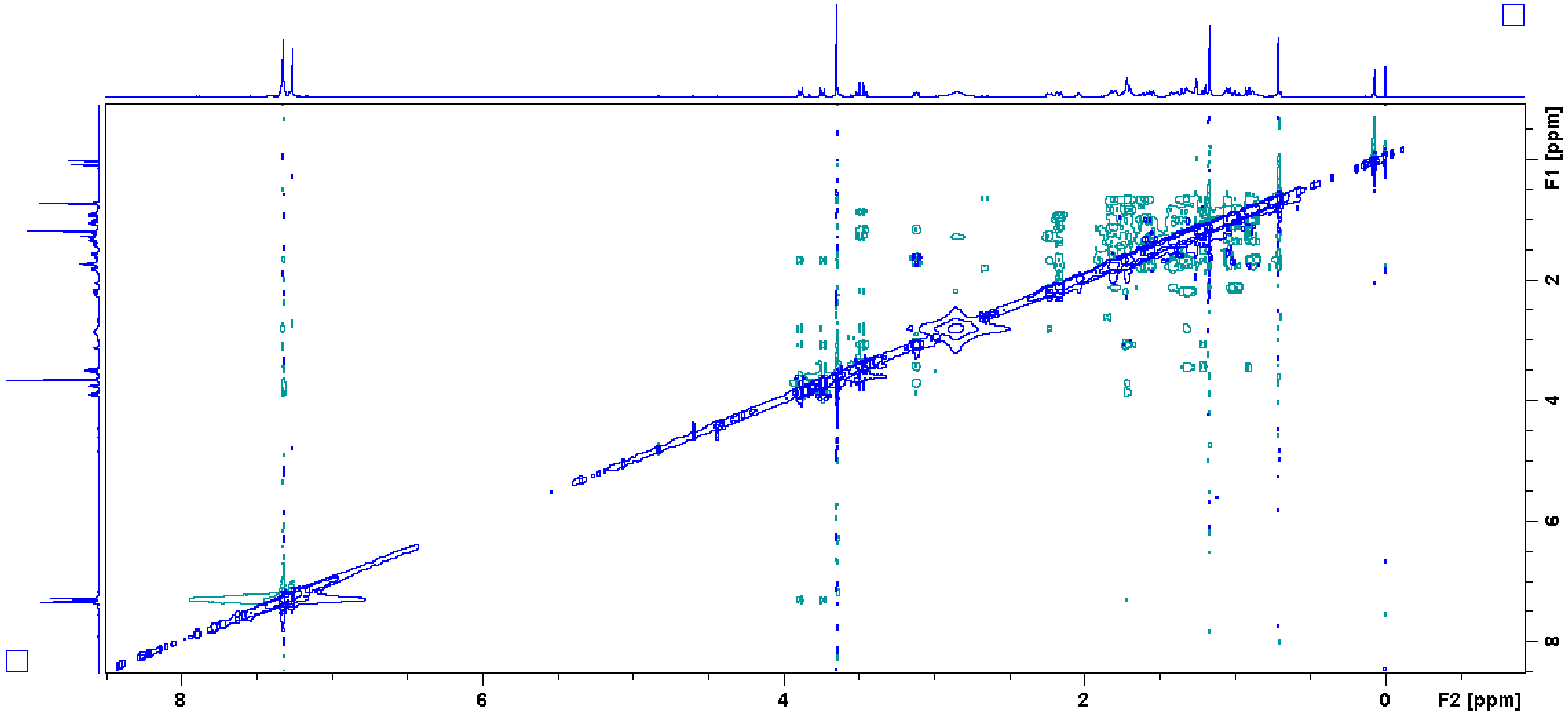

HSQC of compound (4*R*,4a*S*,6a*R*,8*R*,9*R*,11a*R*,11b*S*)-methyl 8-(benzylamino)-9-(hydroxymethyl)-4,11b-dimethyltetradecahydro-6a,9-methanocyclohepta[a]naphthalene-4-carboxylate **(8)**

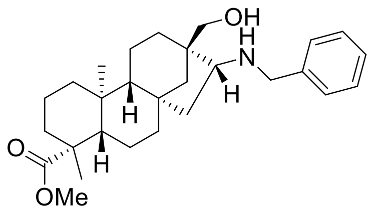

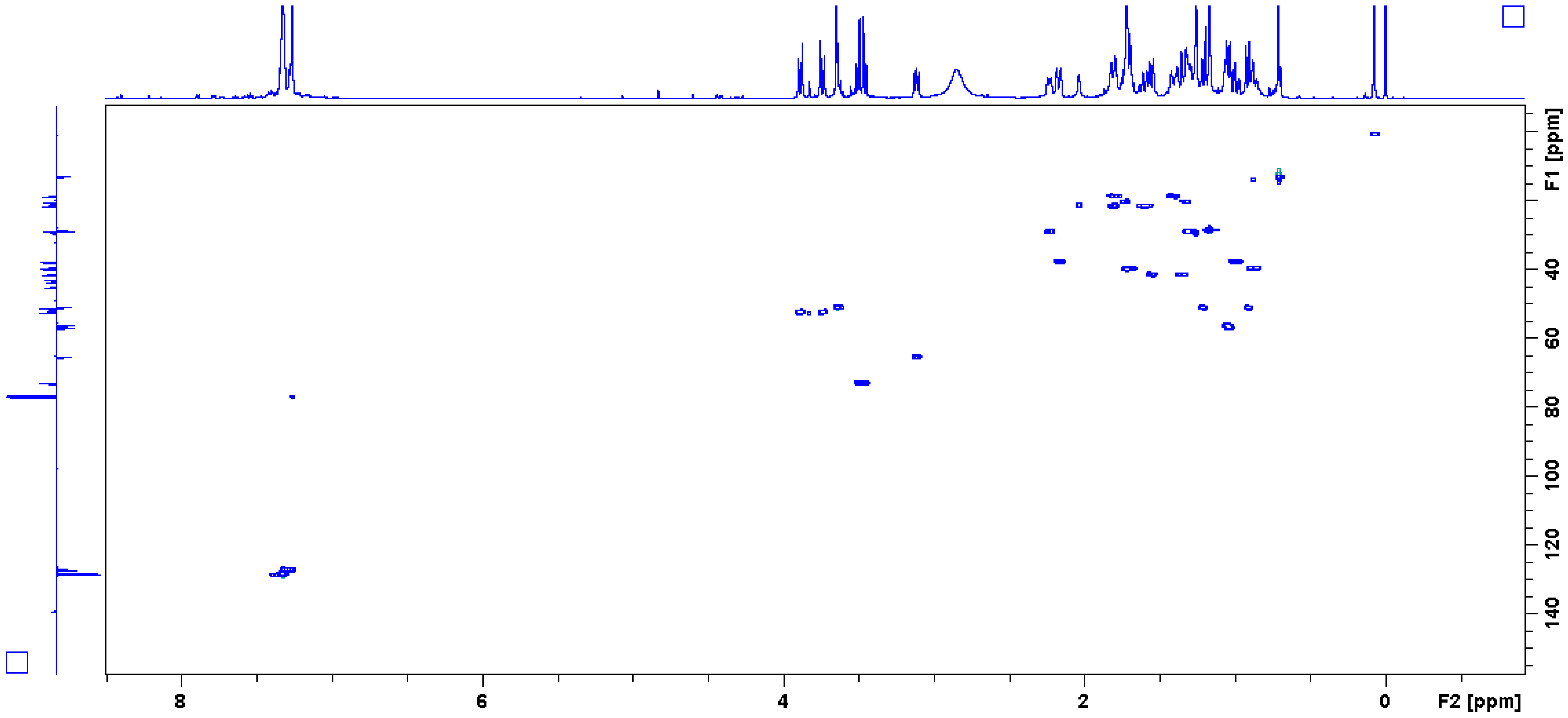

HMBC of compound (4*R*,4a*S*,6a*R*,8*R*,9*R*,11a*R*,11b*S*)-methyl 8-(benzylamino)-9-(hydroxymethyl)-4,11b-dimethyltetradecahydro-6a,9-methanocyclohepta[a]naphthalene-4-carboxylate **(8)**

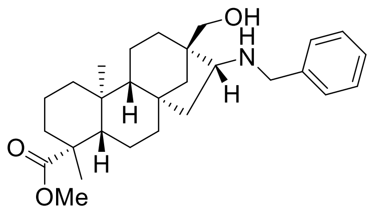

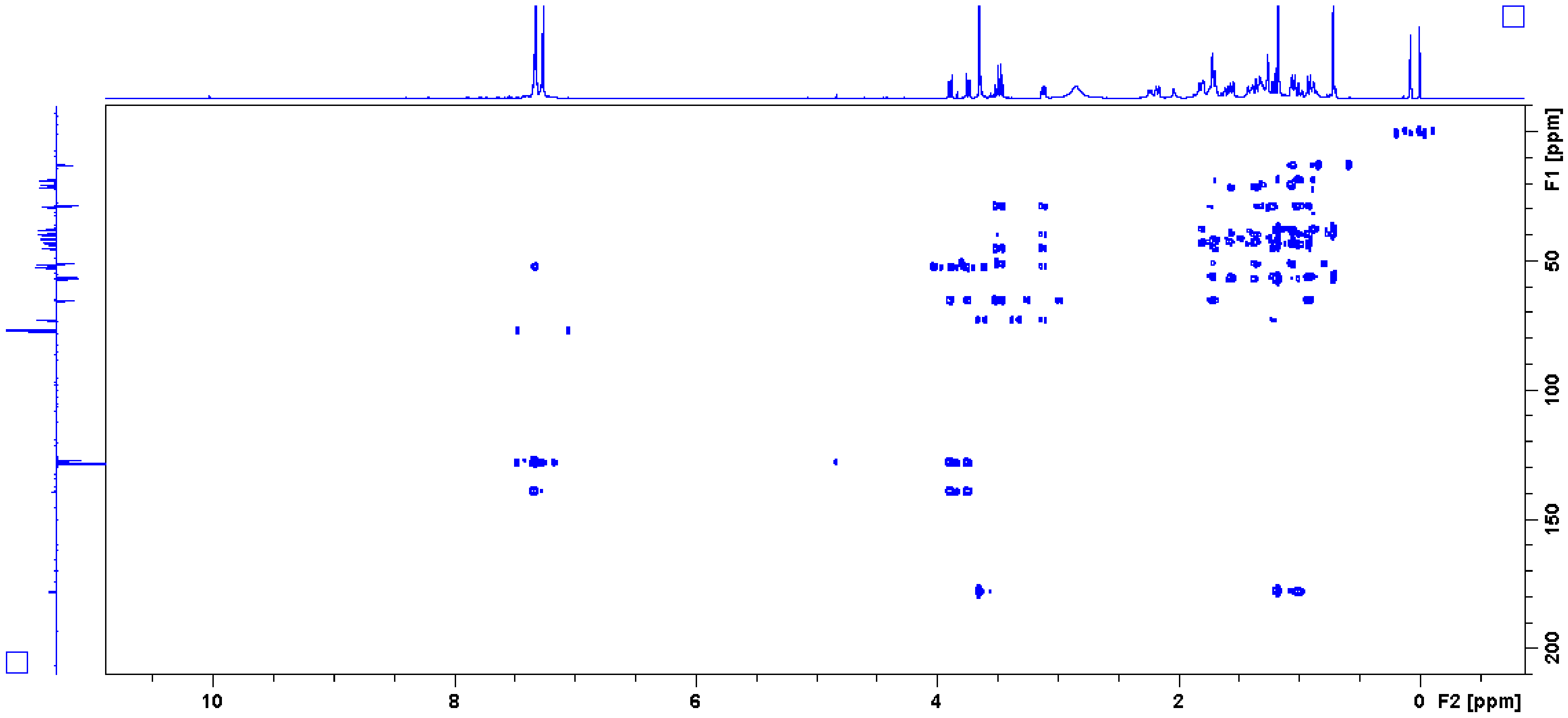

^{1}H-NMR of compound (4*R*,4a*S*,6a*R*,8*R*,9*R*,11a*R*,11b*S*)-methyl 8-((4-fluorobenzyl)amino)-9-(hydroxymethyl)-4,11b-dimethyltetradecahydro-6a,9-methanocyclohepta[a]naphthalene-4-carboxylate **(9)**

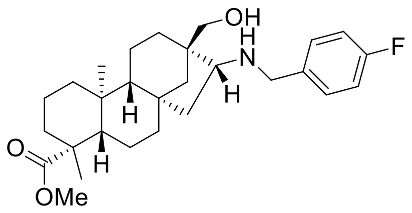

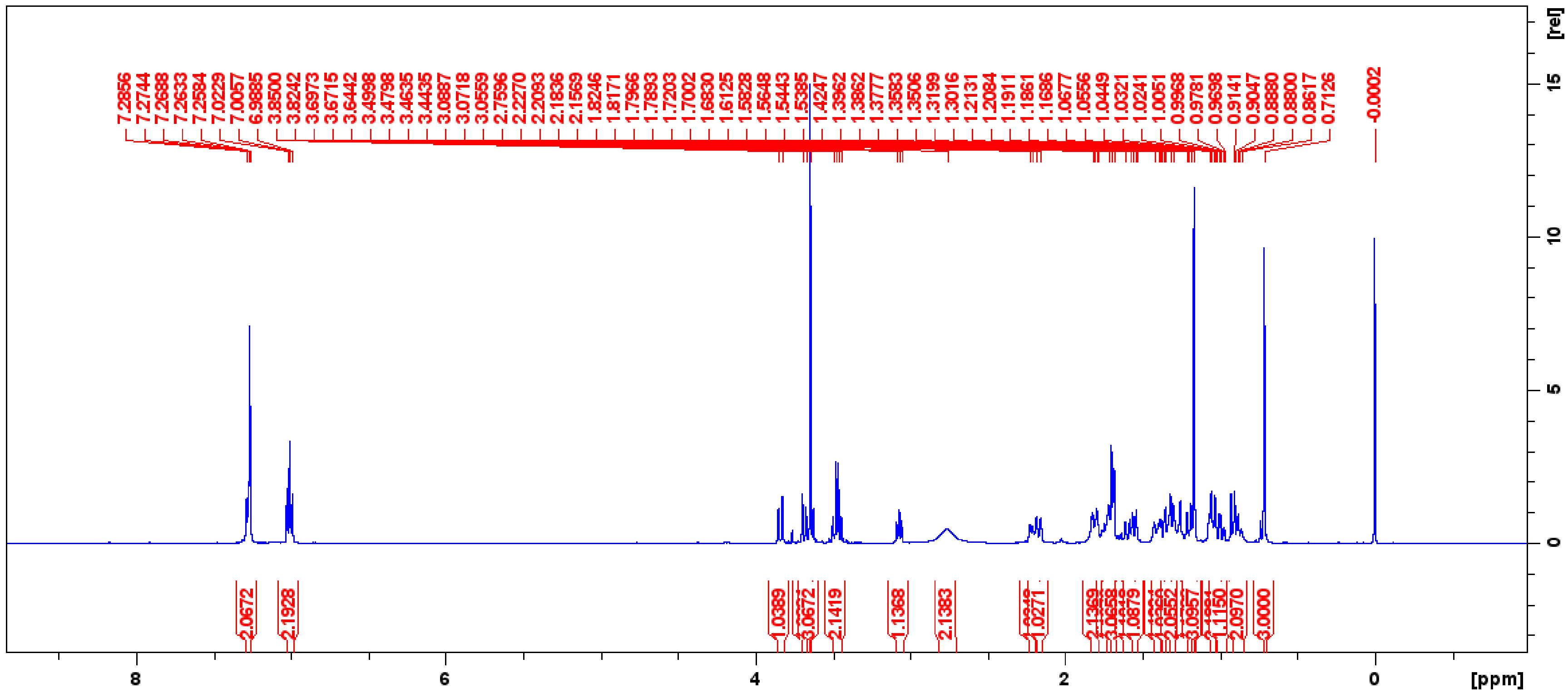

^{13}C-NMR of compound (4*R*,4a*S*,6a*R*,8*R*,9*R*,11a*R*,11b*S*)-methyl 8-((4-fluorobenzyl)amino)-9-(hydroxymethyl)-4,11b-dimethyltetradecahydro-6a,9-methanocyclohepta[a]naphthalene-4-carboxylate **(9)**

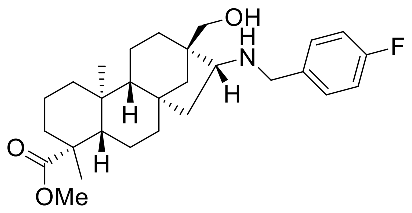

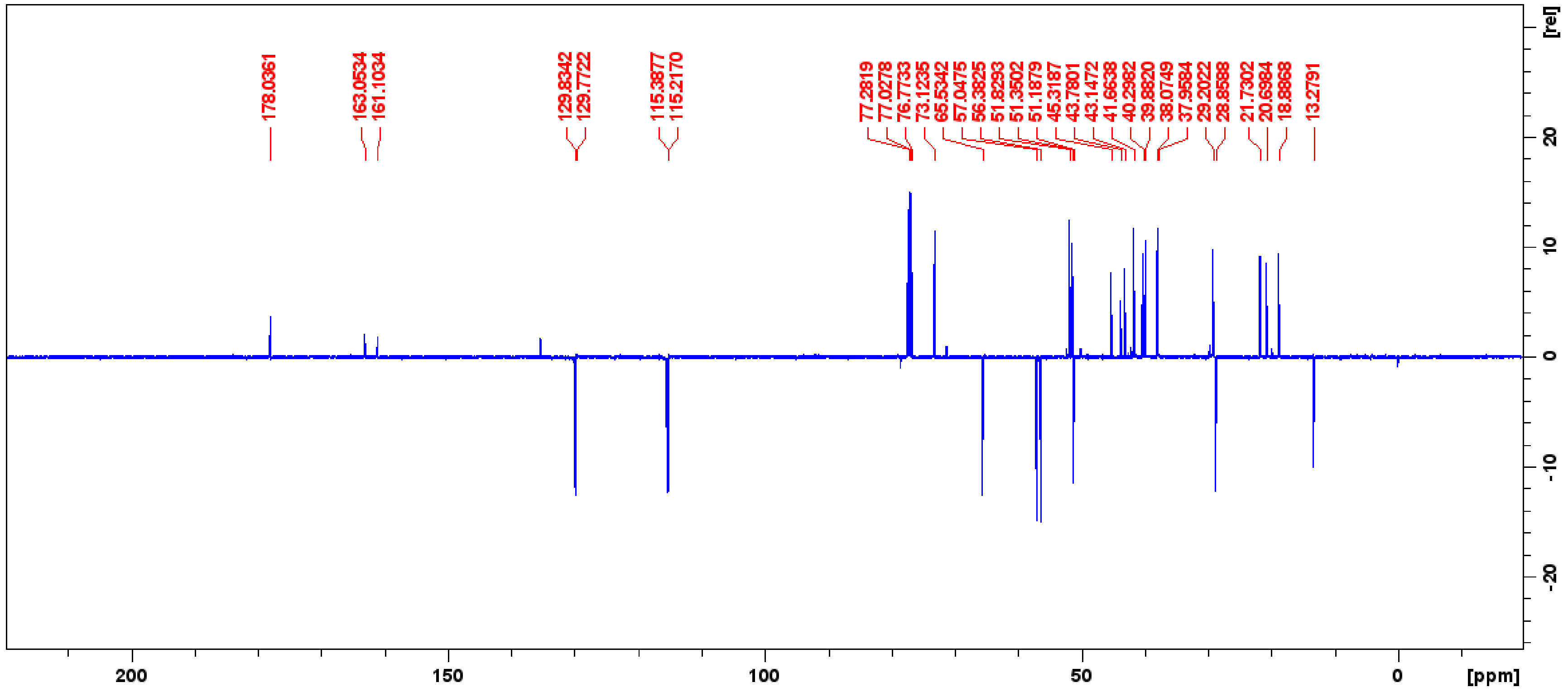

COSY of compound (4*R*,4a*S*,6a*R*,8*R*,9*R*,11a*R*,11b*S*)-methyl 8-((4-fluorobenzyl)amino)-9-(hydroxymethyl)-4,11b-dimethyltetradecahydro-6a,9-methanocyclohepta[a]naphthalene-4-carboxylate **(9)**

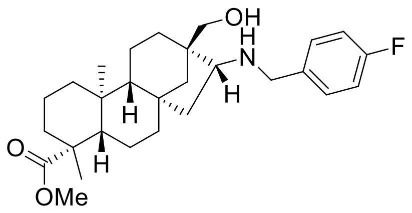

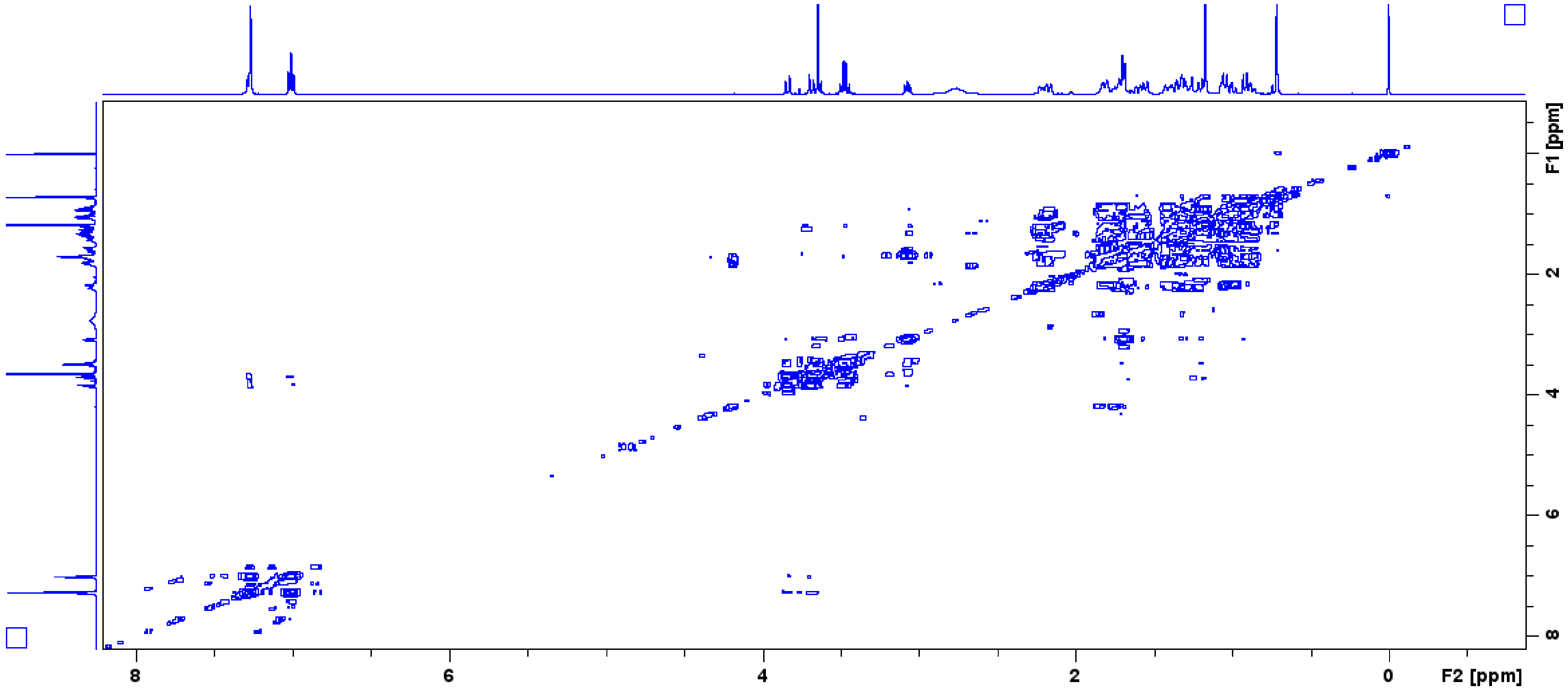

NOESY of compound (4*R*,4a*S*,6a*R*,8*R*,9*R*,11a*R*,11b*S*)-methyl 8-((4-fluorobenzyl)amino)-9-(hydroxymethyl)-4,11b-dimethyltetradecahydro-6a,9-methanocyclohepta[a]naphthalene-4-carboxylate **(9)**

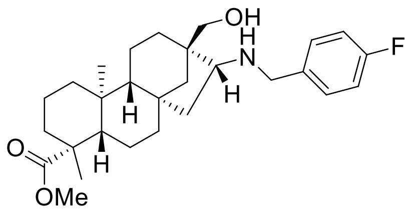

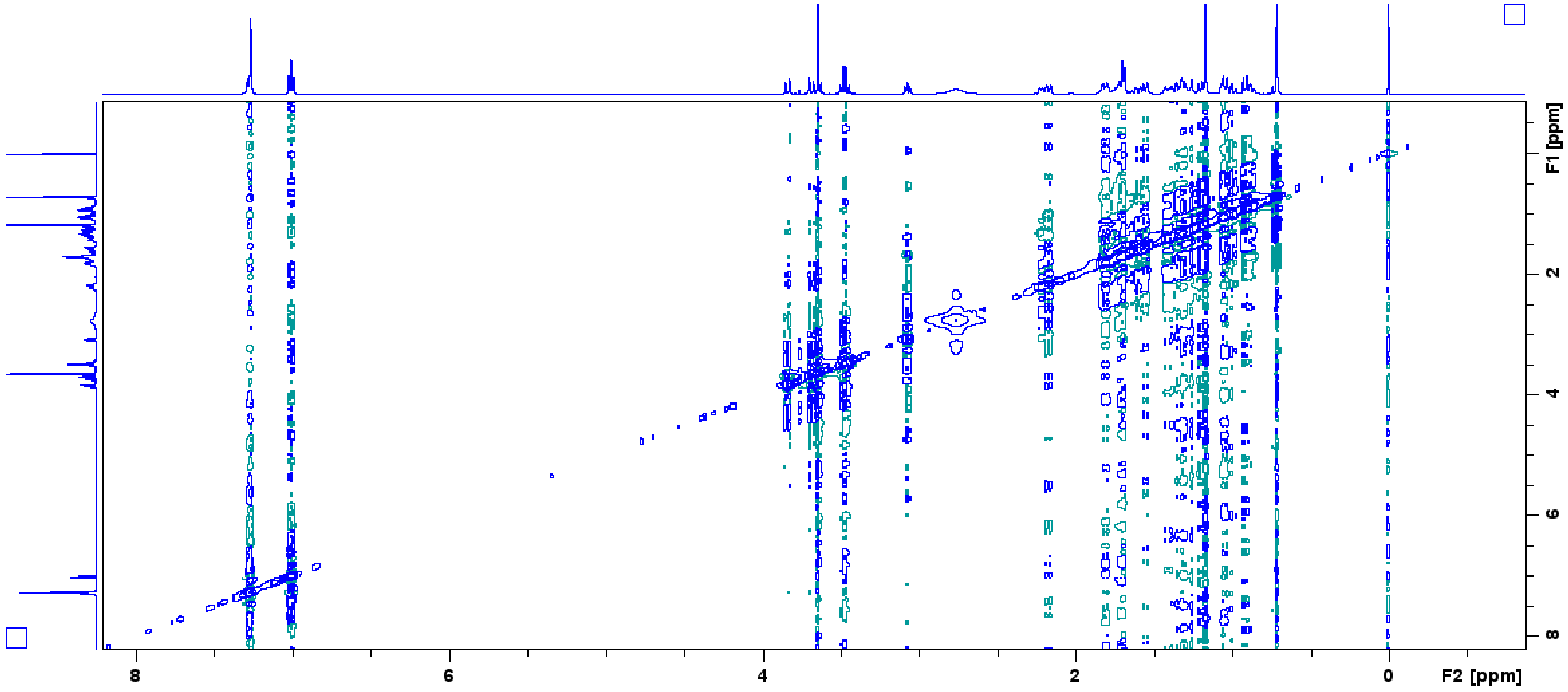

HSQC of compound (4*R*,4a*S*,6a*R*,8*R*,9*R*,11a*R*,11b*S*)-methyl 8-((4-fluorobenzyl)amino)-9-(hydroxymethyl)-4,11b-dimethyltetradecahydro-6a,9-methanocyclohepta[a]naphthalene-4-carboxylate **(9)**

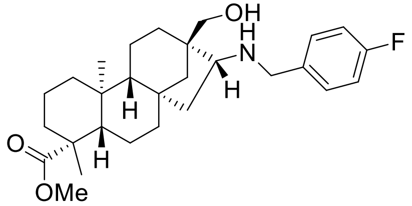

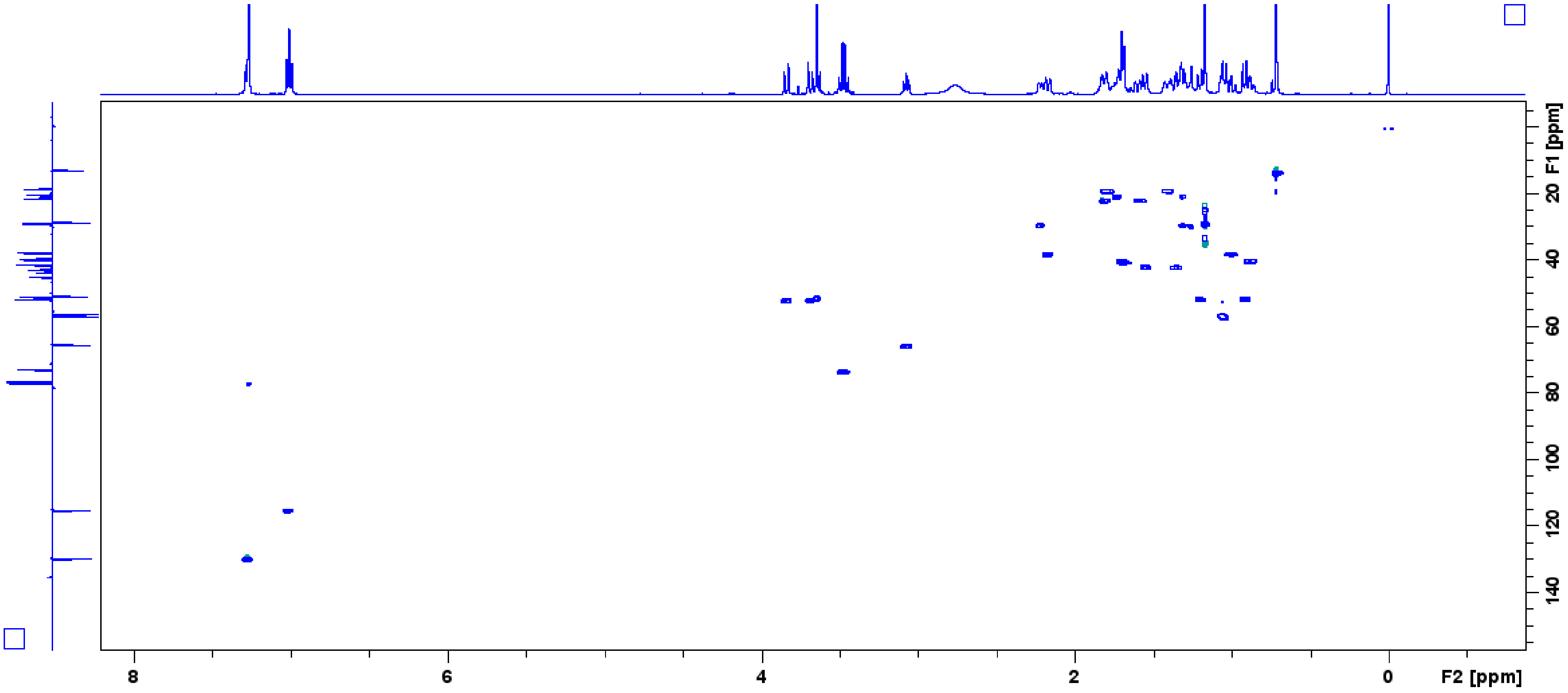

HMBC of compound (4*R*,4a*S*,6a*R*,8*R*,9*R*,11a*R*,11b*S*)-methyl 8-((4-fluorobenzyl)amino)-9-(hydroxymethyl)-4,11b-dimethyltetradecahydro-6a,9-methanocyclohepta[a]naphthalene-4-carboxylate **(9)**

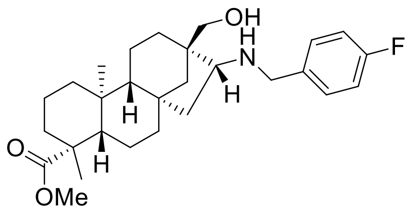

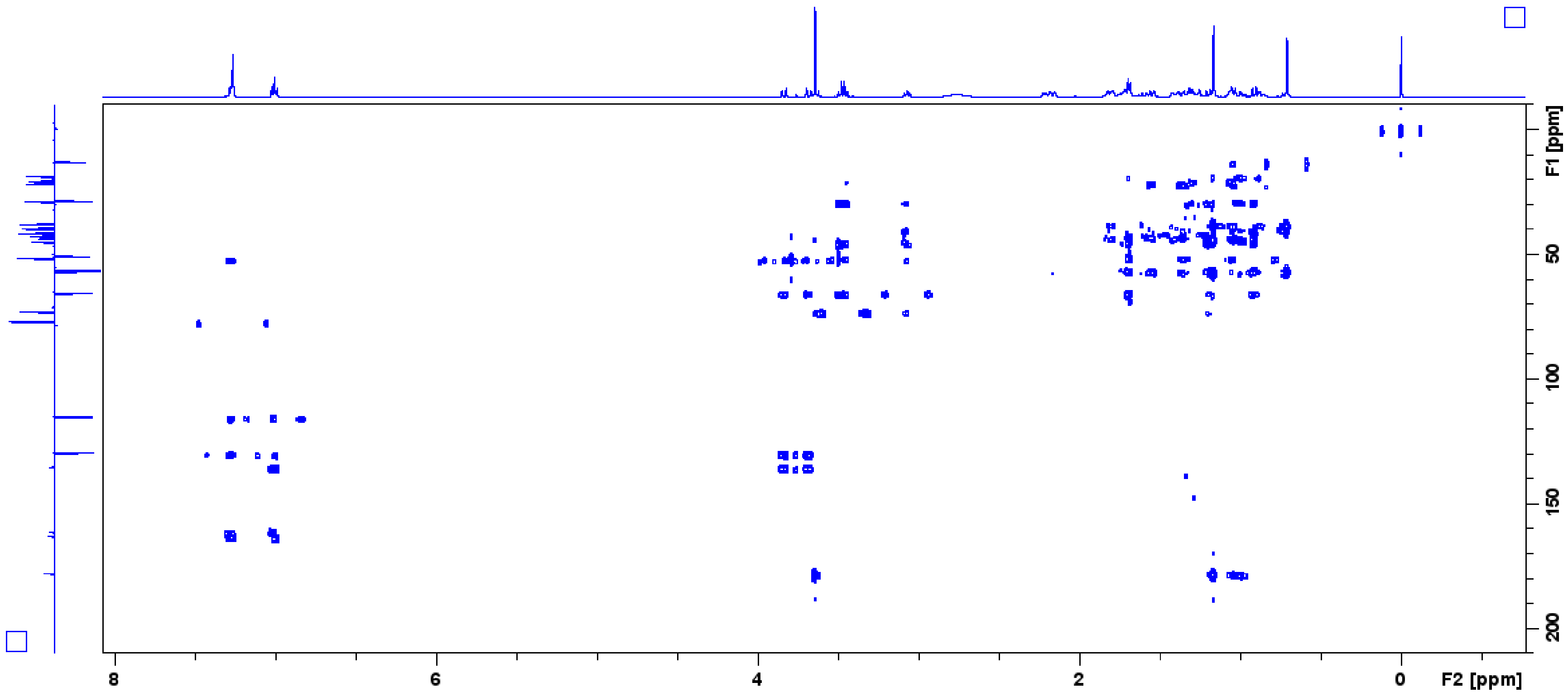

^{19}F-NMR of compound (4*R*,4a*S*,6a*R*,8*R*,9*R*,11a*R*,11b*S*)-methyl 8-((4-fluorobenzyl)amino)-9-(hydroxymethyl)-4,11b-dimethyltetradecahydro-6a,9-methanocyclohepta[a]naphthalene-4-carboxylate **(9)**

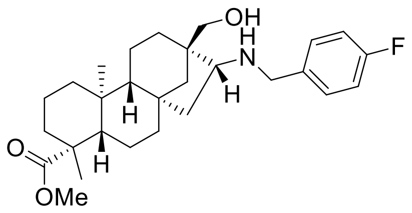

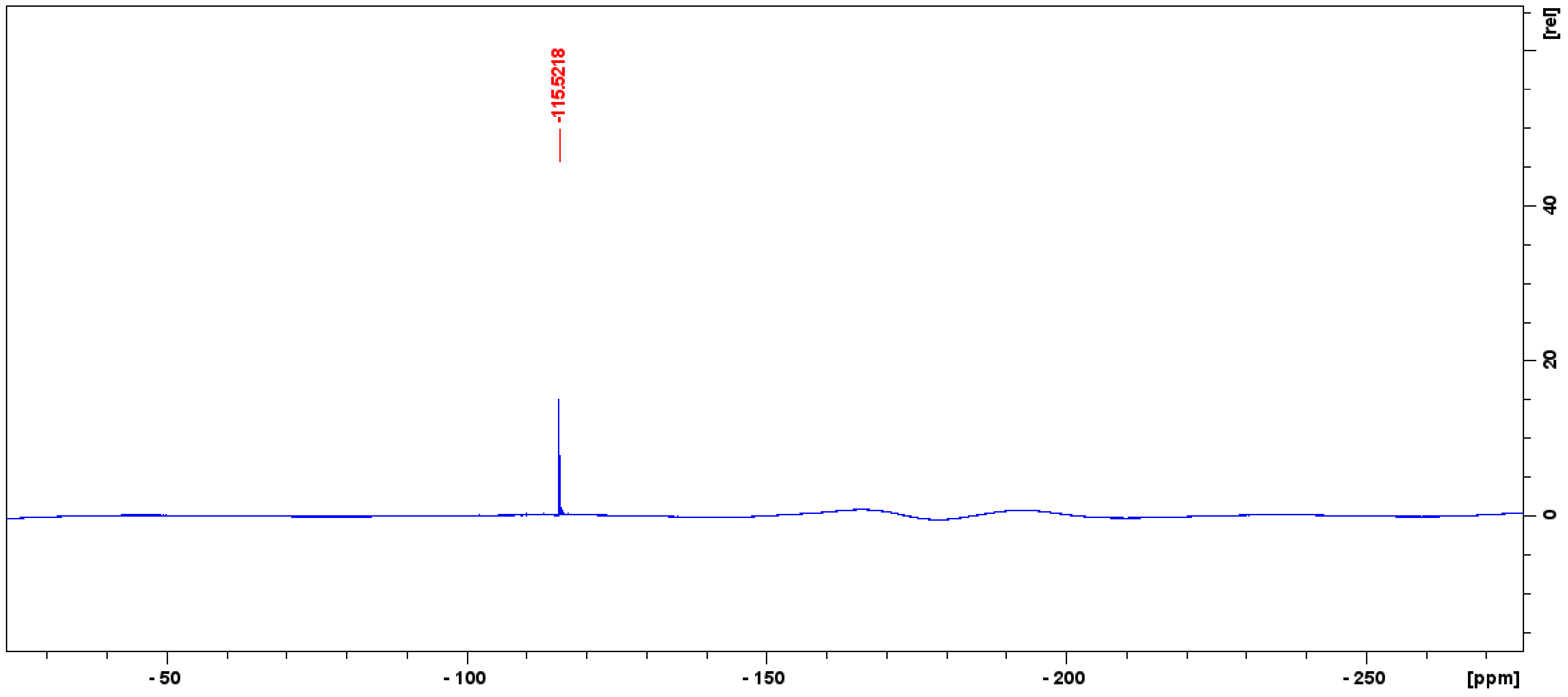

¹H-NMR of compound (4*R*,4a*S*,6a*R*,8*R*,9*R*,11a*R*,11b*S*)-methyl 9-(hydroxymethyl)-8-((4-methoxybenzyl)amino)-4,11b-dimethyltetradecahydro-6a,9-methanocyclohepta[a]naphthalene-4-carboxylate **(10)**

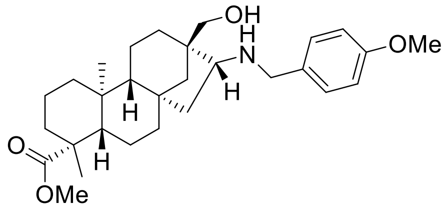

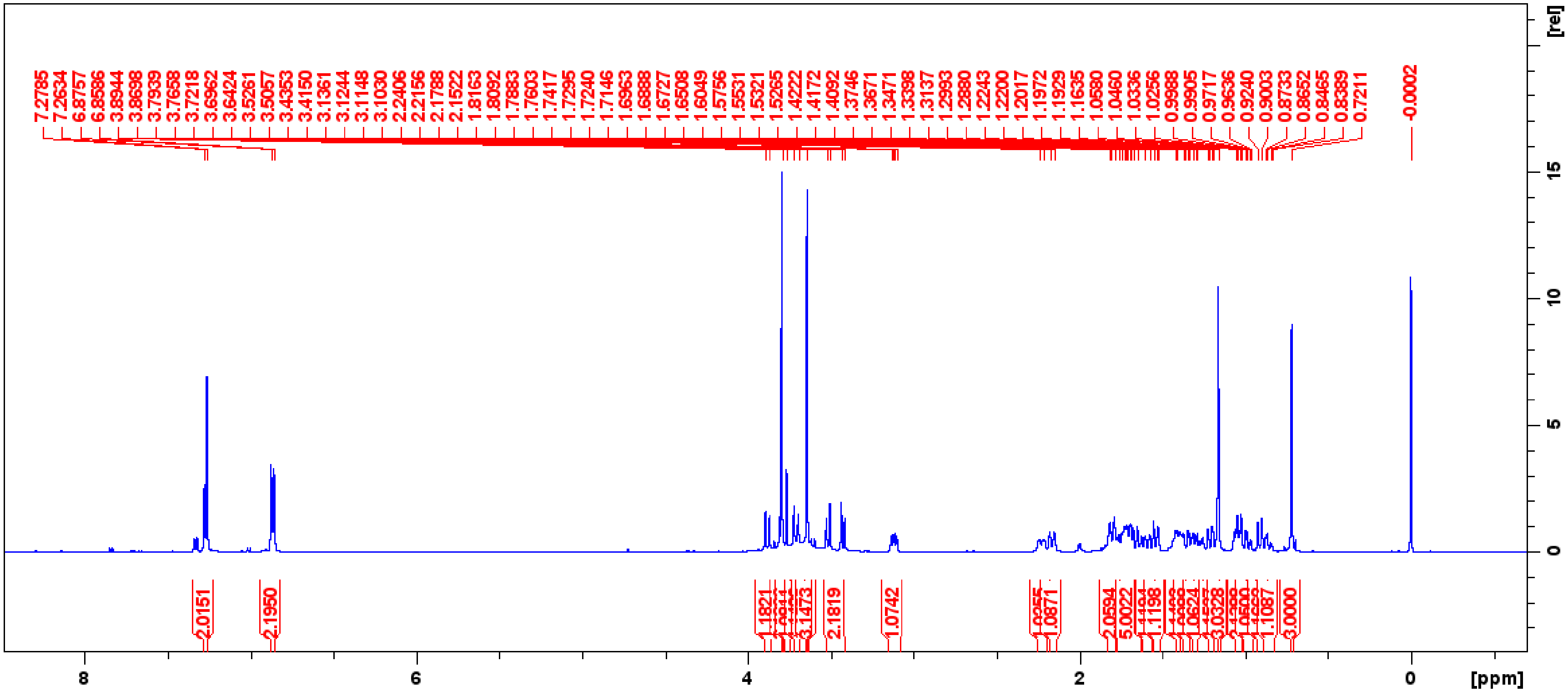

^{13}C-NMR of compound (4*R*,4a*S*,6a*R*,8*R*,9*R*,11a*R*,11b*S*)-methyl 9-(hydroxymethyl)-8-((4-methoxybenzyl)amino)-4,11b-dimethyltetradecahydro-6a,9-methanocyclohepta[a]naphthalene-4-carboxylate **(10)**

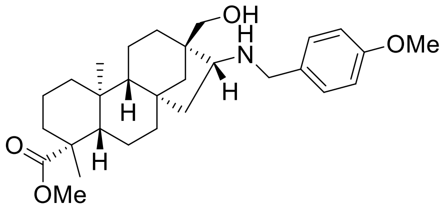

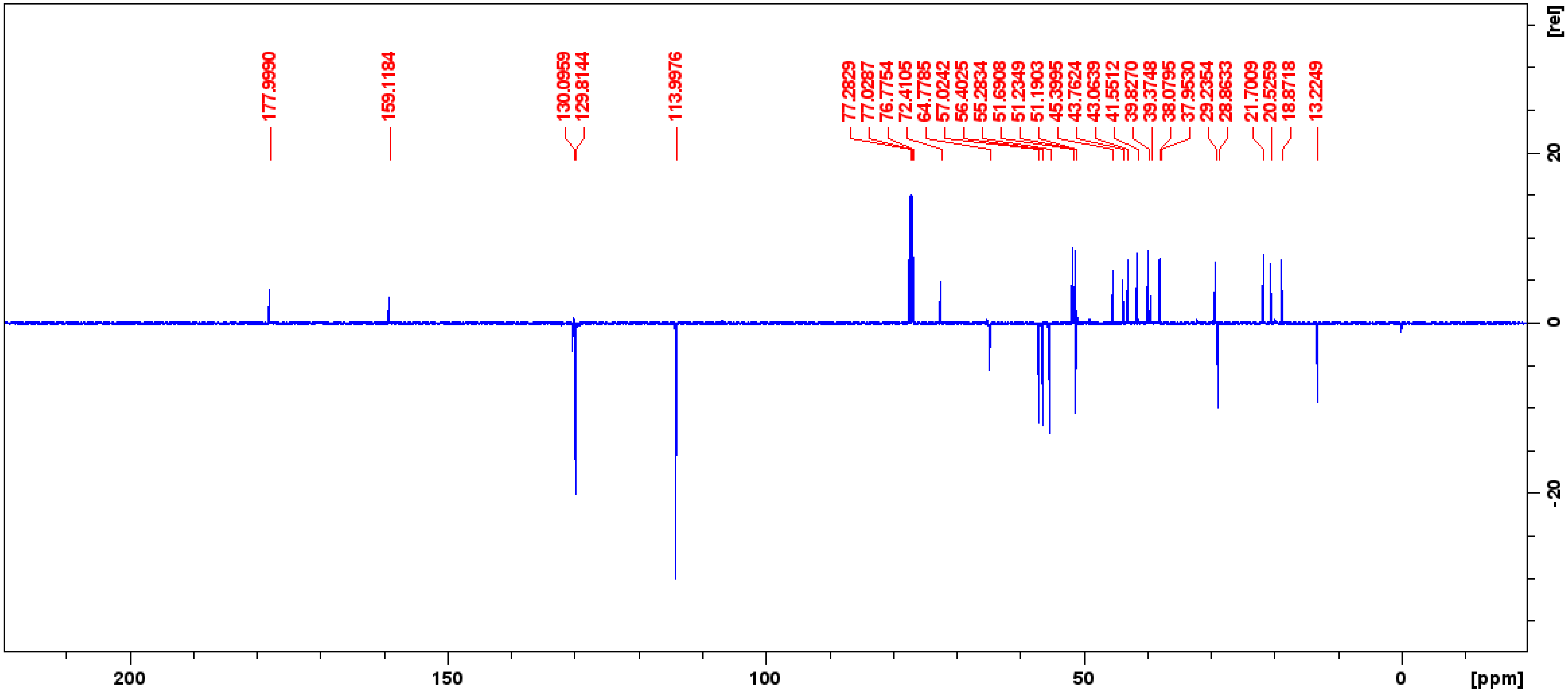

COSY of compound (4*R*,4a*S*,6a*R*,8*R*,9*R*,11a*R*,11b*S*)-methyl 9-(hydroxymethyl)-8-((4-methoxybenzyl)amino)-4,11b-dimethyltetradecahydro-6a,9-methanocyclohepta[a]naphthalene-4-carboxylate **(10)**

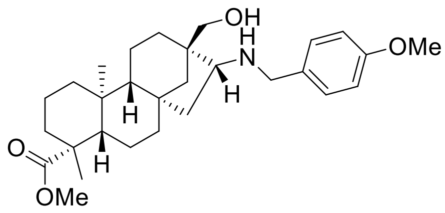

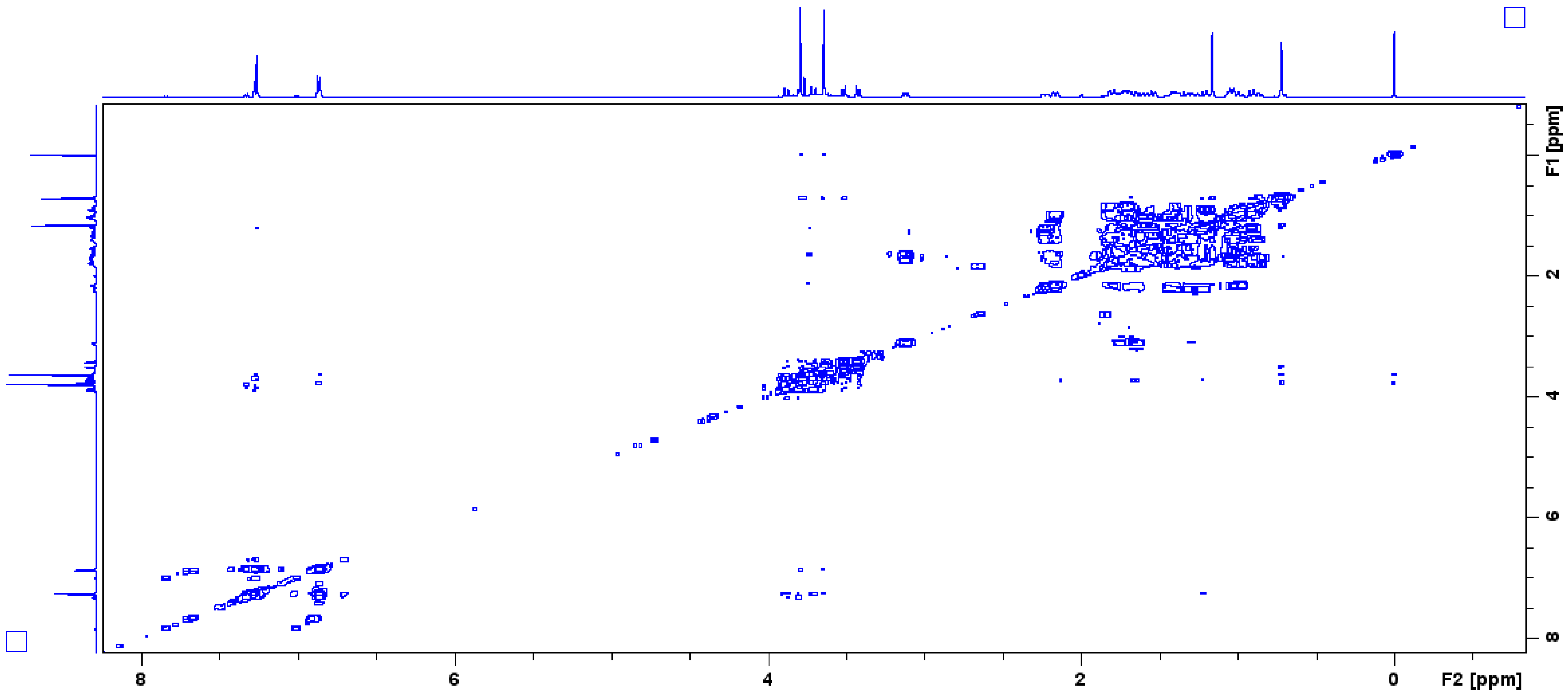

NOESY of compound (4*R*,4a*S*,6a*R*,8*R*,9*R*,11a*R*,11b*S*)-methyl 9-(hydroxymethyl)-8-((4-methoxybenzyl)amino)-4,11b-dimethyltetradecahydro-6a,9-methanocyclohepta[a]naphthalene-4-carboxylate **(10)**

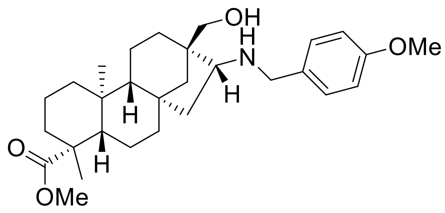

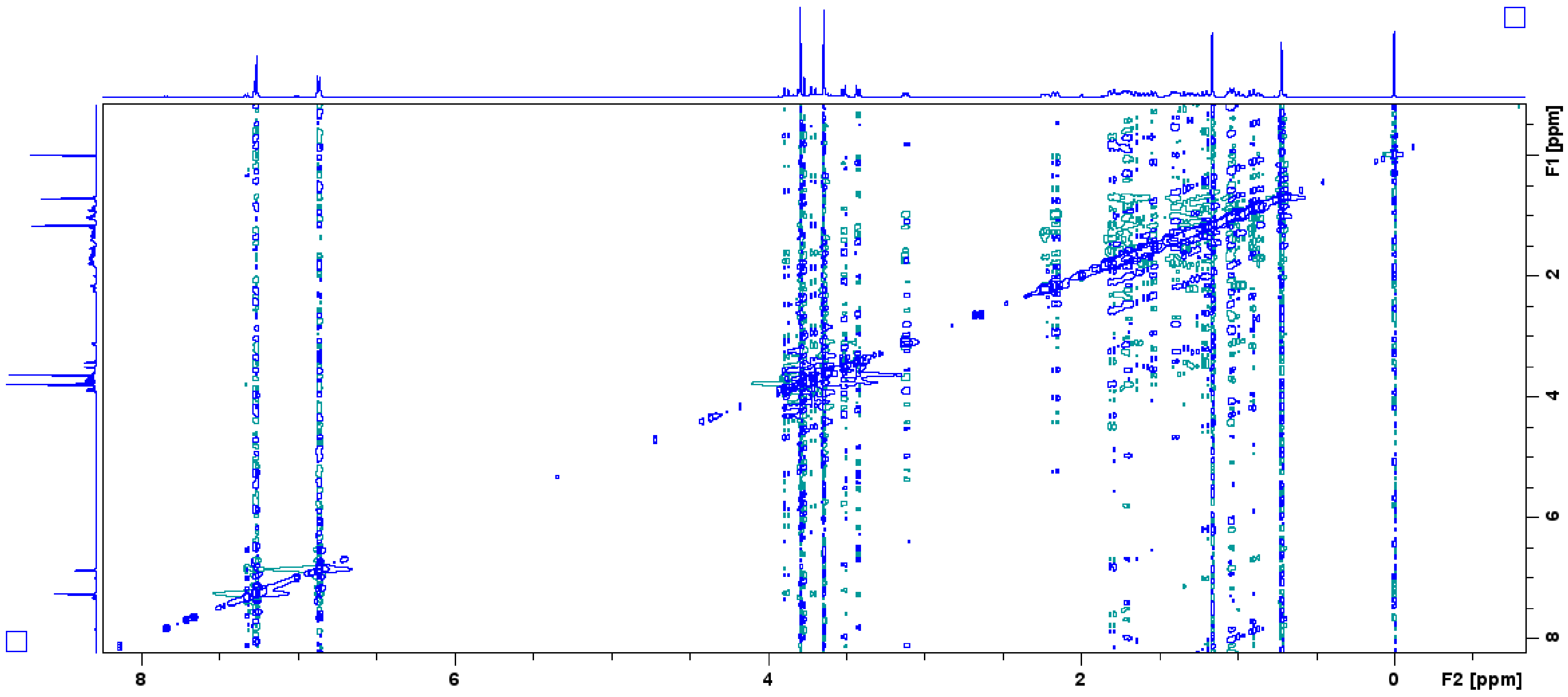

HSQC of compound (4*R*,4a*S*,6a*R*,8*R*,9*R*,11a*R*,11b*S*)-methyl 9-(hydroxymethyl)-8-((4-methoxybenzyl)amino)-4,11b-dimethyltetradecahydro-6a,9-methanocyclohepta[a]naphthalene-4-carboxylate **(10)**

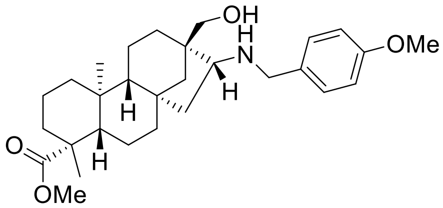

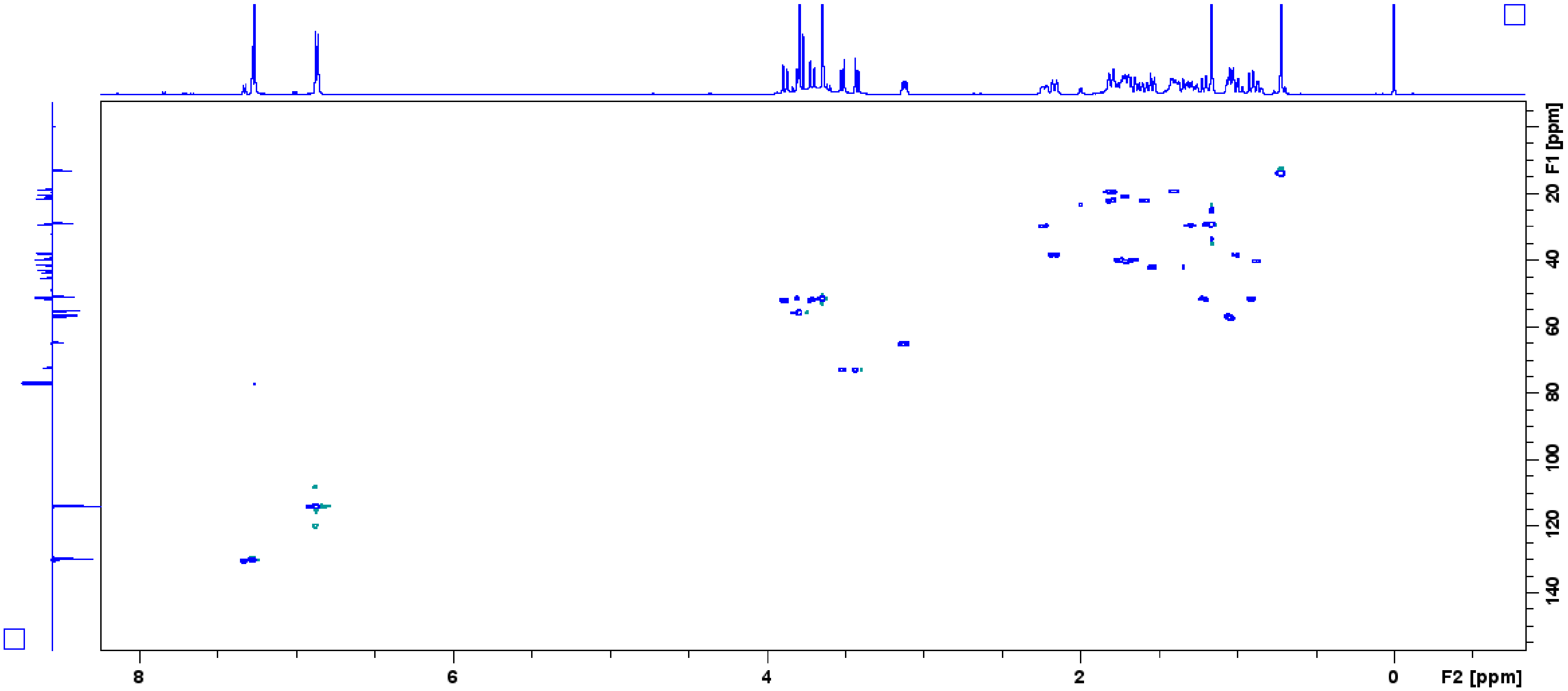

HMBC of compound (4*R*,4a*S*,6a*R*,8*R*,9*R*,11a*R*,11b*S*)-methyl 9-(hydroxymethyl)-8-((4-methoxybenzyl)amino)-4,11b-dimethyltetradecahydro-6a,9-methanocyclohepta[a]naphthalene-4-carboxylate **(10)**

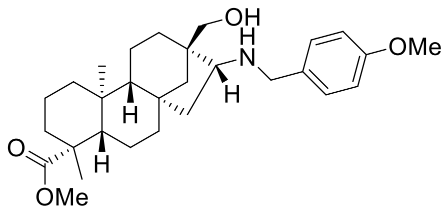

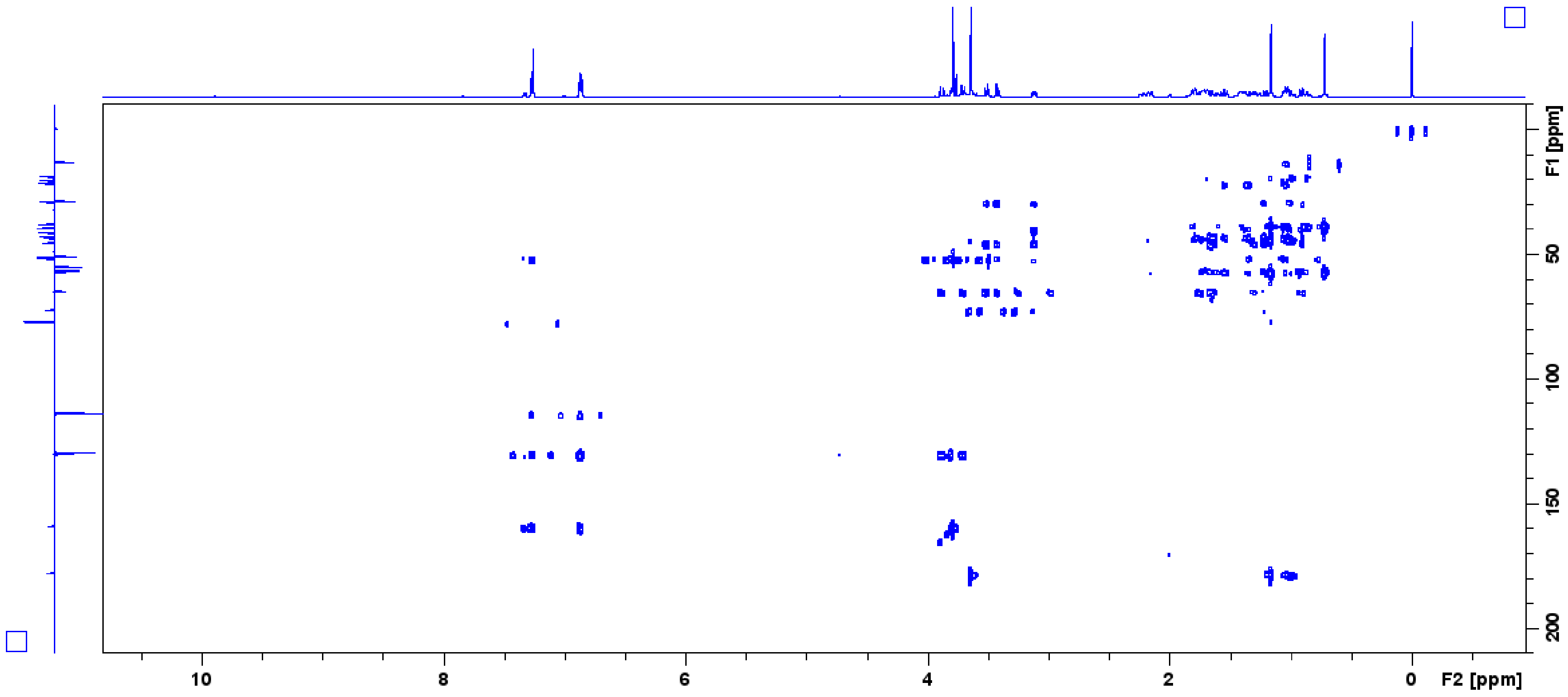

^{1}H-NMR of compound (4*R*,4a*S*,6a*R*,8*R*,9*R*,11a*R*,11b*S*)-methyl 9-(hydroxymethyl)-4,11b-dimethyl-8-(((*S*)-1-phenylpropyl)amino)tetradecahydro-6a,9-methanocyclohepta[a]naphthalene-4-carboxylate **(11)**

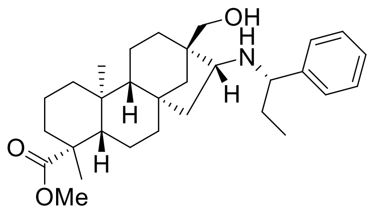

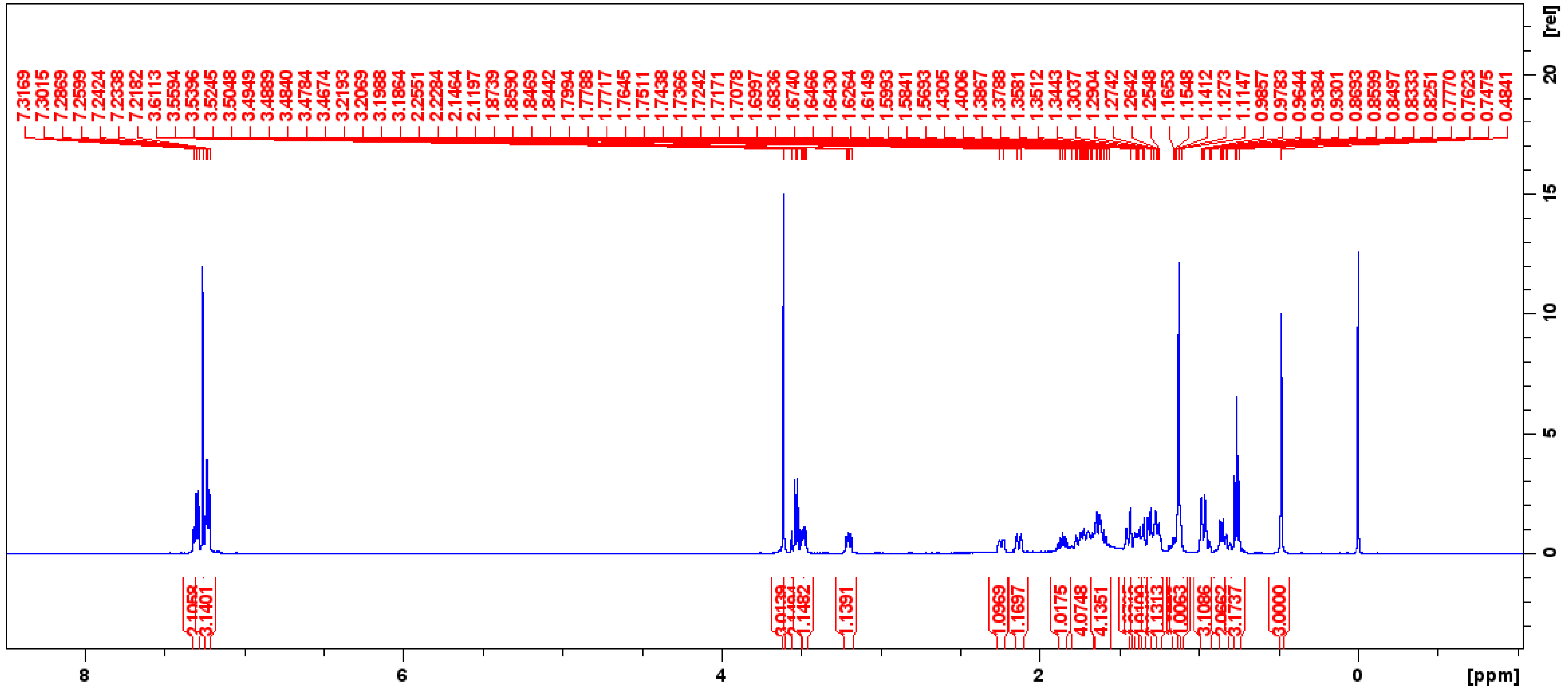

^{13}C-NMR of compound (4*R*,4a*S*,6a*R*,8*R*,9*R*,11a*R*,11b*S*)-methyl 9-(hydroxymethyl)-4,11b-dimethyl-8-(((*S*)-1-phenylpropyl)amino)tetradecahydro-6a,9-methanocyclohepta[a]naphthalene-4-carboxylate **(11)**

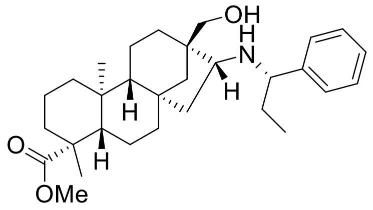

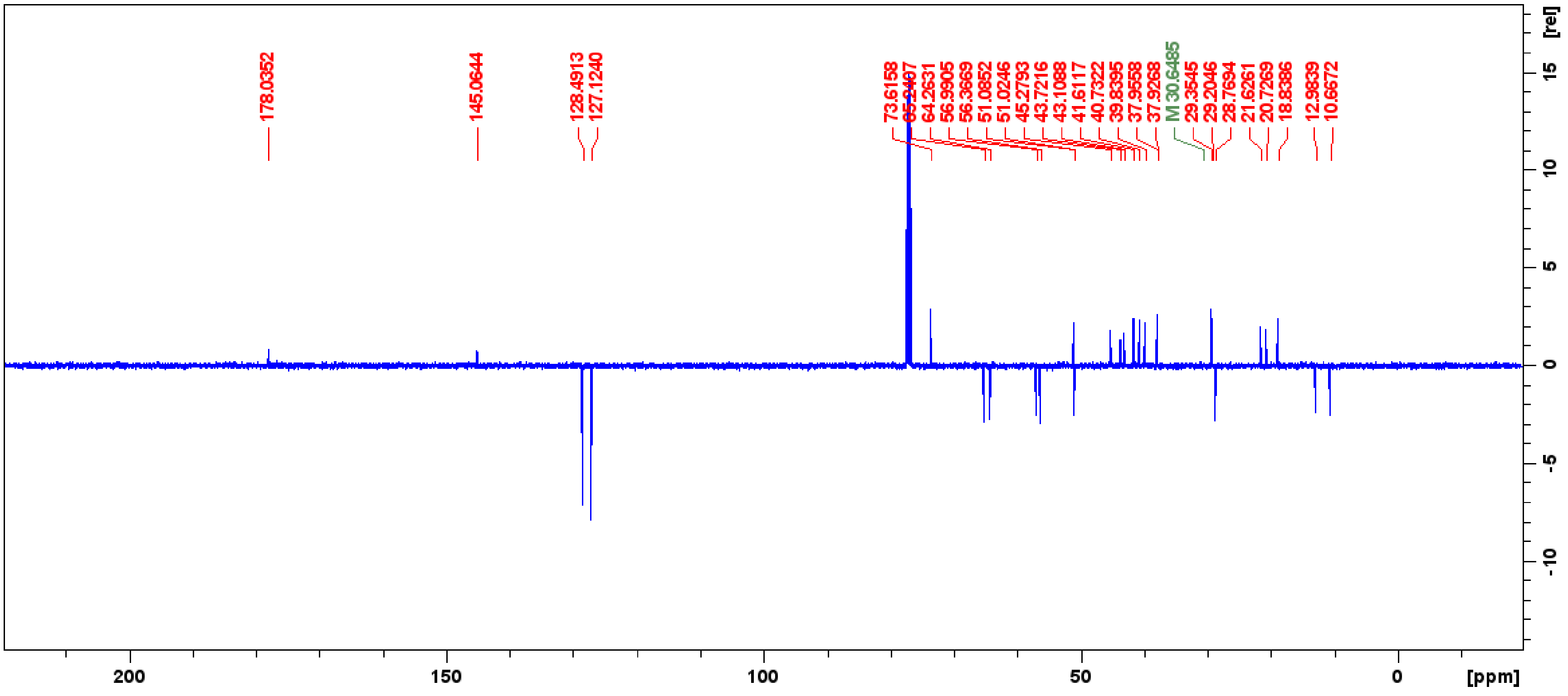

COSY of compound (4*R*,4a*S*,6a*R*,8*R*,9*R*,11a*R*,11b*S*)-methyl 9-(hydroxymethyl)-4,11b-dimethyl-8-(((*S*)-1-phenylpropyl)amino)tetradecahydro-6a,9-methanocyclohepta[a]naphthalene-4-carboxylate **(11)**

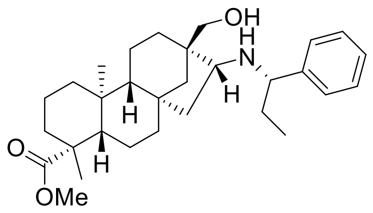

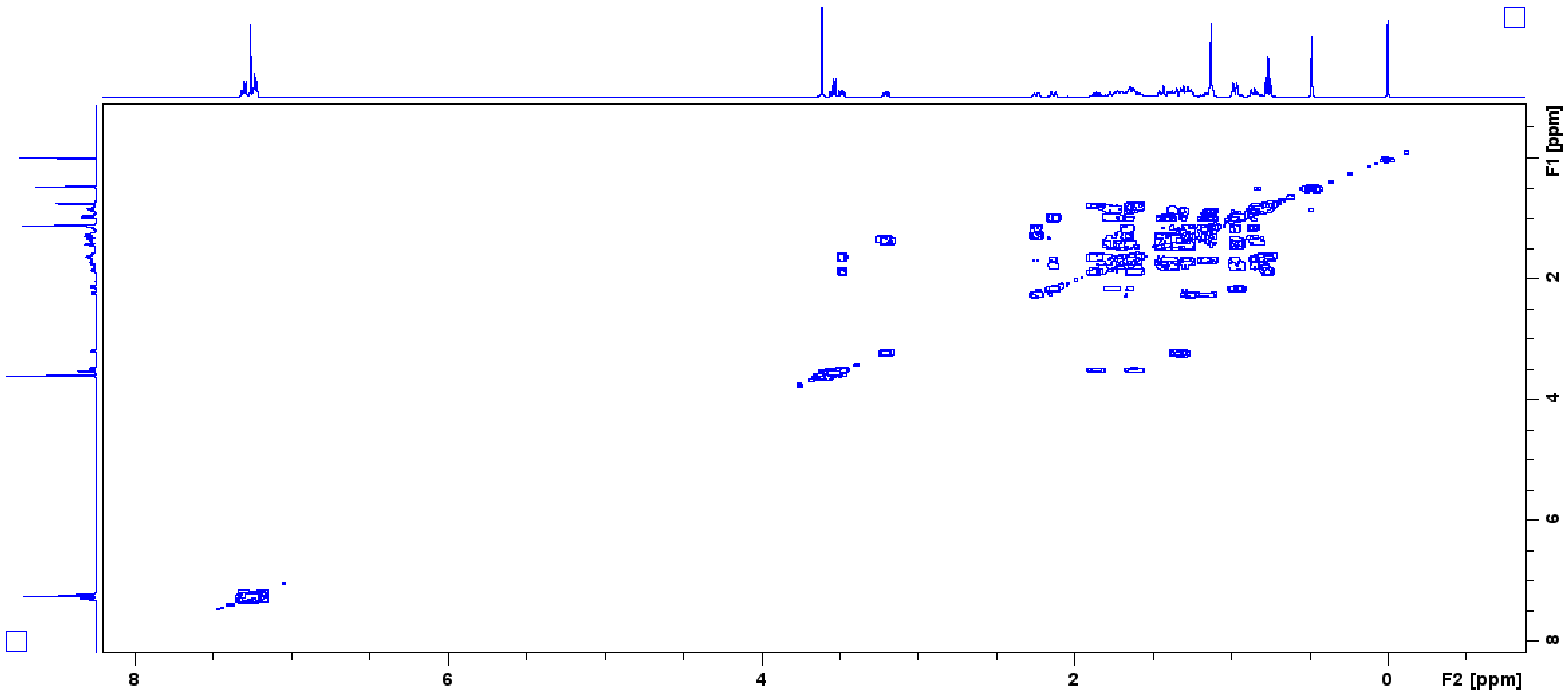

NOESY of compound (4*R*,4a*S*,6a*R*,8*R*,9*R*,11a*R*,11b*S*)-methyl 9-(hydroxymethyl)-4,11b-dimethyl-8-(((*S*)-1-phenylpropyl)amino)tetradecahydro-6a,9-methanocyclohepta[a]naphthalene-4-carboxylate **(11)**

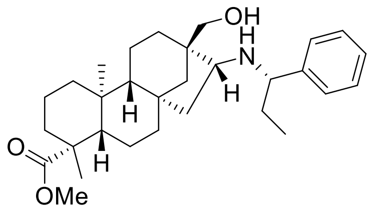
OH
H
N
H
H
H
O
OMe

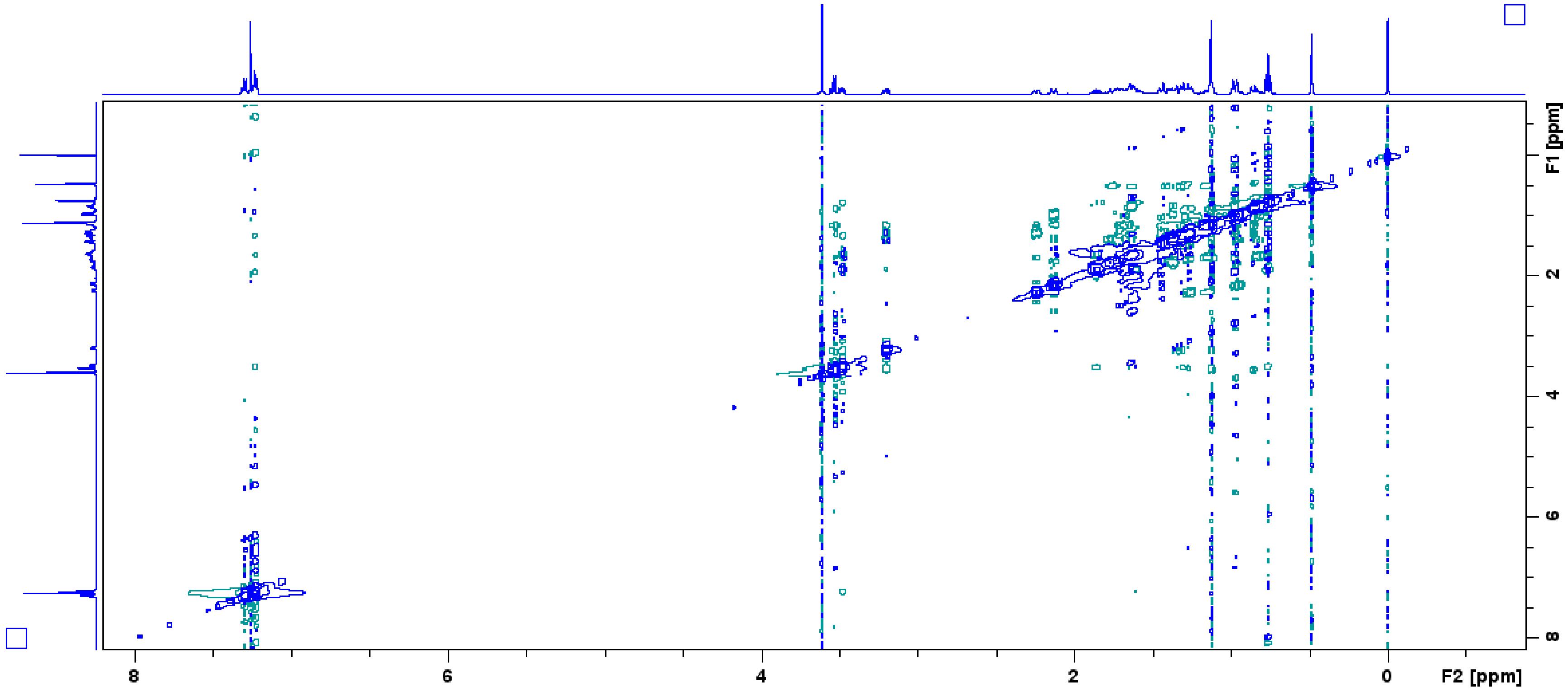
F1 [ppm]
F2 [ppm]
8
6
4
2
0

HSQC of compound (4*R*,4a*S*,6a*R*,8*R*,9*R*,11a*R*,11b*S*)-methyl 9-(hydroxymethyl)-4,11b-dimethyl-8-(((*S*)-1-phenylpropyl)amino)tetradecahydro-6a,9-methanocyclohepta[a]naphthalene-4-carboxylate **(11)**

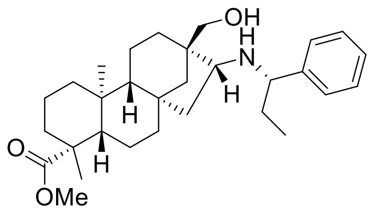

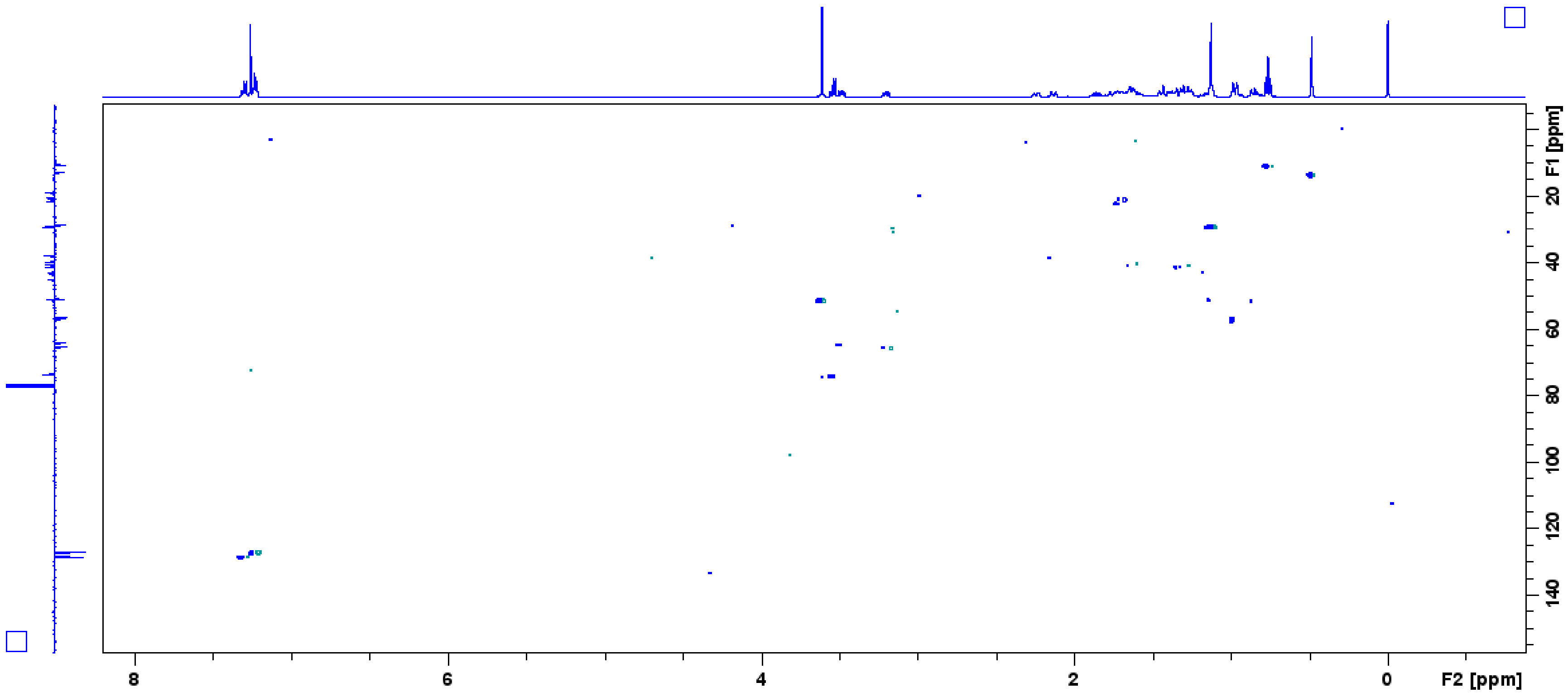

HMBC of compound (4*R*,4a*S*,6a*R*,8*R*,9*R*,11a*R*,11b*S*)-methyl 9-(hydroxymethyl)-4,11b-dimethyl-8-(((*S*)-1-phenylpropyl)amino)tetradecahydro-6a,9-methanocyclohepta[a]naphthalene-4-carboxylate **(11)**

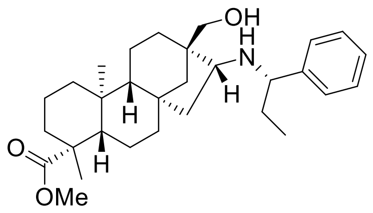

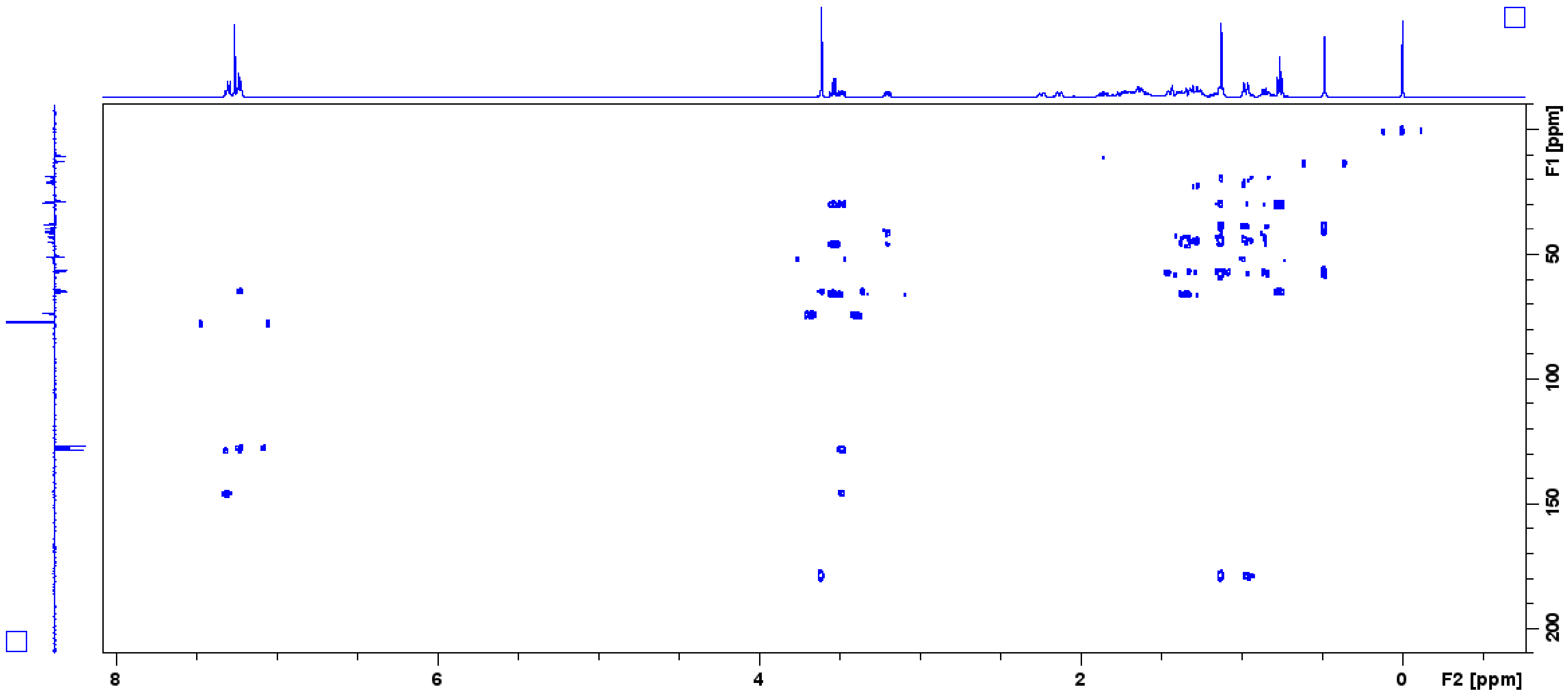

^{1}H-NMR of compound (4*R*,4a*S*,6a*R*,8*R*,9*R*,11a*R*,11b*S*)-methyl 9-(hydroxymethyl)-4,11b-dimethyl-8-(((*R*)-1-phenylpropyl)amino)tetradecahydro-6a,9-methanocyclohepta[a]naphthalene-4-carboxylate **(12)**

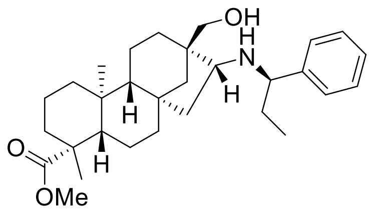

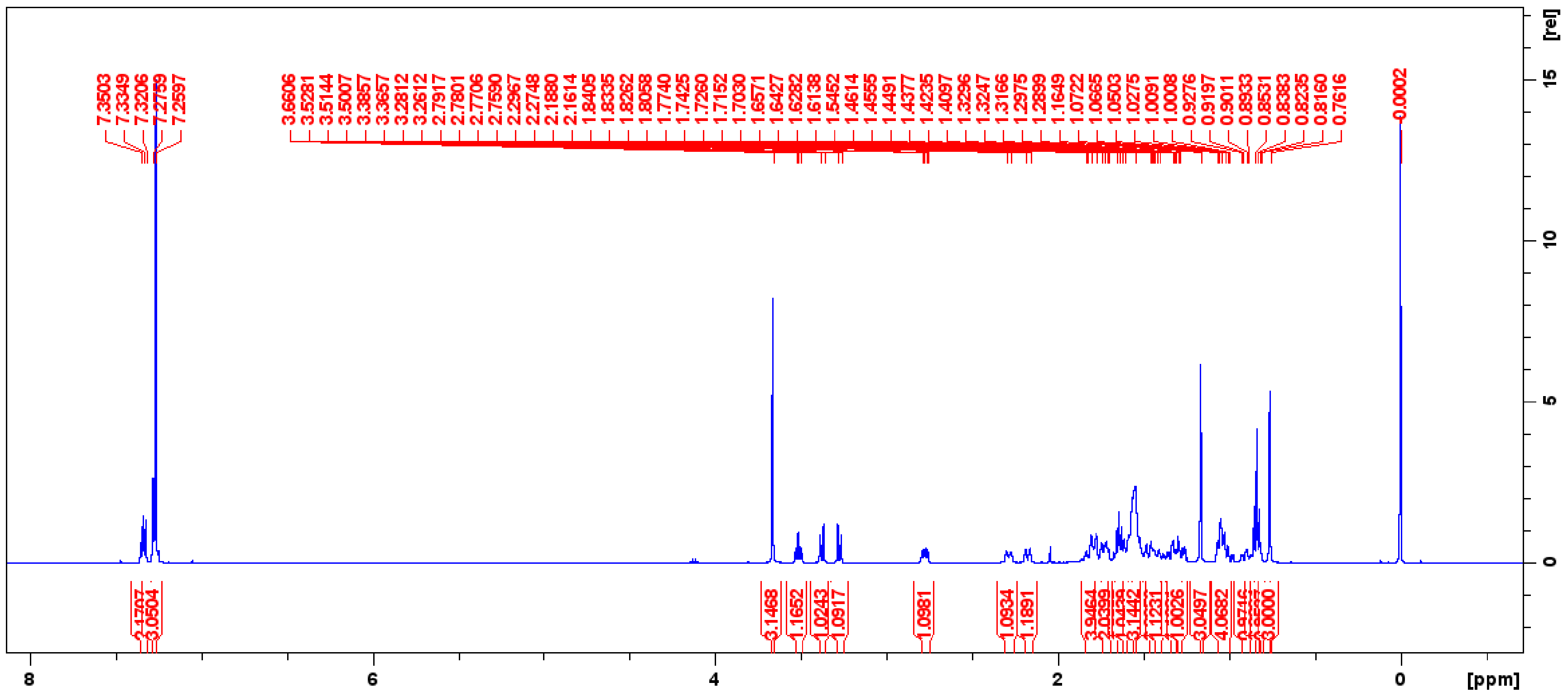

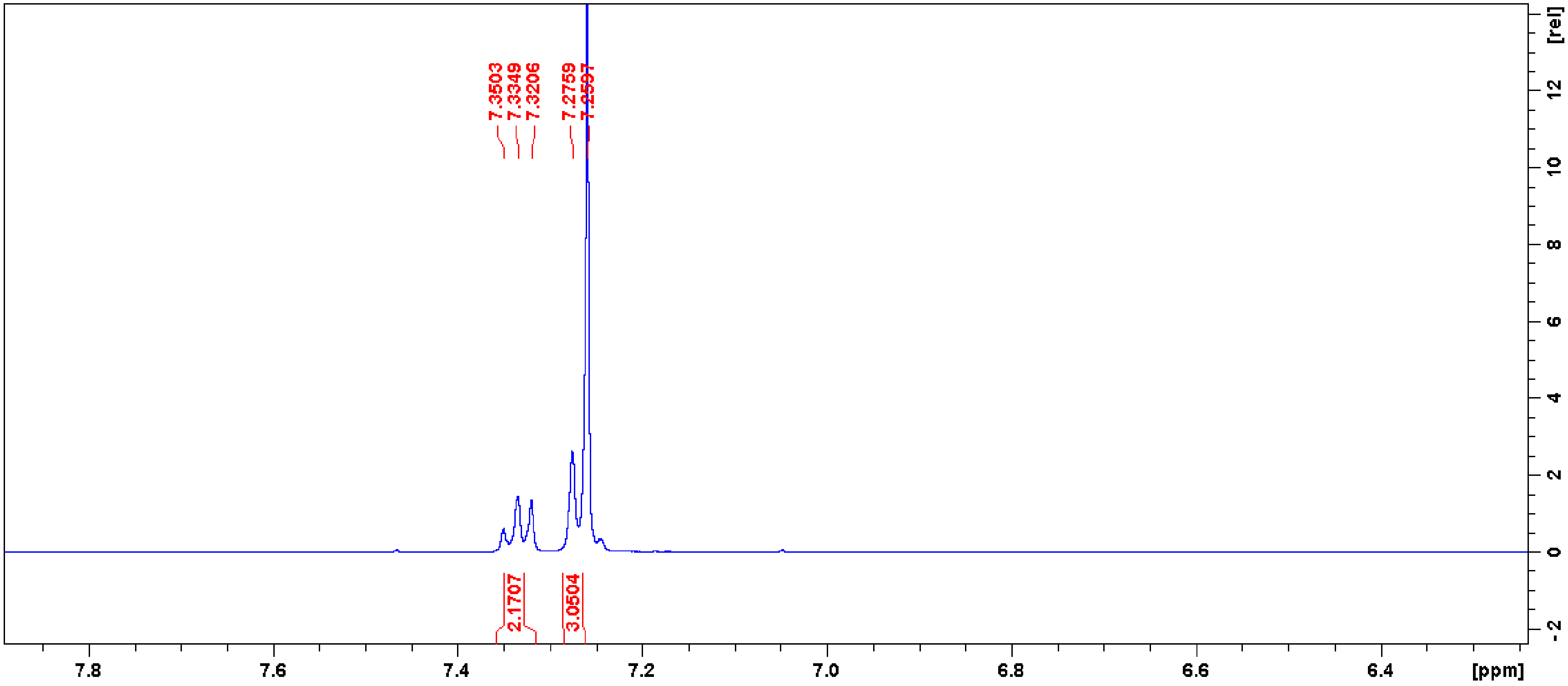
7.3503
7.3349
7.3206
7.2759
7.2597
2.1707
3.0504
7.8
7.6
7.4
7.2
7.0
6.8
6.6
6.4
[ppm]
[rel]
12
10
8
6
4
2
0
-2

^{13}C-NMR of compound (4*R*,4a*S*,6a*R*,8*R*,9*R*,11a*R*,11b*S*)-methyl 9-(hydroxymethyl)-4,11b-dimethyl-8-(((*R*)-1-phenylpropyl)amino)tetradecahydro-6a,9-methanocyclohepta[a]naphthalene-4-carboxylate **(12)**

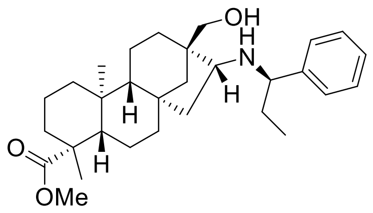

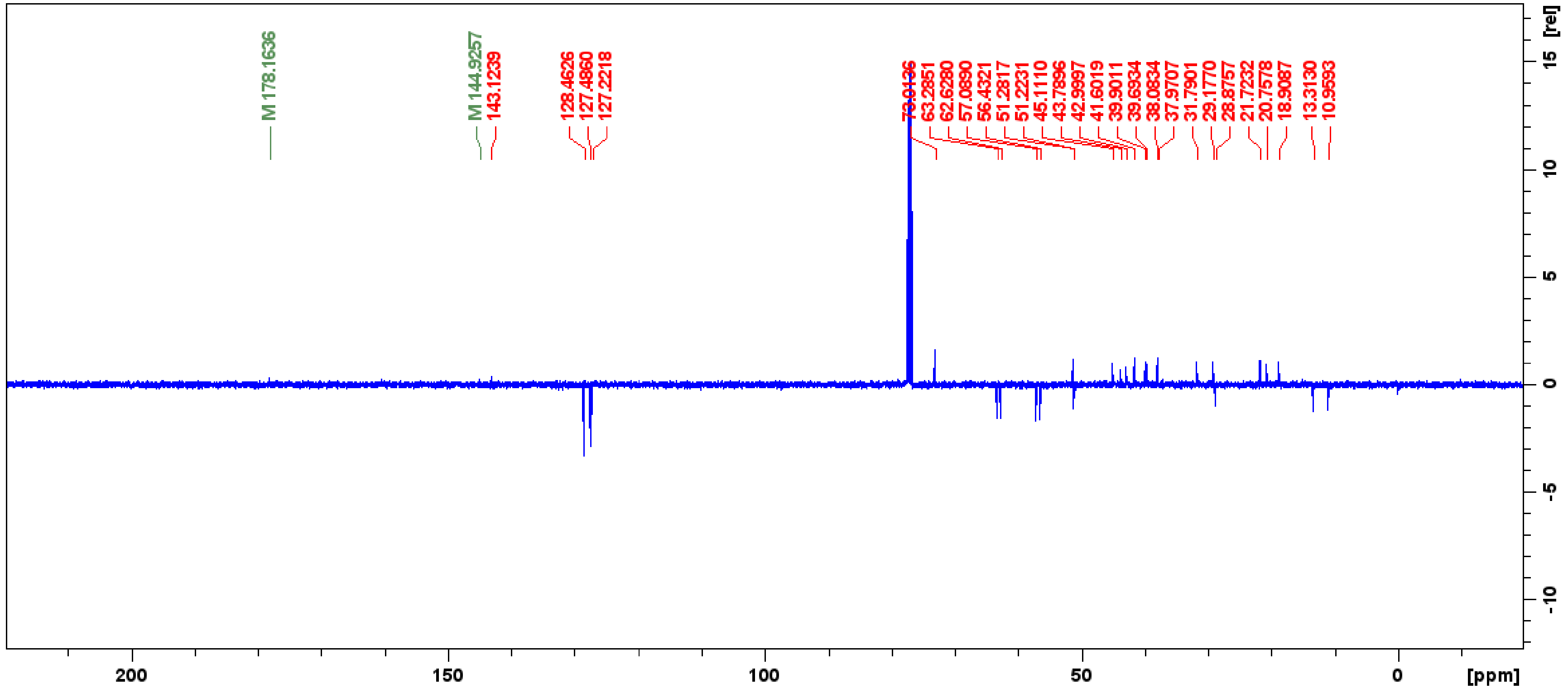

COSY of compound (4*R*,4a*S*,6a*R*,8*R*,9*R*,11a*R*,11b*S*)-methyl 9-(hydroxymethyl)-4,11b-dimethyl-8-(((*R*)-1-phenylpropyl)amino)tetradecahydro-6a,9-methanocyclohepta[a]naphthalene-4-carboxylate **(12)**

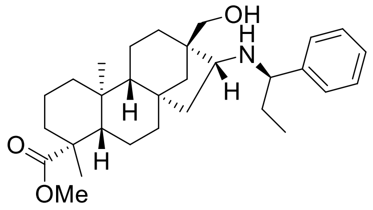

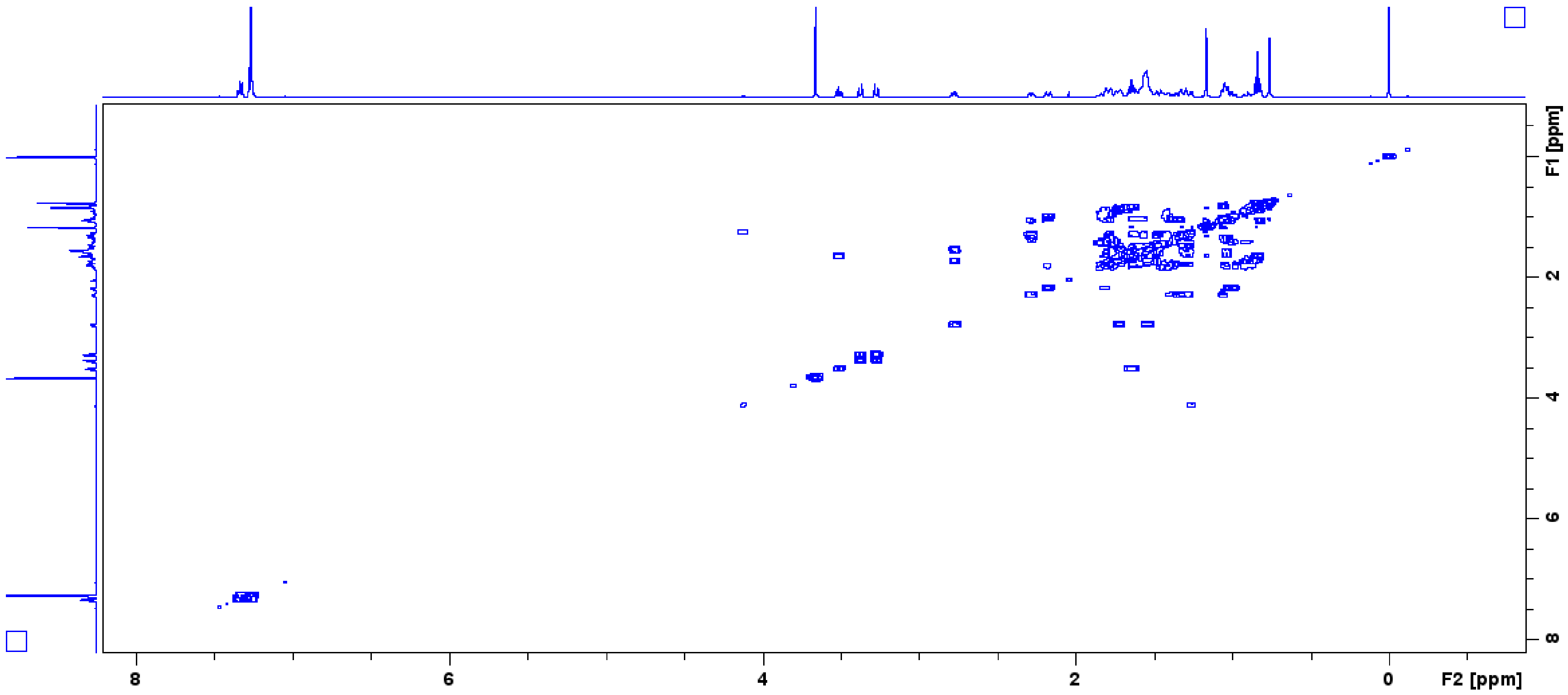

NOESY of compound (4*R*,4a*S*,6a*R*,8*R*,9*R*,11a*R*,11b*S*)-methyl 9-(hydroxymethyl)-4,11b-dimethyl-8-(((*R*)-1-phenylpropyl)amino)tetradecahydro-6a,9-methanocyclohepta[a]naphthalene-4-carboxylate **(12)**

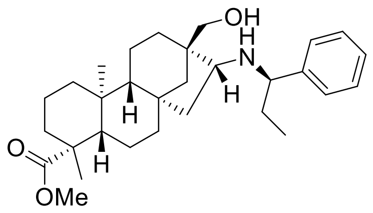

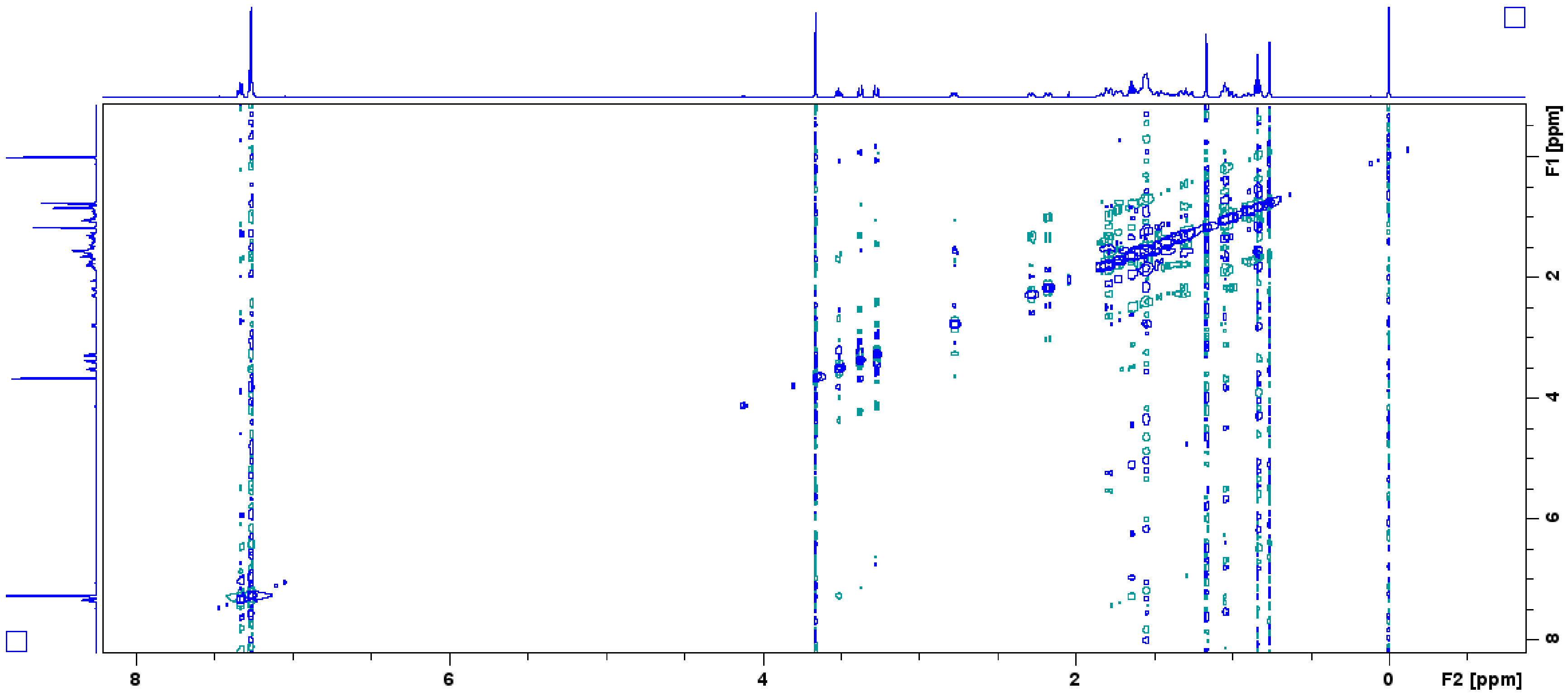

HSQC of compound (4*R*,4a*S*,6a*R*,8*R*,9*R*,11a*R*,11b*S*)-methyl 9-(hydroxymethyl)-4,11b-dimethyl-8-(((*R*)-1-phenylpropyl)amino)tetradecahydro-6a,9-methanocyclohepta[a]naphthalene-4-carboxylate **(12)**

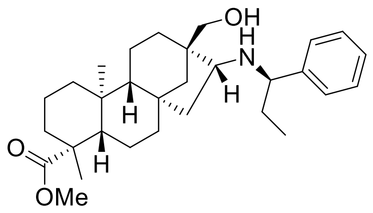

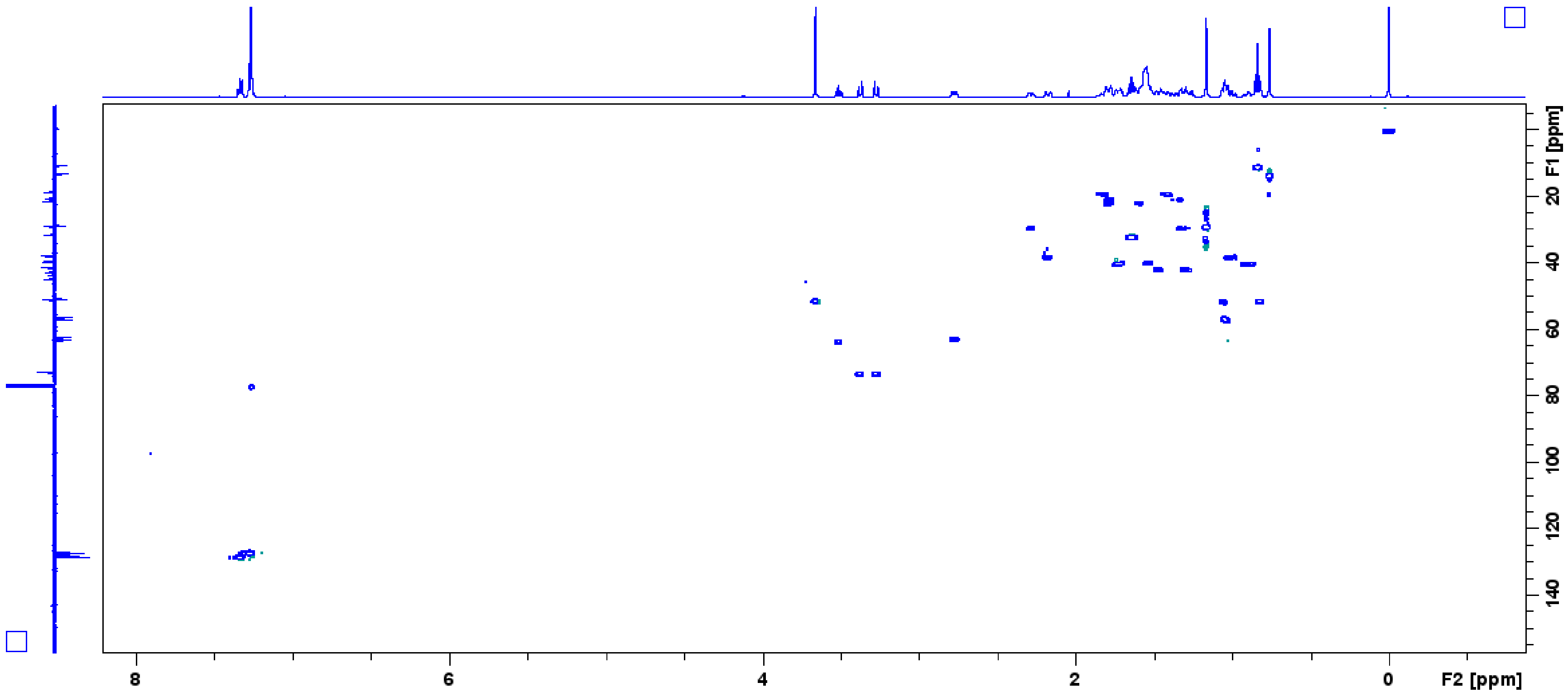

HMBC of compound (4*R*,4a*S*,6a*R*,8*R*,9*R*,11a*R*,11b*S*)-methyl 9-(hydroxymethyl)-4,11b-dimethyl-8-(((*R*)-1-phenylpropyl)amino)tetradecahydro-6a,9-methanocyclohepta[a]naphthalene-4-carboxylate **(12)**

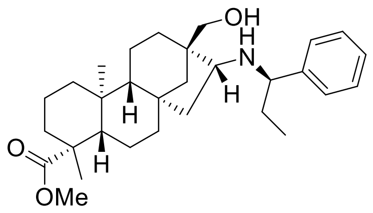

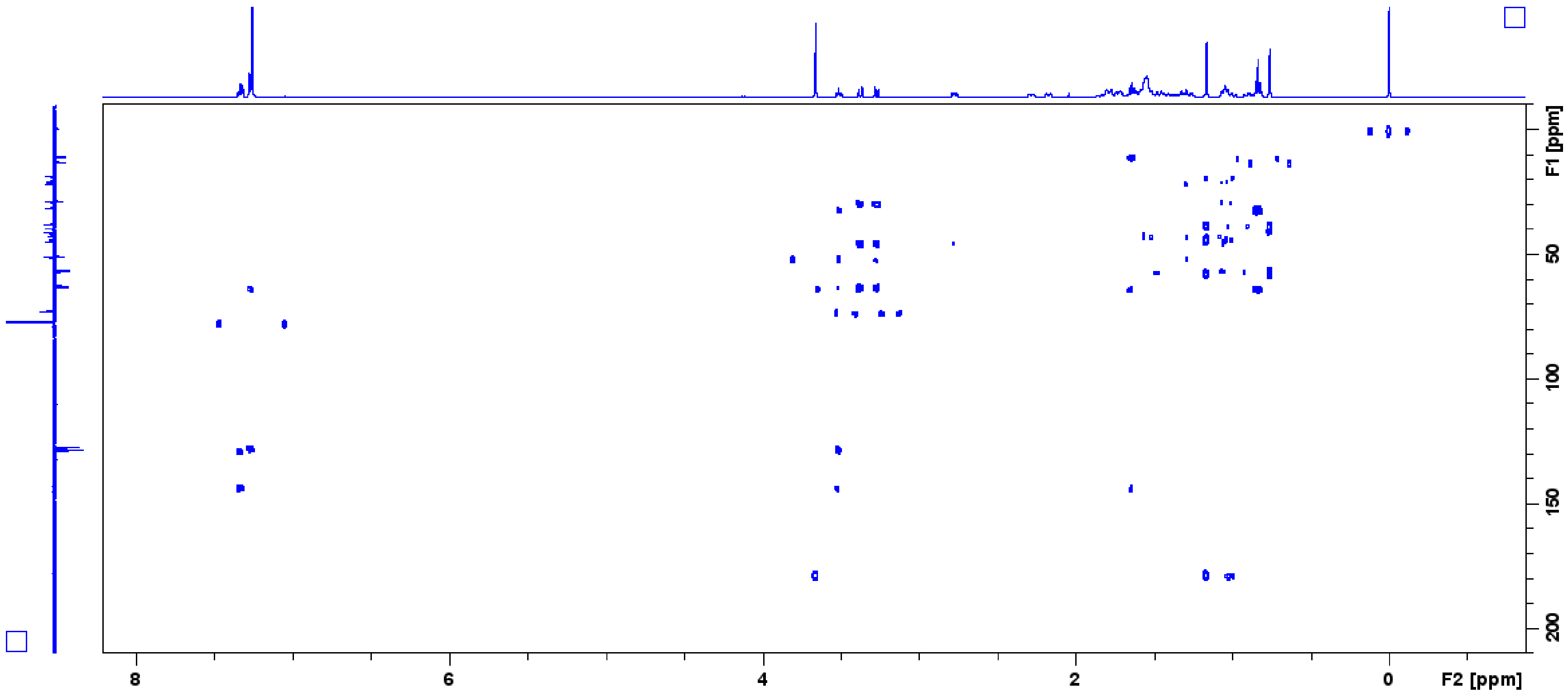

^{1}H-NMR of compound (4*R*,4a*S*,6a*R*,8*R*,9*R*,11a*R*,11b*S*)-methyl 9-(hydroxymethyl)-4,11b-dimethyl-8-(((*R*)-1-(naphthalen-1-yl)ethyl)amino)tetradecahydro-6a,9-methanocyclohepta[a]naphthalene-4-carboxylate **(13)**

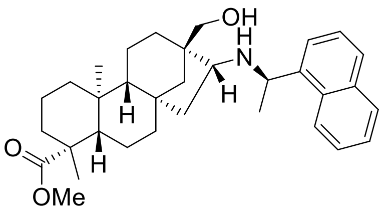

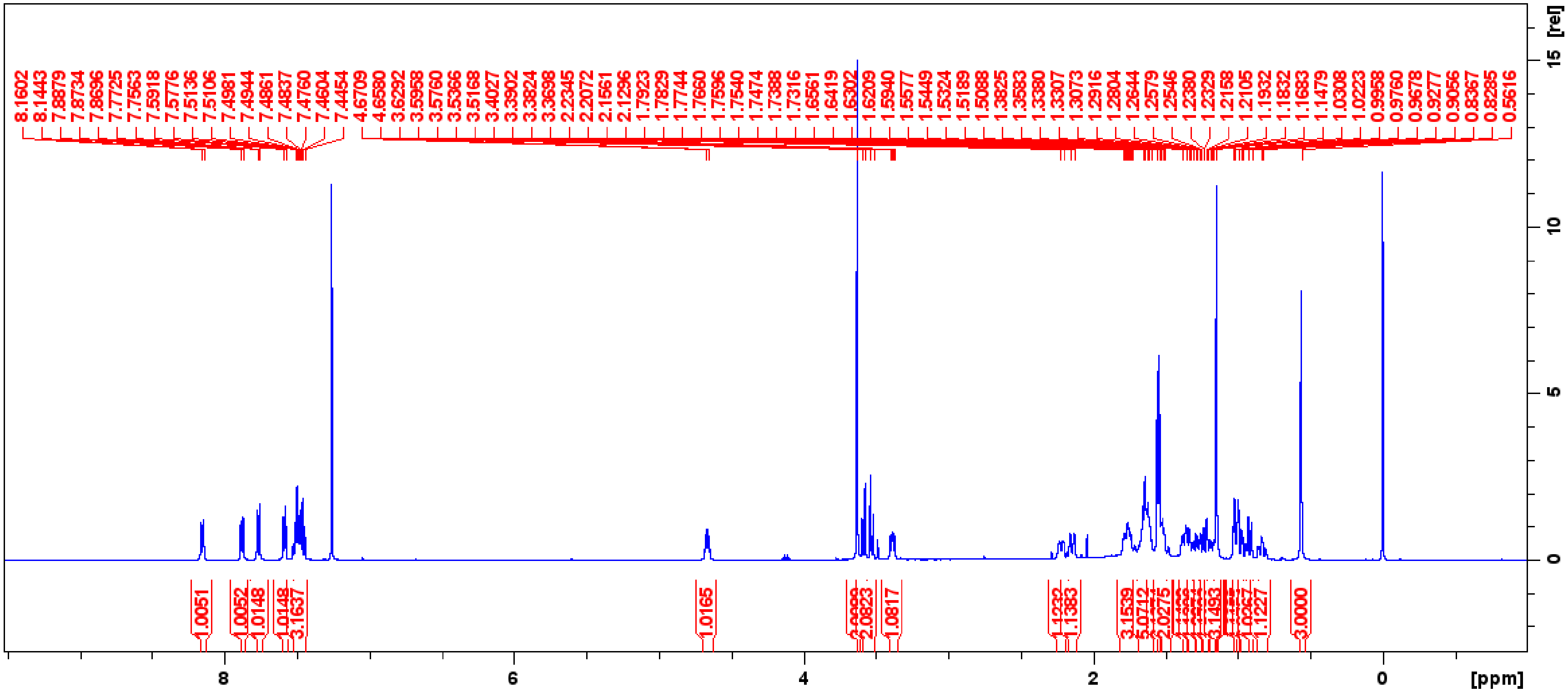

^{13}C-NMR of compound (4*R*,4a*S*,6a*R*,8*R*,9*R*,11a*R*,11b*S*)-methyl 9-(hydroxymethyl)-4,11b-dimethyl-8-(((*R*)-1-(naphthalen-1-yl)ethyl)amino)tetradecahydro-6a,9-methanocyclohepta[a]naphthalene-4-carboxylate **(13)**

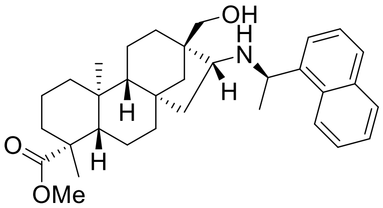

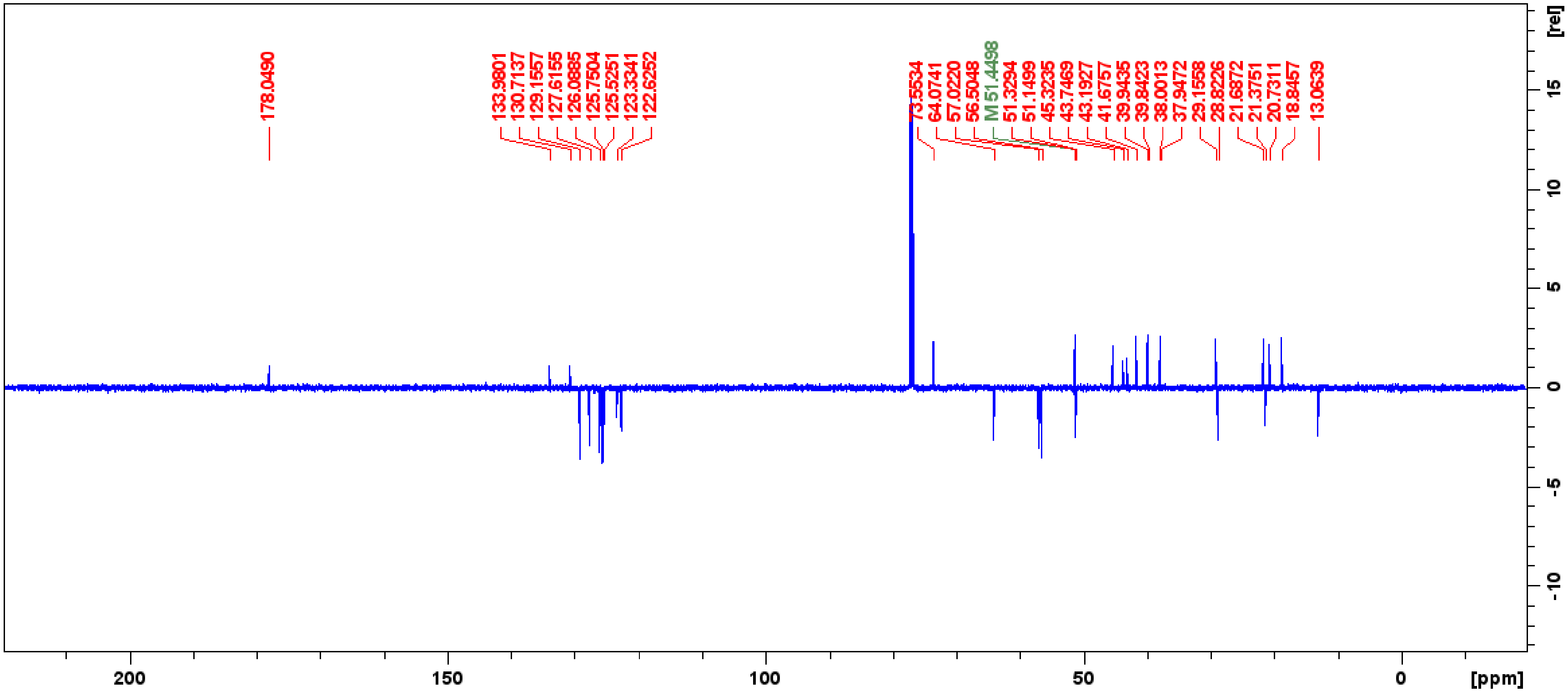

COSY of compound (4*R*,4a*S*,6a*R*,8*R*,9*R*,11a*R*,11b*S*)-methyl 9-(hydroxymethyl)-4,11b-dimethyl-8-(((*R*)-1-(naphthalen-1-yl)ethyl)amino)tetradecahydro-6a,9-methanocyclohepta[a]naphthalene-4-carboxylate **(13)**

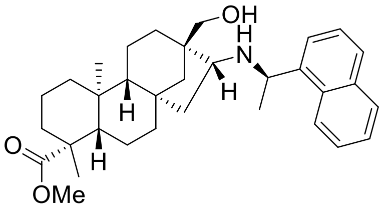

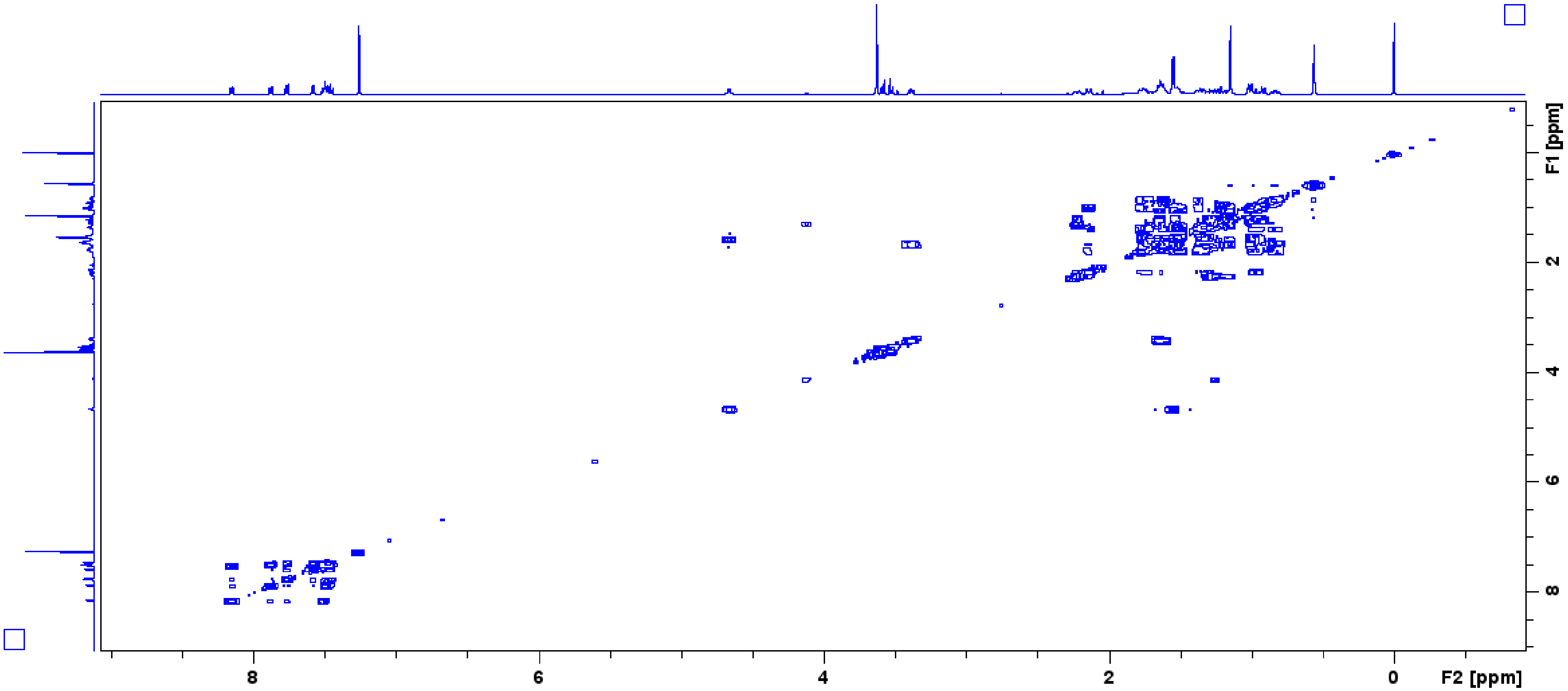

NOESY of compound (4*R*,4a*S*,6a*R*,8*R*,9*R*,11a*R*,11b*S*)-methyl 9-(hydroxymethyl)-4,11b-dimethyl-8-(((*R*)-1-(naphthalen-1-yl)ethyl)amino)tetradecahydro-6a,9-methanocyclohepta[a]naphthalene-4-carboxylate **(13)**

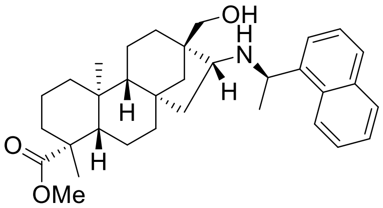

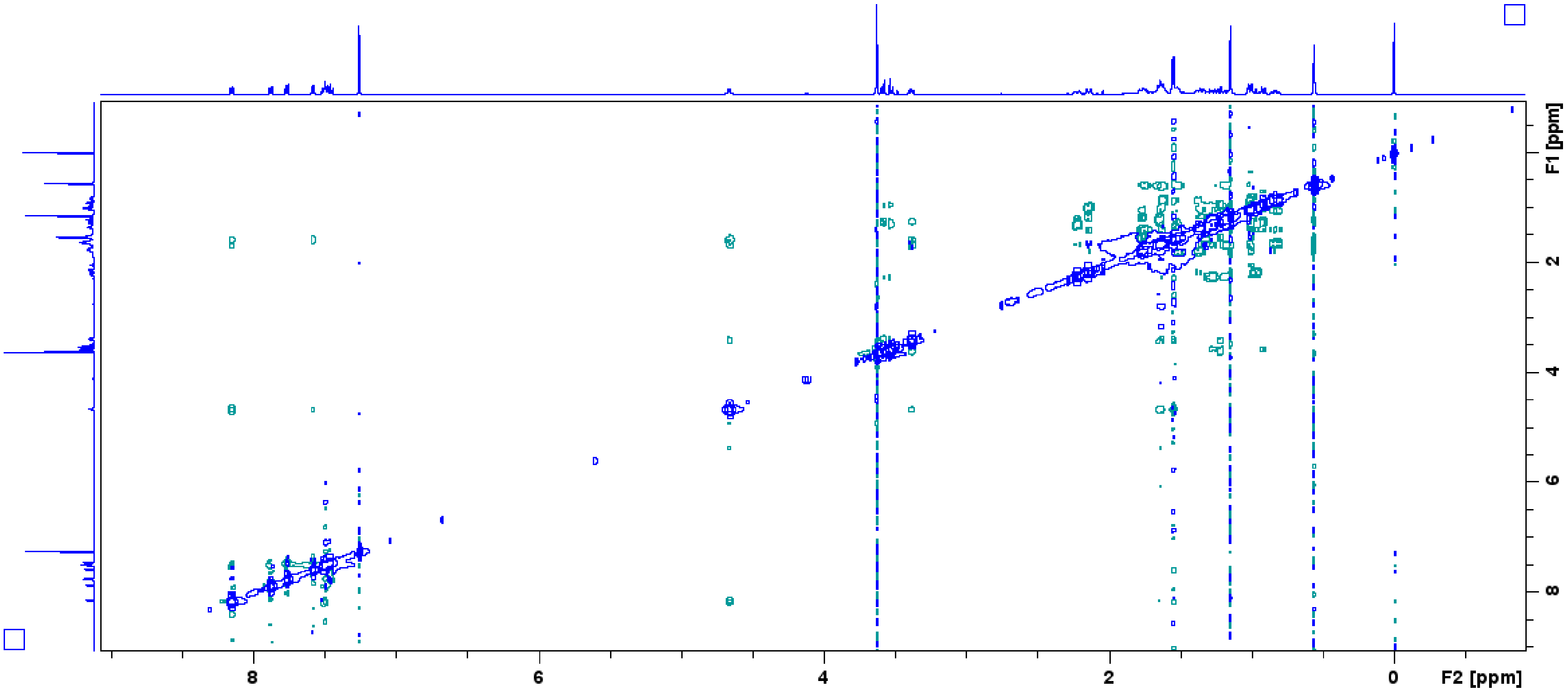

HSQC of compound (4*R*,4a*S*,6a*R*,8*R*,9*R*,11a*R*,11b*S*)-methyl 9-(hydroxymethyl)-4,11b-dimethyl-8-(((*R*)-1-(naphthalen-1-yl)ethyl)amino)tetradecahydro-6a,9-methanocyclohepta[a]naphthalene-4-carboxylate **(13)**

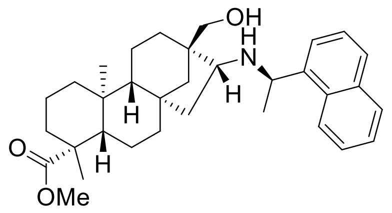

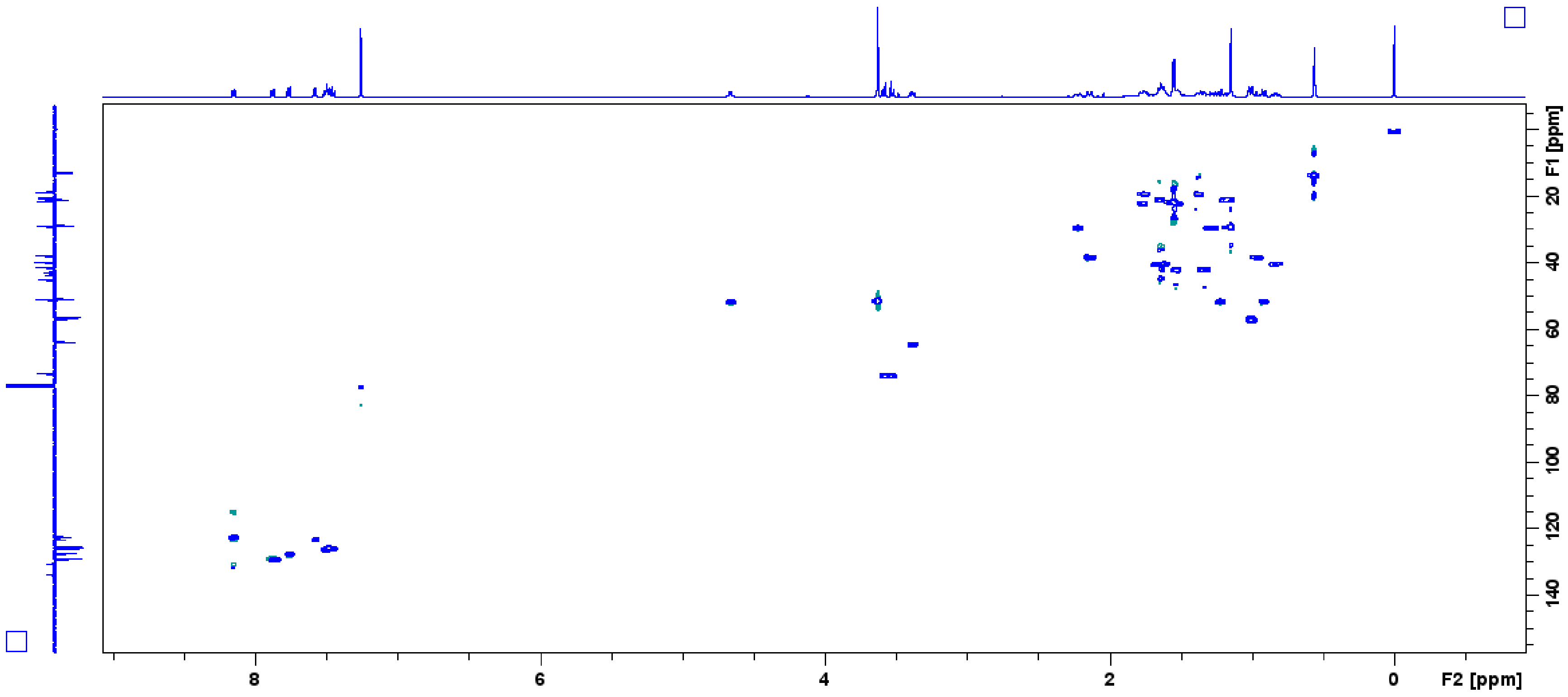

HMBC of compound (4*R*,4a*S*,6a*R*,8*R*,9*R*,11a*R*,11b*S*)-methyl 9-(hydroxymethyl)-4,11b-dimethyl-8-(((*R*)-1-(naphthalen-1-yl)ethyl)amino)tetradecahydro-6a,9-methanocyclohepta[a]naphthalene-4-carboxylate **(13)**

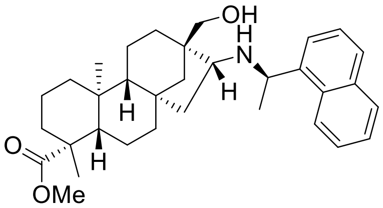

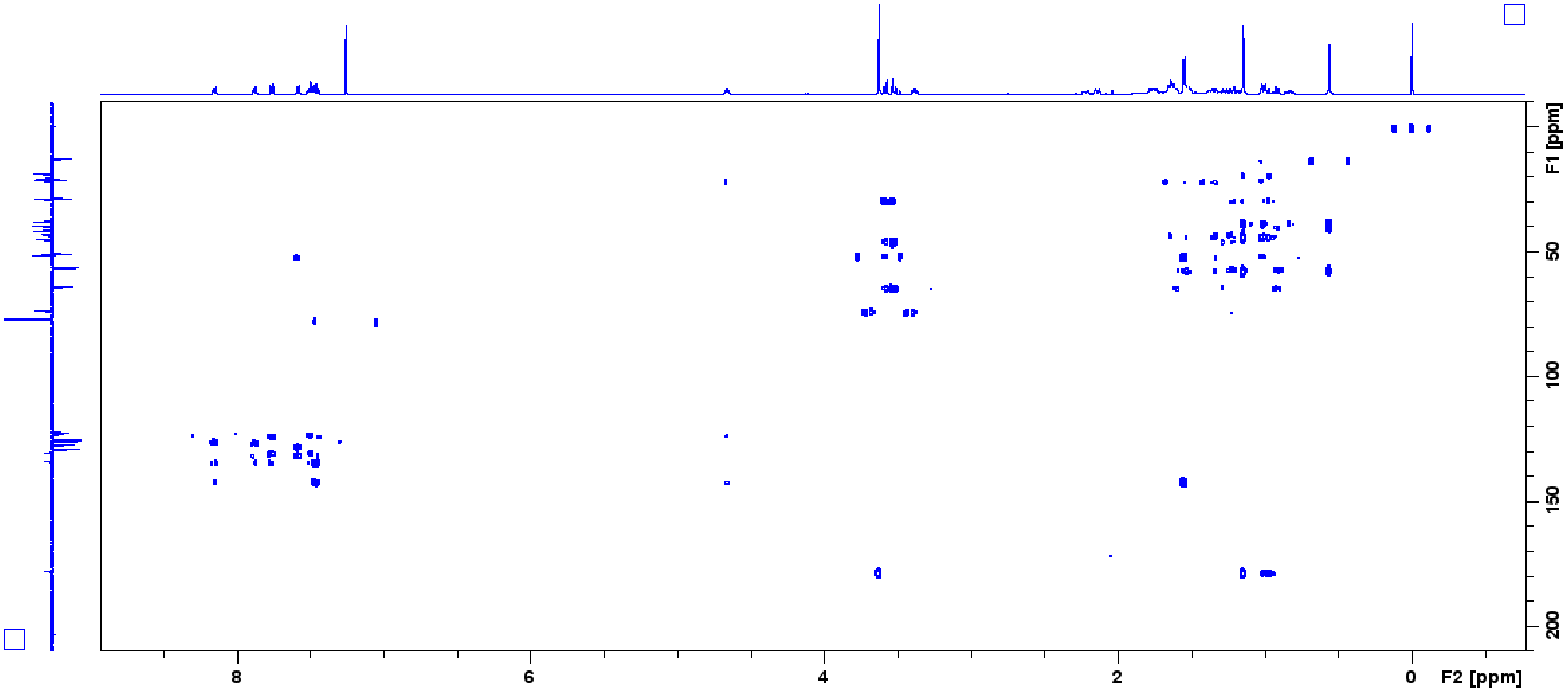

^{1}H-NMR of compound (4*R*,4a*S*,6a*R*,8*R*,9*R*,11a*R*,11b*S*)-methyl 9-(hydroxymethyl)-4,11b-dimethyl-8-(((*S*)-1-(naphthalen-1-yl)ethyl)amino)tetradecahydro-6a,9-methanocyclohepta[a]naphthalene-4-carboxylate **(14)**

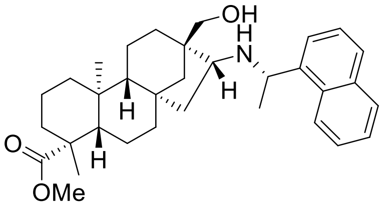

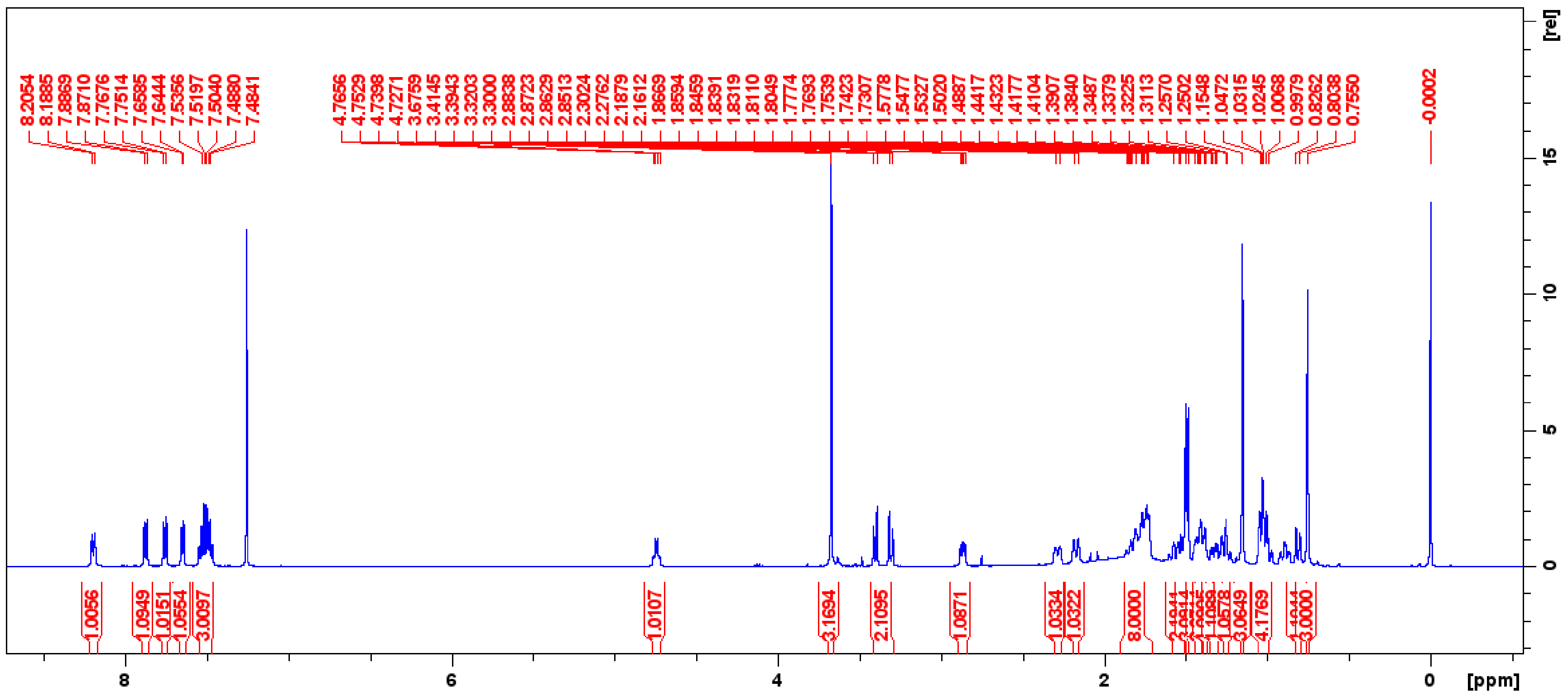

^{13}C-NMR of compound (4*R*,4a*S*,6a*R*,8*R*,9*R*,11a*R*,11b*S*)-methyl 9-(hydroxymethyl)-4,11b-dimethyl-8-(((*S*)-1-(naphthalen-1-yl)ethyl)amino)tetradecahydro-6a,9-methanocyclohepta[a]naphthalene-4-carboxylate **(14)**

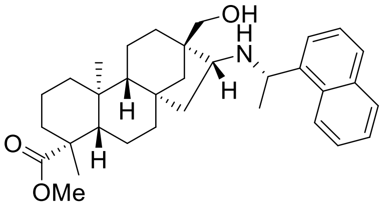

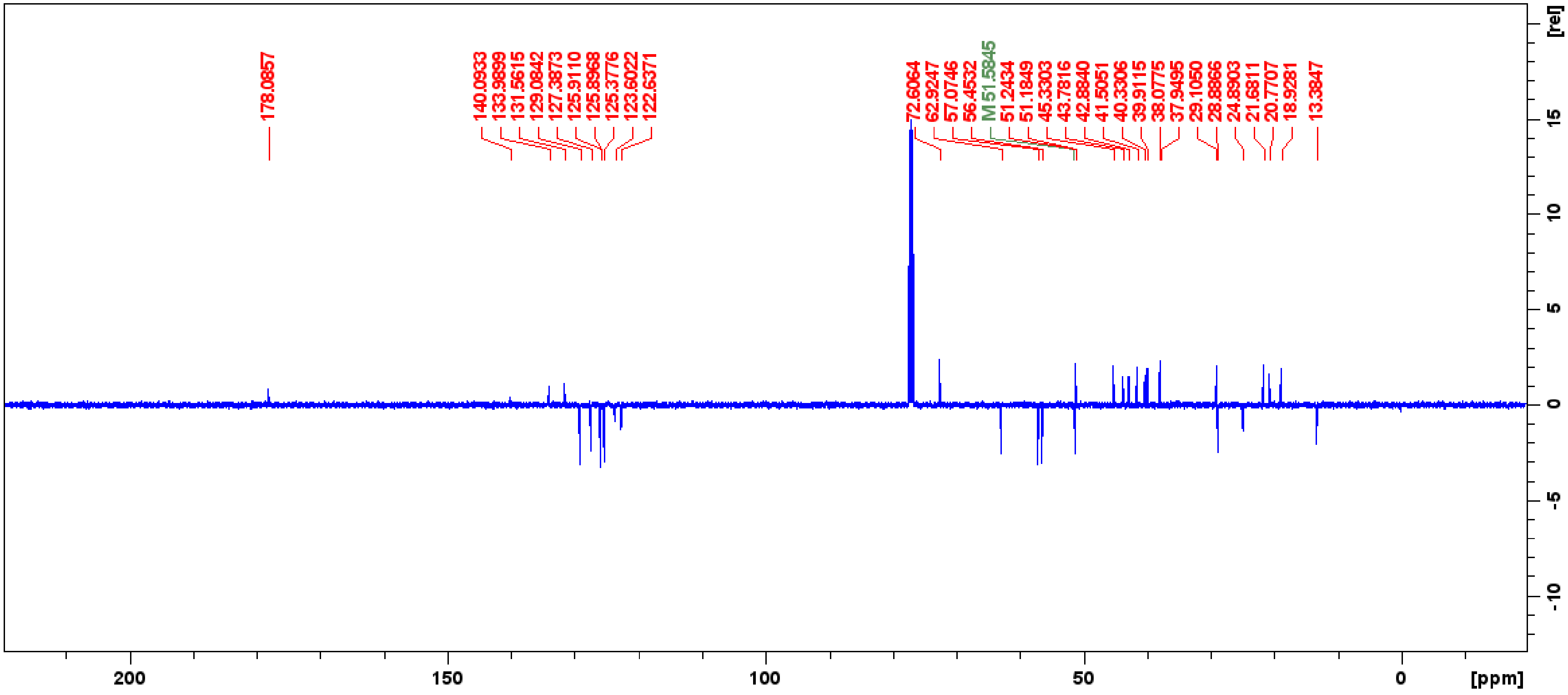

COSY of compound (4*R*,4a*S*,6a*R*,8*R*,9*R*,11a*R*,11b*S*)-methyl 9-(hydroxymethyl)-4,11b-dimethyl-8-(((*S*)-1-(naphthalen-1-yl)ethyl)amino)tetradecahydro-6a,9-methanocyclohepta[a]naphthalene-4-carboxylate **(14)**

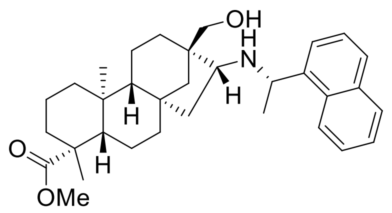

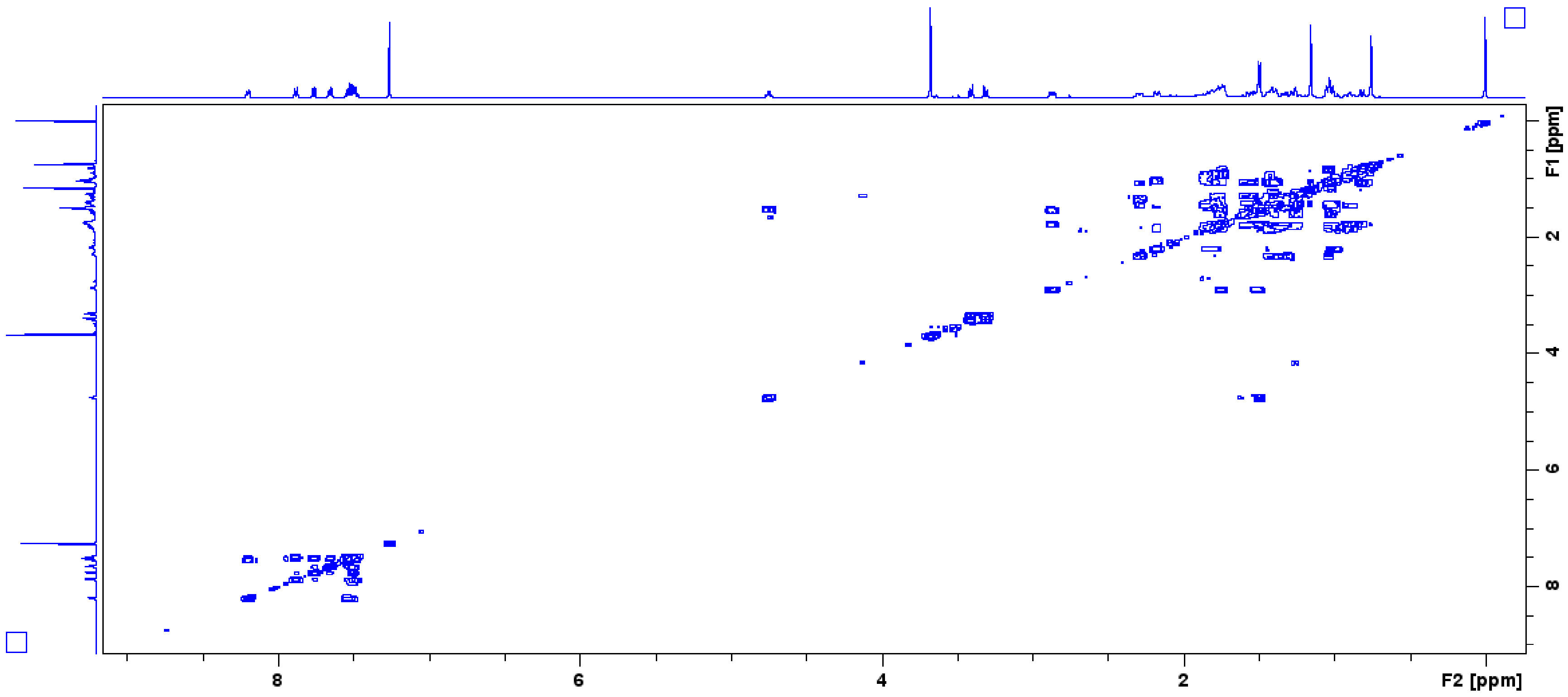

NOESY of compound (4*R*,4a*S*,6a*R*,8*R*,9*R*,11a*R*,11b*S*)-methyl 9-(hydroxymethyl)-4,11b-dimethyl-8-(((*S*)-1-(naphthalen-1-yl)ethyl)amino)tetradecahydro-6a,9-methanocyclohepta[a]naphthalene-4-carboxylate **(14)**

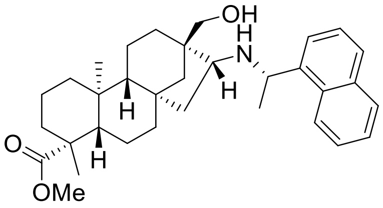

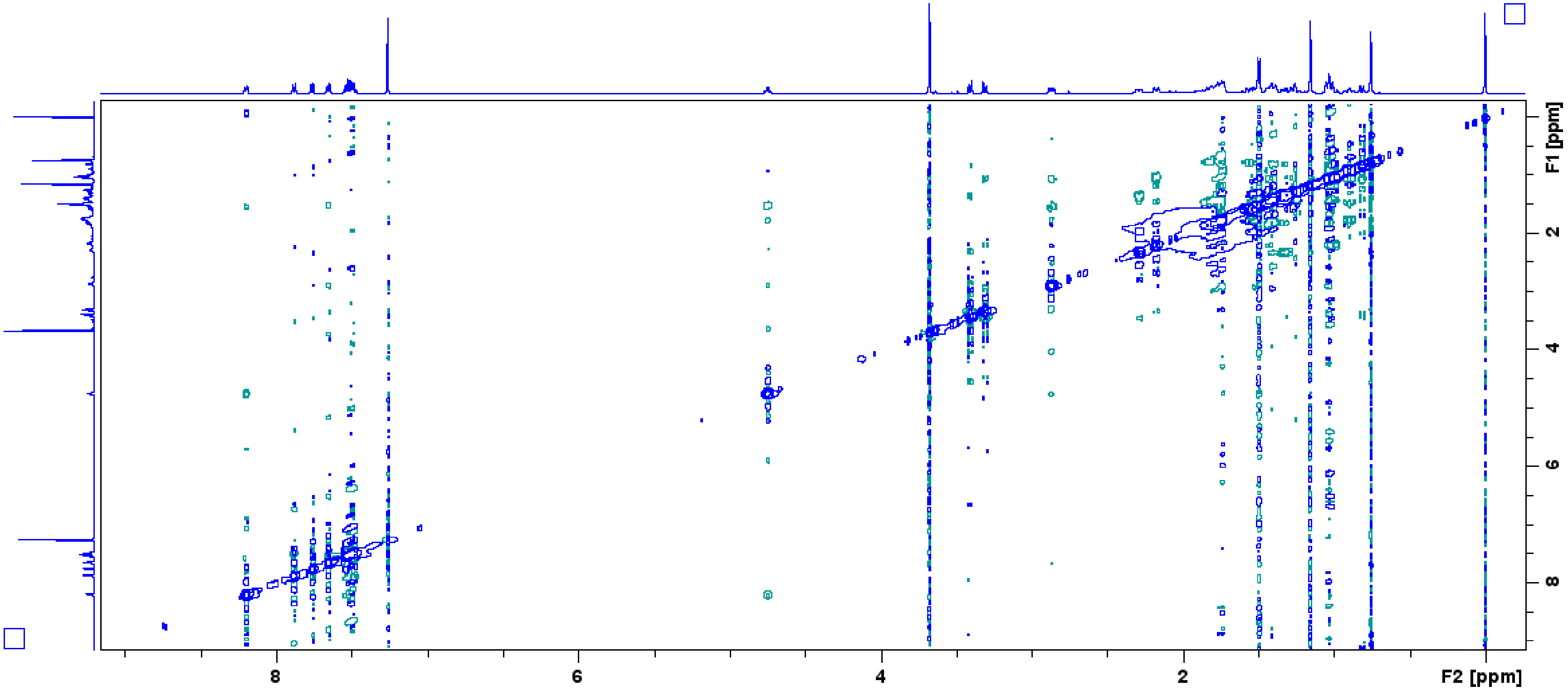

HSQC of compound (4*R*,4a*S*,6a*R*,8*R*,9*R*,11a*R*,11b*S*)-methyl 9-(hydroxymethyl)-4,11b-dimethyl-8-(((*S*)-1-(naphthalen-1-yl)ethyl)amino)tetradecahydro-6a,9-methanocyclohepta[a]naphthalene-4-carboxylate **(14)**

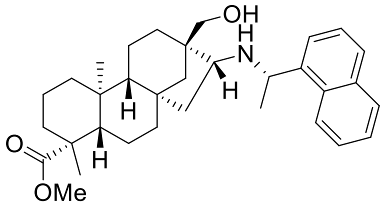

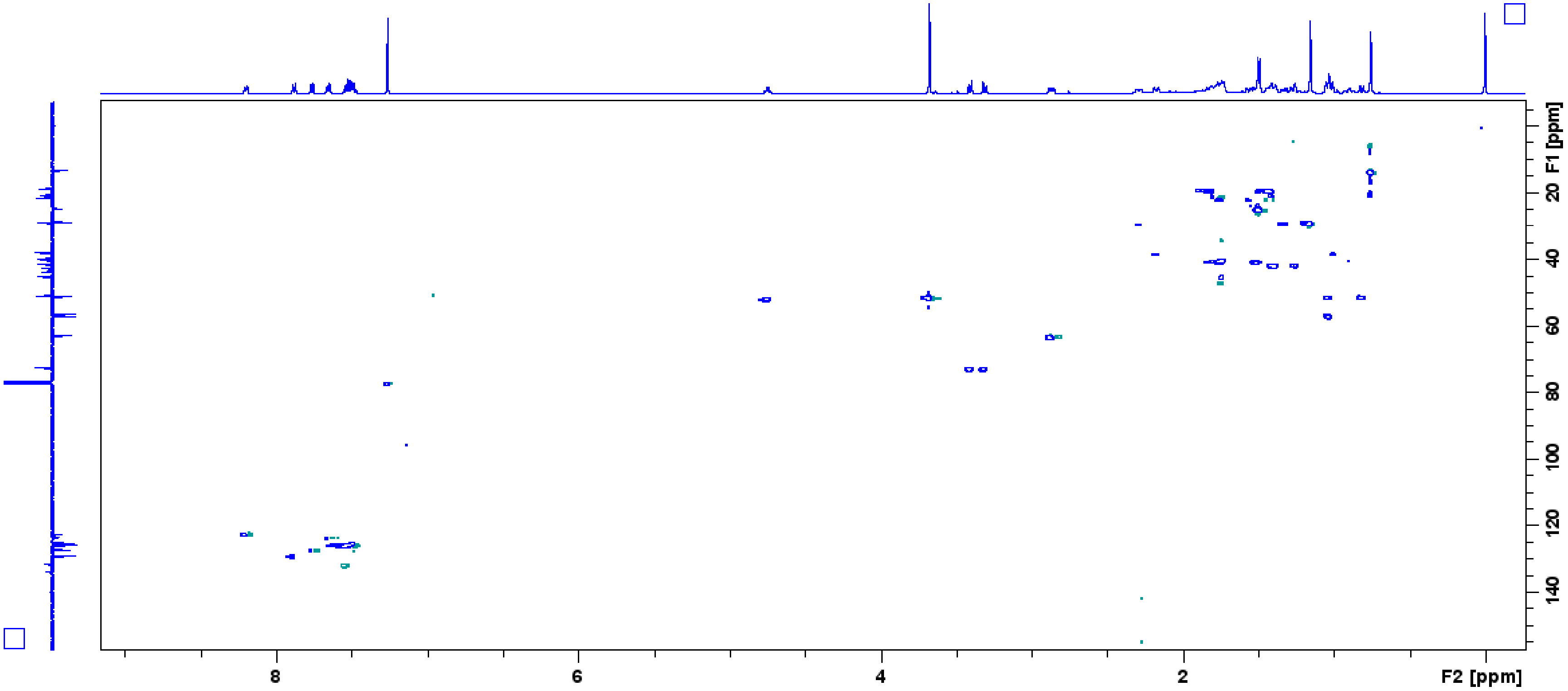

HMBC of compound (4*R*,4a*S*,6a*R*,8*R*,9*R*,11a*R*,11b*S*)-methyl 9-(hydroxymethyl)-4,11b-dimethyl-8-(((*S*)-1-(naphthalen-1-yl)ethyl)amino)tetradecahydro-6a,9-methanocyclohepta[a]naphthalene-4-carboxylate **(14)**

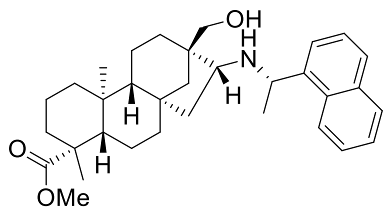

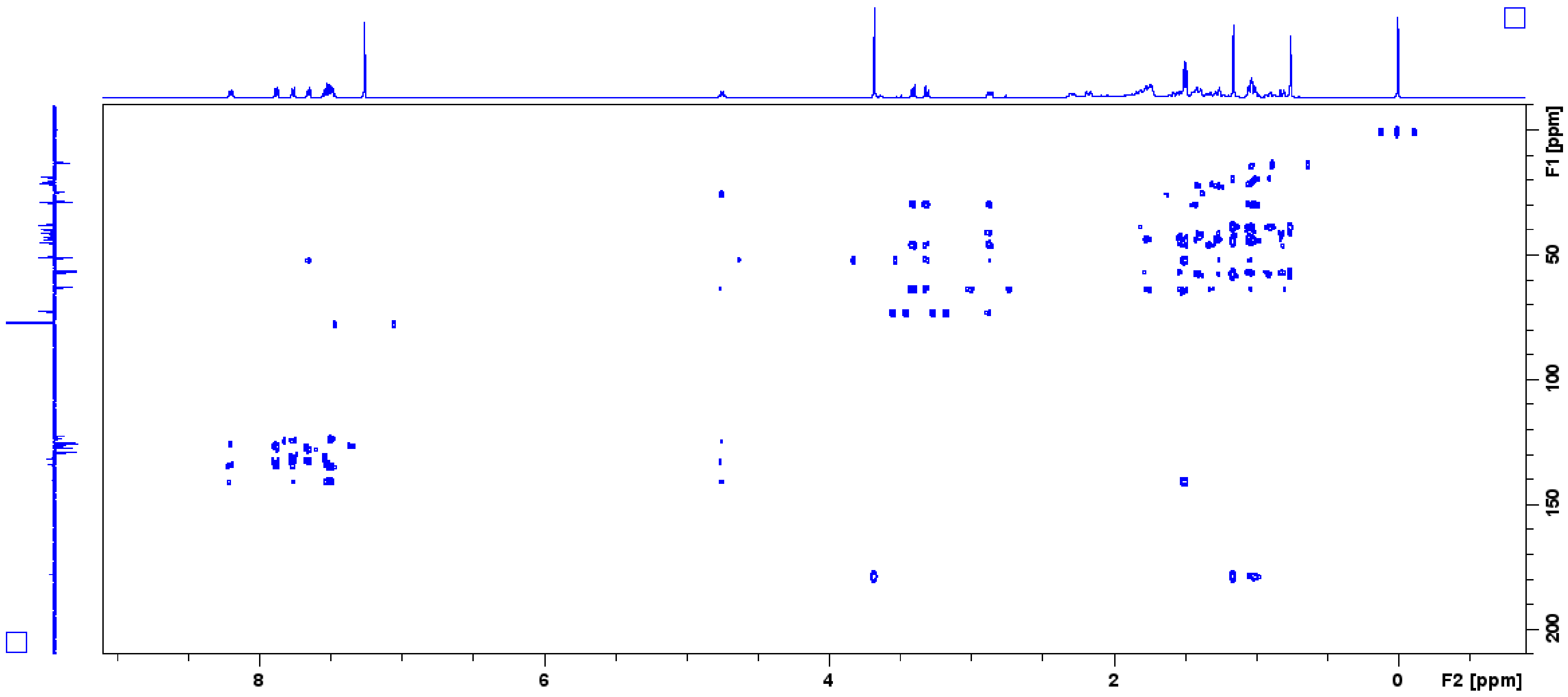

^{1}H-NMR of compound (4*R*,4a*S*,6a*R*,8*R*,9*R*,11a*R*,11b*S*)-methyl 9-(hydroxymethyl)-4,11b-dimethyl-8-((naphthalen-1-ylmethyl)amino)tetradecahydro-6a,9-methanocyclohepta[a]naphthalene-4-carboxylate **(15)**

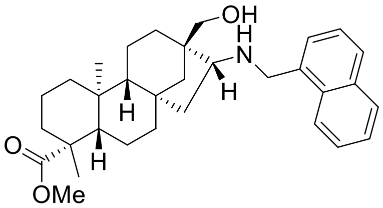

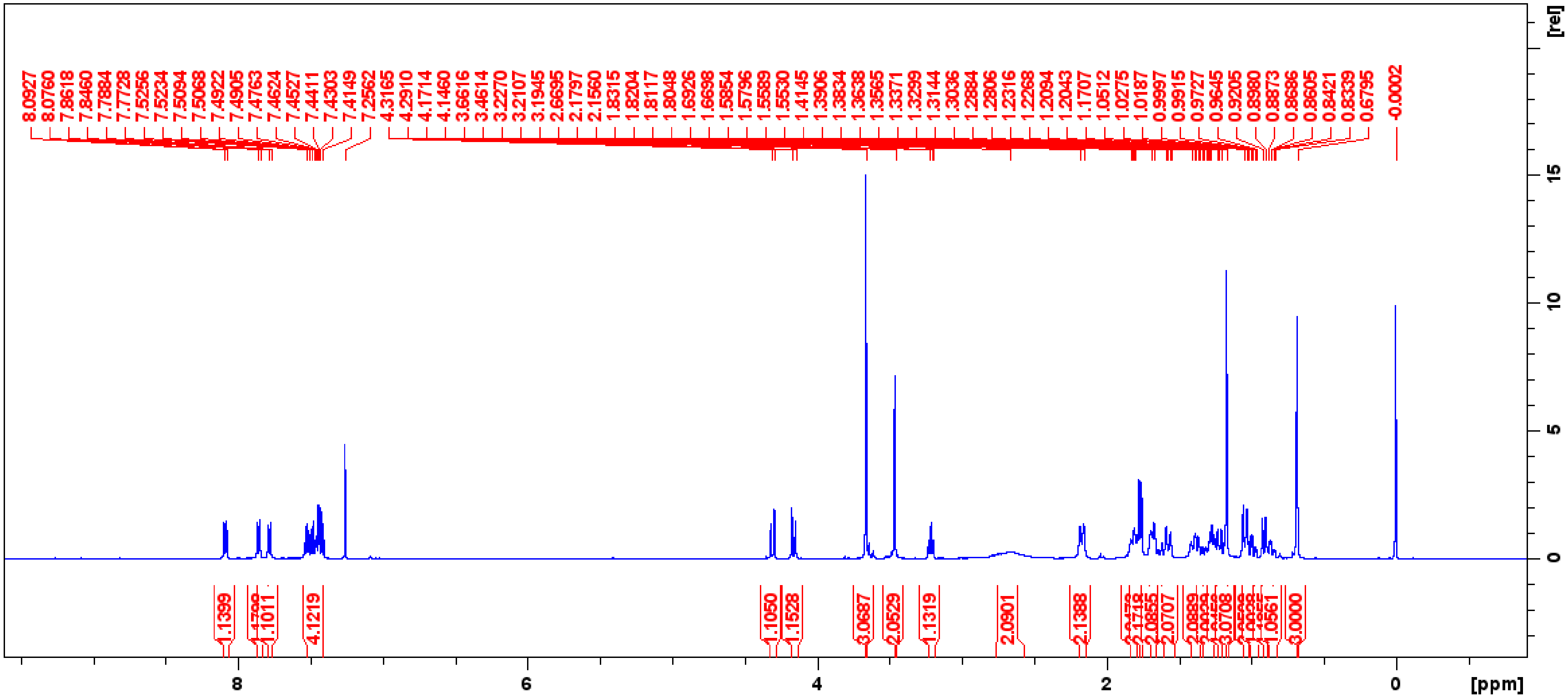

^{13}C-NMR of compound (4*R*,4a*S*,6a*R*,8*R*,9*R*,11a*R*,11b*S*)-methyl 9-(hydroxymethyl)-4,11b-dimethyl-8-((naphthalen-1-ylmethyl)amino)tetradecahydro-6a,9-methanocyclohepta[a]naphthalene-4-carboxylate **(15)**

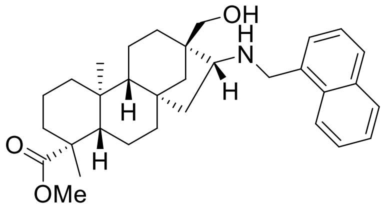

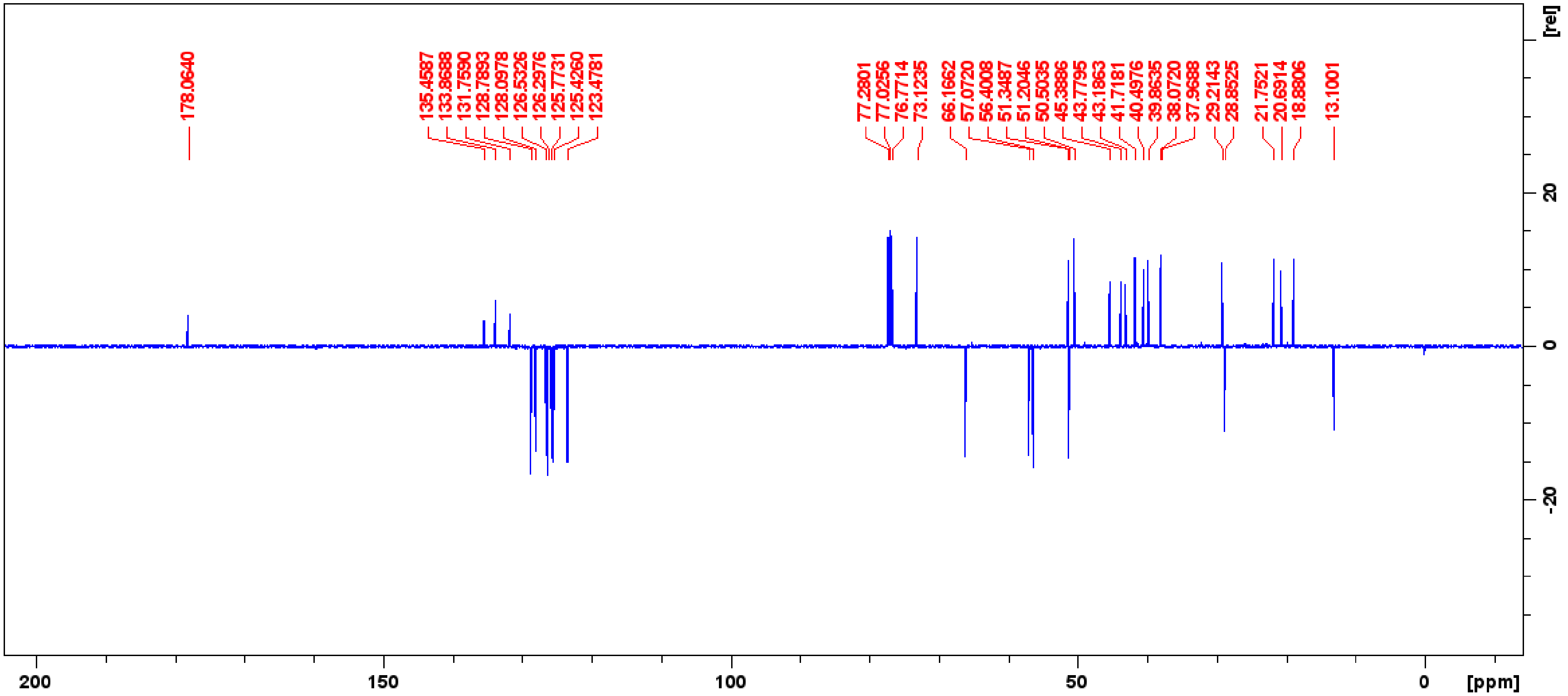

COSY of compound (4*R*,4a*S*,6a*R*,8*R*,9*R*,11a*R*,11b*S*)-methyl 9-(hydroxymethyl)-4,11b-dimethyl-8-((naphthalen-1-ylmethyl)amino)tetradecahydro-6a,9-methanocyclohepta[a]naphthalene-4-carboxylate **(15)**

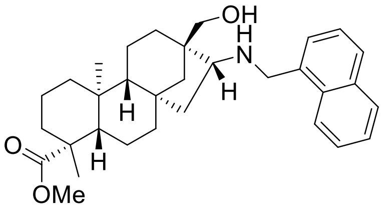

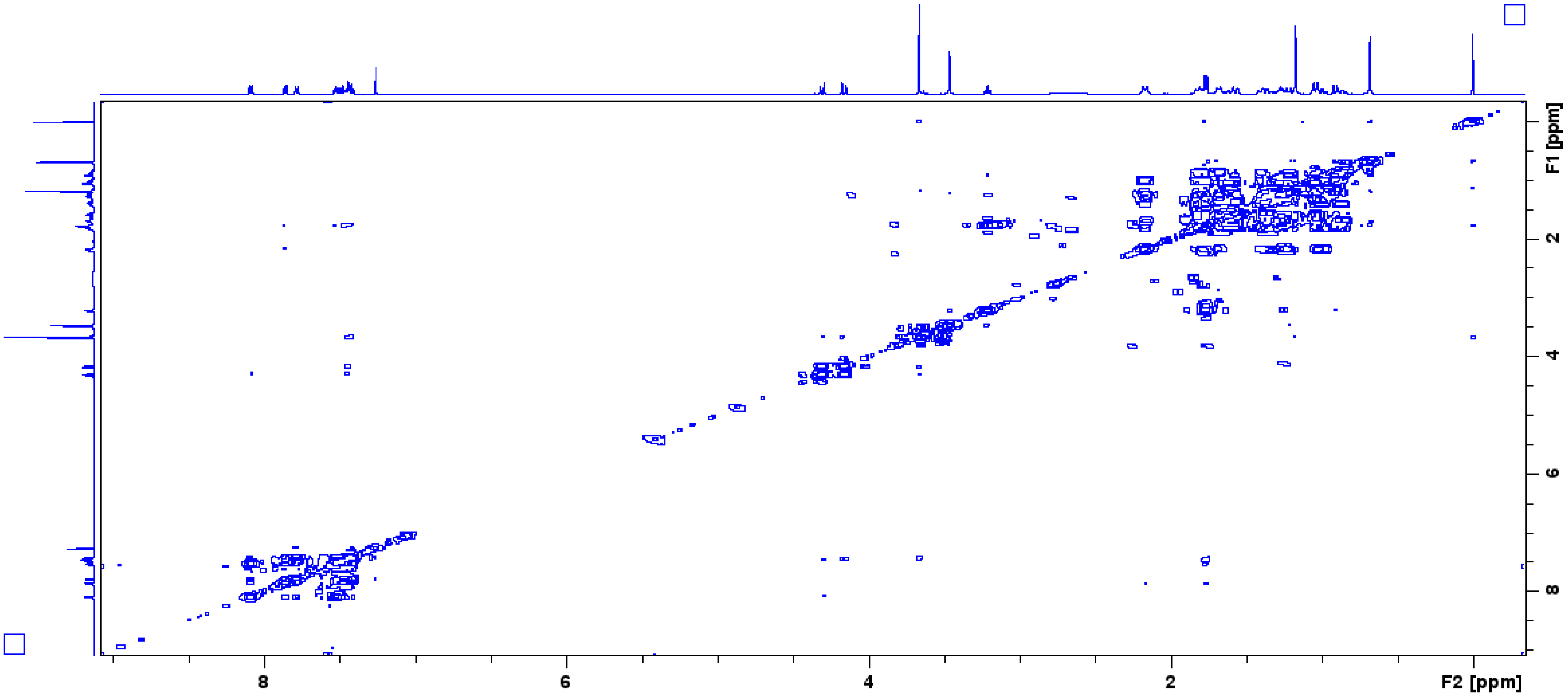

NOESY of compound (4*R*,4a*S*,6a*R*,8*R*,9*R*,11a*R*,11b*S*)-methyl 9-(hydroxymethyl)-4,11b-dimethyl-8-((naphthalen-1-ylmethyl)amino)tetradecahydro-6a,9-methanocyclohepta[a]naphthalene-4-carboxylate **(15)**

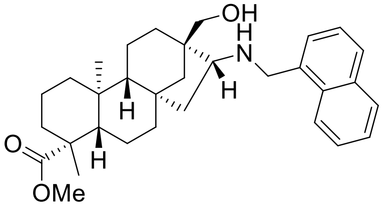

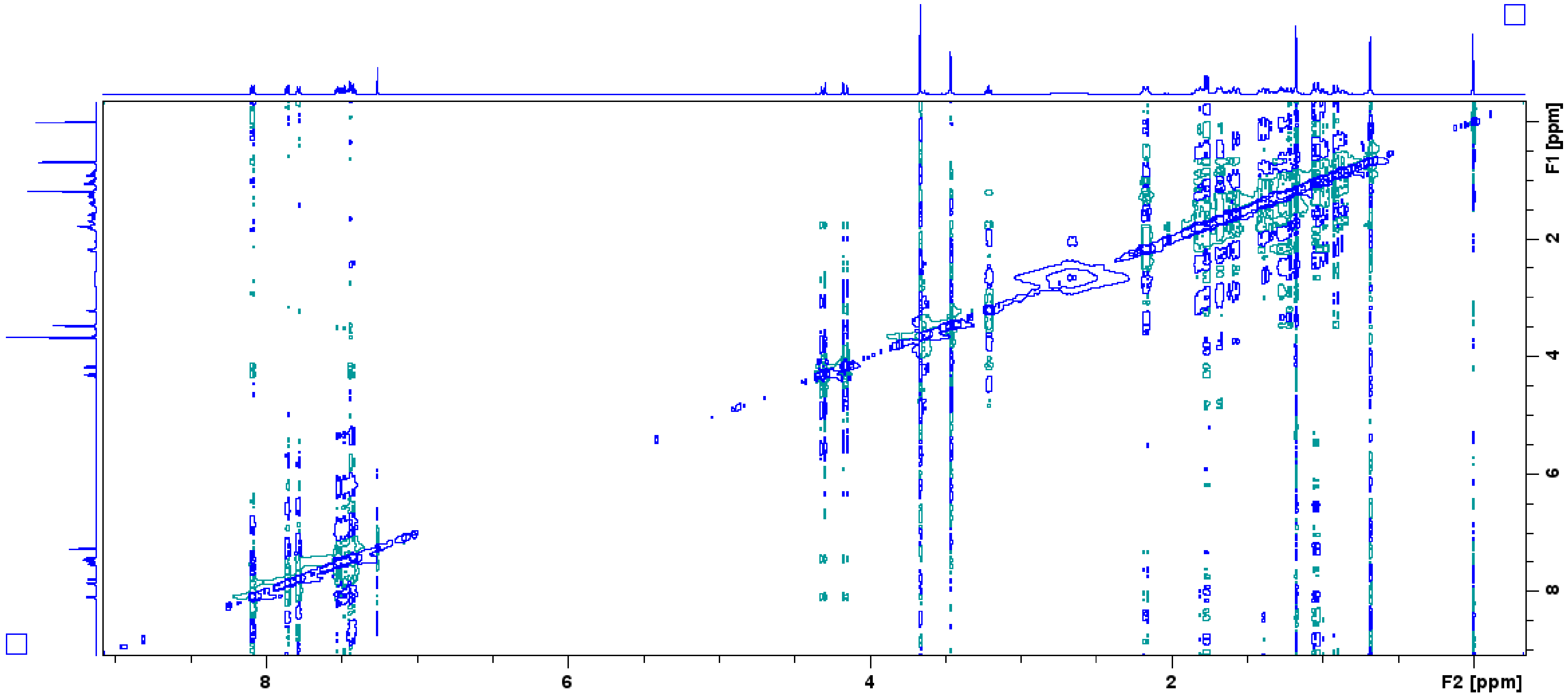

HSQC of compound (4*R*,4a*S*,6a*R*,8*R*,9*R*,11a*R*,11b*S*)-methyl 9-(hydroxymethyl)-4,11b-dimethyl-8-((naphthalen-1-ylmethyl)amino)tetradecahydro-6a,9-methanocyclohepta[a]naphthalene-4-carboxylate **(15)**

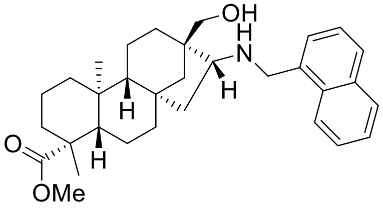

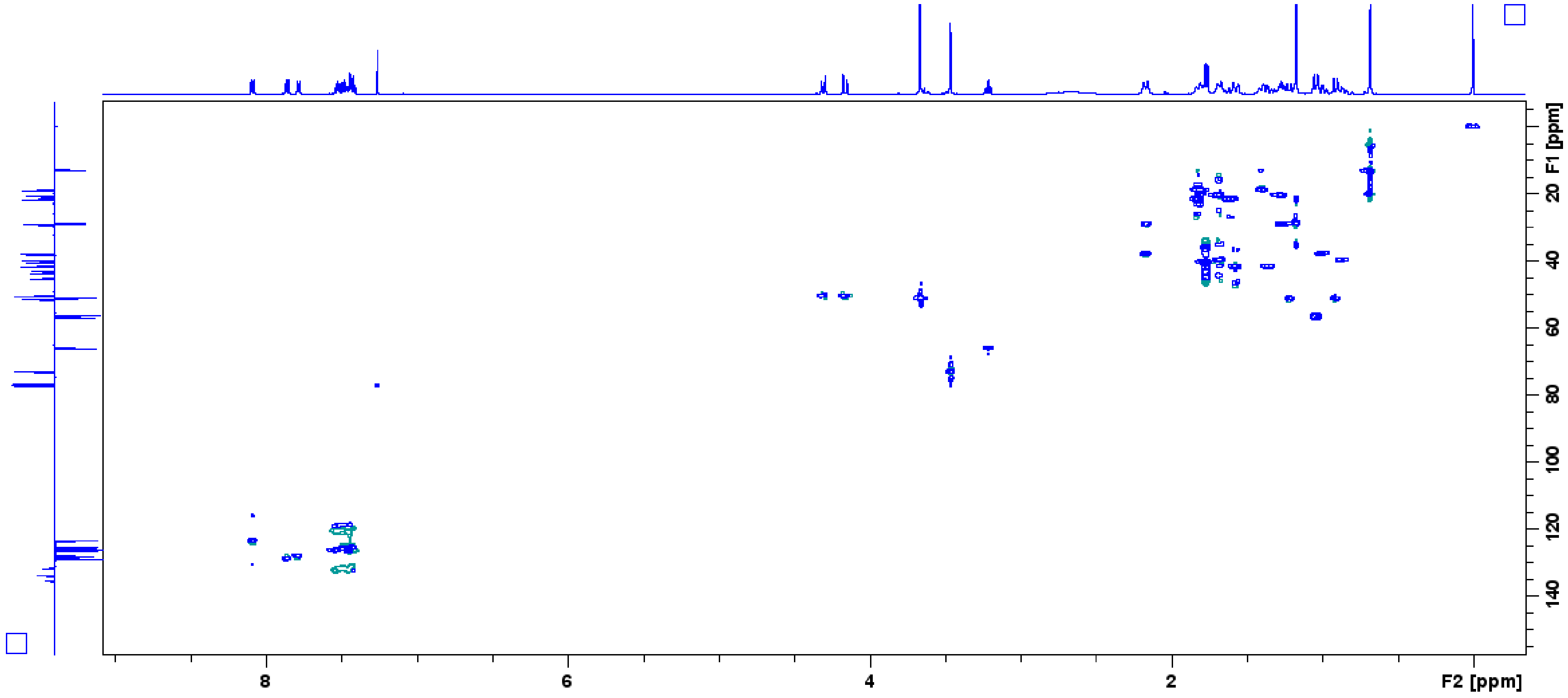

HMBC of compound (4*R*,4a*S*,6a*R*,8*R*,9*R*,11a*R*,11b*S*)-methyl 9-(hydroxymethyl)-4,11b-dimethyl-8-((naphthalen-1-ylmethyl)amino)tetradecahydro-6a,9-methanocyclohepta[a]naphthalene-4-carboxylate **(15)**

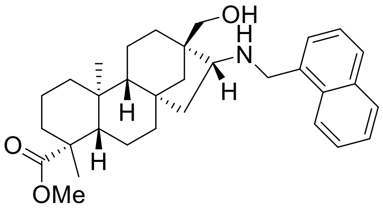

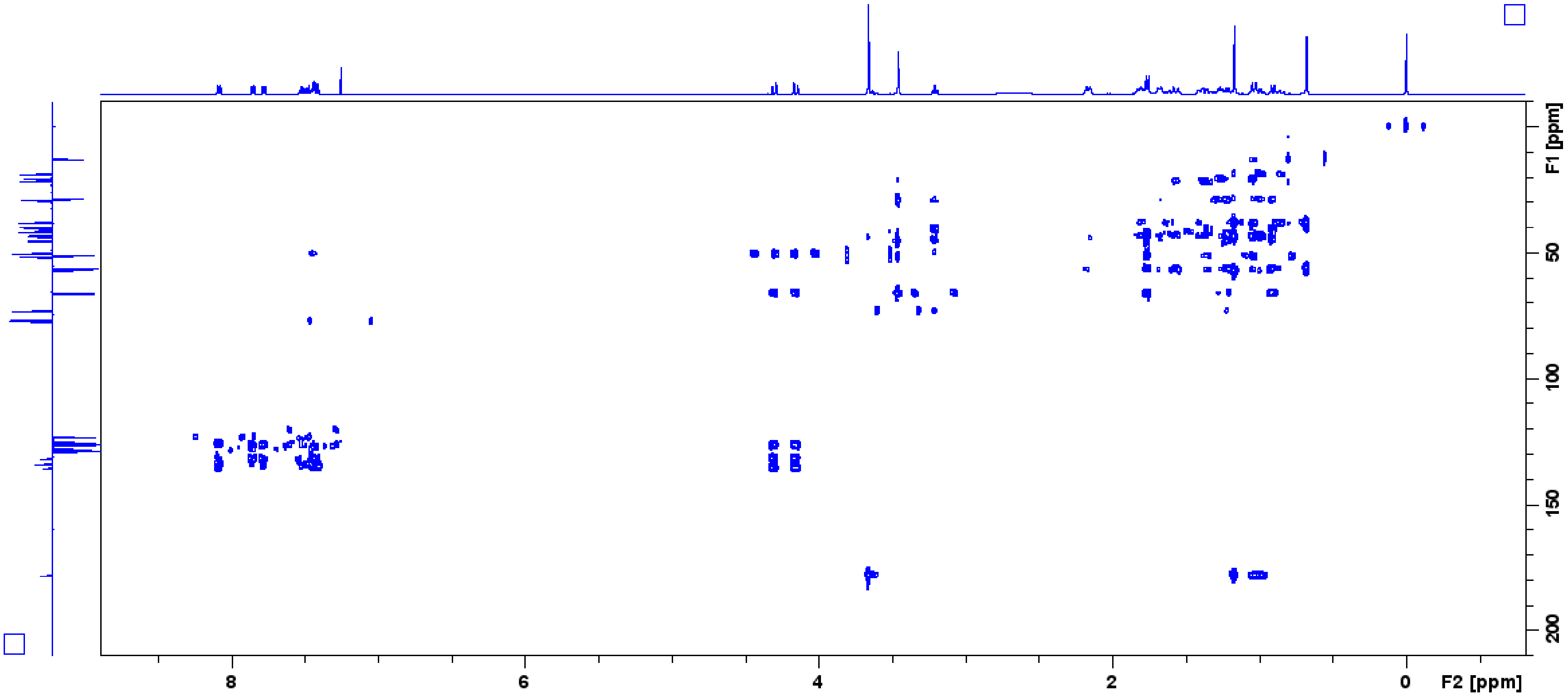

^{1}H-NMR of compound (4*R*,4a*S*,6a*R*,8*R*,9*R*,11a*R*,11b*S*)-methyl 9-(hydroxymethyl)-4,11b-dimethyl-8-(((*S*)-1-(naphthalen-2-yl)ethyl)amino)tetradecahydro-6a,9-methanocyclohepta[a]naphthalene-4-carboxylate **(16)**

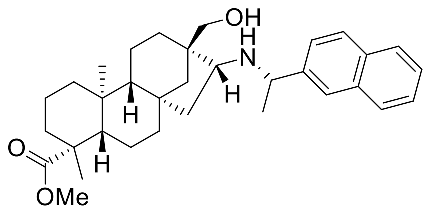

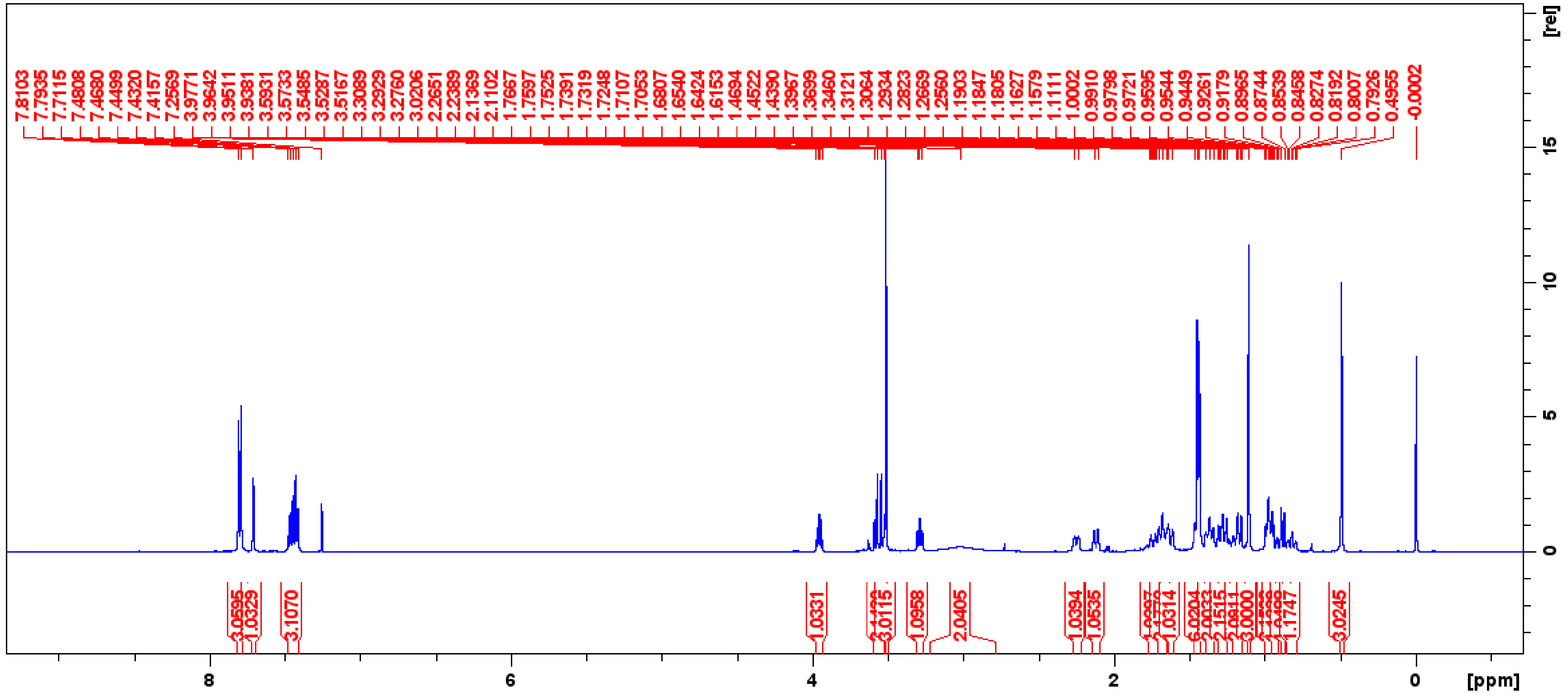

^{13}C-NMR of compound (4*R*,4a*S*,6a*R*,8*R*,9*R*,11a*R*,11b*S*)-methyl 9-(hydroxymethyl)-4,11b-dimethyl-8-(((*S*)-1-(naphthalen-2-yl)ethyl)amino)tetradecahydro-6a,9-methanocyclohepta[a]naphthalene-4-carboxylate **(16)**

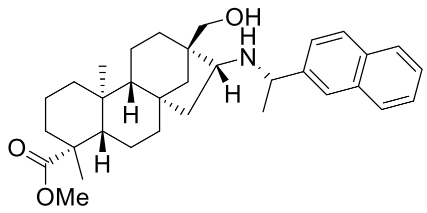

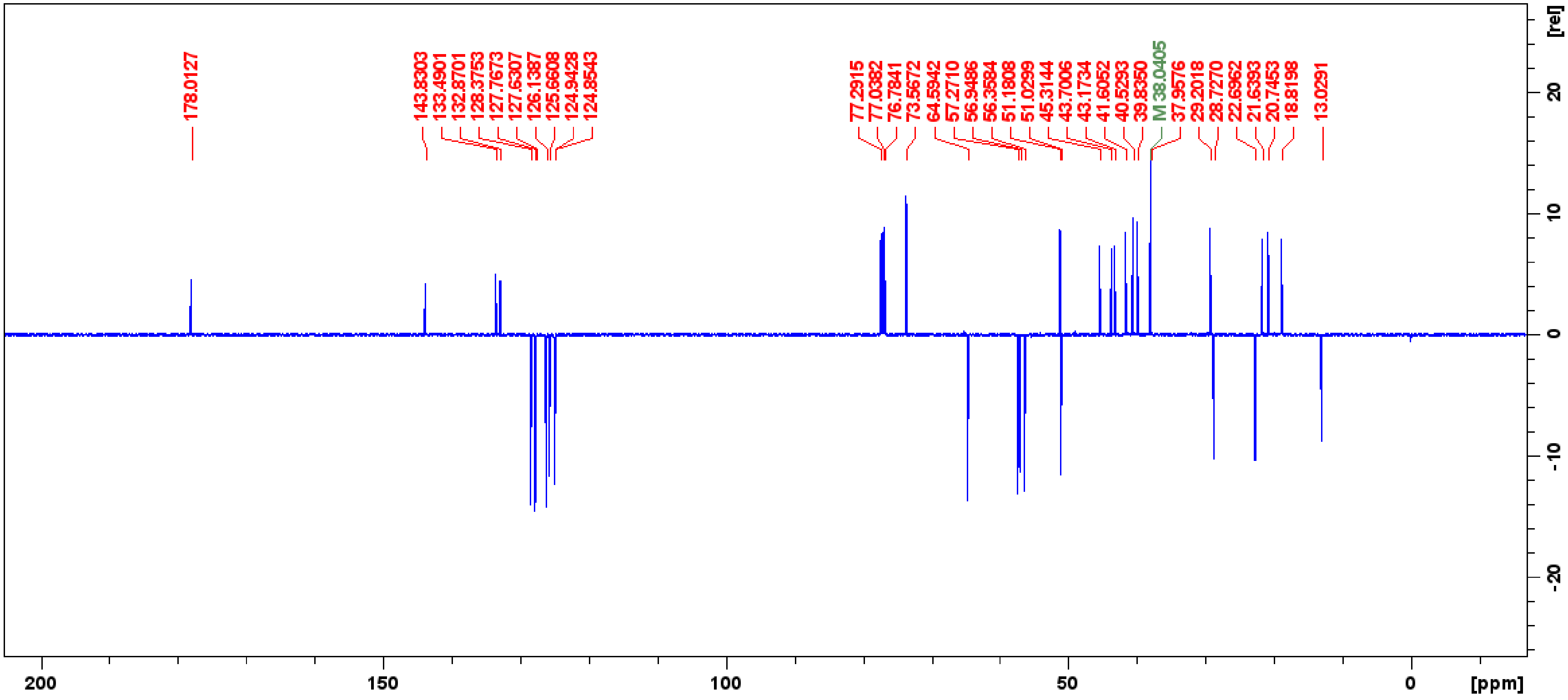

COSY of compound (4*R*,4a*S*,6a*R*,8*R*,9*R*,11a*R*,11b*S*)-methyl 9-(hydroxymethyl)-4,11b-dimethyl-8-(((*S*)-1-(naphthalen-2-yl)ethyl)amino)tetradecahydro-6a,9-methanocyclohepta[a]naphthalene-4-carboxylate **(16)**

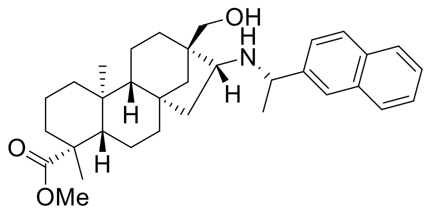

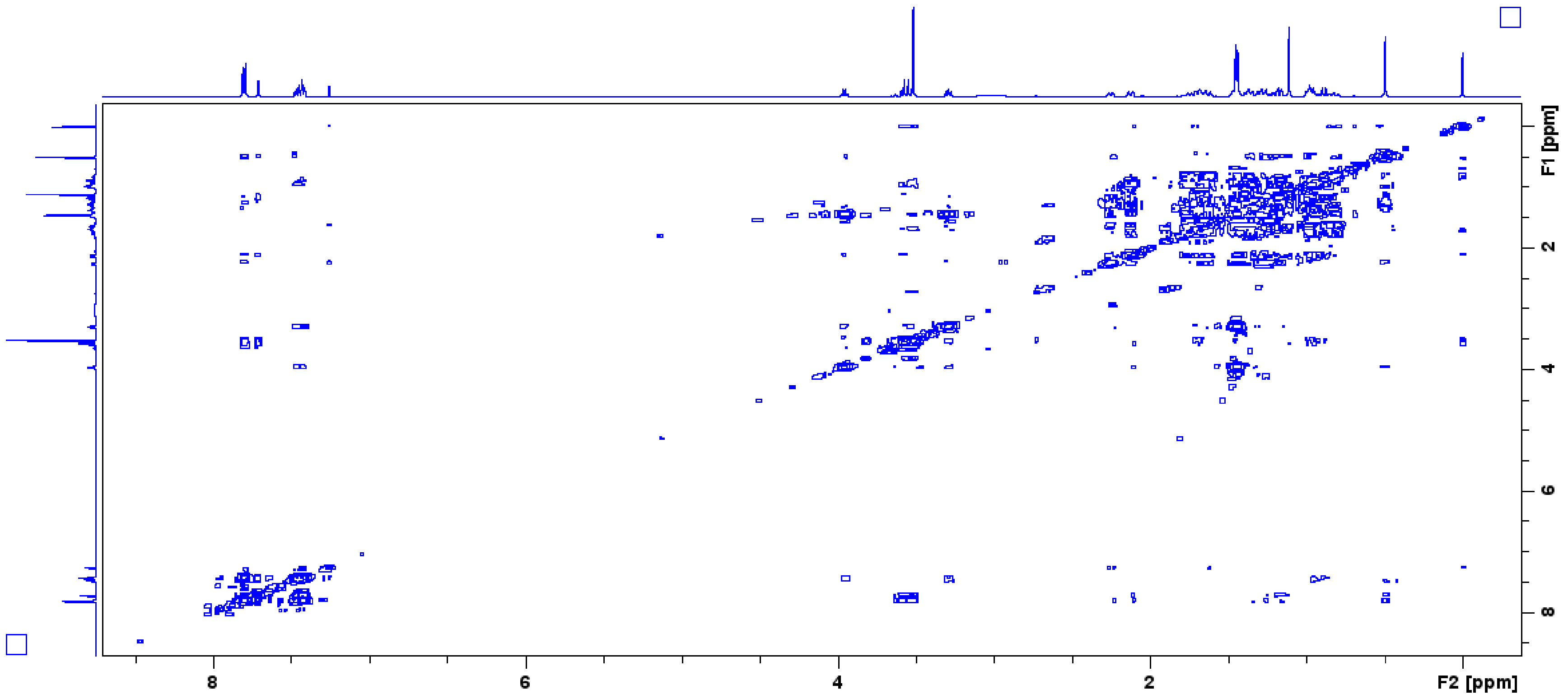

NOESY of compound (4*R*,4a*S*,6a*R*,8*R*,9*R*,11a*R*,11b*S*)-methyl 9-(hydroxymethyl)-4,11b-dimethyl-8-(((*S*)-1-(naphthalen-2-yl)ethyl)amino)tetradecahydro-6a,9-methanocyclohepta[a]naphthalene-4-carboxylate **(16)**

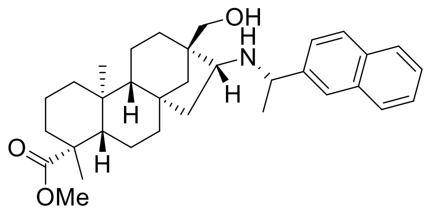

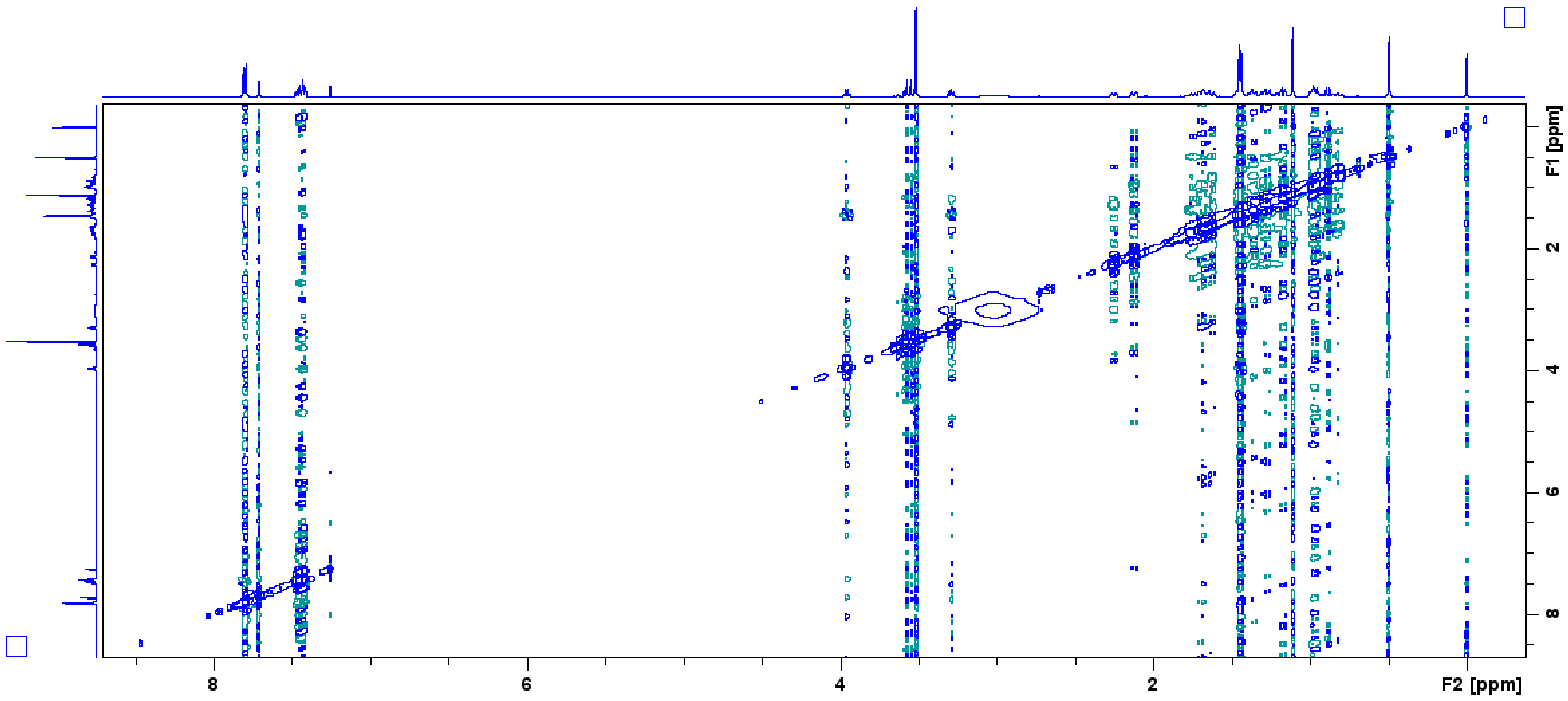

HSQC of compound (4*R*,4a*S*,6a*R*,8*R*,9*R*,11a*R*,11b*S*)-methyl 9-(hydroxymethyl)-4,11b-dimethyl-8-(((*S*)-1-(naphthalen-2-yl)ethyl)amino)tetradecahydro-6a,9-methanocyclohepta[a]naphthalene-4-carboxylate **(16)**

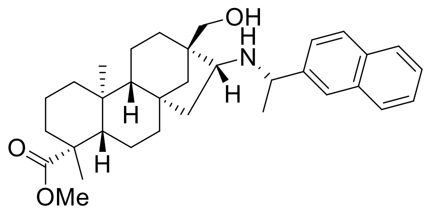

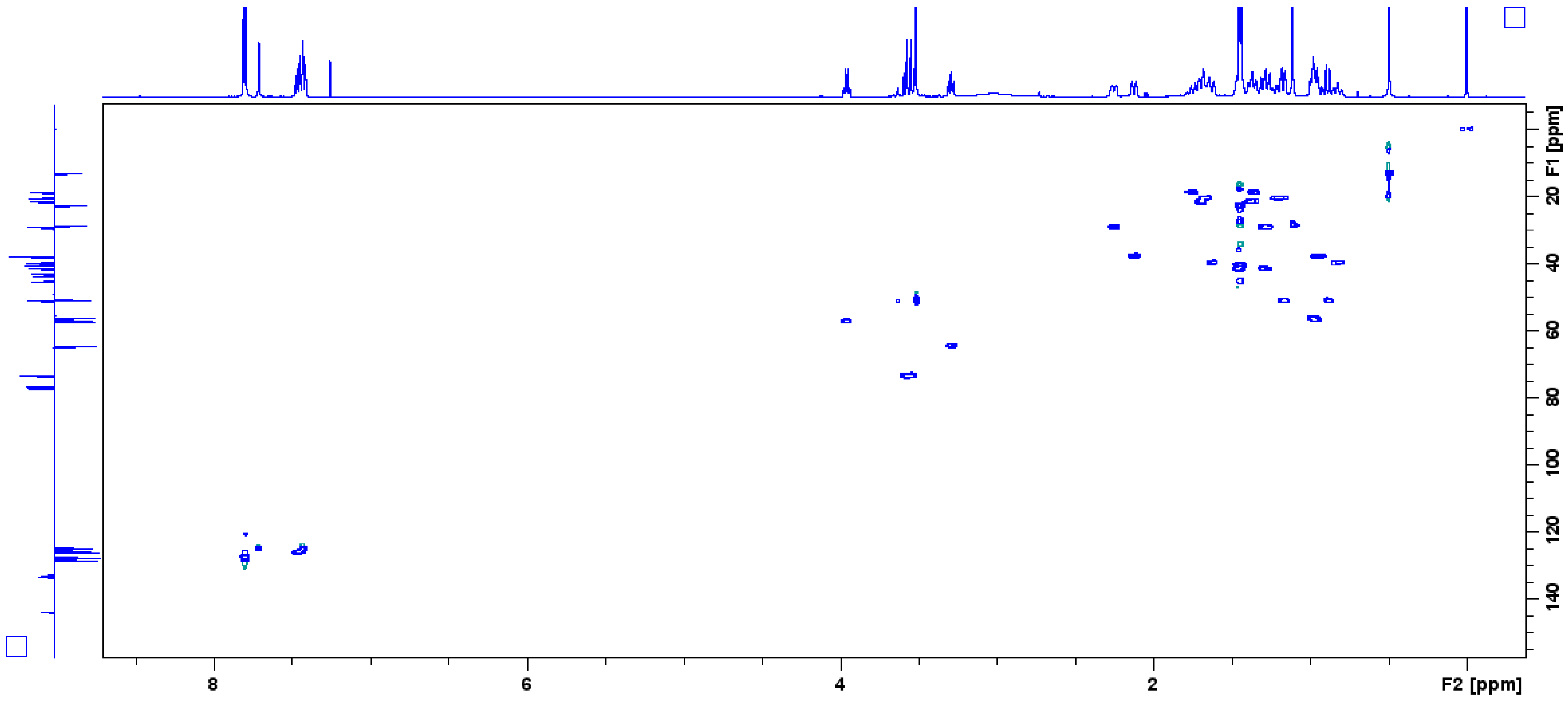

HMBC of compound (4*R*,4a*S*,6a*R*,8*R*,9*R*,11a*R*,11b*S*)-methyl 9-(hydroxymethyl)-4,11b-dimethyl-8-(((*S*)-1-(naphthalen-2-yl)ethyl)amino)tetradecahydro-6a,9-methanocyclohepta[a]naphthalene-4-carboxylate **(16)**

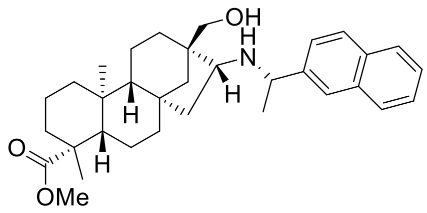

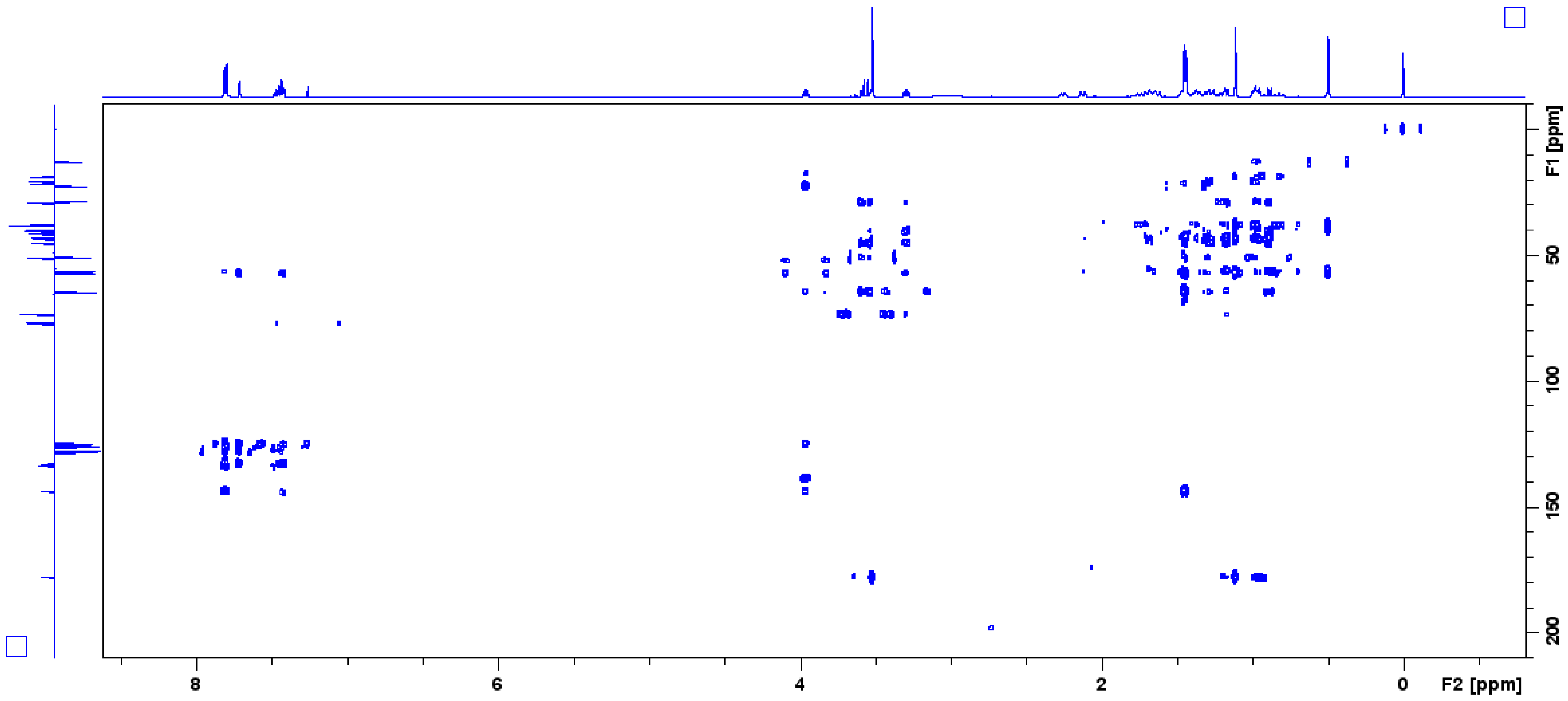

^{1}H-NMR of compound (4*R*,4a*S*,6a*R*,8*R*,9*R*,11a*R*,11b*S*)-methyl 9-(hydroxymethyl)-4,11b-dimethyl-8-(((*R*)-1-(naphthalen-2-yl)ethyl)amino)tetradecahydro-6a,9-methanocyclohepta[a]naphthalene-4-carboxylate **(17)**

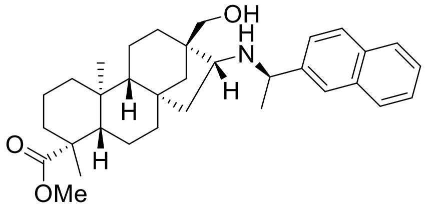

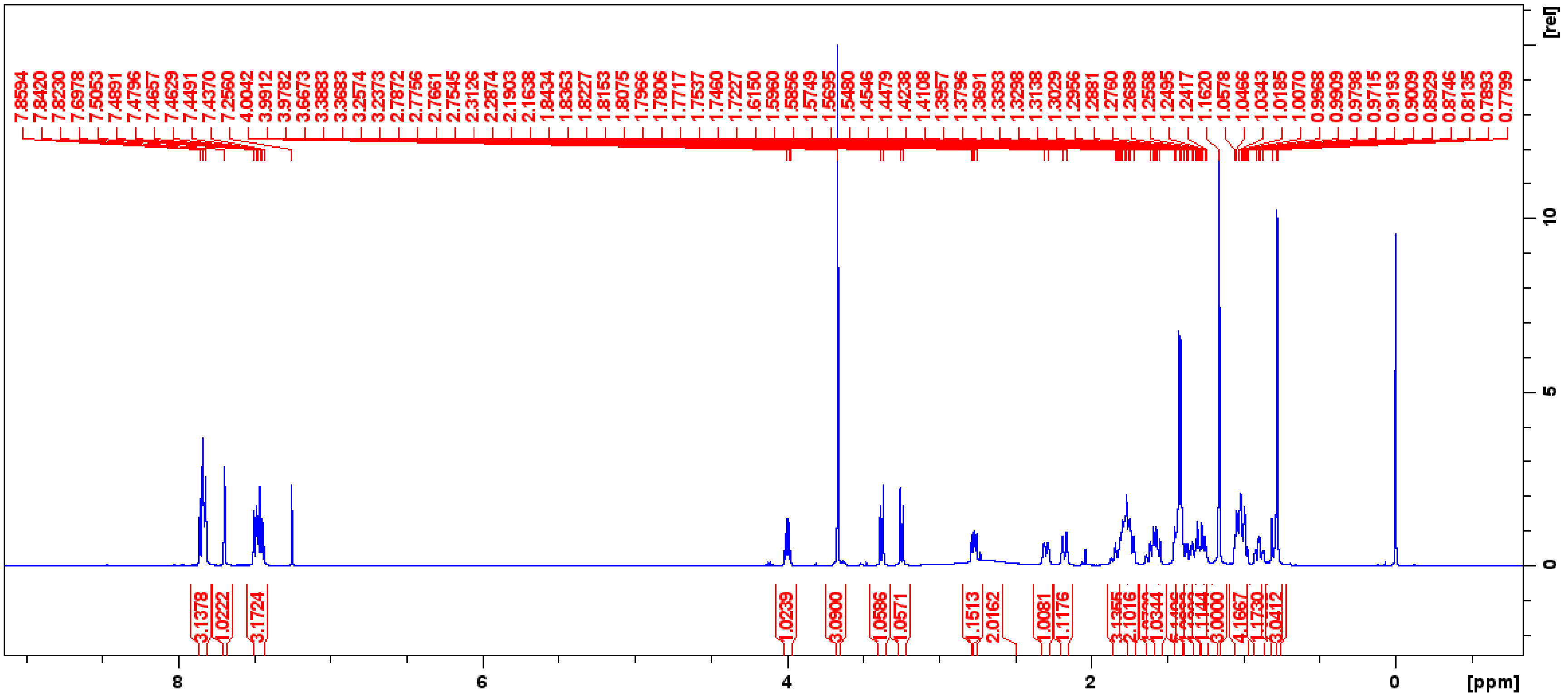

^{13}C-NMR of compound (4*R*,4a*S*,6a*R*,8*R*,9*R*,11a*R*,11b*S*)-methyl 9-(hydroxymethyl)-4,11b-dimethyl-8-(((*R*)-1-(naphthalen-2-yl)ethyl)amino)tetradecahydro-6a,9-methanocyclohepta[a]naphthalene-4-carboxylate **(17)**

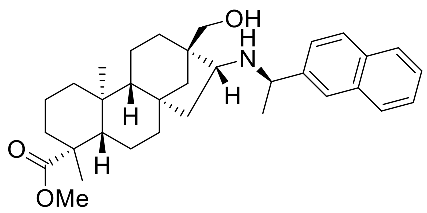

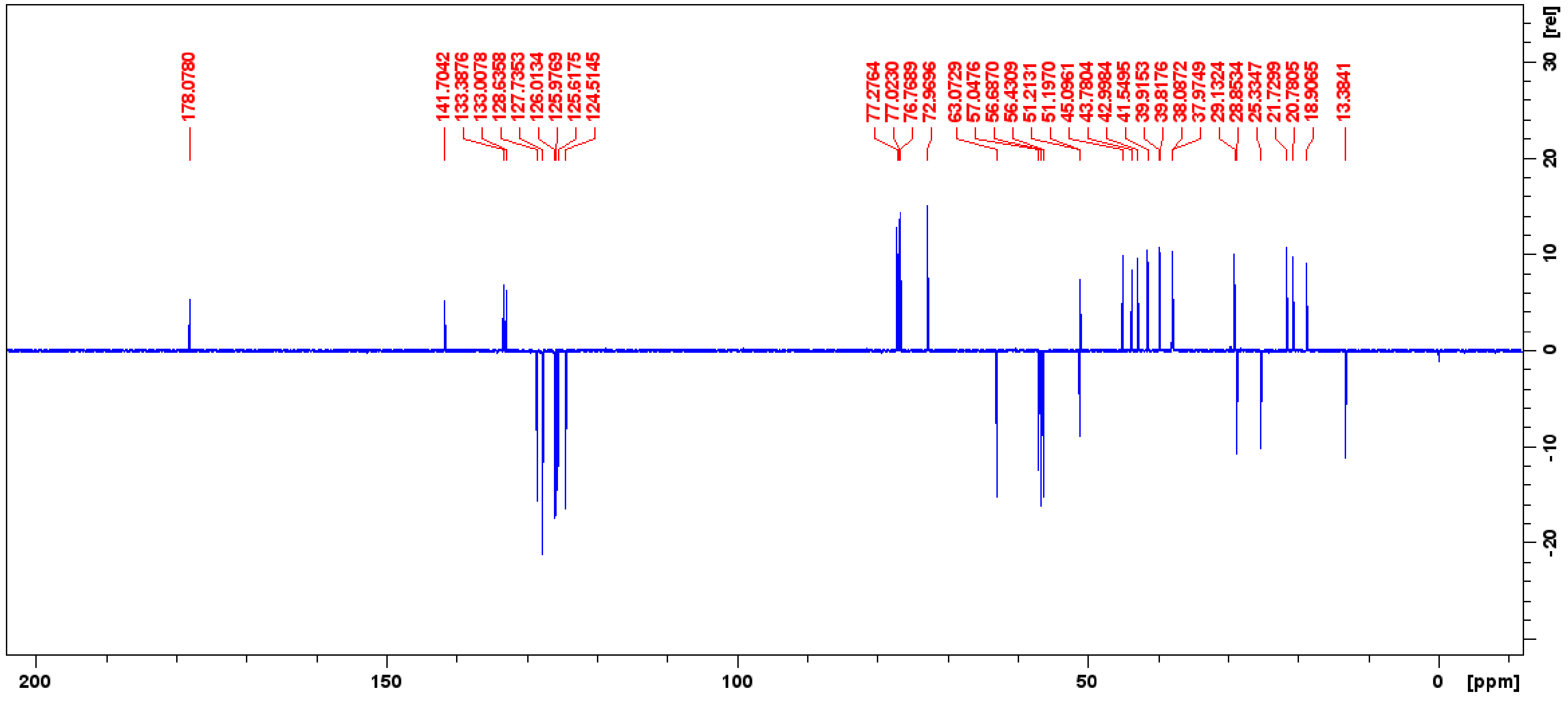

COSY of compound (4*R*,4a*S*,6a*R*,8*R*,9*R*,11a*R*,11b*S*)-methyl 9-(hydroxymethyl)-4,11b-dimethyl-8-(((*R*)-1-(naphthalen-2-yl)ethyl)amino)tetradecahydro-6a,9-methanocyclohepta[a]naphthalene-4-carboxylate **(17)**

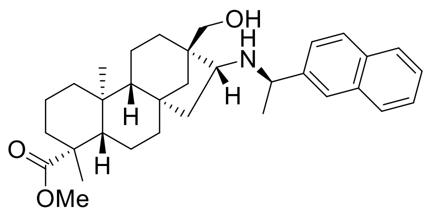

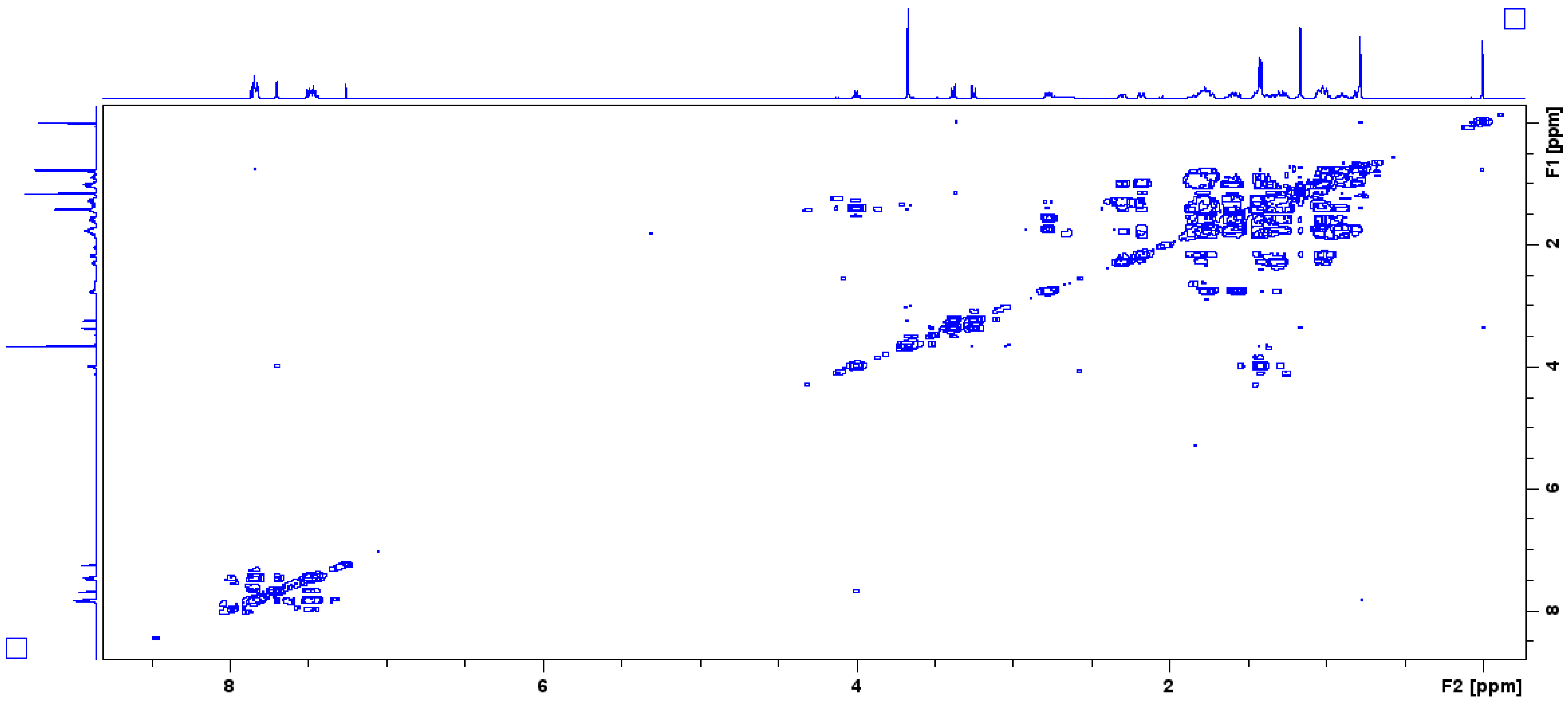

NOESY of compound (4*R*,4a*S*,6a*R*,8*R*,9*R*,11a*R*,11b*S*)-methyl 9-(hydroxymethyl)-4,11b-dimethyl-8-(((*R*)-1-(naphthalen-2-yl)ethyl)amino)tetradecahydro-6a,9-methanocyclohepta[a]naphthalene-4-carboxylate **(17)**

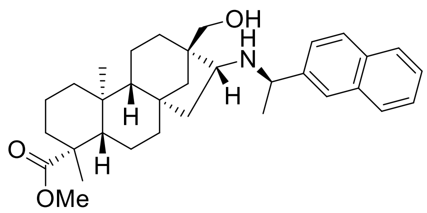

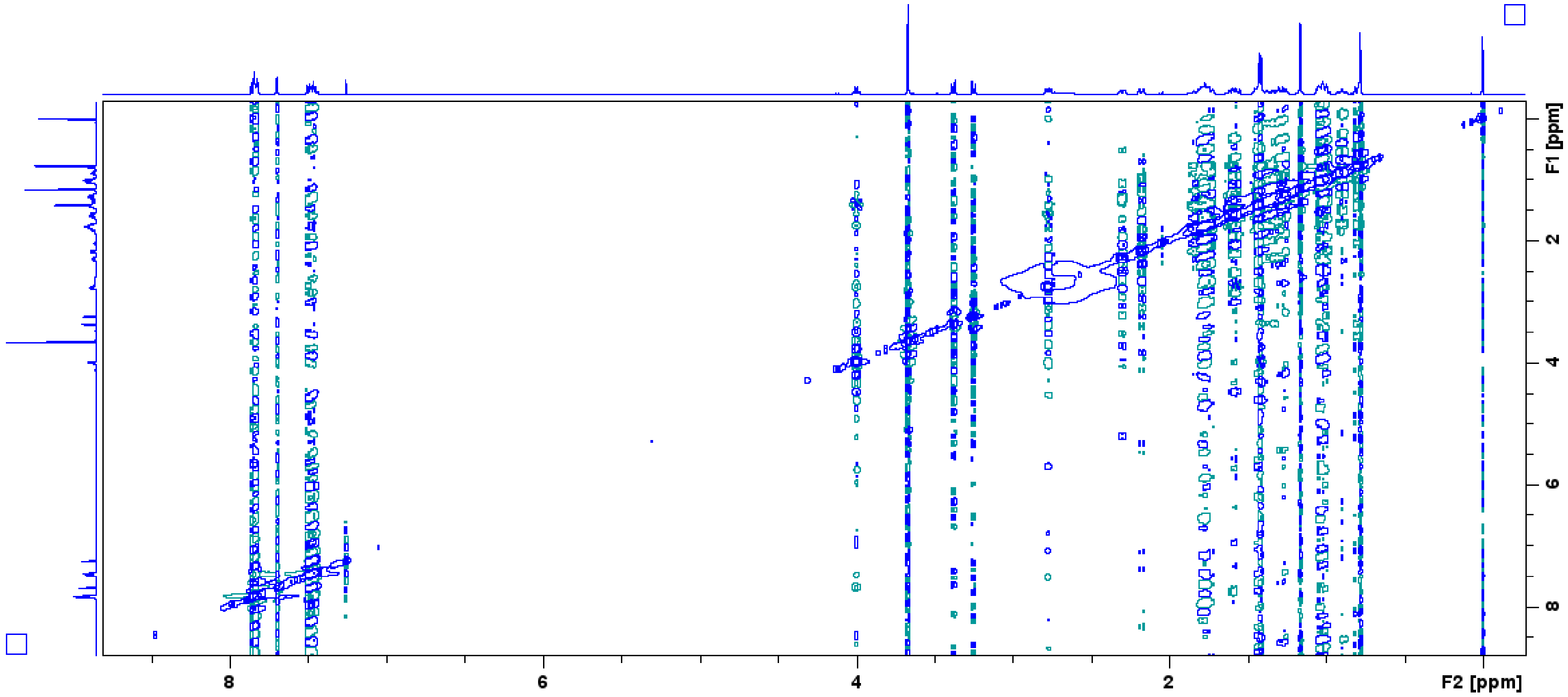

HSQC of compound (4*R*,4a*S*,6a*R*,8*R*,9*R*,11a*R*,11b*S*)-methyl 9-(hydroxymethyl)-4,11b-dimethyl-8-(((*R*)-1-(naphthalen-2-yl)ethyl)amino)tetradecahydro-6a,9-methanocyclohepta[a]naphthalene-4-carboxylate **(17)**

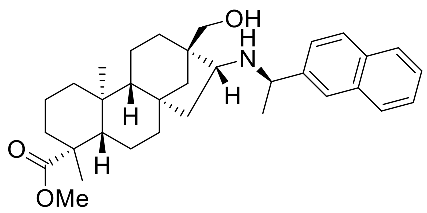

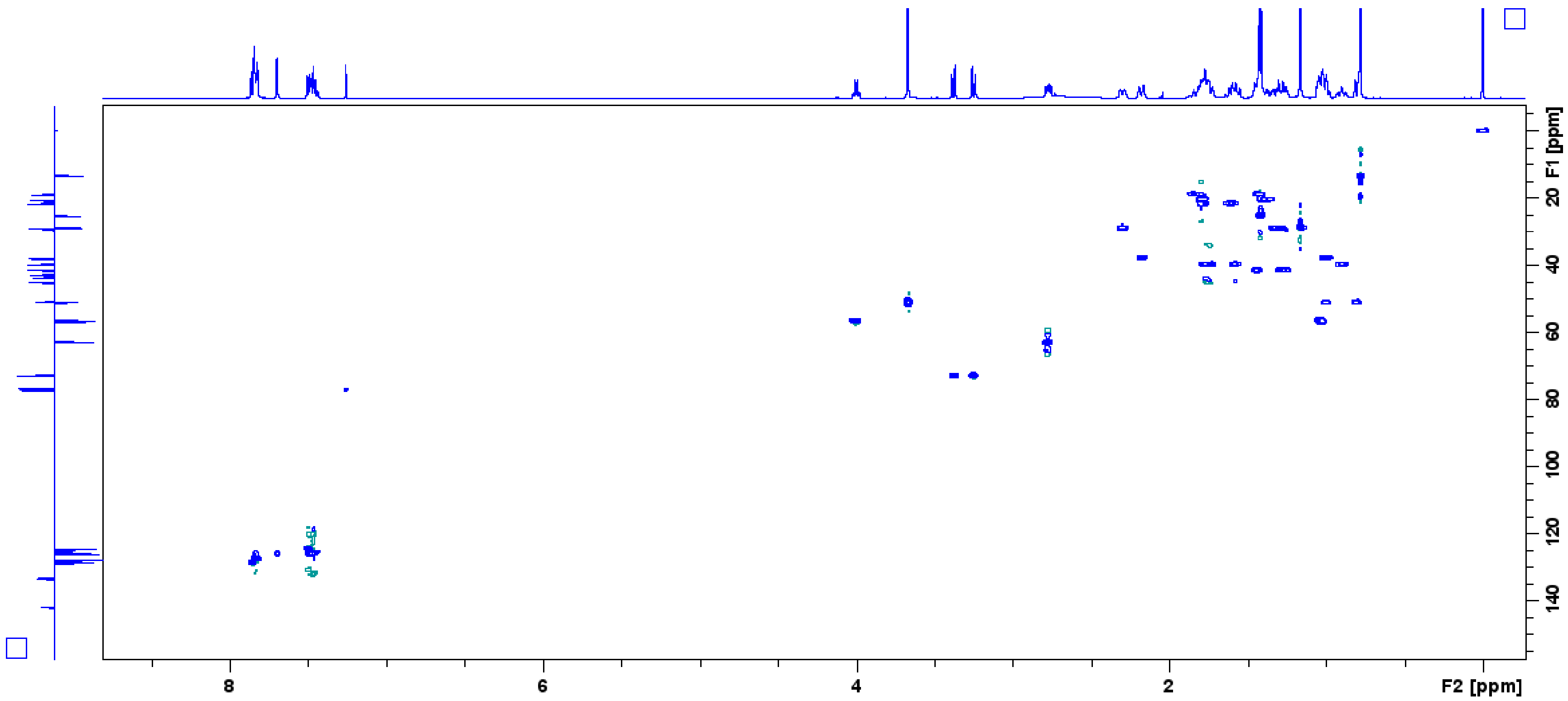

HMBC of compound (4*R*,4a*S*,6a*R*,8*R*,9*R*,11a*R*,11b*S*)-methyl 9-(hydroxymethyl)-4,11b-dimethyl-8-(((*R*)-1-(naphthalen-2-yl)ethyl)amino)tetradecahydro-6a,9-methanocyclohepta[a]naphthalene-4-carboxylate **(17)**

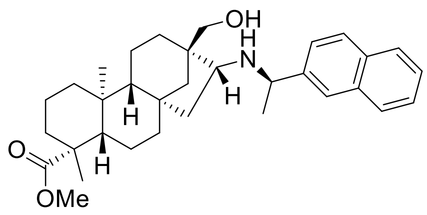

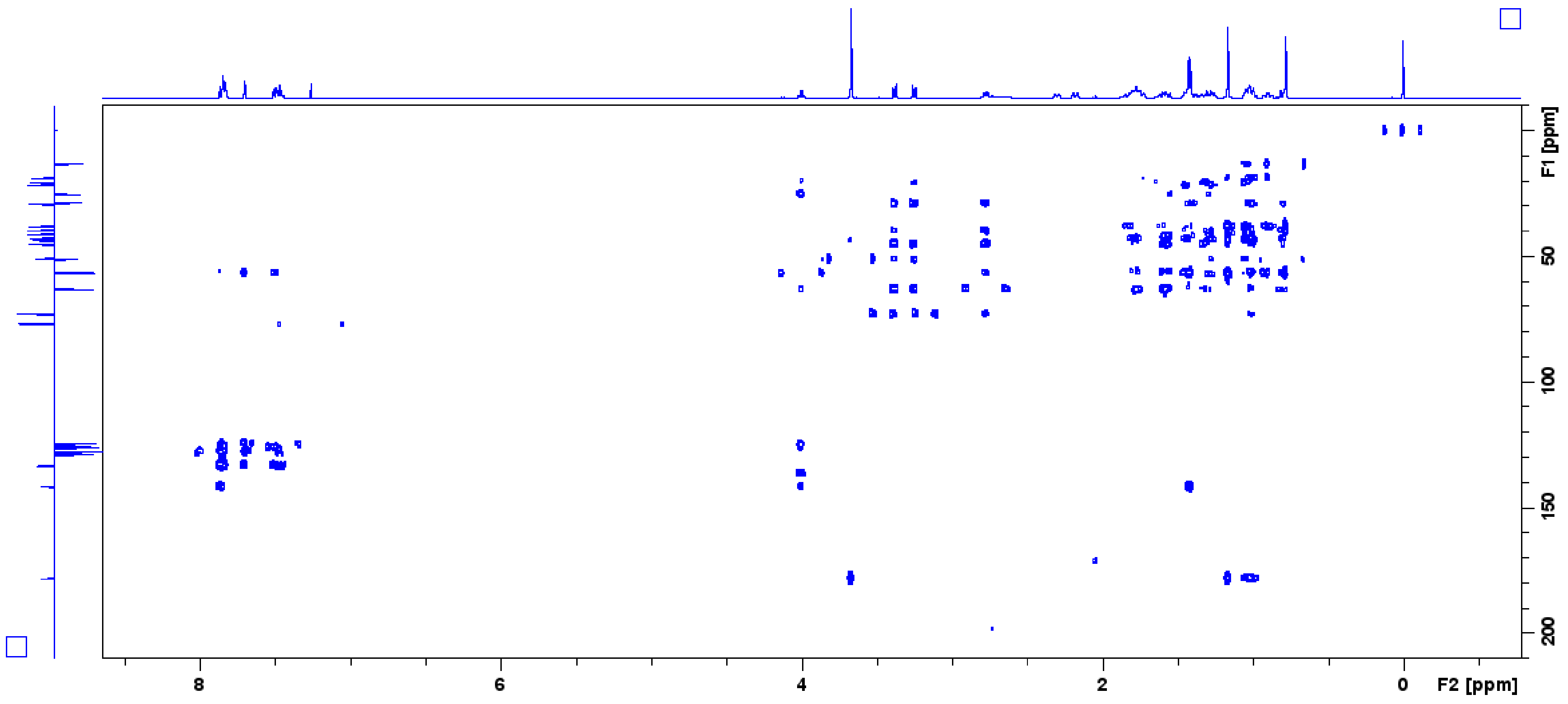

^{1}H-NMR of compound (4*R*,4a*S*,6a*R*,9*S*,11a*R*,11b*S*)-methyl 4,11b-dimethyl-9-(((methylsulfonyl)oxy)methyl)-8-oxotetradecahydro-6a,9-methanocyclohepta[a]naphthalene-4-carboxylate **(18)**

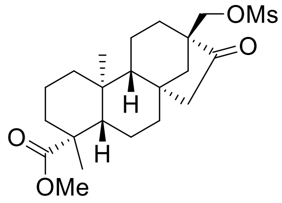

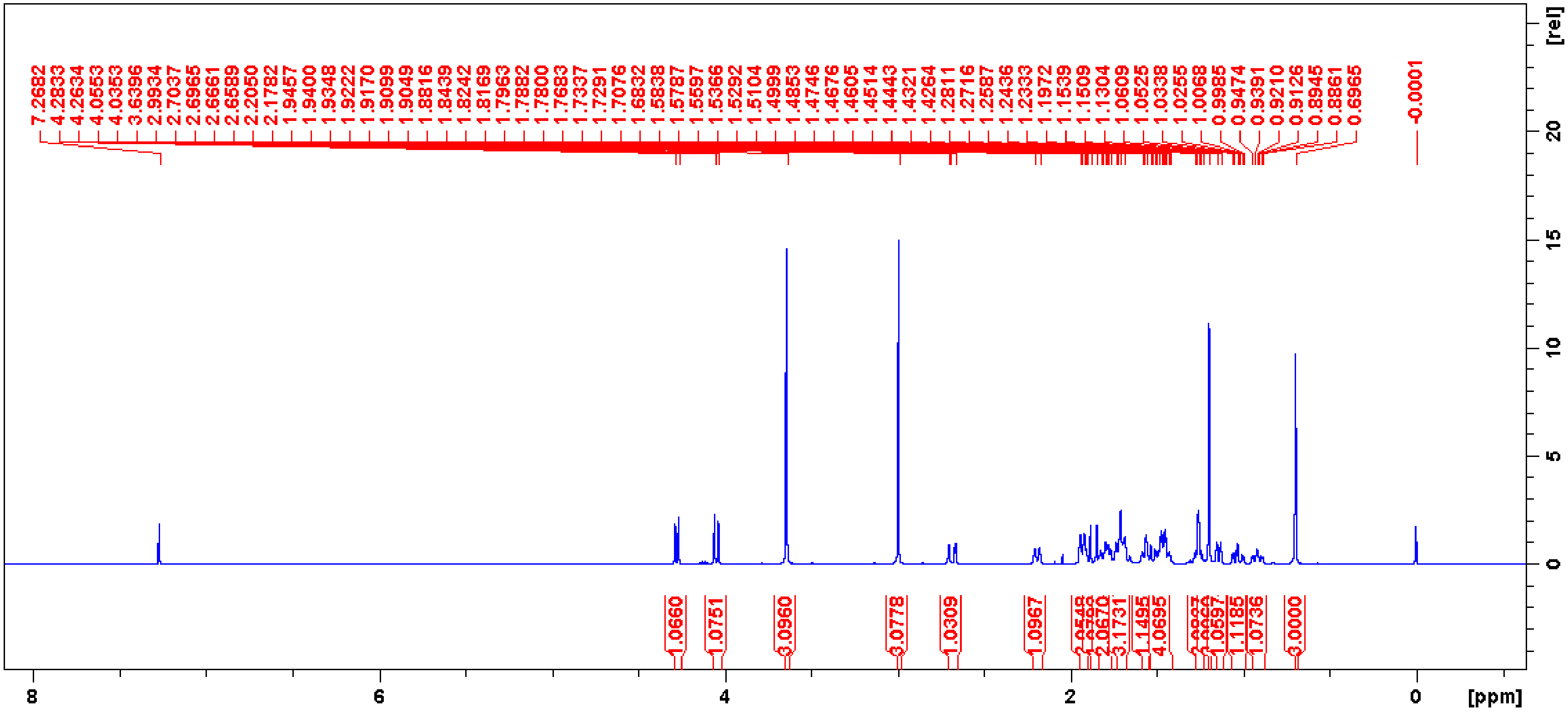

^{13}C-NMR of compound (4*R*,4a*S*,6a*R*,9*S*,11a*R*,11b*S*)-methyl 4,11b-dimethyl-9-(((methylsulfonyl)oxy)methyl)-8-oxotetradecahydro-6a,9-methanocyclohepta[a]naphthalene-4-carboxylate **(18)**

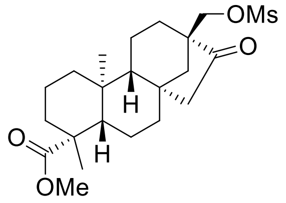

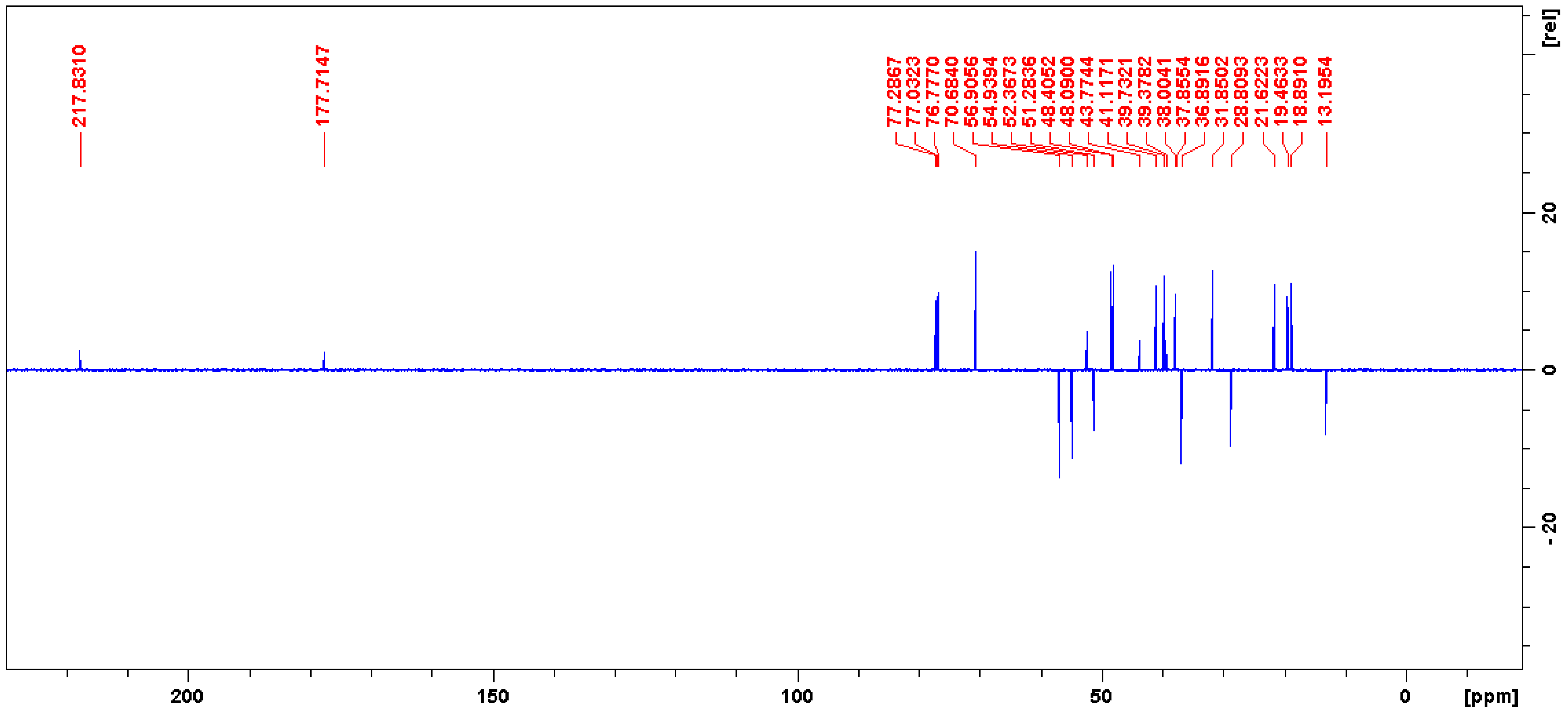

COSY of compound (4*R*,4a*S*,6a*R*,9*S*,11a*R*,11b*S*)-methyl 4,11b-dimethyl-9-(((methylsulfonyl)oxy)methyl)-8-oxotetradecahydro-6a,9-methanocyclohepta[a]naphthalene-4-carboxylate **(18)**

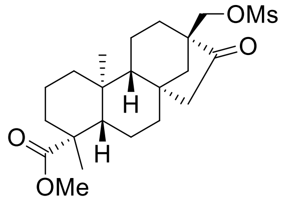

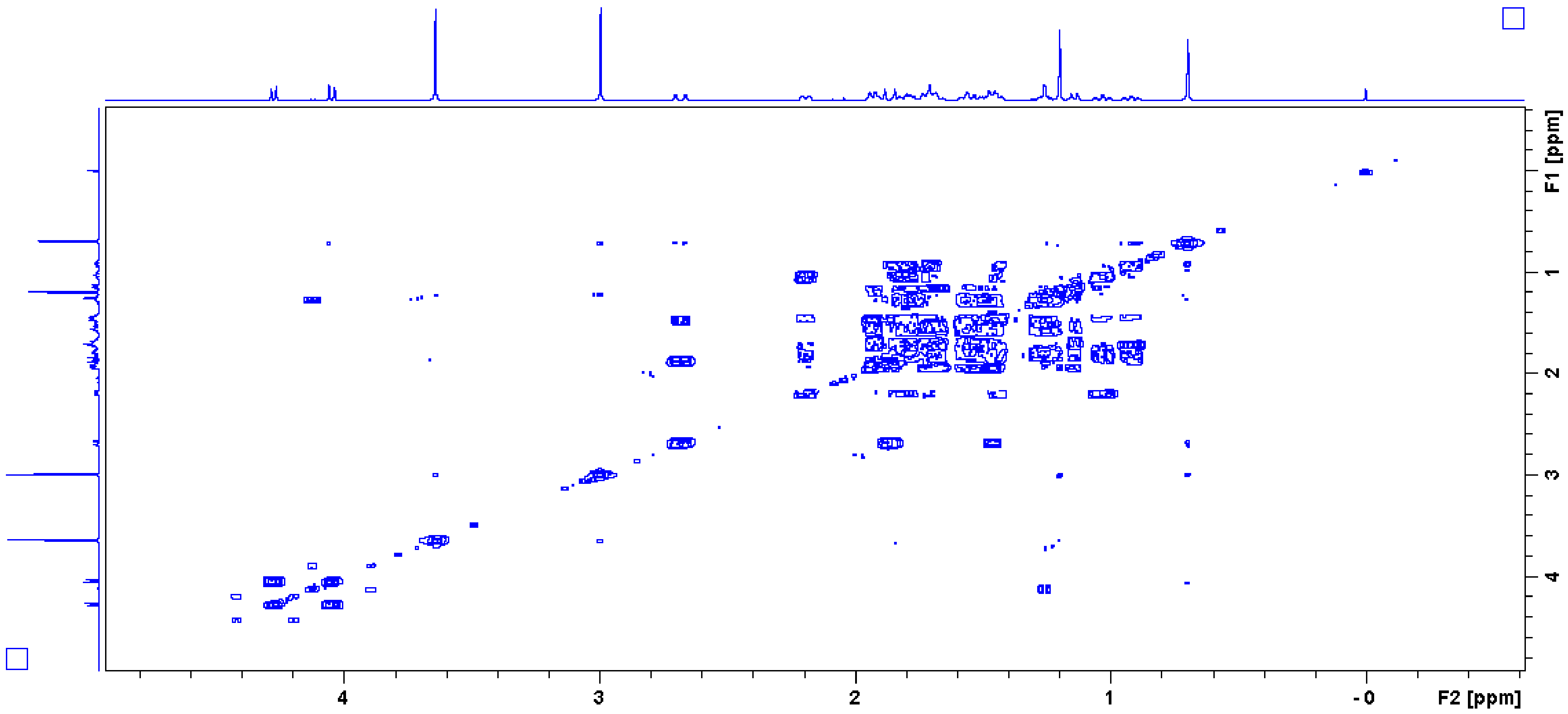

NOESY of compound (4*R*,4a*S*,6a*R*,9*S*,11a*R*,11b*S*)-methyl 4,11b-dimethyl-9-(((methylsulfonyl)oxy)methyl)-8-oxotetradecahydro-6a,9-methanocyclohepta[a]naphthalene-4-carboxylate **(18)**

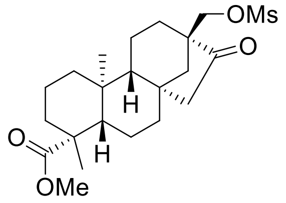

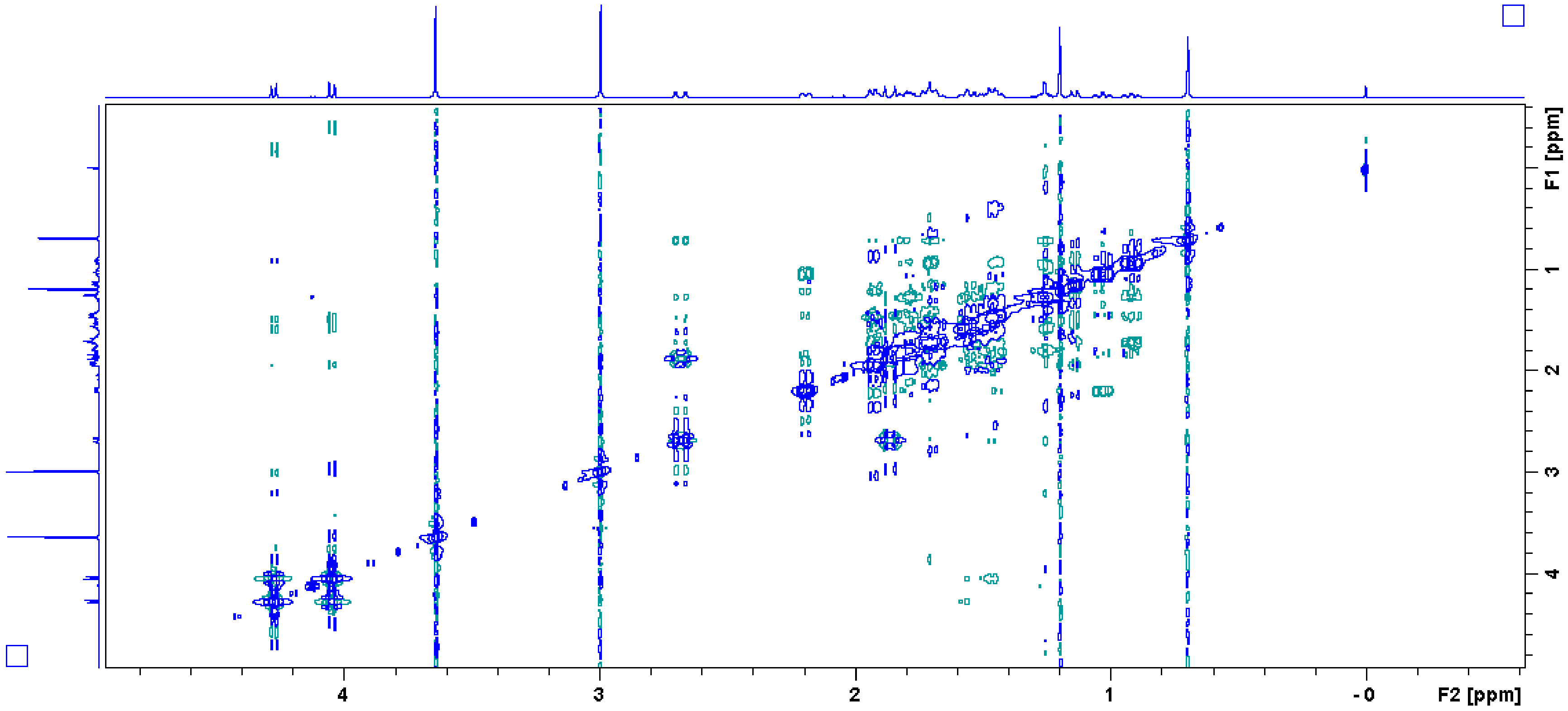

HSQC of compound (4*R*,4a*S*,6a*R*,9*S*,11a*R*,11b*S*)-methyl 4,11b-dimethyl-9-(((methylsulfonyl)oxy)methyl)-8-oxotetradecahydro-6a,9-methanocyclohepta[a]naphthalene-4-carboxylate **(18)**

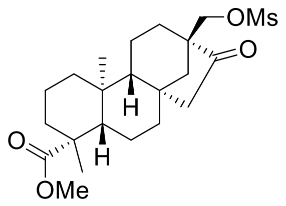

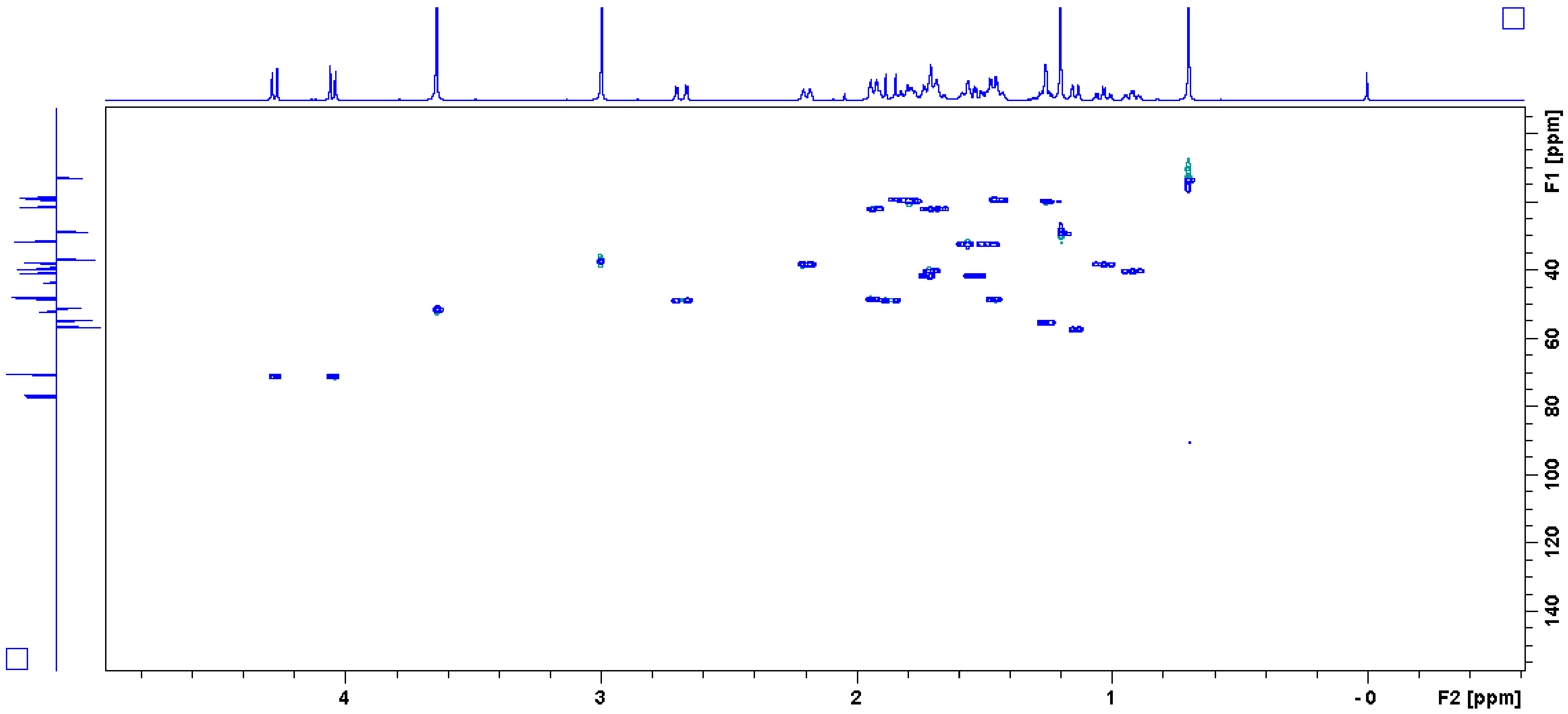

HMBC of compound (4*R*,4a*S*,6a*R*,9*S*,11a*R*,11b*S*)-methyl 4,11b-dimethyl-9-(((methylsulfonyl)oxy)methyl)-8-oxotetradecahydro-6a,9-methanocyclohepta[a]naphthalene-4-carboxylate **(18)**

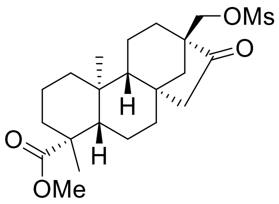

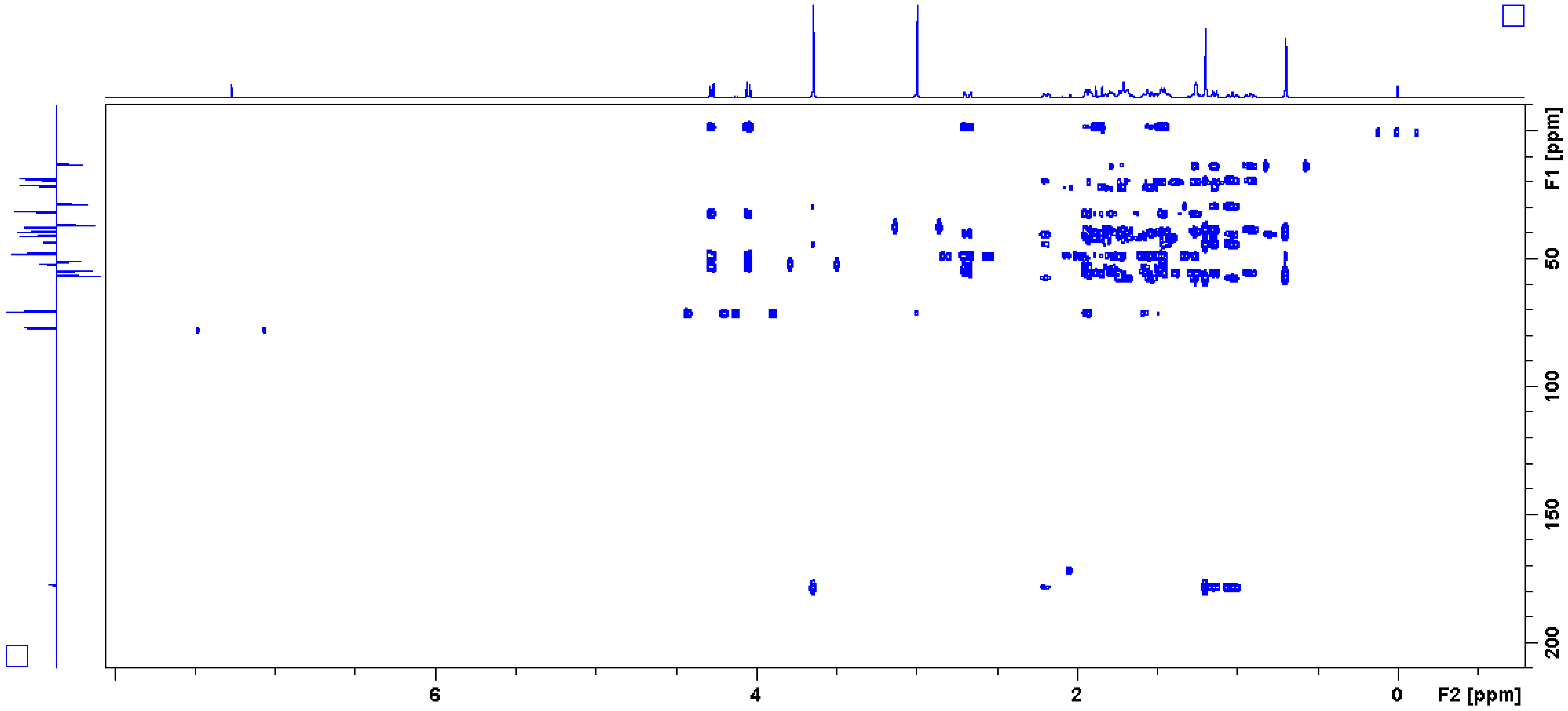

^{1}H-NMR of compound (4*R*,4a*S*,6a*R*,9*S*,11a*R*,11b*S*)-methyl 9-(azidomethyl)-4,11b-dimethyl-8-oxotetradecahydro-6a,9-methanocyclohepta[a]naphthalene-4-carboxylate **(19)**

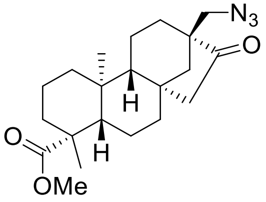

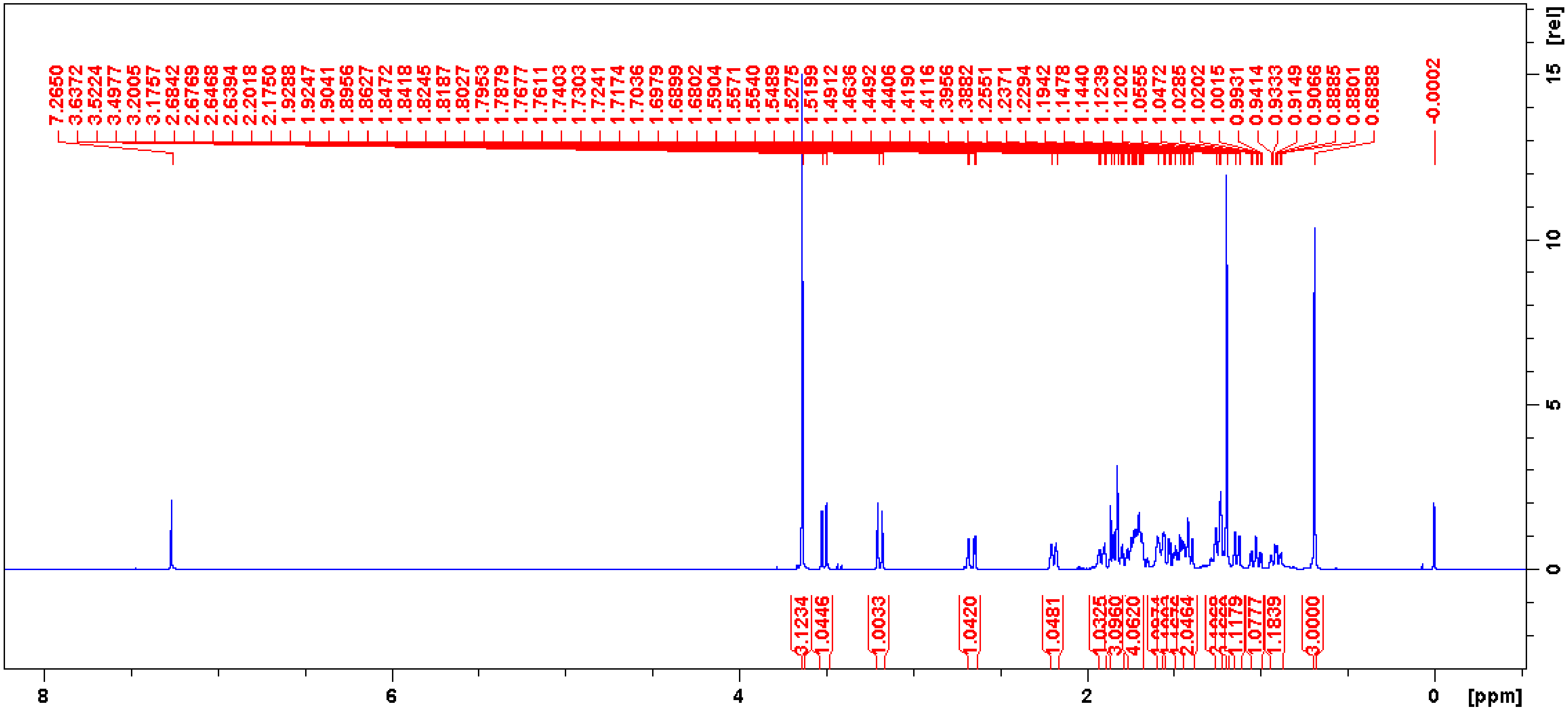

^{13}C-NMR of compound (4*R*,4a*S*,6a*R*,9*S*,11a*R*,11b*S*)-methyl 9-(azidomethyl)-4,11b-dimethyl-8-oxotetradecahydro-6a,9-methanocyclohepta[a]naphthalene-4-carboxylate **(19)**

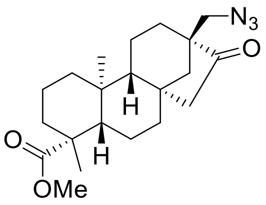

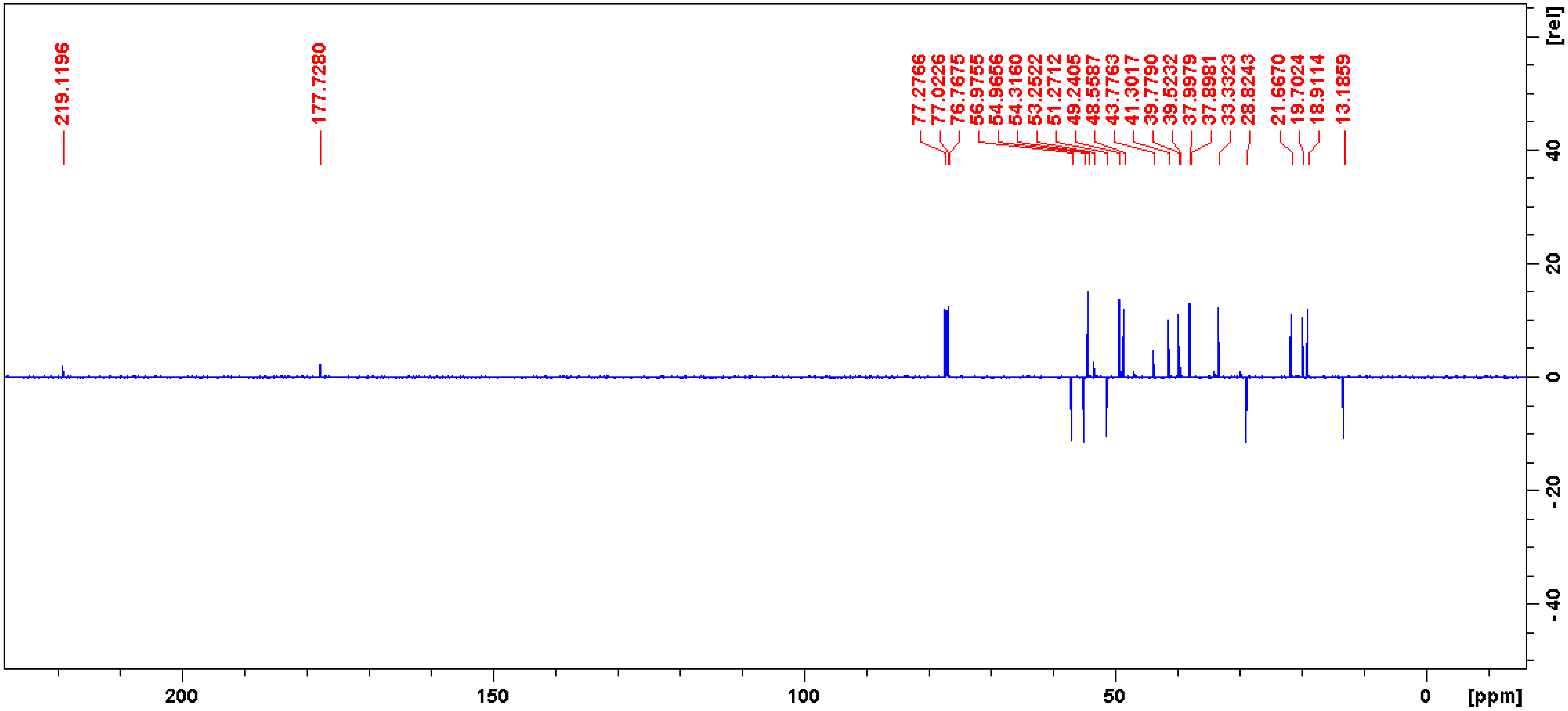

COSY of compound (4*R*,4a*S*,6a*R*,9*S*,11a*R*,11b*S*)-methyl 9-(azidomethyl)-4,11b-dimethyl-8-oxotetradecahydro-6a,9-methanocyclohepta[a]naphthalene-4-carboxylate **(19)**

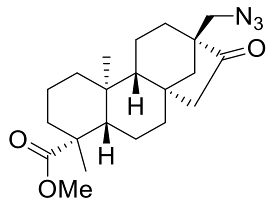

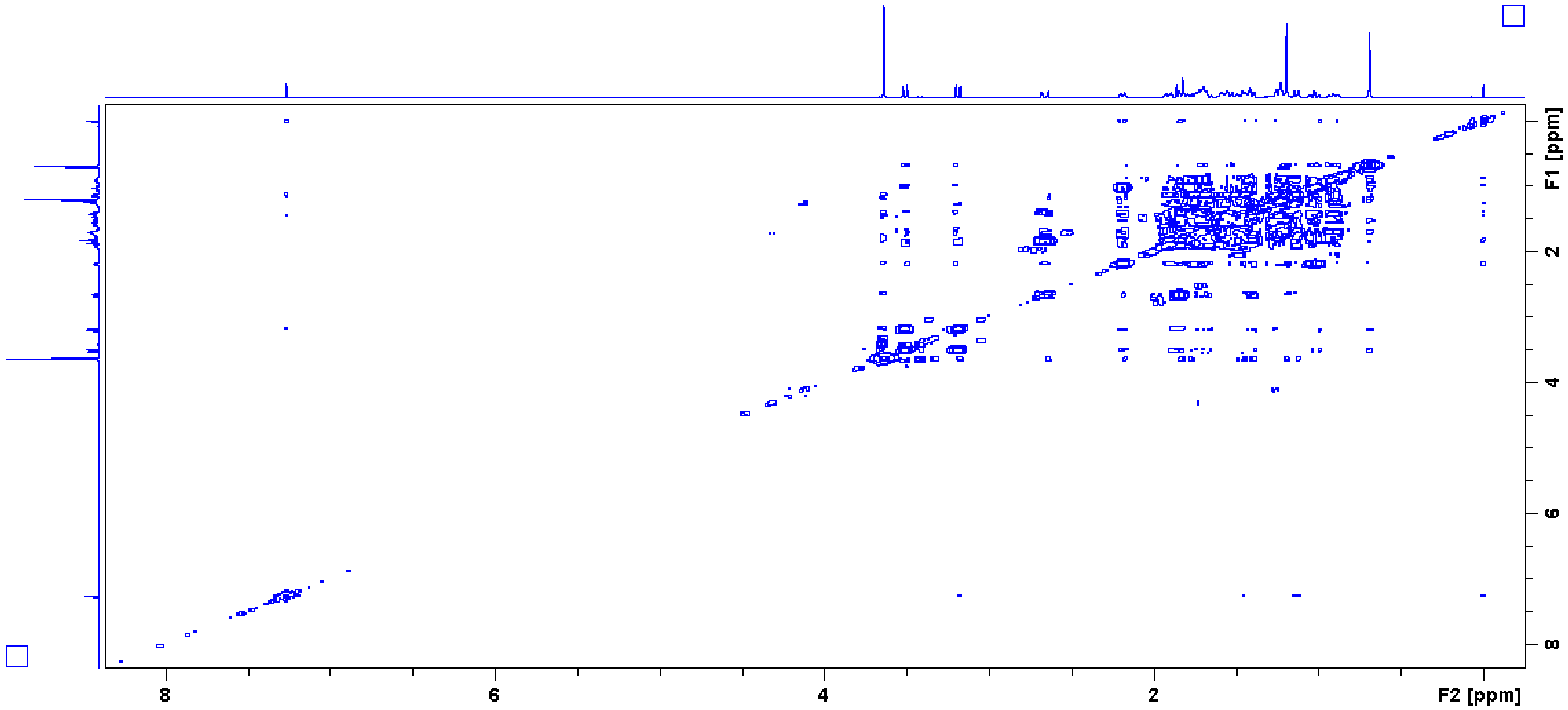

NOESY of compound (4*R*,4a*S*,6a*R*,9*S*,11a*R*,11b*S*)-methyl 9-(azidomethyl)-4,11b-dimethyl-8-oxotetradecahydro-6a,9-methanocyclohepta[a]naphthalene-4-carboxylate **(19)**

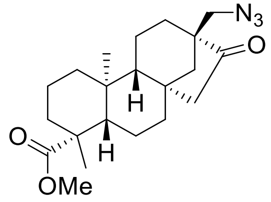

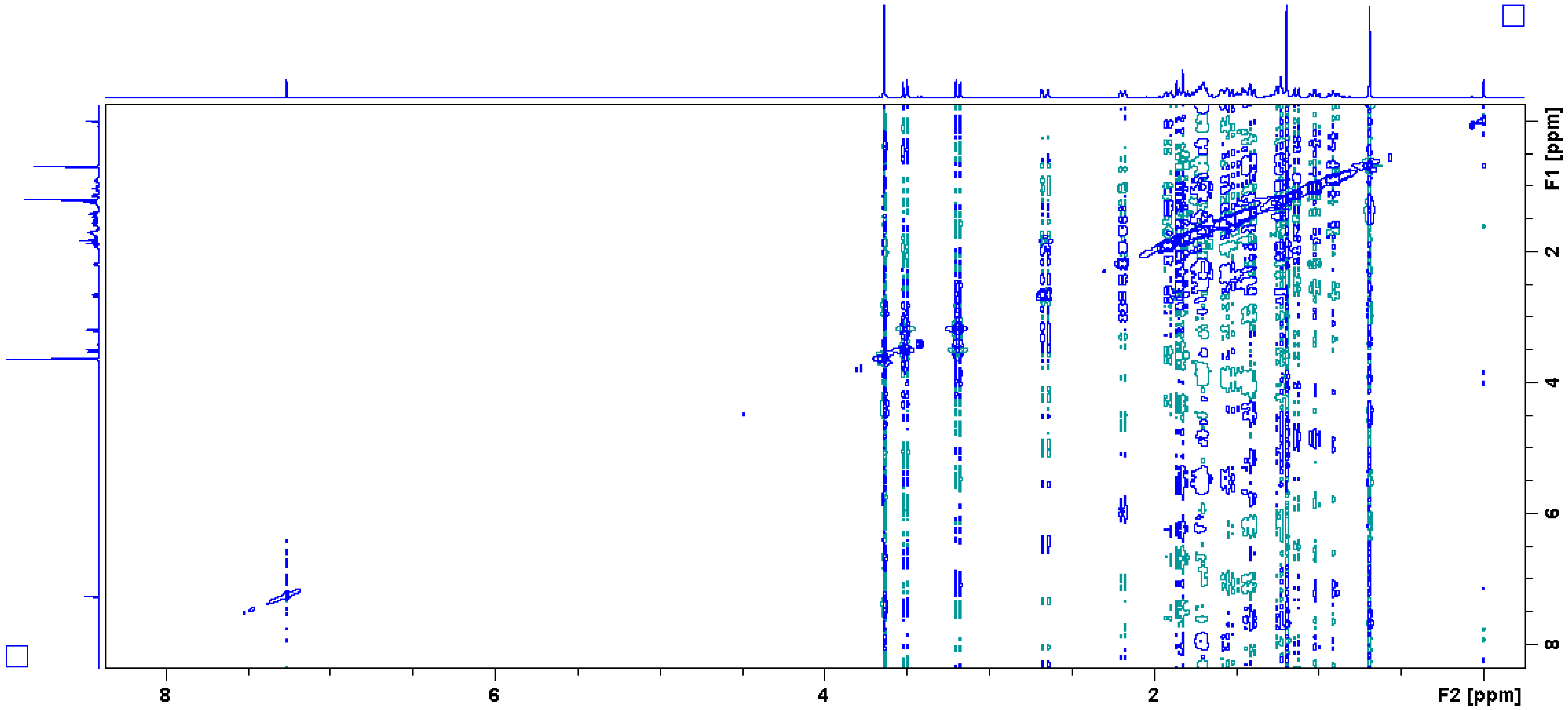

HSQC of compound (4*R*,4a*S*,6a*R*,9*S*,11a*R*,11b*S*)-methyl 9-(azidomethyl)-4,11b-dimethyl-8-oxotetradecahydro-6a,9-methanocyclohepta[a]naphthalene-4-carboxylate **(19)**

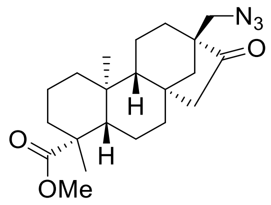

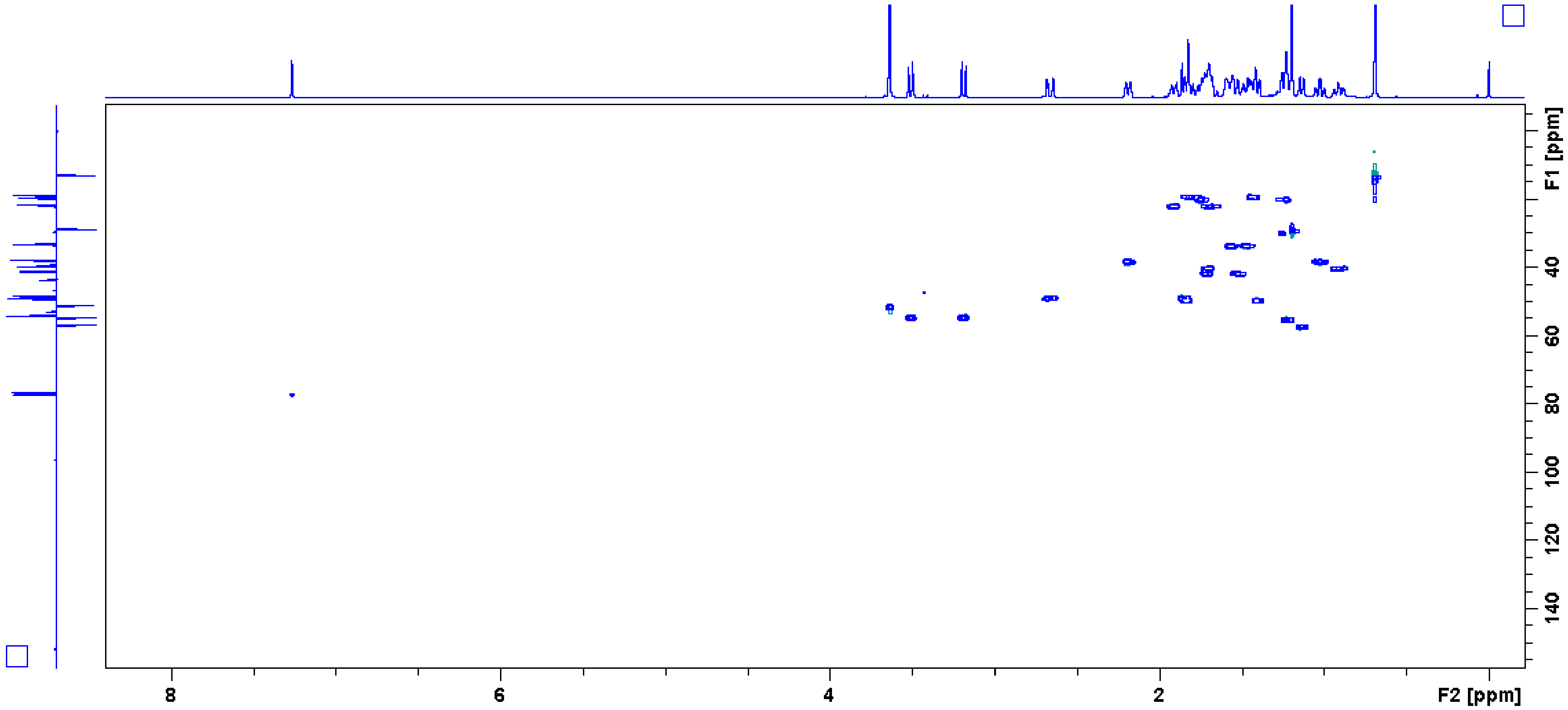

HMBC of compound (4*R*,4a*S*,6a*R*,9*S*,11a*R*,11b*S*)-methyl 9-(azidomethyl)-4,11b-dimethyl-8-oxotetradecahydro-6a,9-methanocyclohepta[a]naphthalene-4-carboxylate **(19)**

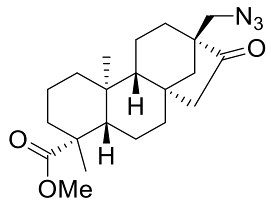

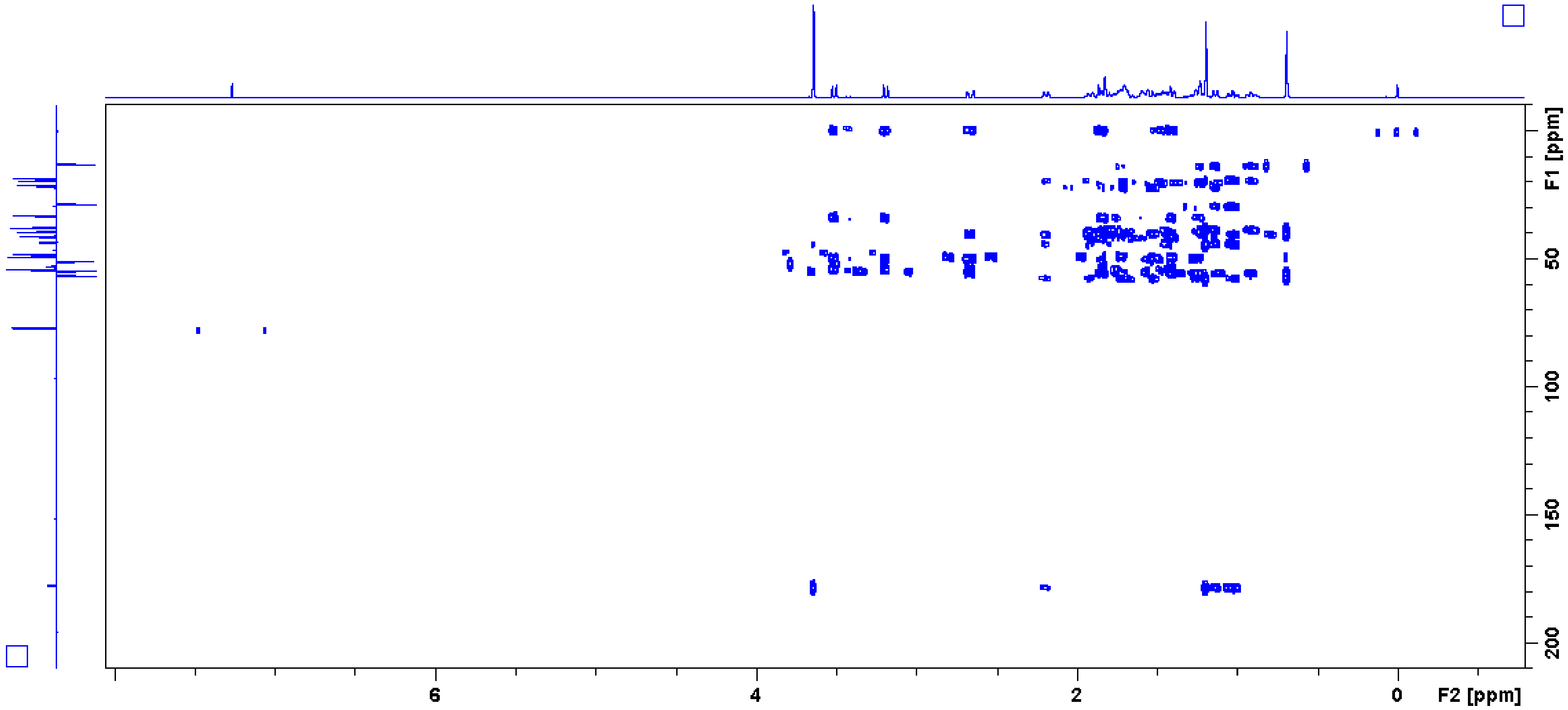

^{1}H-NMR of compound (4*R*,4a*S*,6a*R*,8*R*,9*S*,11a*R*,11b*S*)-methyl 9-(azidomethyl)-8-hydroxy-4,11b-dimethyltetradecahydro-6a,9-methanocyclohepta[a]naphthalene-4-carboxylate **(20)**

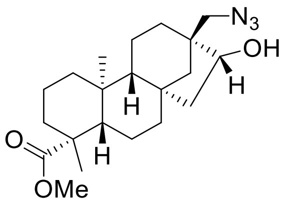

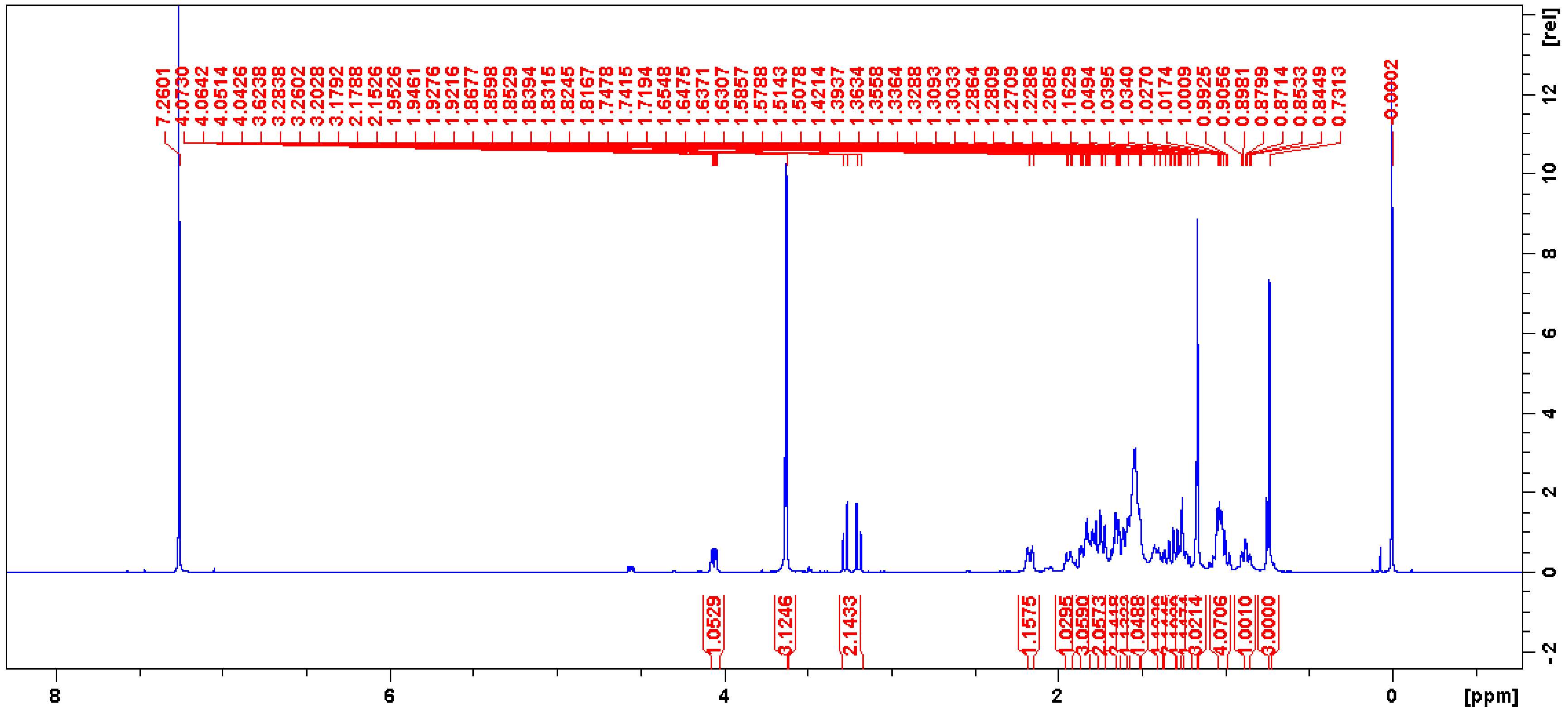

^{13}C-NMR of compound (4*R*,4a*S*,6a*R*,8*R*,9*S*,11a*R*,11b*S*)-methyl 9-(azidomethyl)-8-hydroxy-4,11b-dimethyltetradecahydro-6a,9-methanocyclohepta[a]naphthalene-4-carboxylate **(20)**

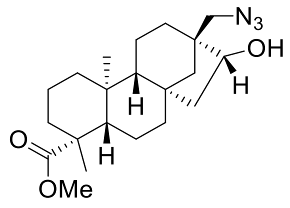

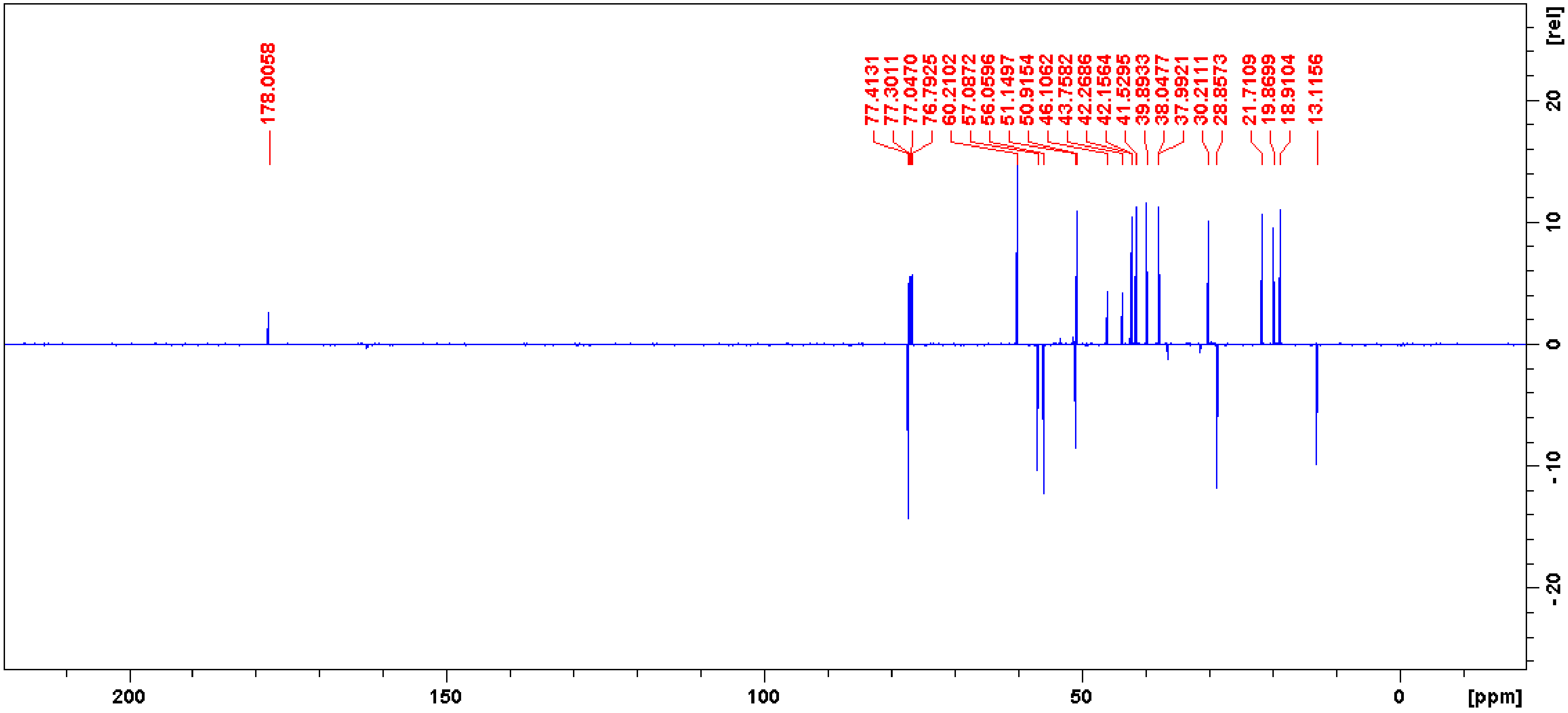

COSY of compound (4*R*,4a*S*,6a*R*,8*R*,9*S*,11a*R*,11b*S*)-methyl 9-(azidomethyl)-8-hydroxy-4,11b-dimethyltetradecahydro-6a,9-methanocyclohepta[a]naphthalene-4-carboxylate **(20)**

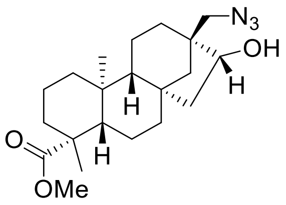

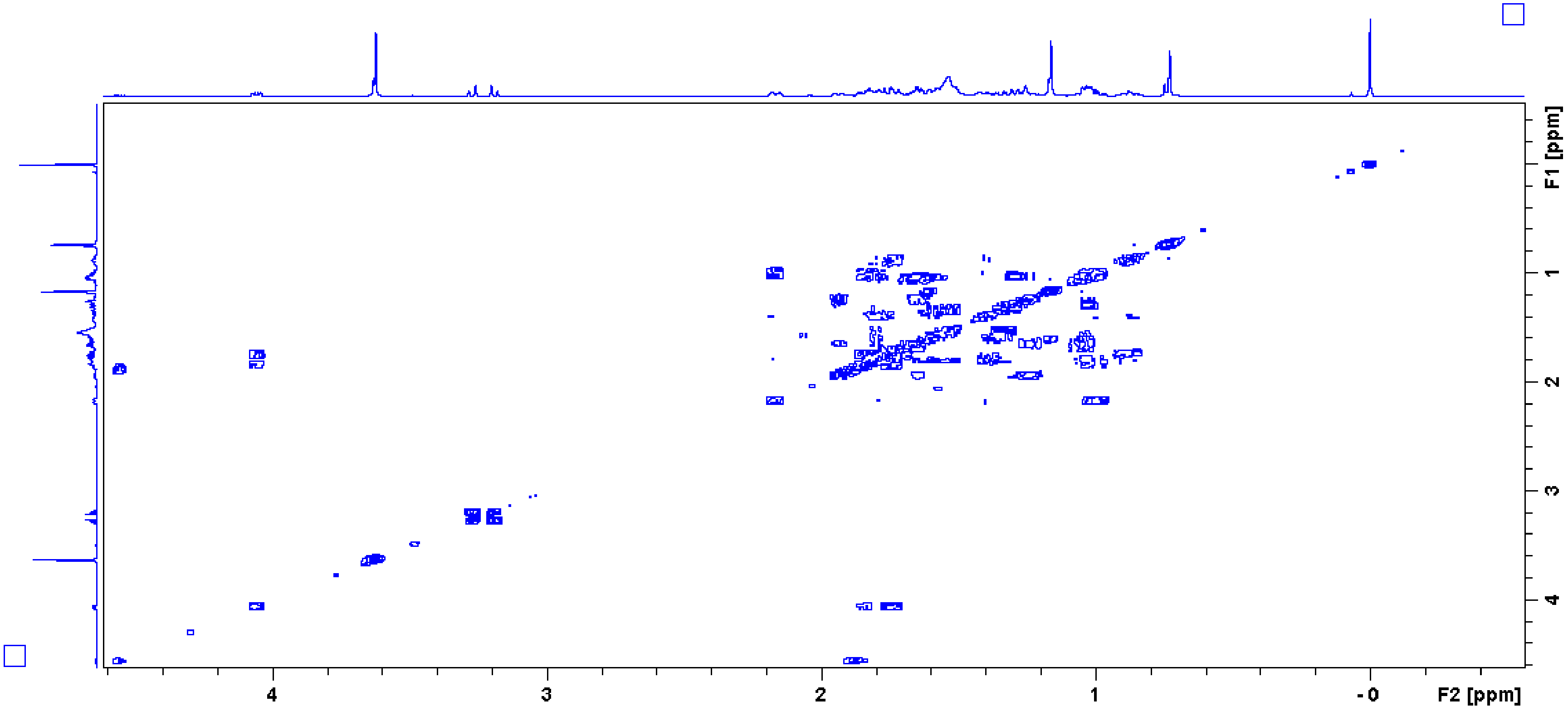

NOESY of compound (4*R*,4a*S*,6a*R*,8*R*,9*S*,11a*R*,11b*S*)-methyl 9-(azidomethyl)-8-hydroxy-4,11b-dimethyltetradecahydro-6a,9-methanocyclohepta[a]naphthalene-4-carboxylate **(20)**

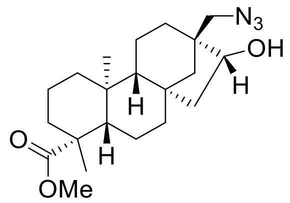

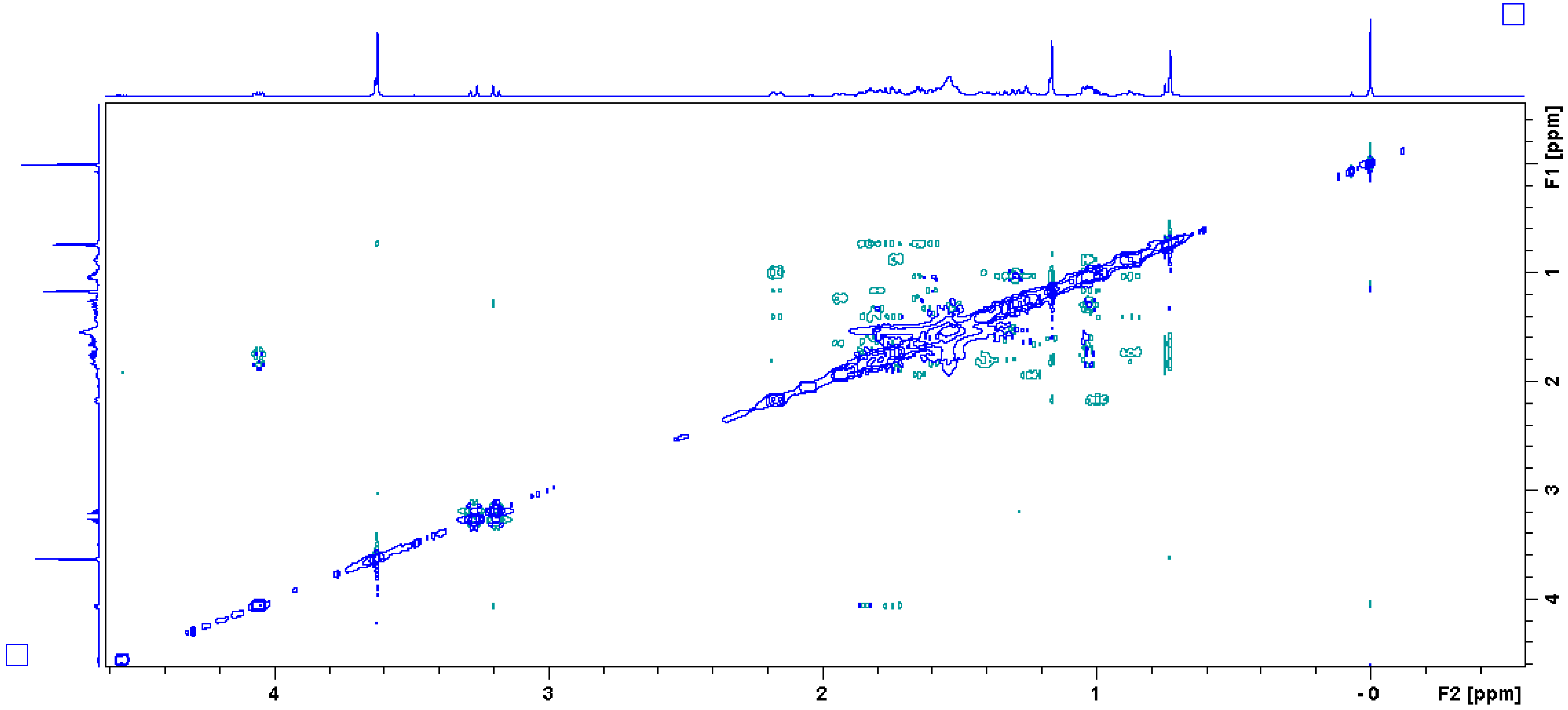

HSQC of compound (4*R*,4a*S*,6a*R*,8*R*,9*S*,11a*R*,11b*S*)-methyl 9-(azidomethyl)-8-hydroxy-4,11b-dimethyltetradecahydro-6a,9-methanocyclohepta[a]naphthalene-4-carboxylate **(20)**

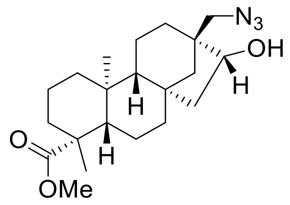

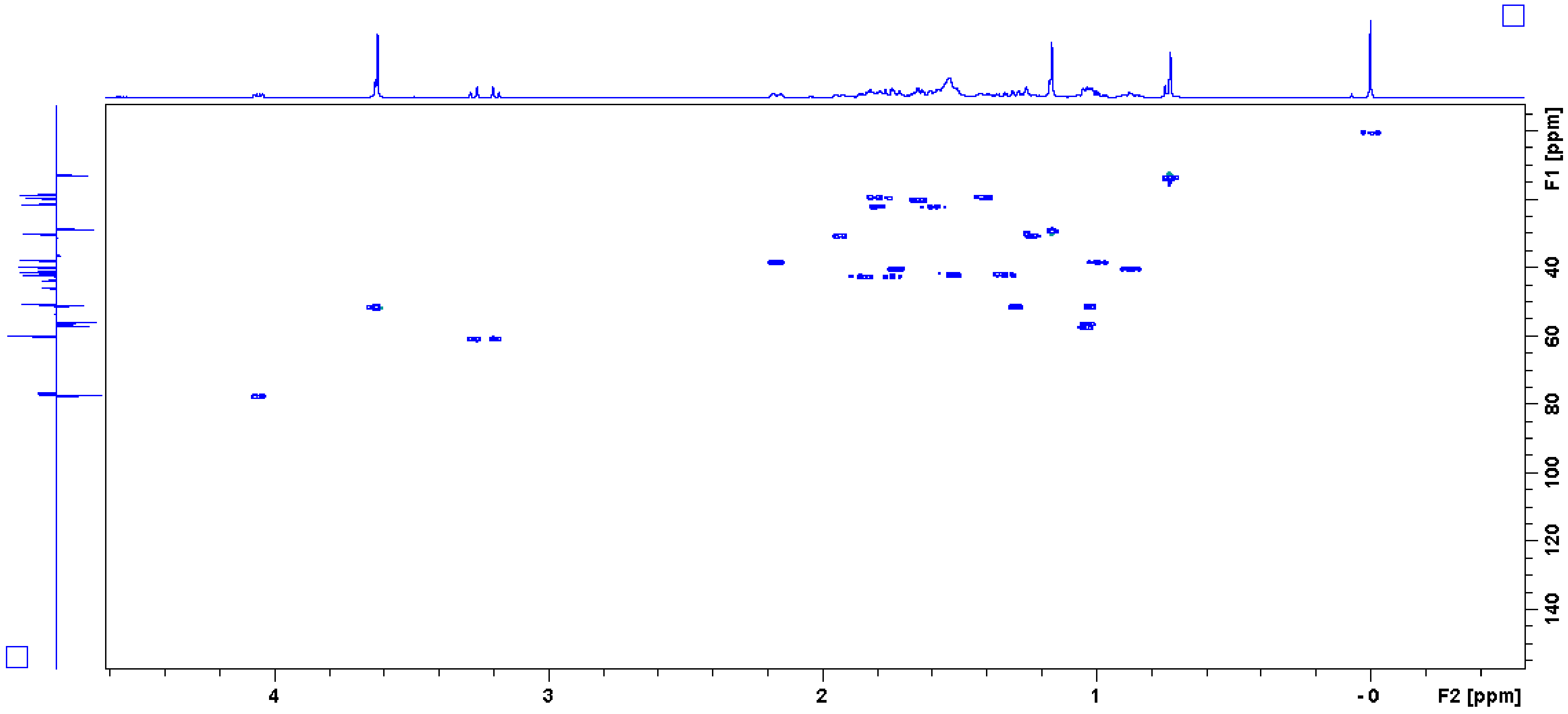

HMBC of compound (4*R*,4a*S*,6a*R*,8*R*,9*S*,11a*R*,11b*S*)-methyl 9-(azidomethyl)-8-hydroxy-4,11b-dimethyltetradecahydro-6a,9-methanocyclohepta[a]naphthalene-4-carboxylate **(20)**

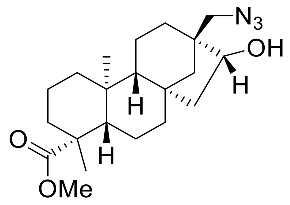

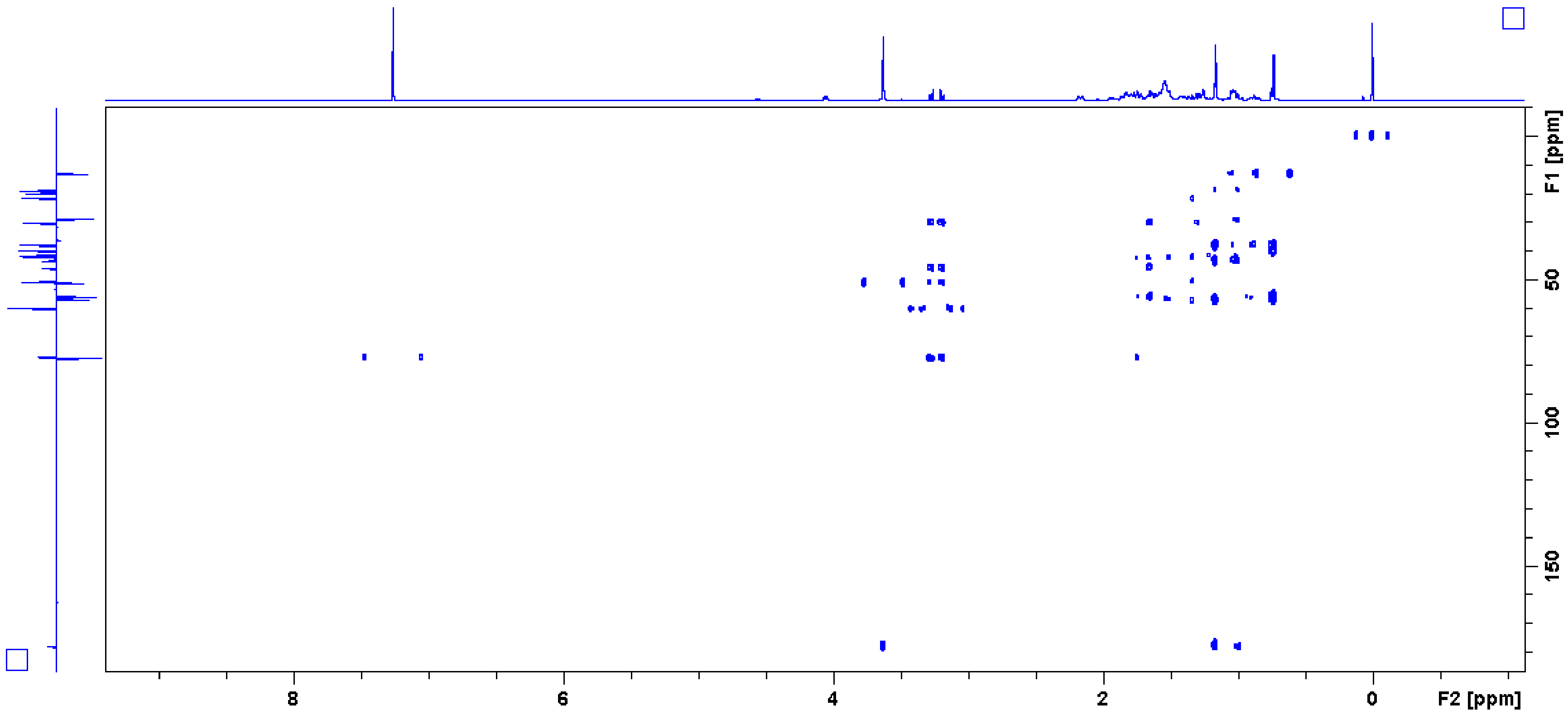

^{1}H-NMR of compound (4*R*,4a*S*,6a*R*,8*R*,9*S*,11a*R*,11b*S*)-methyl 8-hydroxy-4,11b-dimethyl-9-(((methylsulfonyl)oxy)methyl)tetradecahydro-6a,9-methanocyclohepta[a]naphthalene-4-carboxylate **(21)**

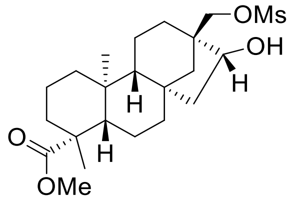

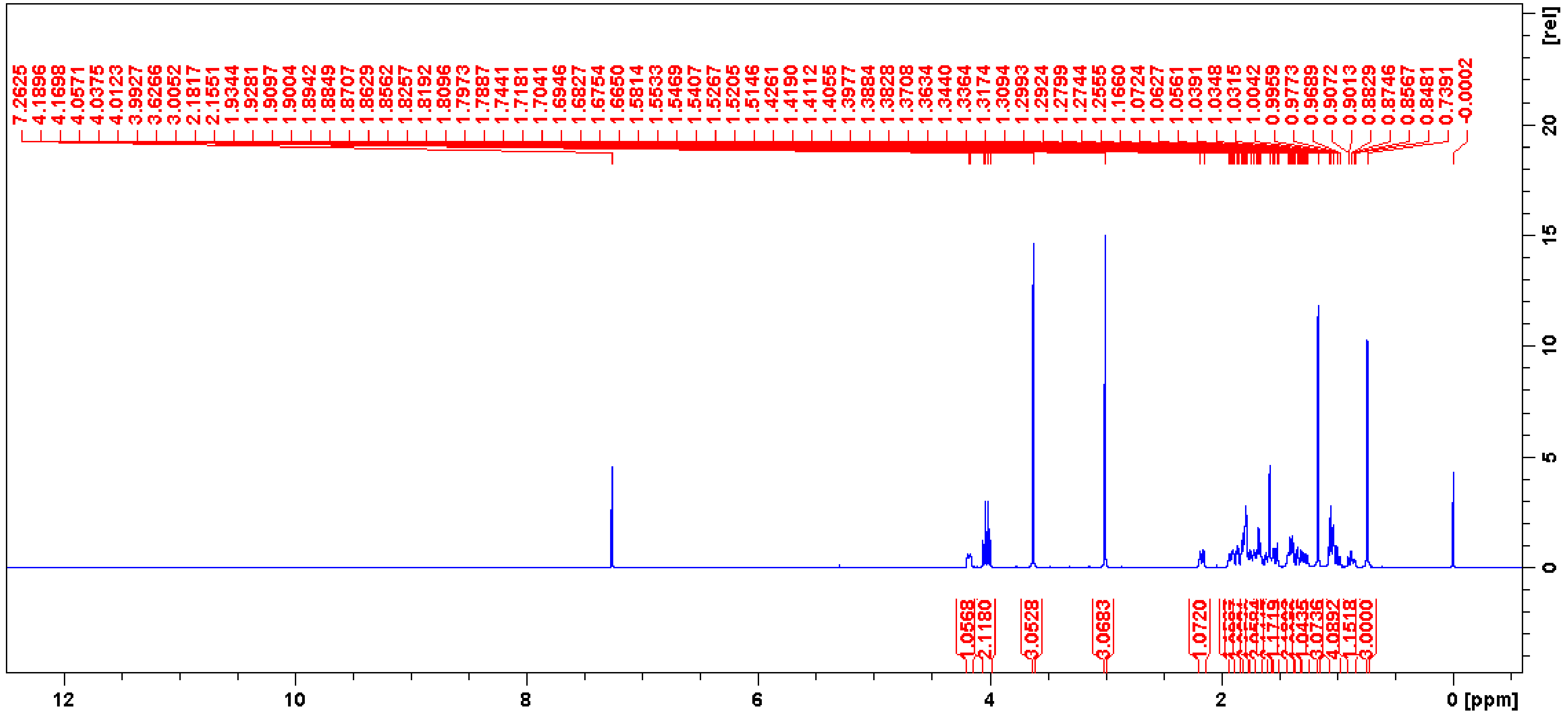

^{13}C-NMR of compound (4*R*,4a*S*,6a*R*,8*R*,9*S*,11a*R*,11b*S*)-methyl 8-hydroxy-4,11b-dimethyl-9-(((methylsulfonyl)oxy)methyl)tetradecahydro-6a,9-methanocyclohepta[a]naphthalene-4-carboxylate **(21)**

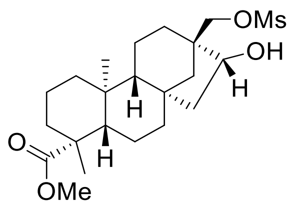

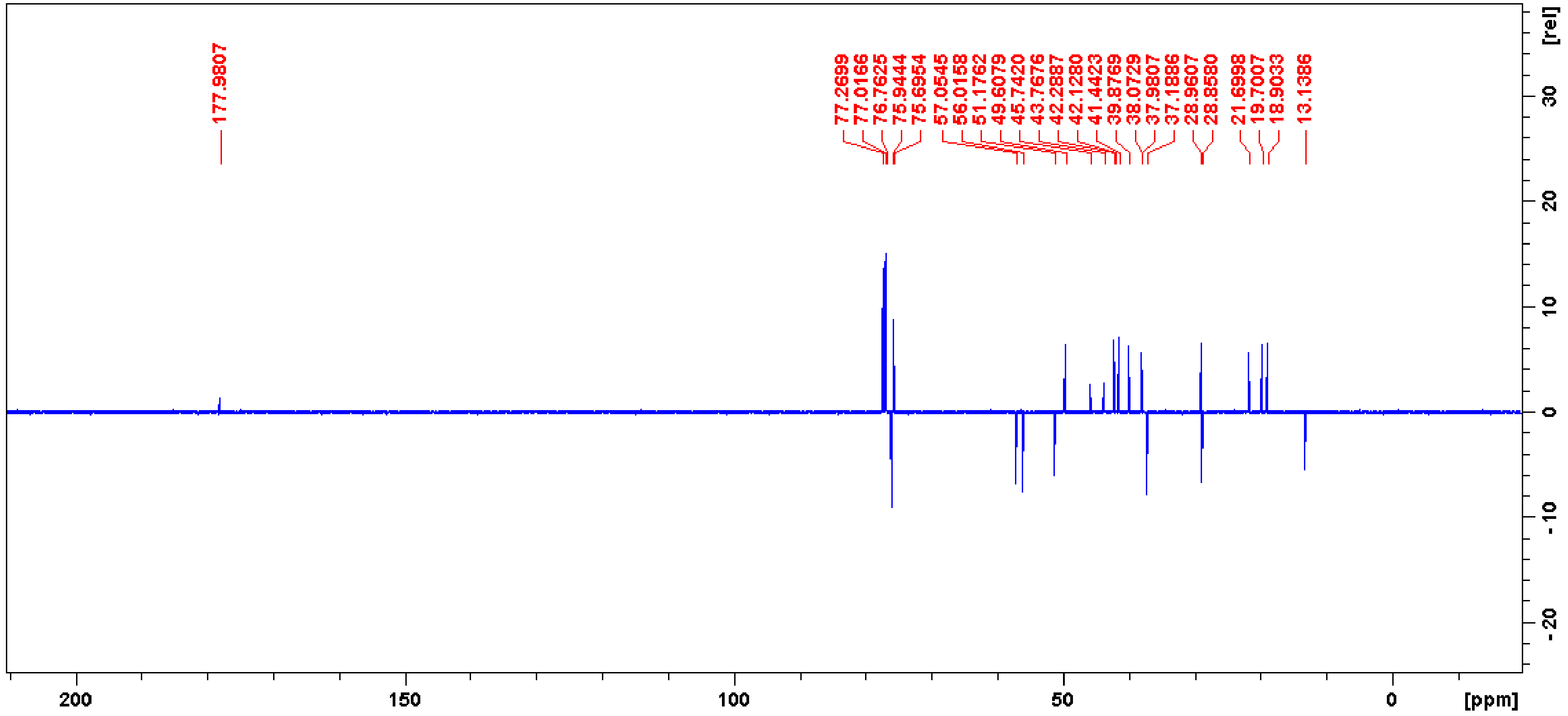

COSY of compound (4*R*,4a*S*,6a*R*,8*R*,9*S*,11a*R*,11b*S*)-methyl 8-hydroxy-4,11b-dimethyl-9-(((methylsulfonyl)oxy)methyl)tetradecahydro-6a,9-methanocyclohepta[a]naphthalene-4-carboxylate **(21)**

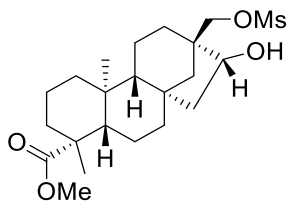

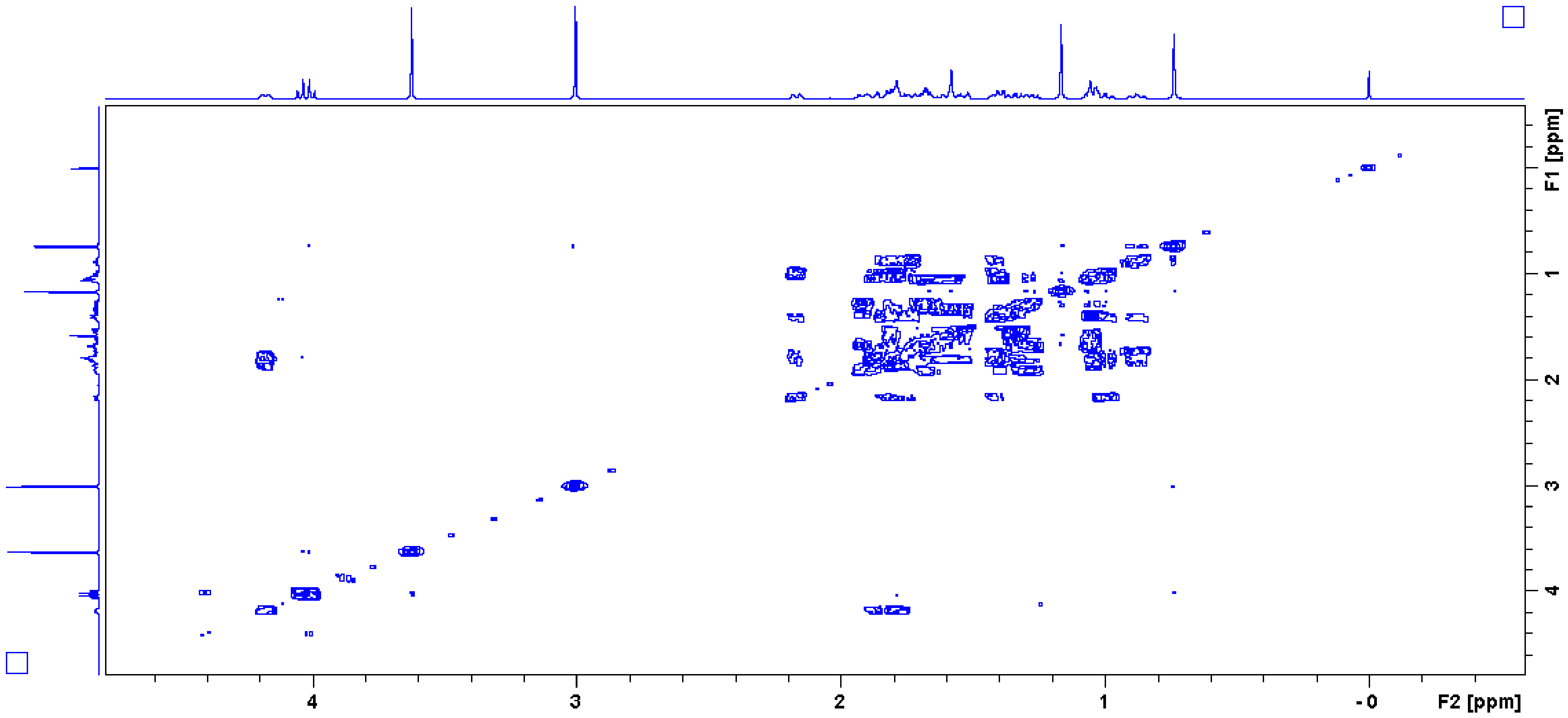

NOESY of compound (4*R*,4a*S*,6a*R*,8*R*,9*S*,11a*R*,11b*S*)-methyl 8-hydroxy-4,11b-dimethyl-9-(((methylsulfonyl)oxy)methyl)tetradecahydro-6a,9-methanocyclohepta[a]naphthalene-4-carboxylate **(21)**

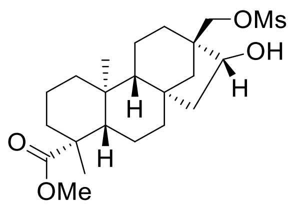

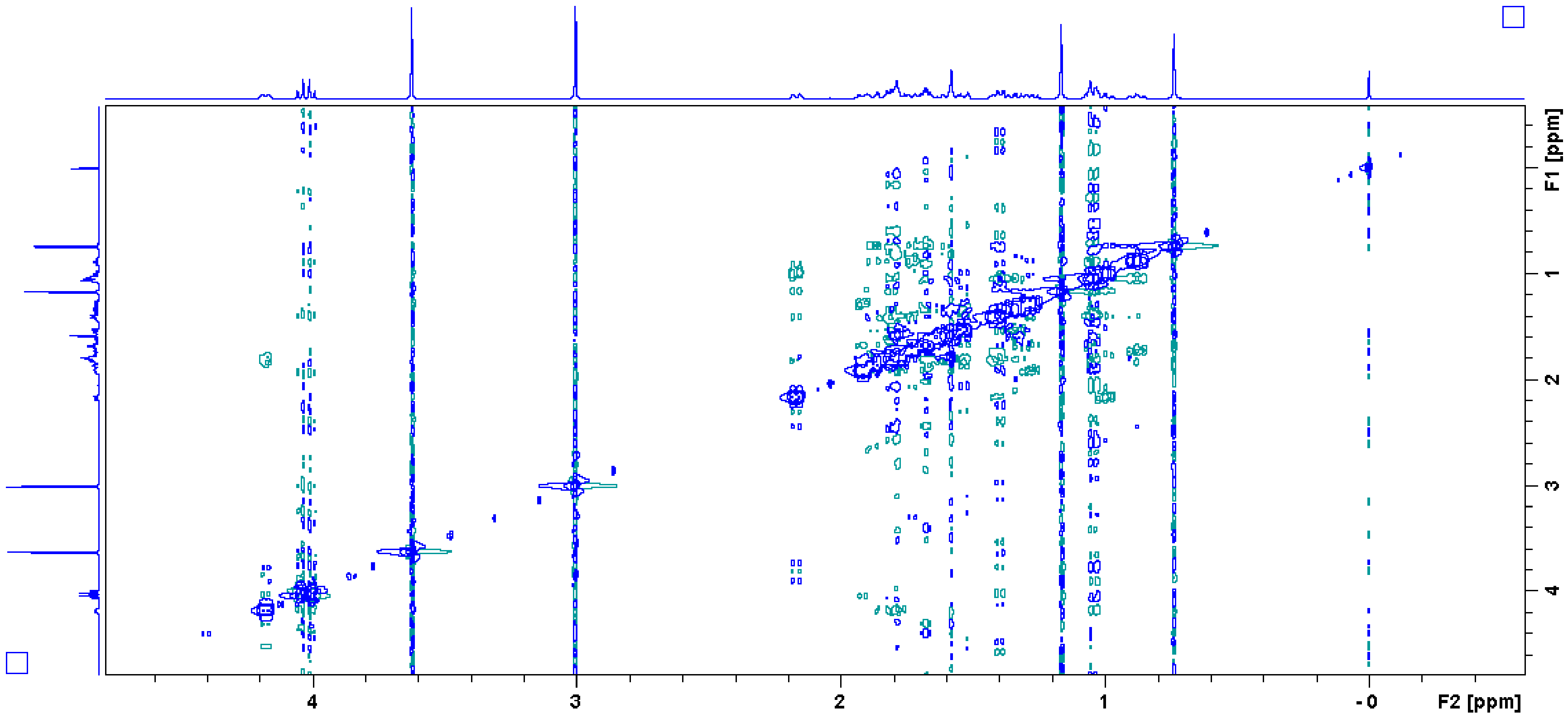

HSQC of compound (4*R*,4a*S*,6a*R*,8*R*,9*S*,11a*R*,11b*S*)-methyl 8-hydroxy-4,11b-dimethyl-9-(((methylsulfonyl)oxy)methyl)tetradecahydro-6a,9-methanocyclohepta[a]naphthalene-4-carboxylate **(21)**

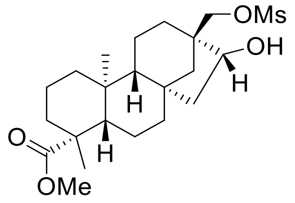

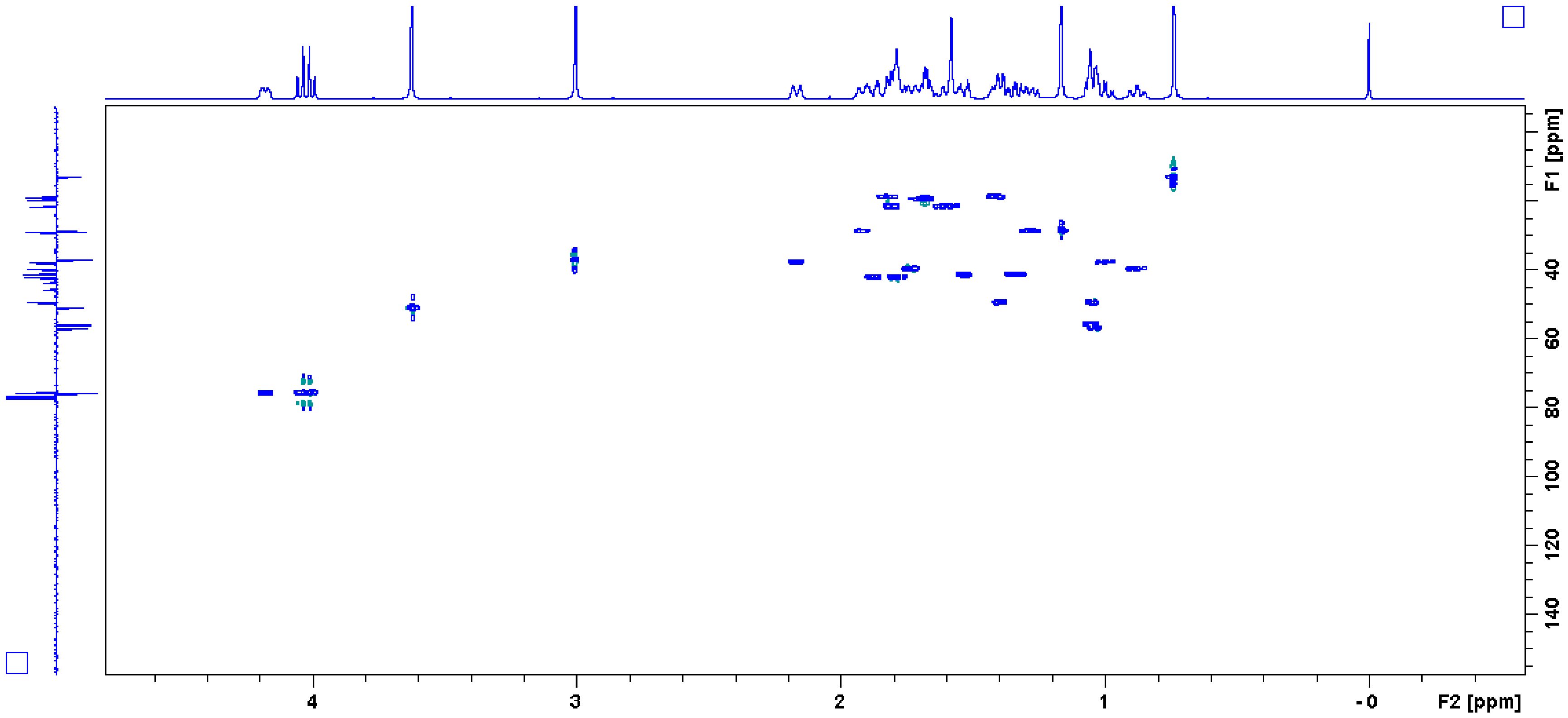

HMBC of compound (4*R*,4a*S*,6a*R*,8*R*,9*S*,11a*R*,11b*S*)-methyl 8-hydroxy-4,11b-dimethyl-9-(((methylsulfonyl)oxy)methyl)tetradecahydro-6a,9-methanocyclohepta[a]naphthalene-4-carboxylate **(21)**

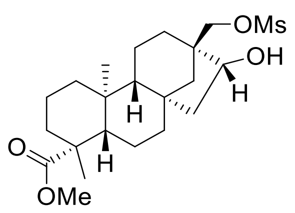

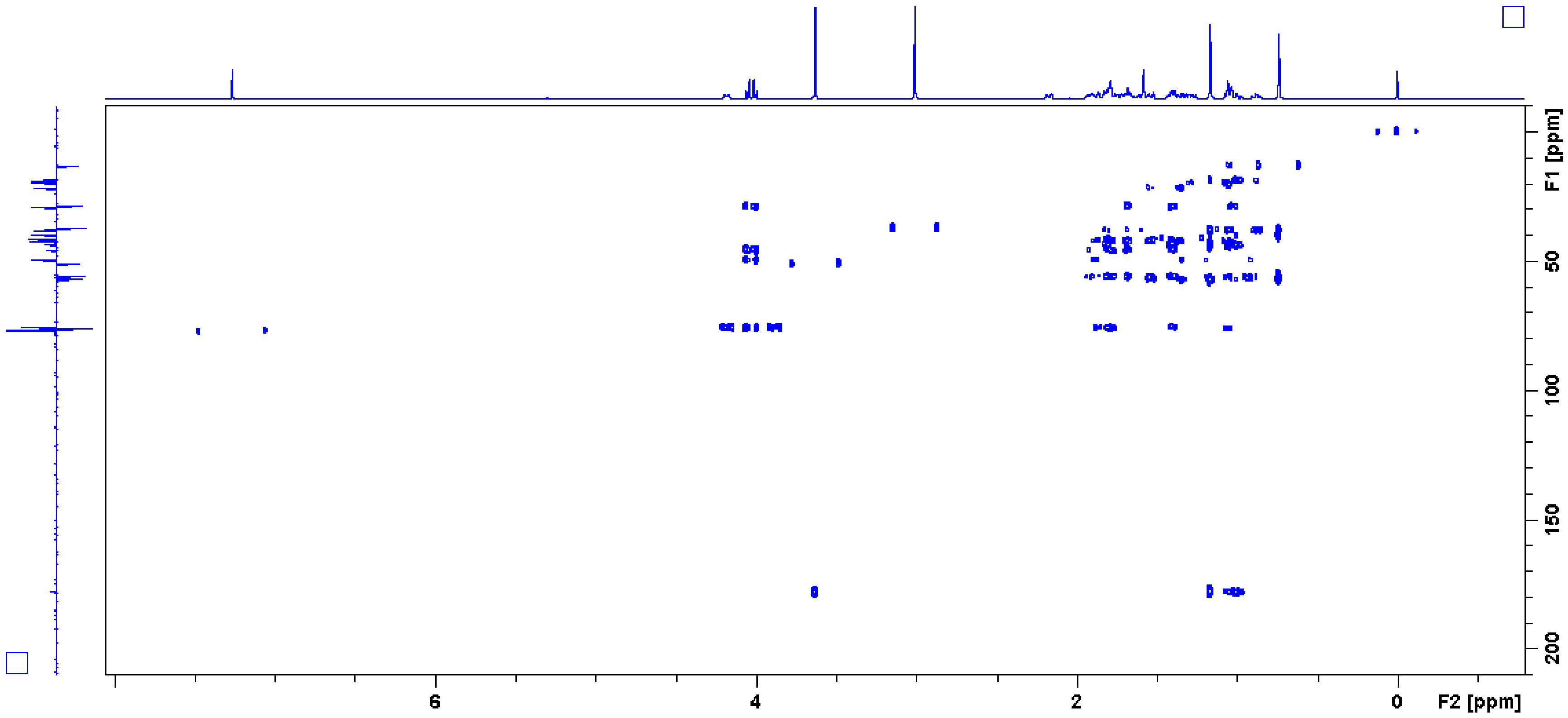

^{1}H-NMR of compound (4*R*,4a*S*,6a*R*,8*R*,9*S*,11a*R*,11b*S*)-methyl 9-((benzylamino)methyl)-8-hydroxy-4,11b-dimethyltetradecahydro-6a,9-methanocyclohepta[a]naphthalene-4-carboxylate **(22)**

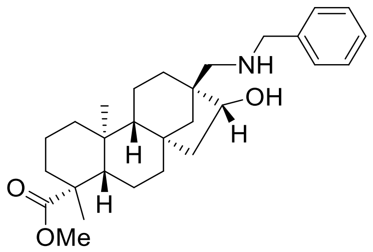

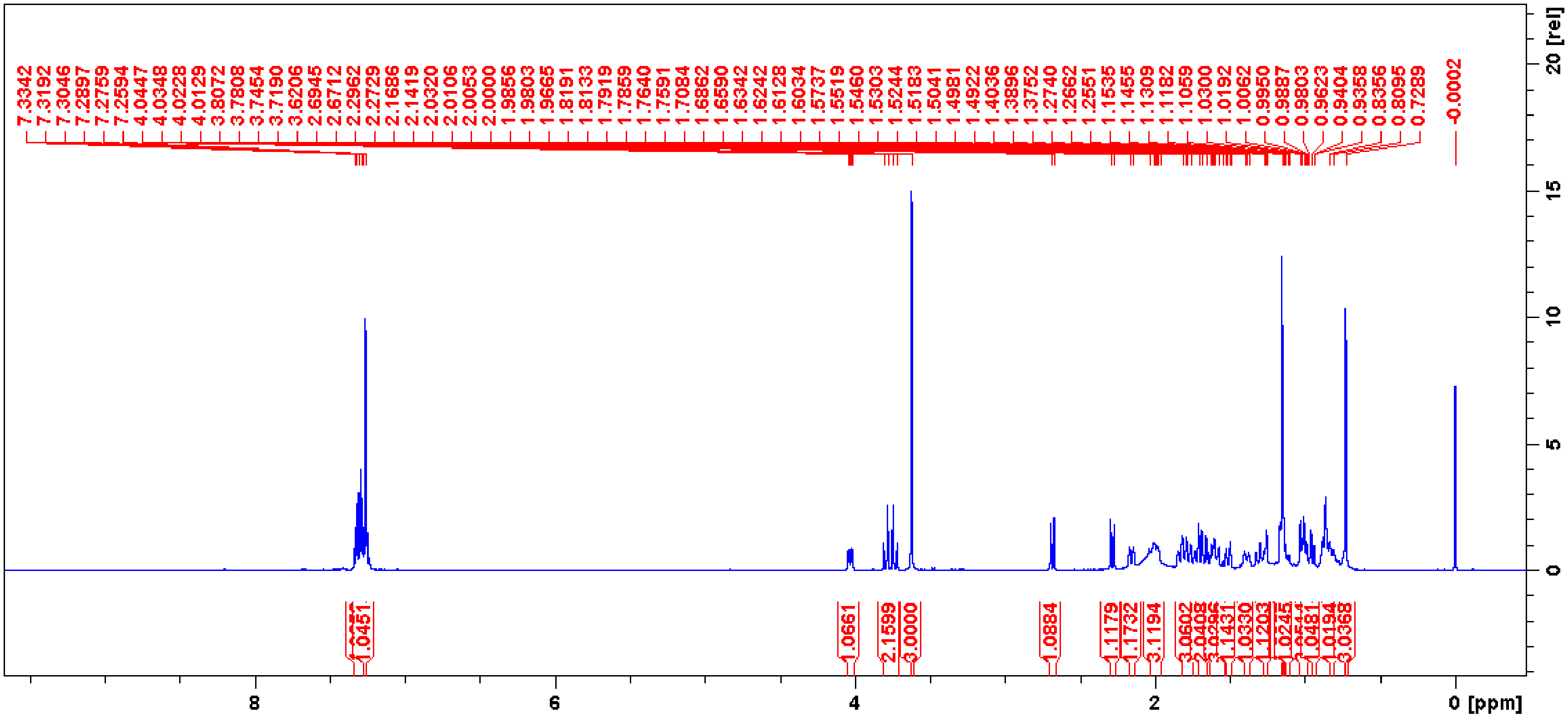

^{13}C-NMR of compound (4*R*,4a*S*,6a*R*,8*R*,9*S*,11a*R*,11b*S*)-methyl 9-((benzylamino)methyl)-8-hydroxy-4,11b-dimethyltetradecahydro-6a,9-methanocyclohepta[a]naphthalene-4-carboxylate **(22)**

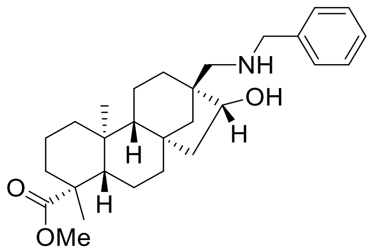

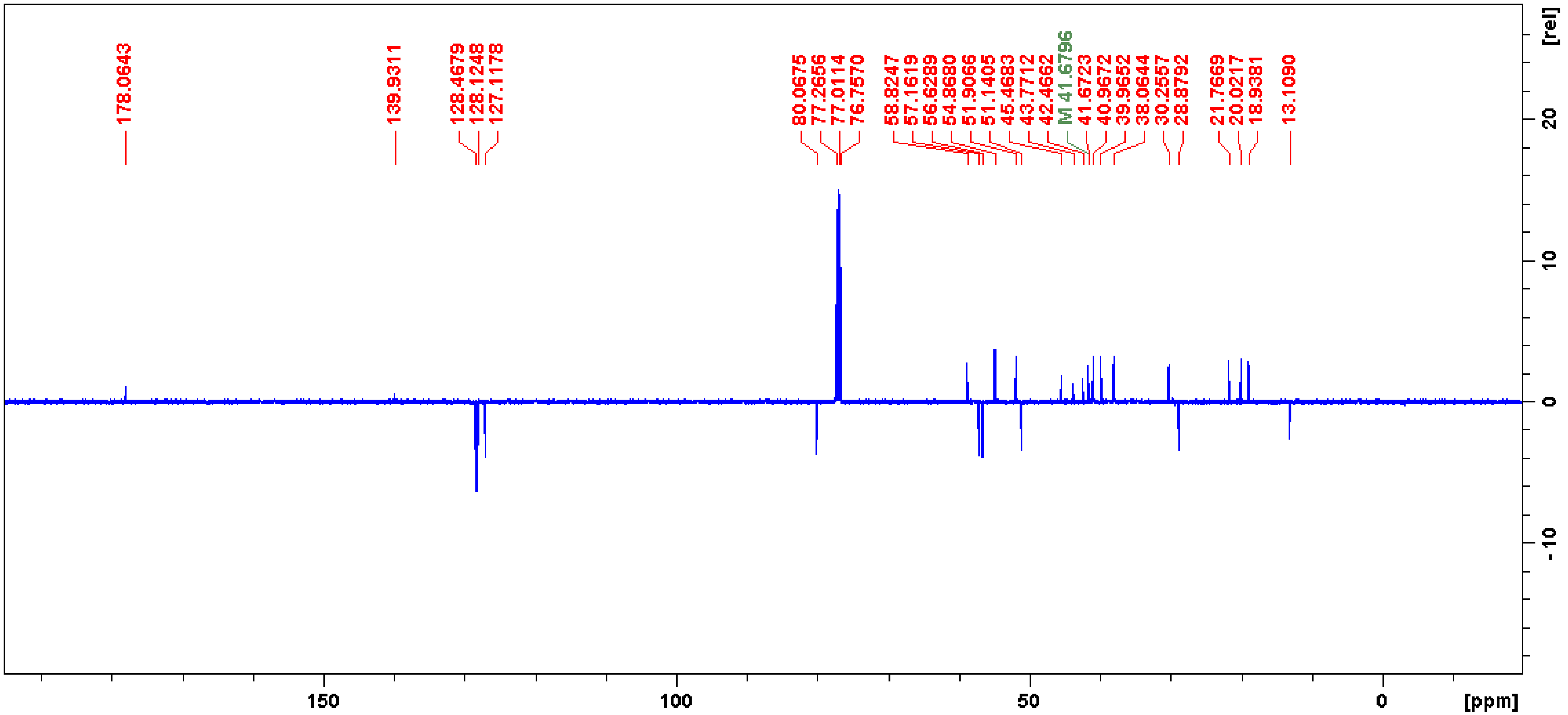

COSY of compound (4*R*,4a*S*,6a*R*,8*R*,9*S*,11a*R*,11b*S*)-methyl 9-((benzylamino)methyl)-8-hydroxy-4,11b-dimethyltetradecahydro-6a,9-methanocyclohepta[a]naphthalene-4-carboxylate **(22)**

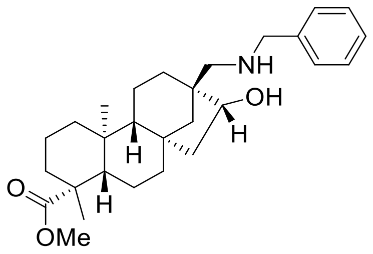

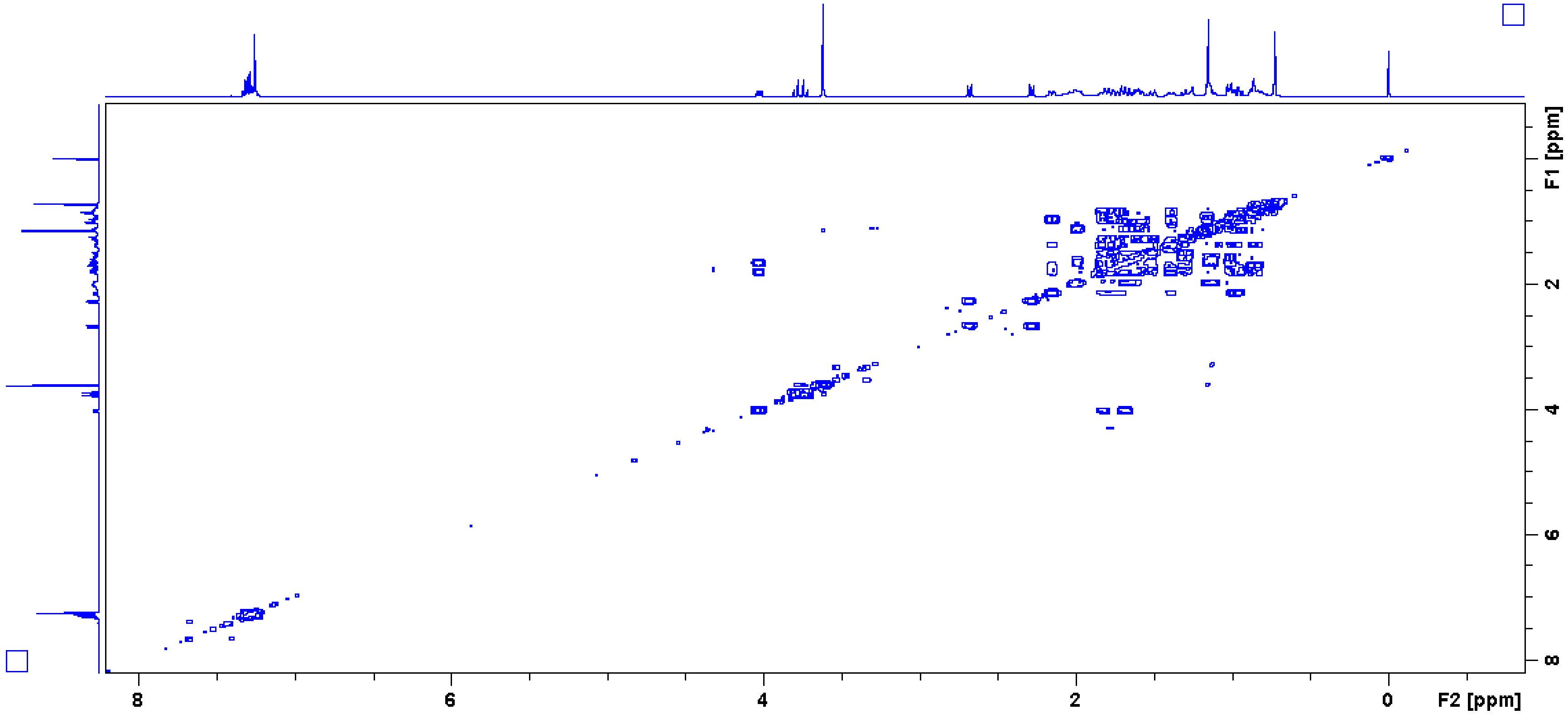

NOESY of compound (4*R*,4a*S*,6a*R*,8*R*,9*S*,11a*R*,11b*S*)-methyl 9-((benzylamino)methyl)-8-hydroxy-4,11b-dimethyltetradecahydro-6a,9-methanocyclohepta[a]naphthalene-4-carboxylate **(22)**

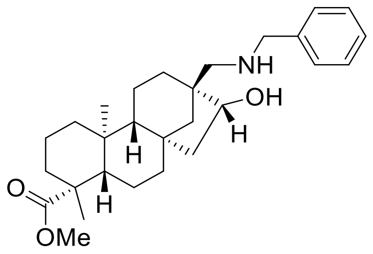

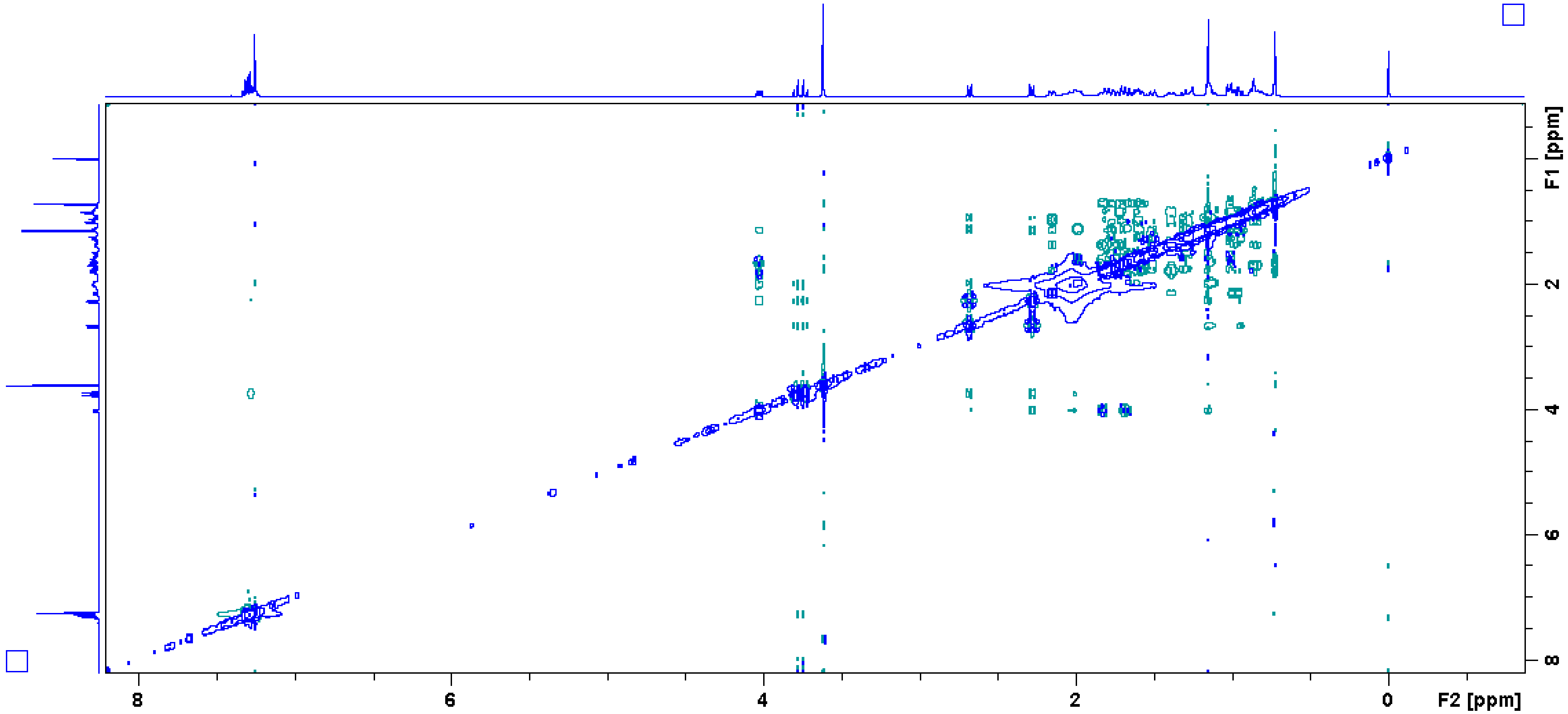

HSQC of compound (4*R*,4a*S*,6a*R*,8*R*,9*S*,11a*R*,11b*S*)-methyl 9-((benzylamino)methyl)-8-hydroxy-4,11b-dimethyltetradecahydro-6a,9-methanocyclohepta[a]naphthalene-4-carboxylate **(22)**

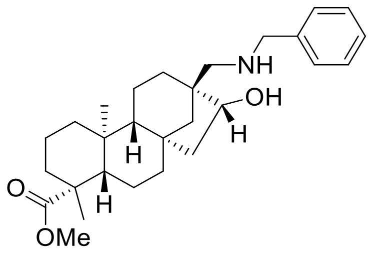

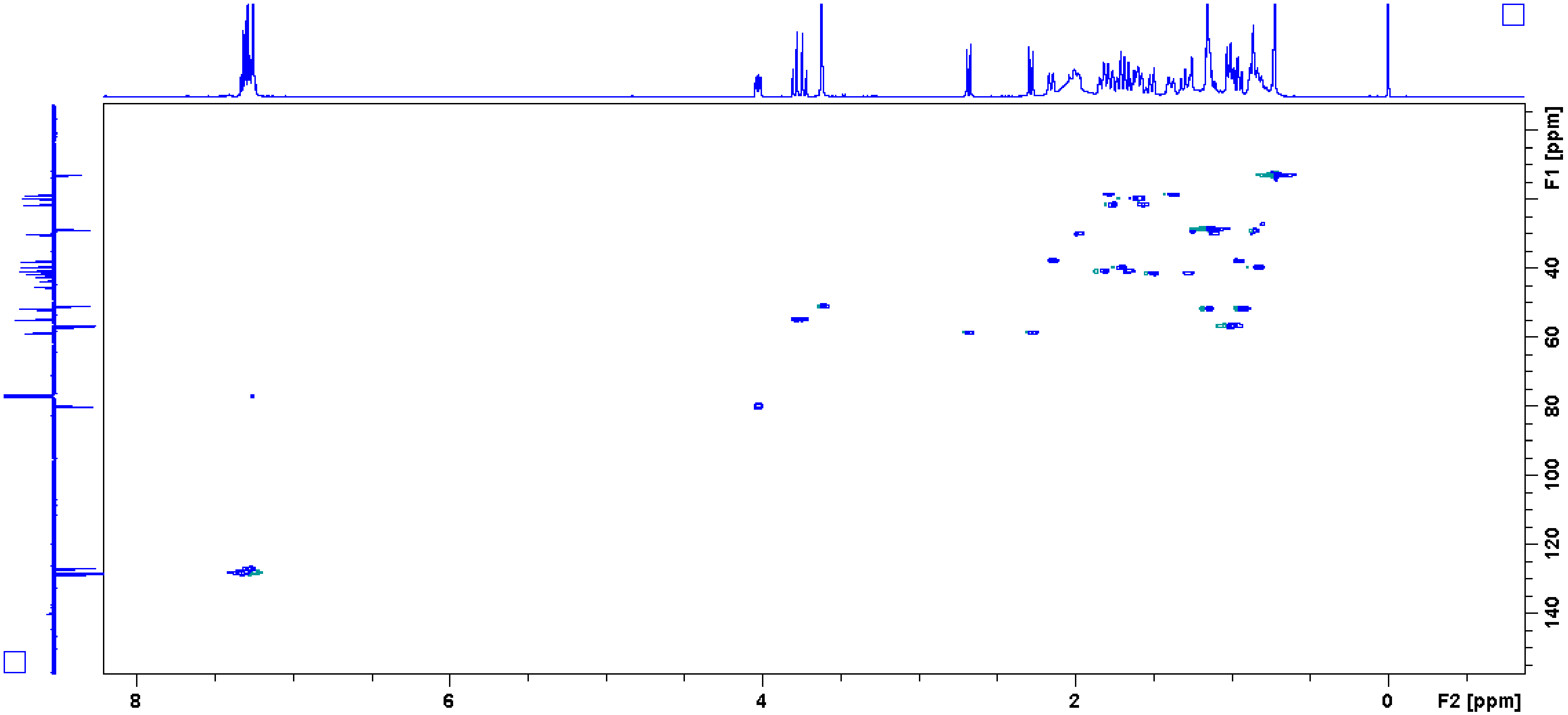

HMBC of compound (4*R*,4a*S*,6a*R*,8*R*,9*S*,11a*R*,11b*S*)-methyl 9-((benzylamino)methyl)-8-hydroxy-4,11b-dimethyltetradecahydro-6a,9-methanocyclohepta[a]naphthalene-4-carboxylate **(22)**

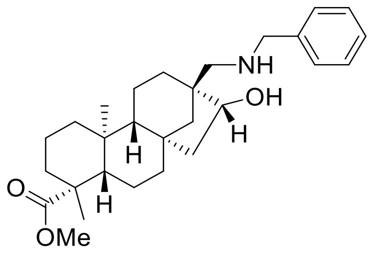

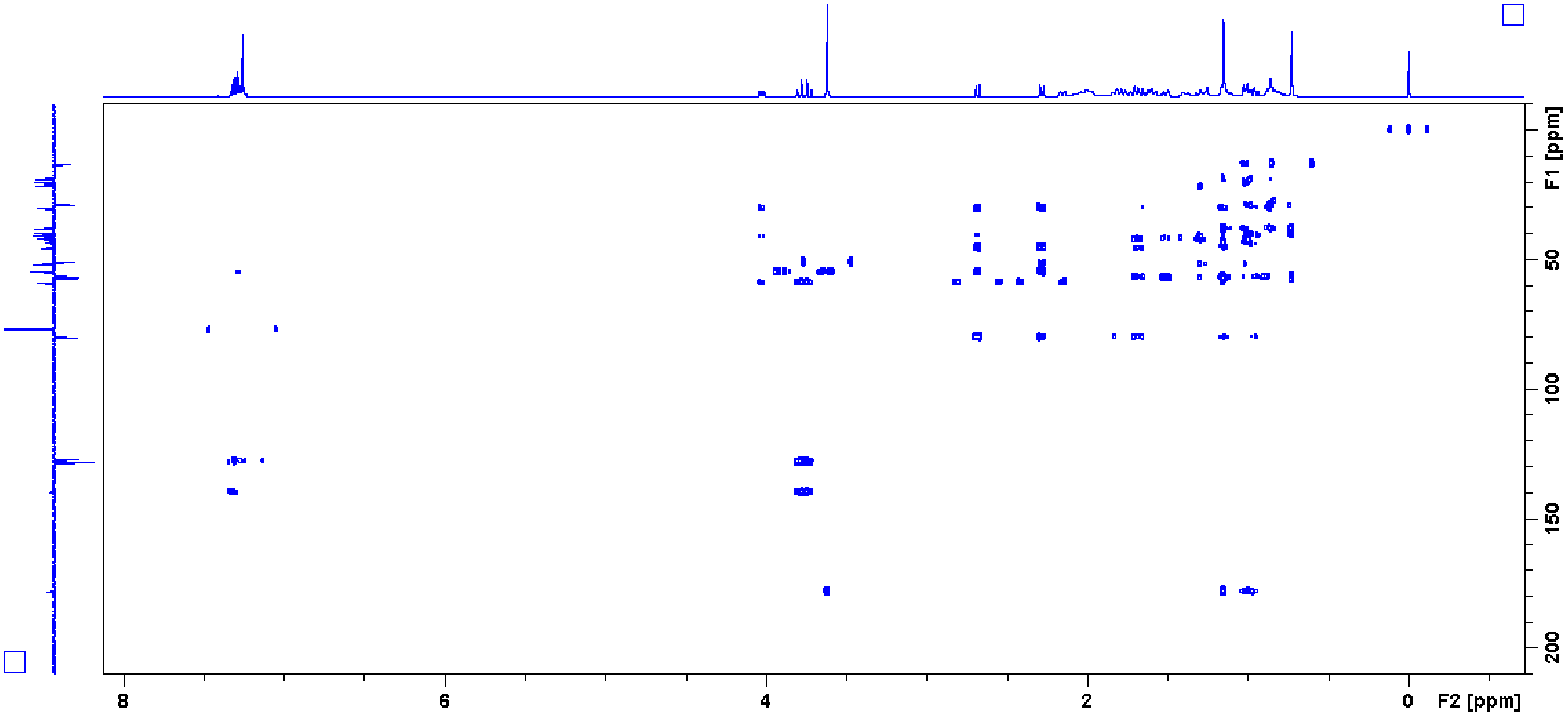

^{1}H-NMR of compound (4*R*,4a*S*,6a*R*,8*R*,9*S*,11a*R*,11b*S*)-methyl 9-(((4-fluorobenzyl)amino)methyl)-8-hydroxy-4,11b-dimethyltetradecahydro-6a,9-methanocyclohepta[a]naphthalene-4-carboxylate **(23)**

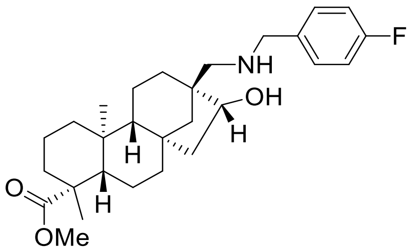

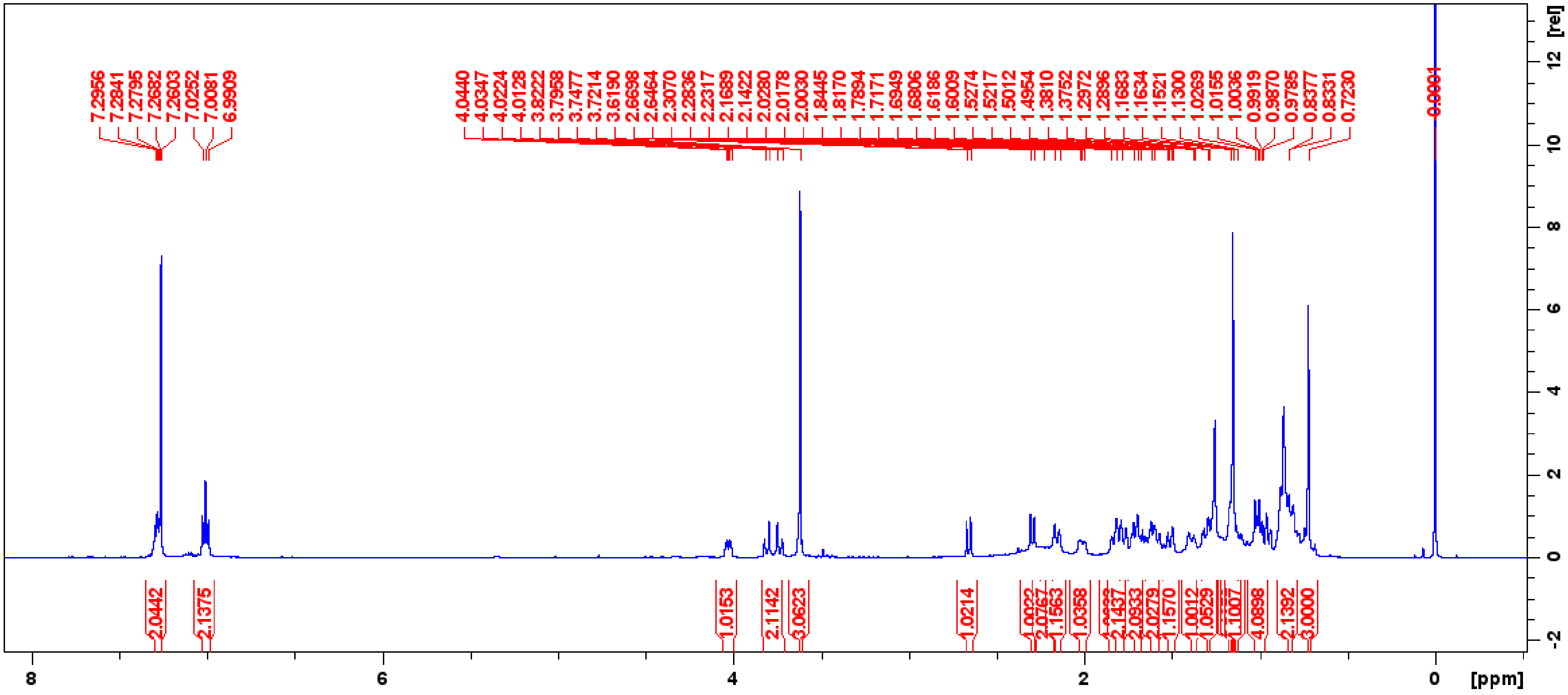

^{13}C-NMR of compound (4*R*,4a*S*,6a*R*,8*R*,9*S*,11a*R*,11b*S*)-methyl 9-(((4-fluorobenzyl)amino)methyl)-8-hydroxy-4,11b-dimethyltetradecahydro-6a,9-methanocyclohepta[a]naphthalene-4-carboxylate **(23)**

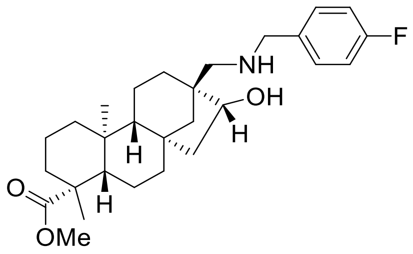

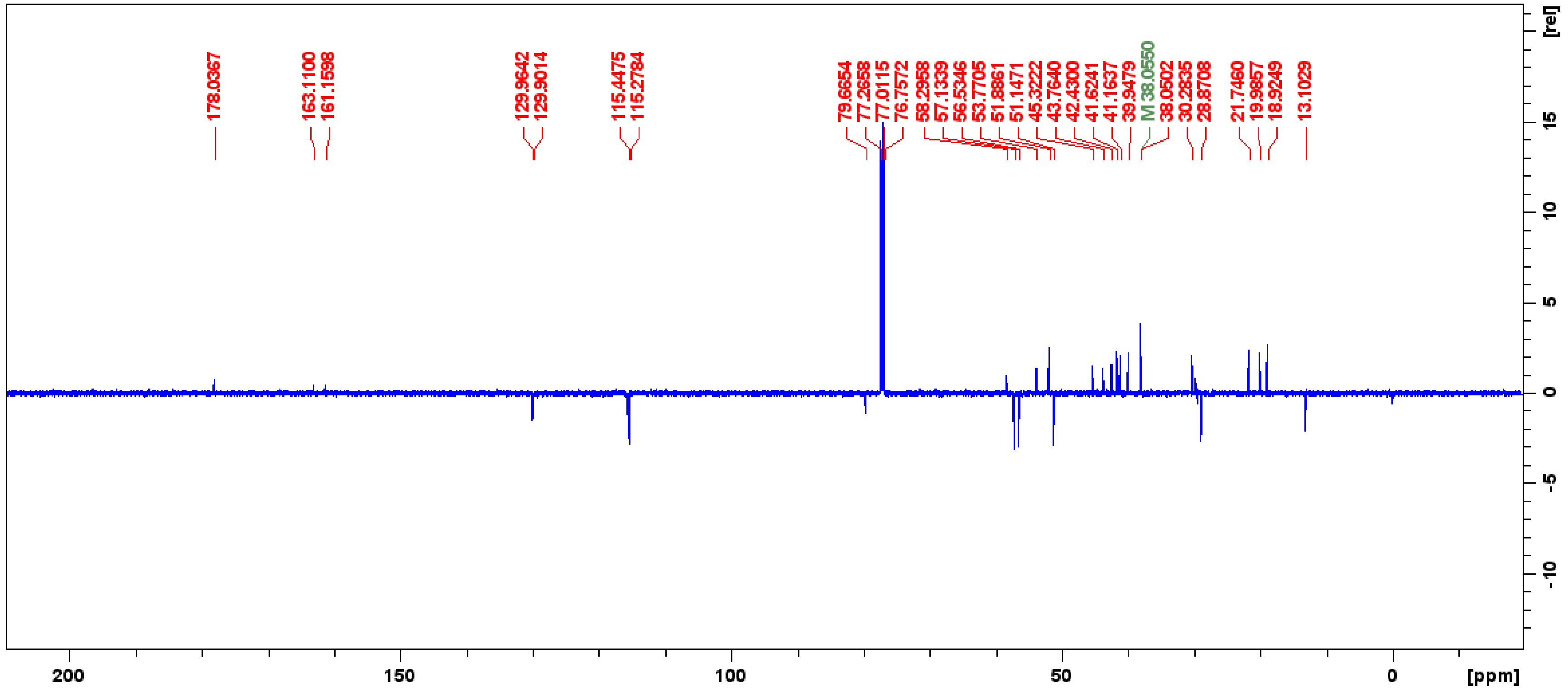

COSY of compound (4*R*,4a*S*,6a*R*,8*R*,9*S*,11a*R*,11b*S*)-methyl 9-(((4-fluorobenzyl)amino)methyl)-8-hydroxy-4,11b-dimethyltetradecahydro-6a,9-methanocyclohepta[a]naphthalene-4-carboxylate **(23)**

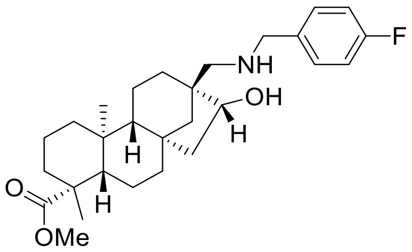

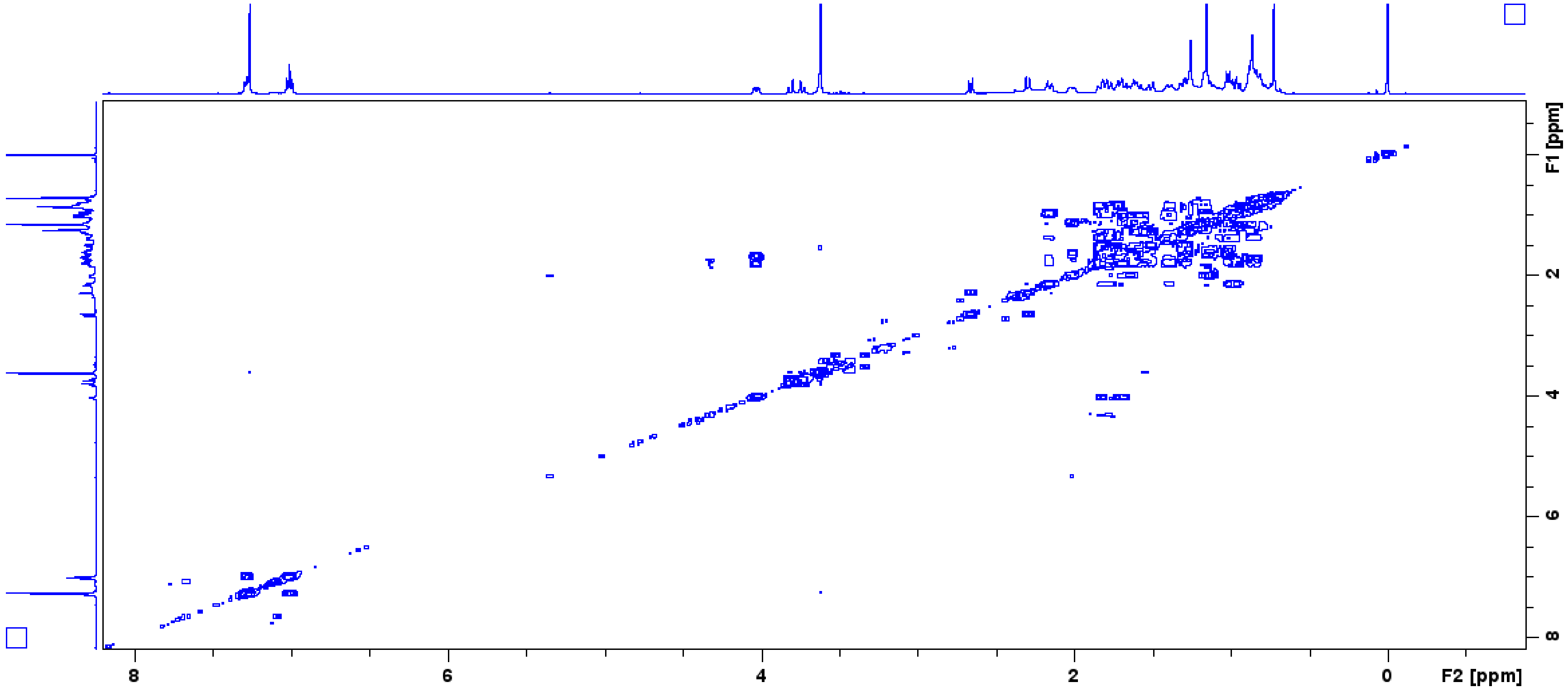

NOESY of compound (4*R*,4a*S*,6a*R*,8*R*,9*S*,11a*R*,11b*S*)-methyl 9-(((4-fluorobenzyl)amino)methyl)-8-hydroxy-4,11b-dimethyltetradecahydro-6a,9-methanocyclohepta[a]naphthalene-4-carboxylate **(23)**

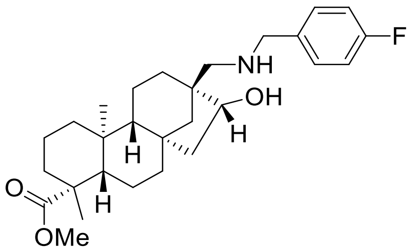

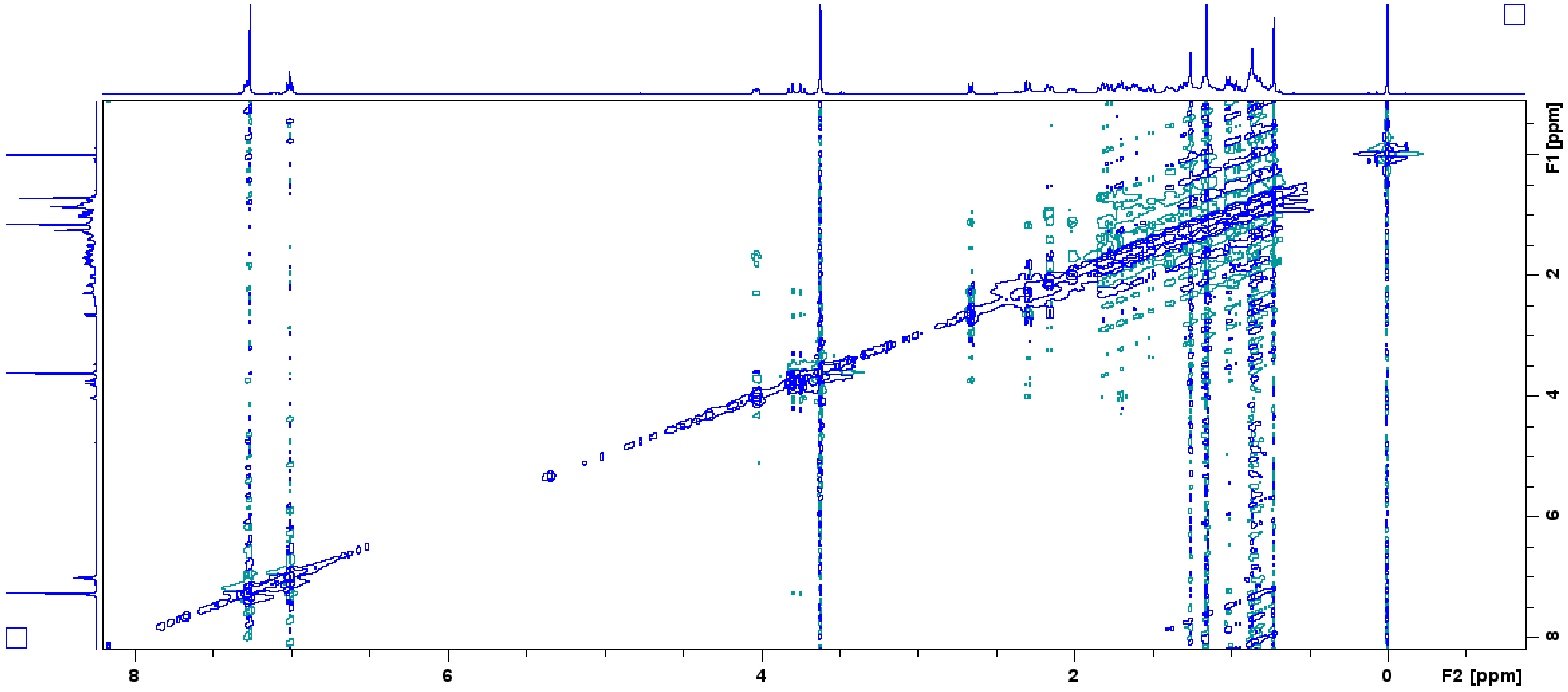

HSQC of compound (4*R*,4a*S*,6a*R*,8*R*,9*S*,11a*R*,11b*S*)-methyl 9-(((4-fluorobenzyl)amino)methyl)-8-hydroxy-4,11b-dimethyltetradecahydro-6a,9-methanocyclohepta[a]naphthalene-4-carboxylate **(23)**

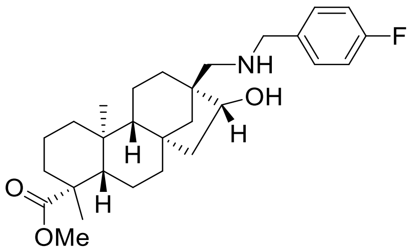

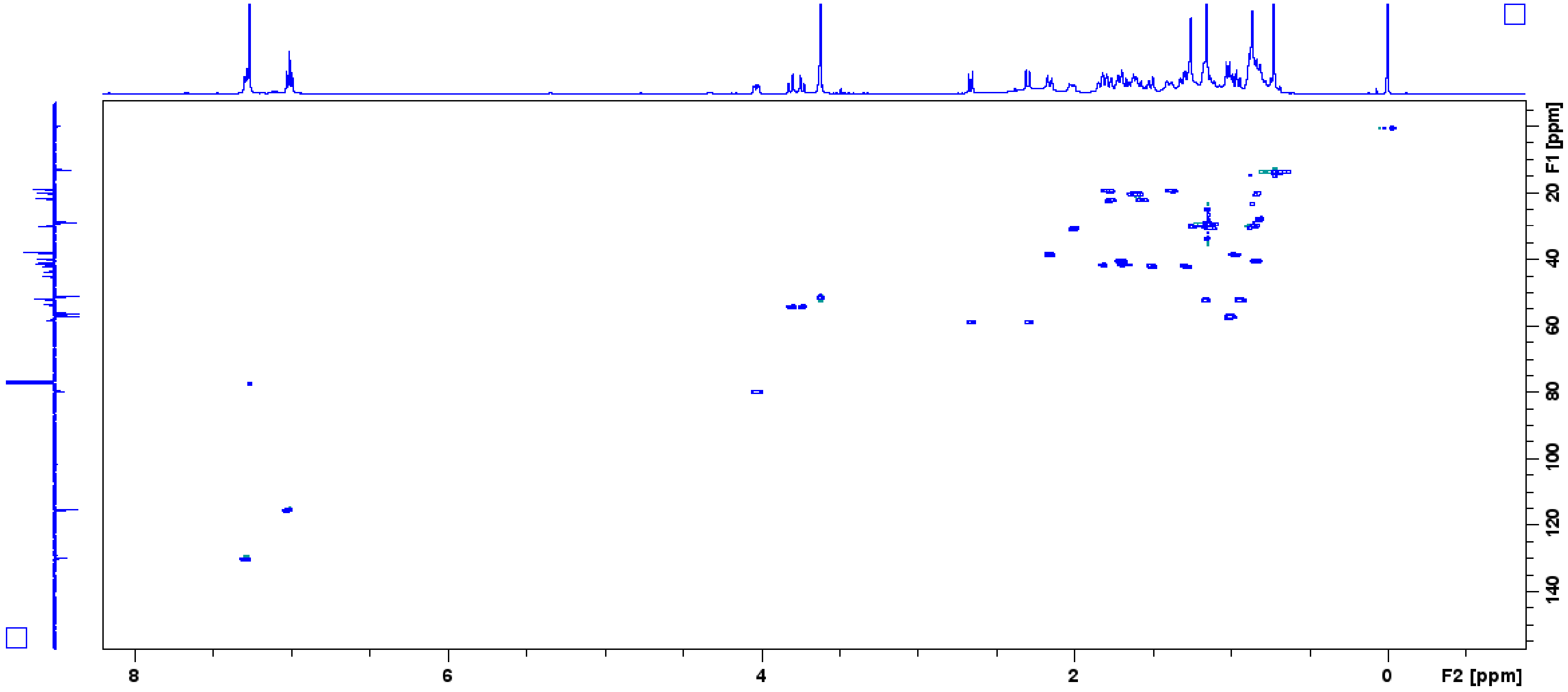

HMBC of compound (4*R*,4a*S*,6a*R*,8*R*,9*S*,11a*R*,11b*S*)-methyl 9-(((4-fluorobenzyl)amino)methyl)-8-hydroxy-4,11b-dimethyltetradecahydro-6a,9-methanocyclohepta[a]naphthalene-4-carboxylate **(23)**

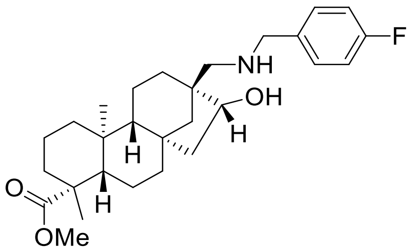

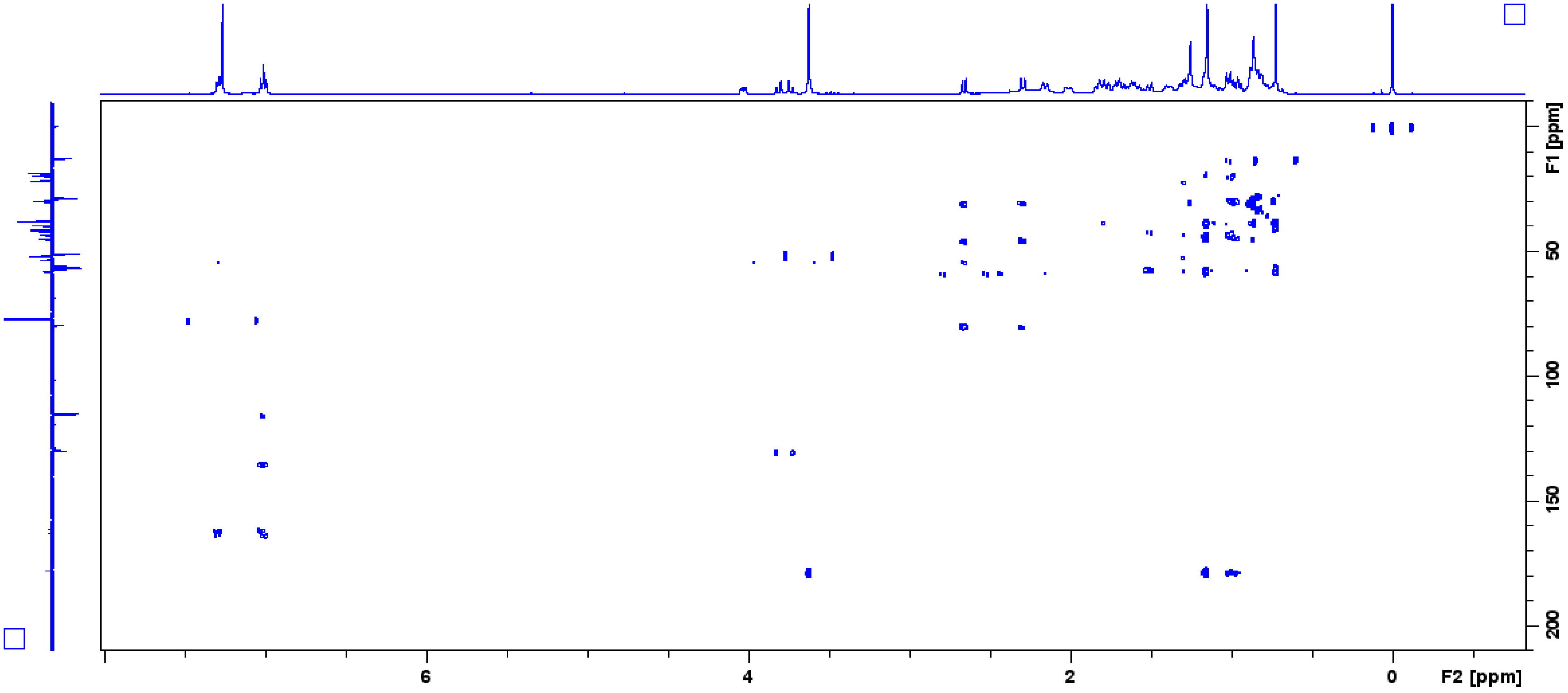

19F-NMR of compound (4*R*,4a*S*,6a*R*,8*R*,9*S*,11a*R*,11b*S*)-methyl 9-(((4-fluorobenzyl)amino)methyl)-8-hydroxy-4,11b-dimethyltetradecahydro-6a,9-methanocyclohepta[a]naphthalene-4-carboxylate **(23)**

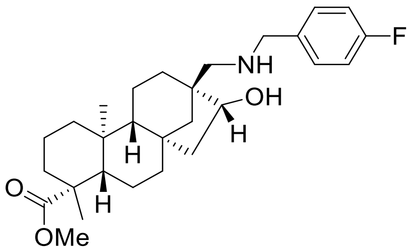

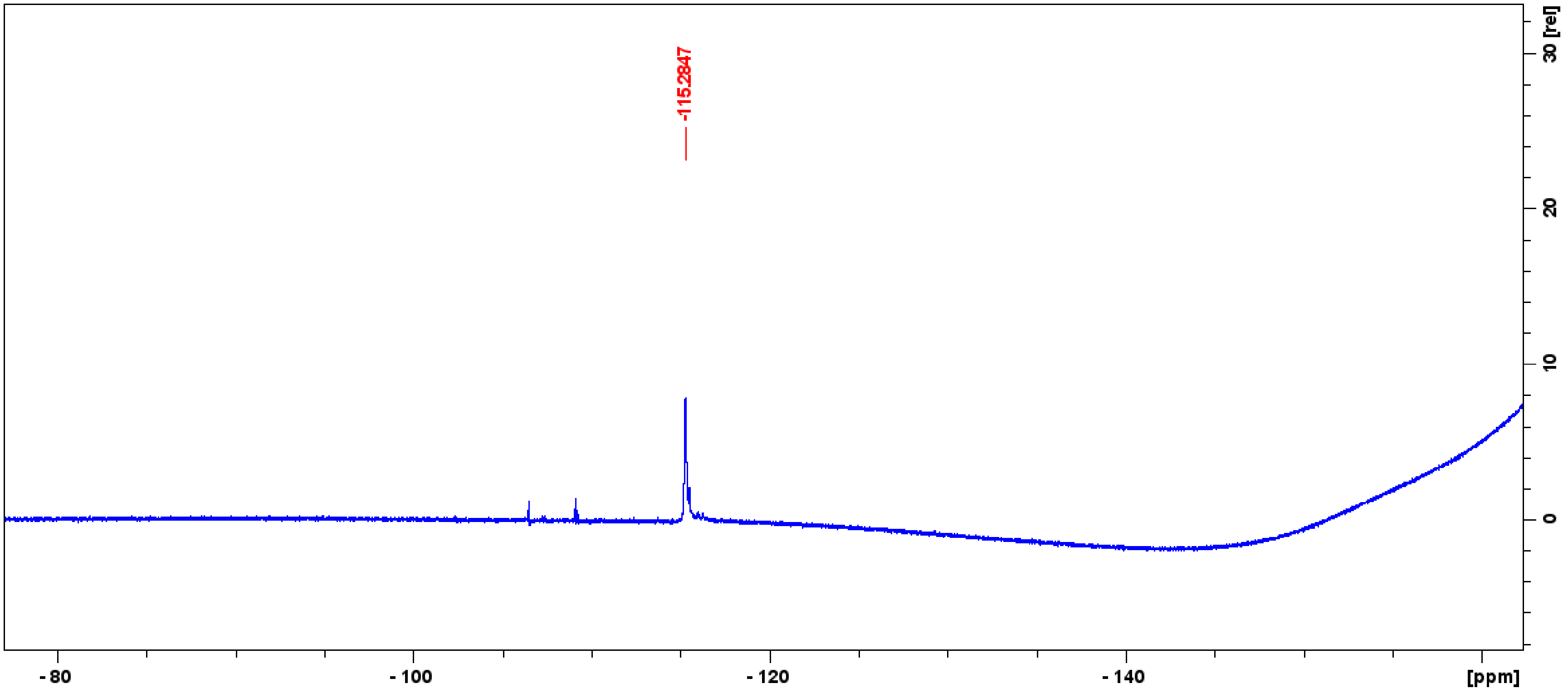

^{1}H-NMR of compound (4*R*,4a*S*,6a*R*,8*R*,9*S*,11a*R*,11b*S*)-methyl 8-hydroxy-4,11b-dimethyl-9-((((*R*)-1-phenylpropyl)amino)methyl)tetradecahydro-6a,9-methanocyclohepta[a]naphthalene-4-carboxylate **(24)**

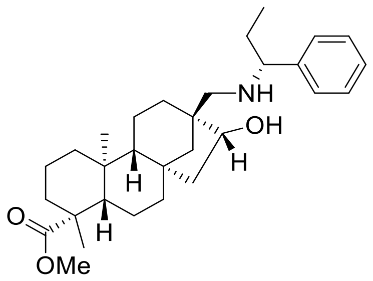

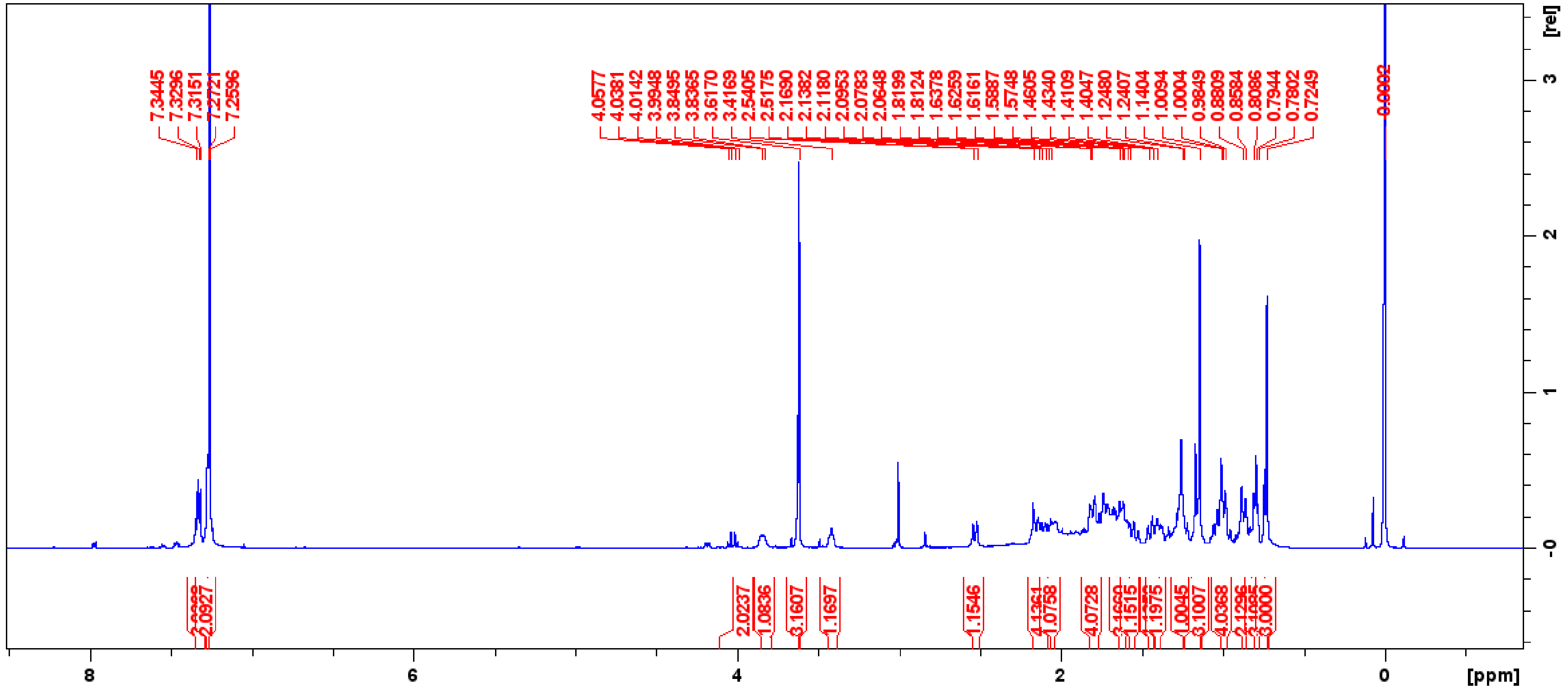

^{13}C-NMR of compound (4*R*,4a*S*,6a*R*,8*R*,9*S*,11a*R*,11b*S*)-methyl 8-hydroxy-4,11b-dimethyl-9-((((*R*)-1-phenylpropyl)amino)methyl)tetradecahydro-6a,9-methanocyclohepta[a]naphthalene-4-carboxylate **(24)**

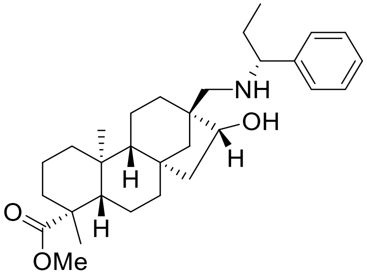

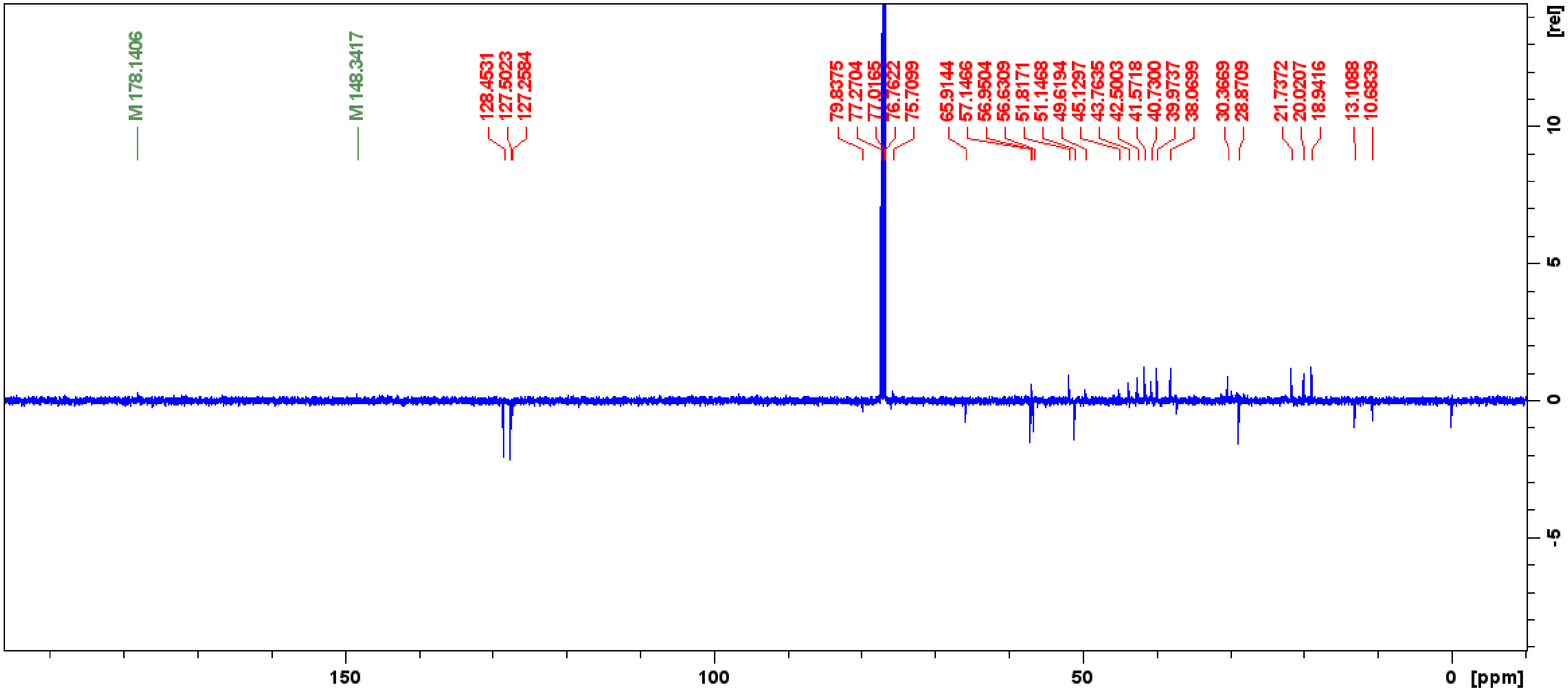

COSY of compound (4*R*,4a*S*,6a*R*,8*R*,9*S*,11a*R*,11b*S*)-methyl 8-hydroxy-4,11b-dimethyl-9-((((*R*)-1-phenylpropyl)amino)methyl)tetradecahydro-6a,9-methanocyclohepta[a]naphthalene-4-carboxylate **(24)**

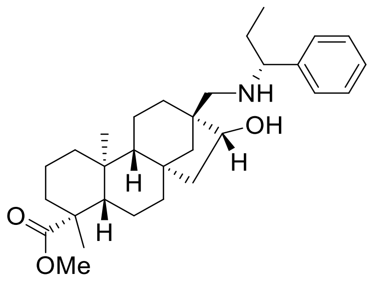

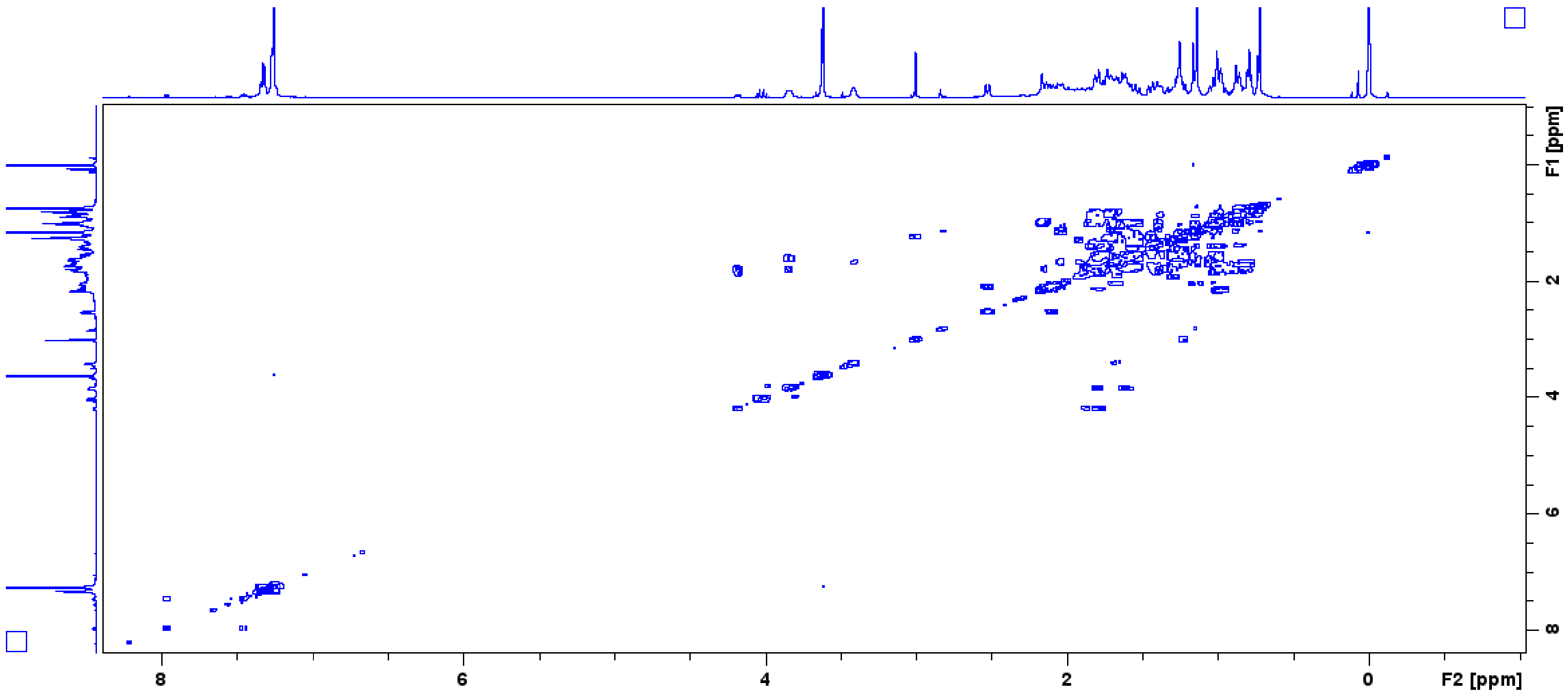

NOESY of compound (4*R*,4a*S*,6a*R*,8*R*,9*S*,11a*R*,11b*S*)-methyl 8-hydroxy-4,11b-dimethyl-9-((((*R*)-1-phenylpropyl)amino)methyl)tetradecahydro-6a,9-methanocyclohepta[a]naphthalene-4-carboxylate **(24)**

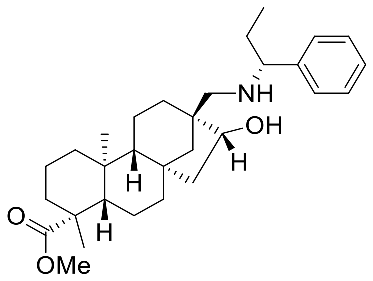

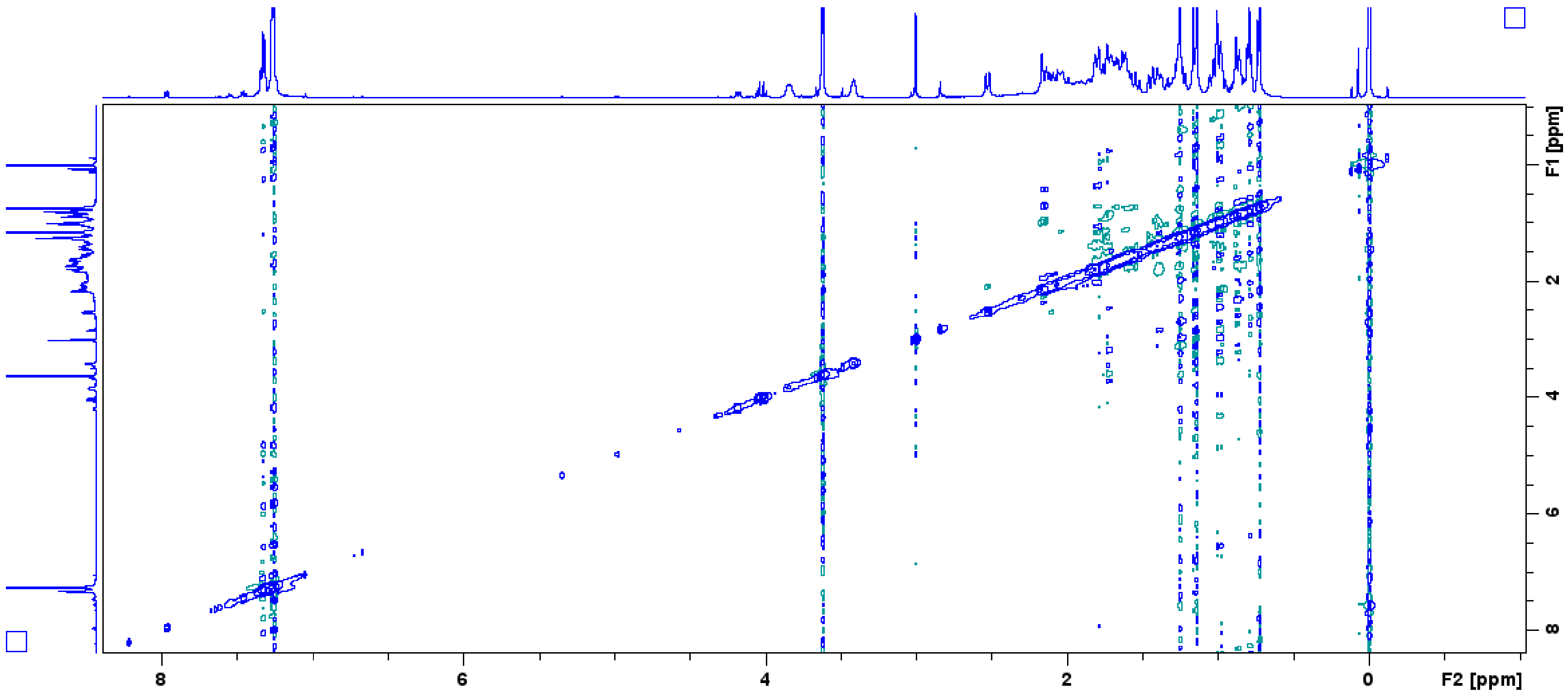

HSQC of compound (4*R*,4a*S*,6a*R*,8*R*,9*S*,11a*R*,11b*S*)-methyl 8-hydroxy-4,11b-dimethyl-9-((((*R*)-1-phenylpropyl)amino)methyl)tetradecahydro-6a,9-methanocyclohepta[a]naphthalene-4-carboxylate **(24)**

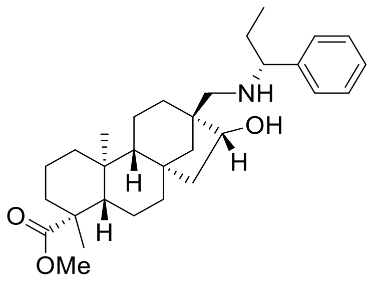

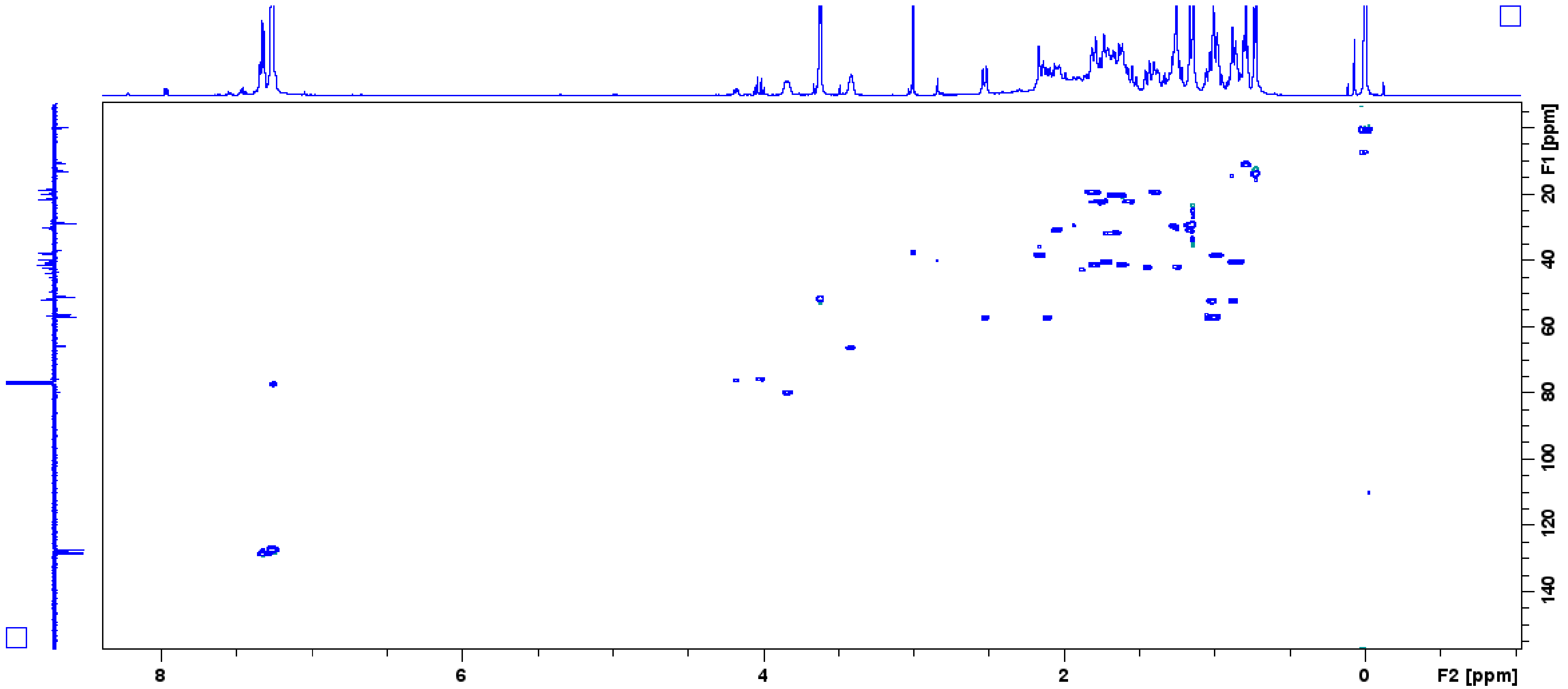

HMBC of compound (4*R*,4a*S*,6a*R*,8*R*,9*S*,11a*R*,11b*S*)-methyl 8-hydroxy-4,11b-dimethyl-9-((((*R*)-1-phenylpropyl)amino)methyl)tetradecahydro-6a,9-methanocyclohepta[a]naphthalene-4-carboxylate **(24)**

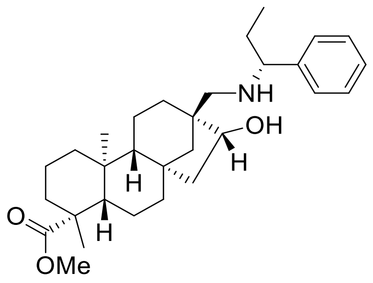

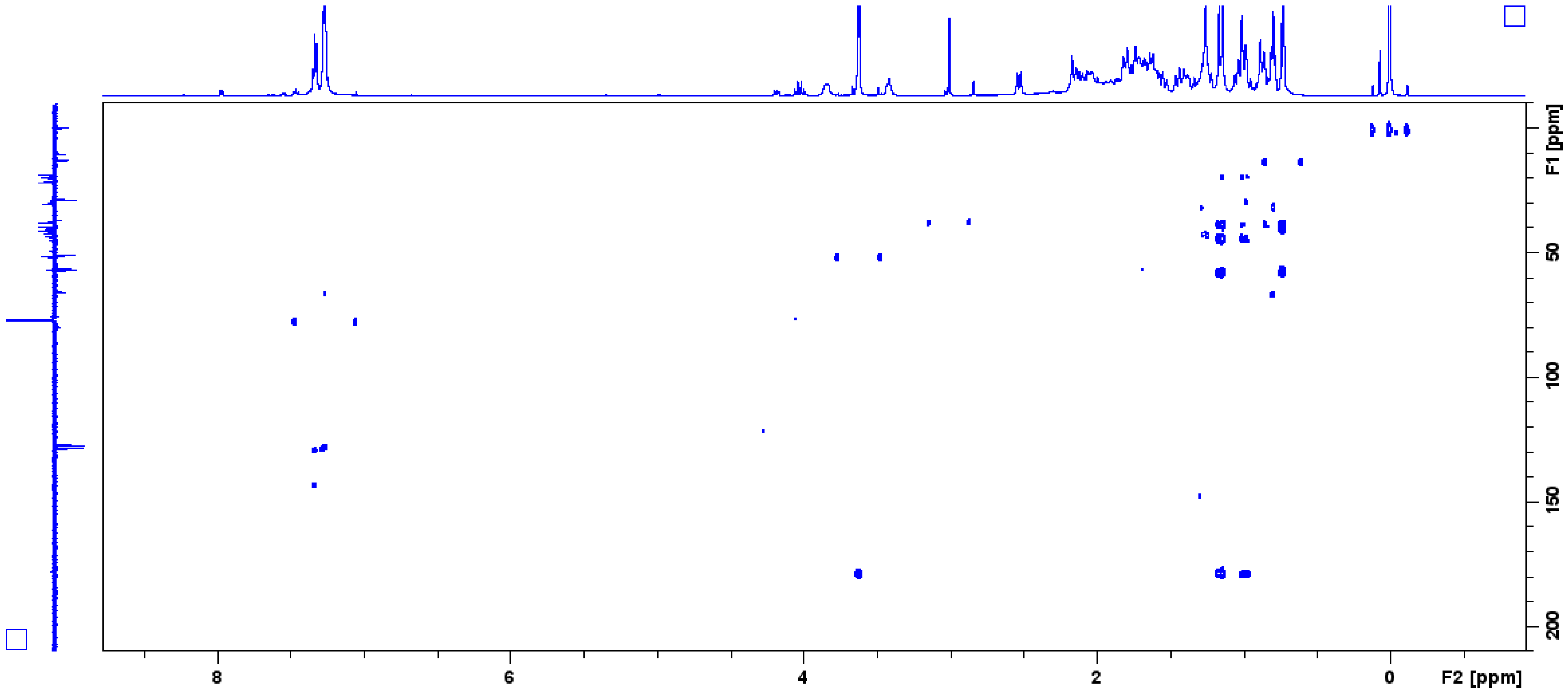

^{1}H-NMR of compound (4*R*,4a*S*,6a*R*,8*R*,9*S*,11a*R*,11b*S*)-methyl 8-hydroxy-4,11b-dimethyl-9-((((*S*)-1-phenylpropyl)amino)methyl)tetradecahydro-6a,9-methanocyclohepta[a]naphthalene-4-carboxylate **(25)**

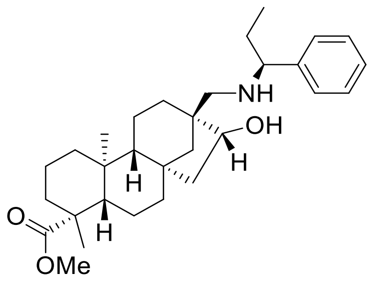

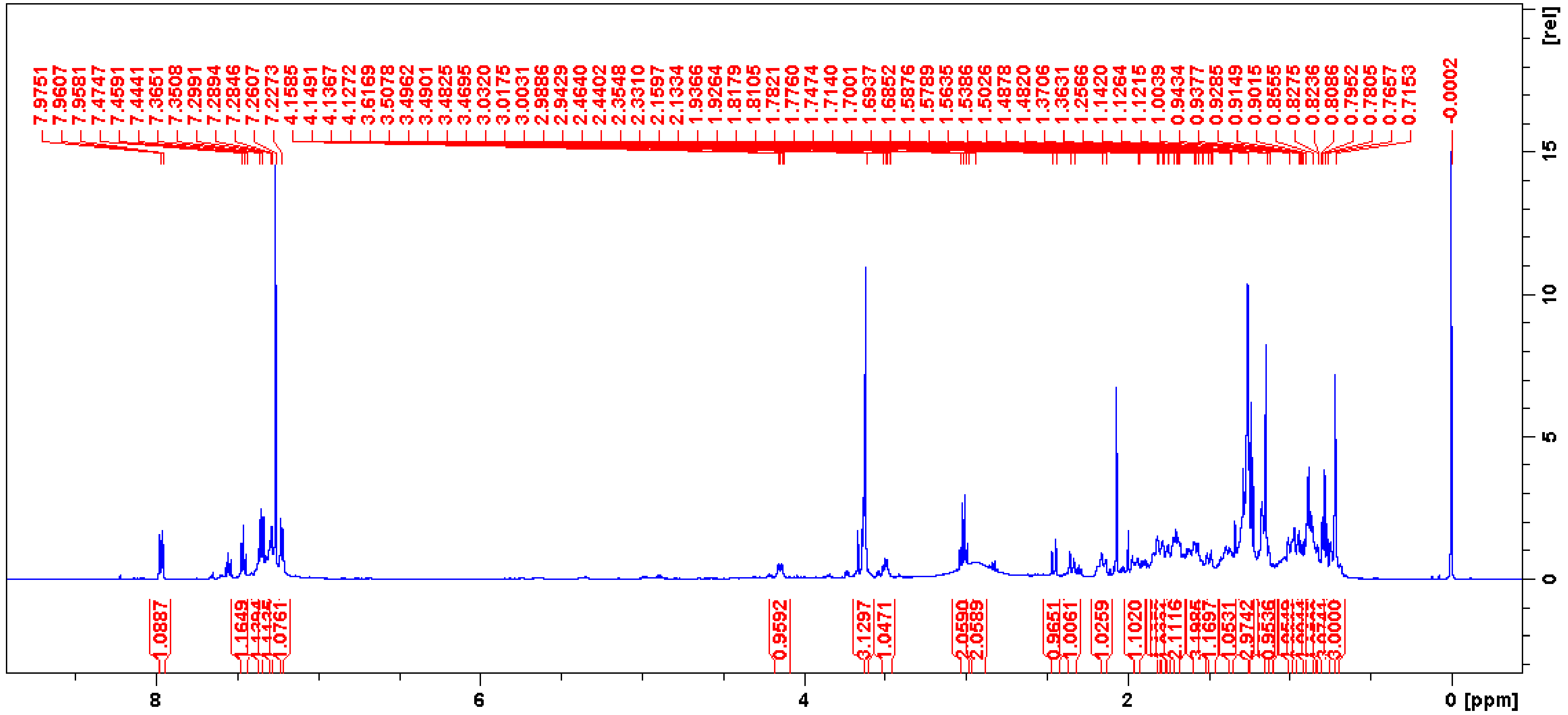

^{13}C-NMR of compound (4*R*,4a*S*,6a*R*,8*R*,9*S*,11a*R*,11b*S*)-methyl 8-hydroxy-4,11b-dimethyl-9-((((*S*)-1-phenylpropyl)amino)methyl)tetradecahydro-6a,9-methanocyclohepta[a]naphthalene-4-carboxylate **(25)**

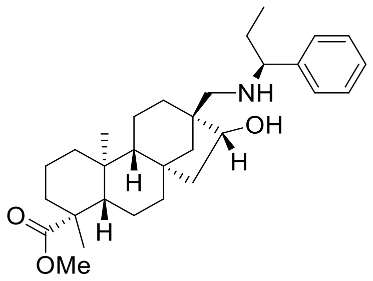

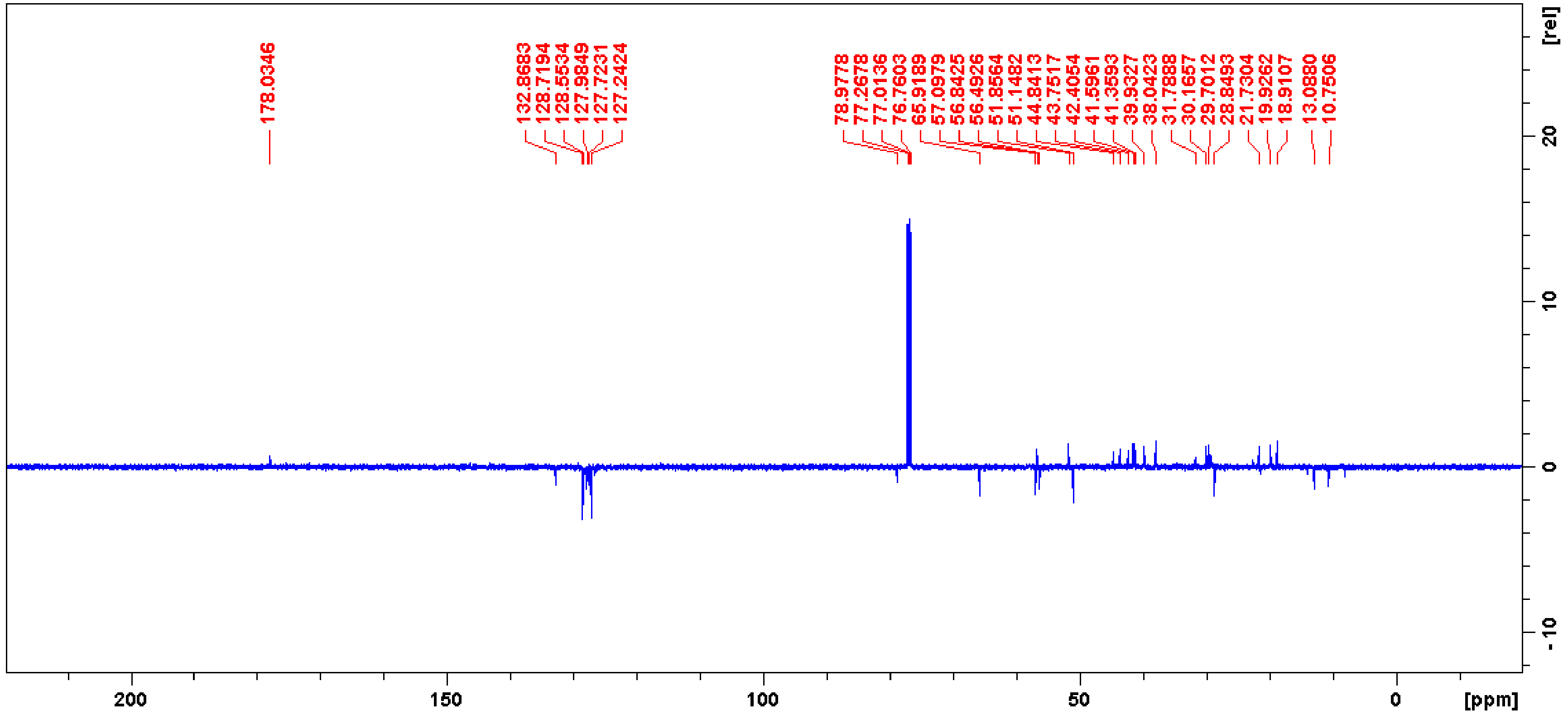

COSY of compound (4*R*,4a*S*,6a*R*,8*R*,9*S*,11a*R*,11b*S*)-methyl 8-hydroxy-4,11b-dimethyl-9-((((*S*)-1-phenylpropyl)amino)methyl)tetradecahydro-6a,9-methanocyclohepta[a]naphthalene-4-carboxylate **(25)**

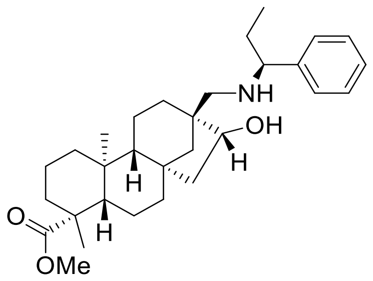

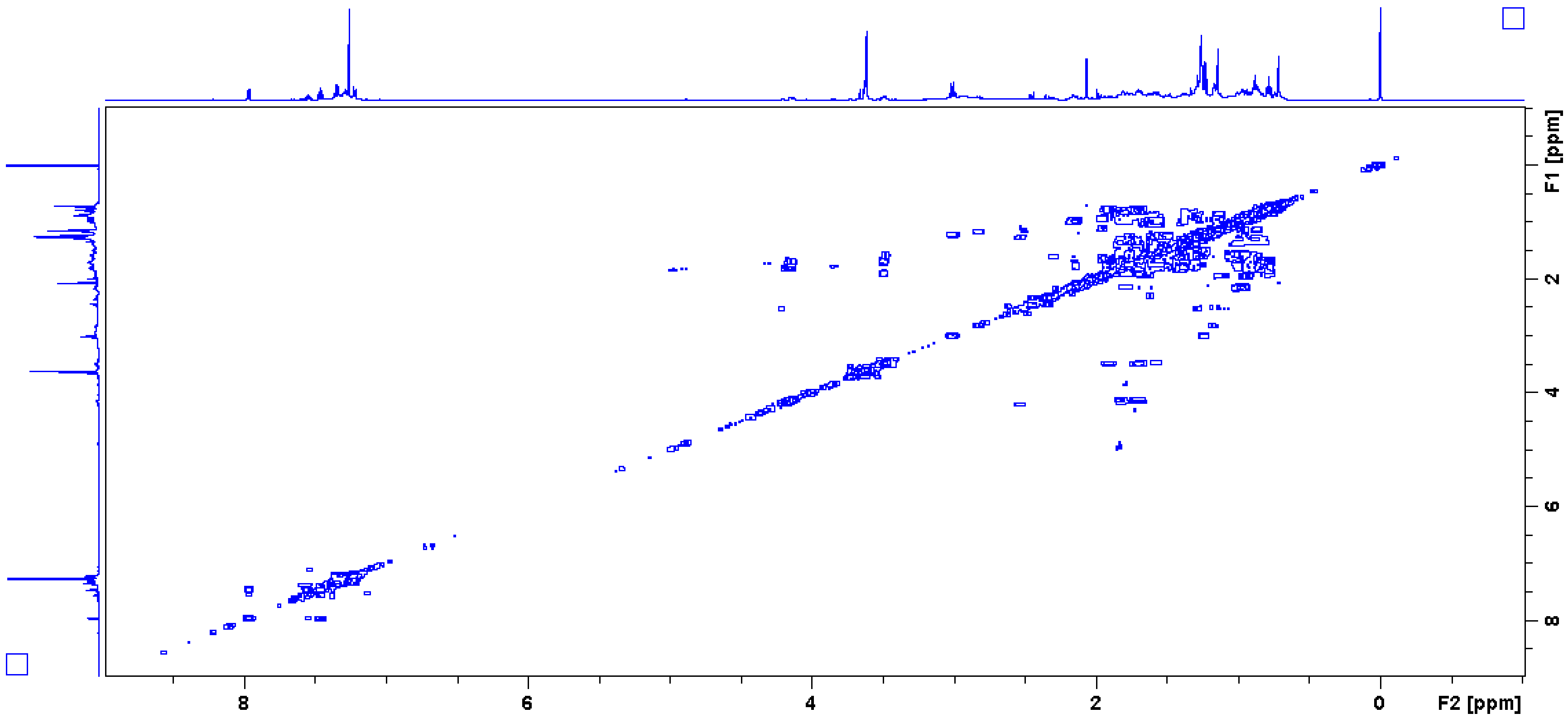

NOESY of compound (4*R*,4a*S*,6a*R*,8*R*,9*S*,11a*R*,11b*S*)-methyl 8-hydroxy-4,11b-dimethyl-9-((((*S*)-1-phenylpropyl)amino)methyl)tetradecahydro-6a,9-methanocyclohepta[a]naphthalene-4-carboxylate **(25)**

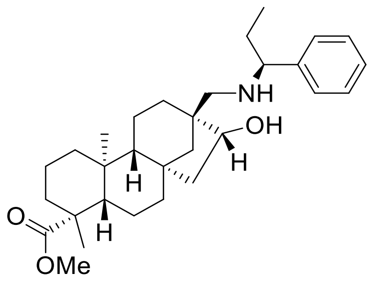

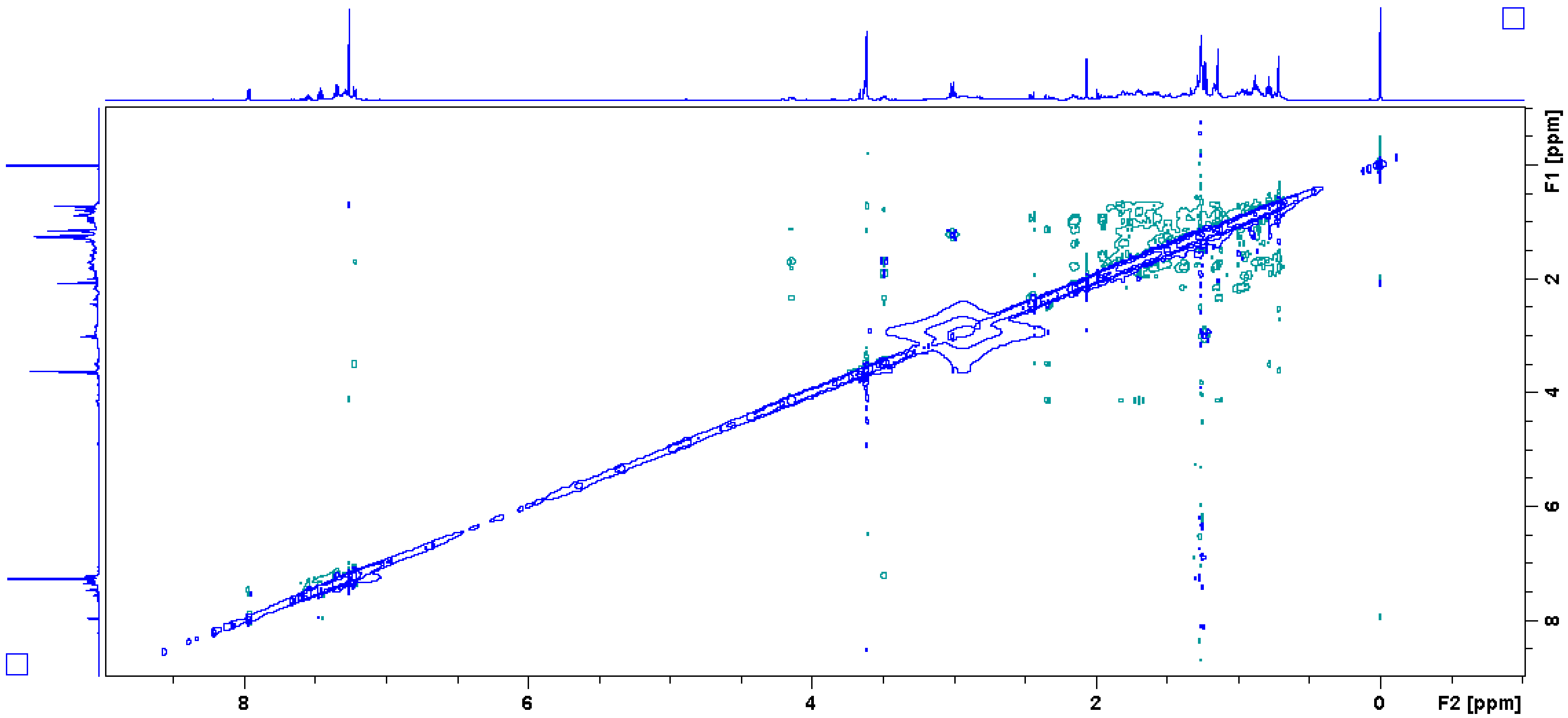

HSQC of compound (4*R*,4a*S*,6a*R*,8*R*,9*S*,11a*R*,11b*S*)-methyl 8-hydroxy-4,11b-dimethyl-9-((((*S*)-1-phenylpropyl)amino)methyl)tetradecahydro-6a,9-methanocyclohepta[a]naphthalene-4-carboxylate **(25)**

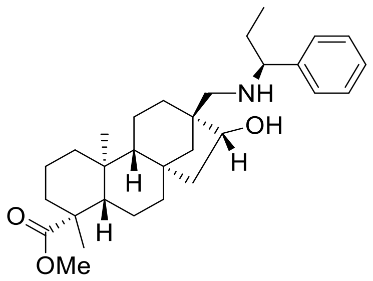

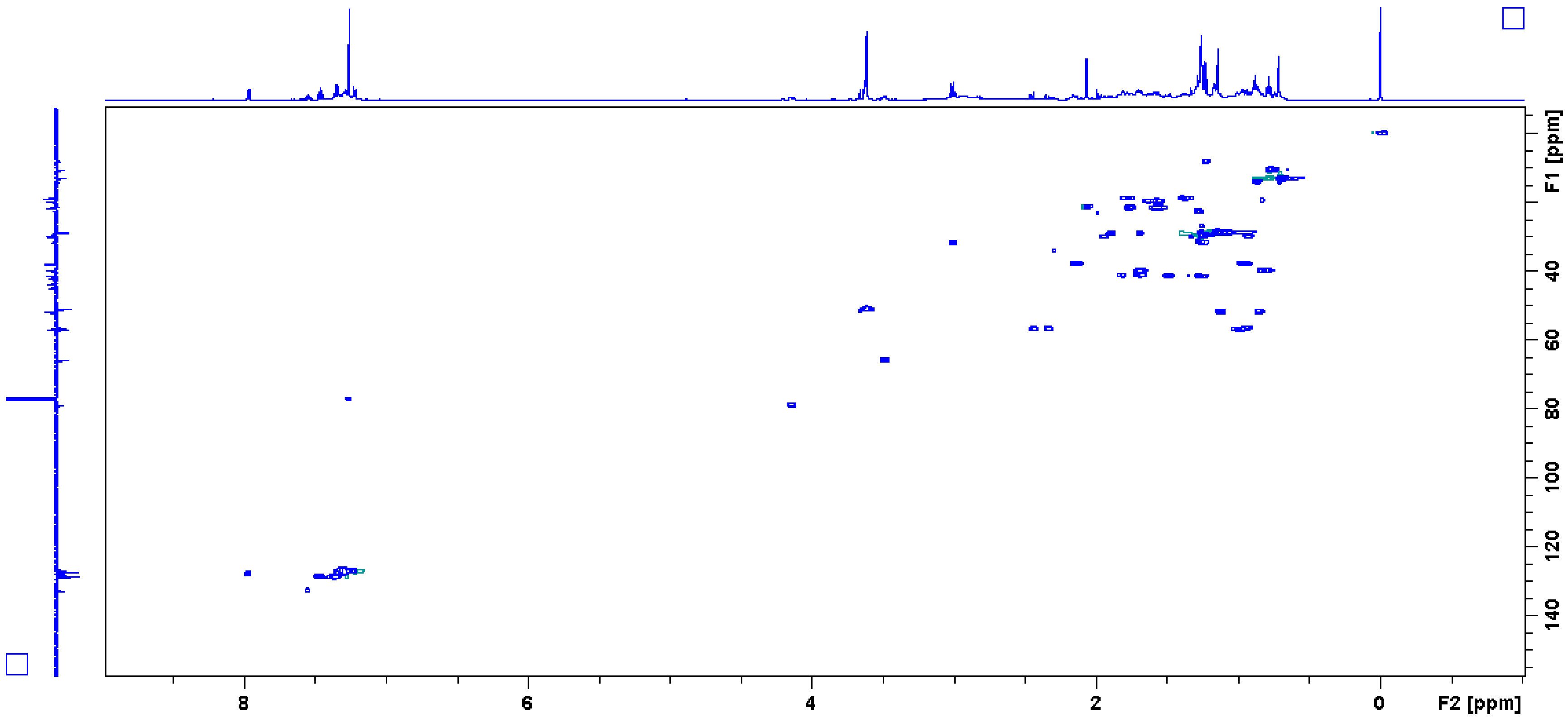

HMBC of compound (4*R*,4a*S*,6a*R*,8*R*,9*S*,11a*R*,11b*S*)-methyl 8-hydroxy-4,11b-dimethyl-9-((((*S*)-1-phenylpropyl)amino)methyl)tetradecahydro-6a,9-methanocyclohepta[a]naphthalene-4-carboxylate **(25)**

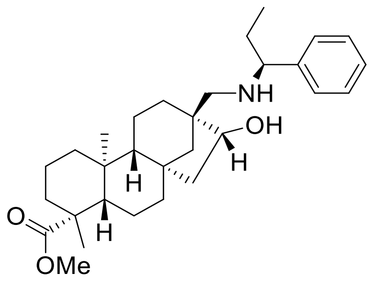

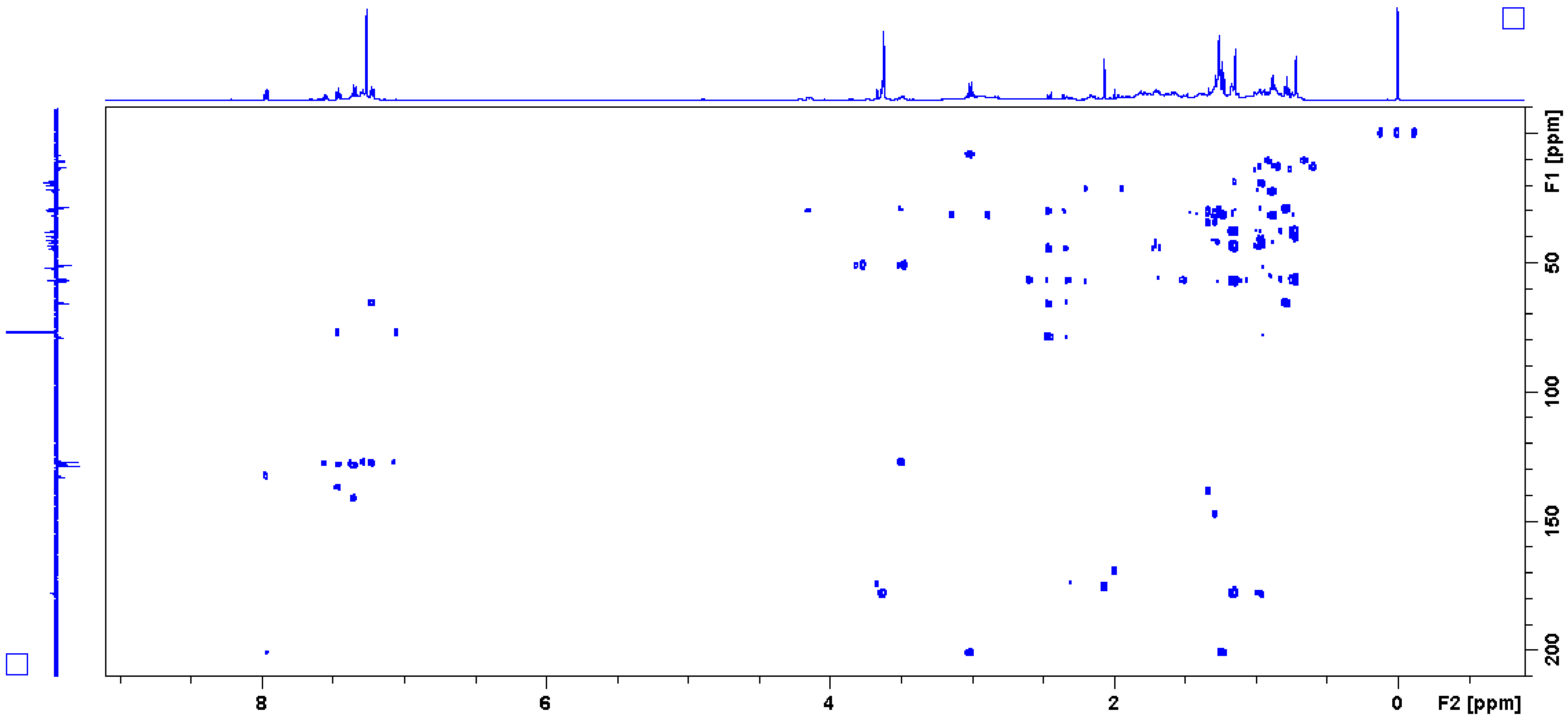

^{1}H-NMR of compound (4*R*,4a*S*,6a*R*,8*R*,9*S*,11a*R*,11b*S*)-methyl 8-hydroxy-4,11b-dimethyl-9-(((naphthalen-1-ylmethyl)amino)methyl)tetradecahydro-6a,9-methanocyclohepta[a]naphthalene-4-carboxylate **(26)**

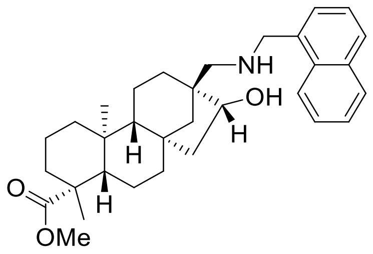

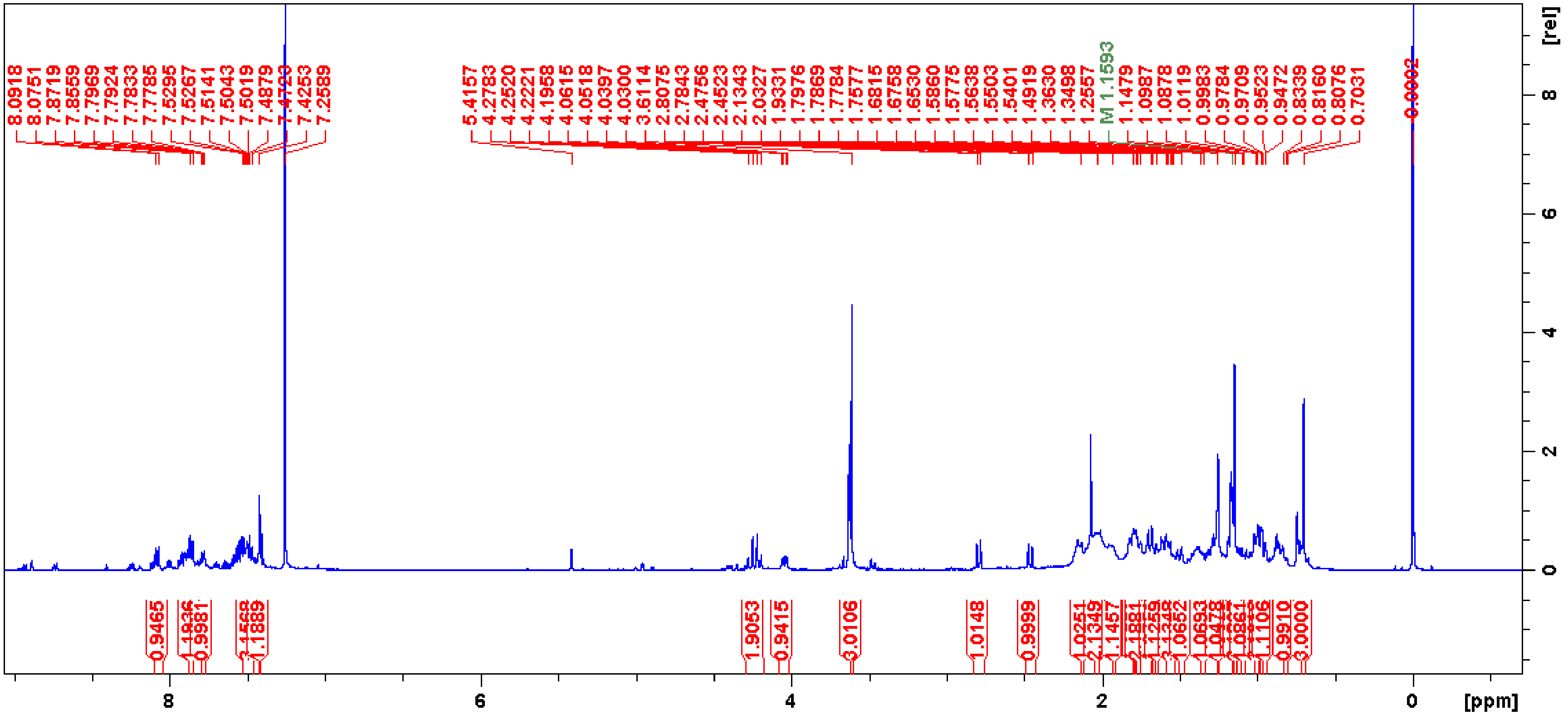

^{13}C-NMR of compound (4*R*,4a*S*,6a*R*,8*R*,9*S*,11a*R*,11b*S*)-methyl 8-hydroxy-4,11b-dimethyl-9-(((naphthalen-1-ylmethyl)amino)methyl)tetradecahydro-6a,9-methanocyclohepta[a]naphthalene-4-carboxylate **(26)**

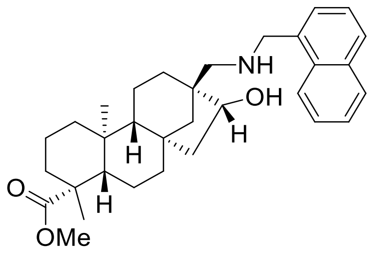

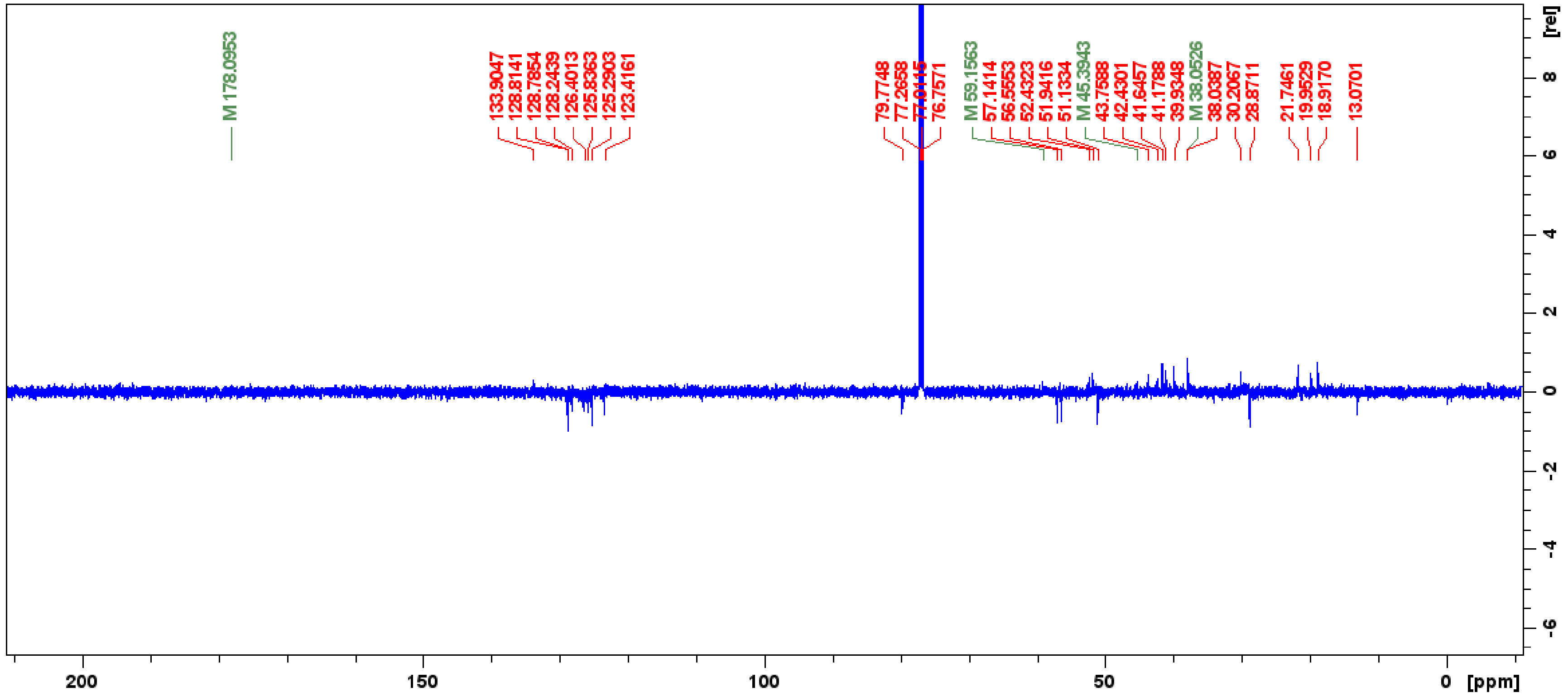

COSY of compound (4*R*,4a*S*,6a*R*,8*R*,9*S*,11a*R*,11b*S*)-methyl 8-hydroxy-4,11b-dimethyl-9-(((naphthalen-1-ylmethyl)amino)methyl)tetradecahydro-6a,9-methanocyclohepta[a]naphthalene-4-carboxylate **(26)**

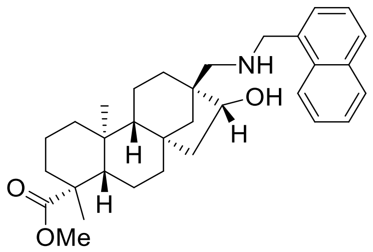

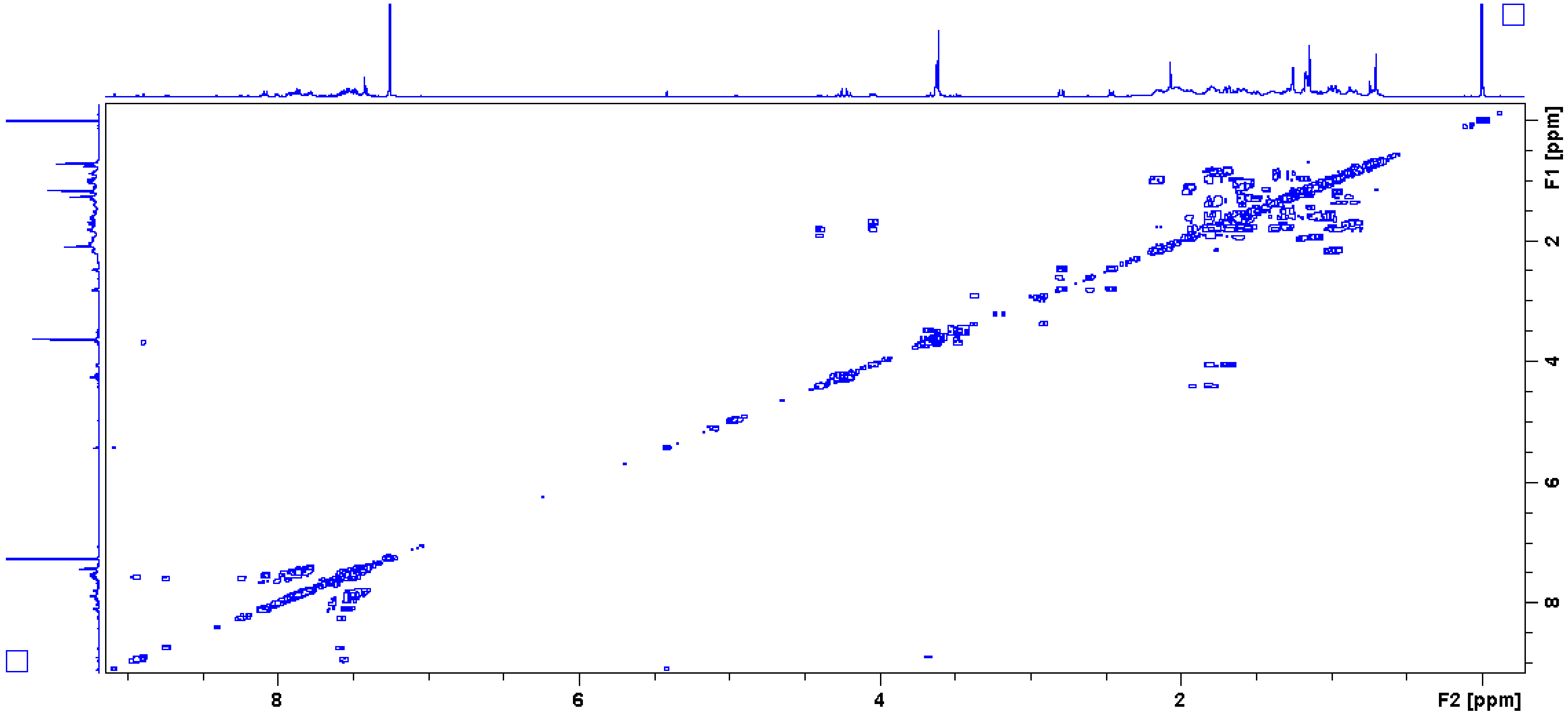

NOESY of compound (4*R*,4a*S*,6a*R*,8*R*,9*S*,11a*R*,11b*S*)-methyl 8-hydroxy-4,11b-dimethyl-9-(((naphthalen-1-ylmethyl)amino)methyl)tetradecahydro-6a,9-methanocyclohepta[a]naphthalene-4-carboxylate **(26)**

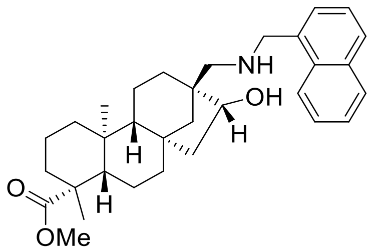

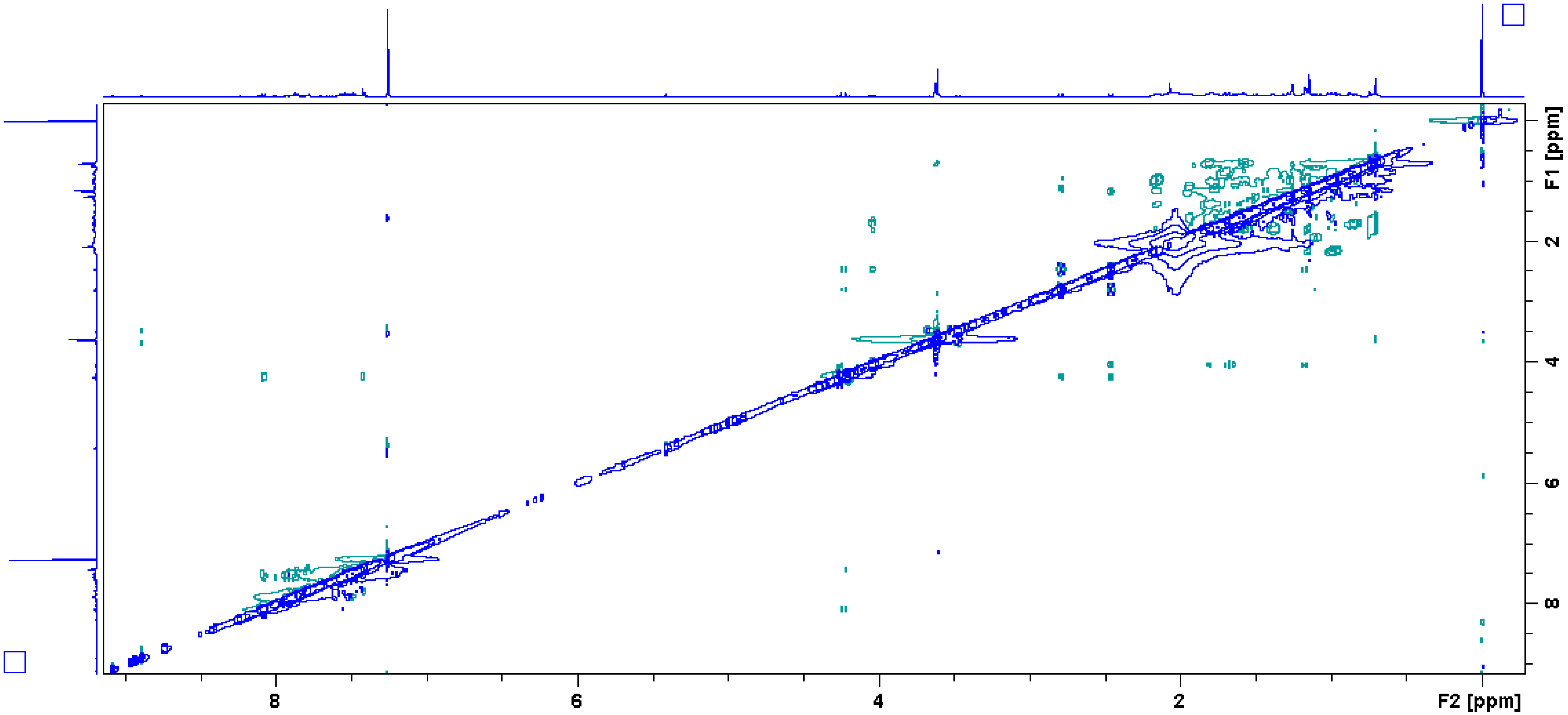

HSQC of compound (4*R*,4a*S*,6a*R*,8*R*,9*S*,11a*R*,11b*S*)-methyl 8-hydroxy-4,11b-dimethyl-9-(((naphthalen-1-ylmethyl)amino)methyl)tetradecahydro-6a,9-methanocyclohepta[a]naphthalene-4-carboxylate **(26)**

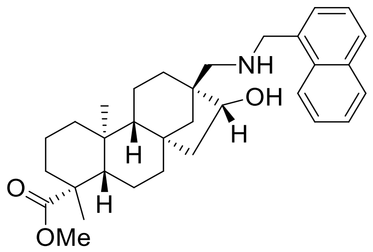

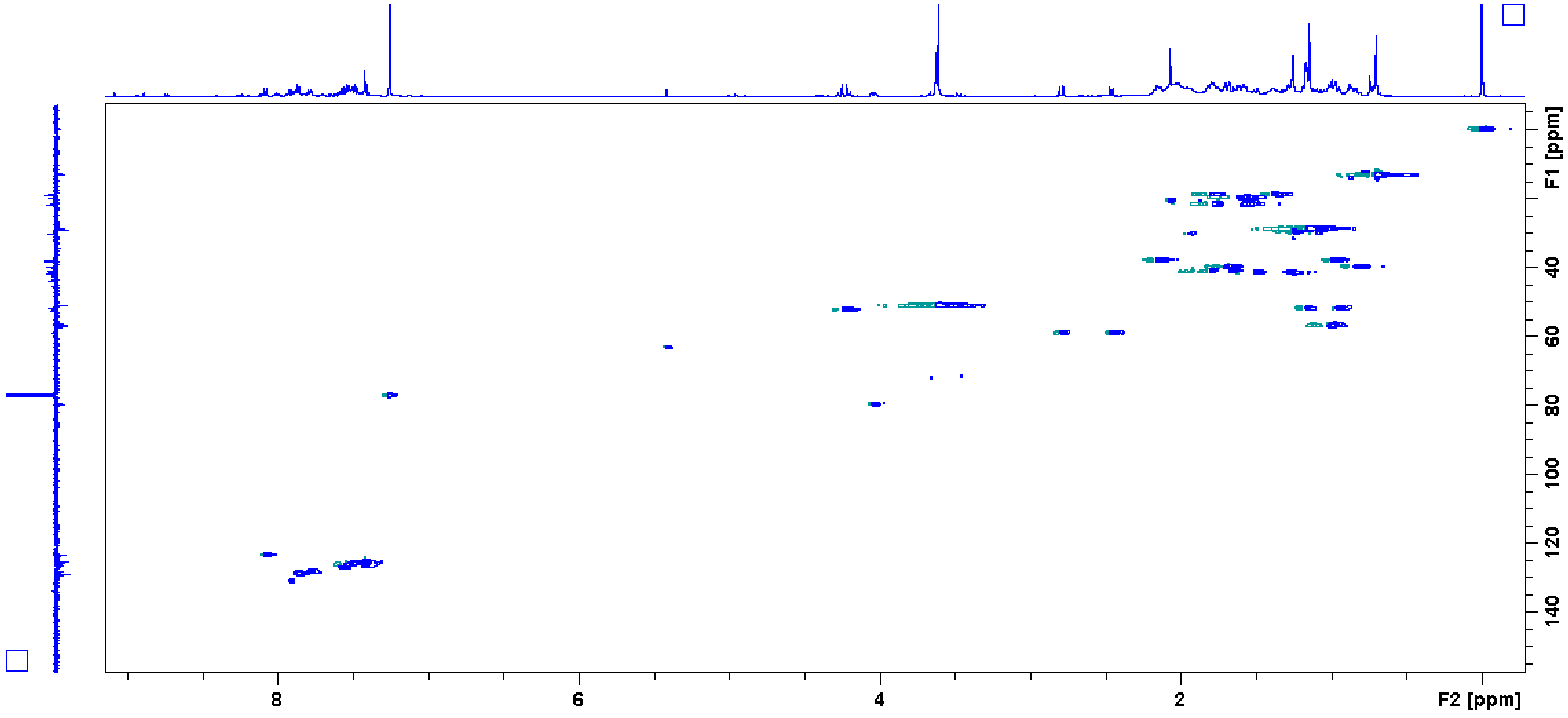

HMBC of compound (4*R*,4a*S*,6a*R*,8*R*,9*S*,11a*R*,11b*S*)-methyl 8-hydroxy-4,11b-dimethyl-9-(((naphthalen-1-ylmethyl)amino)methyl)tetradecahydro-6a,9-methanocyclohepta[a]naphthalene-4-carboxylate **(26)**

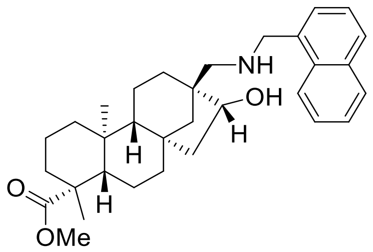

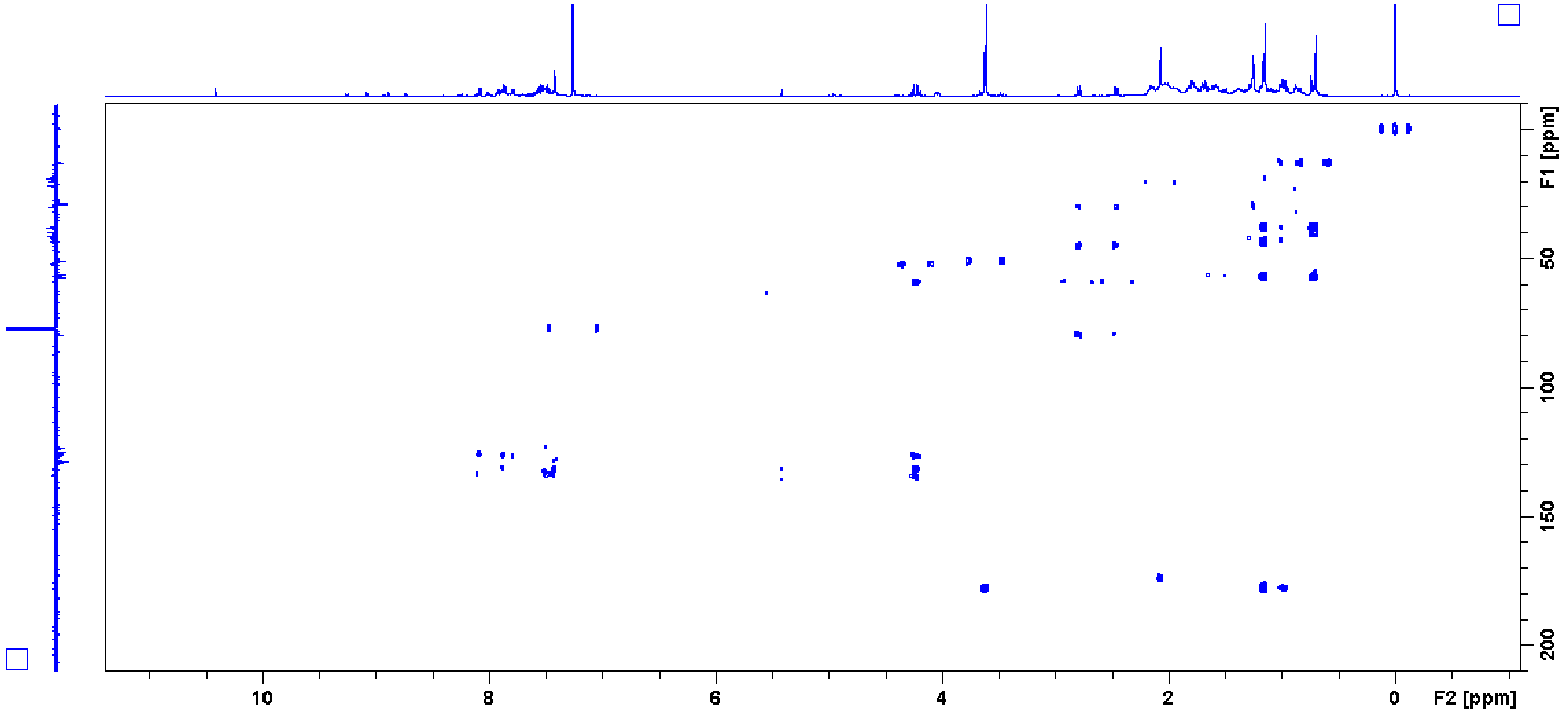

^{1}H-NMR of compound (4*R*,4a*S*,6a*R*,8*R*,9*S*,11a*R*,11b*S*)-methyl 8-hydroxy-4,11b-dimethyl-9-((((*S*)-1-(naphthalen-1-yl)ethyl)amino)methyl)tetradecahydro-6a,9-methanocyclohepta[a]naphthalene-4-carboxylate **(27)**

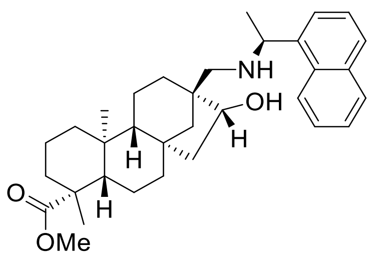

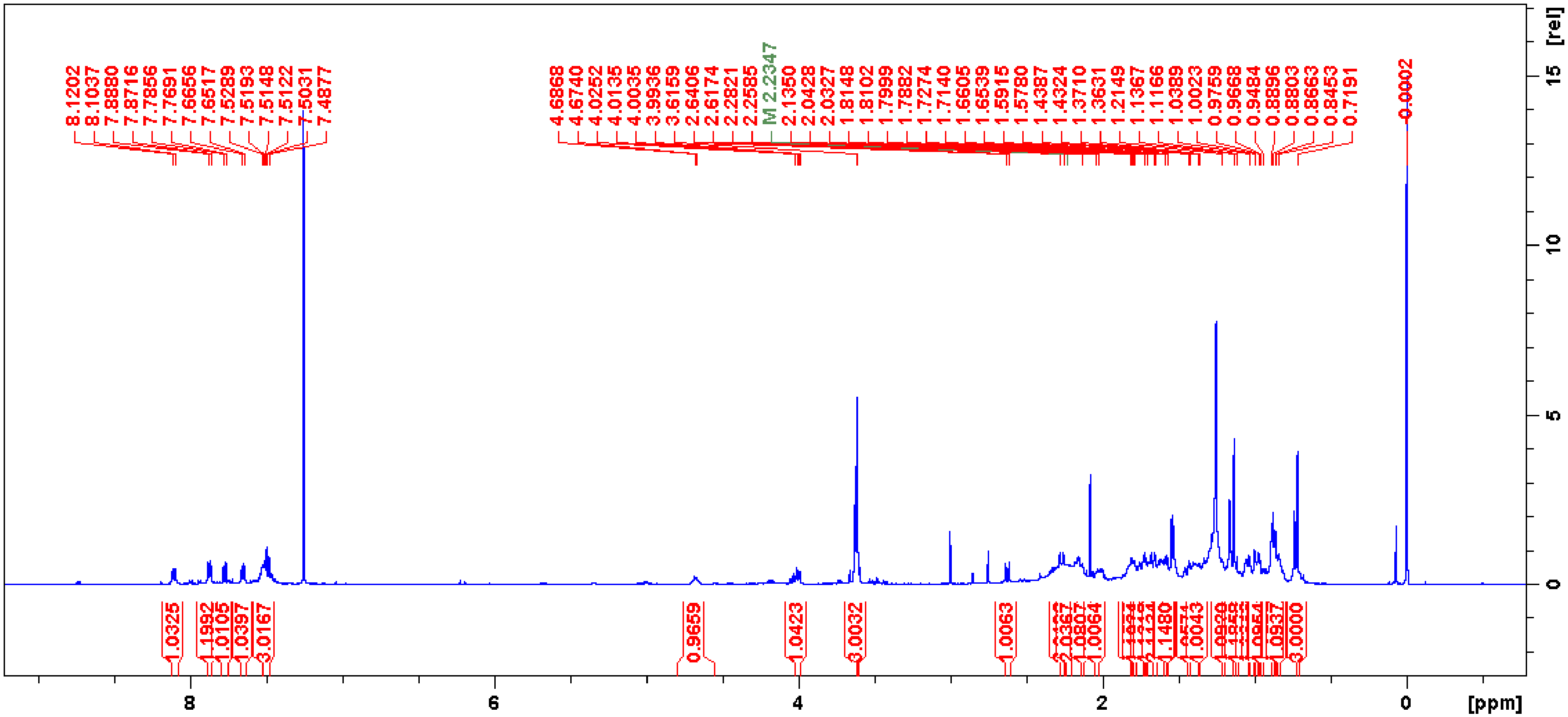

^{13}C-NMR of compound (4*R*,4a*S*,6a*R*,8*R*,9*S*,11a*R*,11b*S*)-methyl 8-hydroxy-4,11b-dimethyl-9-((((*S*)-1-(naphthalen-1-yl)ethyl)amino)methyl)tetradecahydro-6a,9-methanocyclohepta[a]naphthalene-4-carboxylate **(27)**

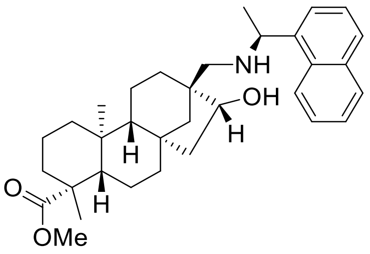

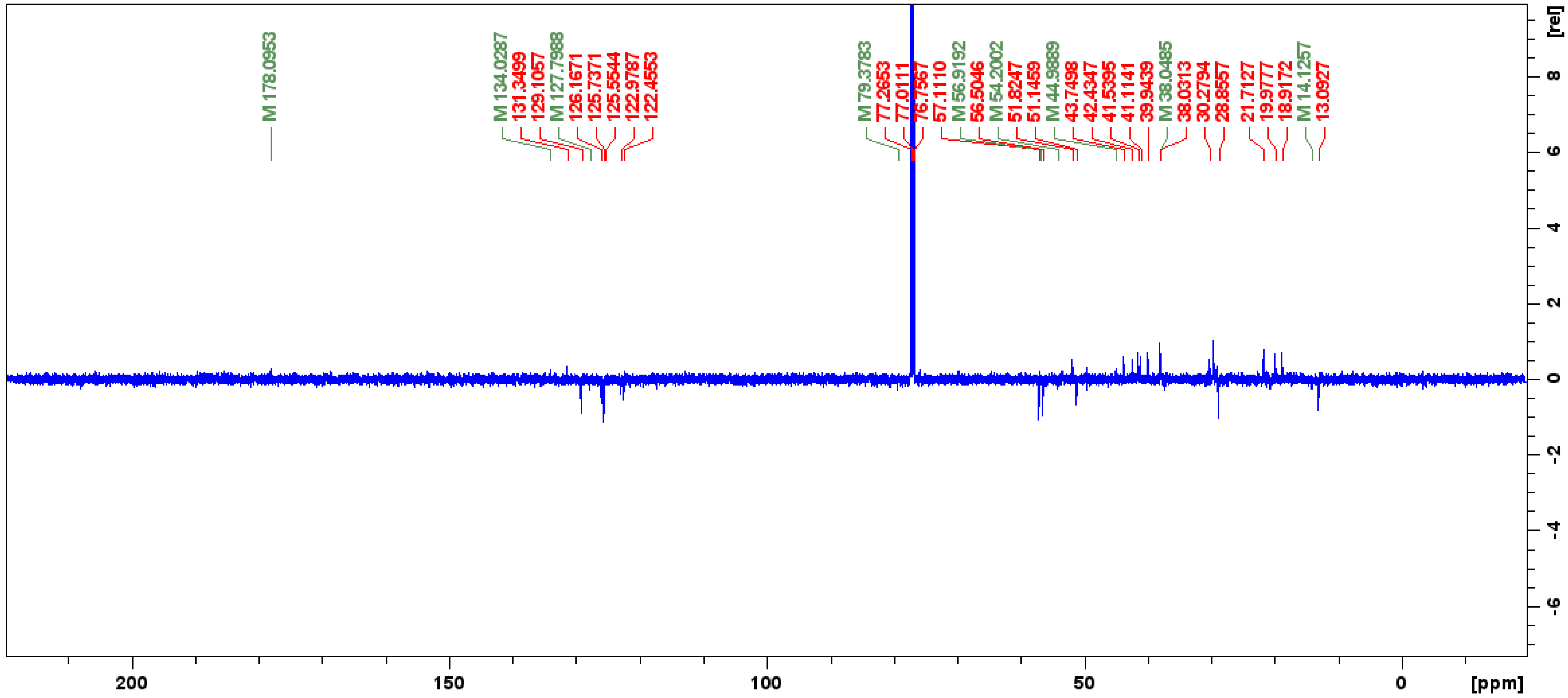

COSY of compound (4*R*,4a*S*,6a*R*,8*R*,9*S*,11a*R*,11b*S*)-methyl 8-hydroxy-4,11b-dimethyl-9-((((*S*)-1-(naphthalen-1-yl)ethyl)amino)methyl)tetradecahydro-6a,9-methanocyclohepta[a]naphthalene-4-carboxylate **(27)**

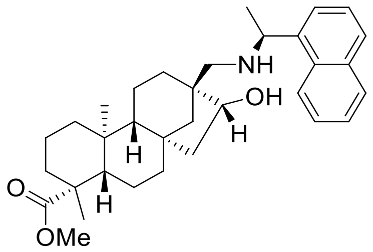

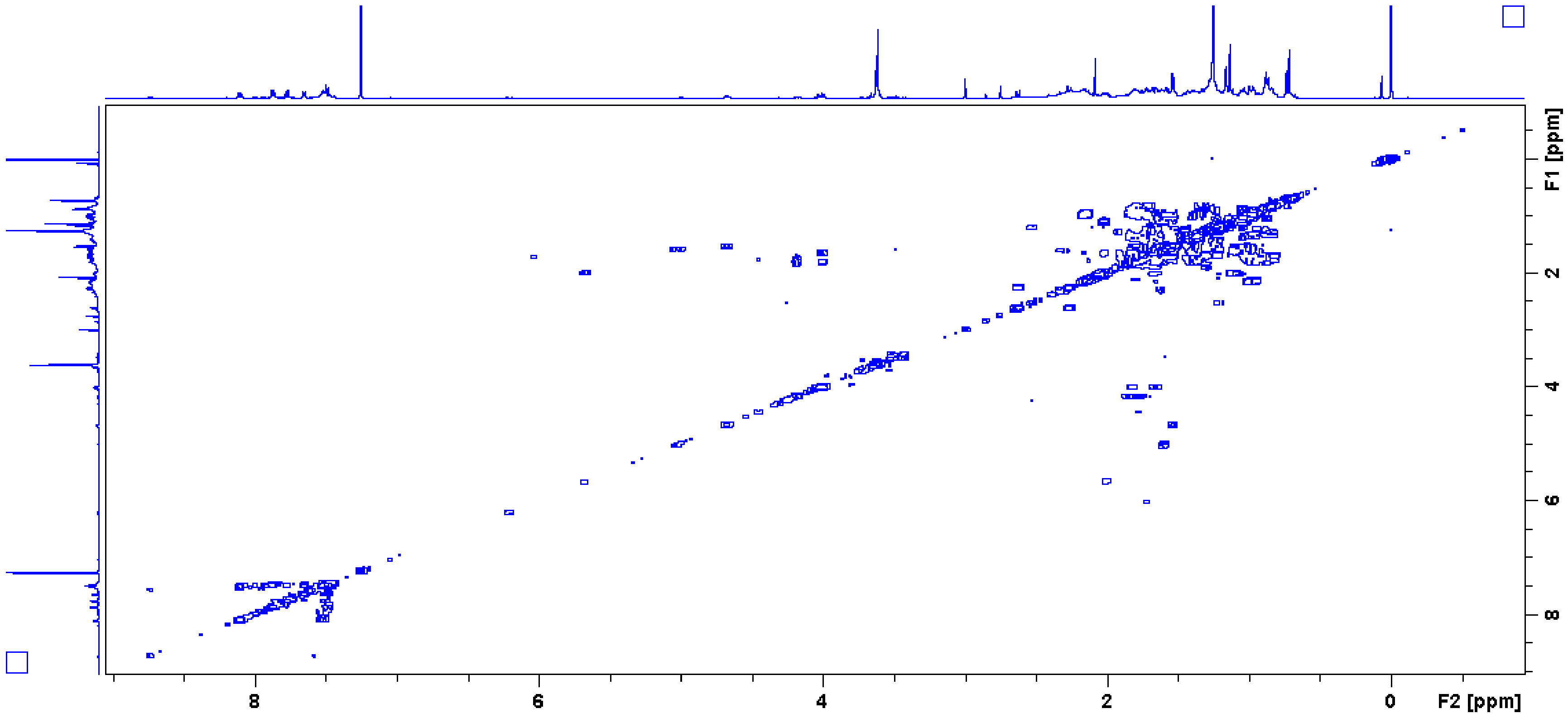

NOESY of compound (4*R*,4a*S*,6a*R*,8*R*,9*S*,11a*R*,11b*S*)-methyl 8-hydroxy-4,11b-dimethyl-9-((((*S*)-1-(naphthalen-1-yl)ethyl)amino)methyl)tetradecahydro-6a,9-methanocyclohepta[a]naphthalene-4-carboxylate **(27)**

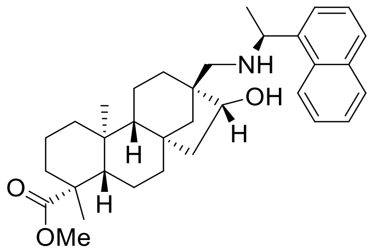

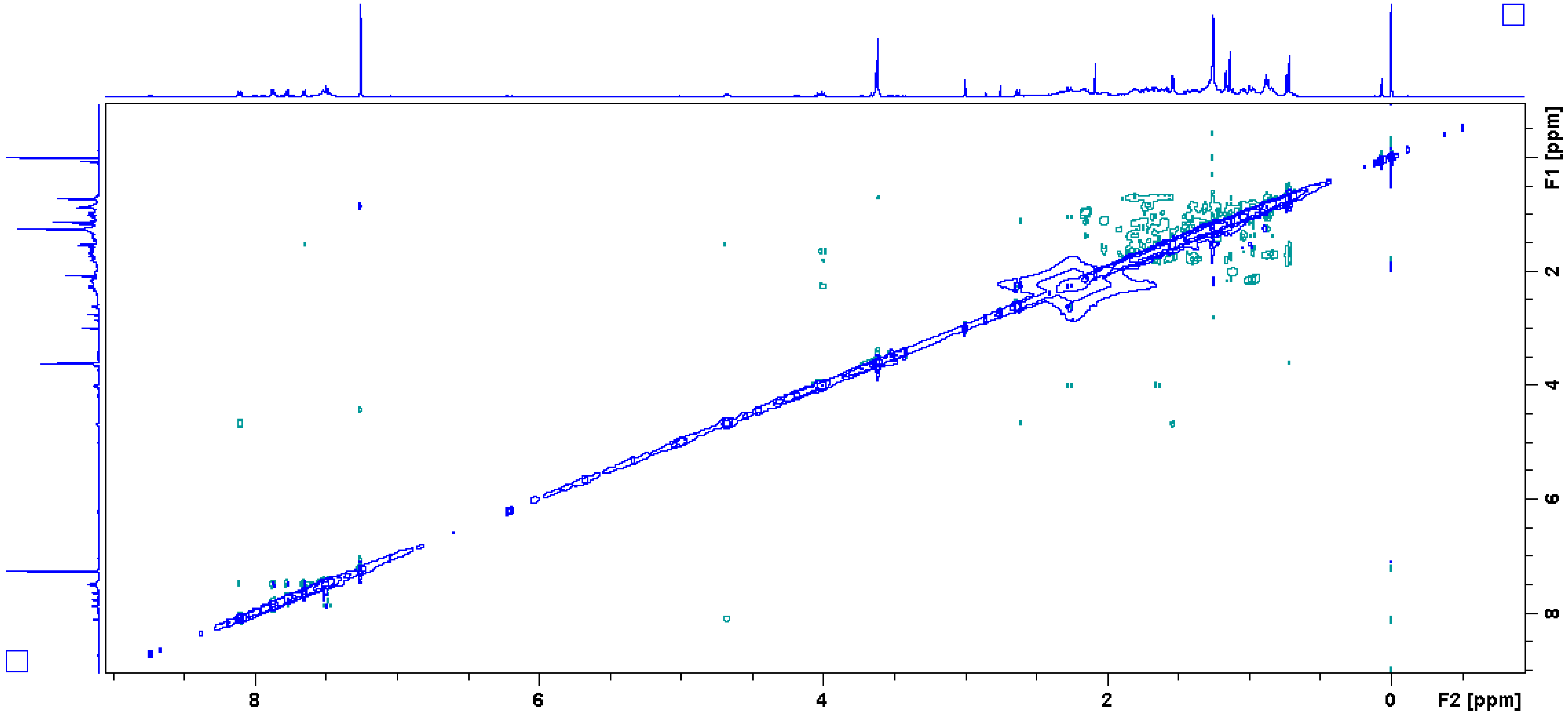

HSQC of compound (4*R*,4a*S*,6a*R*,8*R*,9*S*,11a*R*,11b*S*)-methyl 8-hydroxy-4,11b-dimethyl-9-((((*S*)-1-(naphthalen-1-yl)ethyl)amino)methyl)tetradecahydro-6a,9-methanocyclohepta[a]naphthalene-4-carboxylate **(27)**

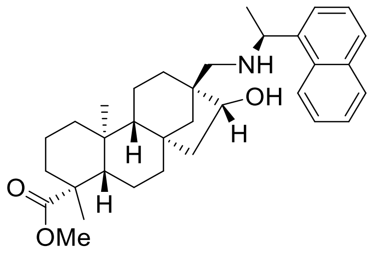

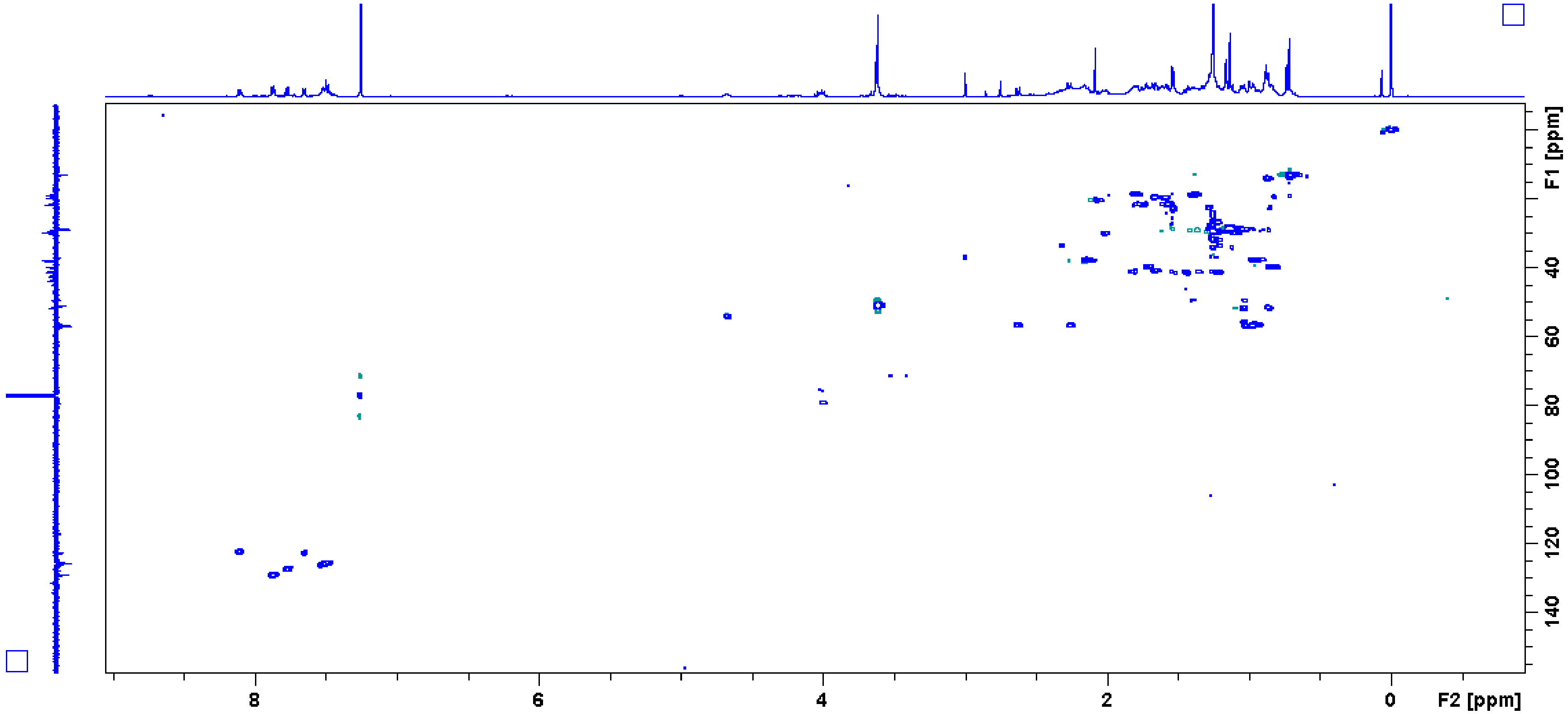

HMBC of compound (4*R*,4a*S*,6a*R*,8*R*,9*S*,11a*R*,11b*S*)-methyl 8-hydroxy-4,11b-dimethyl-9-((((*S*)-1-(naphthalen-1-yl)ethyl)amino)methyl)tetradecahydro-6a,9-methanocyclohepta[a]naphthalene-4-carboxylate **(27)**

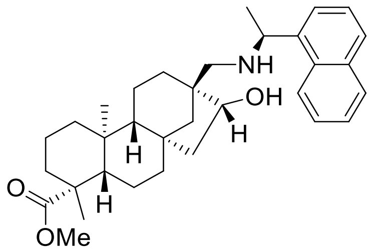

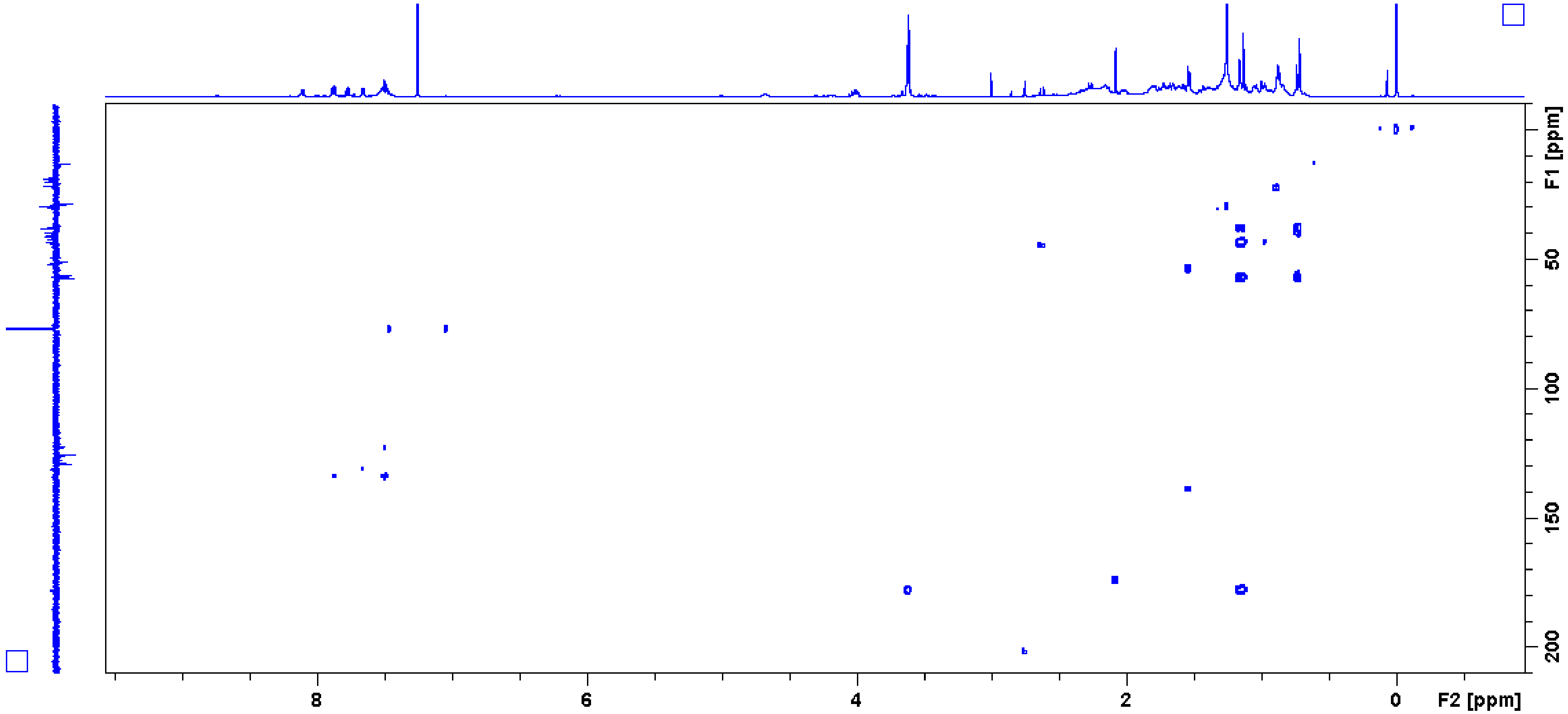

^{1}H-NMR of compound (4*R*,4a*S*,6a*R*,8*R*,9*S*,11a*R*,11b*S*)-methyl 8-hydroxy-4,11b-dimethyl-9-((((*R*)-1-(naphthalen-1-yl)ethyl)amino)methyl)tetradecahydro-6a,9-methanocyclohepta[a]naphthalene-4-carboxylate **(28)**

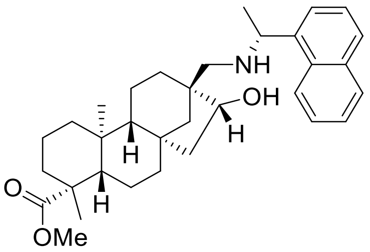

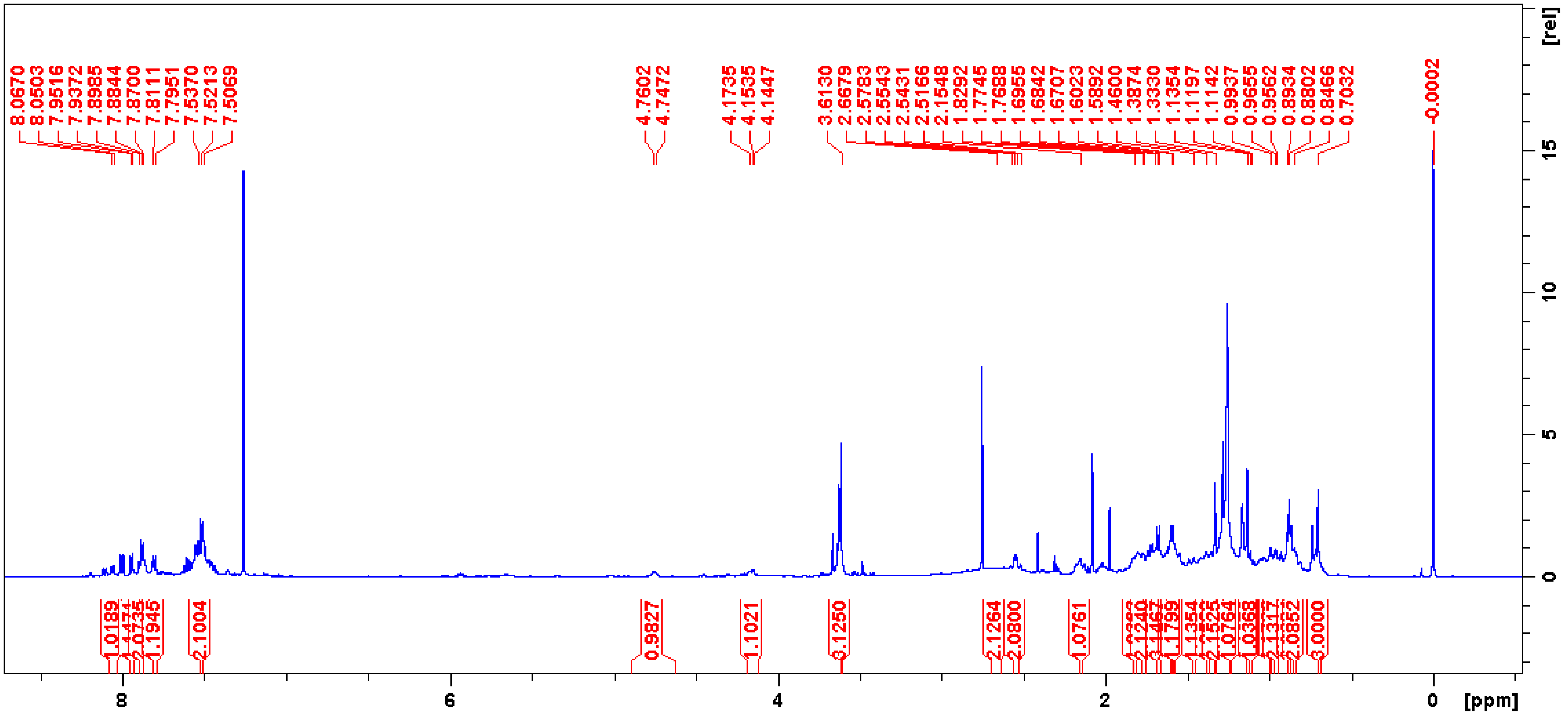

^{13}C-NMR of compound (4*R*,4a*S*,6a*R*,8*R*,9*S*,11a*R*,11b*S*)-methyl 8-hydroxy-4,11b-dimethyl-9-((((*R*)-1-(naphthalen-1-yl)ethyl)amino)methyl)tetradecahydro-6a,9-methanocyclohepta[a]naphthalene-4-carboxylate **(28)**

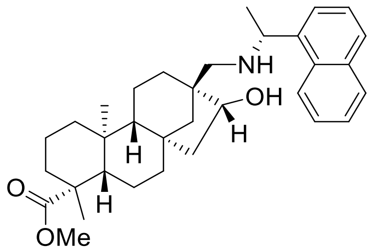

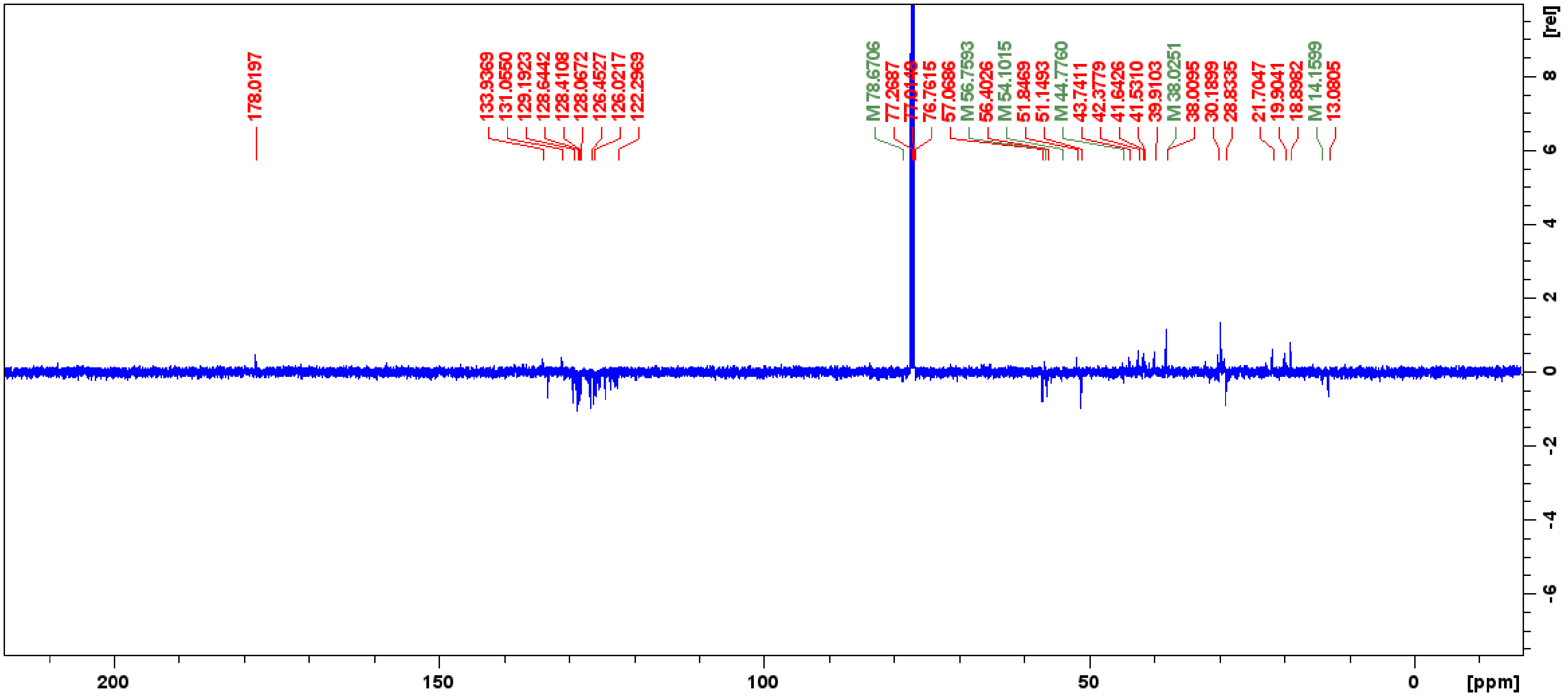

COSY of compound (4*R*,4a*S*,6a*R*,8*R*,9*S*,11a*R*,11b*S*)-methyl 8-hydroxy-4,11b-dimethyl-9-((((*R*)-1-(naphthalen-1-yl)ethyl)amino)methyl)tetradecahydro-6a,9-methanocyclohepta[a]naphthalene-4-carboxylate **(28)**

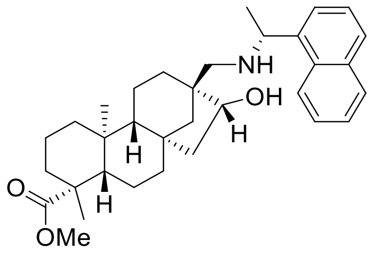

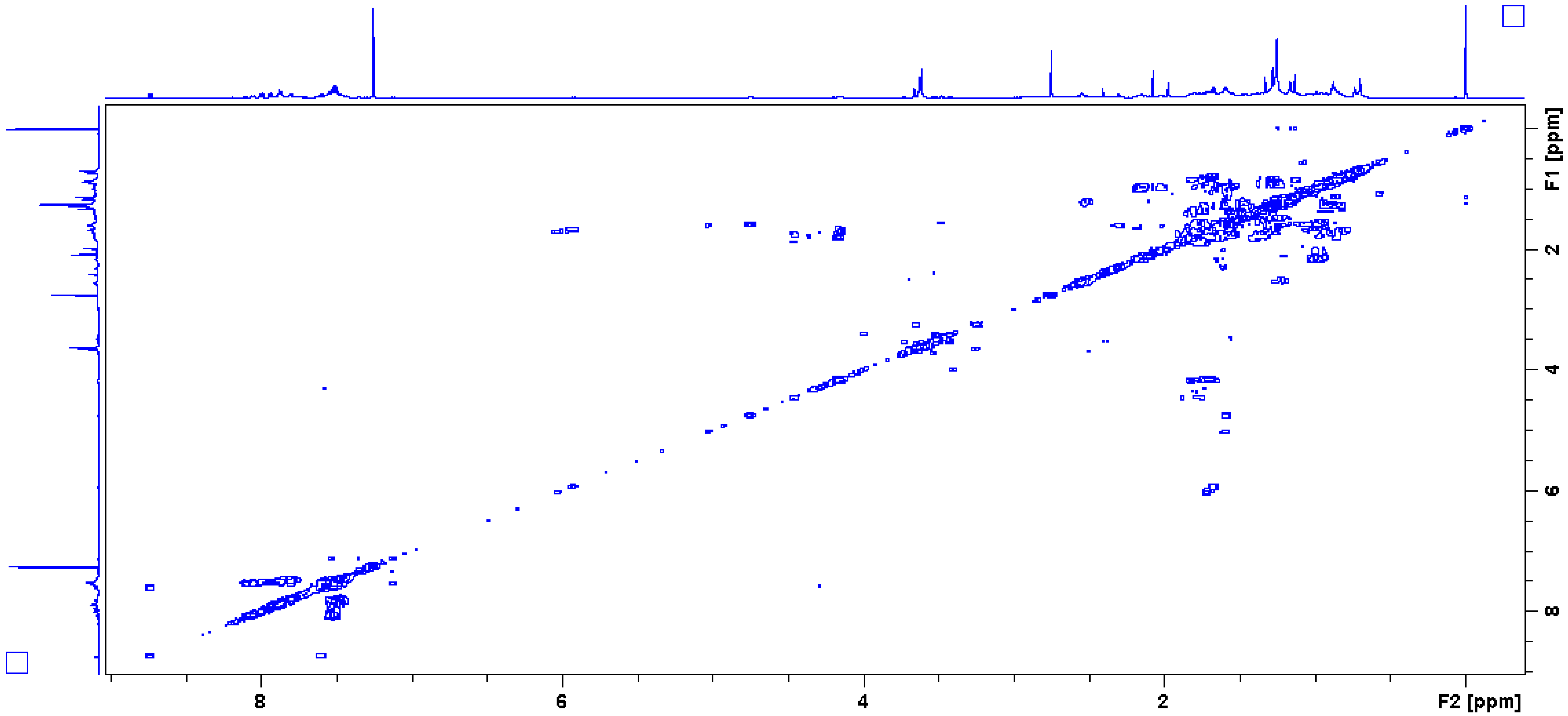

NOESY of compound (4*R*,4a*S*,6a*R*,8*R*,9*S*,11a*R*,11b*S*)-methyl 8-hydroxy-4,11b-dimethyl-9-((((*R*)-1-(naphthalen-1-yl)ethyl)amino)methyl)tetradecahydro-6a,9-methanocyclohepta[a]naphthalene-4-carboxylate **(28)**

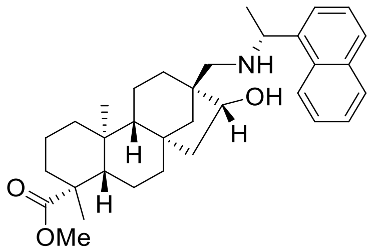

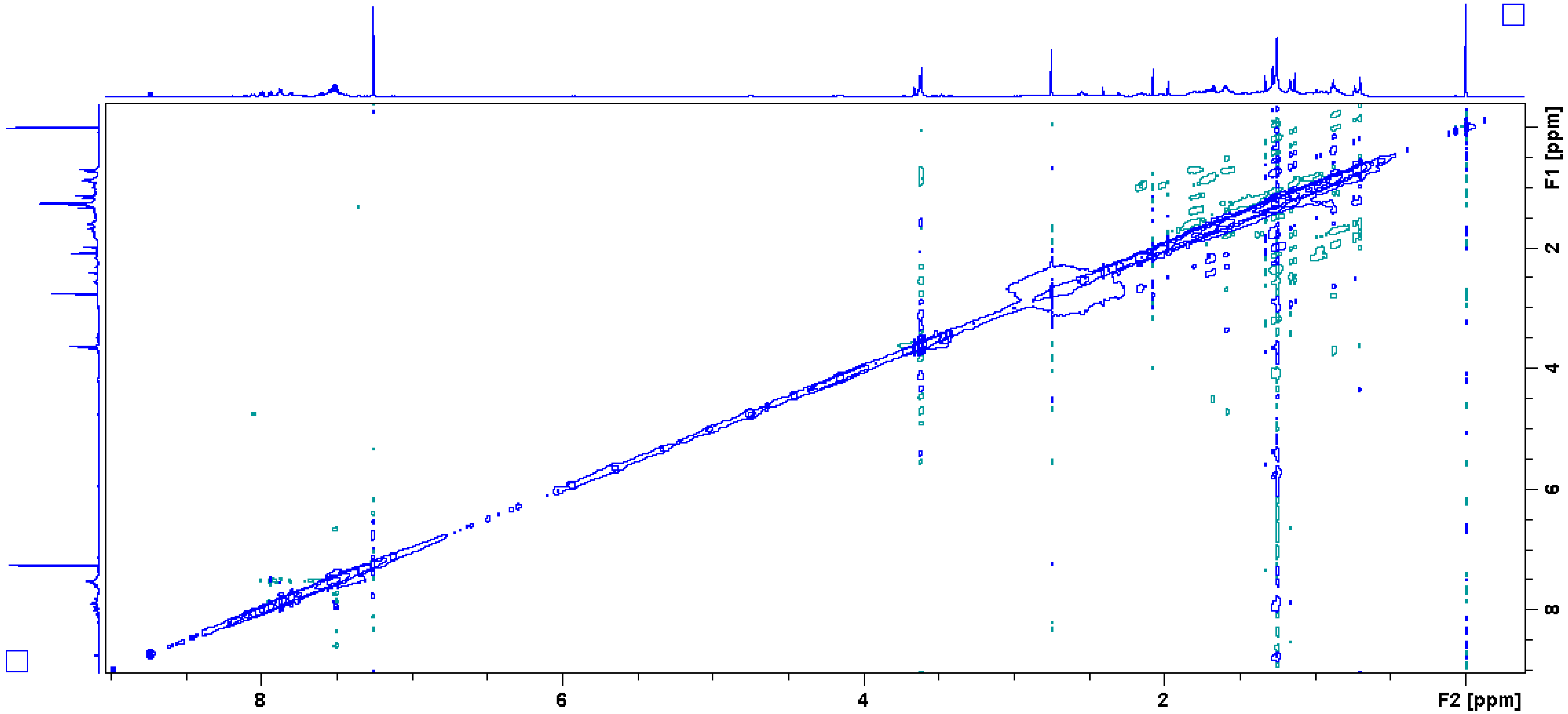

HSQC of compound (4*R*,4a*S*,6a*R*,8*R*,9*S*,11a*R*,11b*S*)-methyl 8-hydroxy-4,11b-dimethyl-9-((((*R*)-1-(naphthalen-1-yl)ethyl)amino)methyl)tetradecahydro-6a,9-methanocyclohepta[a]naphthalene-4-carboxylate **(28)**

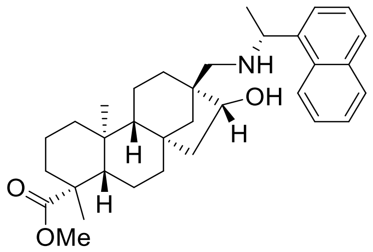

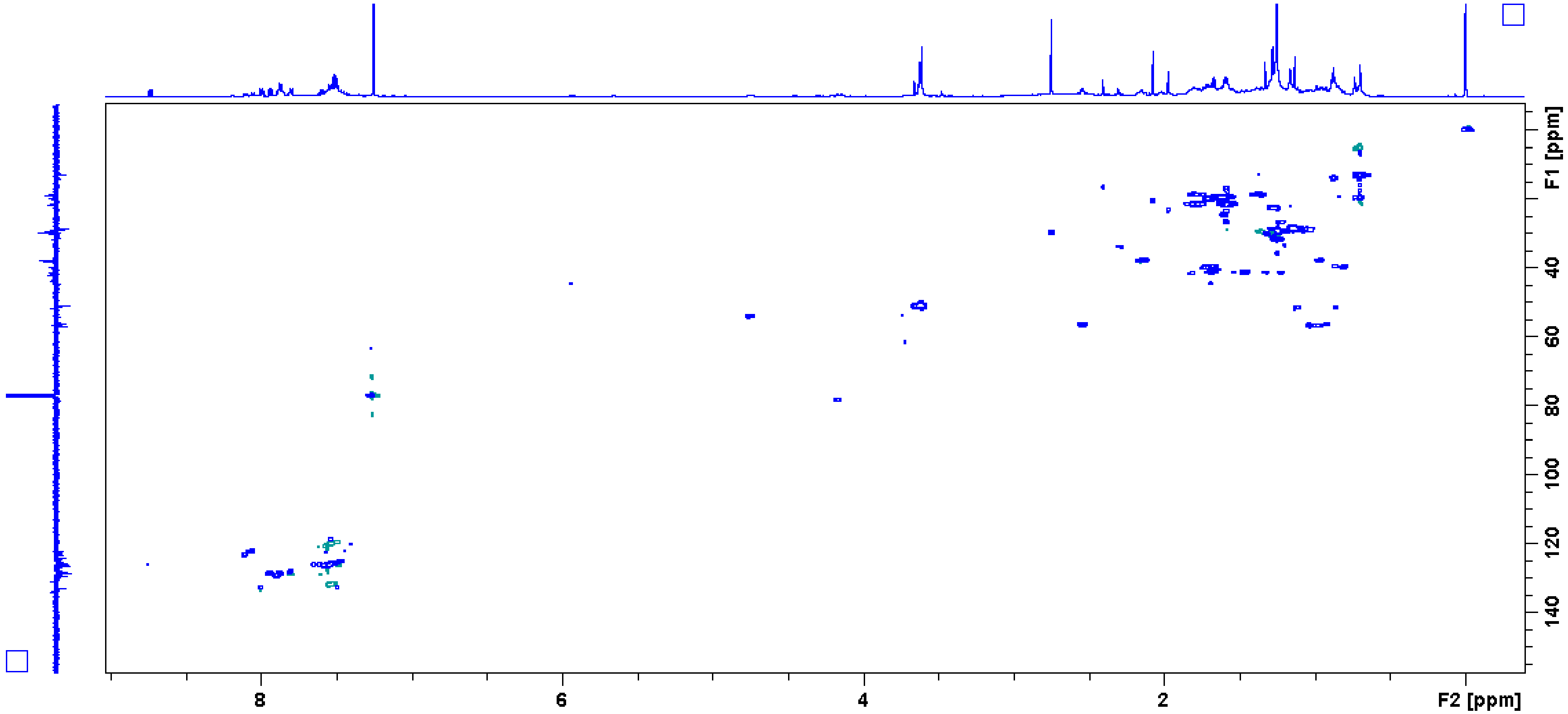

HMBC of compound (4*R*,4a*S*,6a*R*,8*R*,9*S*,11a*R*,11b*S*)-methyl 8-hydroxy-4,11b-dimethyl-9-((((*R*)-1-(naphthalen-1-yl)ethyl)amino)methyl)tetradecahydro-6a,9-methanocyclohepta[a]naphthalene-4-carboxylate **(28)**

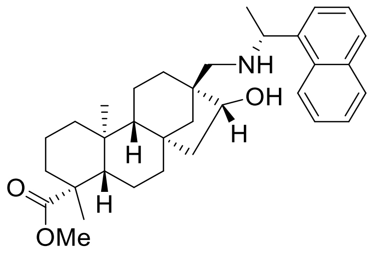

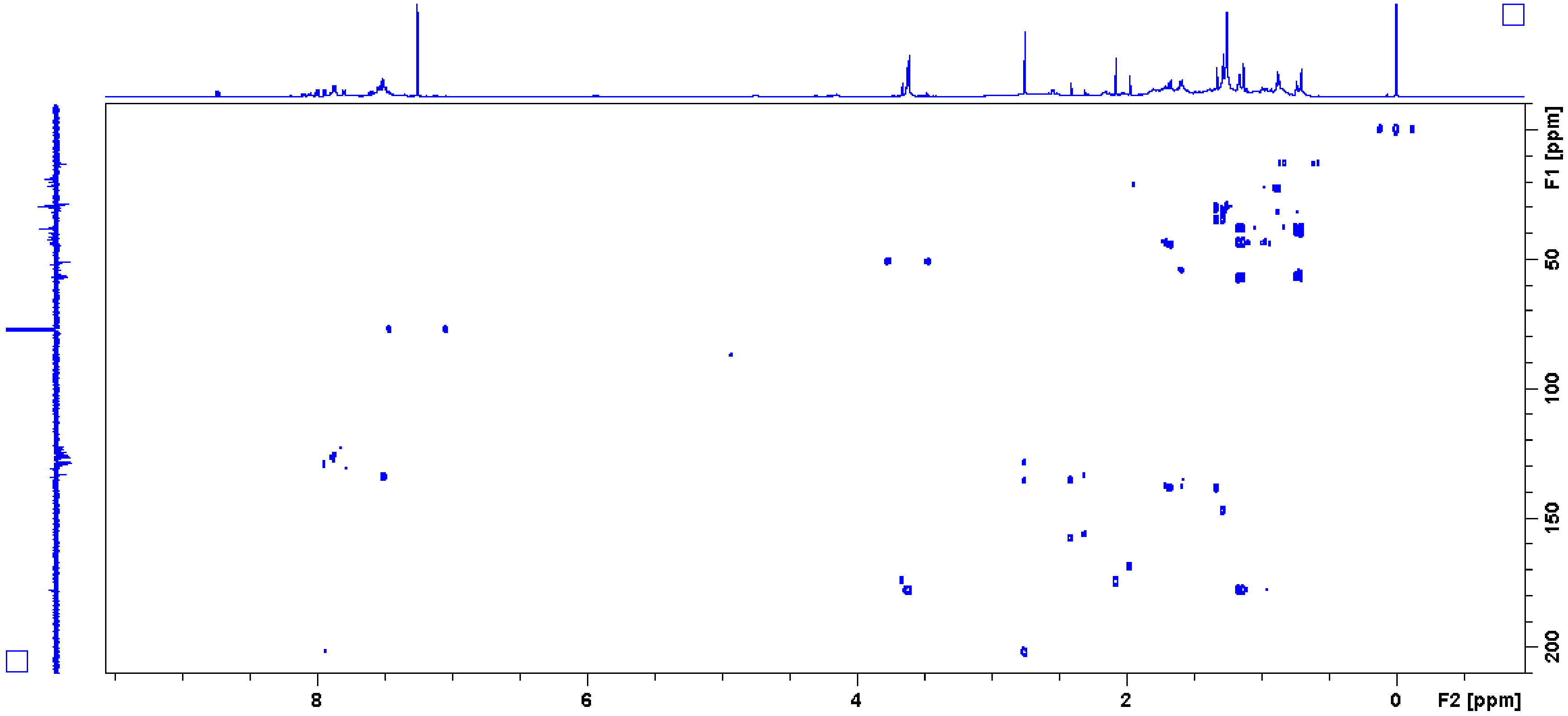

^{1}H-NMR of compound (4*R*,4a*S*,6a*R*,8*R*,9*S*,11a*R*,11b*S*)-methyl 8-hydroxy-4,11b-dimethyl-9-((((*R*)-1-(naphthalen-2-yl)ethyl)amino)methyl)tetradecahydro-6a,9-methanocyclohepta[a]naphthalene-4-carboxylate **(29)**

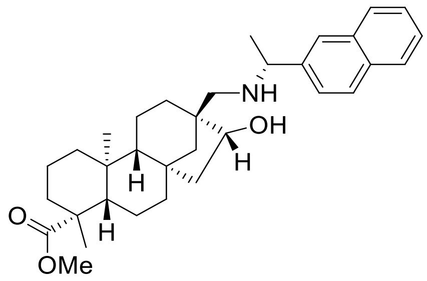

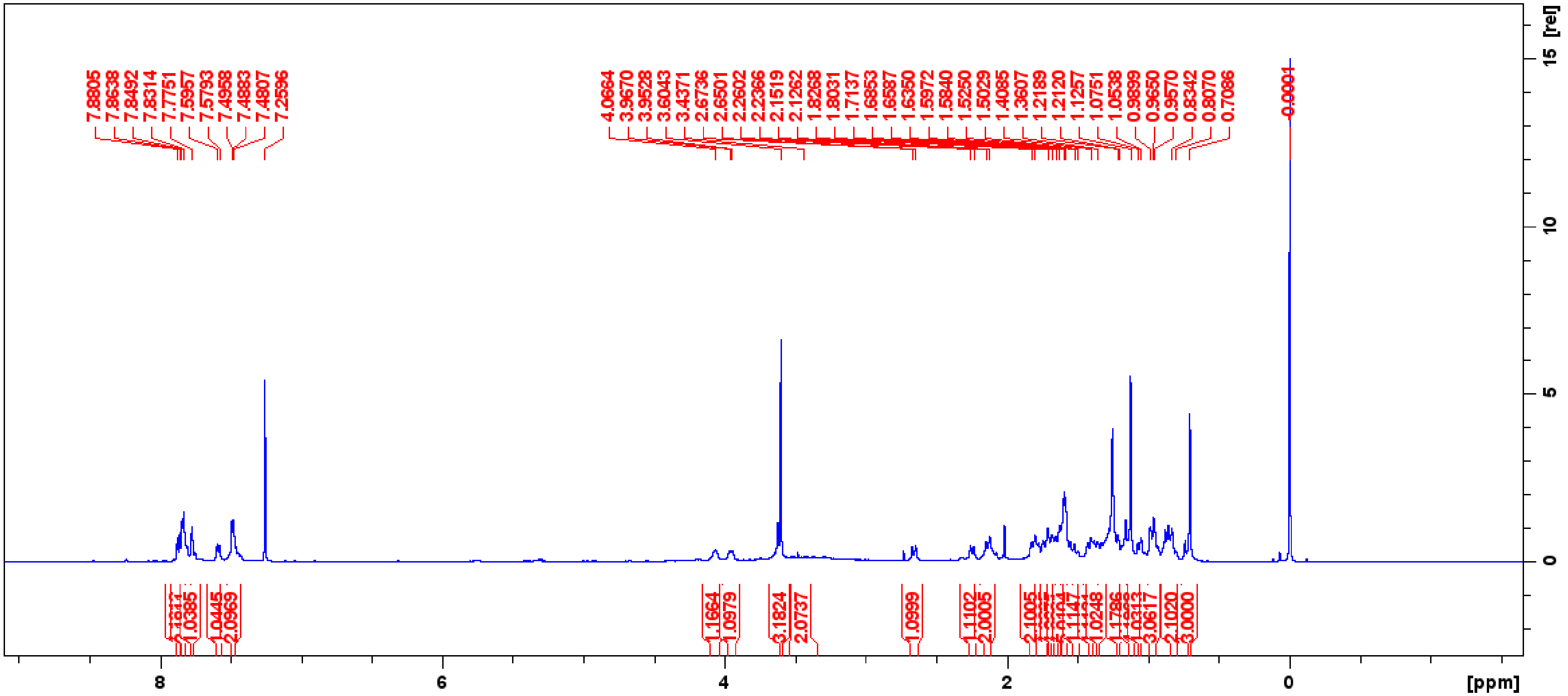

^{13}C-NMR of compound (4*R*,4a*S*,6a*R*,8*R*,9*S*,11a*R*,11b*S*)-methyl 8-hydroxy-4,11b-dimethyl-9-((((*R*)-1-(naphthalen-2-yl)ethyl)amino)methyl)tetradecahydro-6a,9-methanocyclohepta[a]naphthalene-4-carboxylate **(29)**

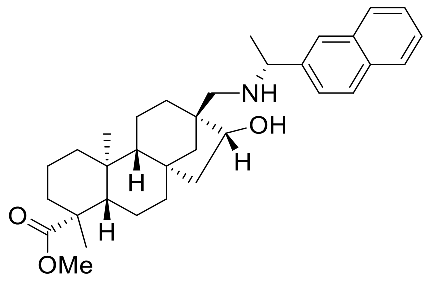

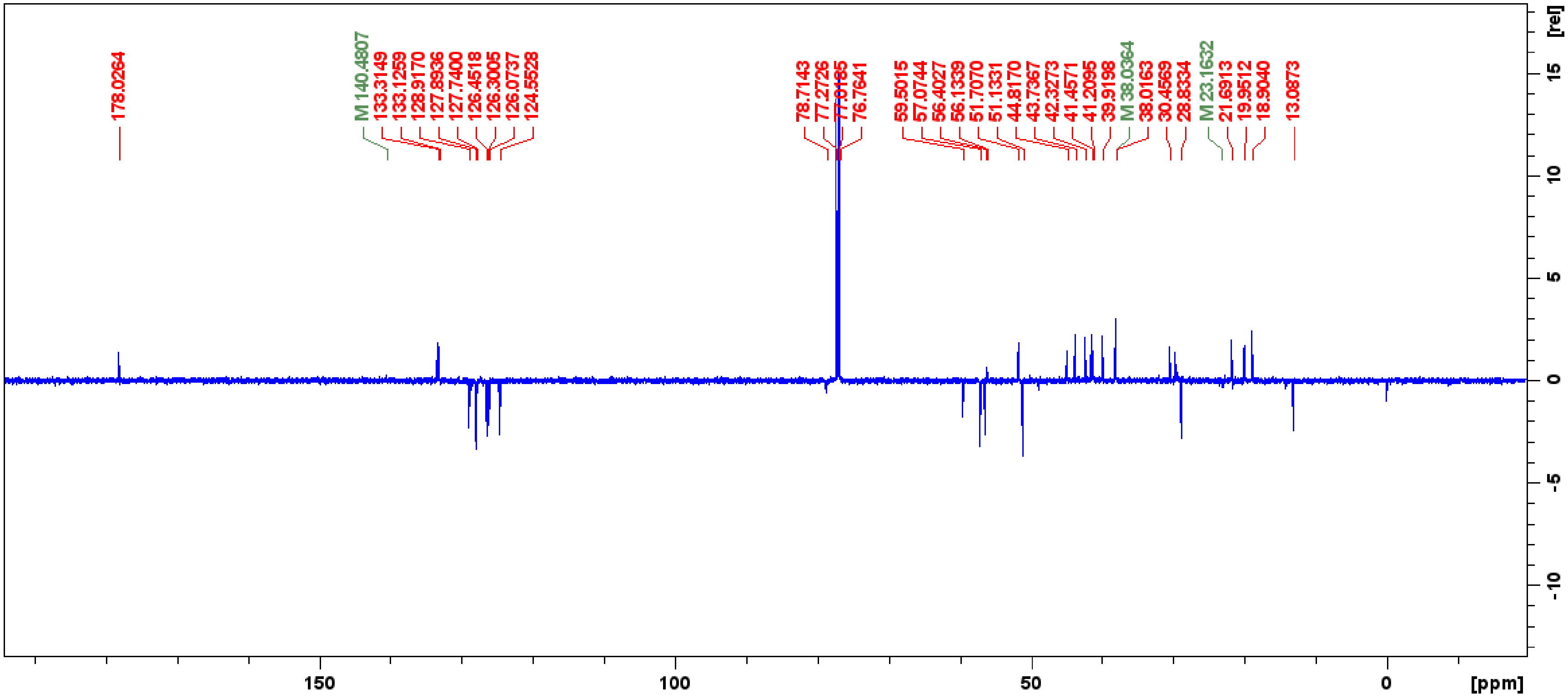

COSY of compound (4*R*,4a*S*,6a*R*,8*R*,9*S*,11a*R*,11b*S*)-methyl 8-hydroxy-4,11b-dimethyl-9-((((*R*)-1-(naphthalen-2-yl)ethyl)amino)methyl)tetradecahydro-6a,9-methanocyclohepta[a]naphthalene-4-carboxylate **(29)**

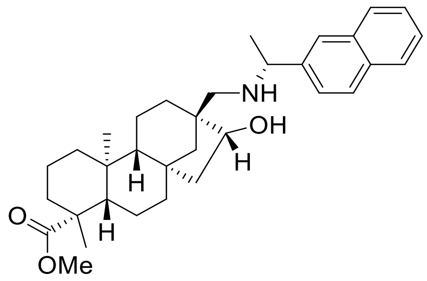

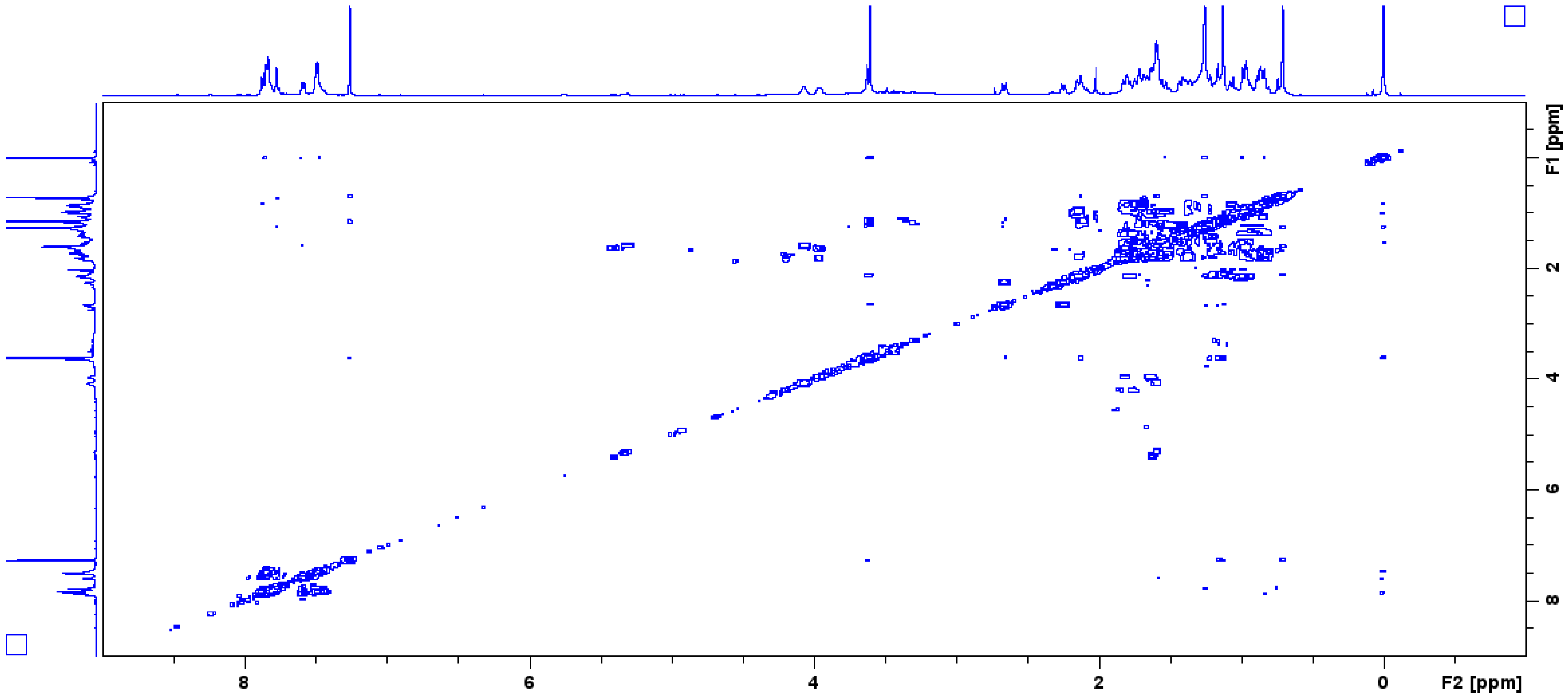

NOESY of compound (4*R*,4a*S*,6a*R*,8*R*,9*S*,11a*R*,11b*S*)-methyl 8-hydroxy-4,11b-dimethyl-9-((((*R*)-1-(naphthalen-2-yl)ethyl)amino)methyl)tetradecahydro-6a,9-methanocyclohepta[a]naphthalene-4-carboxylate **(29)**

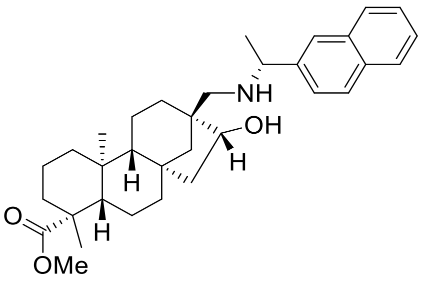

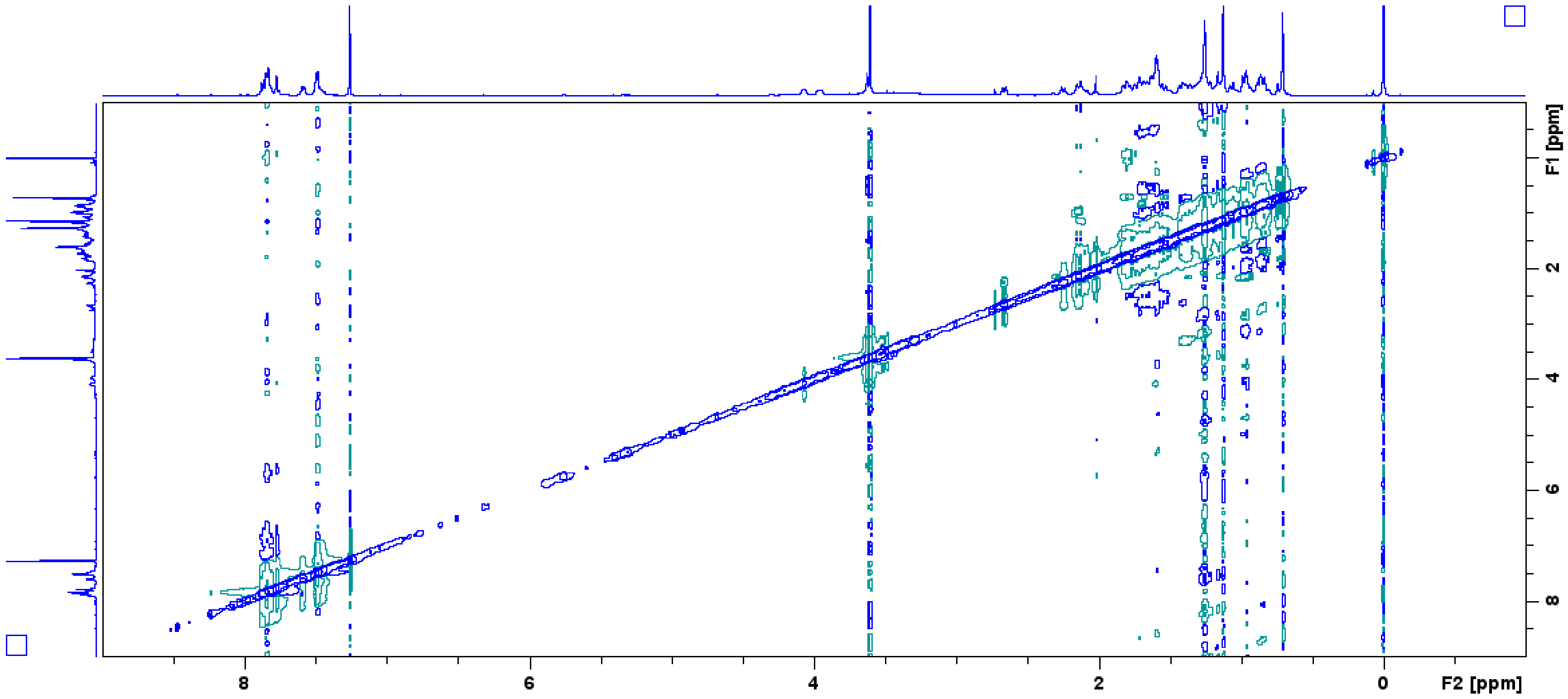

HSQC of compound (4*R*,4a*S*,6a*R*,8*R*,9*S*,11a*R*,11b*S*)-methyl 8-hydroxy-4,11b-dimethyl-9-((((*R*)-1-(naphthalen-2-yl)ethyl)amino)methyl)tetradecahydro-6a,9-methanocyclohepta[a]naphthalene-4-carboxylate **(29)**

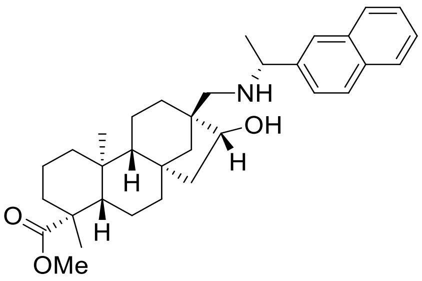

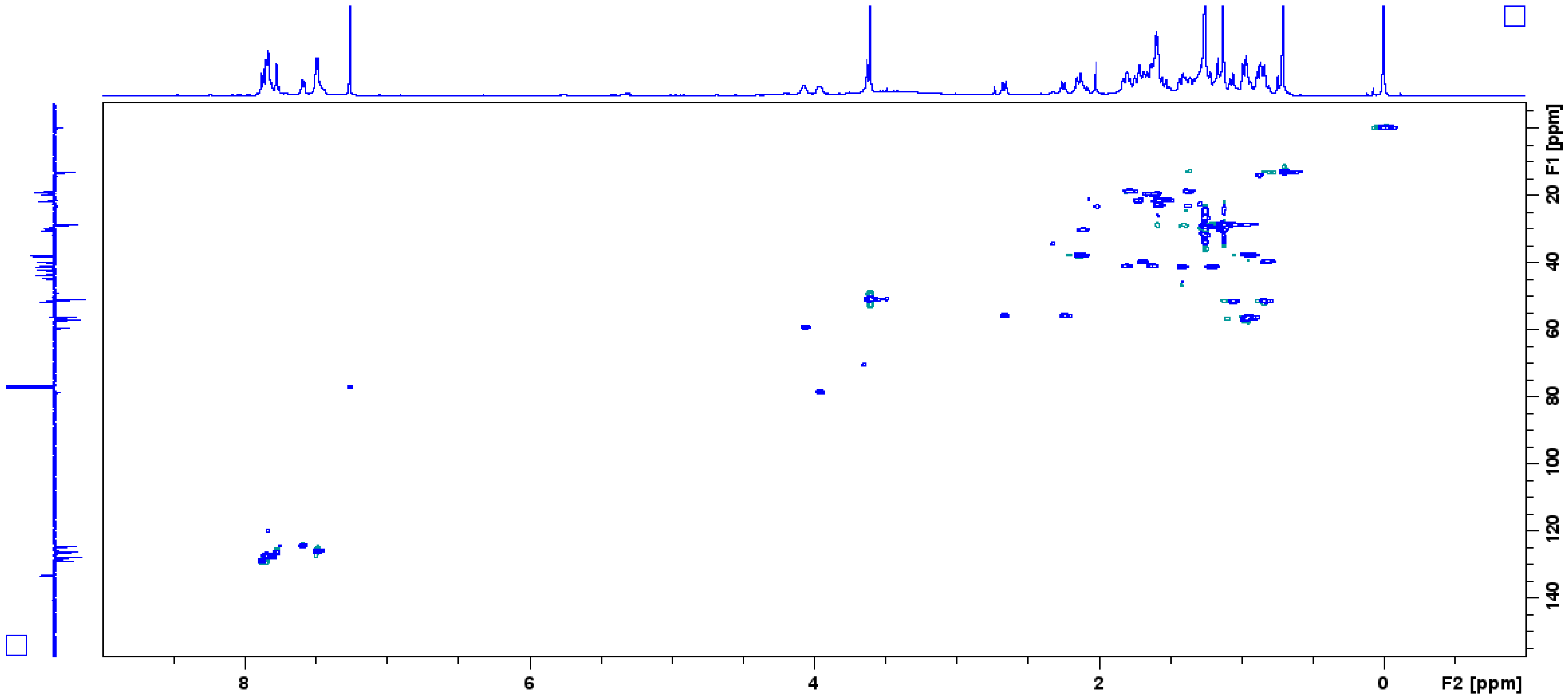

HMBC of compound (4*R*,4a*S*,6a*R*,8*R*,9*S*,11a*R*,11b*S*)-methyl 8-hydroxy-4,11b-dimethyl-9-((((*R*)-1-(naphthalen-2-yl)ethyl)amino)methyl)tetradecahydro-6a,9-methanocyclohepta[a]naphthalene-4-carboxylate **(29)**

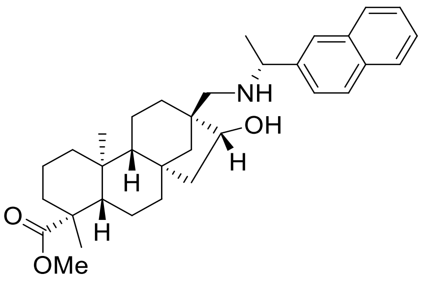

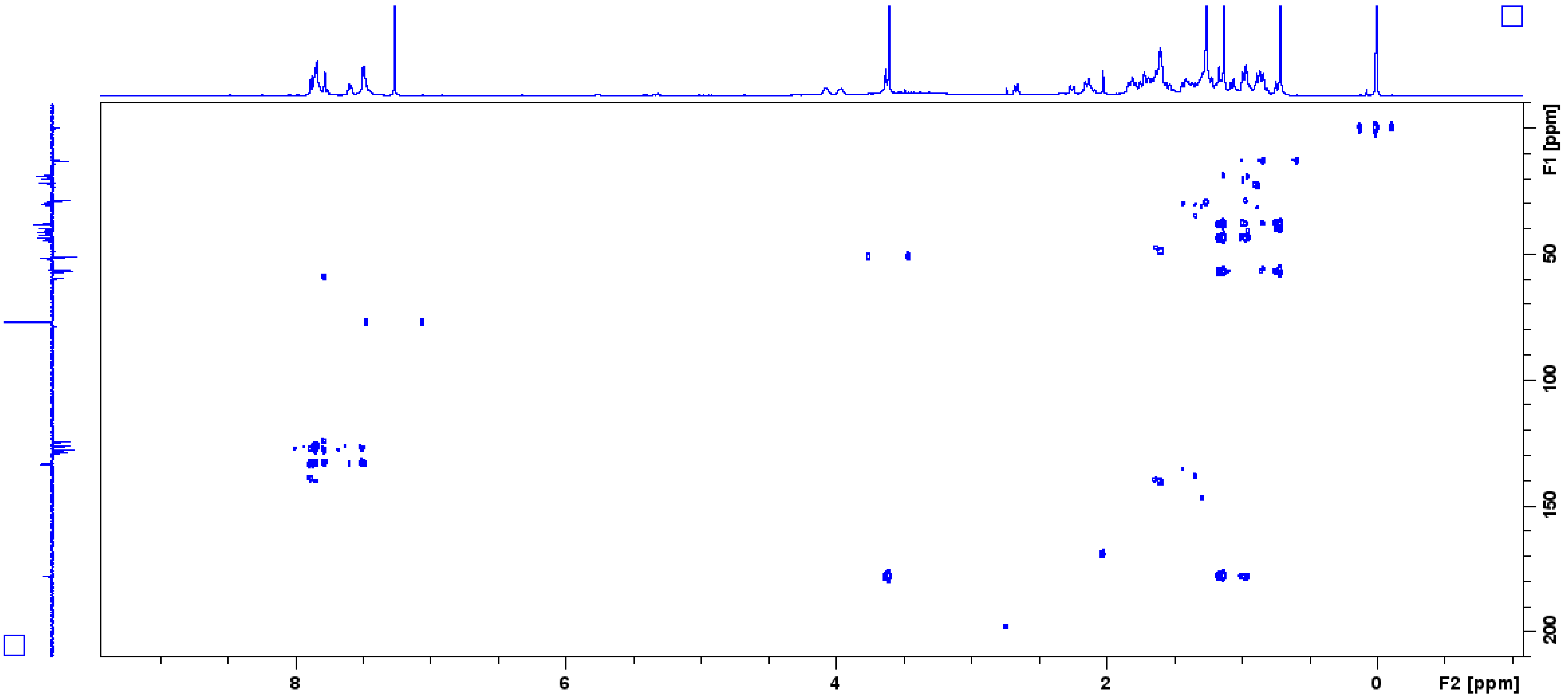

^{1}H-NMR of compound (4*R*,4a*S*,6a*R*,8*R*,9*S*,11a*R*,11b*S*)-methyl 8-hydroxy-4,11b-dimethyl-9-((((*S*)-1-(naphthalen-2-yl)ethyl)amino)methyl)tetradecahydro-6a,9-methanocyclohepta[a]naphthalene-4-carboxylate **(30)**

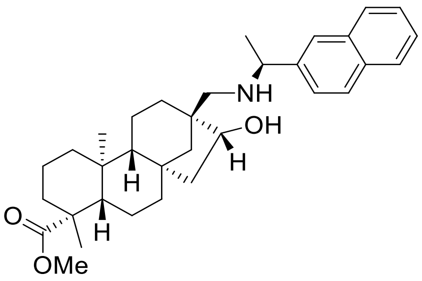

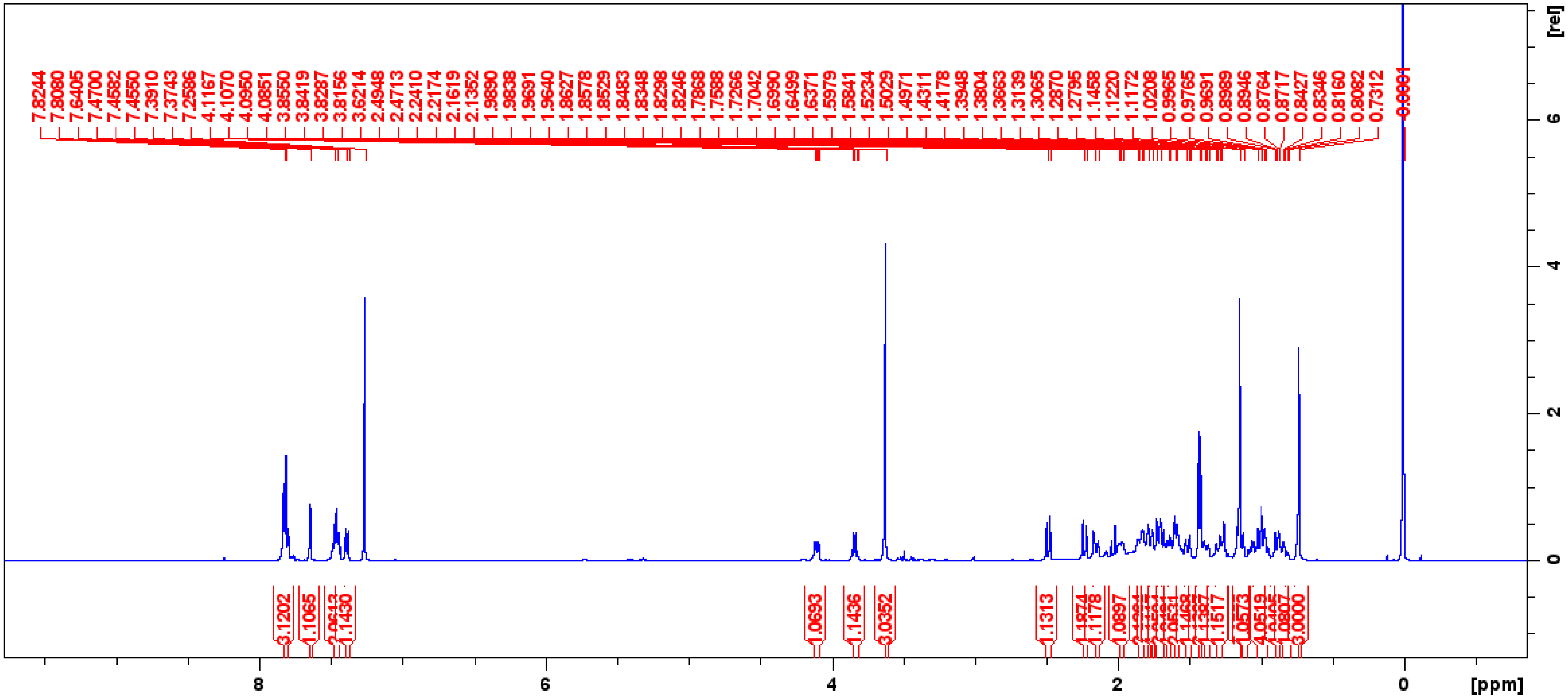

^{13}C-NMR of compound (4*R*,4a*S*,6a*R*,8*R*,9*S*,11a*R*,11b*S*)-methyl 8-hydroxy-4,11b-dimethyl-9-((((*S*)-1-(naphthalen-2-yl)ethyl)amino)methyl)tetradecahydro-6a,9-methanocyclohepta[a]naphthalene-4-carboxylate **(30)**

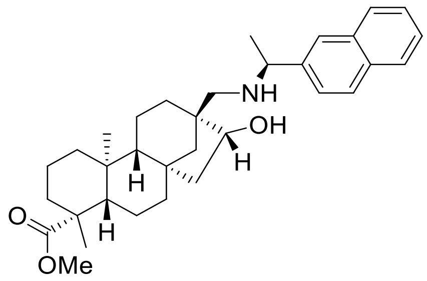

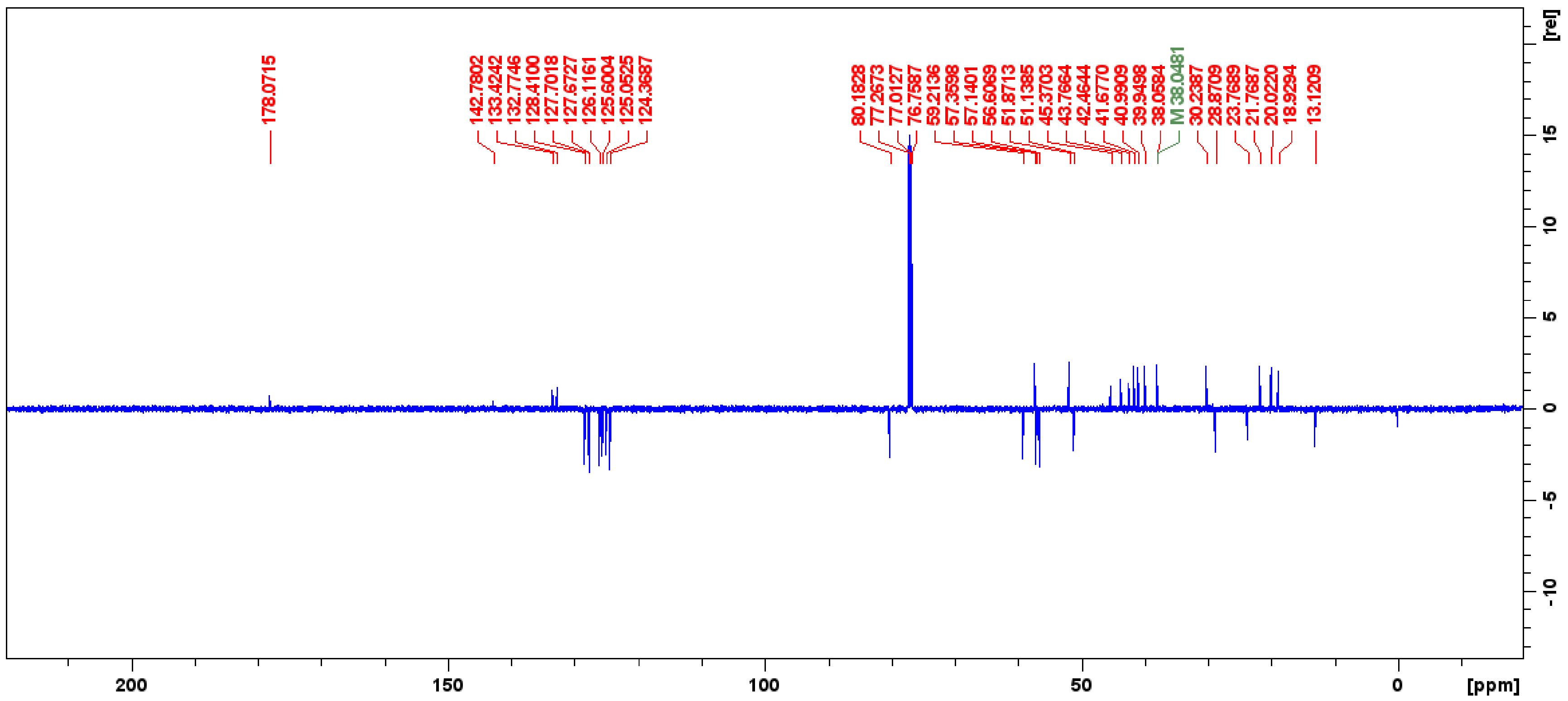

COSY of compound (4*R*,4a*S*,6a*R*,8*R*,9*S*,11a*R*,11b*S*)-methyl 8-hydroxy-4,11b-dimethyl-9-((((*S*)-1-(naphthalen-2-yl)ethyl)amino)methyl)tetradecahydro-6a,9-methanocyclohepta[a]naphthalene-4-carboxylate **(30)**

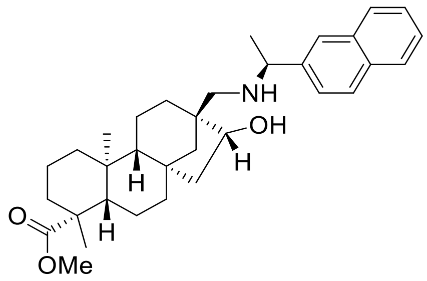

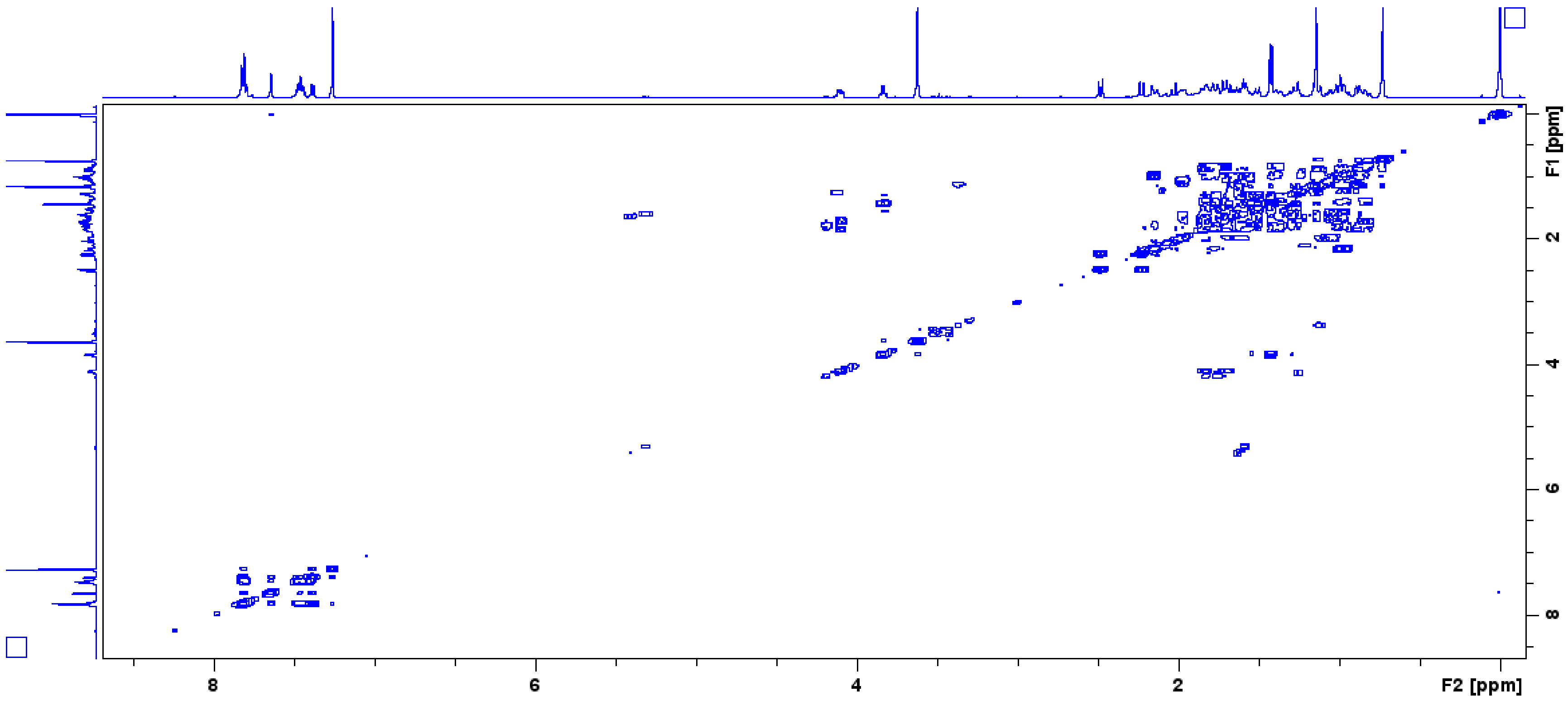

NOESY of compound (4*R*,4a*S*,6a*R*,8*R*,9*S*,11a*R*,11b*S*)-methyl 8-hydroxy-4,11b-dimethyl-9-((((*S*)-1-(naphthalen-2-yl)ethyl)amino)methyl)tetradecahydro-6a,9-methanocyclohepta[a]naphthalene-4-carboxylate **(30)**

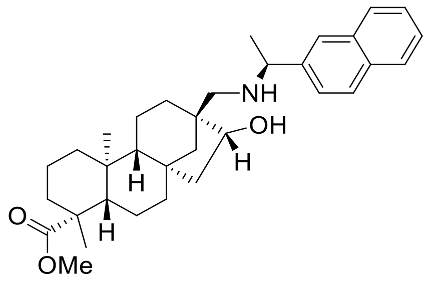

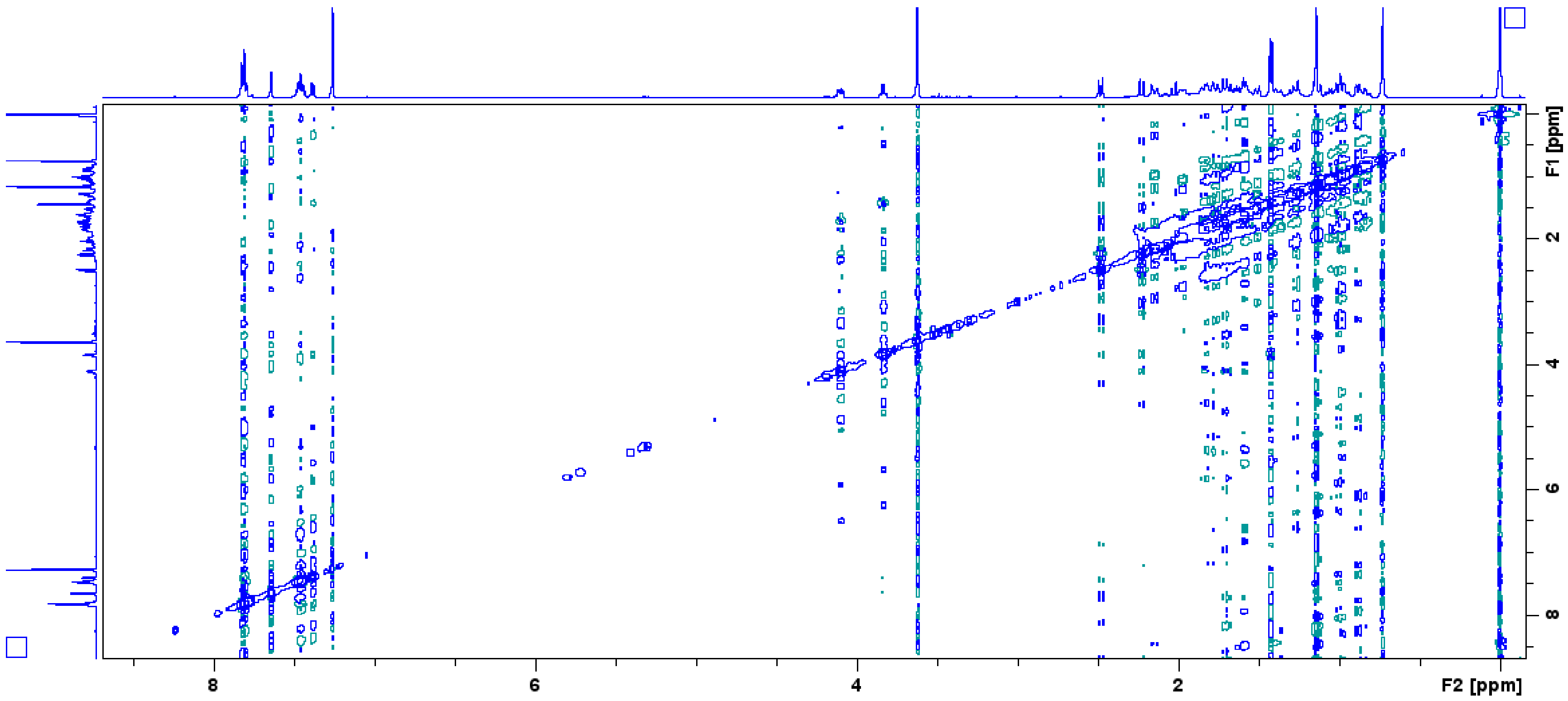

HSQC of compound (4*R*,4a*S*,6a*R*,8*R*,9*S*,11a*R*,11b*S*)-methyl 8-hydroxy-4,11b-dimethyl-9-((((*S*)-1-(naphthalen-2-yl)ethyl)amino)methyl)tetradecahydro-6a,9-methanocyclohepta[a]naphthalene-4-carboxylate **(30)**

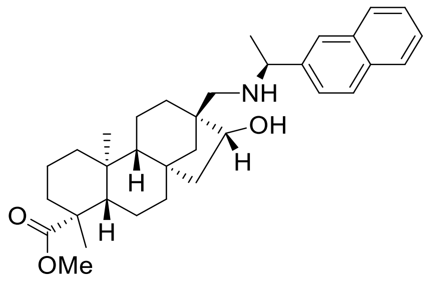

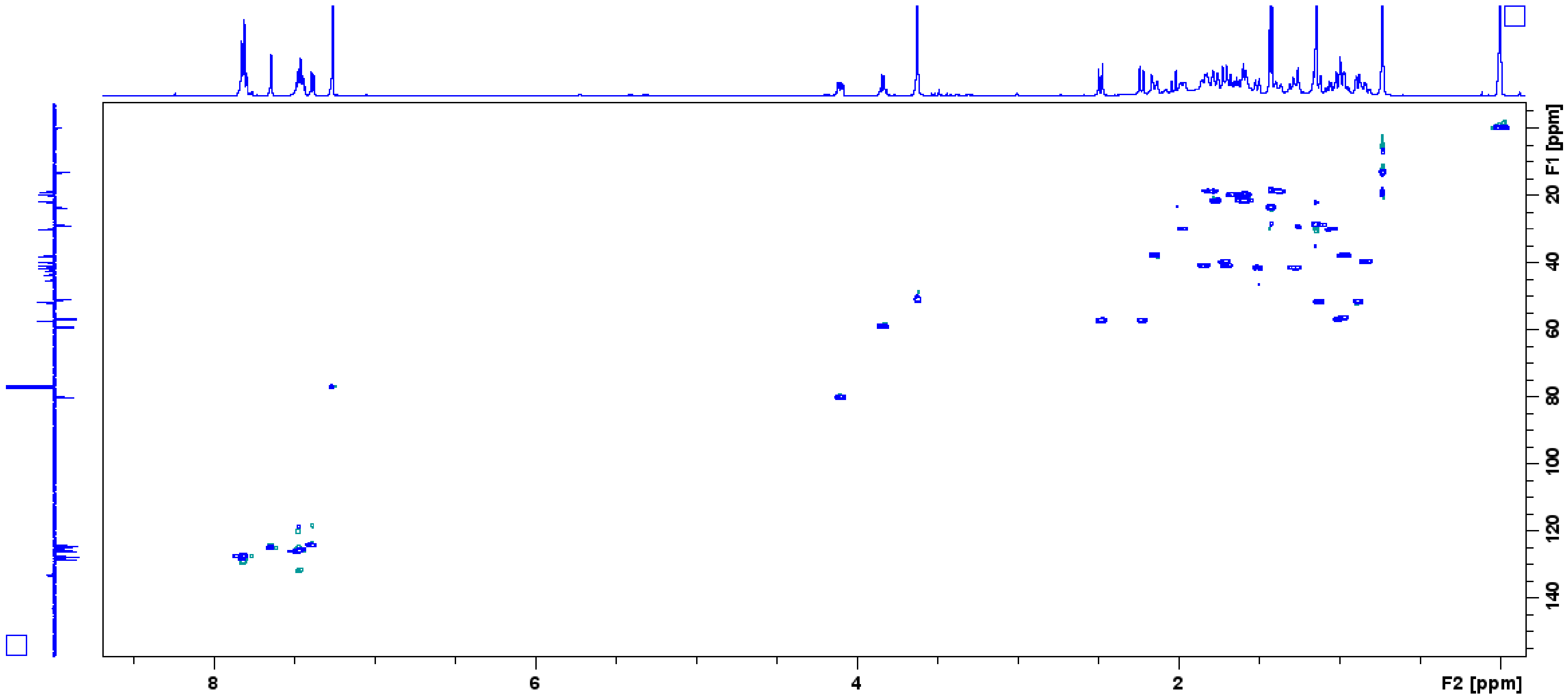

HMBC of compound (4*R*,4a*S*,6a*R*,8*R*,9*S*,11a*R*,11b*S*)-methyl 8-hydroxy-4,11b-dimethyl-9-((((*S*)-1-(naphthalen-2-yl)ethyl)amino)methyl)tetradecahydro-6a,9-methanocyclohepta[a]naphthalene-4-carboxylate **(30)**

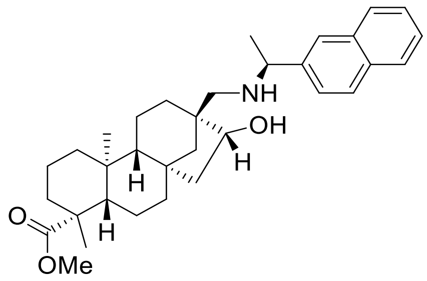

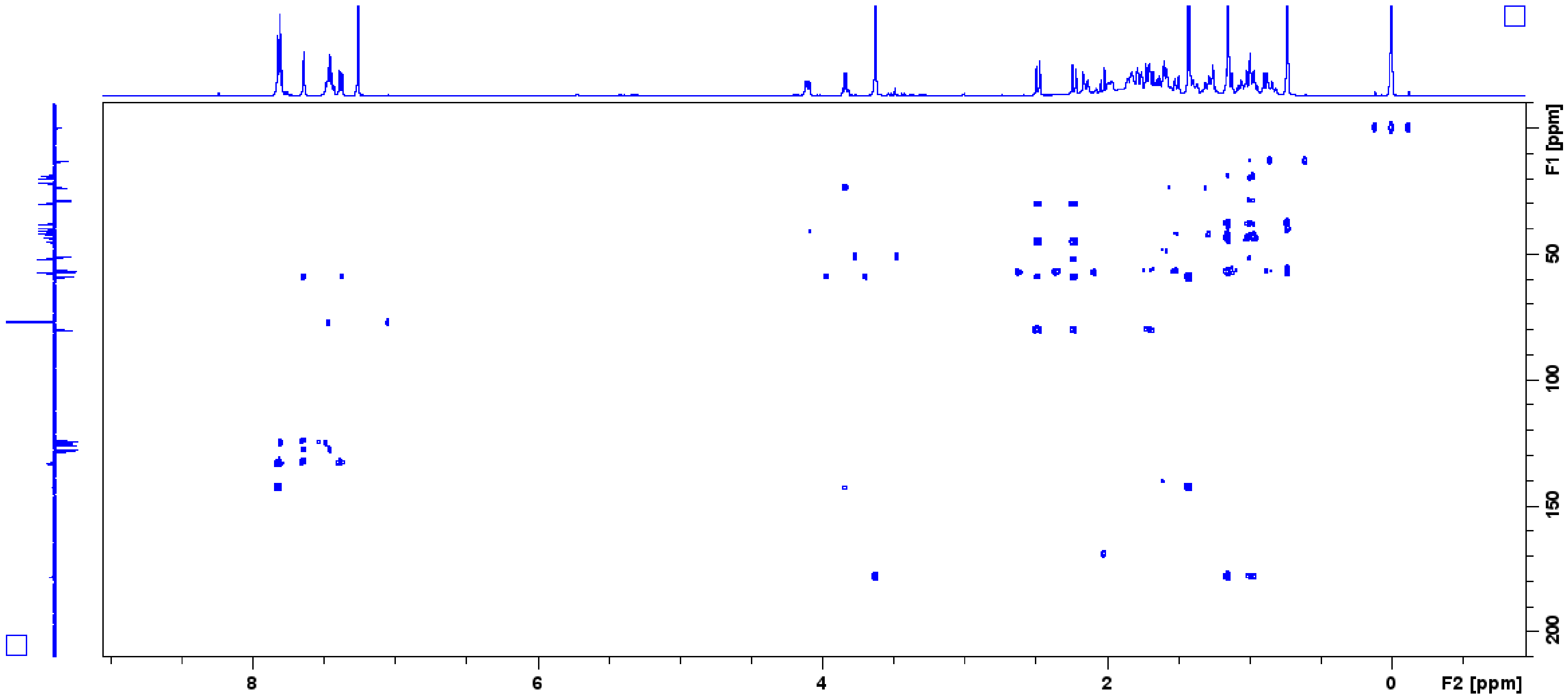

^{1}H-NMR of compound (4*R*,4a*S*,6a*R*,8*R*,9*S*,11a*R*,11b*S*)-methyl 9-(aminomethyl)-8-hydroxy-4,11b-dimethyltetradecahydro-6a,9-methanocyclohepta[a]naphthalene-4-carboxylate **(31)**

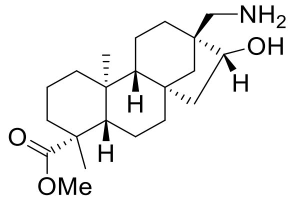

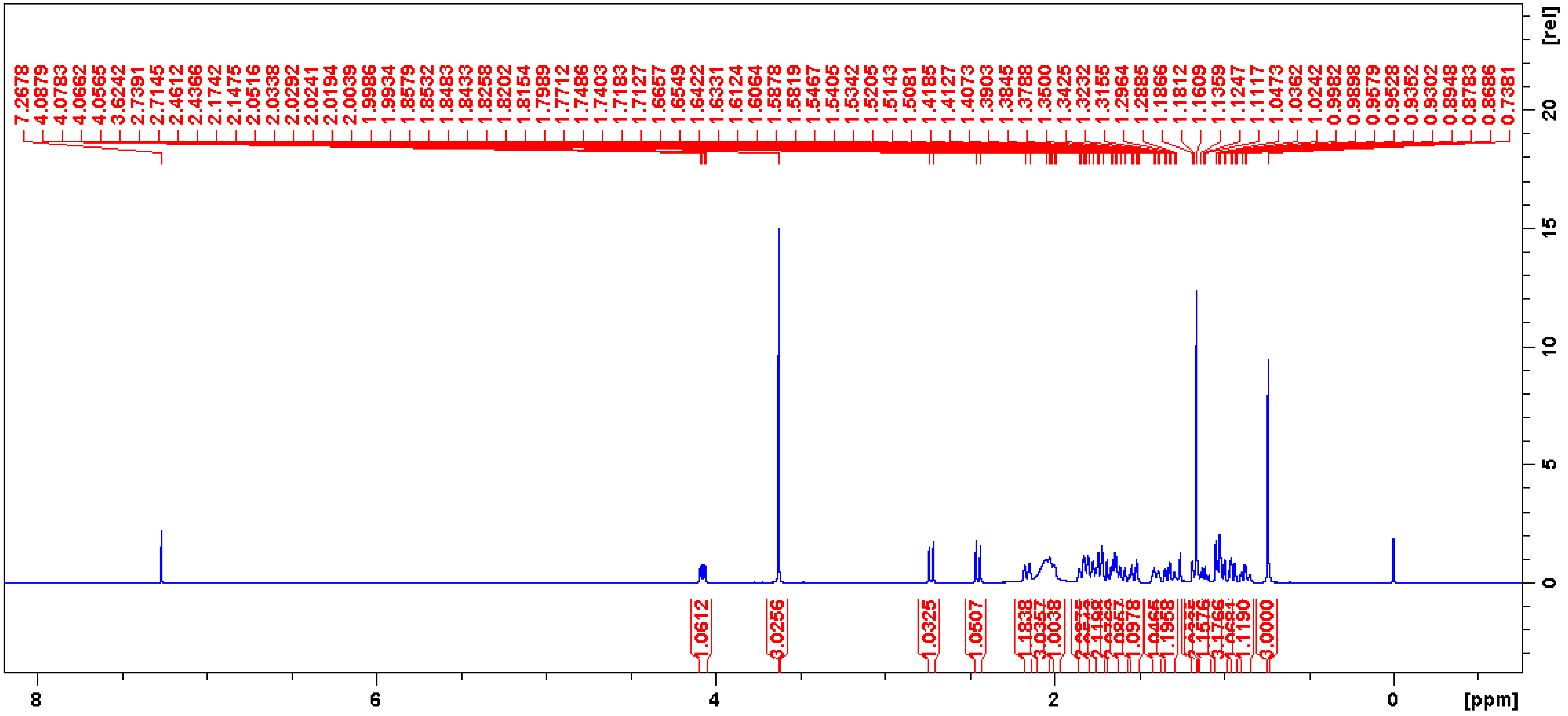

^{13}C-NMR of compound (4*R*,4a*S*,6a*R*,8*R*,9*S*,11a*R*,11b*S*)-methyl 9-(aminomethyl)-8-hydroxy-4,11b-dimethyltetradecahydro-6a,9-methanocyclohepta[a]naphthalene-4-carboxylate **(31)**

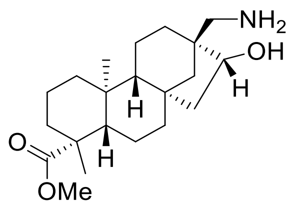

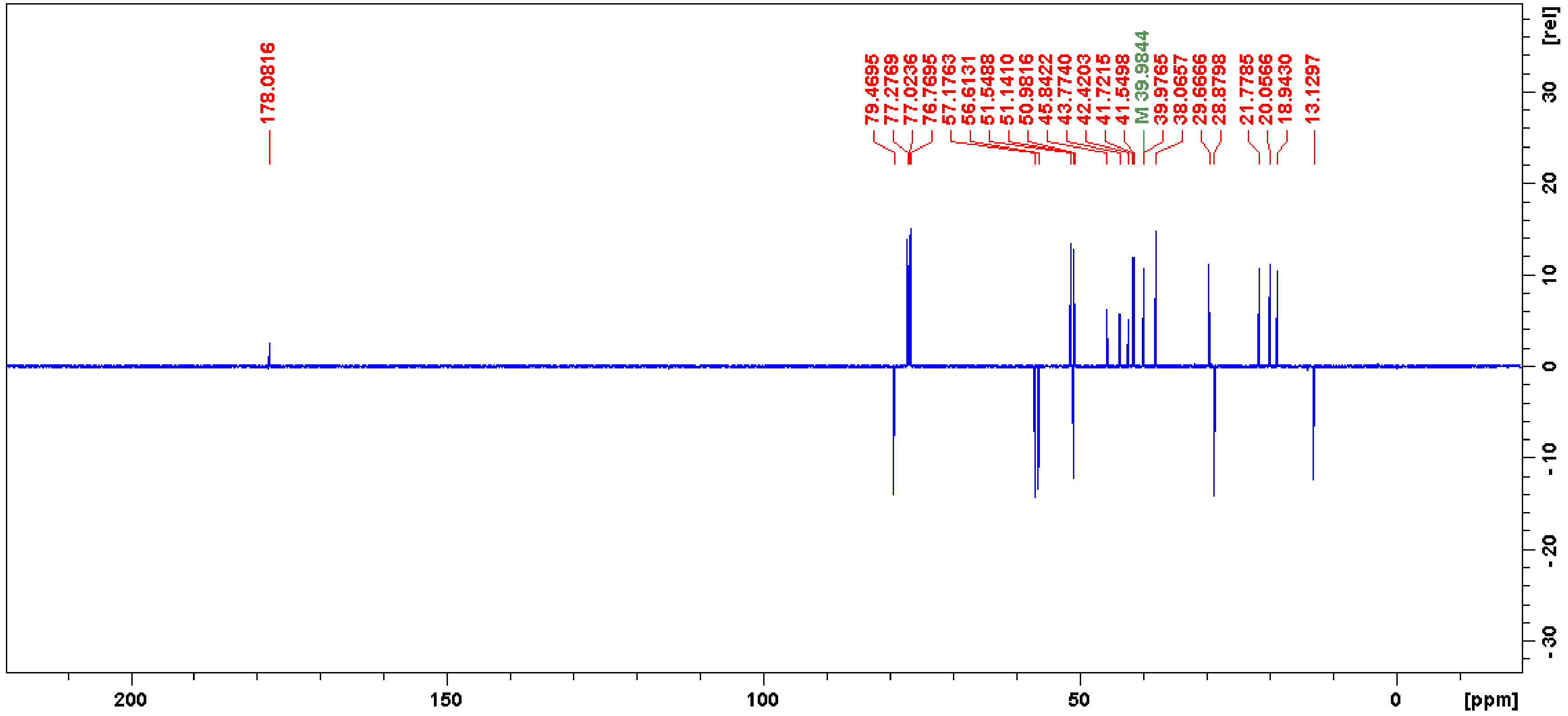

COSY of compound (4*R*,4a*S*,6a*R*,8*R*,9*S*,11a*R*,11b*S*)-methyl 9-(aminomethyl)-8-hydroxy-4,11b-dimethyltetradecahydro-6a,9-methanocyclohepta[a]naphthalene-4-carboxylate **(31)**

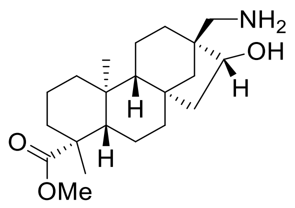

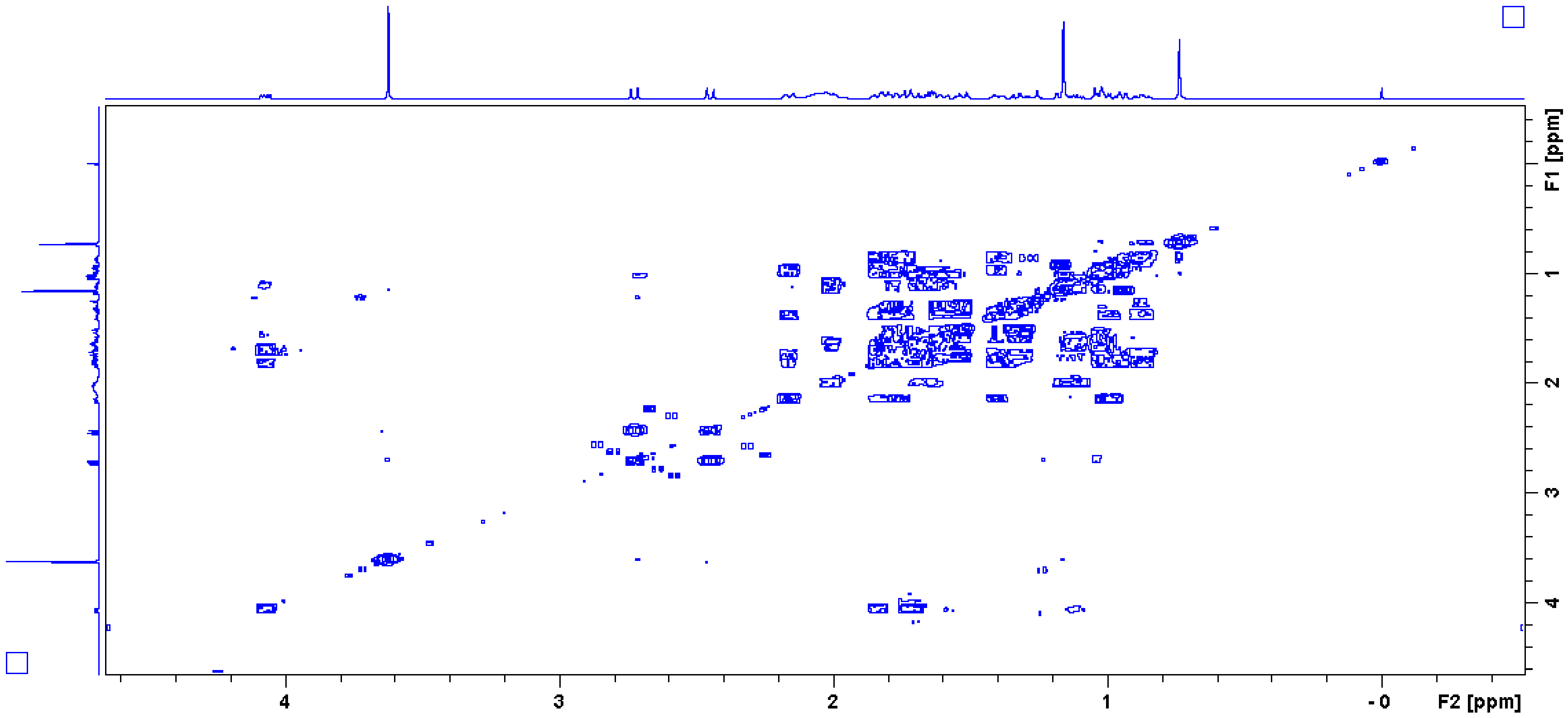

NOESY of compound (4*R*,4a*S*,6a*R*,8*R*,9*S*,11a*R*,11b*S*)-methyl 9-(aminomethyl)-8-hydroxy-4,11b-dimethyltetradecahydro-6a,9-methanocyclohepta[a]naphthalene-4-carboxylate **(31)**

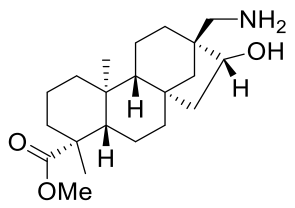

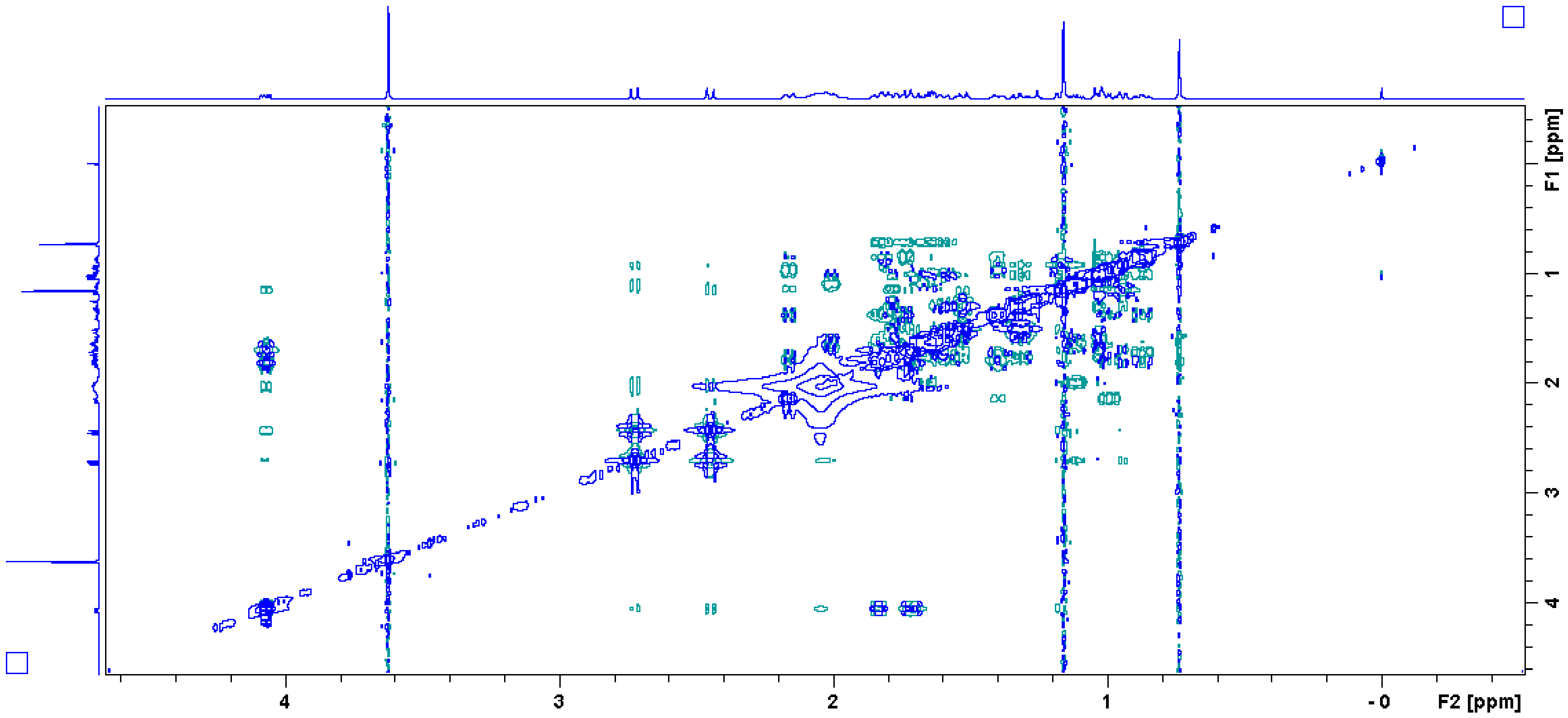

HSQC of compound (4*R*,4a*S*,6a*R*,8*R*,9*S*,11a*R*,11b*S*)-methyl 9-(aminomethyl)-8-hydroxy-4,11b-dimethyltetradecahydro-6a,9-methanocyclohepta[a]naphthalene-4-carboxylate **(31)**

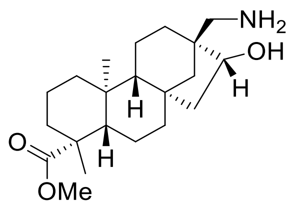

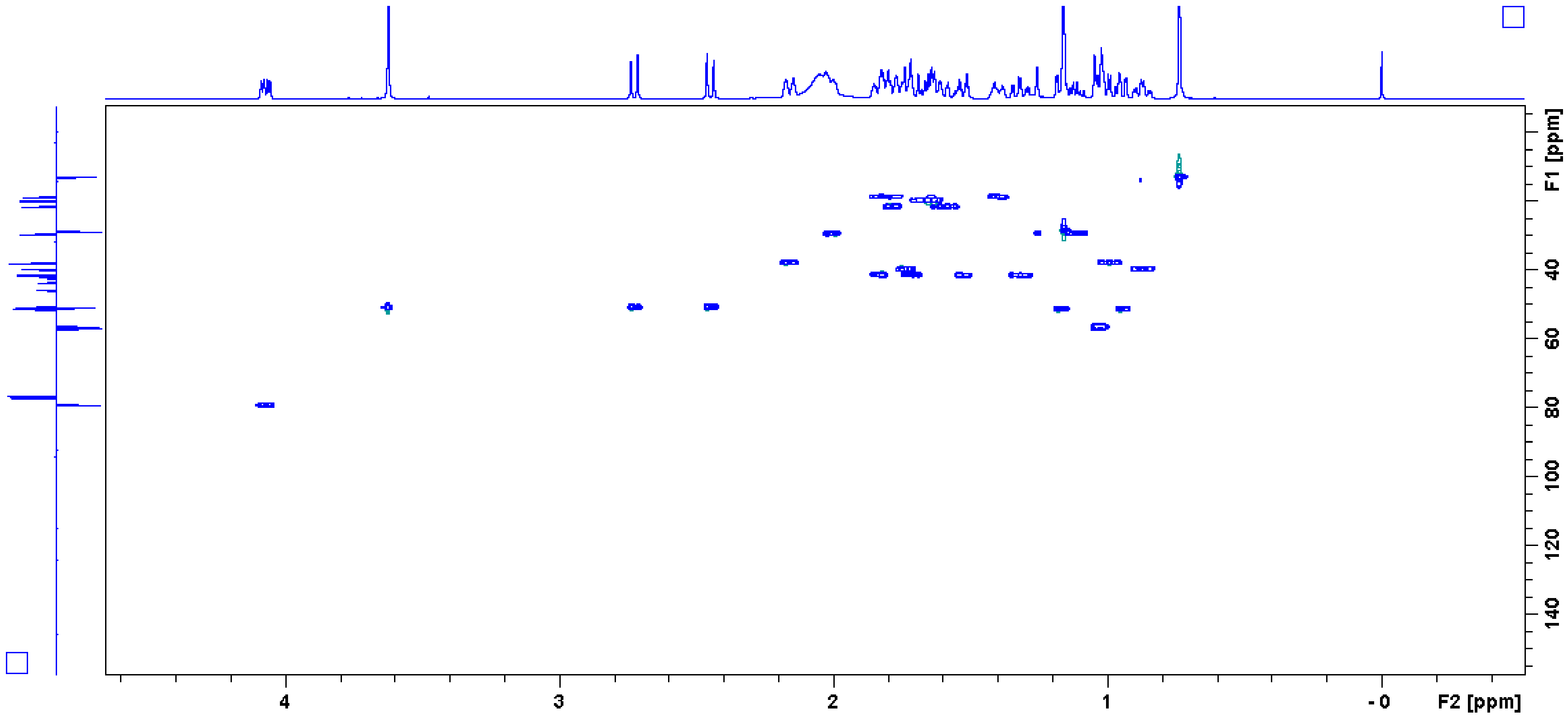

HMBC of compound (4*R*,4a*S*,6a*R*,8*R*,9*S*,11a*R*,11b*S*)-methyl 9-(aminomethyl)-8-hydroxy-4,11b-dimethyltetradecahydro-6a,9-methanocyclohepta[a]naphthalene-4-carboxylate **(31)**

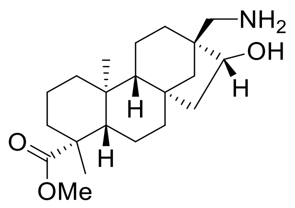

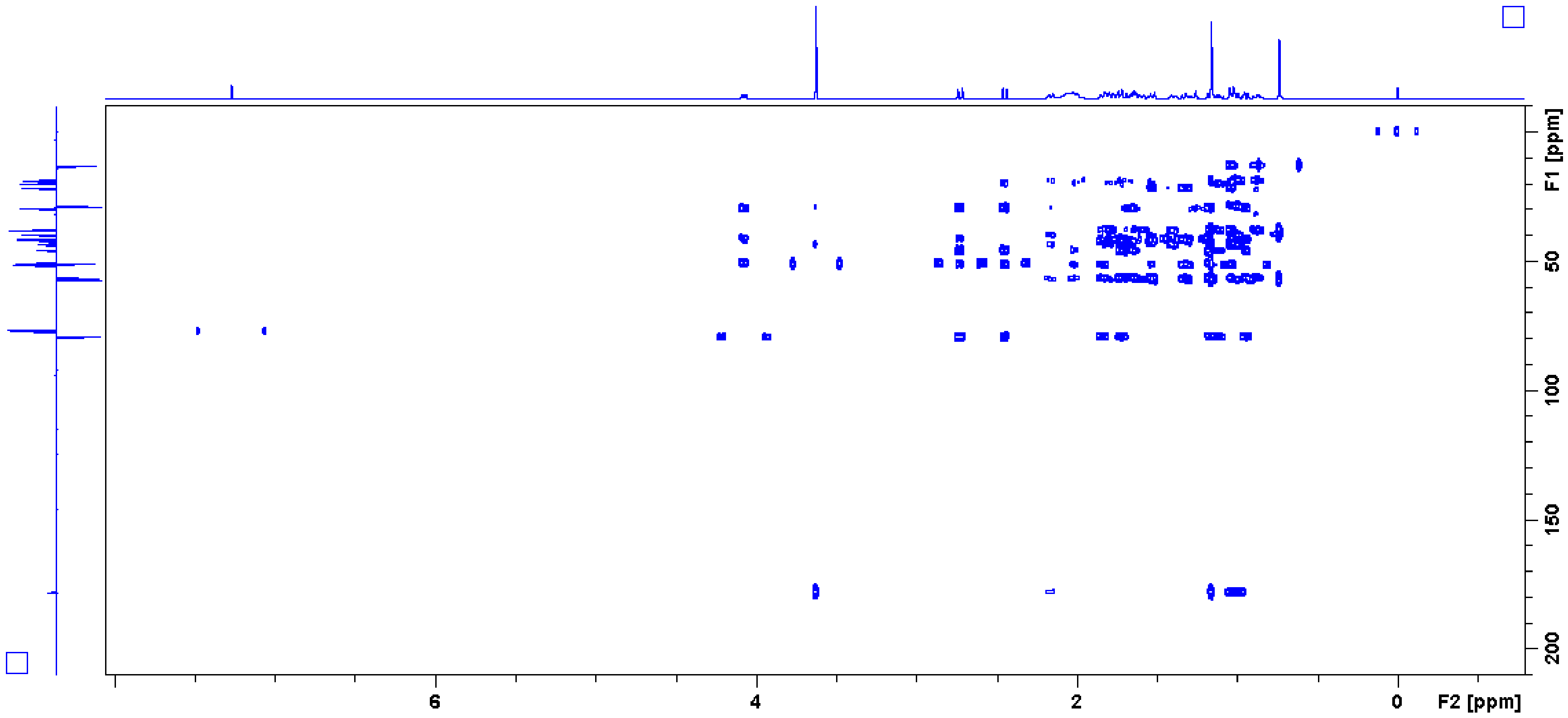

^{1}H-NMR of compound (4*R*,4a*S*,6a*R*,8*R*,9*S*,11a*R*,11b*S*)-methyl 8-hydroxy-9-(((4-methoxybenzyl)amino)methyl)-4,11b-dimethyltetradecahydro-6a,9-methanocyclohepta[a]naphthalene-4-carboxylate **(32)**

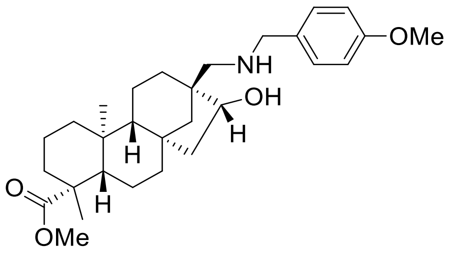

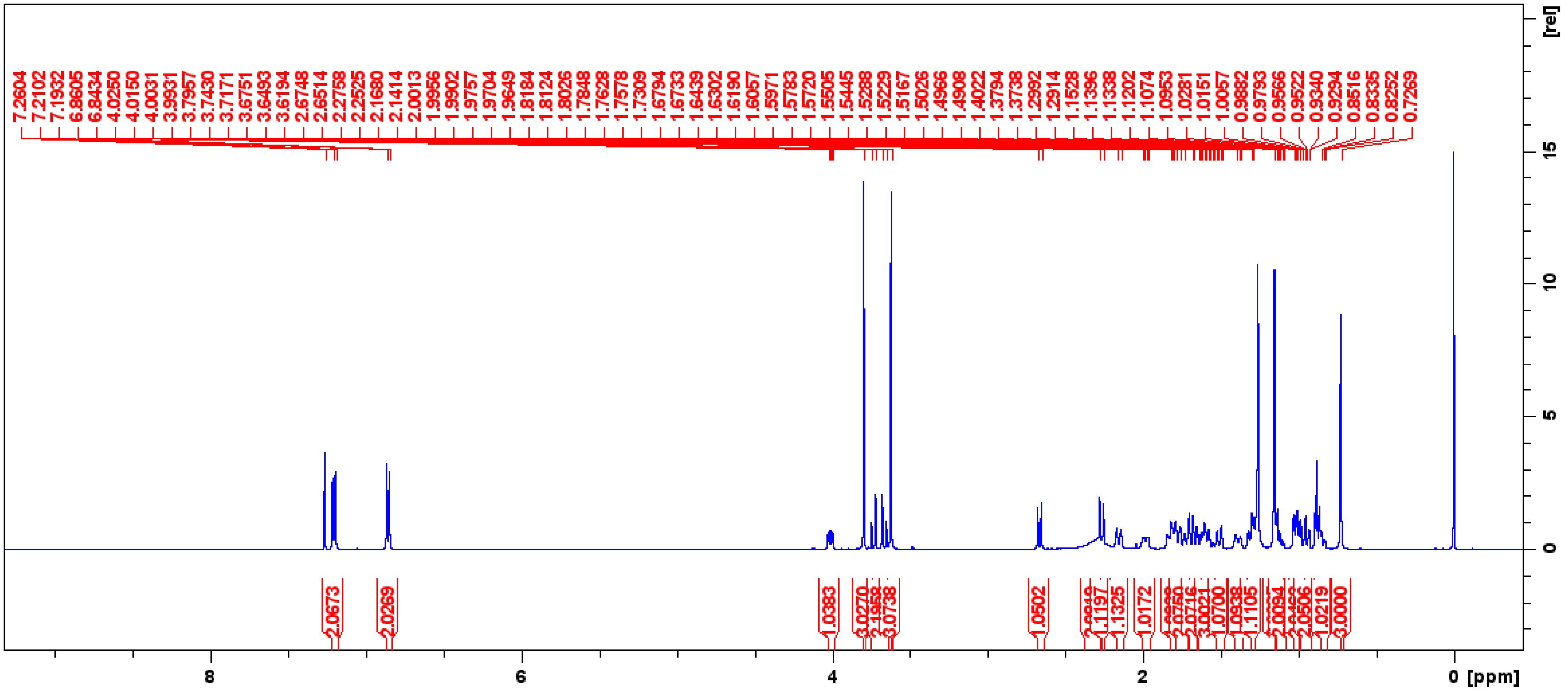

^{13}C-NMR of compound (4*R*,4a*S*,6a*R*,8*R*,9*S*,11a*R*,11b*S*)-methyl 8-hydroxy-9-(((4-methoxybenzyl)amino)methyl)-4,11b-dimethyltetradecahydro-6a,9-methanocyclohepta[a]naphthalene-4-carboxylate **(32)**

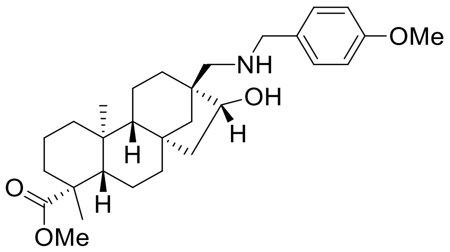

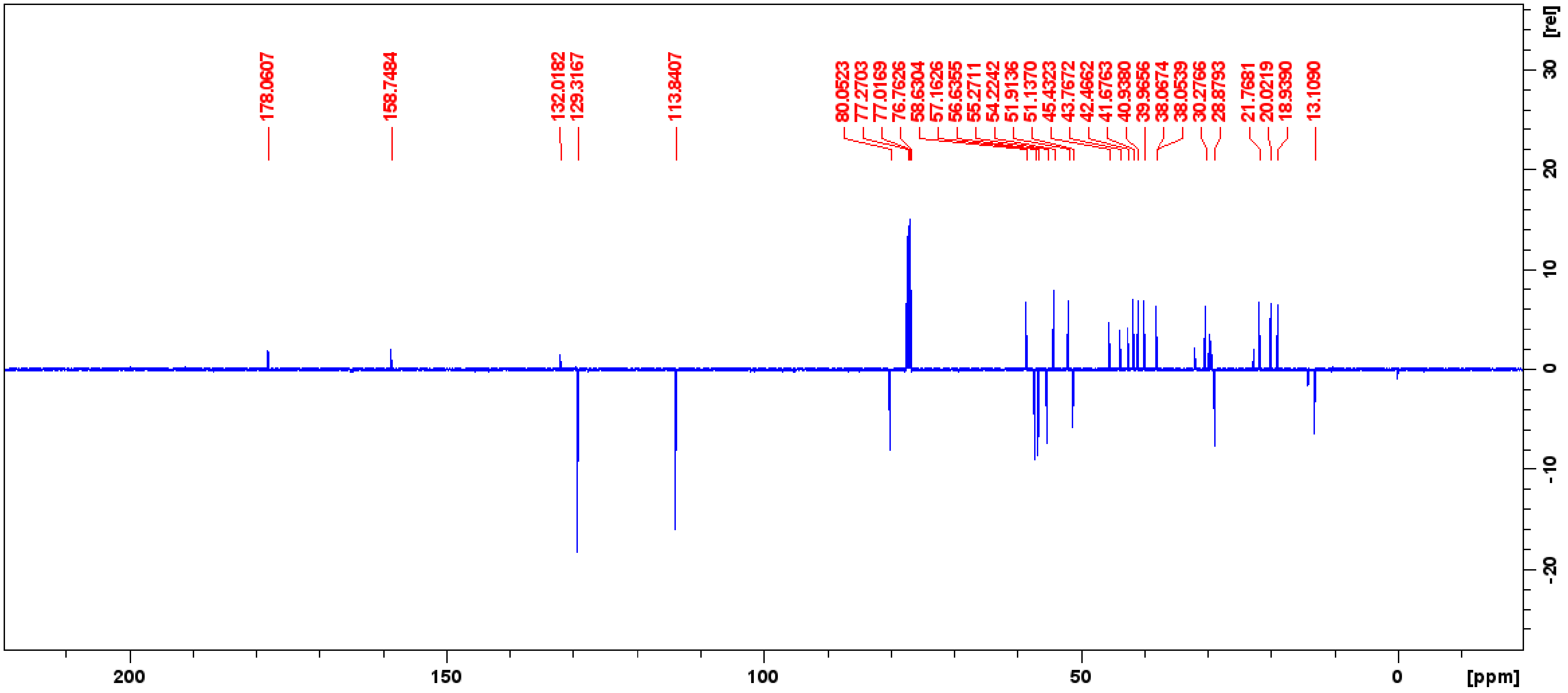

COSY of compound (4*R*,4a*S*,6a*R*,8*R*,9*S*,11a*R*,11b*S*)-methyl 8-hydroxy-9-(((4-methoxybenzyl)amino)methyl)-4,11b-dimethyltetradecahydro-6a,9-methanocyclohepta[a]naphthalene-4-carboxylate **(32)**

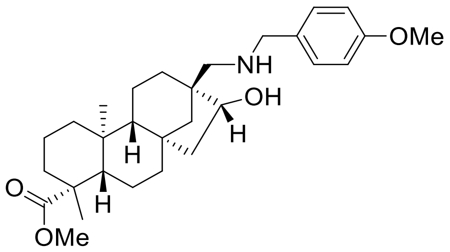

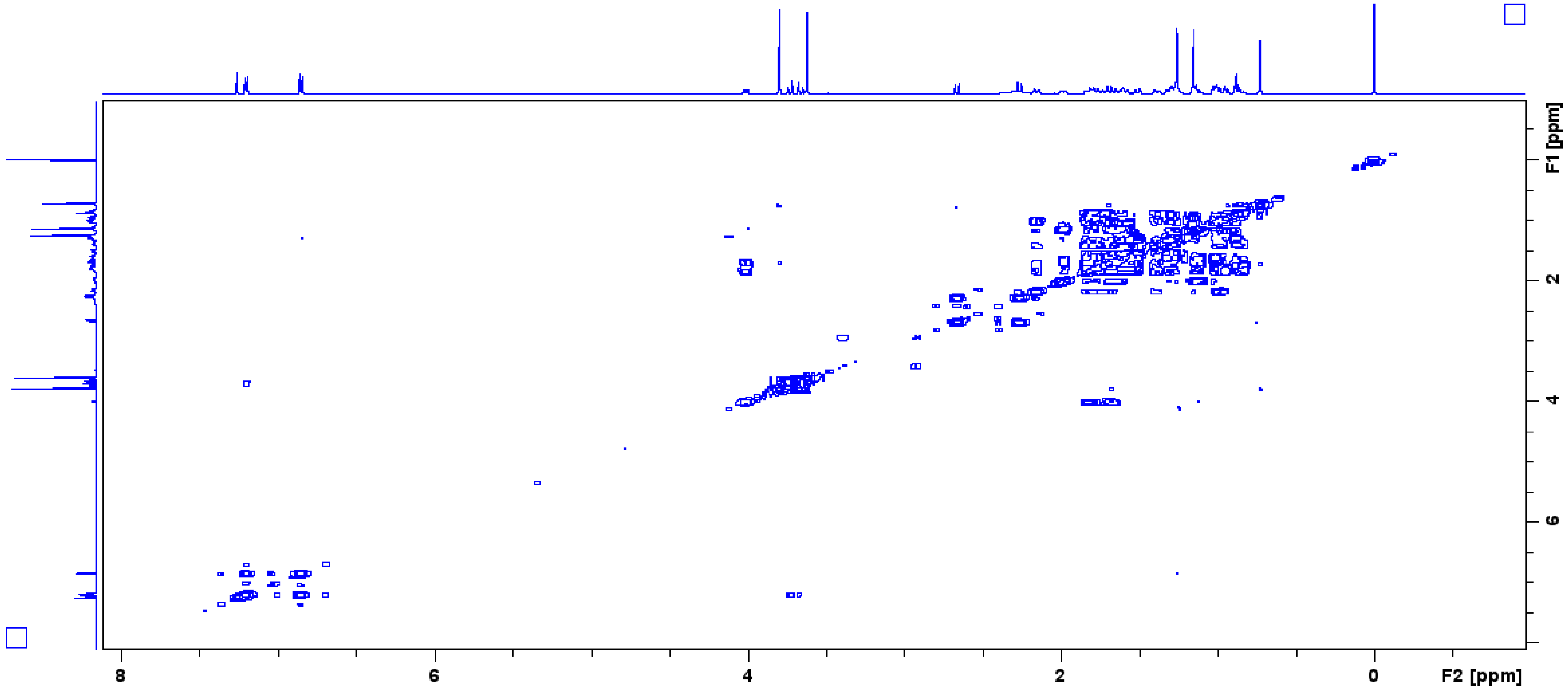

NOESY of compound (4*R*,4a*S*,6a*R*,8*R*,9*S*,11a*R*,11b*S*)-methyl 8-hydroxy-9-(((4-methoxybenzyl)amino)methyl)-4,11b-dimethyltetradecahydro-6a,9-methanocyclohepta[a]naphthalene-4-carboxylate **(32)**

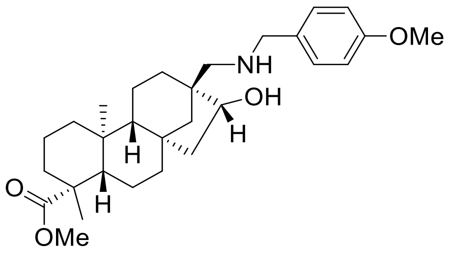

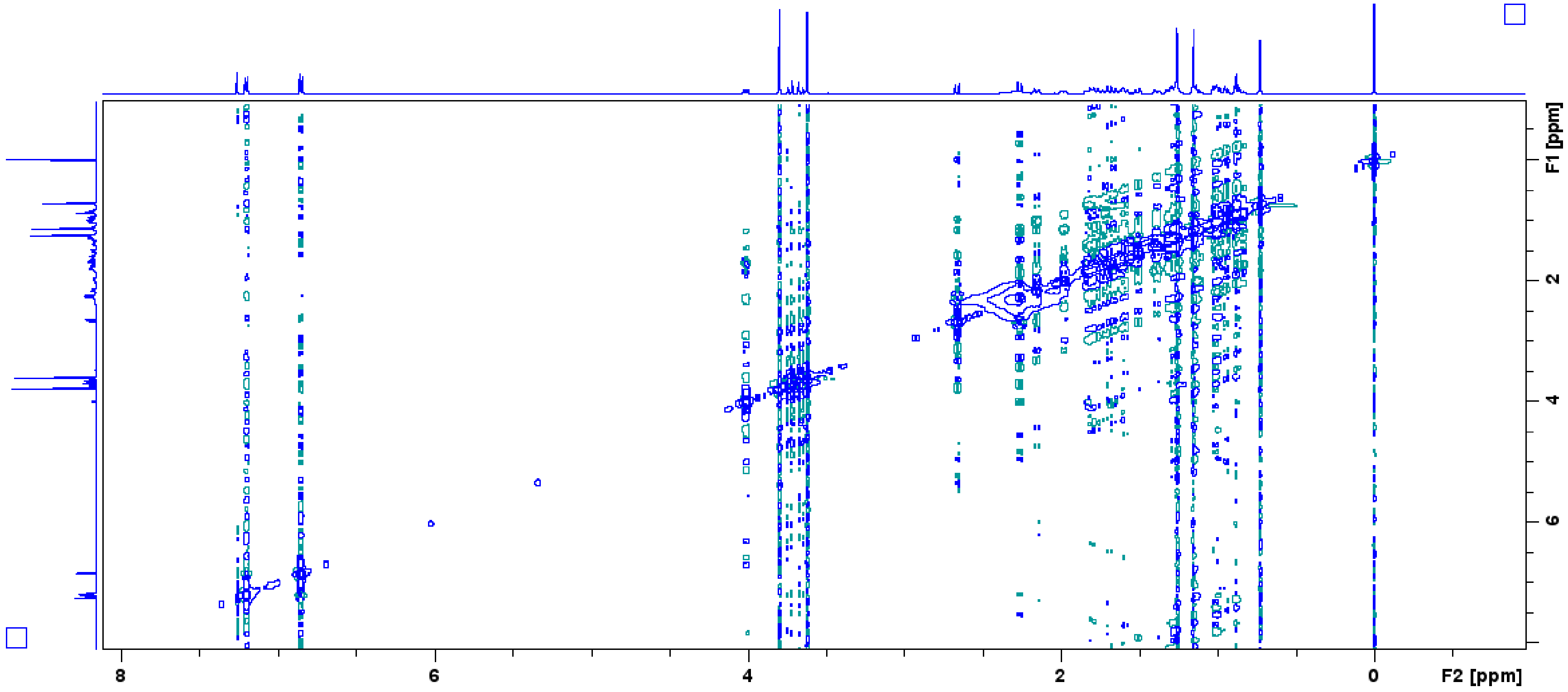

HSQC of compound (4*R*,4a*S*,6a*R*,8*R*,9*S*,11a*R*,11b*S*)-methyl 8-hydroxy-9-(((4-methoxybenzyl)amino)methyl)-4,11b-dimethyltetradecahydro-6a,9-methanocyclohepta[a]naphthalene-4-carboxylate **(32)**

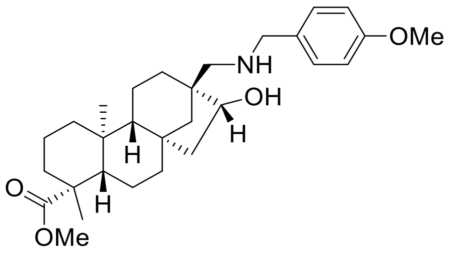

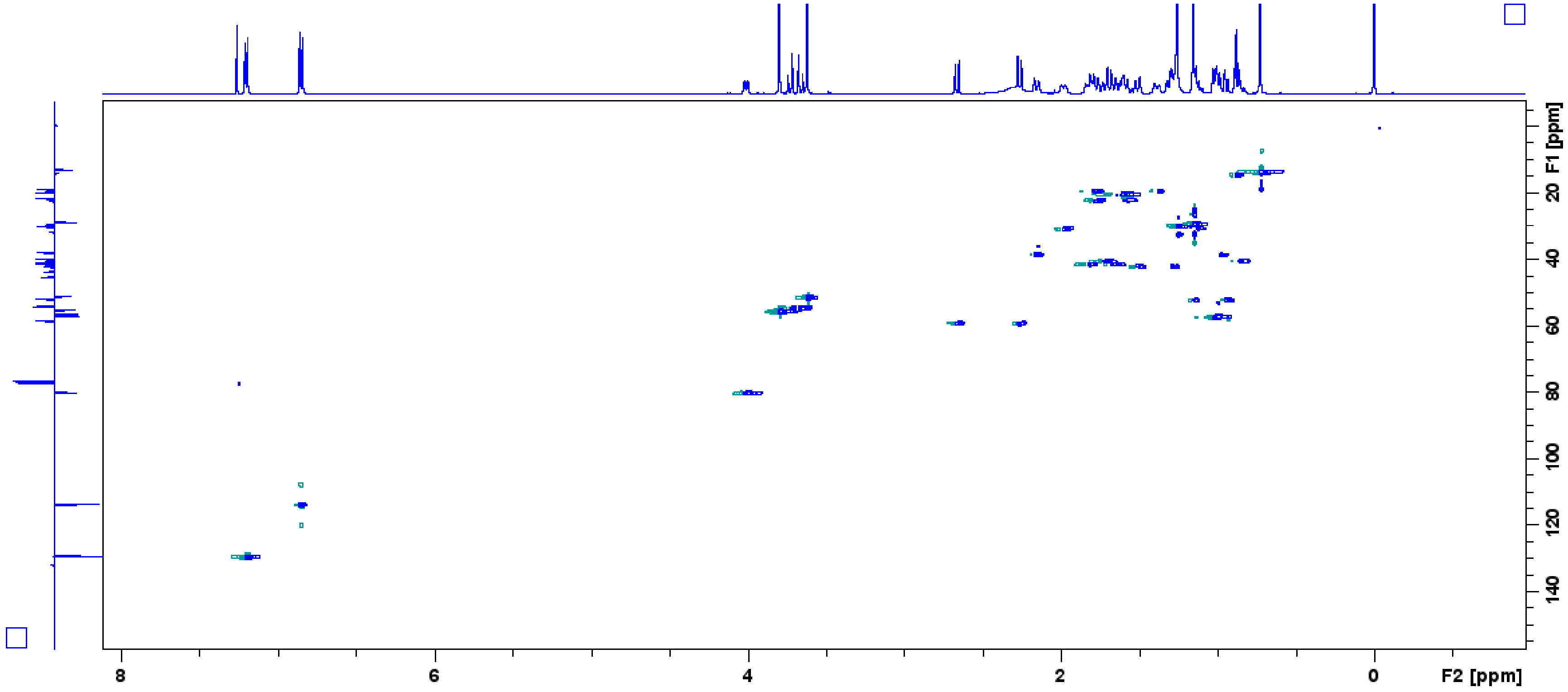

HMBC of compound (4*R*,4a*S*,6a*R*,8*R*,9*S*,11a*R*,11b*S*)-methyl 8-hydroxy-9-(((4-methoxybenzyl)amino)methyl)-4,11b-dimethyltetradecahydro-6a,9-methanocyclohepta[a]naphthalene-4-carboxylate **(32)**

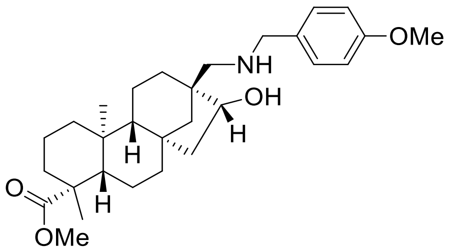

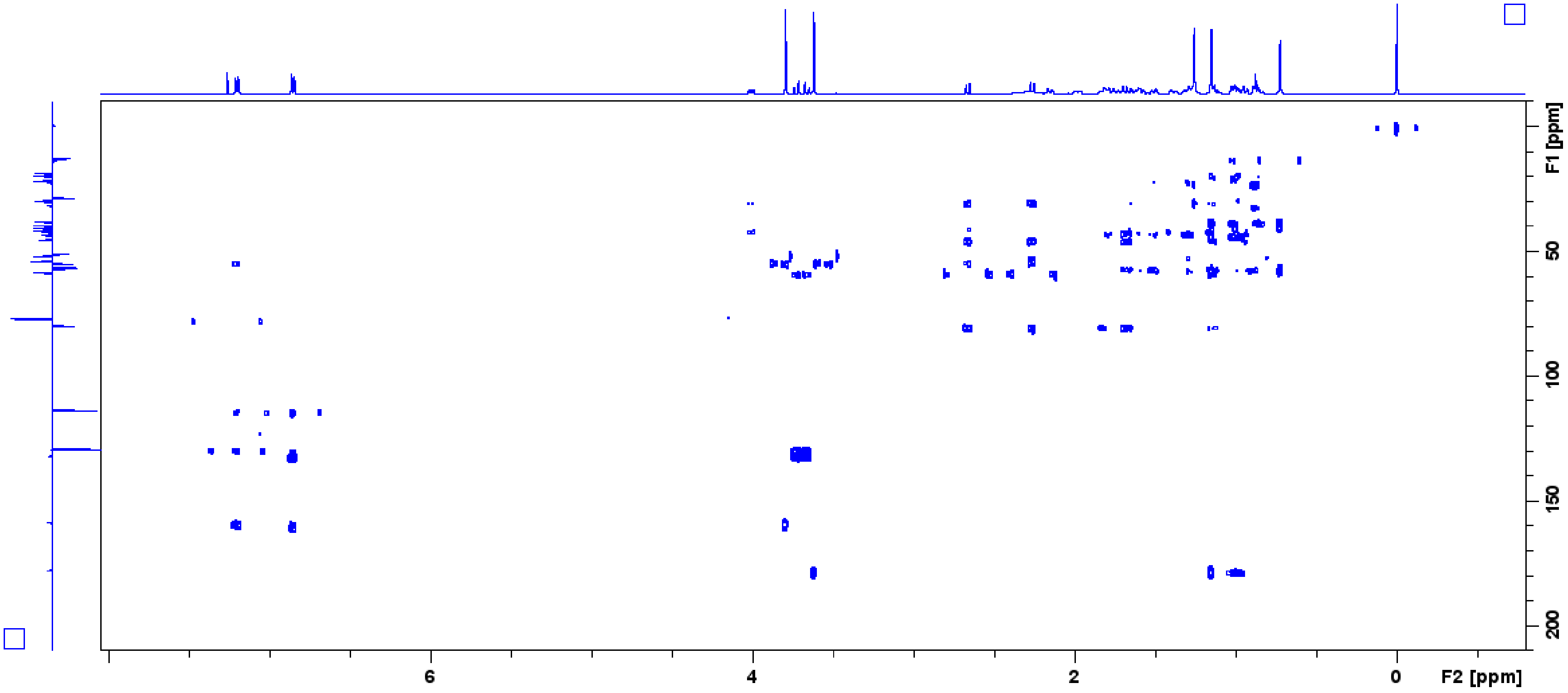

^{1}H-NMR of compound (4*R*,4a*S*,6a*R*,9*S*,11a*R*,11b*S*)-methyl 4,11b-dimethyl-8-oxo-9-((4-phenyl-1*H*-1,2,3-triazol-1-yl)methyl)tetradecahydro-6a,9-methanocyclohepta[a]naphthalene-4-carboxylate **(33)**

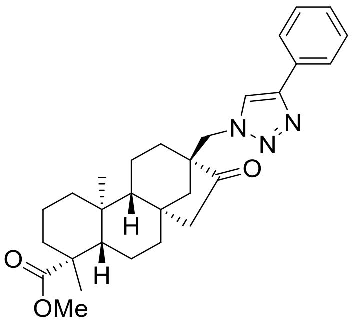

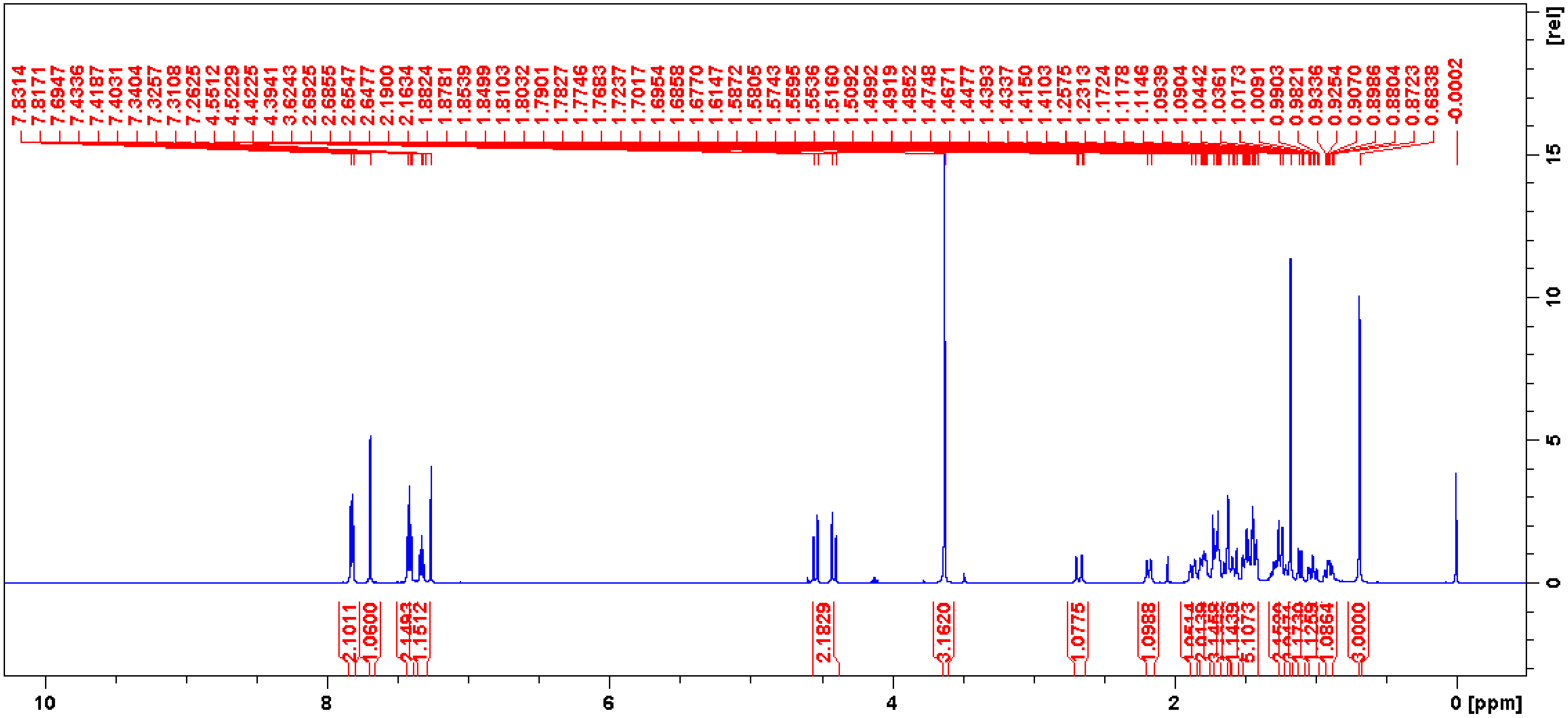

^{13}C-NMR of compound (4*R*,4a*S*,6a*R*,9*S*,11a*R*,11b*S*)-methyl 4,11b-dimethyl-8-oxo-9-((4-phenyl-1*H*-1,2,3-triazol-1-yl)methyl)tetradecahydro-6a,9-methanocyclohepta[a]naphthalene-4-carboxylate **(33)**

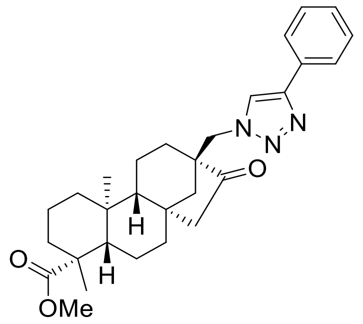

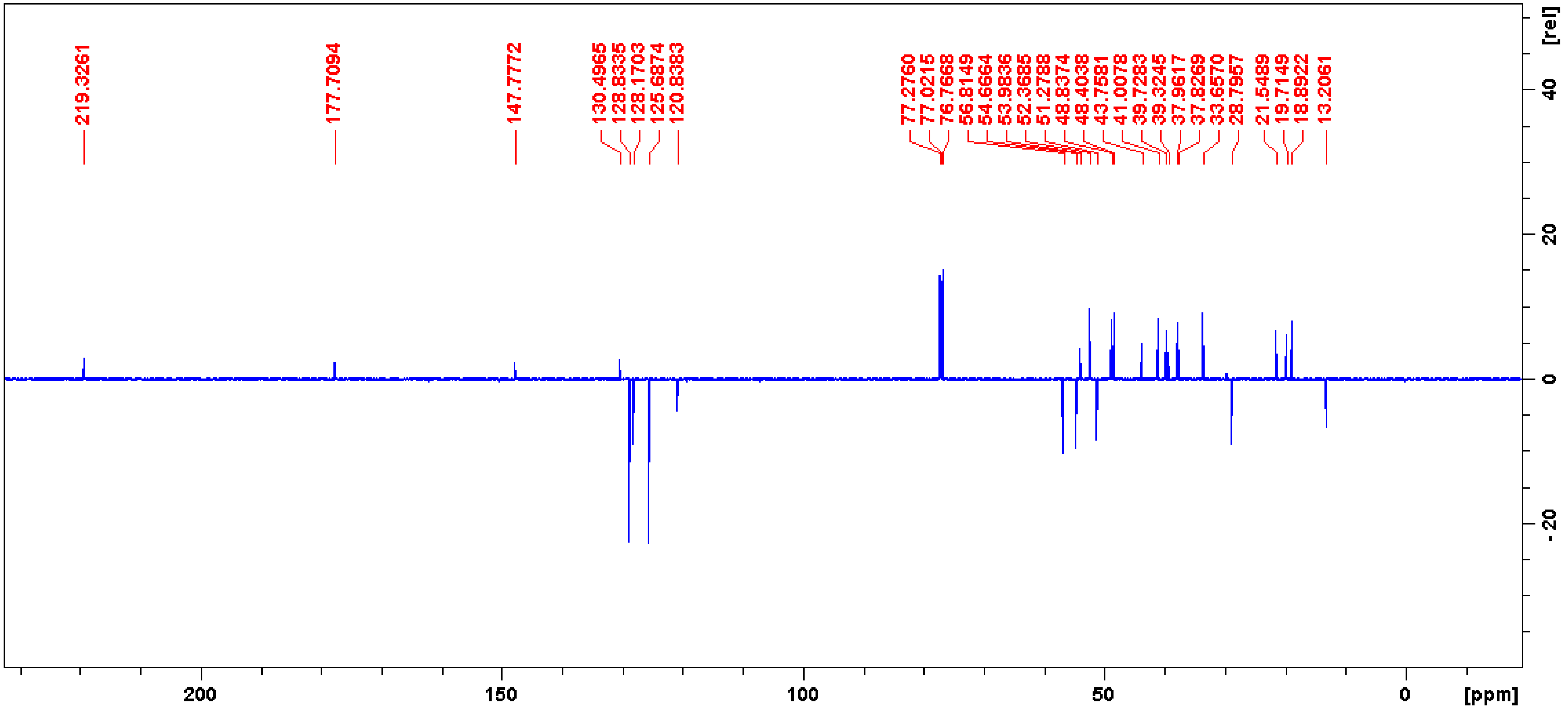

COSY of compound (4*R*,4a*S*,6a*R*,9*S*,11a*R*,11b*S*)-methyl 4,11b-dimethyl-8-oxo-9-((4-phenyl-1*H*-1,2,3-triazol-1-yl)methyl)tetradecahydro-6a,9-methanocyclohepta[a]naphthalene-4-carboxylate **(33)**

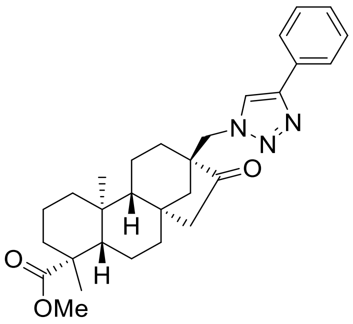

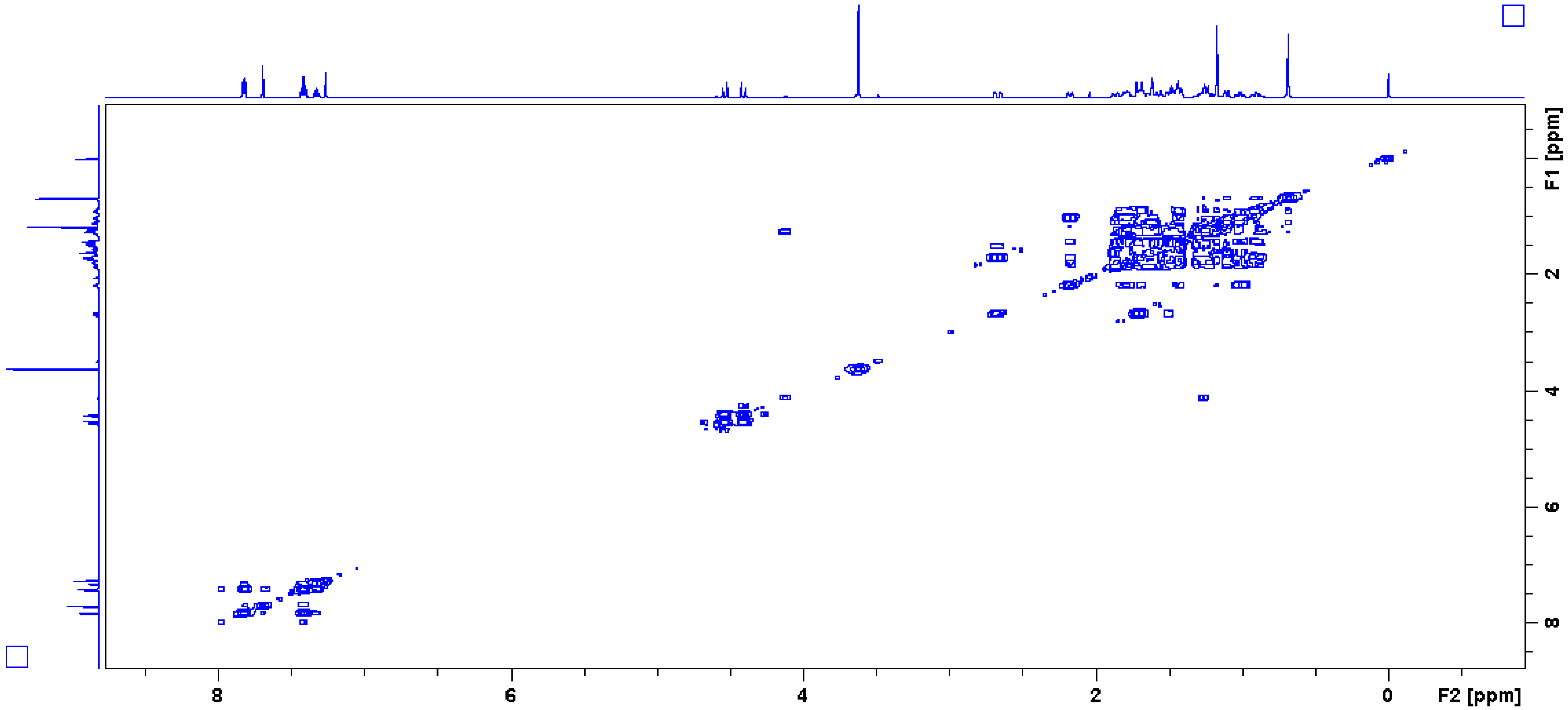

NOESY of compound (4*R*,4a*S*,6a*R*,9*S*,11a*R*,11b*S*)-methyl 4,11b-dimethyl-8-oxo-9-((4-phenyl-1*H*-1,2,3-triazol-1-yl)methyl)tetradecahydro-6a,9-methanocyclohepta[a]naphthalene-4-carboxylate **(33)**

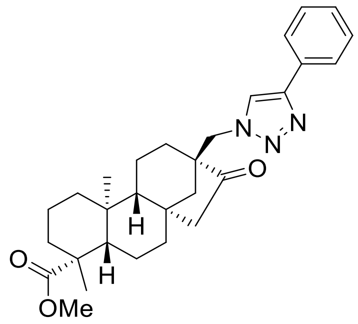

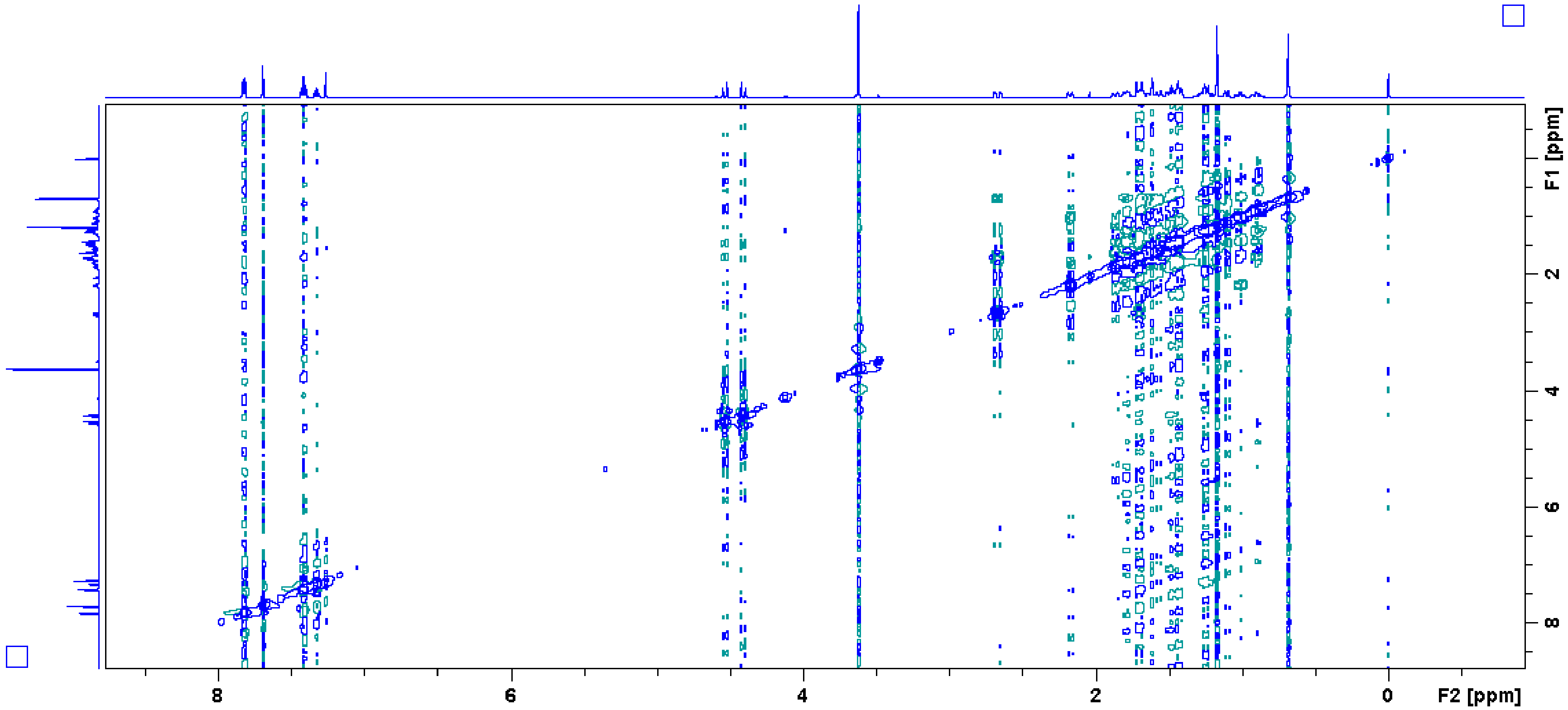

HSQC of compound (4*R*,4a*S*,6a*R*,9*S*,11a*R*,11b*S*)-methyl 4,11b-dimethyl-8-oxo-9-((4-phenyl-1*H*-1,2,3-triazol-1-yl)methyl)tetradecahydro-6a,9-methanocyclohepta[a]naphthalene-4-carboxylate **(33)**

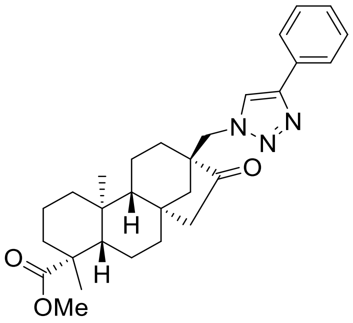

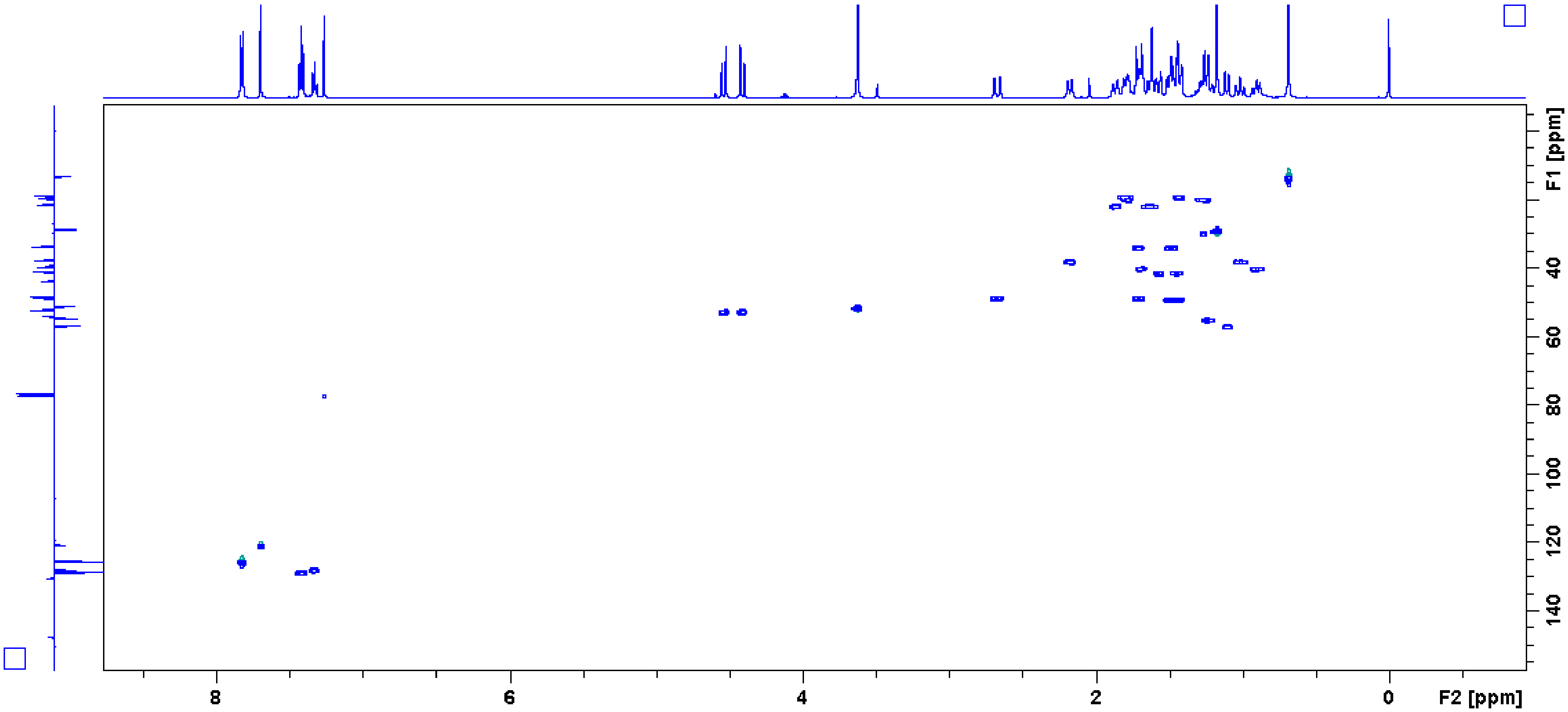

HMBC of compound (4*R*,4a*S*,6a*R*,9*S*,11a*R*,11b*S*)-methyl 4,11b-dimethyl-8-oxo-9-((4-phenyl-1*H*-1,2,3-triazol-1-yl)methyl)tetradecahydro-6a,9-methanocyclohepta[a]naphthalene-4-carboxylate **(33)**

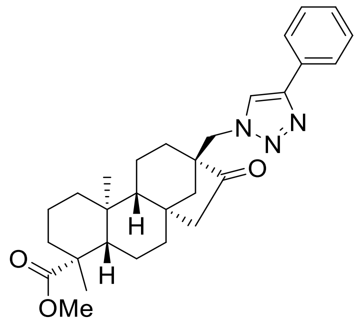

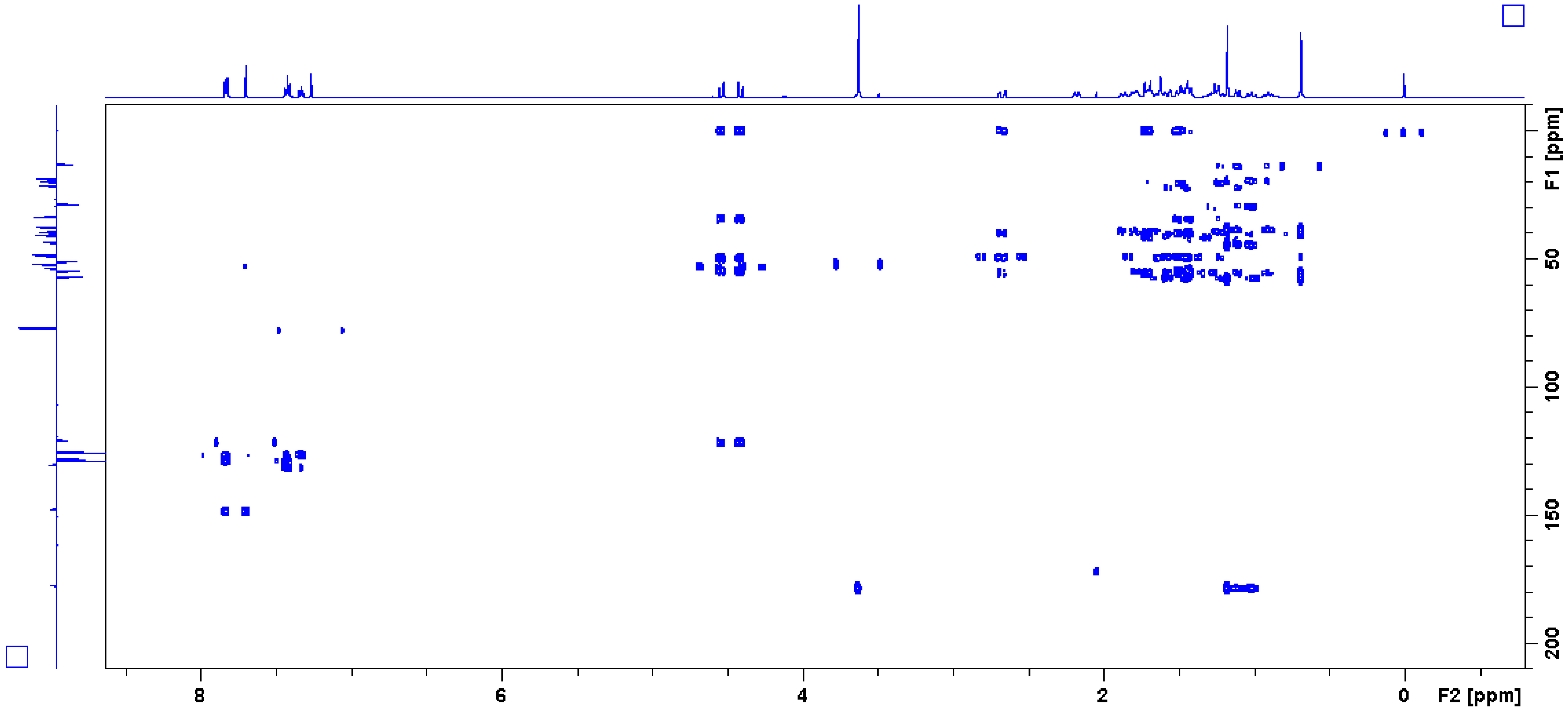

^{1}H-NMR of compound (4*R*,4a*S*,6a*R*,9*S*,11a*R*,11b*S*)-methyl 9-((4-benzyl-1*H*-1,2,3-triazol-1-yl)methyl)-4,11b-dimethyl-8-oxotetradecahydro-6a,9-methanocyclohepta[a]naphthalene-4-carboxylate **(34)**

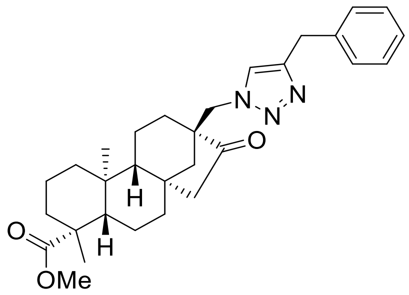

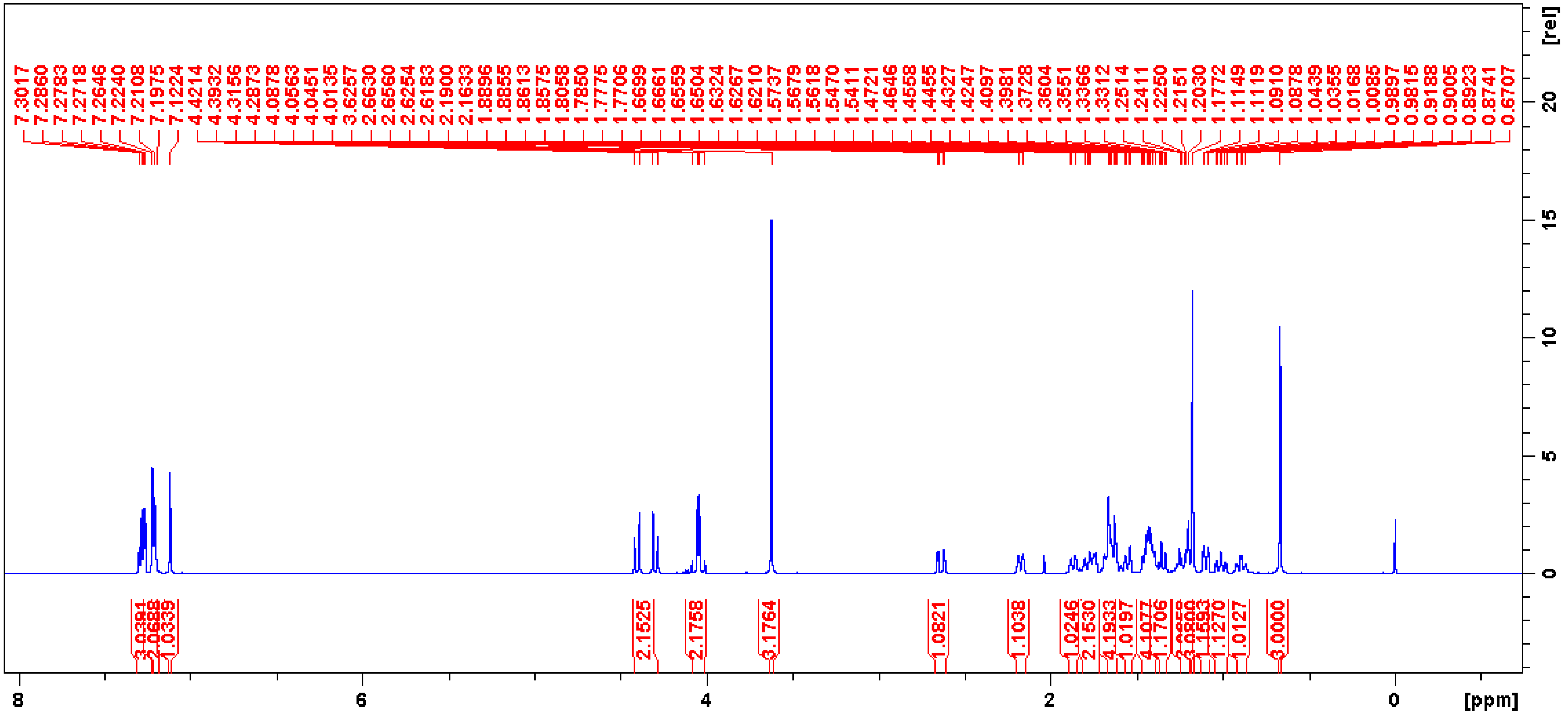

^{13}C-NMR of compound (4*R*,4a*S*,6a*R*,9*S*,11a*R*,11b*S*)-methyl 9-((4-benzyl-1*H*-1,2,3-triazol-1-yl)methyl)-4,11b-dimethyl-8-oxotetradecahydro-6a,9-methanocyclohepta[a]naphthalene-4-carboxylate **(34)**

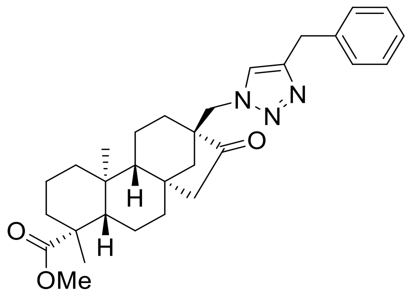

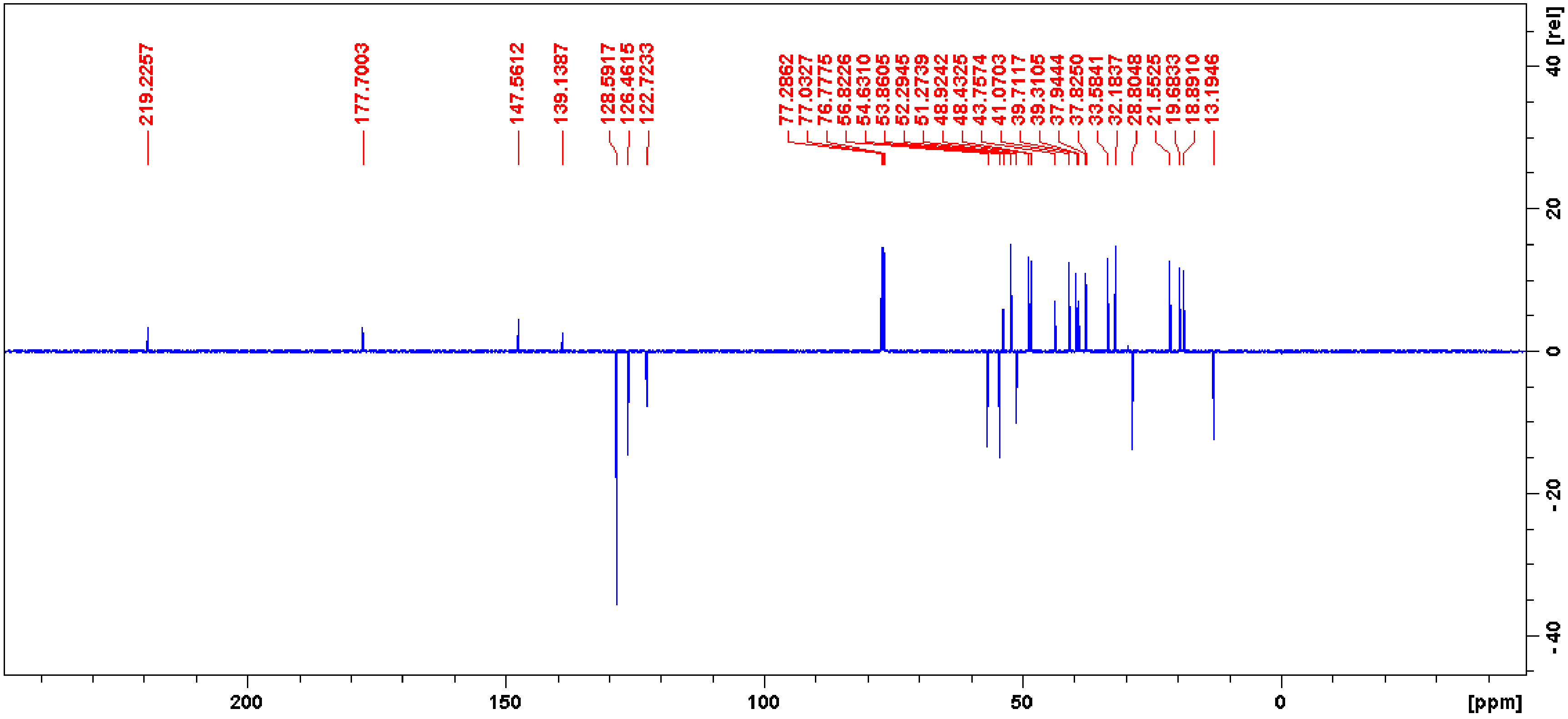

COSY of compound (4*R*,4a*S*,6a*R*,9*S*,11a*R*,11b*S*)-methyl 9-((4-benzyl-1*H*-1,2,3-triazol-1-yl)methyl)-4,11b-dimethyl-8-oxotetradecahydro-6a,9-methanocyclohepta[a]naphthalene-4-carboxylate **(34)**

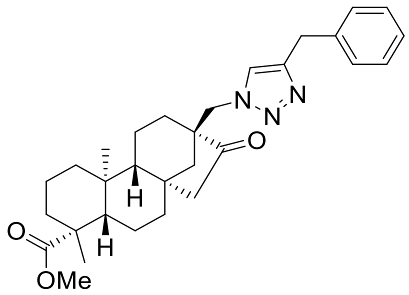

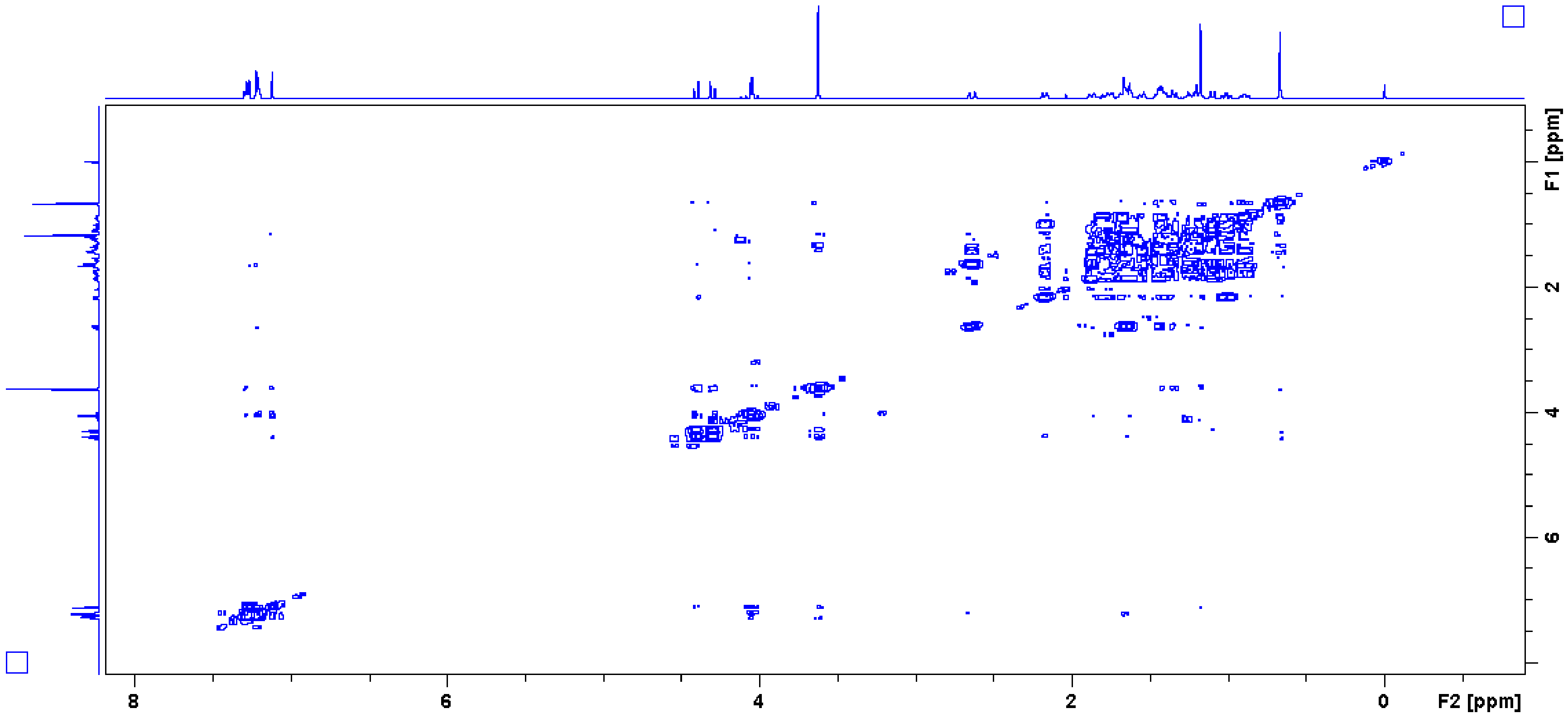

NOESY of compound (4*R*,4a*S*,6a*R*,9*S*,11a*R*,11b*S*)-methyl 9-((4-benzyl-1*H*-1,2,3-triazol-1-yl)methyl)-4,11b-dimethyl-8-oxotetradecahydro-6a,9-methanocyclohepta[a]naphthalene-4-carboxylate **(34)**

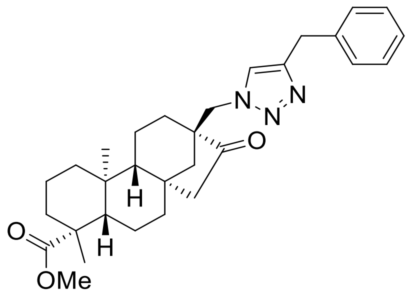

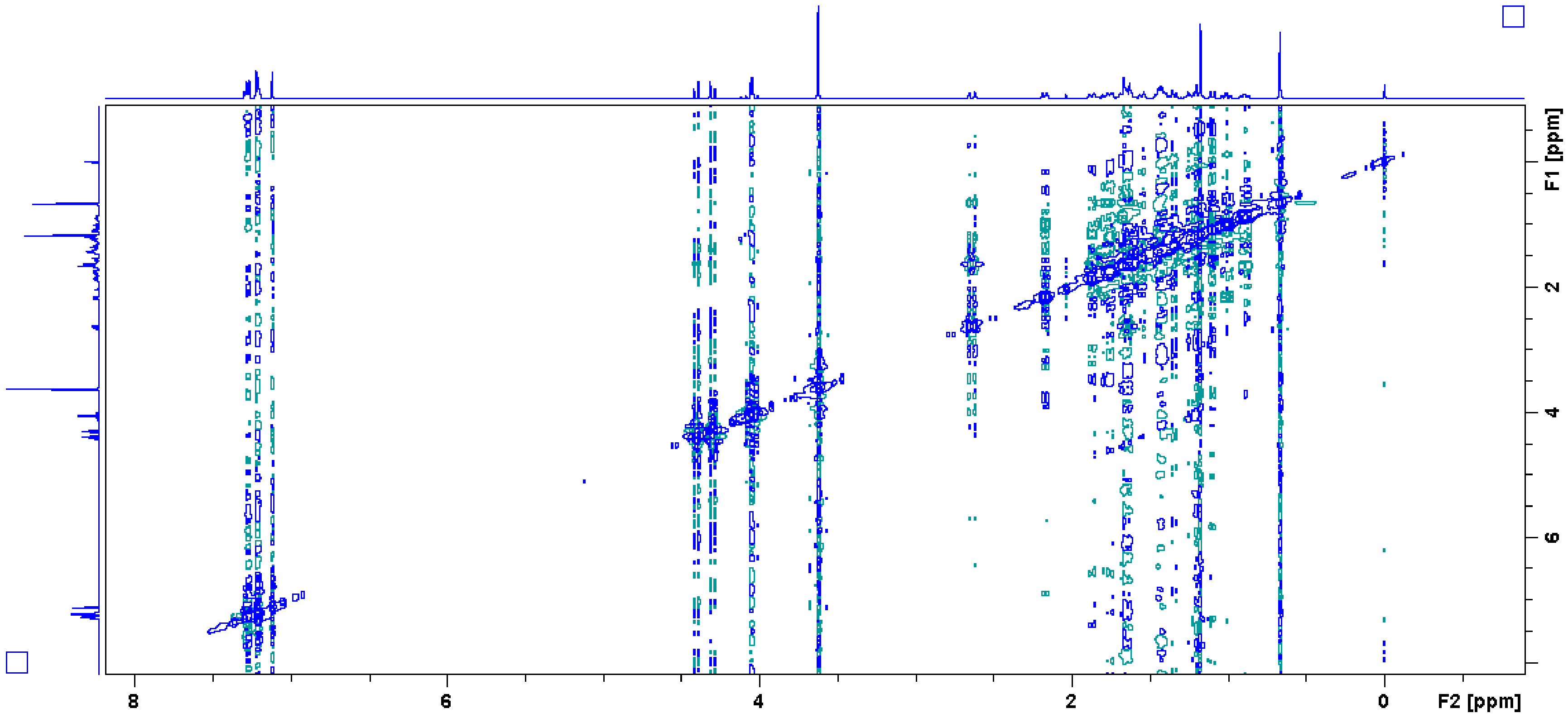

HSQC of compound (4*R*,4a*S*,6a*R*,9*S*,11a*R*,11b*S*)-methyl 9-((4-benzyl-1*H*-1,2,3-triazol-1-yl)methyl)-4,11b-dimethyl-8-oxotetradecahydro-6a,9-methanocyclohepta[a]naphthalene-4-carboxylate **(34)**

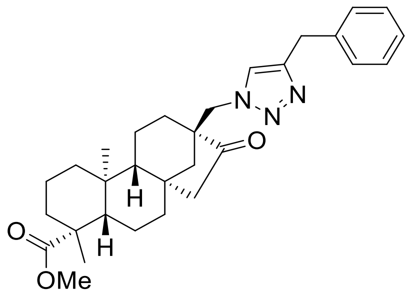

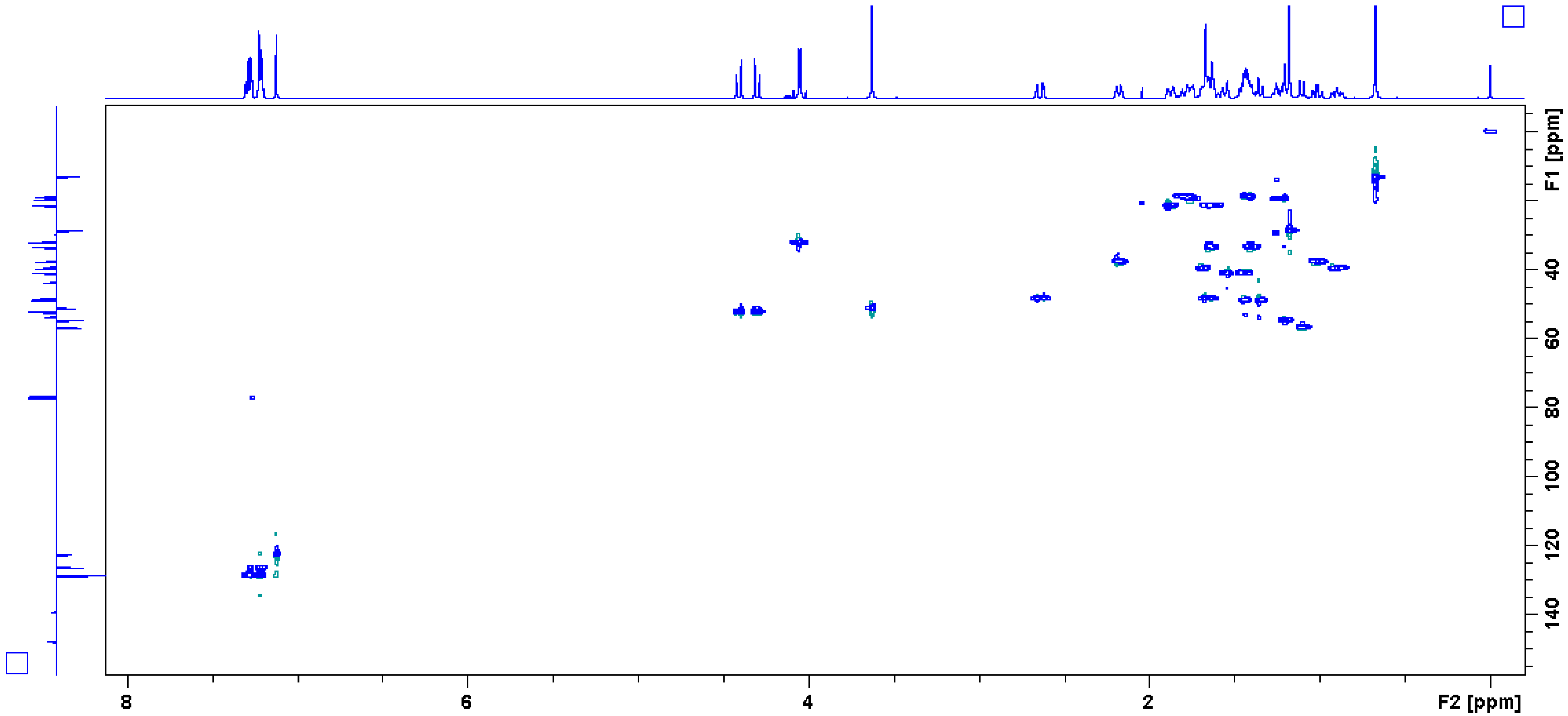

HMBC of compound (4*R*,4a*S*,6a*R*,9*S*,11a*R*,11b*S*)-methyl 9-((4-benzyl-1*H*-1,2,3-triazol-1-yl)methyl)-4,11b-dimethyl-8-oxotetradecahydro-6a,9-methanocyclohepta[a]naphthalene-4-carboxylate **(34)**

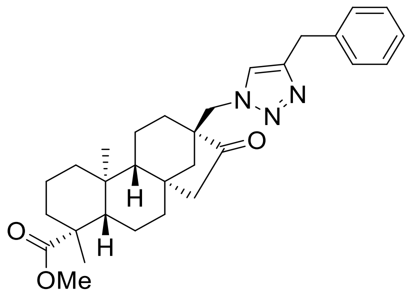

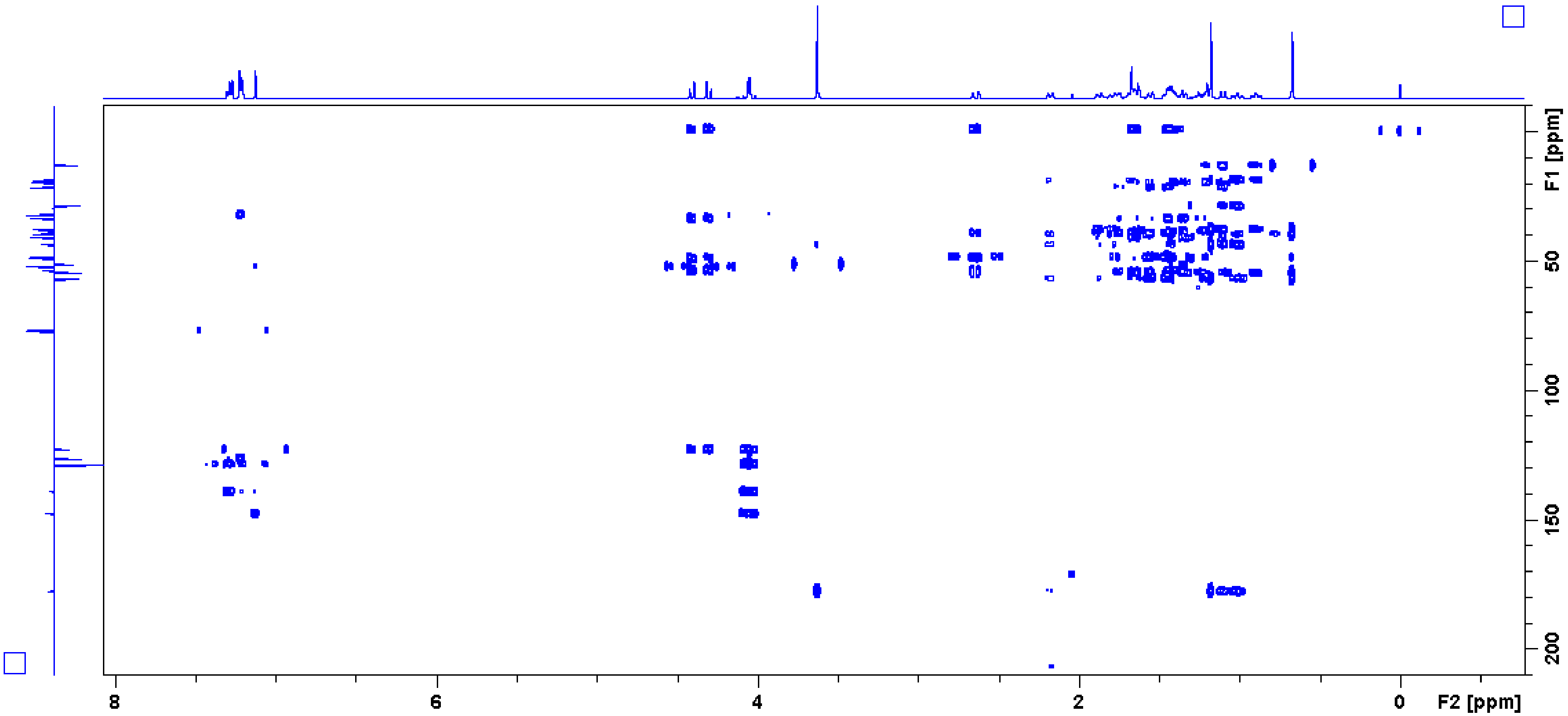

^{1}H-NMR of compound (4*R*,4a*S*,6a*R*,8*R*,9*S*,11a*R*,11b*S*)-methyl 8-hydroxy-4,11b-dimethyl-9-((4-phenyl-1*H*-1,2,3-triazol-1-yl)methyl)tetradecahydro-6a,9-methanocyclohepta[a]naphthalene-4-carboxylate **(35)**

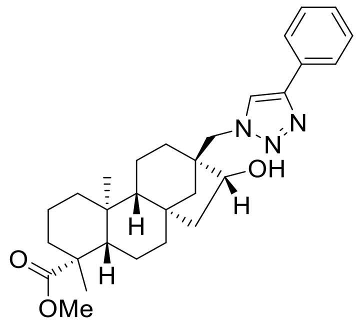

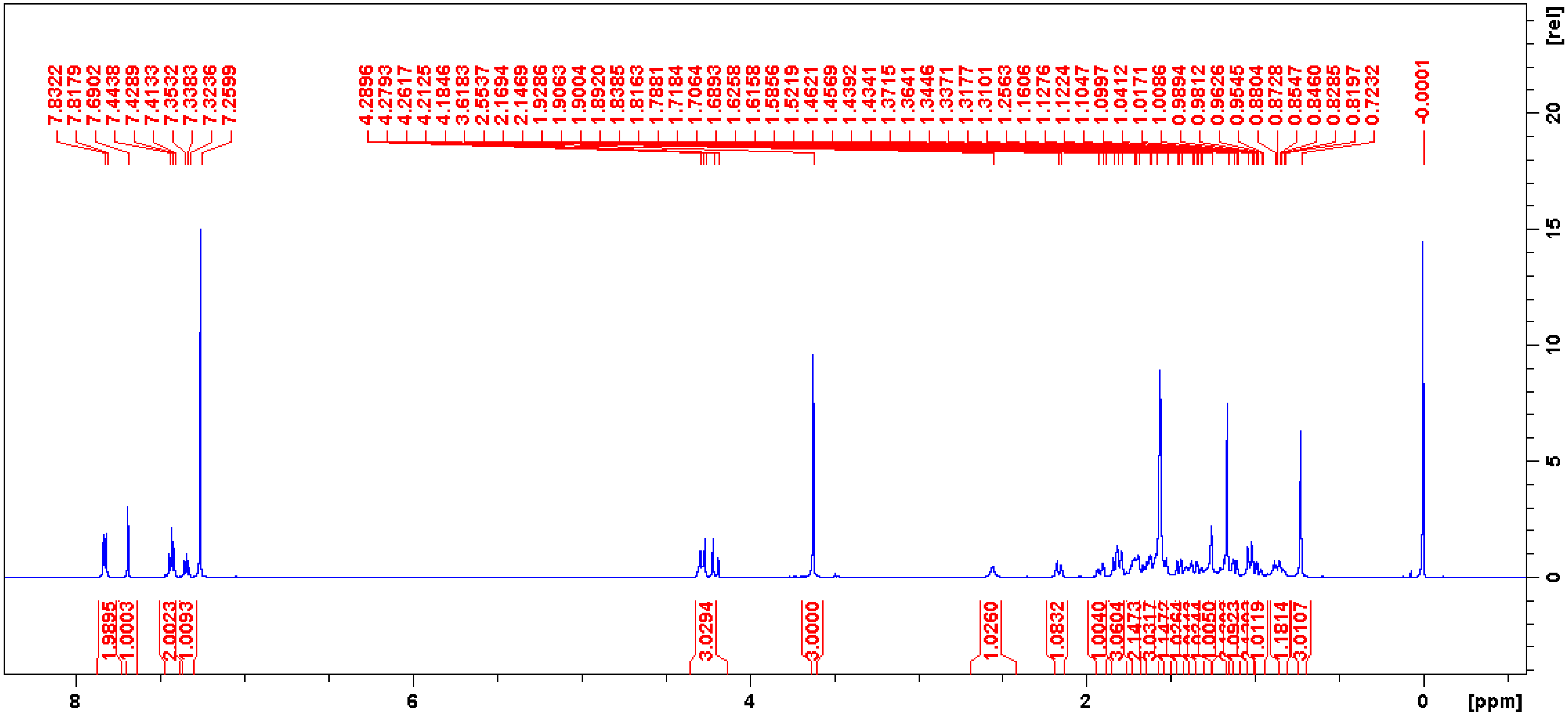

^{13}C-NMR of compound (4*R*,4a*S*,6a*R*,8*R*,9*S*,11a*R*,11b*S*)-methyl 8-hydroxy-4,11b-dimethyl-9-((4-phenyl-1*H*-1,2,3-triazol-1-yl)methyl)tetradecahydro-6a,9-methanocyclohepta[a]naphthalene-4-carboxylate **(35)**

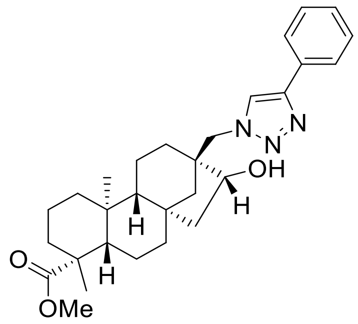

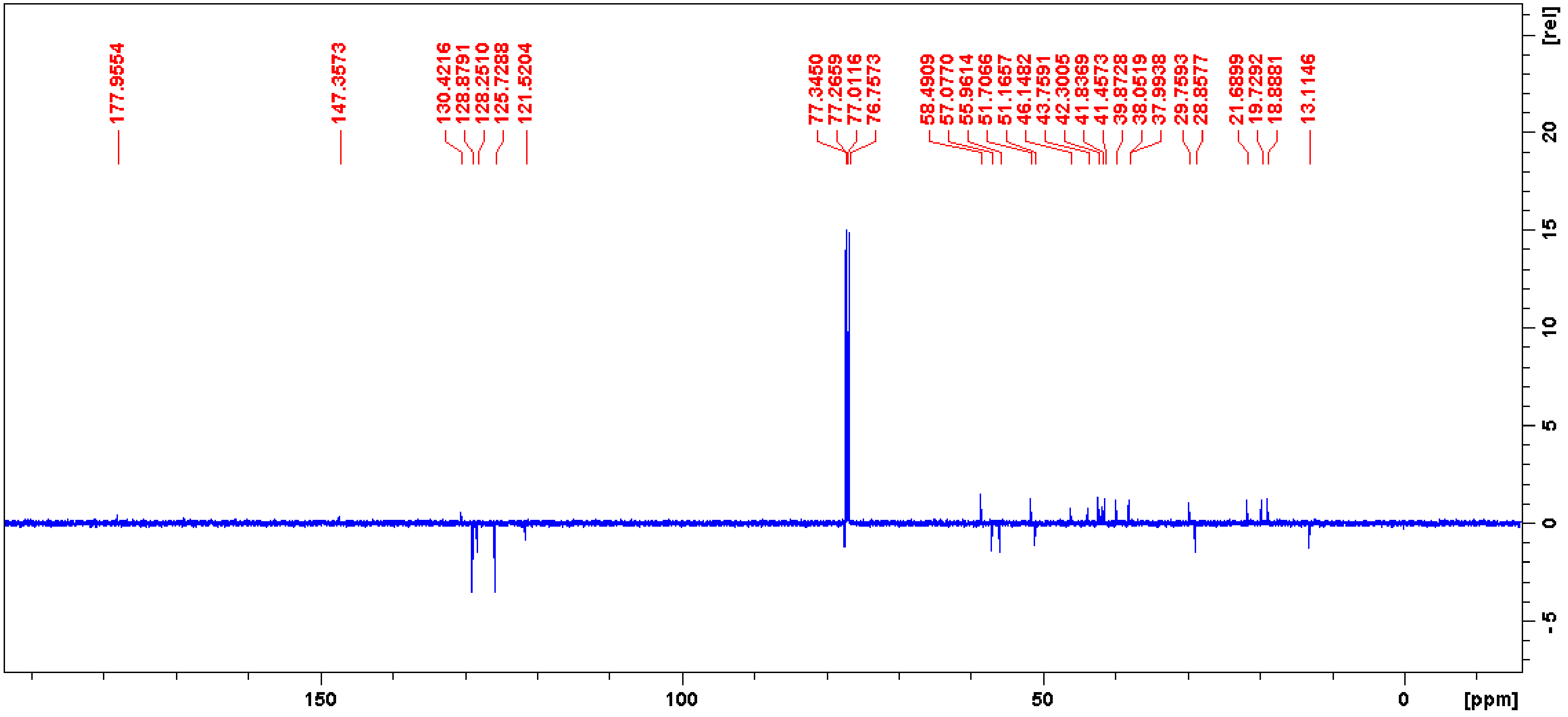

COSY of compound (4*R*,4a*S*,6a*R*,8*R*,9*S*,11a*R*,11b*S*)-methyl 8-hydroxy-4,11b-dimethyl-9-((4-phenyl-1*H*-1,2,3-triazol-1-yl)methyl)tetradecahydro-6a,9-methanocyclohepta[a]naphthalene-4-carboxylate **(35)**

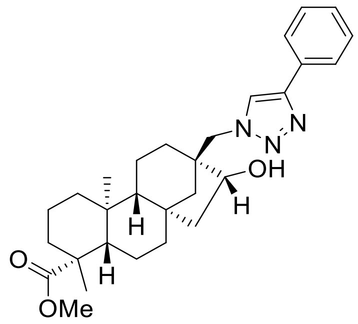

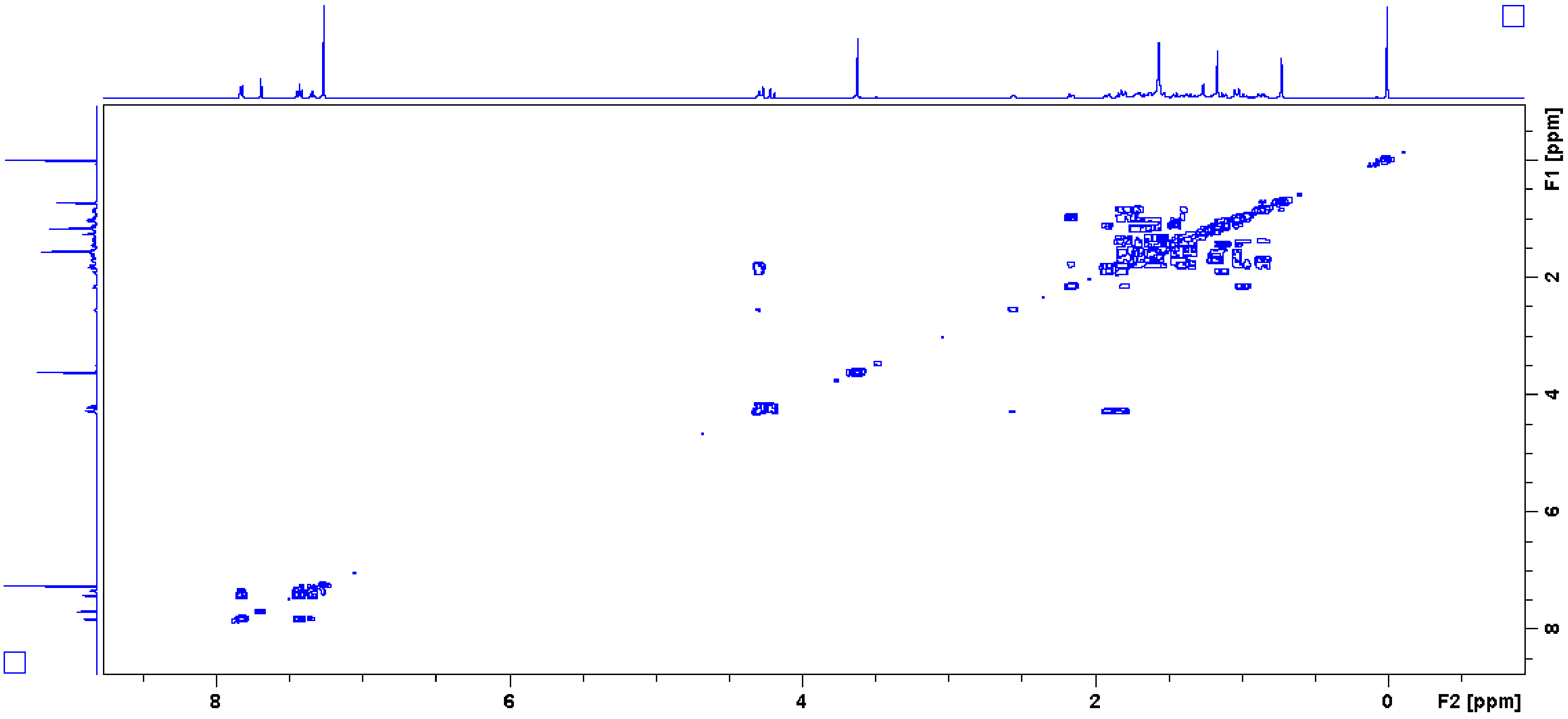

NOESY of compound (4*R*,4a*S*,6a*R*,8*R*,9*S*,11a*R*,11b*S*)-methyl 8-hydroxy-4,11b-dimethyl-9-((4-phenyl-1*H*-1,2,3-triazol-1-yl)methyl)tetradecahydro-6a,9-methanocyclohepta[a]naphthalene-4-carboxylate **(35)**

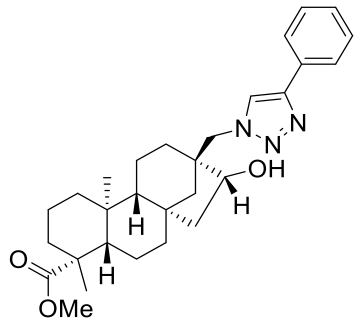

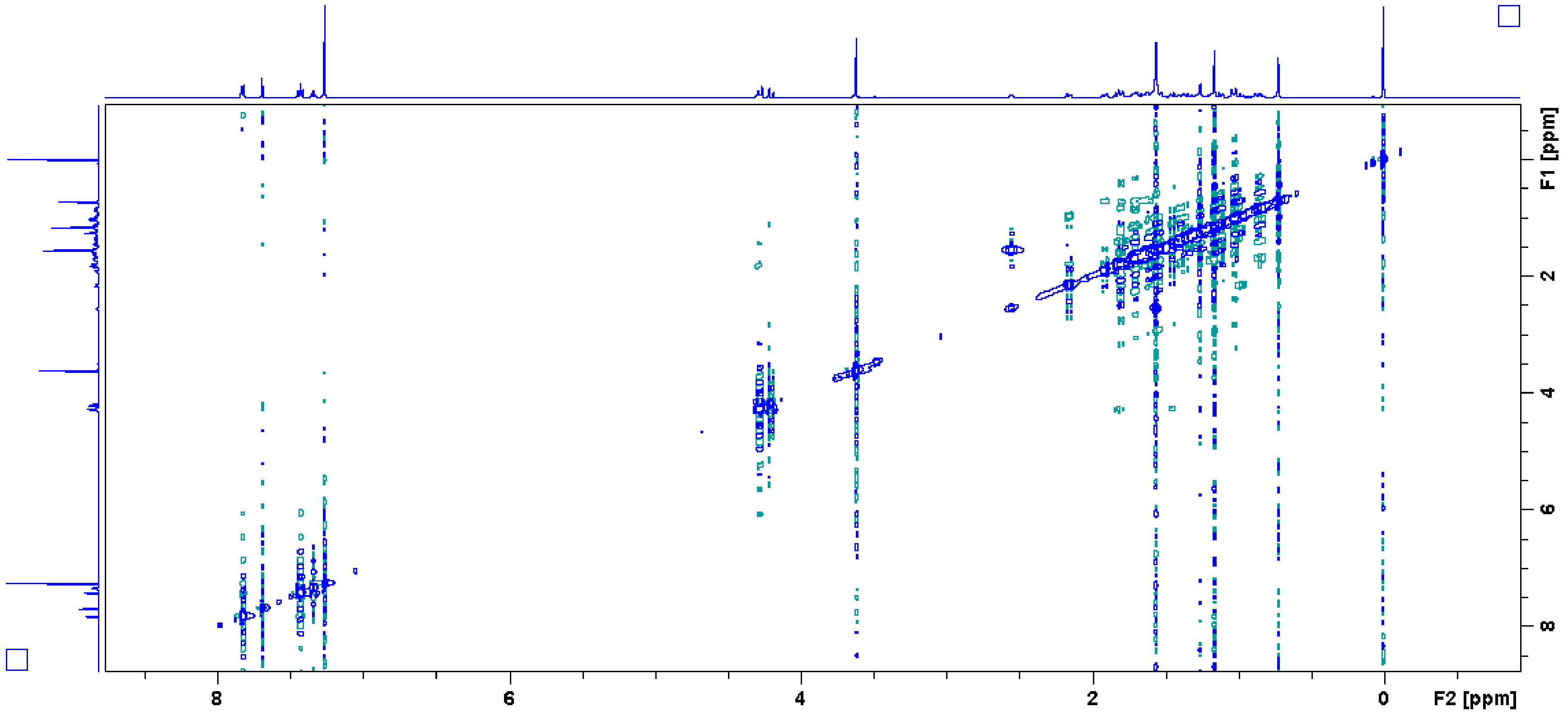

HSQC of compound (4*R*,4a*S*,6a*R*,8*R*,9*S*,11a*R*,11b*S*)-methyl 8-hydroxy-4,11b-dimethyl-9-((4-phenyl-1*H*-1,2,3-triazol-1-yl)methyl)tetradecahydro-6a,9-methanocyclohepta[a]naphthalene-4-carboxylate **(35)**

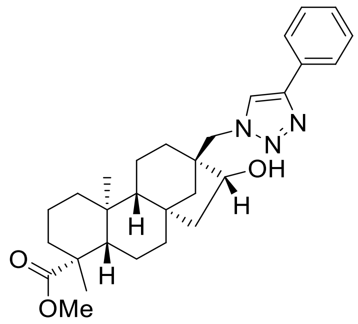

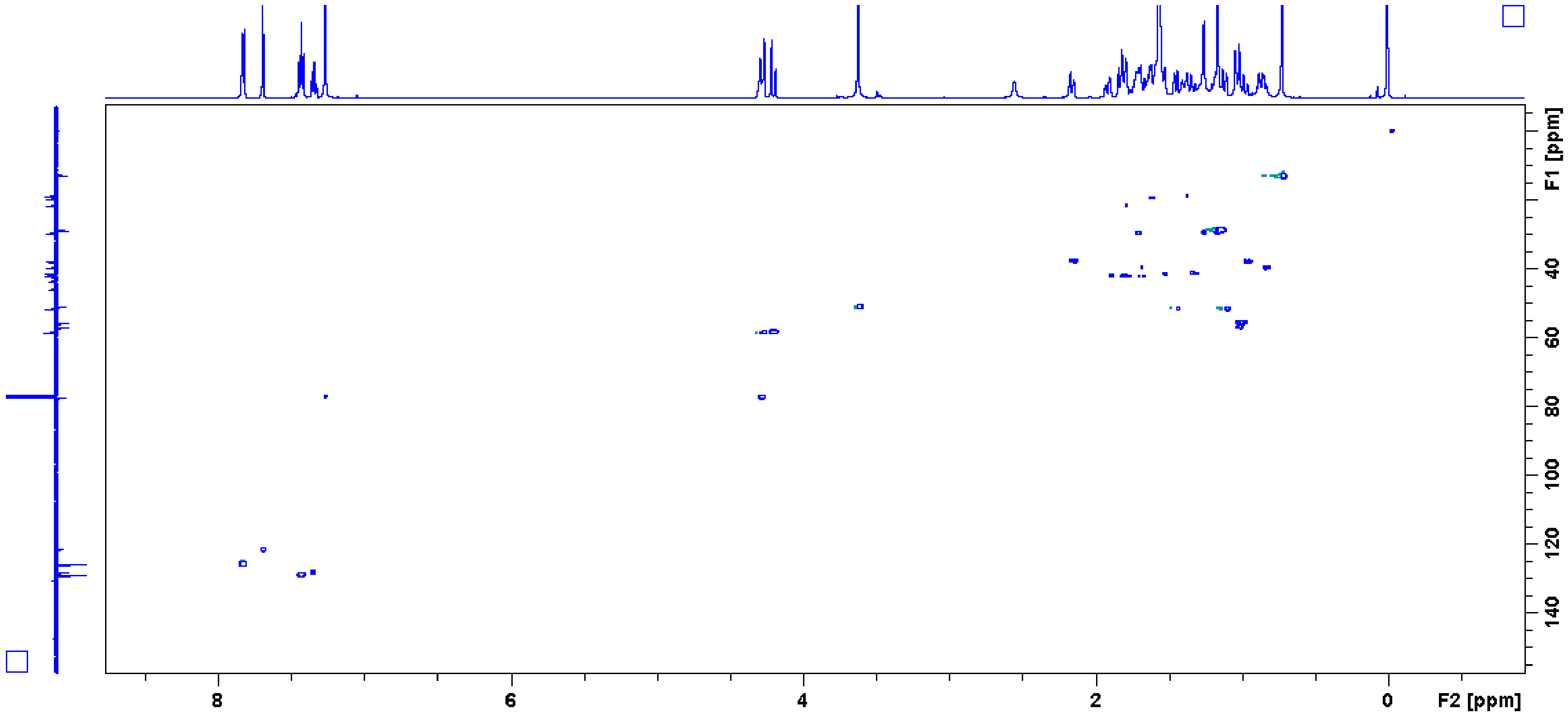

HMBC of compound (4*R*,4a*S*,6a*R*,8*R*,9*S*,11a*R*,11b*S*)-methyl 8-hydroxy-4,11b-dimethyl-9-((4-phenyl-1*H*-1,2,3-triazol-1-yl)methyl)tetradecahydro-6a,9-methanocyclohepta[a]naphthalene-4-carboxylate **(35)**

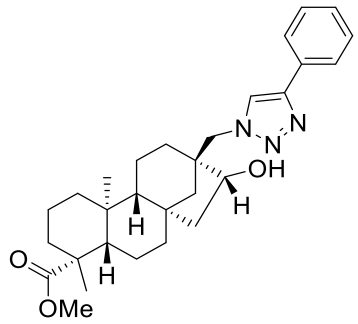

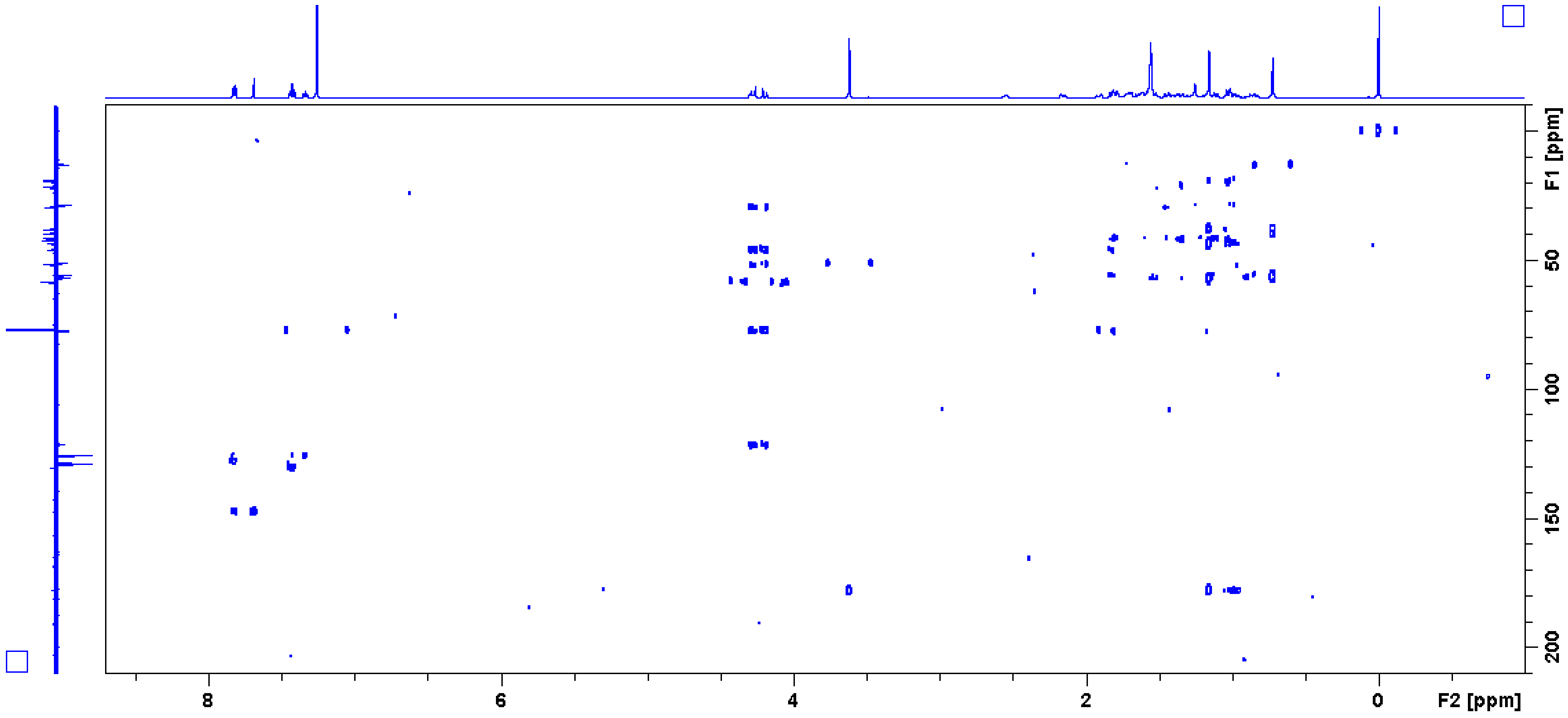

^{1}H-NMR of compound (4*R*,4a*S*,6a*R*,8*R*,9*S*,11a*R*,11b*S*)-methyl 9-((4-benzyl-1*H*-1,2,3-triazol-1-yl)methyl)-8-hydroxy-4,11b-dimethyltetradecahydro-6a,9-methanocyclohepta[a]naphthalene-4-carboxylate **(36)**

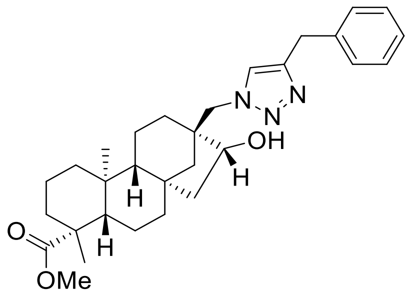

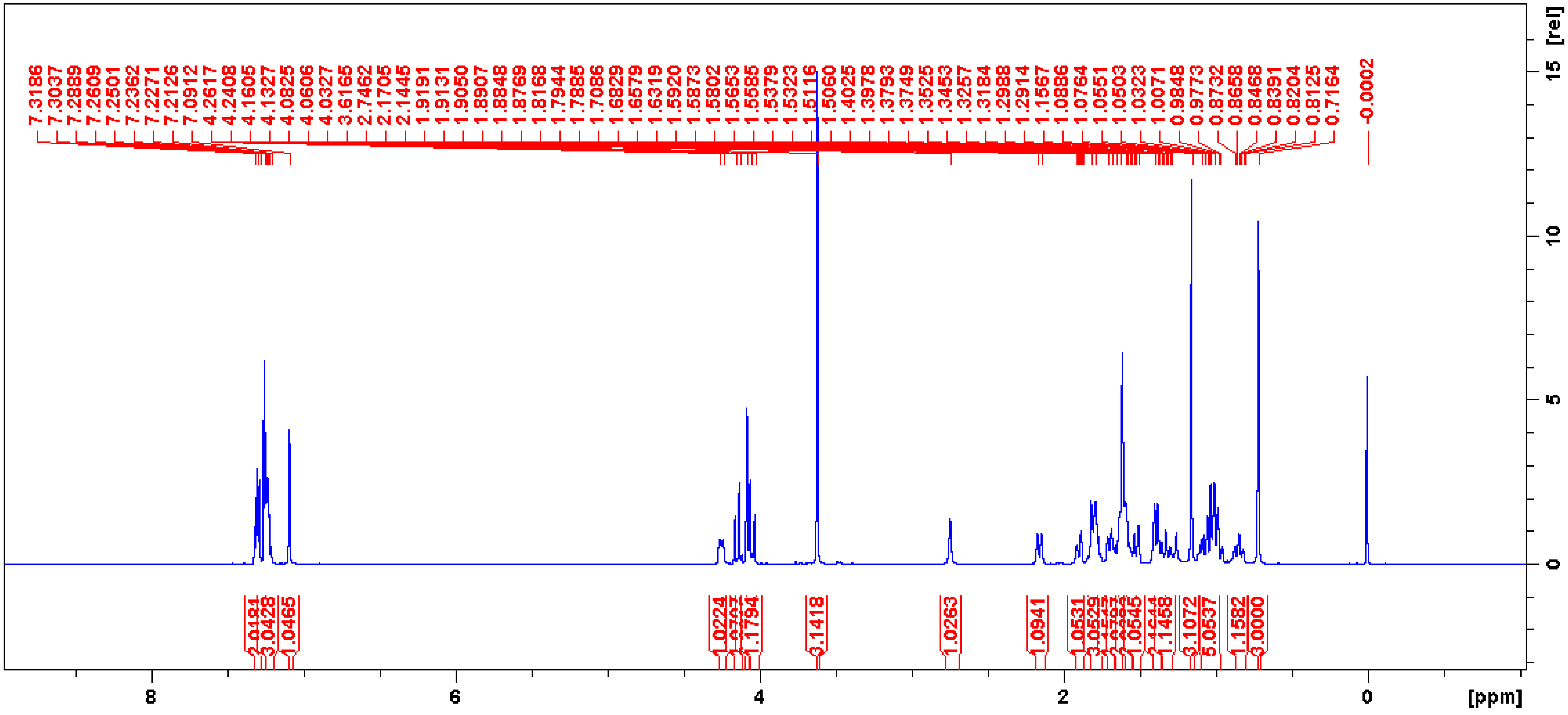

^{13}C-NMR of compound (4*R*,4a*S*,6a*R*,8*R*,9*S*,11a*R*,11b*S*)-methyl 9-((4-benzyl-1*H*-1,2,3-triazol-1-yl)methyl)-8-hydroxy-4,11b-dimethyltetradecahydro-6a,9-methanocyclohepta[a]naphthalene-4-carboxylate **(36)**

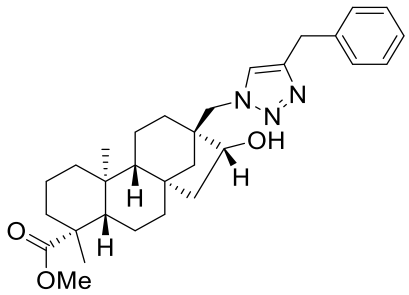

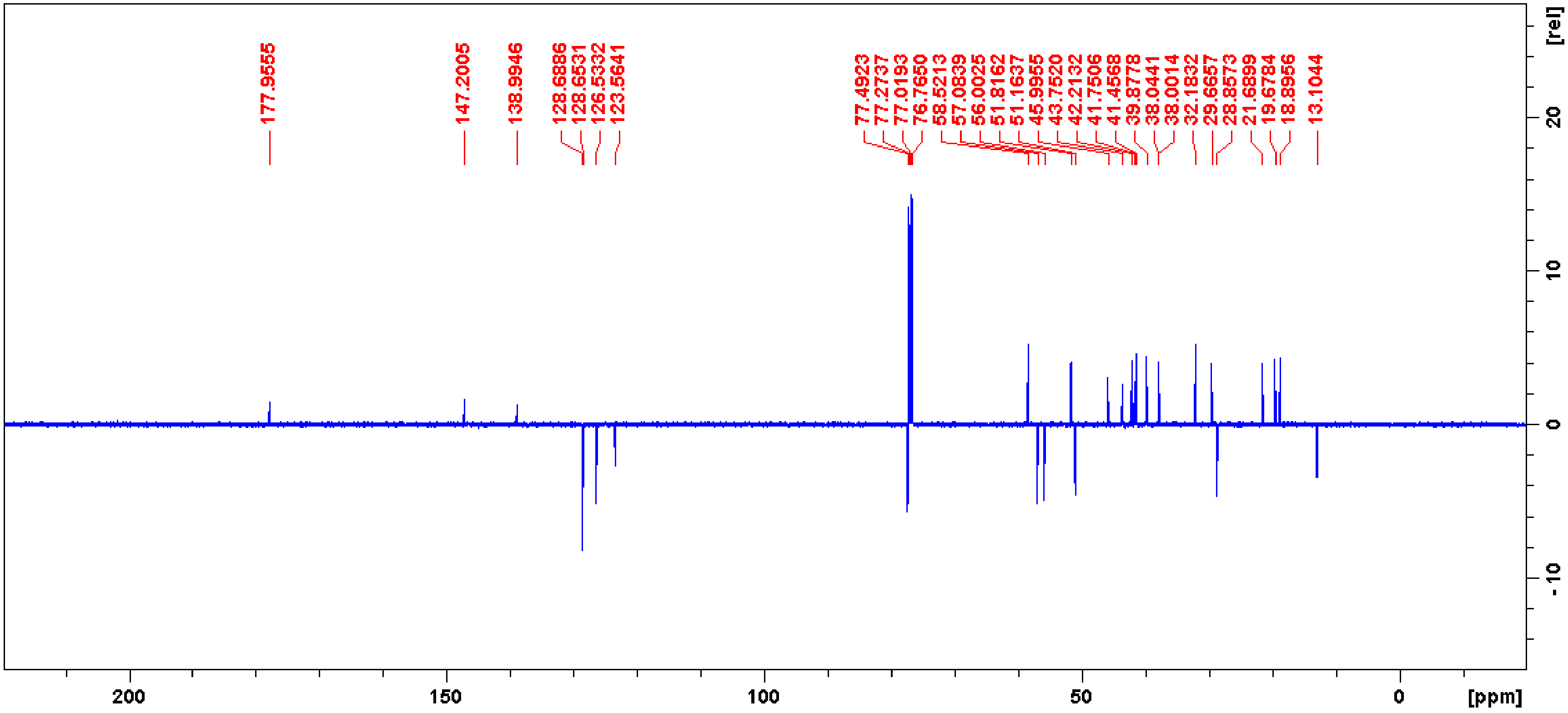

COSY of compound (4*R*,4a*S*,6a*R*,8*R*,9*S*,11a*R*,11b*S*)-methyl 9-((4-benzyl-1*H*-1,2,3-triazol-1-yl)methyl)-8-hydroxy-4,11b-dimethyltetradecahydro-6a,9-methanocyclohepta[a]naphthalene-4-carboxylate **(36)**

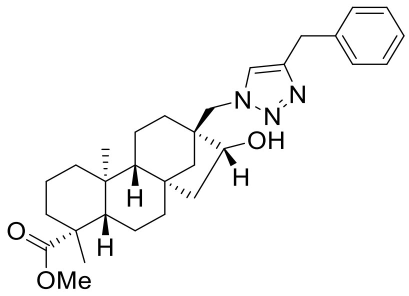

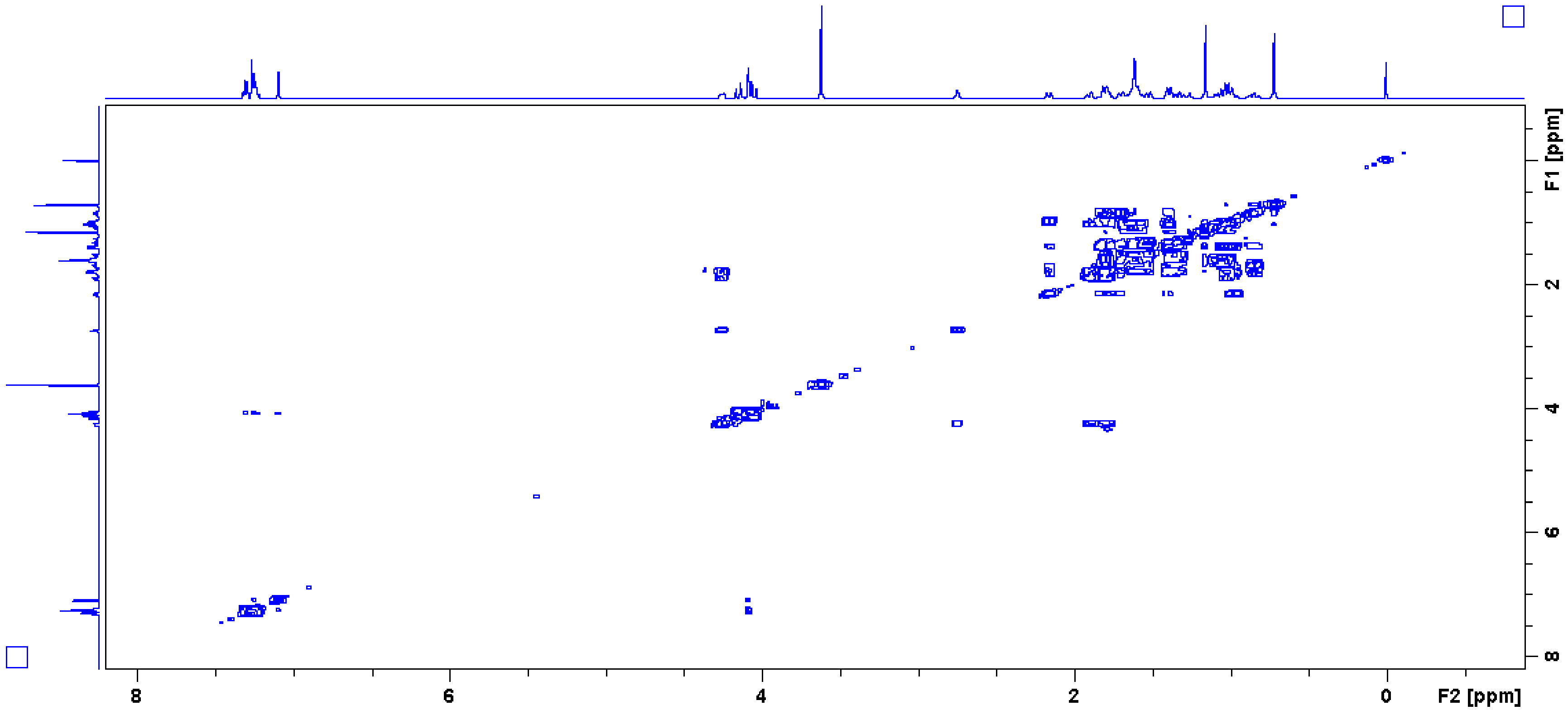

NOESY of compound (4*R*,4a*S*,6a*R*,8*R*,9*S*,11a*R*,11b*S*)-methyl 9-((4-benzyl-1*H*-1,2,3-triazol-1-yl)methyl)-8-hydroxy-4,11b-dimethyltetradecahydro-6a,9-methanocyclohepta[a]naphthalene-4-carboxylate **(36)**

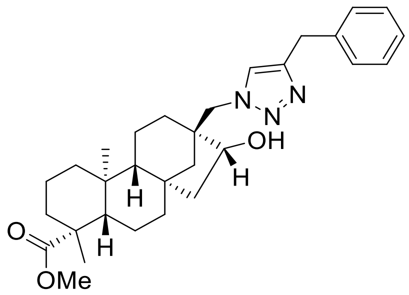

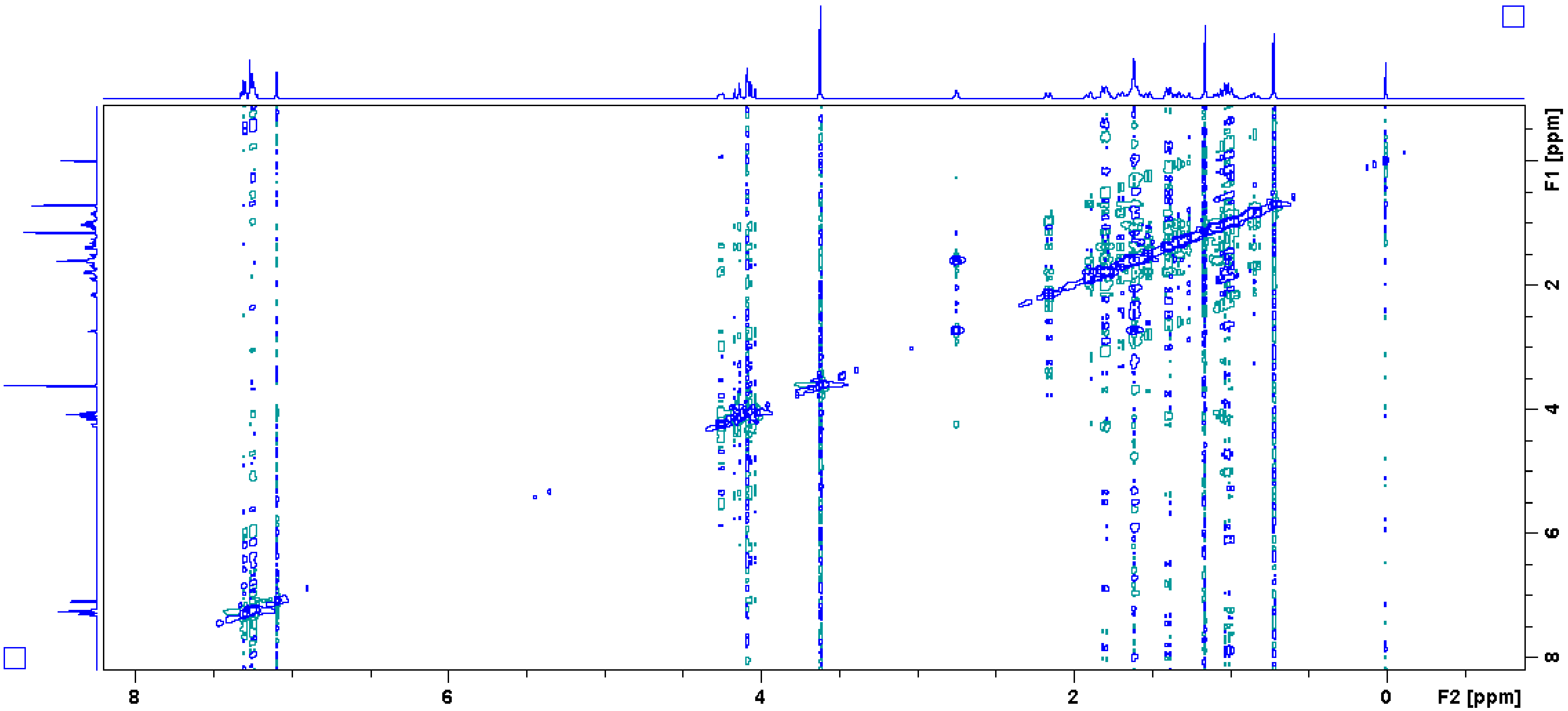

HSQC of compound (4*R*,4a*S*,6a*R*,8*R*,9*S*,11a*R*,11b*S*)-methyl 9-((4-benzyl-1*H*-1,2,3-triazol-1-yl)methyl)-8-hydroxy-4,11b-dimethyltetradecahydro-6a,9-methanocyclohepta[a]naphthalene-4-carboxylate **(36)**

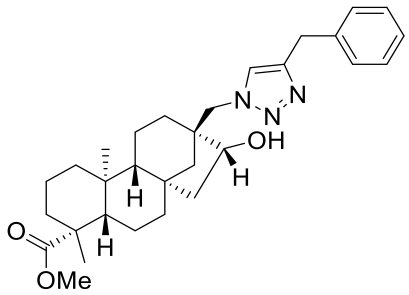

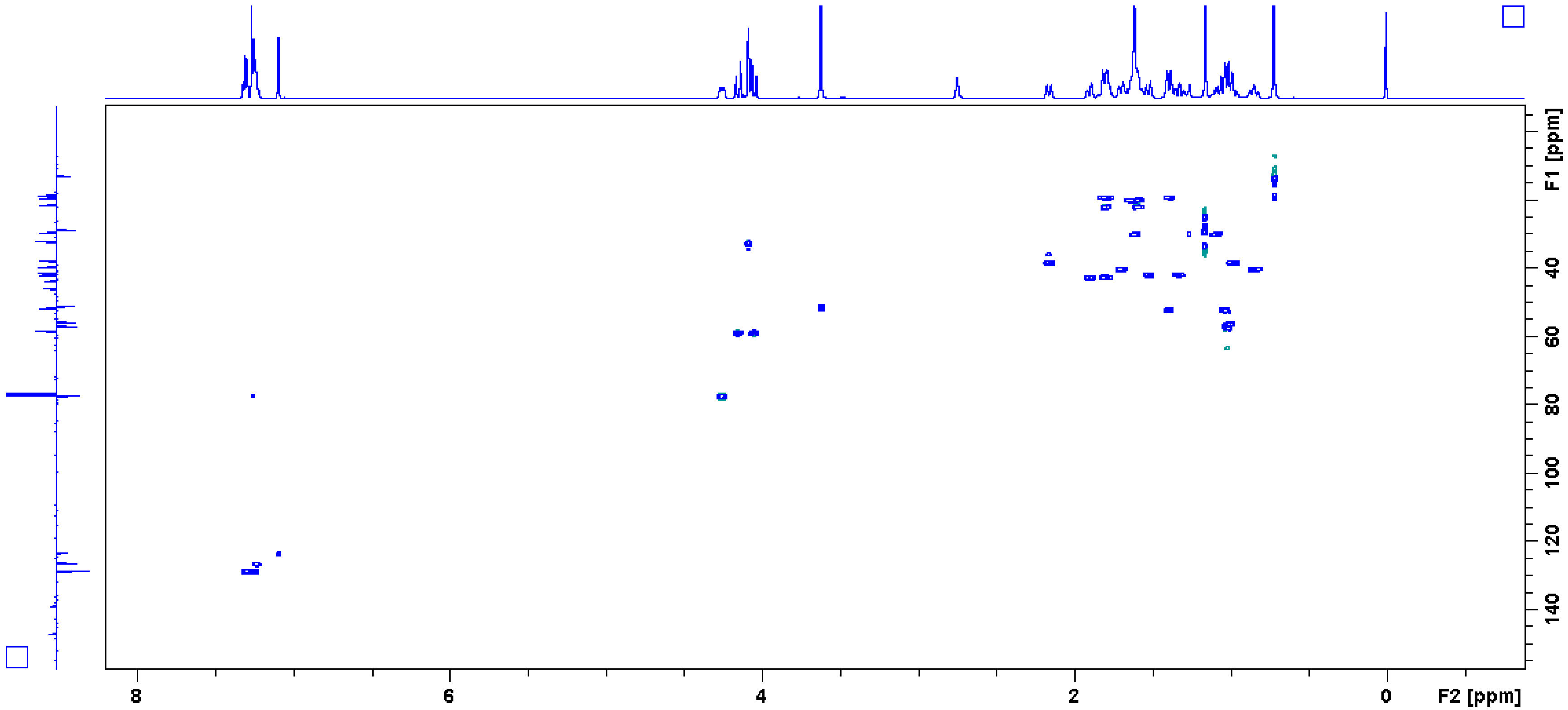

HMBC of compound (4*R*,4a*S*,6a*R*,8*R*,9*S*,11a*R*,11b*S*)-methyl 9-((4-benzyl-1*H*-1,2,3-triazol-1-yl)methyl)-8-hydroxy-4,11b-dimethyltetradecahydro-6a,9-methanocyclohepta[a]naphthalene-4-carboxylate **(36)**

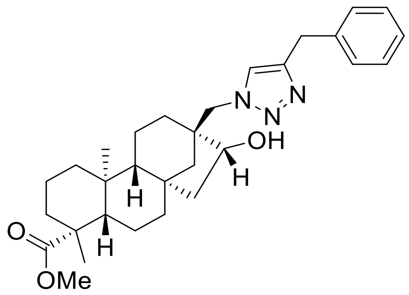

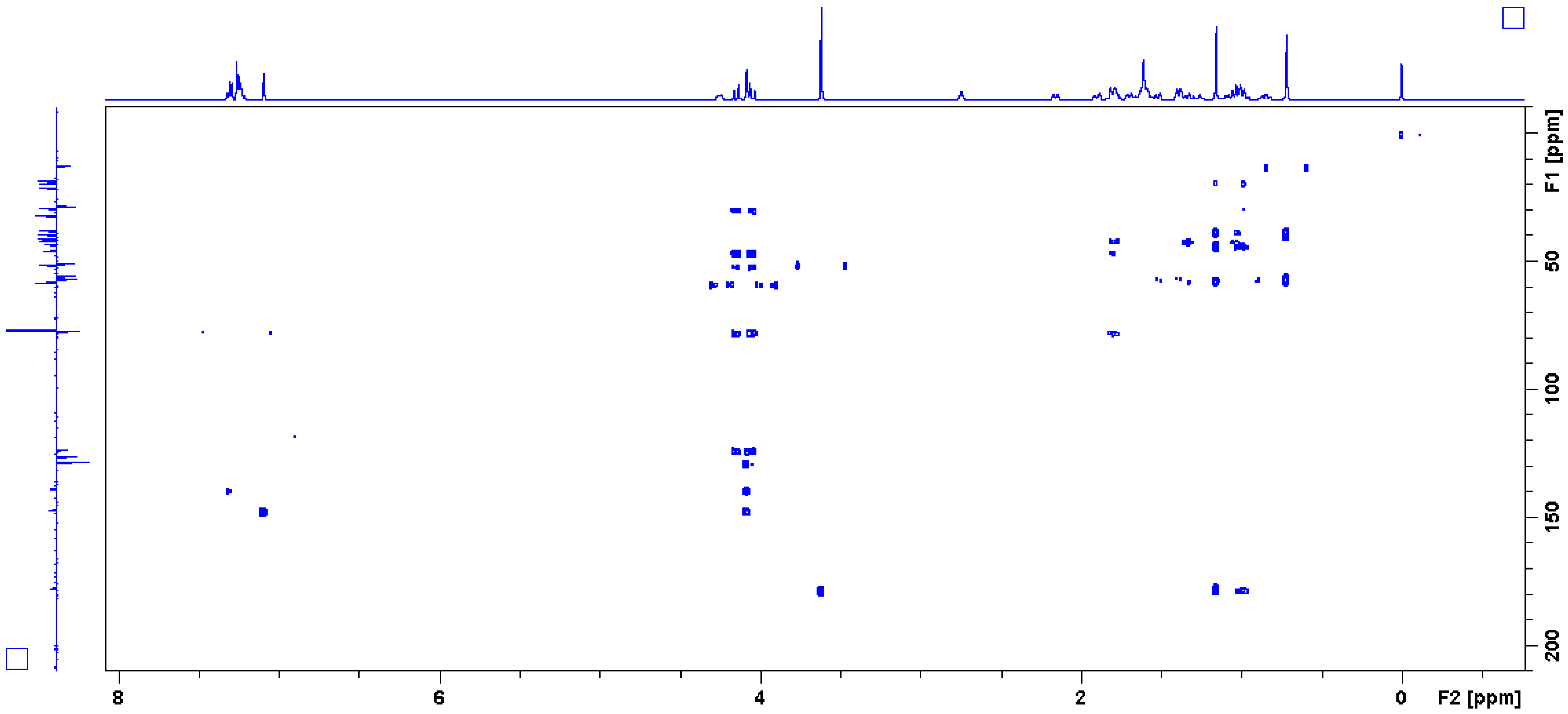

HRMS of compound (2'*S*,4*R*,4a*S*,6a*S*,11a*R*,11b*S*)-methyl 9-hydroxy-4,11b-dimethyldodecahydro-1H-spiro[6a,9-methanocyclohepta[a]naphthalene-8,2'-oxirane]-4-carboxylate **(3)**

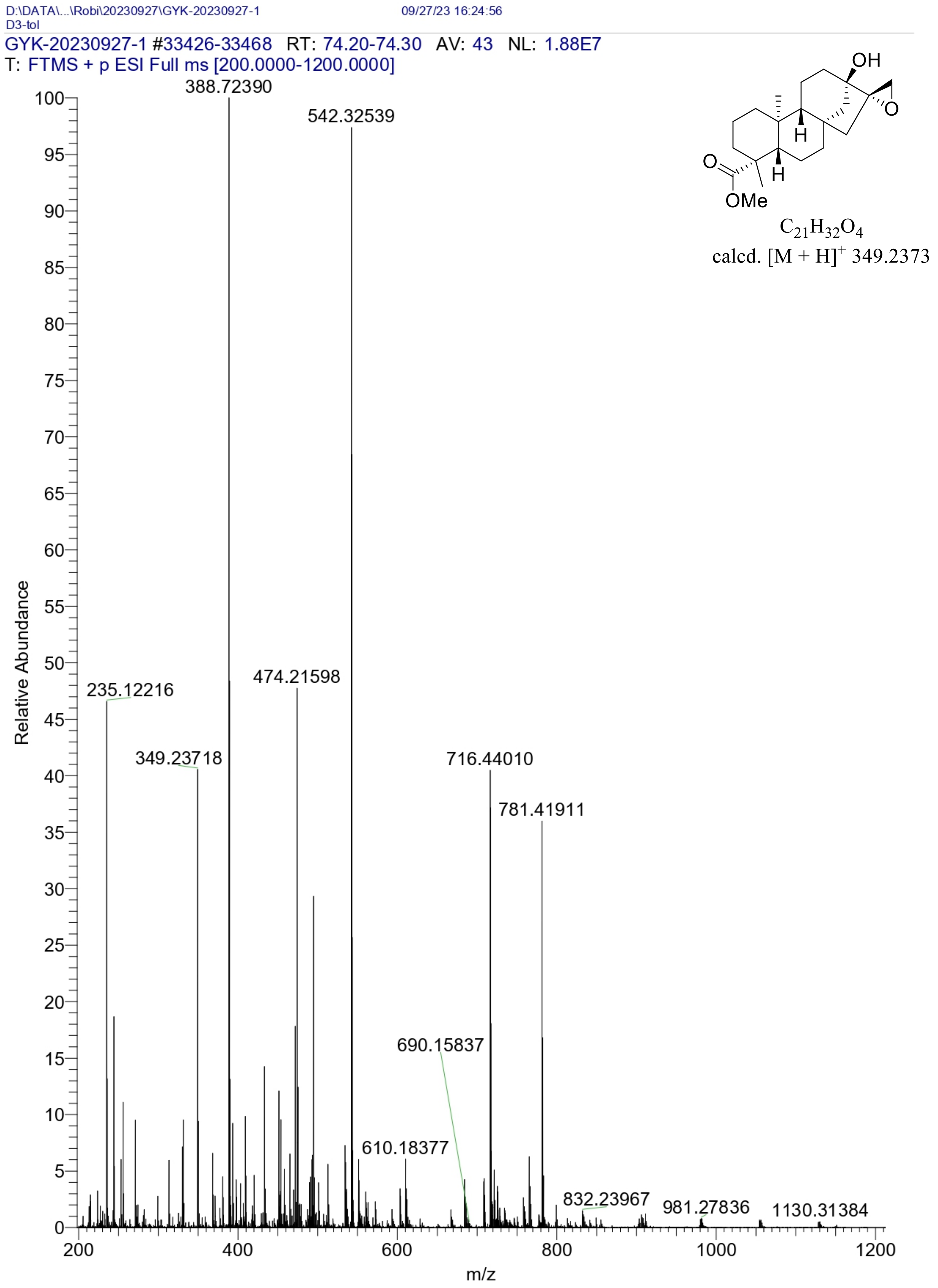

HRMS of compound (4*R*,4a*S*,6a*R*,9*S*,11a*R*,11b*S*)-methyl 9-(hydroxymethyl)-4,11b-dimethyl-8-oxotetradecahydro-6a,9-methanocyclohepta[a]naphthalene-4-carboxylate **(4)**

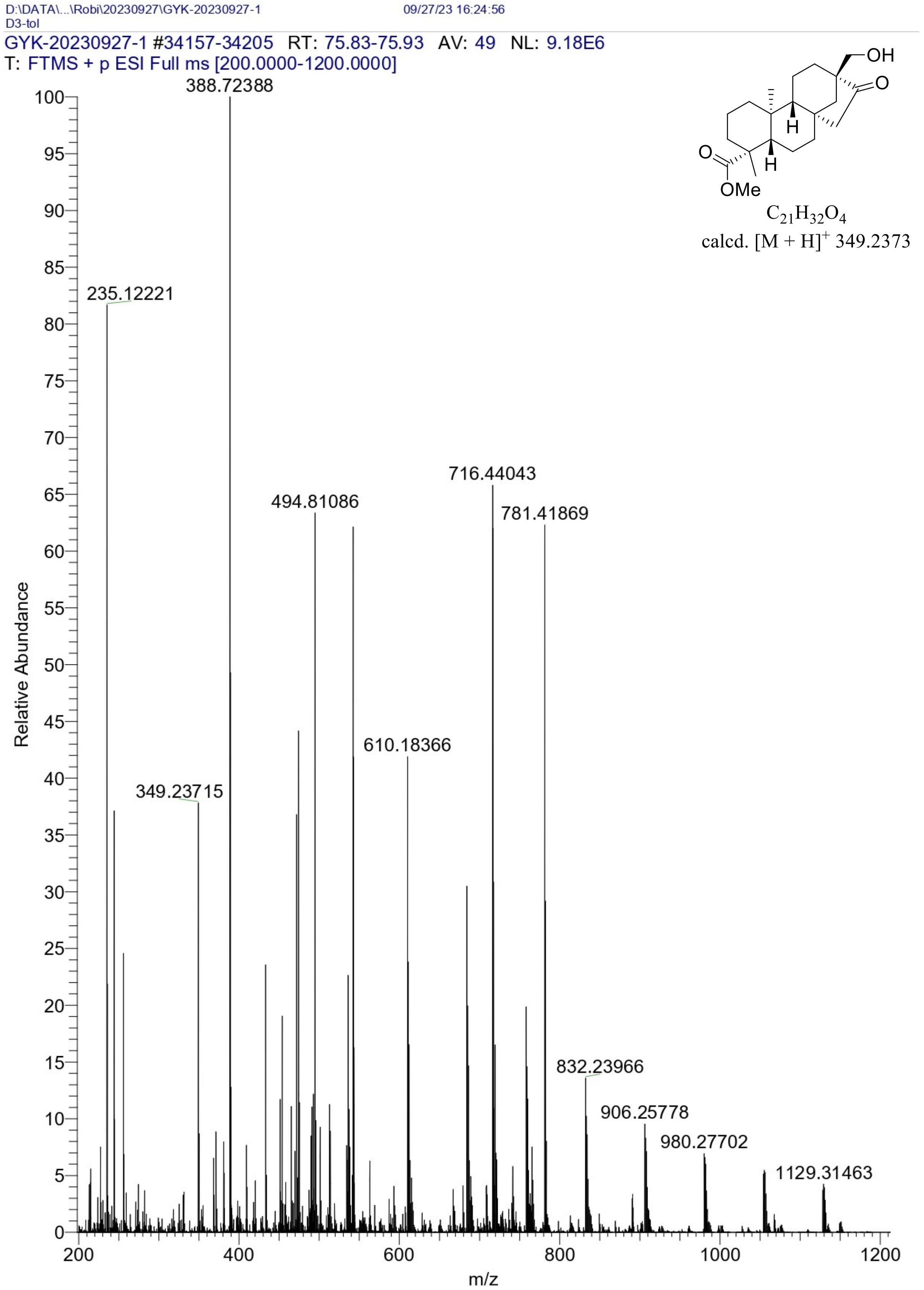

HRMS of compound (4*R*,4a*S*,6a*R*,9*R*,11a*R*,11b*S*,E)-methyl 8-(hydroxyimino)-9-(hydroxymethyl)-4,11b-dimethyltetradecahydro-6a,9-methanocyclohepta[a]naphthalene-4-carboxylate **(5)**

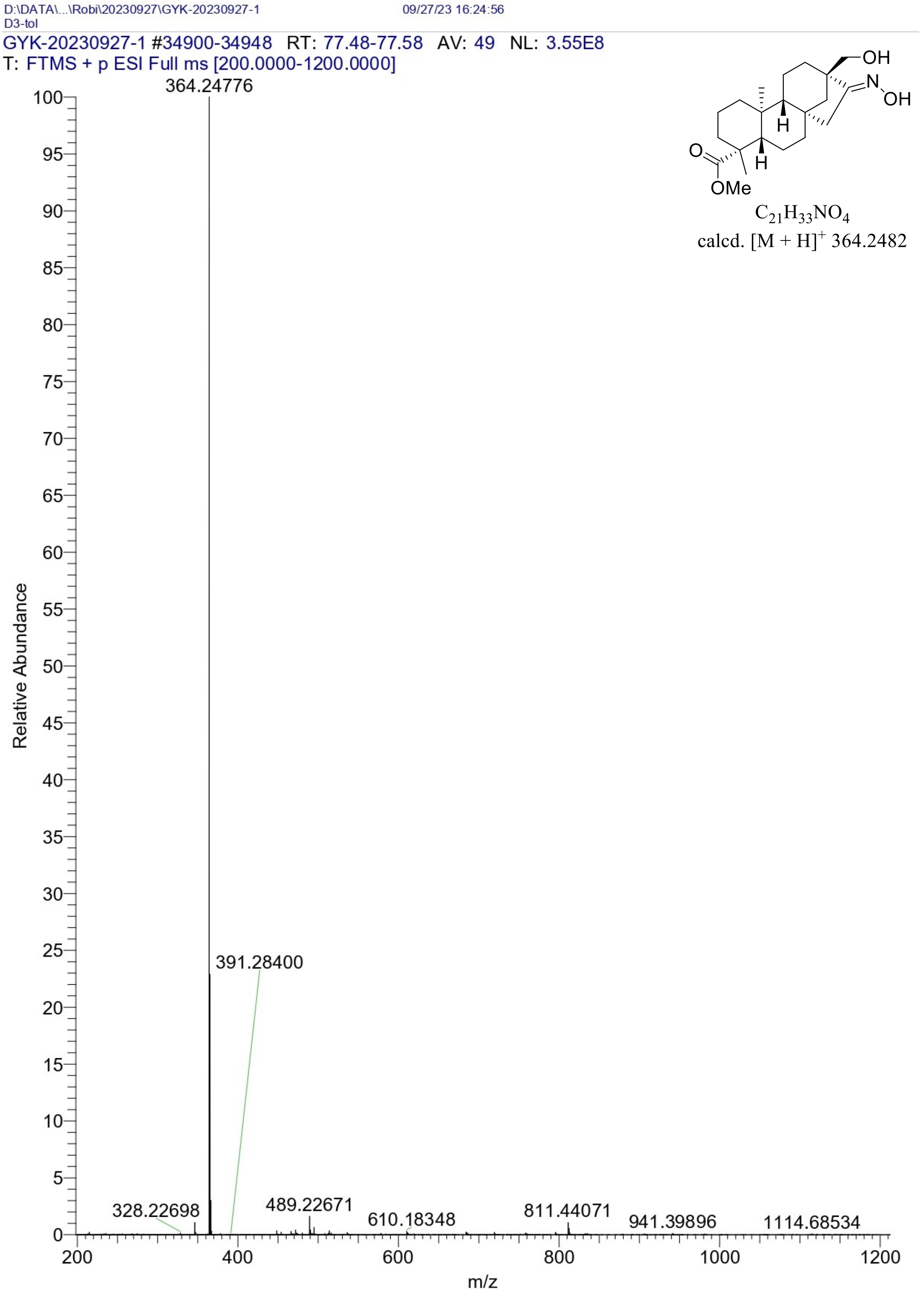

HRMS of compound (4*R*,4a*S*,6a*R*,8*R*,9*R*,11a*R*,11b*S*)-methyl 8-amino-9-(hydroxymethyl)-4,11b-dimethyltetradecahydro-6a,9-methanocyclohepta[a]naphthalene-4-carboxylate **(6)**

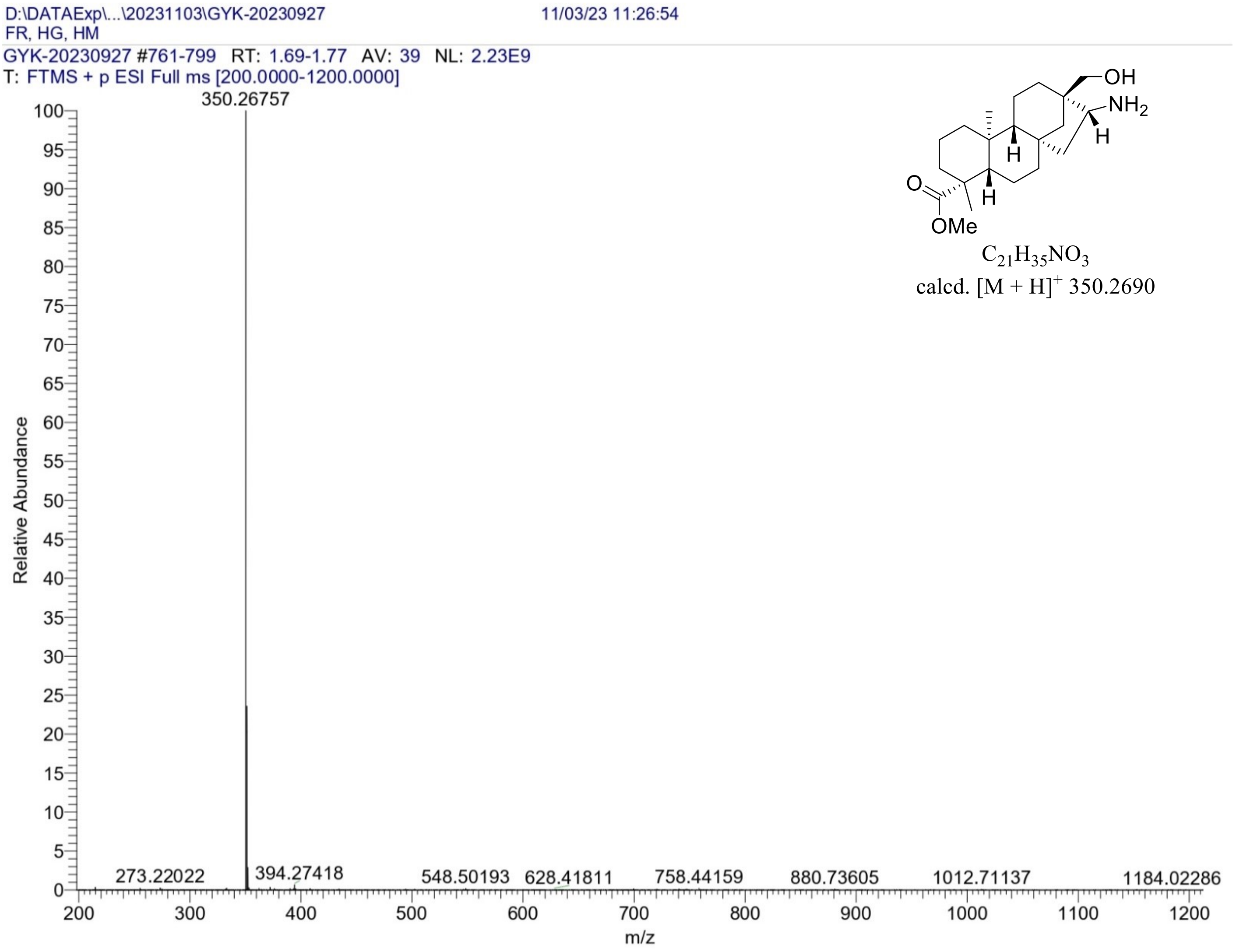

HRMS of compound (4*R*,4a*S*,6a*R*,8*S*,9*R*,11a*R*,11b*S*)-methyl 8-amino-9-(hydroxymethyl)-4,11b-dimethyltetradecahydro-6a,9-methanocyclohepta[a]naphthalene-4-carboxylate **(7)**

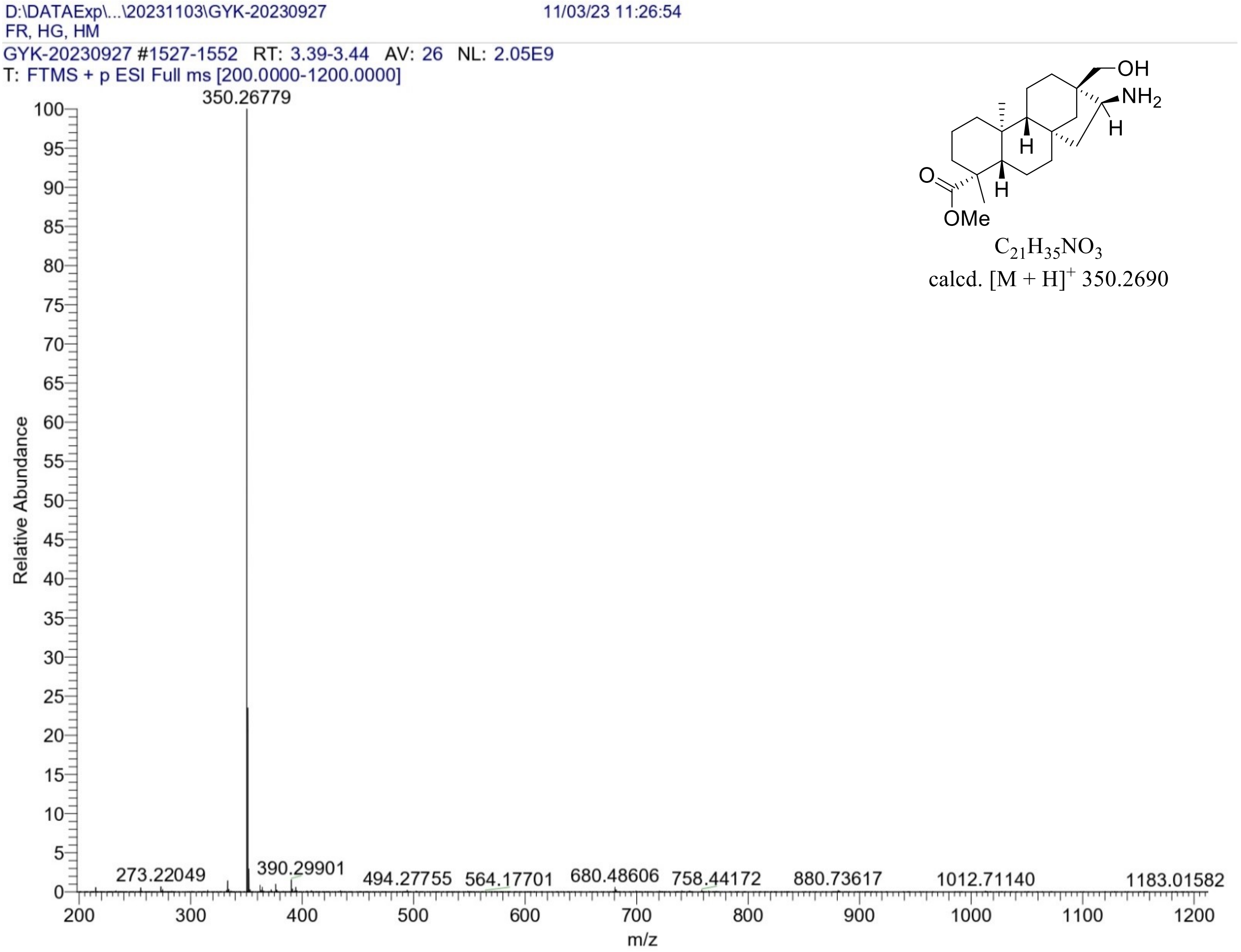

HRMS of compound (4*R*,4a*S*,6a*R*,8*R*,9*R*,11a*R*,11b*S*)-methyl 8-(benzylamino)-9-(hydroxymethyl)-4,11b-dimethyltetradecahydro-6a,9-methanocyclohepta[a]naphthalene-4-carboxylate **(8)**

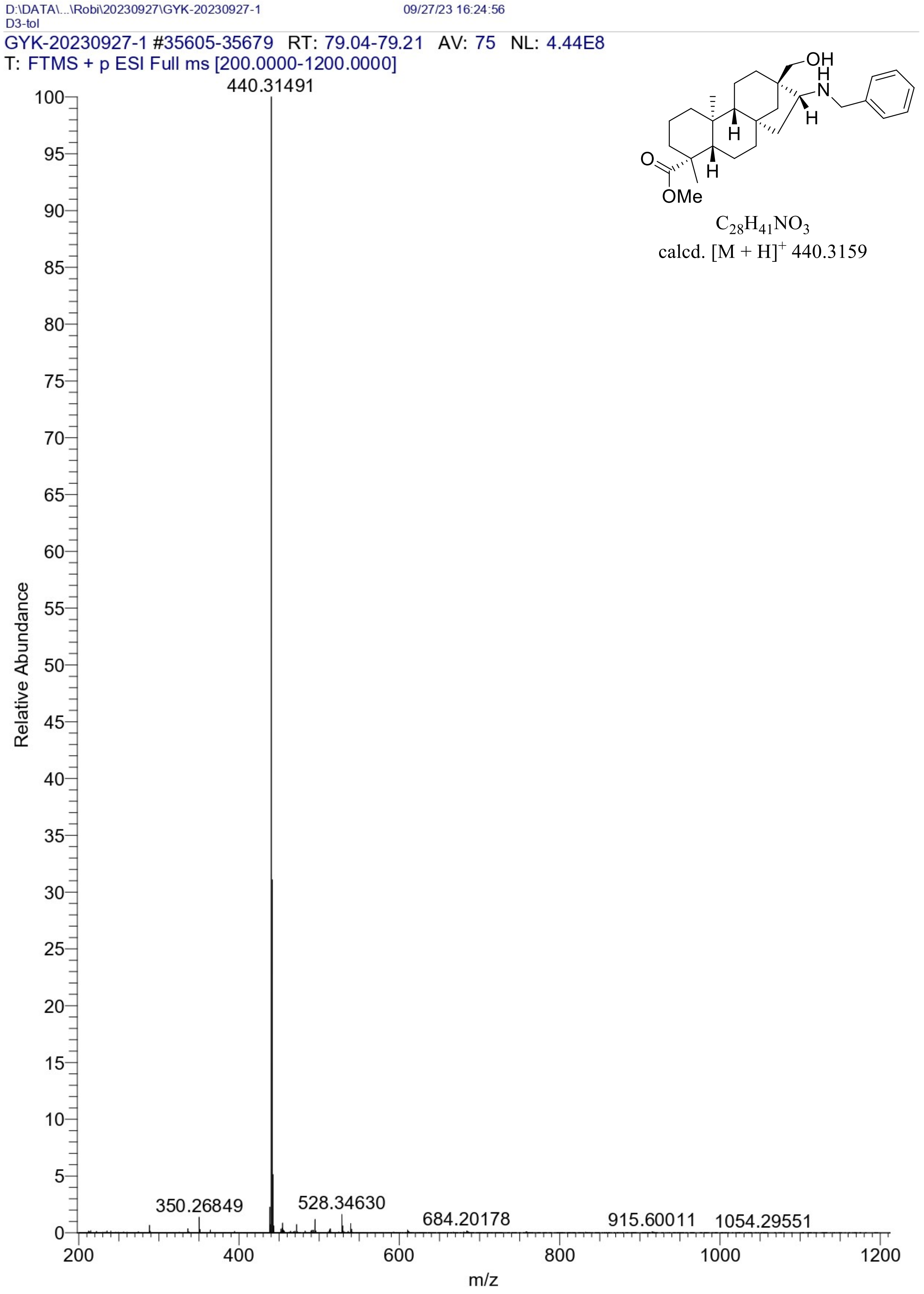

$C_{28}H_{41}NO_3$
calcd. $[M + H]^+$ 440.3159

HRMS of compound (4*R*,4a*S*,6a*R*,8*R*,9*R*,11a*R*,11b*S*)-methyl 8-((4-fluorobenzyl)amino)-9-(hydroxymethyl)-4,11b-dimethyltetradecahydro-6a,9-methanocyclohepta[a]naphthalene-4-carboxylate **(9)**

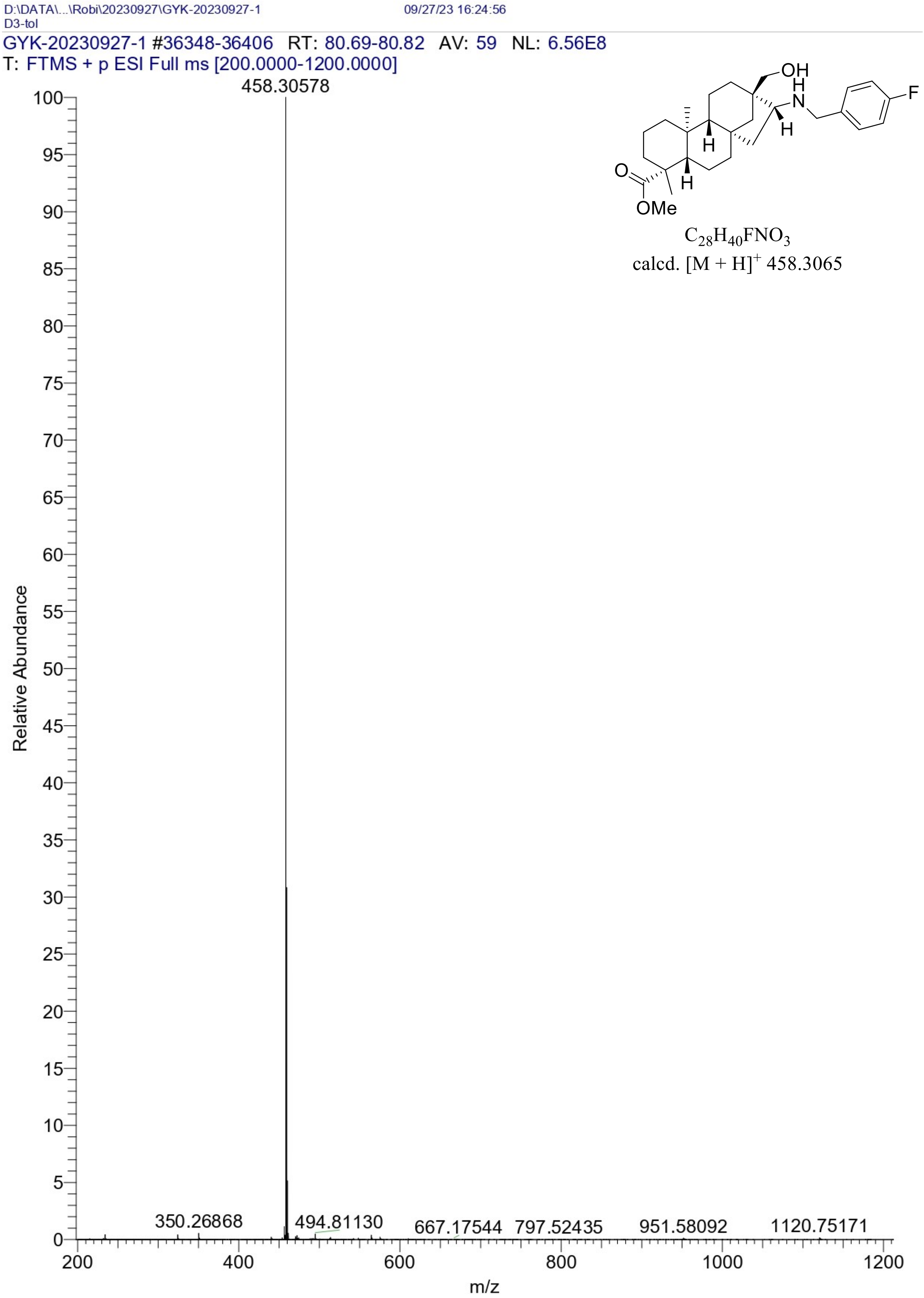

HRMS of compound (4*R*,4a*S*,6a*R*,8*R*,9*R*,11a*R*,11b*S*)-methyl 9-(hydroxymethyl)-8-((4-methoxybenzyl)amino)-4,11b-dimethyltetradecahydro-6a,9-methanocyclohepta[a]naphthalene-4-carboxylate **(10)**

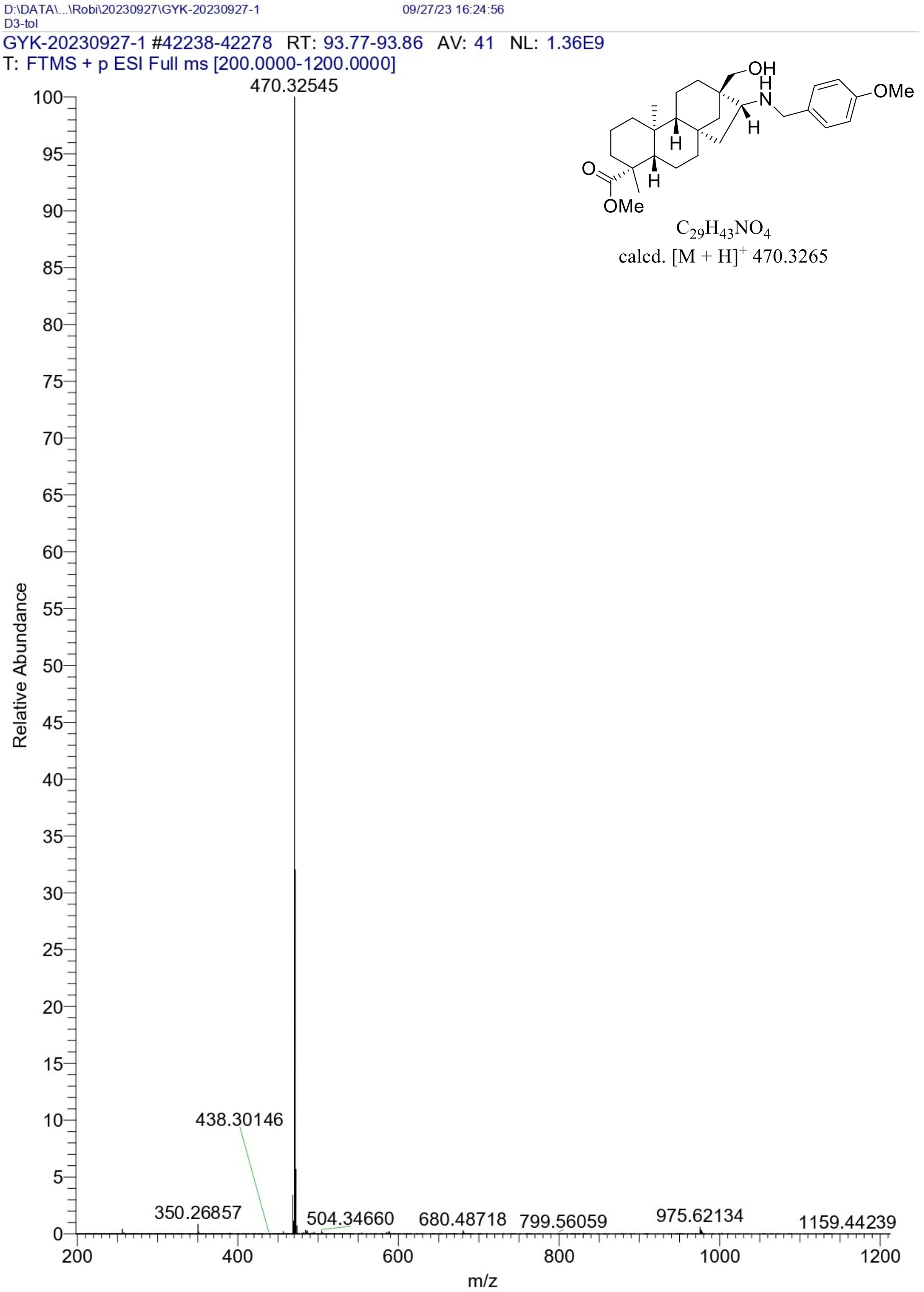

HRMS of compound (4*R*,4a*S*,6a*R*,8*R*,9*R*,11a*R*,11b*S*)-methyl 9-(hydroxymethyl)-4,11b-dimethyl-8-(((*S*)-1-phenylpropyl)amino)tetradecahydro-6a,9-methanocyclohepta[a]naphthalene-4-carboxylate **(11)**

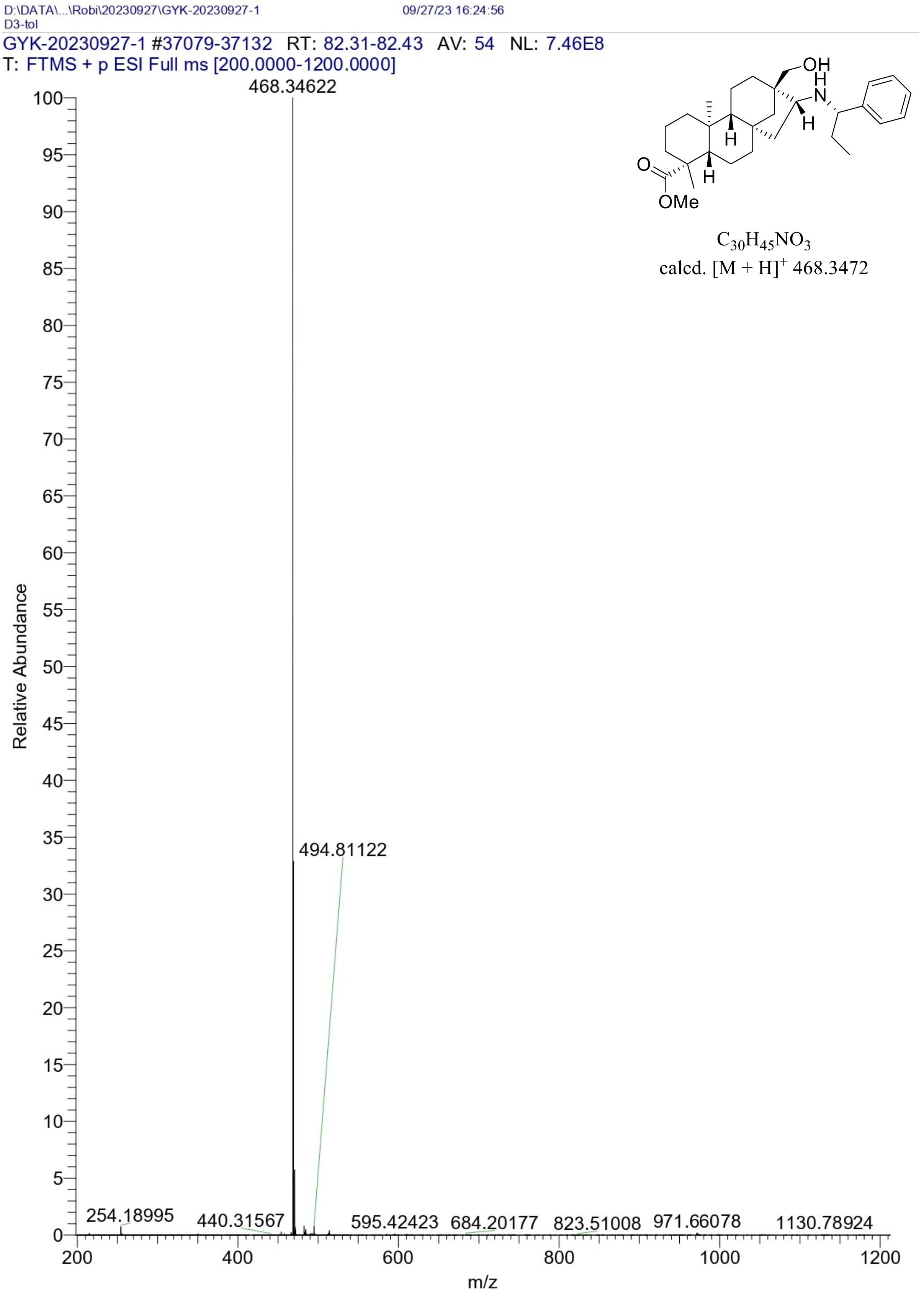

HRMS of compound (4*R*,4a*S*,6a*R*,8*R*,9*R*,11a*R*,11b*S*)-methyl 9-(hydroxymethyl)-4,11b-dimethyl-8-(((*R*)-1-phenylpropyl)amino)tetradecahydro-6a,9-methanocyclohepta[a]naphthalene-4-carboxylate **(12)**

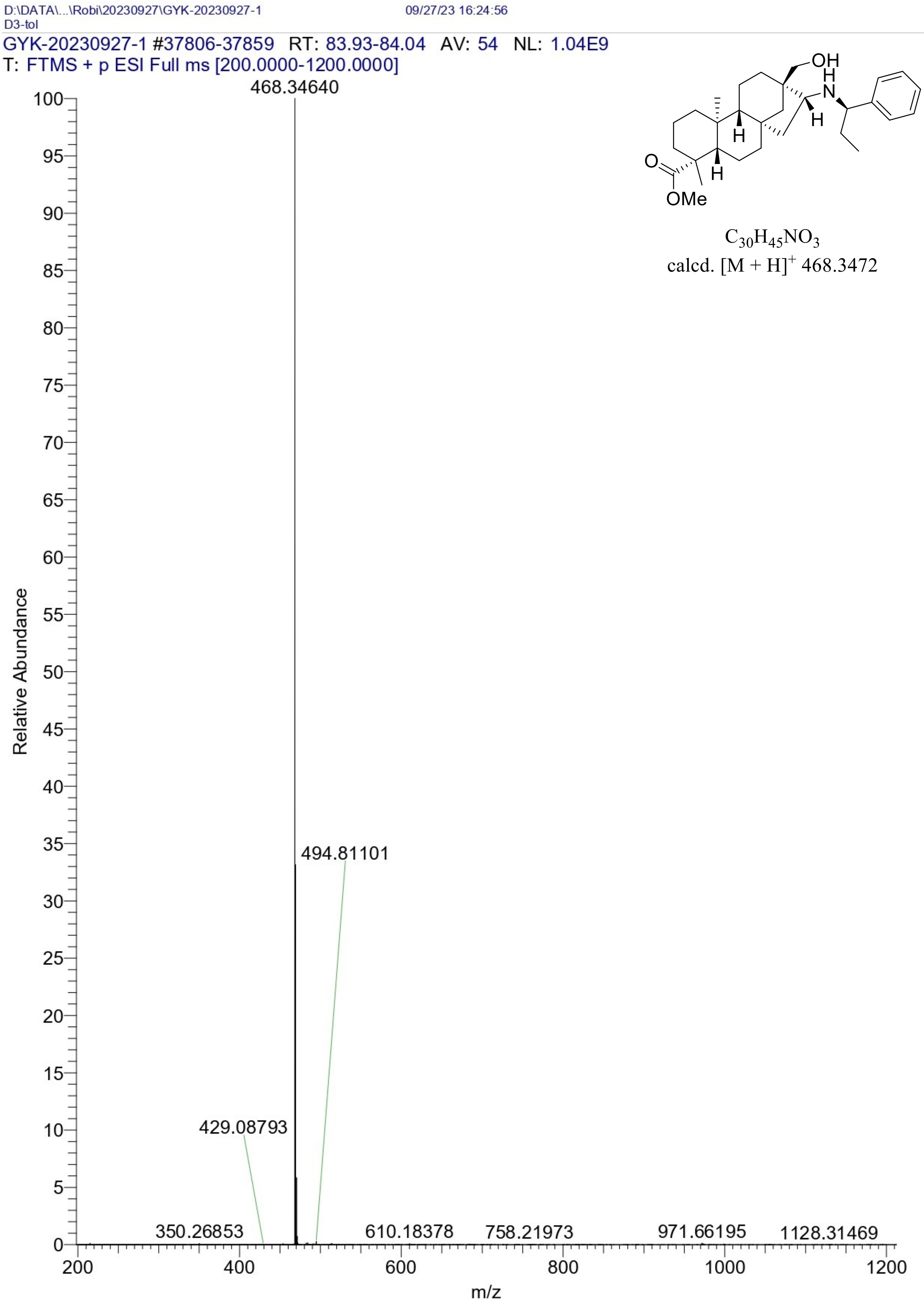

HRMS of compound (4*R*,4a*S*,6a*R*,8*R*,9*R*,11a*R*,11b*S*)-methyl 9-(hydroxymethyl)-4,11b-dimethyl-8-(((*R*)-1-(naphthalen-1-yl)ethyl)amino)tetradecahydro-6a,9-methanocyclohepta[a]naphthalene-4-carboxylate **(13)**

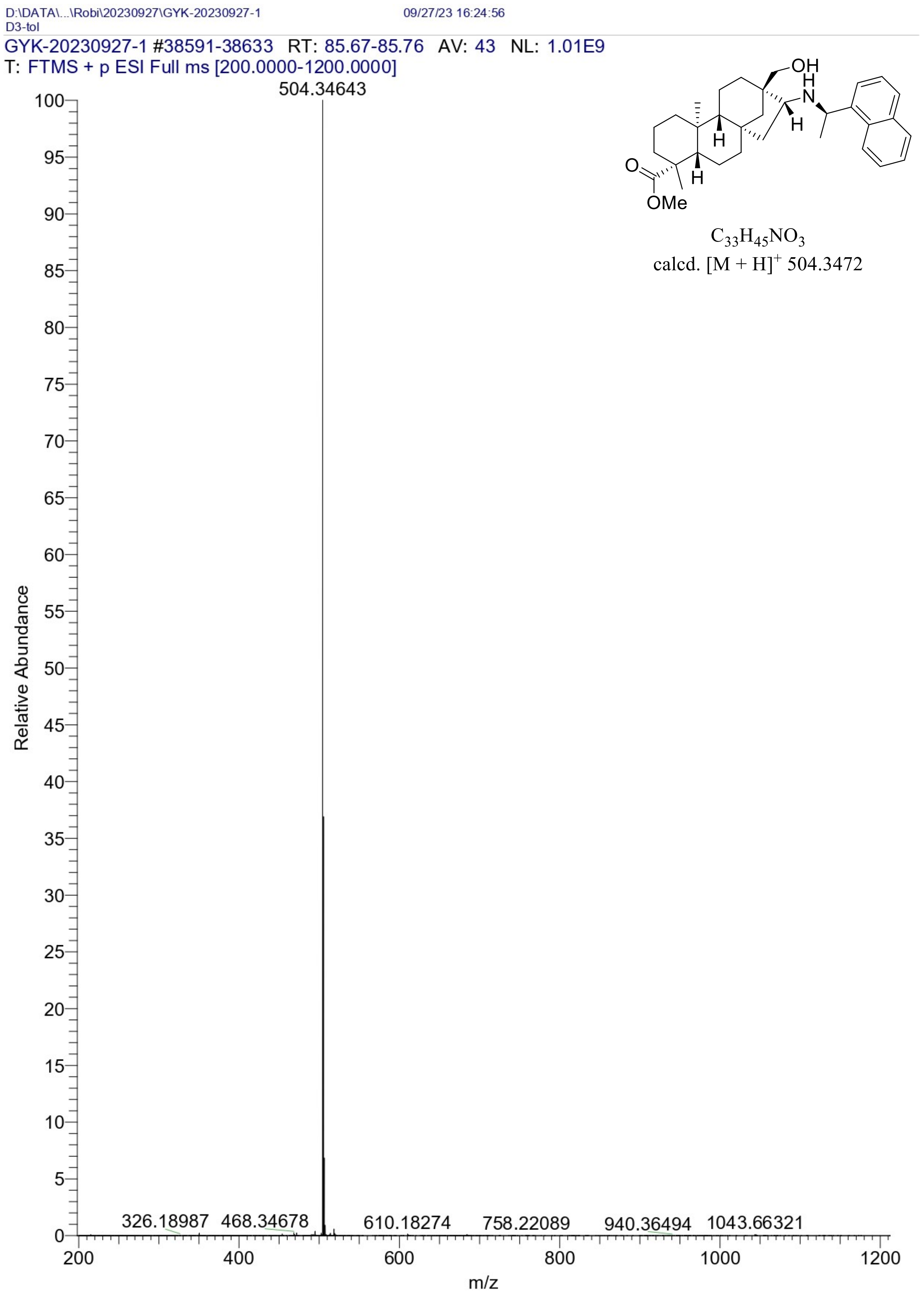

HRMS of compound (4*R*,4a*S*,6a*R*,8*R*,9*R*,11a*R*,11b*S*)-methyl 9-(hydroxymethyl)-4,11b-dimethyl-8-(((*S*)-1-(naphthalen-1-yl)ethyl)amino)tetradecahydro-6a,9-methanocyclohepta[a]naphthalene-4-carboxylate **(14)**

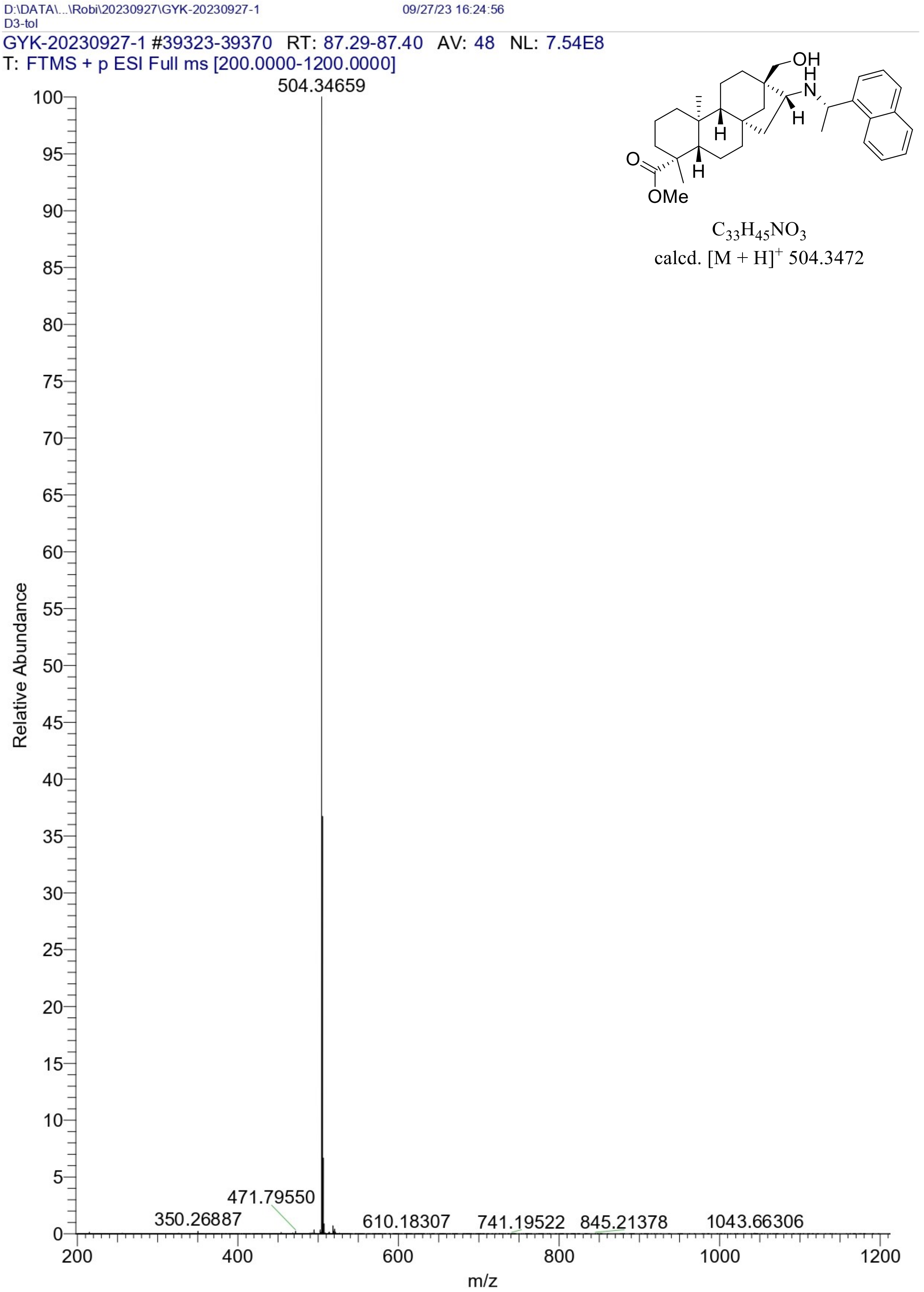

$C_{33}H_{45}NO_3$

calcd. $[M + H]^+$ 504.3472

HRMS of compound (4*R*,4a*S*,6a*R*,8*R*,9*R*,11a*R*,11b*S*)-methyl 9-(hydroxymethyl)-4,11b-dimethyl-8-((naphthalen-1-ylmethyl)amino)tetradecahydro-6a,9-methanocyclohepta[a]naphthalene-4-carboxylate **(15)**

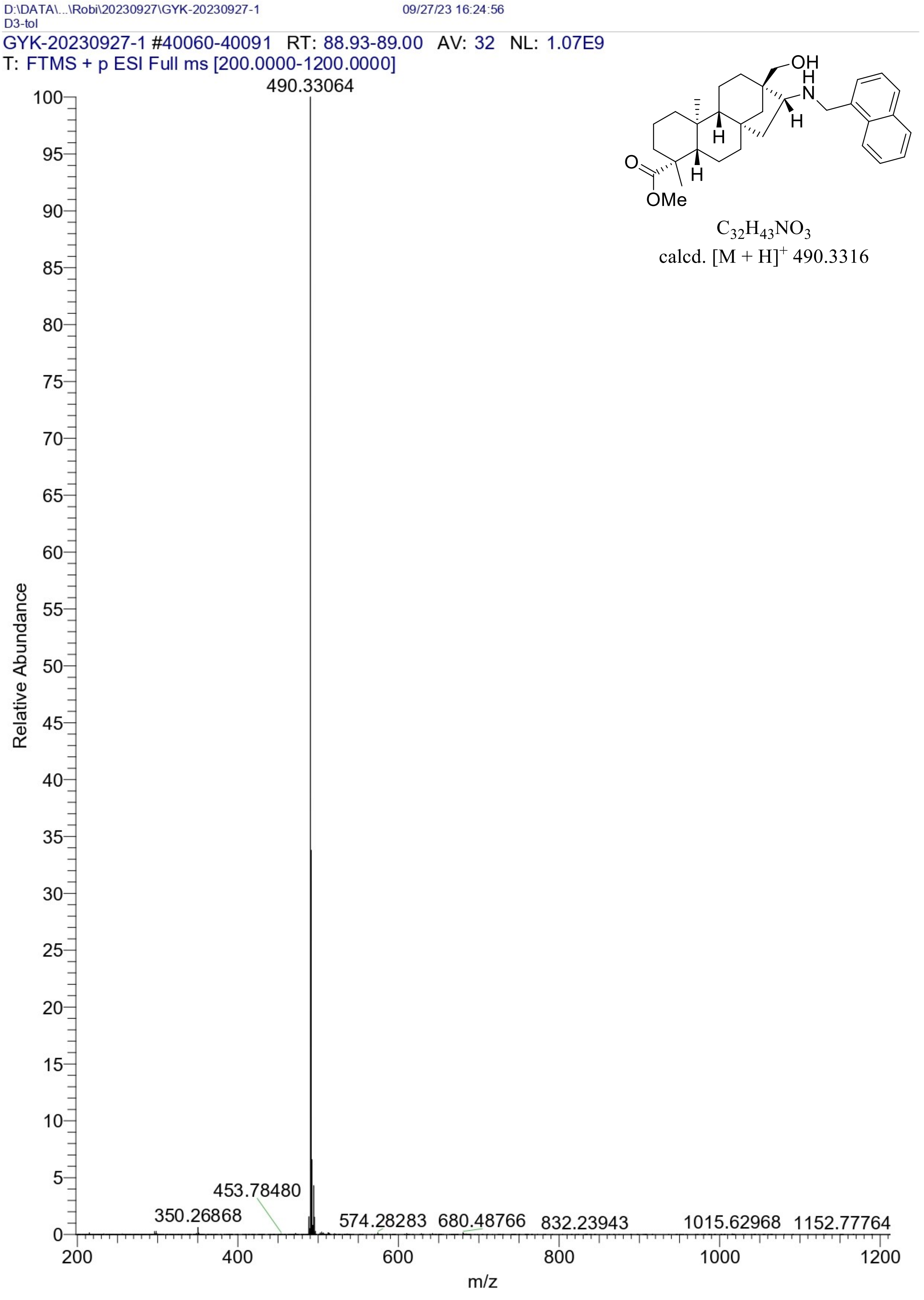

$C_{32}H_{43}NO_3$

calcd. $[M + H]^+$ 490.3316

HRMS of compound (4*R*,4a*S*,6a*R*,8*R*,9*R*,11a*R*,11b*S*)-methyl 9-(hydroxymethyl)-4,11b-dimethyl-8-(((*S*)-1-(naphthalen-2-yl)ethyl)amino)tetradecahydro-6a,9-methanocyclohepta[a]naphthalene-4-carboxylate **(16)**

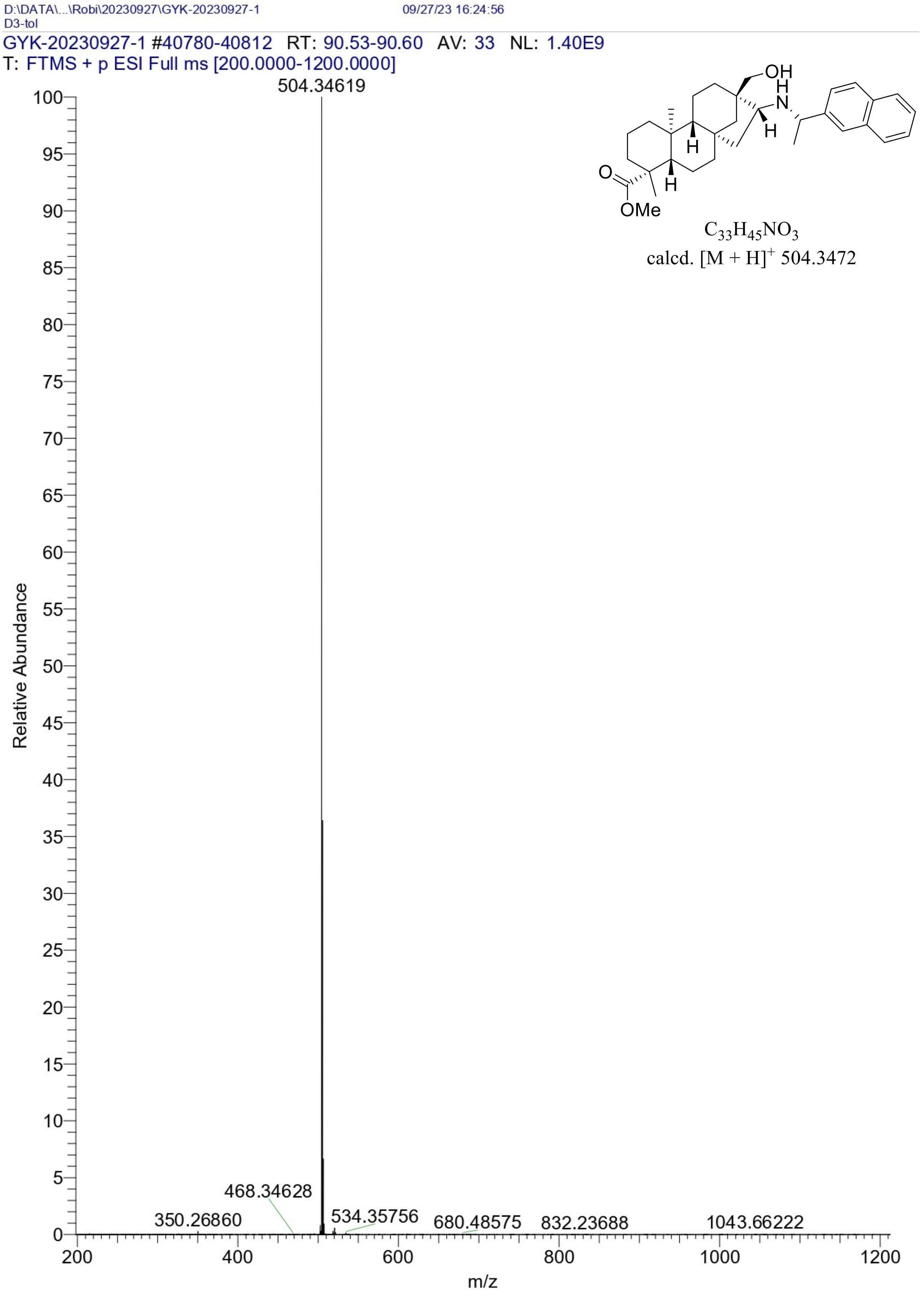

HRMS of compound (4*R*,4a*S*,6a*R*,8*R*,9*R*,11a*R*,11b*S*)-methyl 9-(hydroxymethyl)-4,11b-dimethyl-8-(((*R*)-1-(naphthalen-2-yl)ethyl)amino)tetradecahydro-6a,9-methanocyclohepta[a]naphthalene-4-carboxylate **(17)**

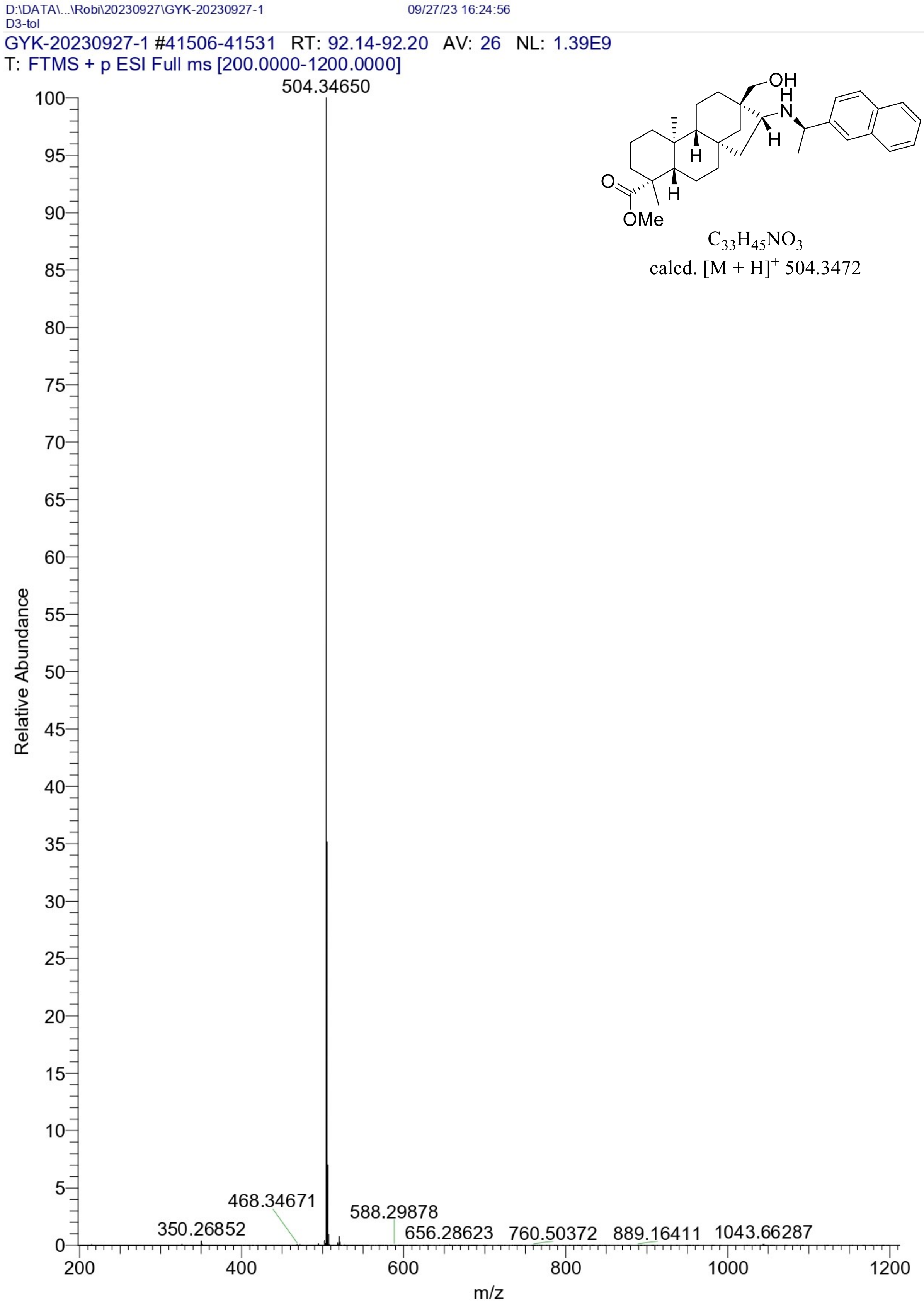

HRMS of compound (4*R*,4a*S*,6a*R*,9*S*,11a*R*,11b*S*)-methyl 4,11b-dimethyl-9-(((methylsulfonyl)oxy)methyl)-8-oxotetradecahydro-6a,9-methanocyclohepta[a]naphthalene-4-carboxylate **(18)**

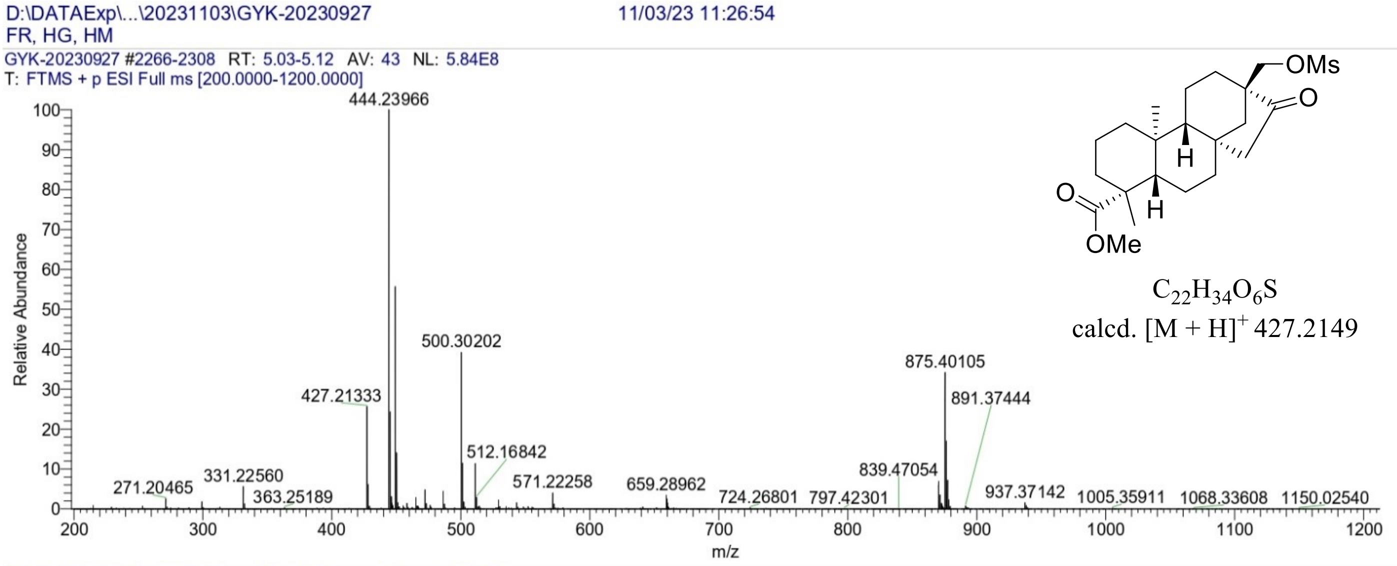

$C_{22}H_{34}O_6S$

calcd. $[M + H]^+$ 427.2149

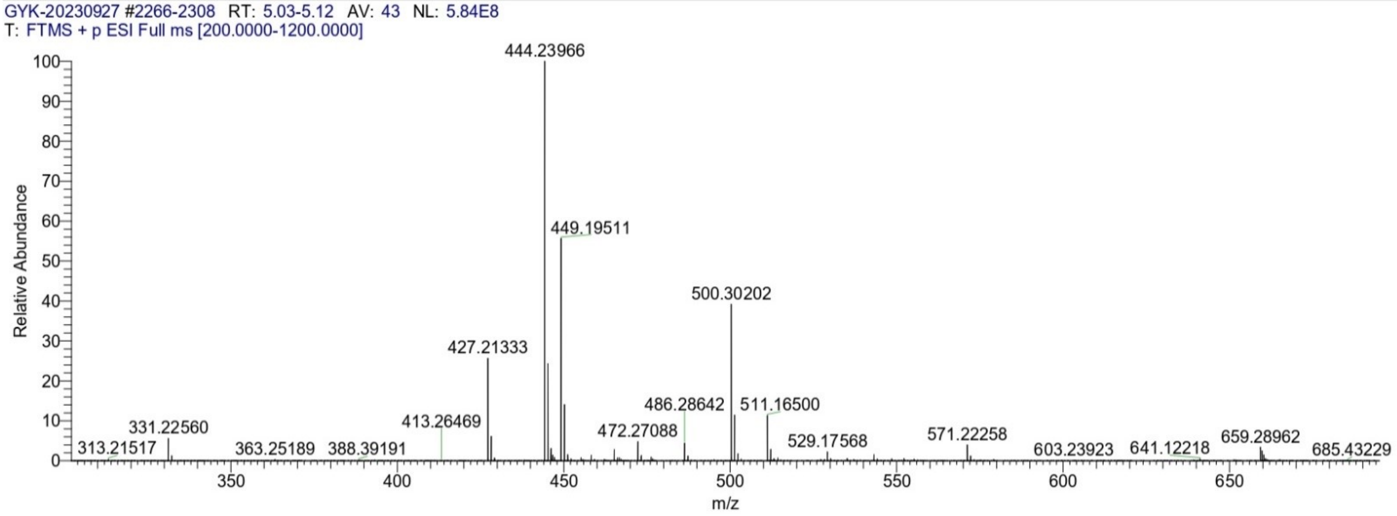

HRMS of compound (4*R*,4a*S*,6a*R*,9*S*,11a*R*,11b*S*)-methyl 9-(azidomethyl)-4,11b-dimethyl-8-oxotetradecahydro-6a,9-methanocyclohepta[a]naphthalene-4-carboxylate **(19)**

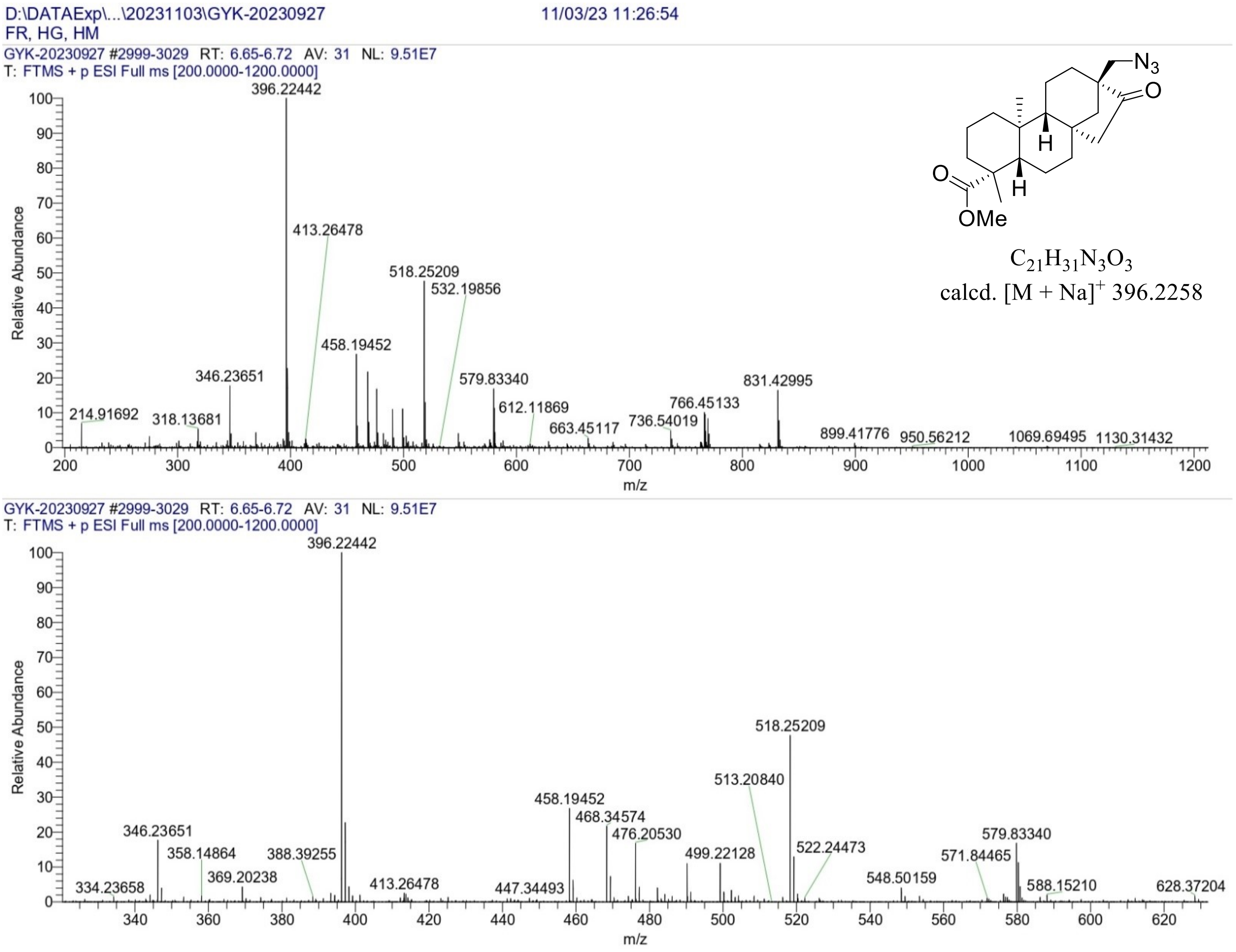

$C_{21}H_{31}N_3O_3$

calcd. $[M + Na]^+$ 396.2258

HRMS of compound (4*R*,4a*S*,6a*R*,8*R*,9*S*,11a*R*,11b*S*)-methyl 9-(azidomethyl)-8-hydroxy-4,11b-dimethyltetradecahydro-6a,9-methanocyclohepta[a]naphthalene-4-carboxylate **(20)**

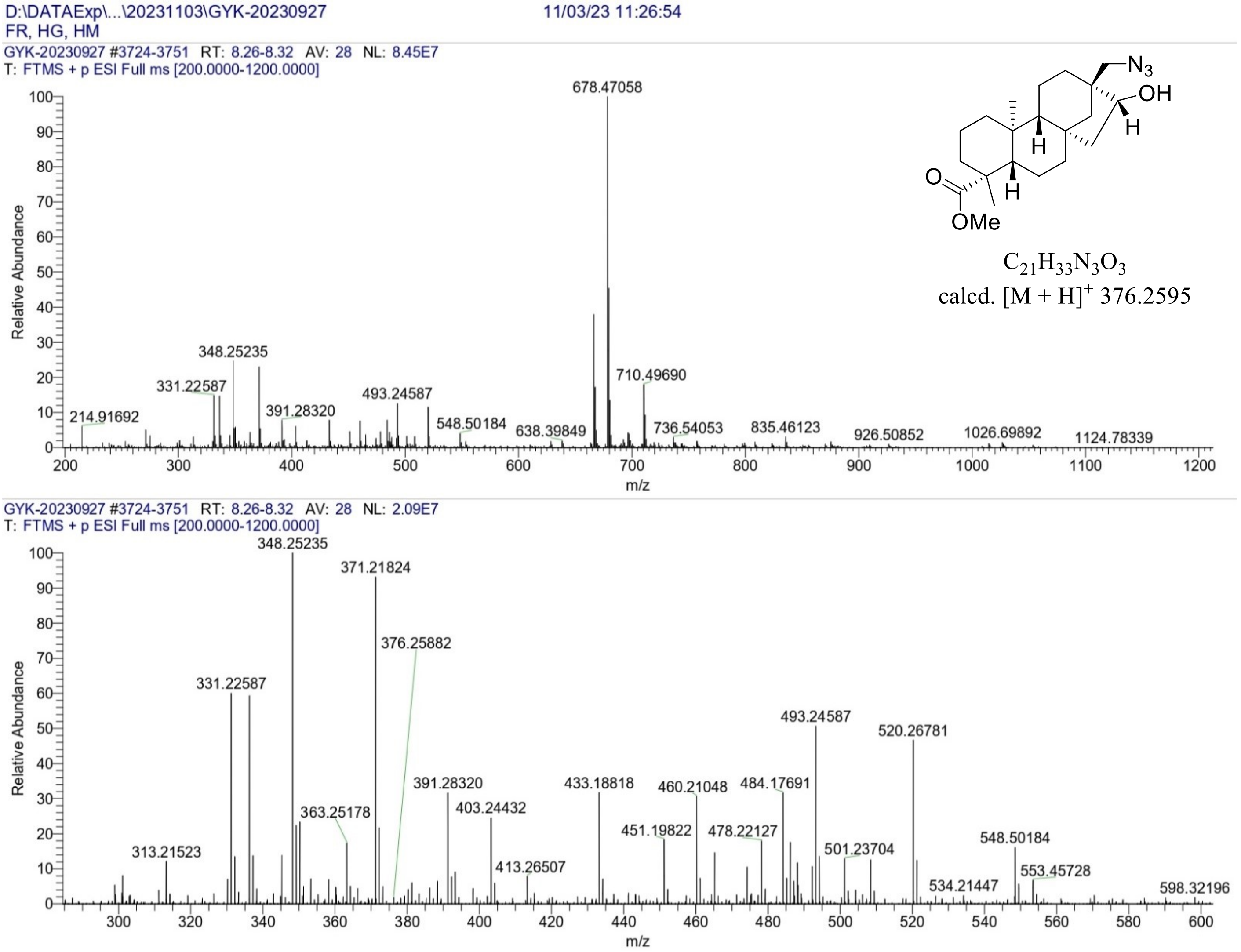

HRMS of compound (4*R*,4a*S*,6a*R*,8*R*,9*S*,11a*R*,11b*S*)-methyl 8-hydroxy-4,11b-dimethyl-9-(((methylsulfonyl)oxy)methyl)tetradecahydro-6a,9-methanocyclohepta[a]naphthalene-4-carboxylate **(21)**

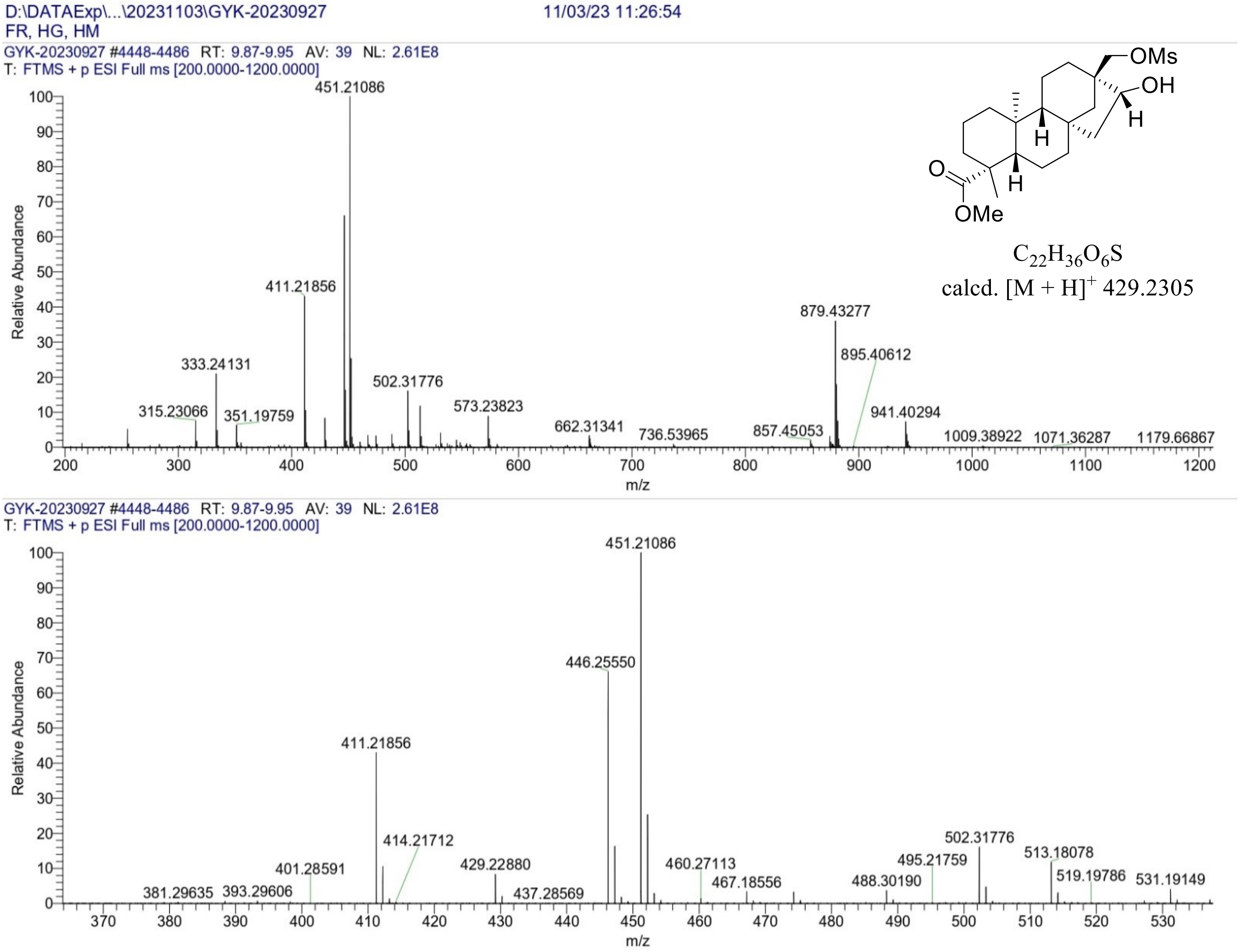

HRMS of compound (4*R*,4a*S*,6a*R*,8*R*,9*S*,11a*R*,11b*S*)-methyl 9-((benzylamino)methyl)-8-hydroxy-4,11b-dimethyltetradecahydro-6a,9-methanocyclohepta[a]naphthalene-4-carboxylate **(22)**

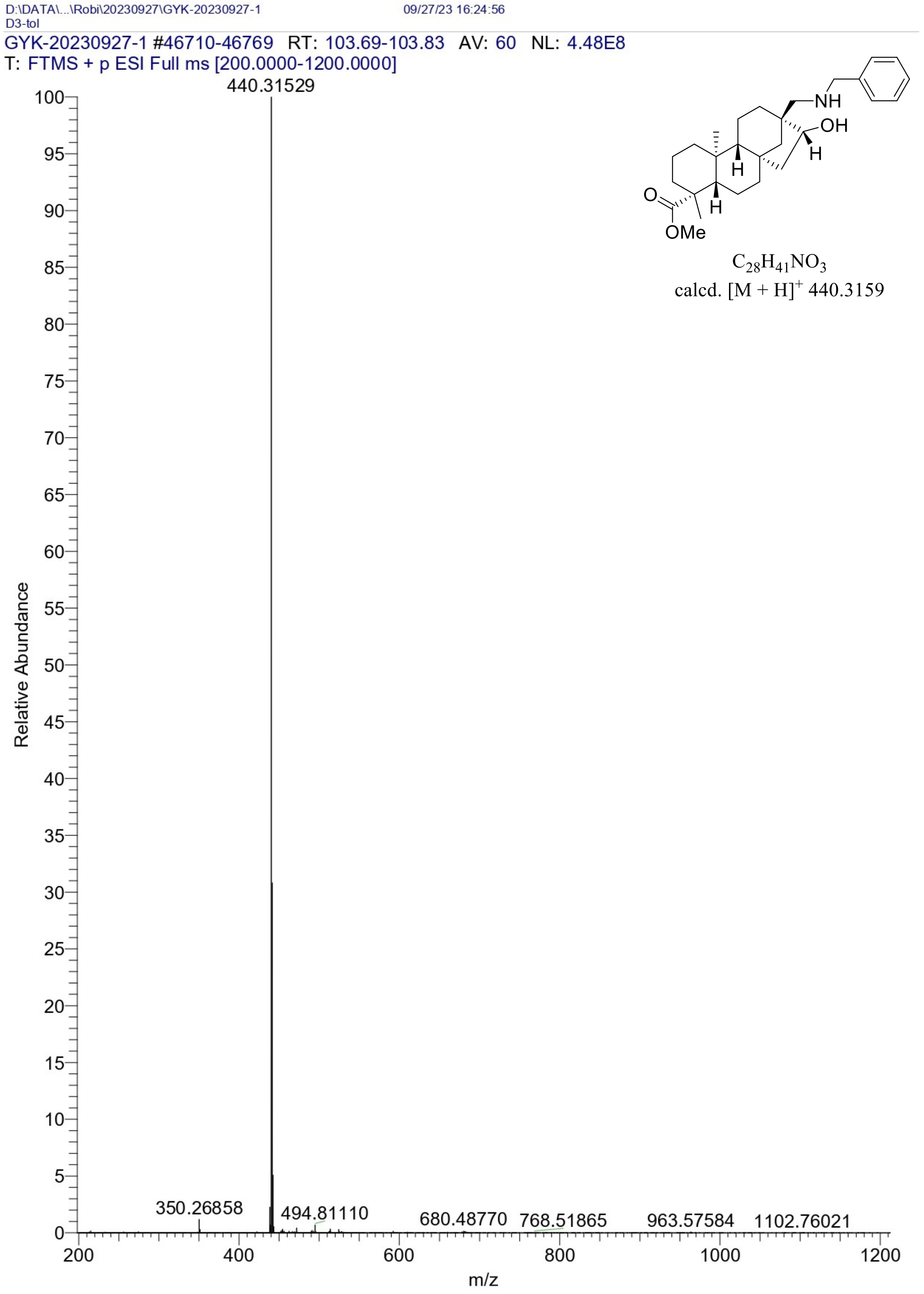

HRMS of compound (4*R*,4a*S*,6a*R*,8*R*,9*S*,11a*R*,11b*S*)-methyl 9-(((4-fluorobenzyl)amino)methyl)-8-hydroxy-4,11b-dimethyltetradecahydro-6a,9-methanocyclohepta[a]naphthalene-4-carboxylate **(23)**

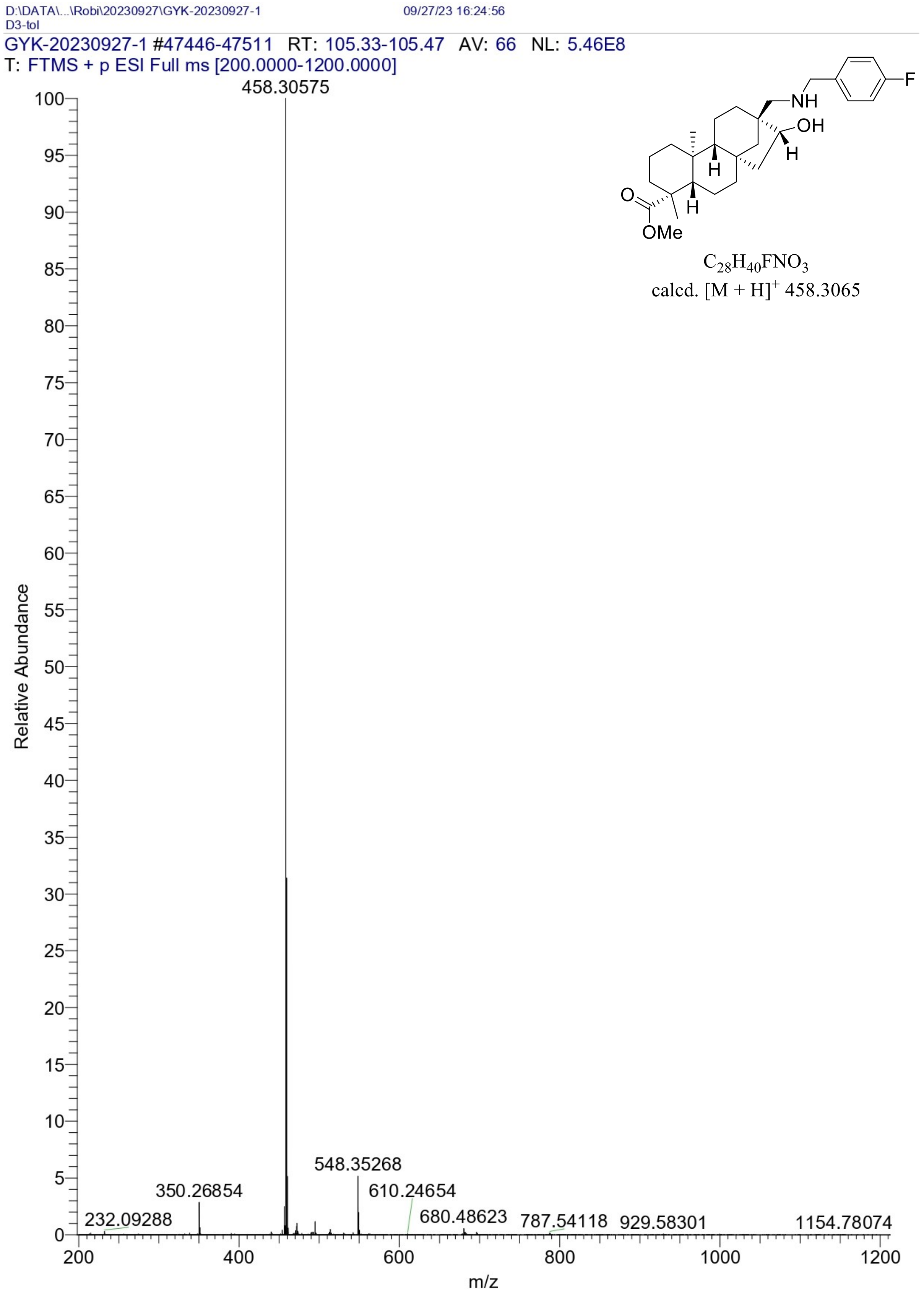

HRMS of compound (4*R*,4a*S*,6a*R*,8*R*,9*S*,11a*R*,11b*S*)-methyl 8-hydroxy-4,11b-dimethyl-9-((((*R*)-1-phenylpropyl)amino)methyl)tetradecahydro-6a,9-methanocyclohepta[a]naphthalene-4-carboxylate **(24)**

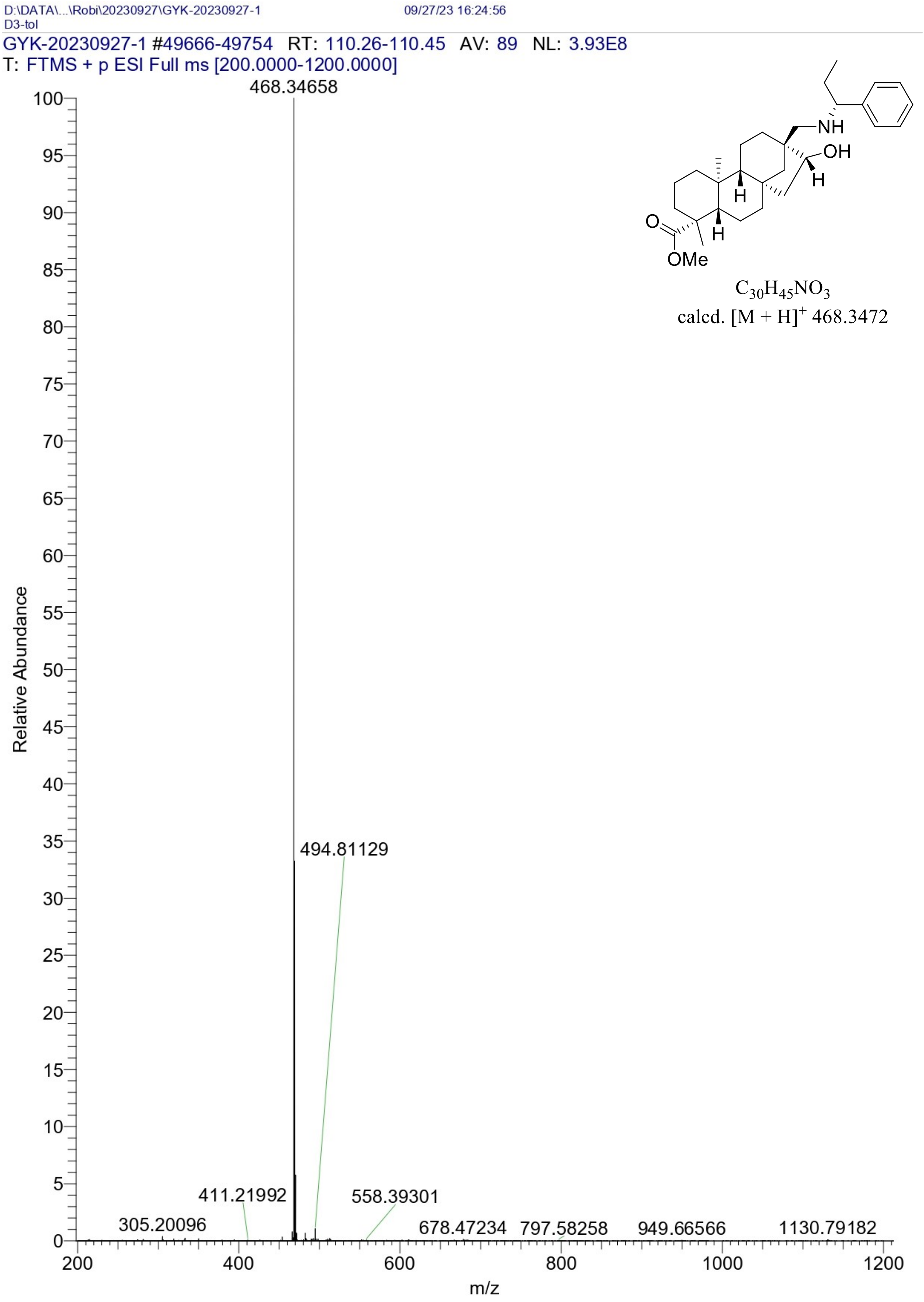

$C_{30}H_{45}NO_3$

calcd. $[M + H]^+$ 468.3472

HRMS of compound (4*R*,4a*S*,6a*R*,8*R*,9*S*,11a*R*,11b*S*)-methyl 8-hydroxy-4,11b-dimethyl-9-((((*S*)-1-phenylpropyl)amino)methyl)tetradecahydro-6a,9-methanocyclohepta[a]naphthalene-4-carboxylate **(25)**

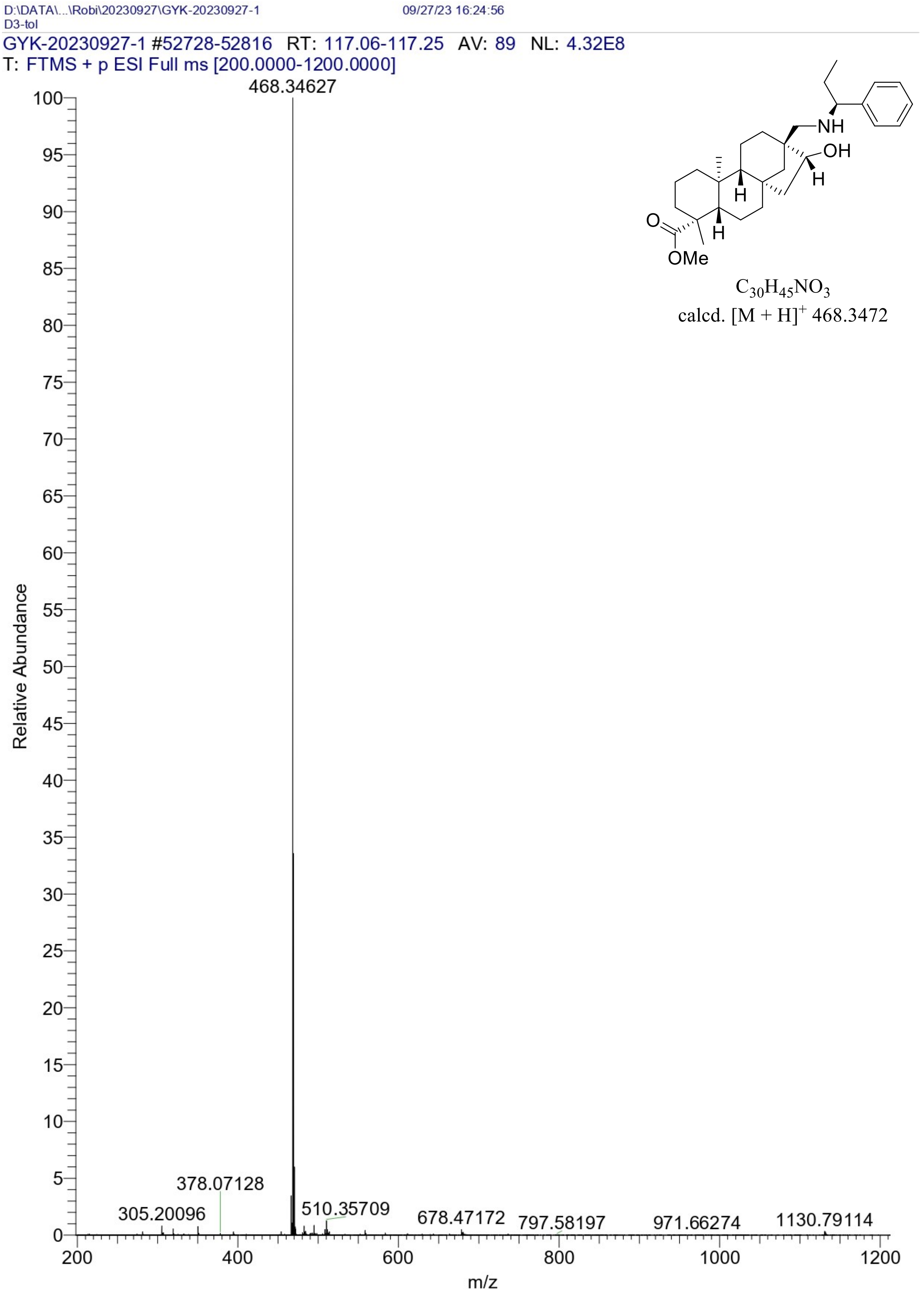

HRMS of compound (4*R*,4a*S*,6a*R*,8*R*,9*S*,11a*R*,11b*S*)-methyl 8-hydroxy-4,11b-dimethyl-9-(((naphthalen-1-ylmethyl)amino)methyl)tetradecahydro-6a,9-methanocyclohepta[a]naphthalene-4-carboxylate **(26)**

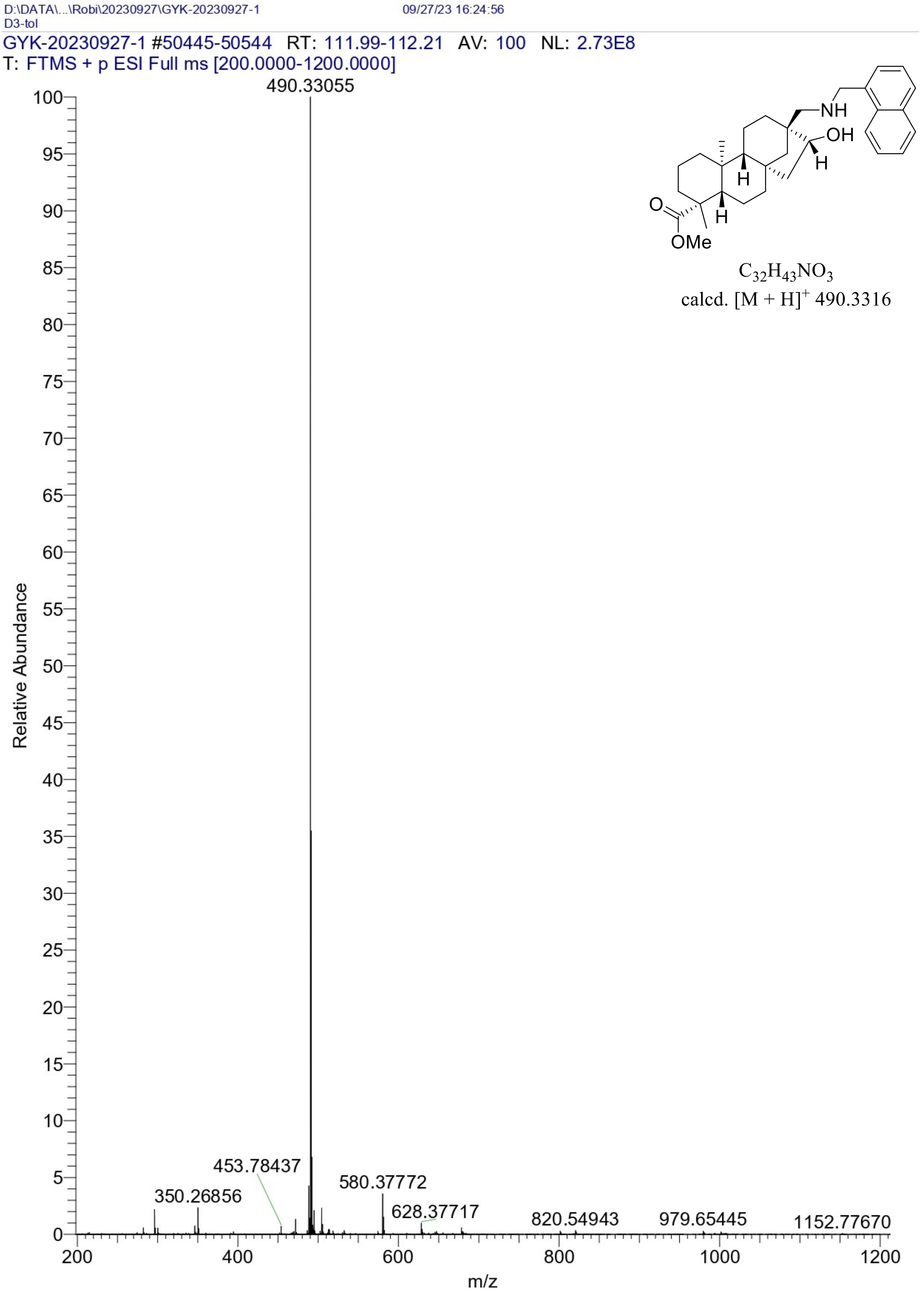

HRMS of compound (4*R*,4a*S*,6a*R*,8*R*,9*S*,11a*R*,11b*S*)-methyl 8-hydroxy-4,11b-dimethyl-9-((((*S*)-1-(naphthalen-1-yl)ethyl)amino)methyl)tetradecahydro-6a,9-methanocyclohepta[a]naphthalene-4-carboxylate **(27)**

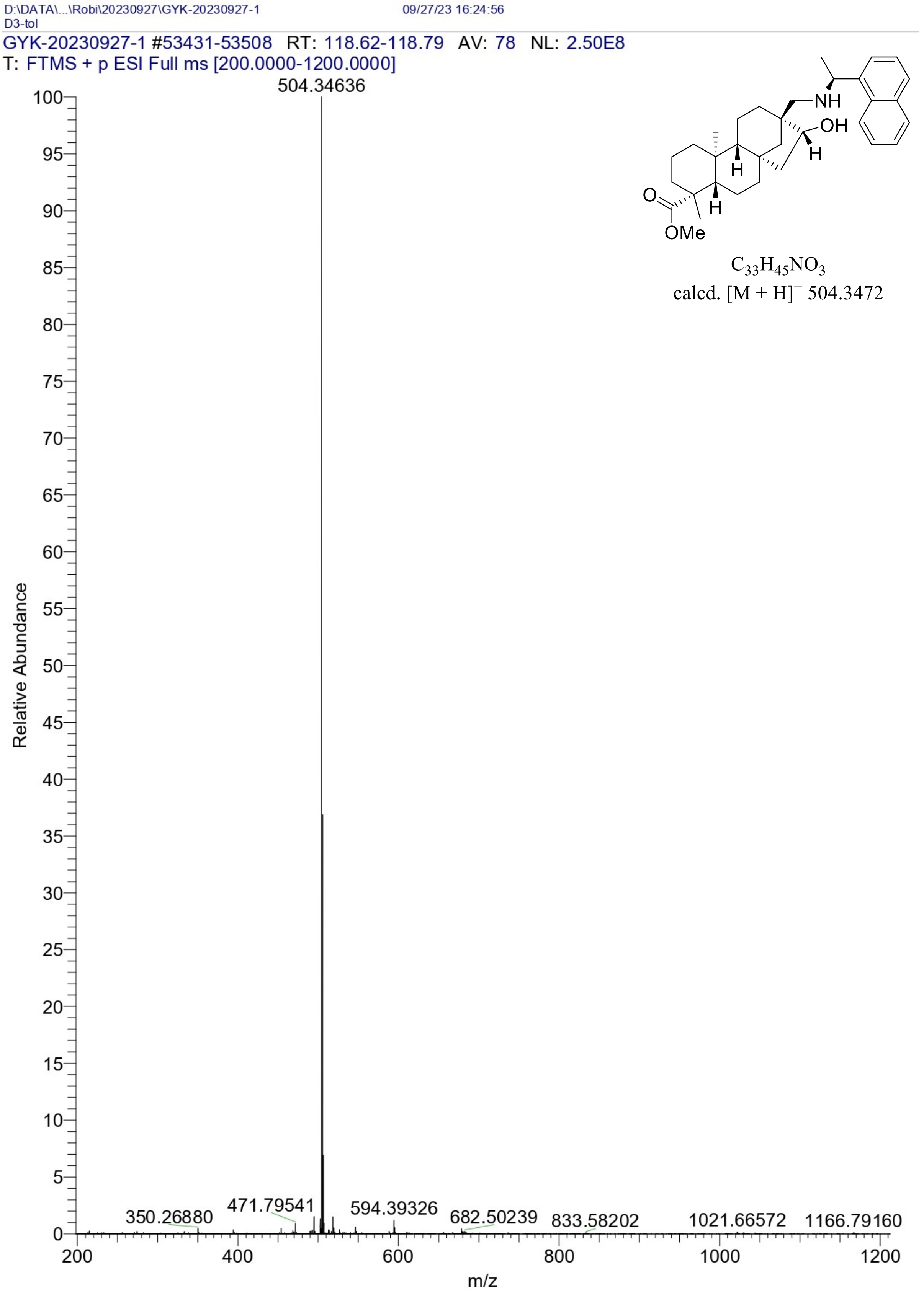

$C_{33}H_{45}NO_3$

calcd. $[M + H]^+$ 504.3472

HRMS of compound (4*R*,4a*S*,6a*R*,8*R*,9*S*,11a*R*,11b*S*)-methyl 8-hydroxy-4,11b-dimethyl-9-((((*R*)-1-(naphthalen-1-yl)ethyl)amino)methyl)tetradecahydro-6a,9-methanocyclohepta[a]naphthalene-4-carboxylate **(28)**

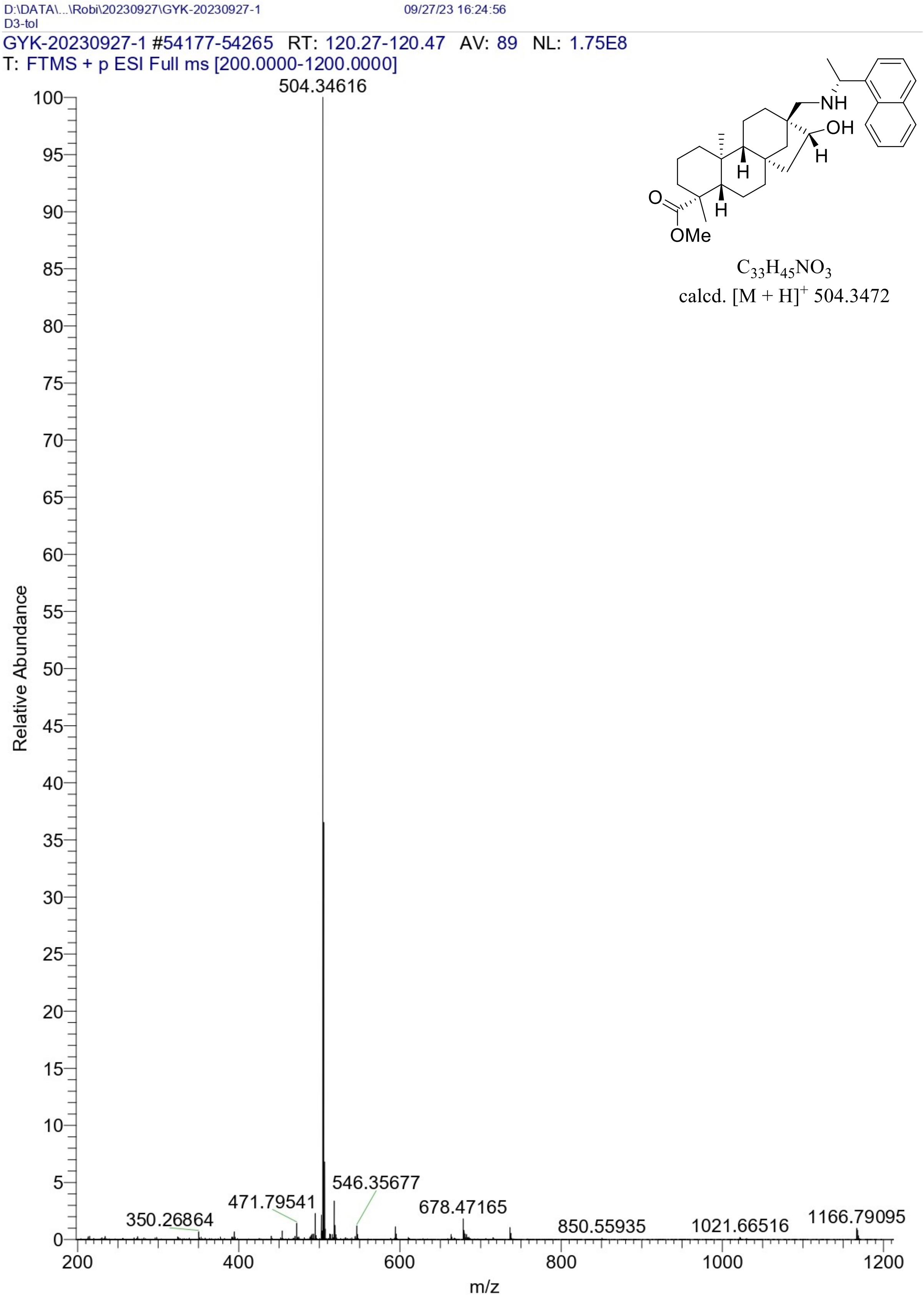

$C_{33}H_{45}NO_3$

calcd. $[M + H]^+$ 504.3472

HRMS of compound (4*R*,4a*S*,6a*R*,8*R*,9*S*,11a*R*,11b*S*)-methyl 8-hydroxy-4,11b-dimethyl-9-((((*R*)-1-(naphthalen-2-yl)ethyl)amino)methyl)tetradecahydro-6a,9-methanocyclohepta[a]naphthalene-4-carboxylate **(29)**

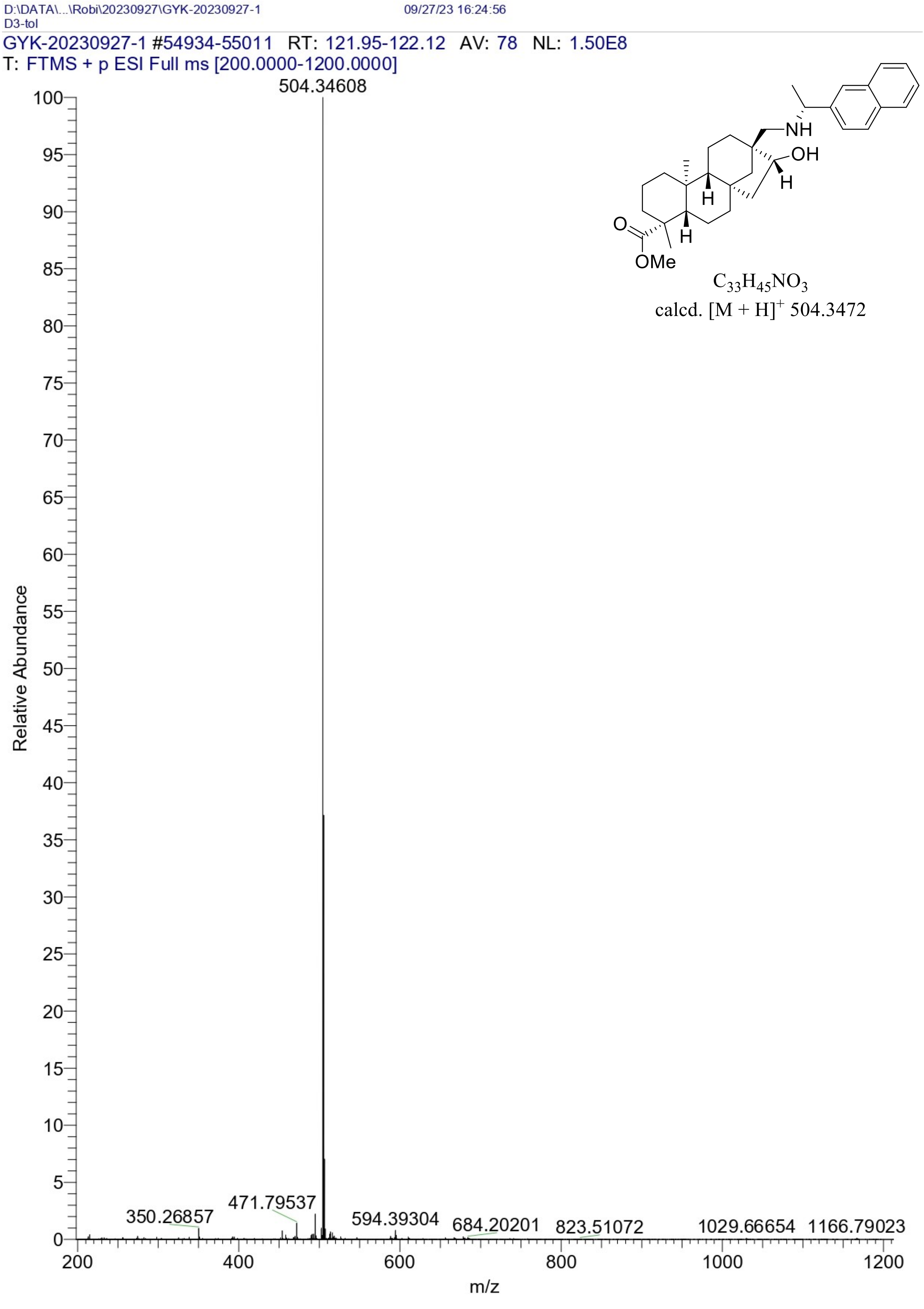

HRMS of compound (4*R*,4a*S*,6a*R*,8*R*,9*S*,11a*R*,11b*S*)-methyl 8-hydroxy-4,11b-dimethyl-9-((((*S*)-1-(naphthalen-2-yl)ethyl)amino)methyl)tetradecahydro-6a,9-methanocyclohepta[a]naphthalene-4-carboxylate **(30)**

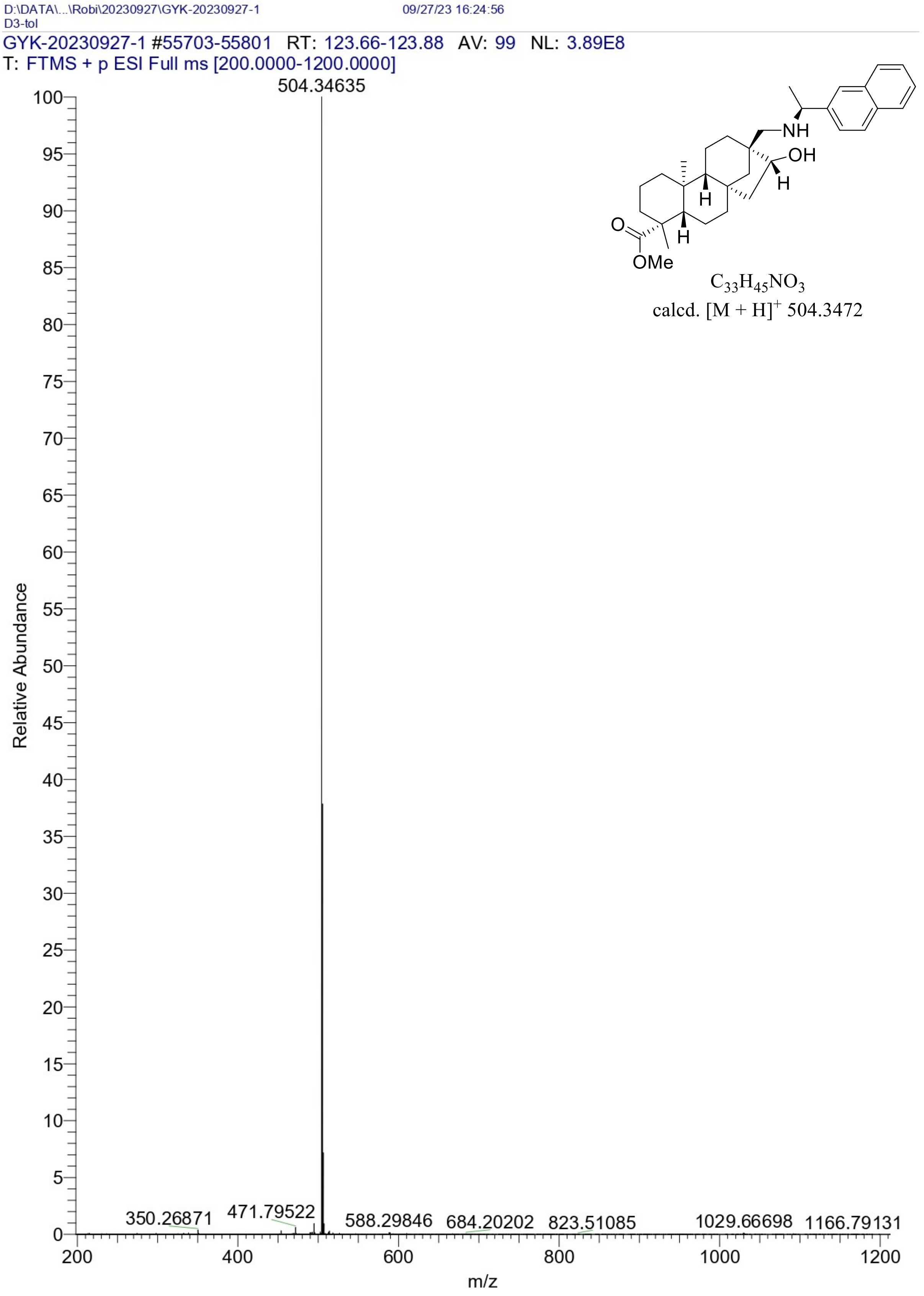

HRMS of compound (4*R*,4a*S*,6a*R*,8*R*,9*S*,11a*R*,11b*S*)-methyl 9-(aminomethyl)-8-hydroxy-4,11b-dimethyltetradecahydro-6a,9-methanocyclohepta[a]naphthalene-4-carboxylate **(31)**

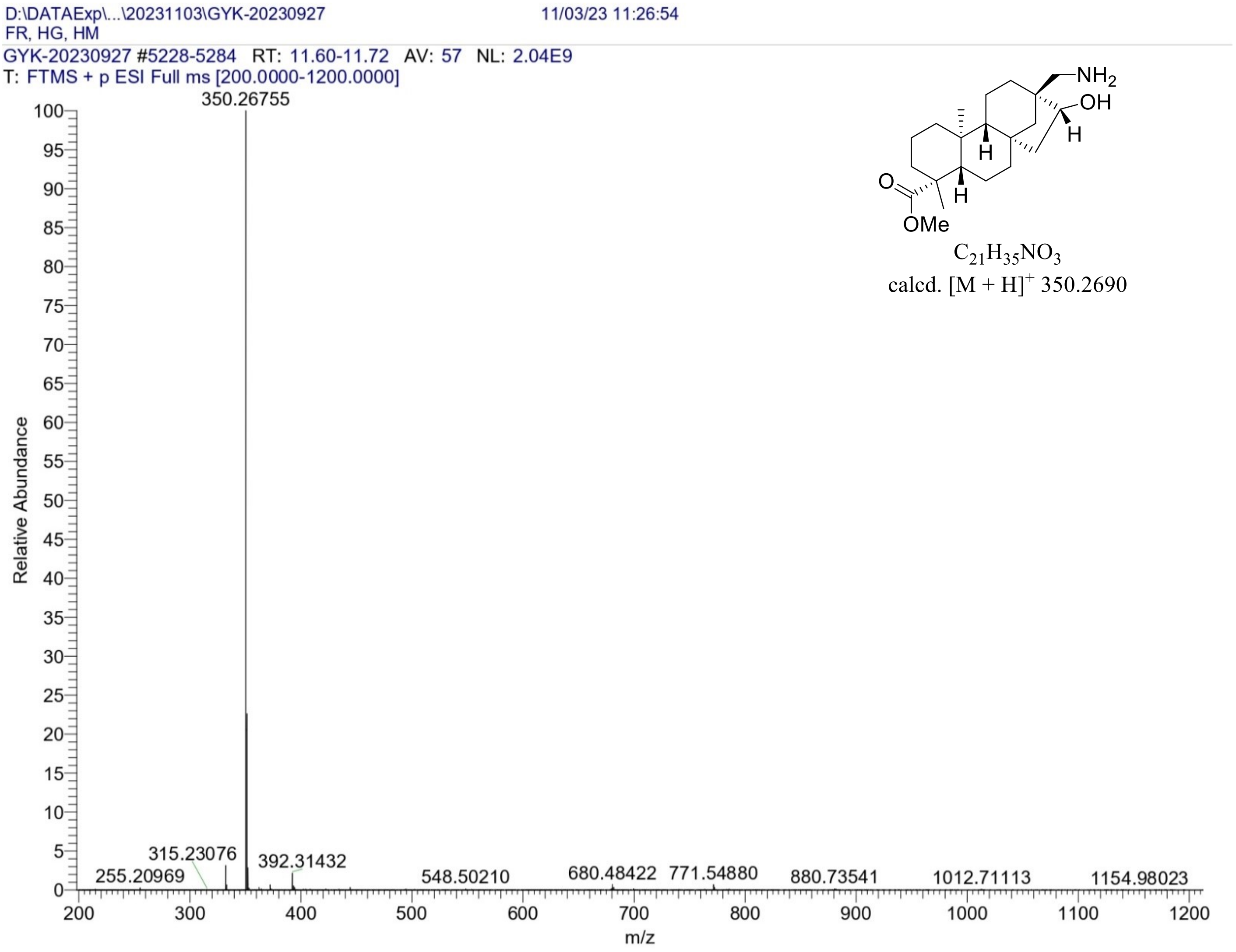

HRMS of compound (4*R*,4a*S*,6a*R*,8*R*,9*S*,11a*R*,11b*S*)-methyl 8-hydroxy-9-(((4-methoxybenzyl)amino) methyl)-4,11b-dimethyltetradecahydro-6a,9-methanocyclohepta[a]naphthalene-4-carboxylate **(32)**

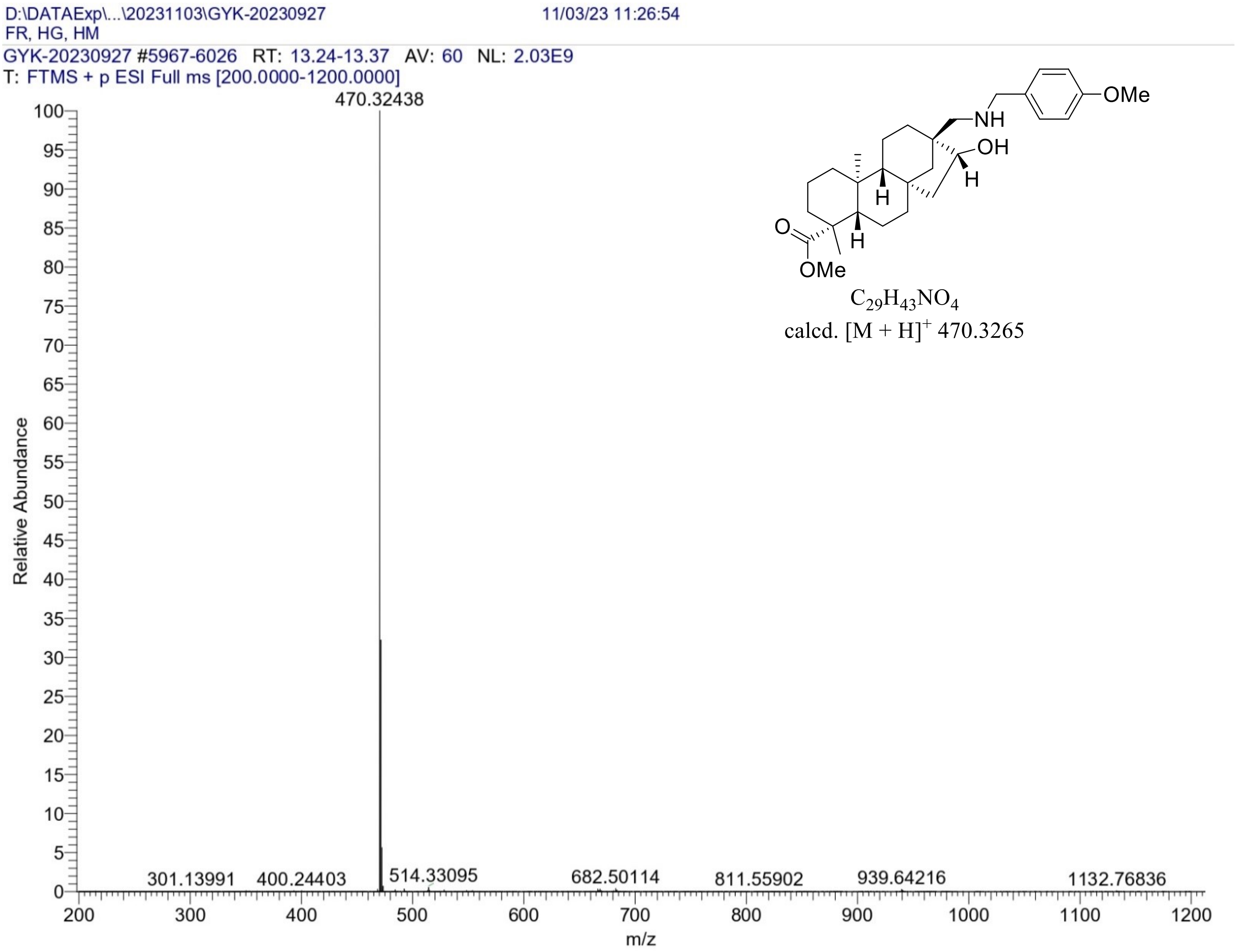

HRMS of compound (4*R*,4a*S*,6a*R*,9*S*,11a*R*,11b*S*)-methyl 4,11b-dimethyl-8-oxo-9-((4-phenyl-1*H*-1,2,3-triazol-1-yl)methyl)tetradecahydro-6a,9-methanocyclohepta[a]naphthalene-4-carboxylate **(33)**

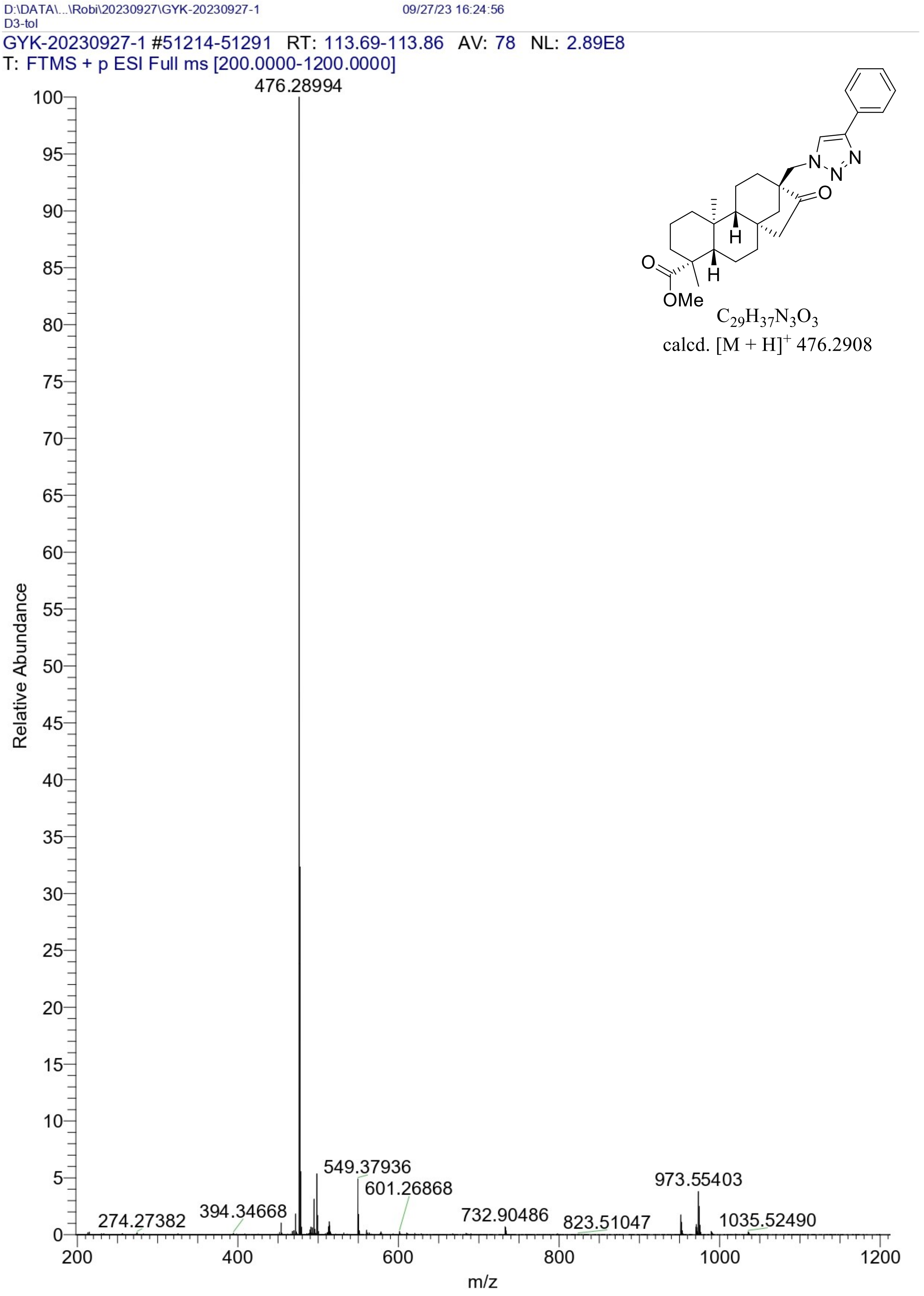

HRMS of compound (4*R*,4a*S*,6a*R*,9*S*,11a*R*,11b*S*)-methyl 9-((4-benzyl-1*H*-1,2,3-triazol-1-yl)methyl)-4,11b-dimethyl-8-oxotetradecahydro-6a,9-methanocyclohepta[a]naphthalene-4-carboxylate **(34)**

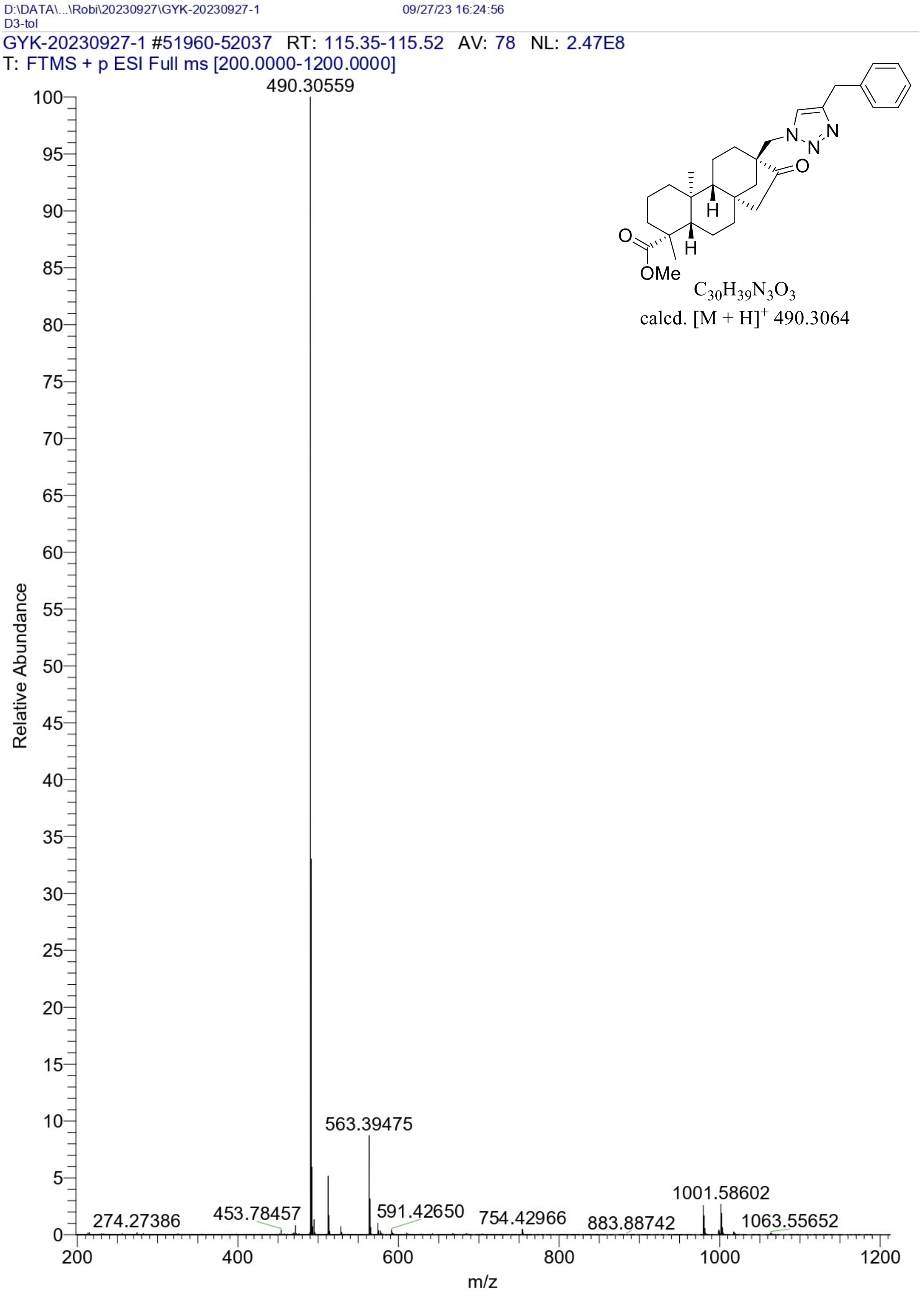

HRMS of compound (4*R*,4a*S*,6a*R*,8*R*,9*S*,11a*R*,11b*S*)-methyl 8-hydroxy-4,11b-dimethyl-9-((4-phenyl-1*H*-1,2,3-triazol-1-yl)methyl)tetradecahydro-6a,9-methanocyclohepta[a]naphthalene-4-carboxylate **(35)**

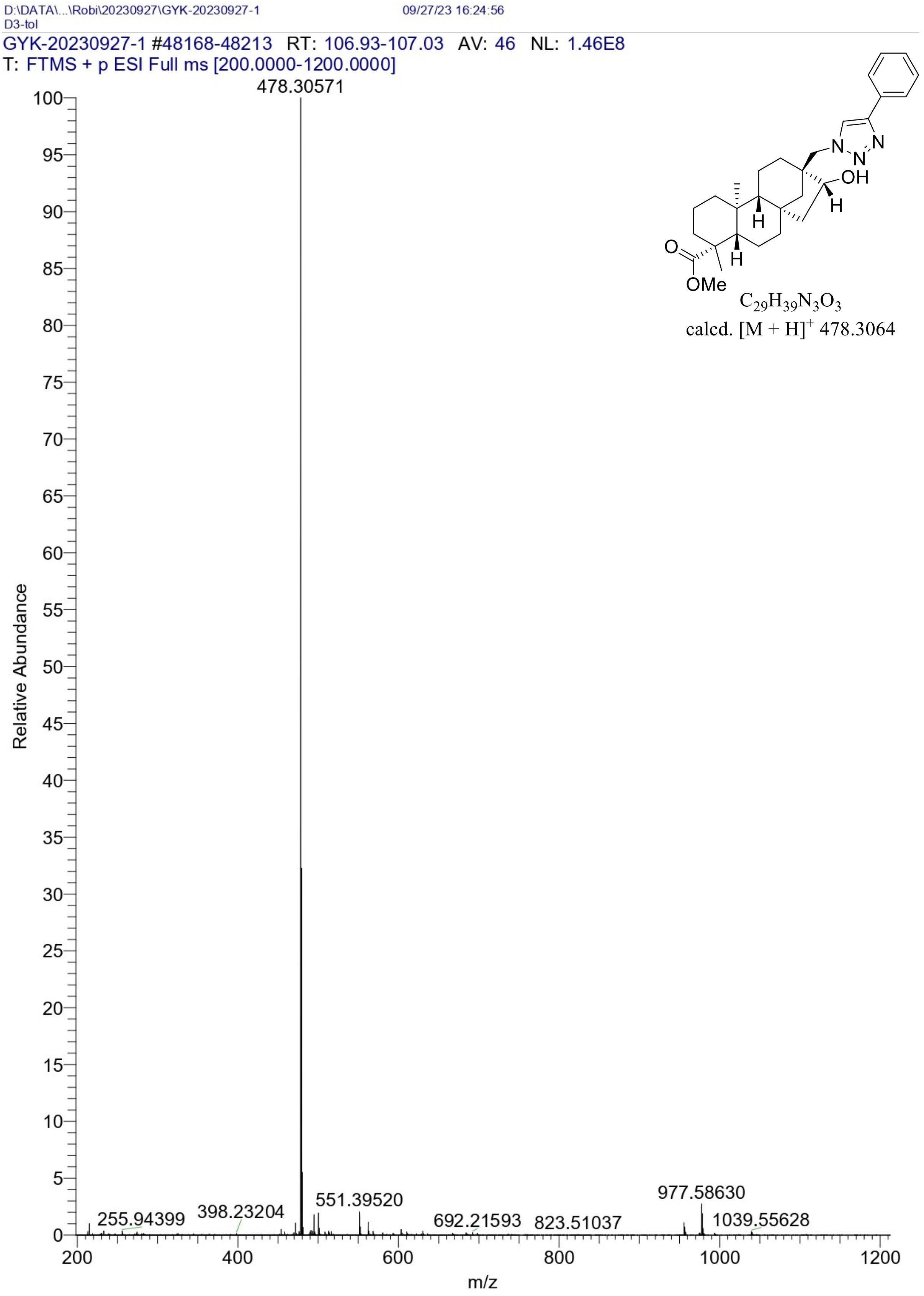

$C_{29}H_{39}N_3O_3$

calcd. $[M + H]^+$ 478.3064

HRMS of compound (4*R*,4a*S*,6a*R*,8*R*,9*S*,11a*R*,11b*S*)-methyl 9-((4-benzyl-1*H*-1,2,3-triazol-1-yl)methyl)-8-hydroxy-4,11b-dimethyltetradecahydro-6a,9-methanocyclohepta[a]naphthalene-4-carboxylate **(36)**

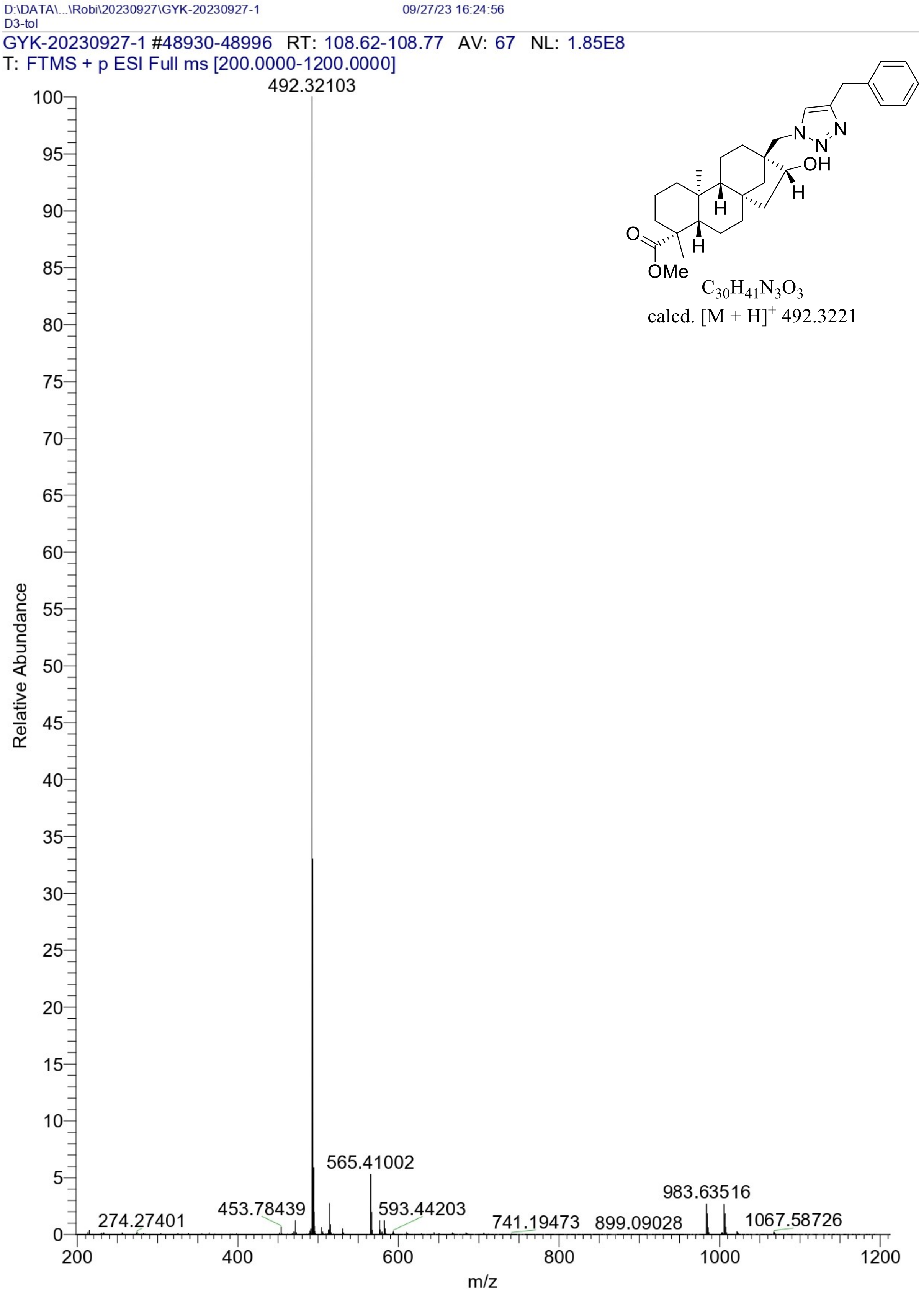

Supplement: Supplementary file 1 [file molecules-28-07962-s001.zip › molecules-2727575-supplementary.pdf]
